# Trialkylammonium salt degradation: implications for methylation and cross-coupling†

**DOI:** 10.1039/d1sc00757b

**Published:** 2021-04-13

**Authors:** Jack B. Washington, Michele Assante, Chunhui Yan, David McKinney, Vanessa Juba, Andrew G. Leach, Sharon E. Baillie, Marc Reid

**Affiliations:** WestCHEM Department of Pure and Applied Chemistry, University of Strathclyde, Thomas Graham Building 295 Cathedral Street Glasgow UK marc.reid.100@strath.ac.uk; School of Pharmacy and Biomolecular Sciences, Liverpool John Moores University Byrom Street Liverpool UK; Division of Pharmacy and Optometry, University of Manchester Stopford Building Oxford Road Manchester UK; GlaxoSmithKline Shewalton Road Irvine North Ayrshire UK

## Abstract

Trialkylammonium (most notably *N*,*N*,*N*-trimethylanilinium) salts are known to display dual reactivity through both the aryl group and the *N*-methyl groups. These salts have thus been widely applied in cross-coupling, aryl etherification, fluorine radiolabelling, phase-transfer catalysis, supramolecular recognition, polymer design, and (more recently) methylation. However, their application as electrophilic methylating reagents remains somewhat underexplored, and an understanding of their arylation *versus* methylation reactivities is lacking. This study presents a mechanistic degradation analysis of *N*,*N*,*N*-trimethylanilinium salts and highlights the implications for synthetic applications of this important class of salts. Kinetic degradation studies, in both solid and solution phases, have delivered insights into the physical and chemical parameters affecting anilinium salt stability. ^1^H NMR kinetic analysis of salt degradation has evidenced thermal degradation to methyl iodide and the parent aniline, consistent with a closed-shell S_N_2-centred degradative pathway, and methyl iodide being the key reactive species in applied methylation procedures. Furthermore, the effect of halide and non-nucleophilic counterions on salt degradation has been investigated, along with deuterium isotope and solvent effects. New mechanistic insights have enabled the investigation of the use of trimethylanilinium salts in *O*-methylation and in improved cross-coupling strategies. Finally, detailed computational studies have helped highlight limitations in the current state-of-the-art of solvation modelling of reaction in which the bulk medium undergoes experimentally observable changes over the reaction timecourse.

## Abstract 2

The dual reactivity of *N*,*N*,*N*-trimethylanilinium salts towards arylation and methylation is decoupled in this mechanistic investigation to enable more strategic application of these salts in either reaction class.

## Graphical abstract

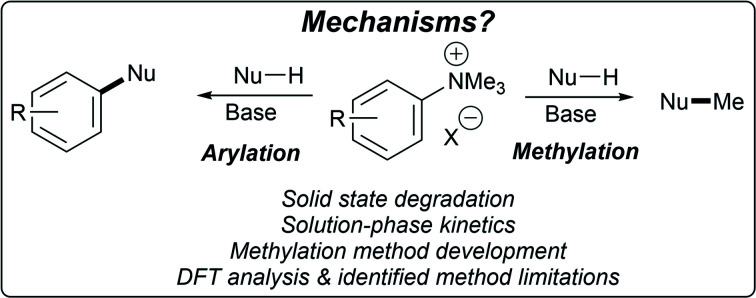

## Introduction

### Applications and divergent reactivity of trialkylammonium salts

Trialkylammonium (specifically *N*,*N*,*N*-trimethylanilinium) salts have found wide-ranging applications in synthesis, spanning phase-transfer catalysis,^1^ supramolecular ion-pairing catalysis,^2,3^ host-guest binding studies,^4^*O*-methylation,^5^*O*-arylation,^6^ heteroatom arylations,^7^ C–H methylation,^8^*C*-arylation,^9–11^ fluorine radiolabeling,^12–15^ organometallic ligand design,^16–21^ antimicrobial polymer design,^22^ arene reduction,^23^ pH sensing,^24^ and a range of metal-catalysed cross-coupling methodologies.^25–32^ The dichotomy of arylation *versus* methylation reactivity, while important for optimising the above-listed applications, is rarely studied in detail. Understanding the dual reactivity of *N*,*N*,*N*-trimethylanilinium salts thus serves as the focus of our study.

**Fig. 1:**
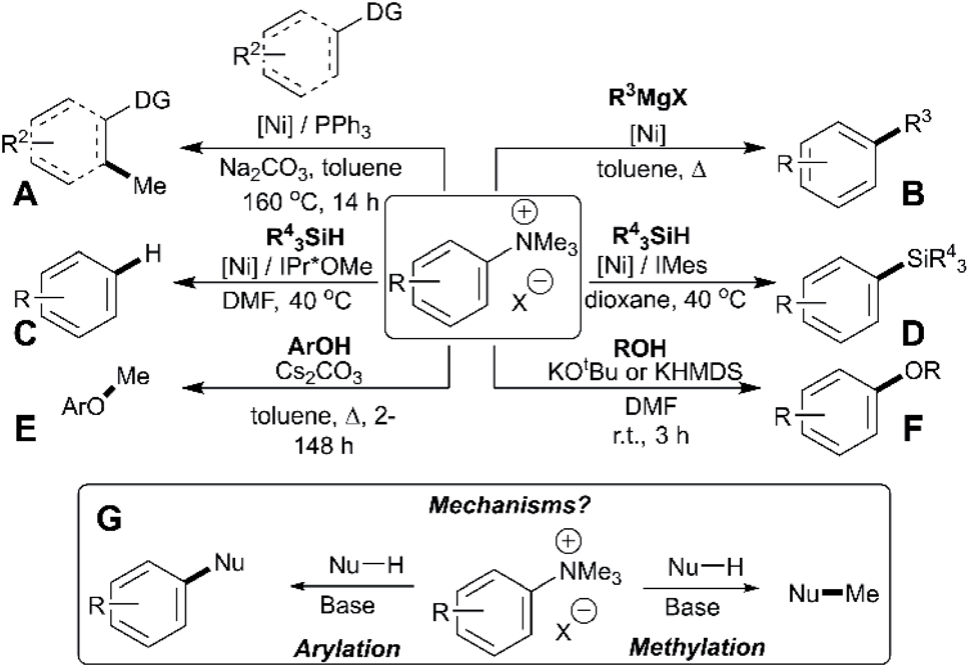
Demonstration of dichotomous reactivity of trimethylanilinium salts in: (A) C–H methylation,^8^ (B) Kumada coupling,^9^ (C) arene reduction,^23^ (D) silylation,^23^ (E) phenol methylation,^5^ and (F) alcohol arylation.^6^ Part (G) summarises the dual reactivity of trimethylanilinium salts and the key mechanistic problem investigated in this study.

Arylation methodologies are arguably the most widely studied of the above-listed anilinium salt applications. Pioneering developments by Wenkert *et al.* involved a nickel-catalysed Kumada-type coupling between Grignard reagents and trimethylanilinium iodides to generate biaryls *via* C–C bond formation (Fig. 1, Part B).^9^ Later developments from Reeves used trimethylanilinium triflates and aryl Grignard reagents with a palladium catalyst to produce functionalised biaryl motifs in high yields under mild conditions.^10^ Similarly, MacMillan *et al.* found that trimethylanilinium triflates were suitable coupling partners with arylboronic acids in Suzuki reactions.^11^ Other notable examples where trimethylanilinium salts have been used as electrophilic coupling partners include Negishi coupling,^33^ borylation,^34,35^ amination,^36^ and azole arylation.^37^ In contrast to the wealth of transition-metal catalysed arylation reactions using anilinium salts, Chatani's team provided a rare demonstration that *N*,*N*,*N*-trimethylanilinium iodide can be used to transfer a methyl (as opposed to phenyl) group in nickel-catalysed C(sp^2^)–H and C(sp^3^)–H bond formations (Fig. 1, Part A).^8^ Pioneering nickel-catalysed arene reduction methods from Rand and Montgomery^23^ showed that ligand and solvent variations could be exploited to enable either C–H or C–Si bond formation from the same anilinium salt source (Fig. 1, Parts C and D). Base-mediated reactions with anilinium salts display a similar dichotomy in arylation *versus* methylation (Fig. 1, Parts E and F). To optimise the predictable use of trimethylanilinium salts in synthesis, a structured mechanistic analysis of their arylation and methylation reactivities is required. Herein, we present our investigation of the factors governing this dichotomous reactivity (Fig. 1, Part G).

### Drivers towards understanding the methylating ability of anilinium salts

The presence or absence of small methyl groups in organic molecules can bring about large changes in physicochemical properties.^38,39^ This has been a particular focus in drug design where the installation of methyl groups can have drastic effects on solubility, potency and selectivity; this contested phenomenon is dubbed the magic methyl effect.^40,41^ As such, the present study to understand trimethylanilinium salts is, in part, an answer to the call for new methylation reactions.^40^ Parallel to drug design, formal access to highly toxic electrophilic methyl halide reagents *in situ via* anilinium salts may provide safer alternatives to more widely known carcinogenic and low-boiling methylating reagents.^42^

**Fig. 2:**
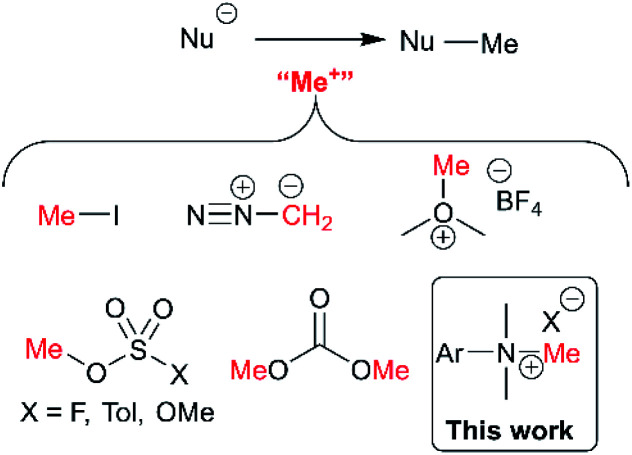
Commonly used electrophilic methylating reagents and the focus of this study to understand the place of trimethylanilinium salts among them.

Of the various classes of methylating reagent available,^43–46^ electrophilic sources still represent a significant tool for synthetic chemists (Fig. 2). As with all reagents, toxicity, safety, scalability, and operational difficulty play a key role in the decision to use or replace said reagent.^42^ Within the electrophilic class of methylating reagents, quaternary ammonium salts are somewhat underexplored.^44^ Early examples applied tetraalkylammonium and trimethylanilinium hydroxides and halides to achieve heteroatom-alkylations.^5,47–50^ From the patent literature, anilinium salts have been used to achieve *O*-methylation in alkaloids.^51,52^

To the above-mentioned design and safety-focussed ends, methods that allow for methylation of a substrate in a straight-forward, safe, and predictable manner are highly desirable. Simultaneously, this directed investigation of trimethylanilinium salts, and specifically their degradation behaviour, can provide strategic insights for the myriad of aforementioned applications of these salts.

## Results & discussion

### Solid phase degradation analysis

**Fig. 3:**
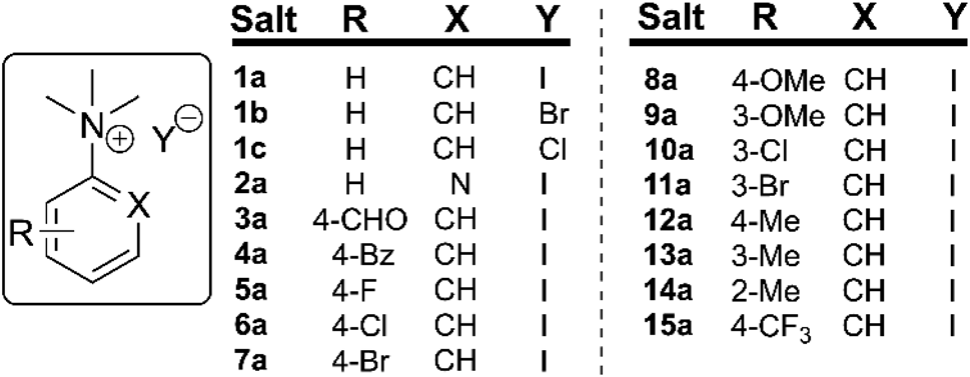
Library of *N*,*N*,*N*-trimethylarylammonium (phenyl and pyridyl) salts used in mechanistic degradation studies. Further anionic variations are also considered.

To understand the potential for long-term safe storage of trimethylanilinium salts, and knowing the potential for reagent storage to cause adverse degradative effects prior to their use in synthesis,^53^ our studies began with an investigation of the solid-state stability of arylammonium halides (primarily aniline derivatives). Salts **1a–1c** and **2a–15a** composed the core structural library employed in our investigations (Fig. 3). Details on the synthesis and availability of all salts are available in the Experimental ESI (ESI; Section 3).†

**Fig. 4:**
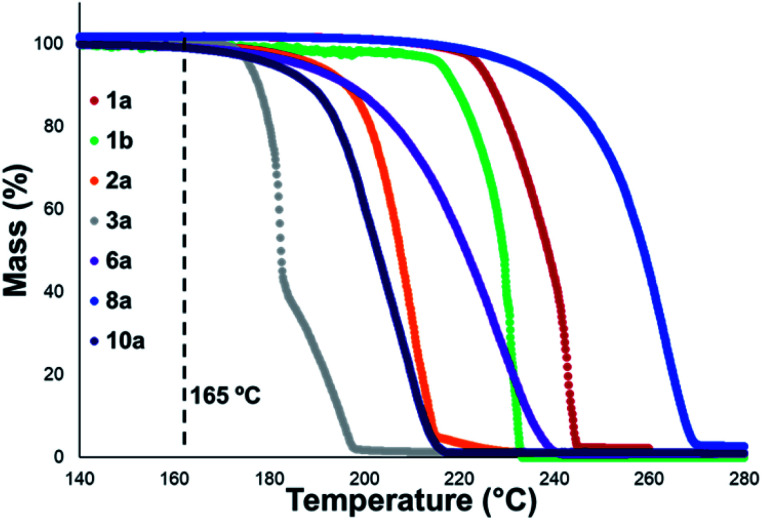
Mass *vs.* temperature trace determined *via* thermal gravimetric analysis (TGA) for an illustrative subset of the salt library, analysed under an argon atmosphere. Temperature range: 40–300 °C. Temperature ramp: 10 °C min^−1^. Temperatures between 40–139 °C and >280 °C have been omitted as no observable change in mass was observed for any of the salts in these temperature ranges. Single-step degradation profiles are indicative of sublimation.

Thermal gravimetric analysis (TGA) was used to estimate relative thermal stabilities of the salt library over 40–300 °C under both argon and air atmospheres. TGA traces for the argon experiments are shown in Fig. 4. Detailed onset and peak degradation temperatures are shown in the Experimental ESI Section 4.† All salts tested were stable up to 165 °C, suggesting that storage of these compounds at room temperature would be viable. TGA traces in air were comparable to TGA results obtained under argon.

**Fig. 5:**
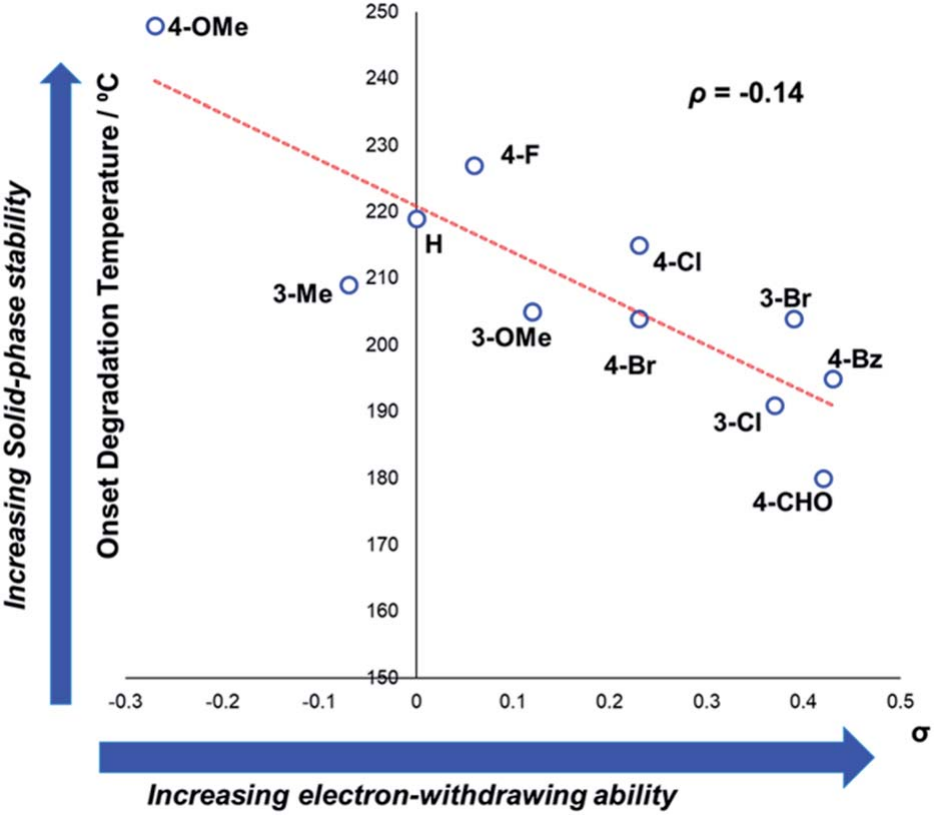
TGA analysis for the degradation of anilinium iodide salts, supporting the role of electron-withdrawing aryl substituents in promoting thermal degradation. Additional analyses for samples degraded under air can be found in the Experimental ESI, Section 4.†^54^

In general, compounds with more electron deficient aryl groups were more susceptible to thermal degradation (Fig. 5). All salts, with the exception of **1c** (unsubstituted chloride), **3a** (4-CHO) and **4a** (4-Bz), showed a smooth, single-step TGA trace, potentially indicative of sublimation. It seemed plausible that the main salt decomposition products would be the parent functionalised amino arene and its respective methyl halide, arising from a retro-Menshutkin reaction.^54^ This hypothesis was consistent with the observation that more electron-poor salts degraded at lower temperatures than comparitively electron rich salts. Complementary thermal volumetric analysis (TVA)^55^ coupled with a sub-ambient distillation apparatus (TVA-SAD) was used to monitor the decomposition of anilinium iodide salts **1a** (unsubstituted iodide), **7a** (4-Br) and **12a** (4-Me). Alongside infrared (IR) analysis, the TVA-SAD studies provided evidence that methyl iodide was generated during the thermal decomposition of these trimethylanilinium iodide salts (see Experimental ESI Section 5†). It is important to note that the relative position of an electrophilic N–Me group and a halide counterion arising from crystal packing in the solid state could contribute to different relative thermal stabilities.

### Single-point solution phase degradation studies – substituent and anion effects

**Fig. 6:**
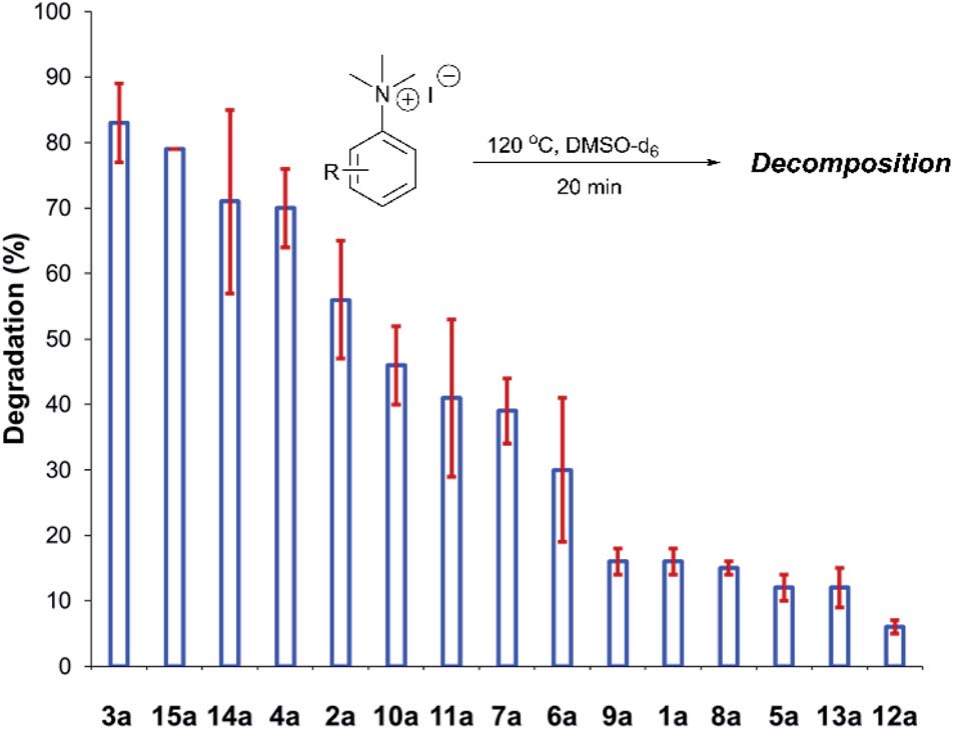
Relative degradation of a range of *N*,*N*,*N*-trimethylanilinium iodides upon heating in DMSO-d_6_ (0.06 M) at 120 °C for 20 min; 1,2,4,5-tetramethylbenzene was used as an internal standard to calculate the concentration of the anilinium salt before and after heating. Results and associated errors are calculated from triplicate runs.

**Fig. 7:**
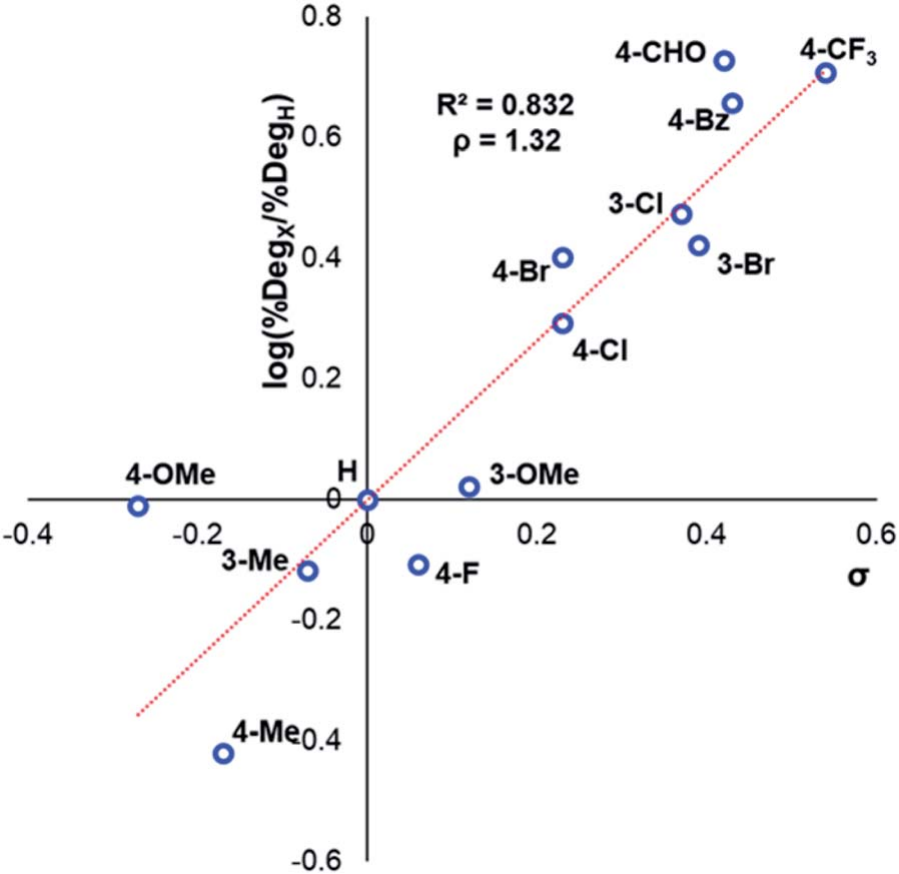
Hammett analysis of solution-phase degradation experiments shown in Fig. 5, supporting the approximation that more electron-deficient ring substituents accelerate anilinium iodide degradation by demethylation as the predominant pathway.

After investigating the stability of the *N*,*N*,*N*-trimethylanilinium halide library in the solid state, our attention turned to investigating their degradation behaviour in solution. DMSO was strategically chosen as a solvent due to its ability to solubilise the salts, enabling homogeneous ^1^H NMR kinetic analysis. Additionally, the high boiling point of DMSO allowed for flexible temperature studies. This solvent also links to earlier NMR kinetics investigations involving methyl iodide^56^ and DMSO-mediated methylation studies.^57,58^ Importantly, detailed studies applying solvents applicable to a broader range of methylation and catalytic chemistries is also included (see Experimental ESI Sections 20 and 22†). A graph showing the extent of degradation of each salt after a 20 min period is shown in Fig. 6. Consistent with TGA analysis, electron-poor salts were the most susceptible to thermal degradation in DMSO (Fig. 7); salts with a predominantly resonance electron-withdrawing 4-substituent, 4-formyl (**3a**) and 4-benzoyl (**4a**), were shown to degrade by 83 ± 6% and 70 ± 6%, respectively, and significantly more so than the unsubstituted iodide **1a** at 16 ± 2%. Salts containing 4-chloro (**6a**; 30 ± 11%) and 4-bromo (**7a**; 39 ± 5%) substituents were only slightly destabilised with respect to **1a**, while 4-methyl (**12a**; 6 ± 1%) was more stable than **1a**. Halogen substituents have inductive electron withdrawing effects which would destabilise the anilinium cation, but they also have the competing ability to stabilise positive charge through resonance donation;^59^ the trend in decreasing stability from **5a** to **7a** (4-F > 4-Cl > 4-Br) is consistent with decreasing resonance donation down the halogen group. Conversely, 4-CF_3_ (**15a**; 79 ± 0%) shows that a strong inductive electron-withdrawing effect significantly destabilises the anilinium salt *versus* the unsubstituted **1a**. 2-Me (**14a**; 71 ± 1%) shows high anilinium salt degradation over 20 min, and can presumably be attributed to the steric encumberment of the 2-Me and resulting destabilisation of the reactive quaternary nitrogen centre.

**Fig. 8:**
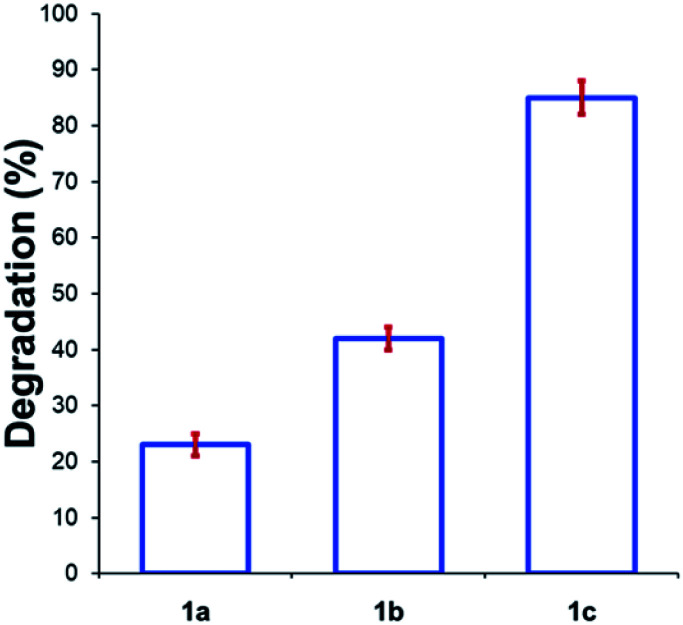
Degradation of **1a** (X = I), **1b** (X = Br), and **1c** (X = Cl) upon heating in DMSO-d_6_ (0.1 M) at 120 °C for 20 min. Results and errors are taken from triplicate runs.

Beyond substitution effects of the aromatic moiety on *N*,*N*,*N*-trimethylanilinium iodide stability, we next investigated the counter-anion effect on solution-phase stability. The degradations of *N*,*N*,*N*-trimethylanilinium iodide (**1a**), bromide (**1b**), and chloride (**1c**) were analysed after heating to 120 °C for 20 min in DMSO-d_6_ (Fig. 8). A clear trend emerged where degradation was more advanced after 20 min in **1c** (chloride; 85 ± 2%) than **1b** (bromide; 42 ± 2%) and **1a** (iodide; 23 ± 3%). This is consistent with the reported order of halide nucleophilicity in polar aprotic solvents.^60,61^ As described below, the reaction in which methyl halide is formed is close to thermoneutrality and thus small changes in the stability of ion pairs and of carbon halide bonds can provide a thermodynamic explanation for the observed order of thermal stability in solution.^12,62^

**Fig. 9:**
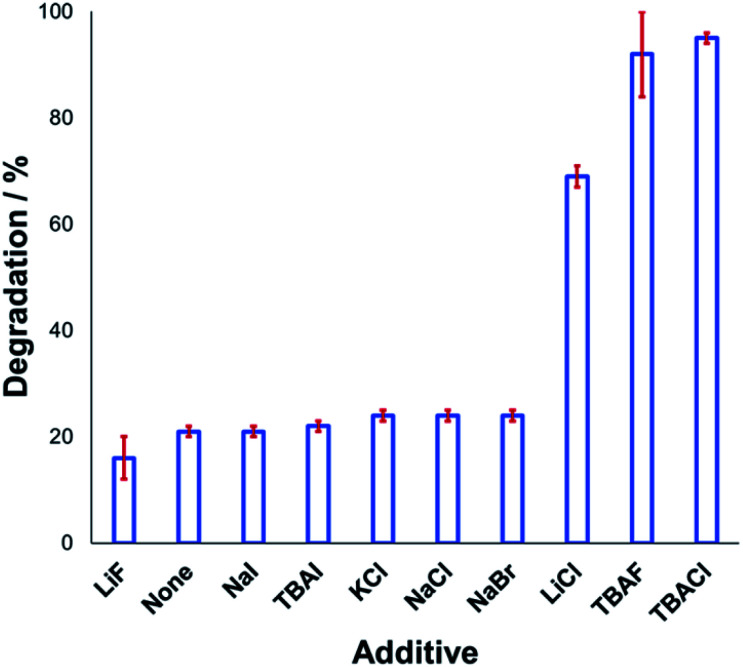
Relative degradation of *N*,*N*,*N*-trimethylanilinium iodide **1a** upon heating in DMSO-d_6_ (0.1 M) at 120 °C for 20 min in the presence of 1 equiv. halide additive. Results and associated error bars taken from triplicate runs.

Understanding the effect of additives alongside salt degradation is relevant for applications of these anilinium salts in cross-coupling methodologies.^11–17^ To this end, an equimolar quantity of selected halide salts was added to solutions of **1a** in DMSO-d_6_ and the degradation measured over 20 min at 120 °C (Fig. 9). Anilinium degradation was only accelerated by 3 of the 10 additives tested, namely LiCl, and tetrabutylammonium (TBA) salts of fluoride and chloride. The more pronounced anilinium cation degradation in the presence of TBACl *versus* LiCl and KCl is tentatively attributed to higher solubility of the former in DMSO. The low solubility of LiF, NaI, NaBr, NaCl, and KCl in DMSO means it also possible that there was a lower-than-calculated (or even negligible) concentration of additive halide ions in solution. Indeed, with these reactions being conducted in NMR tubes, inefficient mixing may only exacerbate any solubility issues. Addition of TBAI (*i.e.* additional iodide beyond that in the anilinium salt itself) proved statistically similar to no additive at all. A related report on fluoride-mediated anilinium degradation has evidenced strong ion-pairing in TBAF that undergoes immediate salt metathesis in the presence of anilinium triflates to afford anilinium fluorides.^12,63^

If ion-pairing indeed dictates preorganisation prior to anilinium degradation, data in Fig. 9 suggest that only the latter three additives contribute significant salt metathesis to form *in situ* ion pairs more prone to degradation than the parent anilinium iodide, **1a**.

**Fig. 10:**
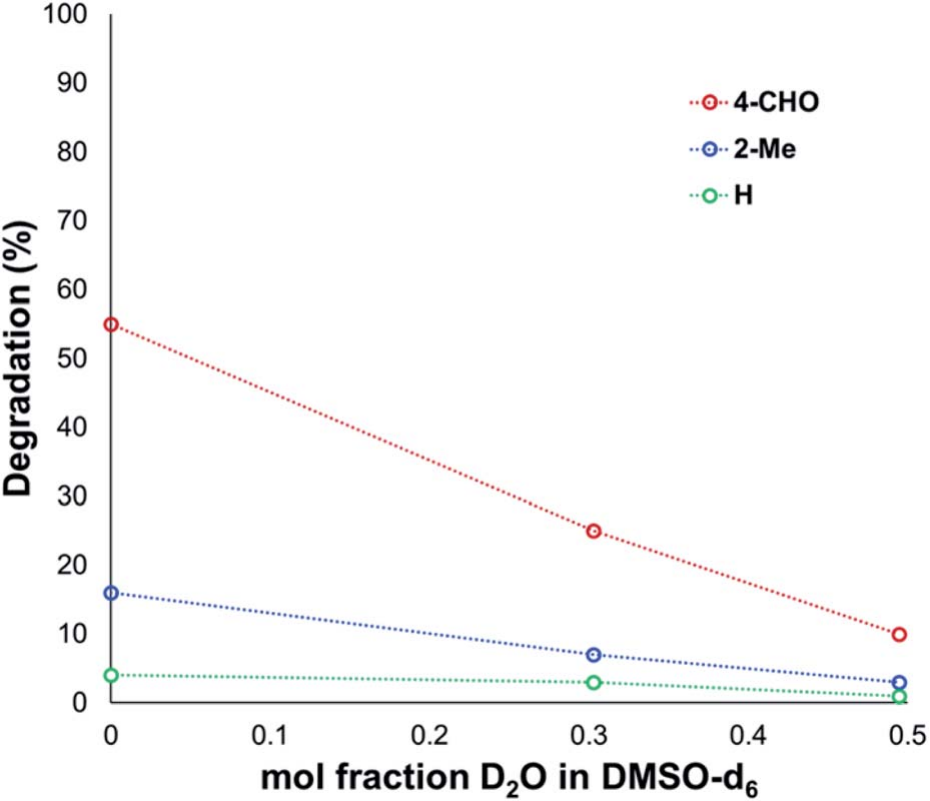
Attenuated degradation of anilinium iodides in the presence of additive concentrations of water. *N*,*N*,*N*-Trimethylanilinium iodides (0.1 M) heated in DMSO-d_6_ with 0, 10 or 20 v/v% D_2_O, at 90 °C for 120 min; maleic acid was used as an internal standard to calculate the concentration of the anilinium salt before and after heating.

Another contributing factor in the apparent banality of LiF comes from its notably hygroscopic nature. In turn, this may have led to the introduction of water into the degradation mixture. A follow-up investigation showed that increasing water concentration – in the presence and absence of TBAF – attenuated anilinium degradation (Fig. 10), likely due to a decrease in halide nucleophilicity through more efficient anion solvation in water *versus* DMSO. Alternatively, the apparent decrease in halide nucleophilicity could also emerge through changes in the equilibrium, caused by enhanced solvation of ionic reactants as opposed to electronically neutral methylhalide and dimethylanilines.^60,64^

Whereas the increased decomposition of the anilinium salts to generate the parent aniline and methyl halide *in situ* may be favourable for use in methylation chemistries, minimising degradation is desired for use of anilinium salts in cross-coupling reactions.^8–11,34–37^ Thus, we expanded our degradation studies to include a range of substituted anilinium salts bearing non-nucleophilic anions, including several anilinium partner anions employed in cross-coupling methodologies.

**Fig. 11:**
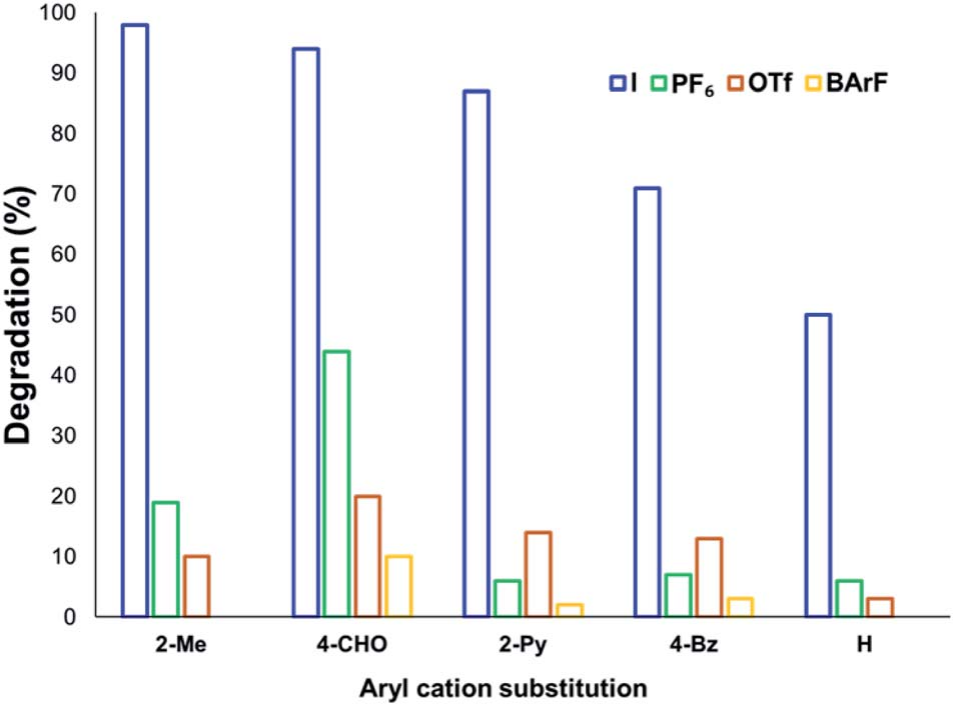
Relative degradation of *N*,*N*,*N*-trimethylanilinium cations partnered with weakly coordinating (less nucleophilic) anions upon heating in DMSO-d_6_ (0.1 M) at 120 °C for 20 min.

The solution-phase thermal degradation of these non-halide anilinium salts are shown in Fig. 11. Non-nucleophilic counterions significantly retarded the decomposition of anilinium salts in solution, particularly when using tetrakis(3,5-bis(trifluoromethyl)phenyl)borate (BArF^−^) salts which proved even more thermally stable than the commonly-applied triflate salts.

### NMR degradation kinetics

**Fig. 12:**
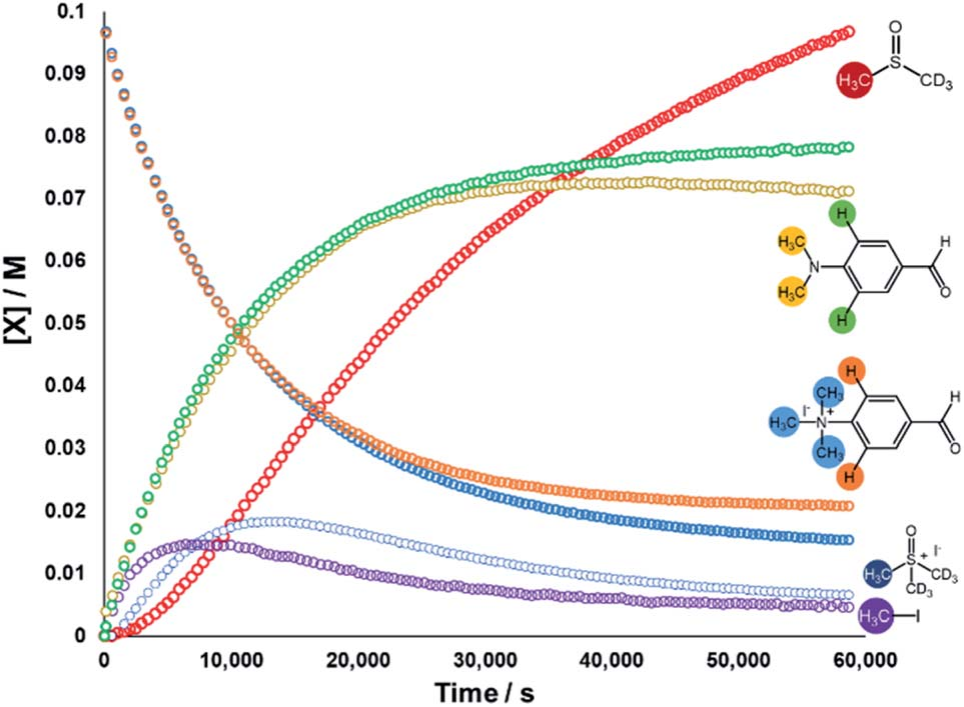
^1^H NMR time course for the degradation of **3a**. Conditions: **3a** (0.1 M) in DMSO-d_6_ (0.6 mL), 1,2,4,5-tetramethylbenzene as internal standard (0.06 M; not plotted) at 80 °C. Lines are colour-coded according to the proton shift followed, as indicated on the figure. Methyl transfer and isotopic scrambling were trackable.

We next sought to monitor the anilinium iodide degradation reaction time course to gain a richer insight into the active mechanism(s) of degradation. From a practical perspective, we hypothesised that this additional insight could enable practitioners to use anilinium salts as a controlled source of methyl iodide generated *in situ*. Again, ^1^H NMR spectroscopy was employed to quantify reaction data. 4-Formyl-*N*,*N*,*N*-trimethylanilinium iodide (**3a**), was selected as an appropriate salt to exemplify reaction kinetics since its degradation was significantly advanced (approximately three half-lives over 16 hours) to capture relevant mechanistic information in a feasible timescale. Additionally, the formyl proton on **3a** was easily identifiable in NMR spectra due its isolated downfield chemical shift. The concentration of various species were calculated against the internal standard (Fig. 12).

The depletion of **3a** could be monitored over time by following either the formyl, –NMe_3_^+^, and 2-aryl protons. Similarly, the evolution of 4-formyl-*N*,*N*-dimethylaniline could be seen by following the analogous –NMe_2_, formyl and 2-aryl protons. Furthermore, the evolution of methyl iodide (*d*_0_) could be observed in the same experiment. Interestingly, the calculated concentration of both the anilinium or aniline *via* aromatic and formyl protons, while being equal, were each greater than the concentration calculated *via* the *N*-methyl groups (orange *vs.* blue, and green *vs.* yellow lines in Fig. 12). This is suggestive of isotopic scrambling of N–CH_3_ for N–CD_3_ groups *via* interaction with the evidently non-innocent DMSO-d_6_ solvent. Deuterium scrambling is consistent with the emergence of trimethyl sulfoxonium (d_6_,h_3_) as a key intermediate, and DMSO-d_3_ as a final product.

**Fig. 13:**
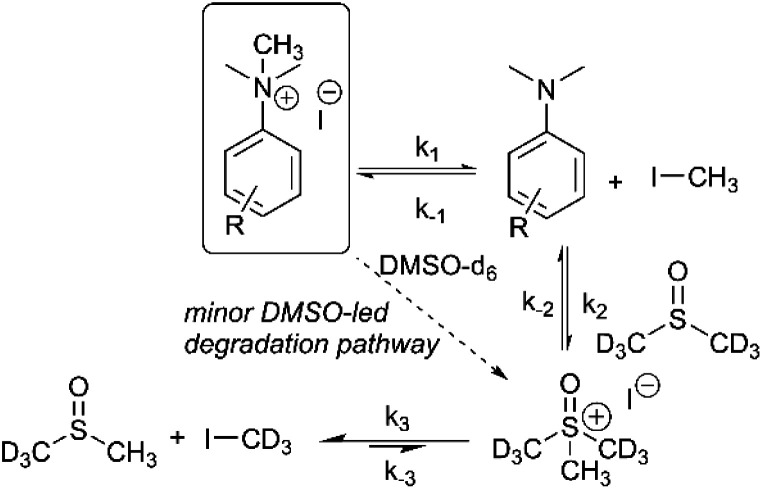
First proposed degradation pathway of *N*,*N*,*N*-trimethylanilinium iodide salts in DMSO-d_6_, with rate constants assigned to each step. Minor deuterium scrambling steps have been omitted for clarity.

Our combined mechanistic evidence to this stage led to the tentative proposal of the mechanistic pathways summarised in Fig. 13. Mass-calibrated diffusion-ordered NMR spectroscopy (DOSY) analysis of various anilinium salts in DMSO at room temperature did not support the existence of observable concentrations of higher order salt aggregates (see Experimental ESI Section 13†), though this does not directly rule out their transient existence. We proposed that the primary pathway, *via* a relatively rapid approach to equilibrium, of an approximately thermoneutral self-immolative degradation led by nucleophilic attack of iodide onto a *N*-methyl unit of the anilinium cation, leading directly to the parent dimethylaniline and methyl iodide. In the bulk solvent, we proposed that DMSO could act as a S-centred nucleophile^56,65^ and attack the methyl iodide in an S_N_2 fashion, producing trimethylsulfoxonium iodide (TMSO-d_6_,h_3_ iodide). The TMSO-d_6_,h_3_ cation could then undergo the reverse reaction with the aniline or instead undergo a self-immolative process to release d_3_-methyl iodide. The incorporation of CD_3_ in the anilinium cation, which could remain present in the degradative aniline product, is consistent with the difference in anilinium and aniline concentrations calculated using ^1^H NMR spectroscopy, assuming negligible aromatic H/D exchange. In further support of this mechanistic hypothesis, methyl iodide formed at an initial rate consistent with aniline formation (Fig. 12 green and purple), while the TMSO-d_6_,h_3_ intermediate and, in turn, DMSO-d_3_ product formation displayed an induction period. This suggested that iodide-led degradation of anilinium cation is the major (though not exclusive) precursor to methyl transfer to solvent. In short, methyl iodide forms directly, in the first degradation step, not later.

The proposed degradation pathway takes place *via* a series of two-electron closed-shell processes. In order to probe for the possibility of radical degradation pathways, the degradation of **3a** (4-CHO) was carried out separately under UV light and in darkness. Comparatively, the amount of **3a** degraded in each case was almost identical, suggesting that photo-degradation mechanisms are not dominant (see Experimental ESI Section 15†). The absence of radical-mediated mechanisms is also supported by additional control experiments in applied methylation chemistries (see Applications section below). It is plausible that the depletion of the anilinium salt could also occur through an S_N_Ar-type displacement of trimethylamine by the iodide counterion, however the corresponding aryl iodide was not observed (see Experimental ESI Section 14†).

**Fig. 14:**
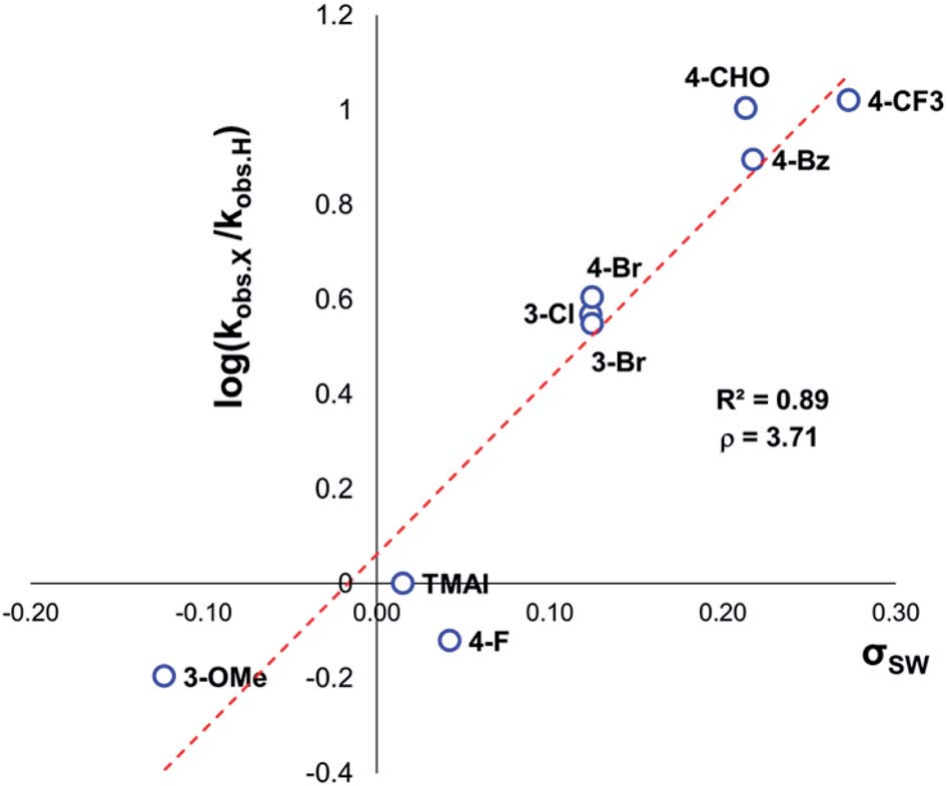
Swain–Lupton analysis using initial rate values (not explicitly derived rate-constants) for anilinium iodide degradation. Modified Hammett substituent constants were derived from Swain–Lupton *F* and *R* constants such that *σ*_SW_ = 0.52*F* + 0.48*R*.

The same ^1^H NMR kinetics method was used to follow the degradation time course of a wider range of trimethylanilinium salts (Experimental ESI Section 11†). With the exception of **2a** (2-py) and **14a** (2-Me), most salts appeared to reach equilibrium that did not involve full salt degradation, consistent with a process that is approximately thermoneutral. Exploratory Swain–Lupton analysis^66^ was applied to solution-phase initial rate data to delineate field and resonance contributions of aryl ring substituents to salt degradation. This analysis revealed near equal field (*F*) and resonance (*R*) contributions (52% *Fversus* 48% *R*; Fig. 14). The positive reaction constant (*ρ* = +3.71) was consistent with earlier observations that more electron-deficient anilinium salts degrade more quickly (*cf.*Fig. 5 and 7). Additional Swain–Lupton analysis using the simulated *k*_1_ values based on the tentative reaction model shown in Fig. 13 lead to similar conclusions (51% *R versus* 49% *F*; *ρ* = +0.89; see Experimental ESI Section 11†).

**Fig. 15:**
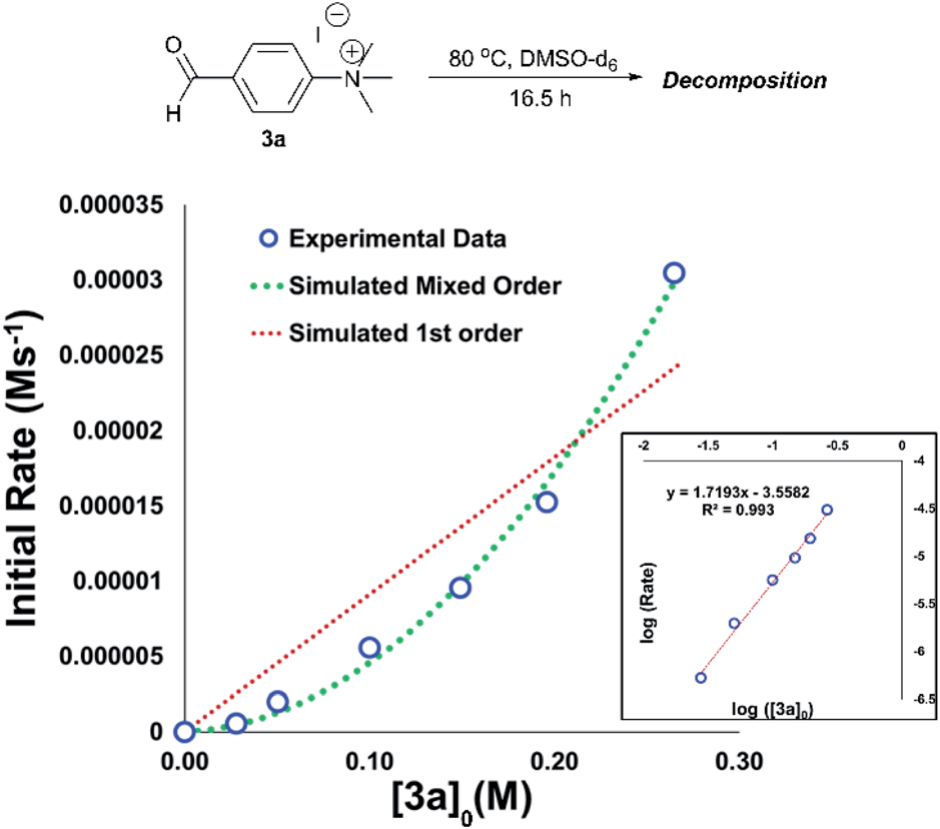
Main plot: Initial concentration of **3a***versus* calculated initial degradation rate showing apparent mixed order kinetics. Real data (blue open circles) show agreement with simulated 1st + 2nd mixed order kinetics (green dashed line) *versus* simulated first order kinetics (red dashed line). Inset: log–log plot showing apparent reaction order of approximately 1.7 in [**3a**].

An important consideration when using a new reagent is the concentration at which it is to be used. Our time course experiments up until this point had been carried out with an anilinium salt concentration of 0.1 M in DMSO-d_6_ for comparability. To understand the dependence of anilinium degradation on concentration, we carried out five additional degradation monitoring experiments at various anilinium iodide starting concentrations, [**3a**]_0_. Inspection of the data revealed a deviation from linearity, providing evidence of greater than first order reaction order kinetics in [**3a**]_0_ (Fig. 15). This is not consistent with a simple self-immolation mechanism involving a single ion pair as the sole degradation mechanism, as suggested in Fig. 13. Consistent with data reported in Fig. 11, analysis of degradation of the BArF salt of [**3a**] *via in situ* NMR kinetics revealed that the background rate of degradation exclusively *via* the DMSO pathway (in the absence of nucleophilic iodide) was negligible at 80 °C (see Experimental ESI Section 12†). While DOSY NMR revealed no appreciable concentration of aggregates beyond the simple anilinium iodide ion pair at room temperature under the range of concentrations studied, the presented data (most notably Fig. 15) remain consistent with the possibility of degradation of the anilinium iodide salt *via* a transient dimeric ion pair or alternative higher order reaction.^63^ Dimeric degradation pathways were later considered computationally (below, and Computational ESI, Section 3†).

**Fig. 16:**
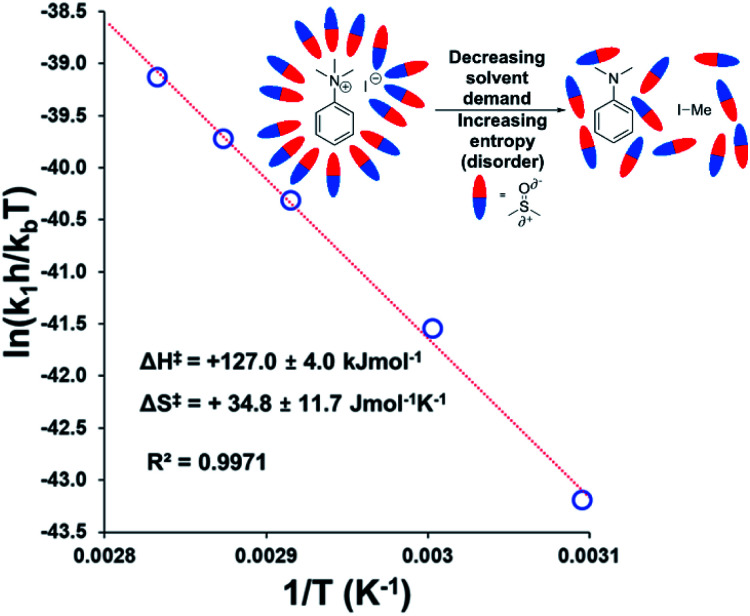
Exemplar Eyring plot for the degradation of **3a** between 50–80 °C, using simulated first order rate constants, showing counterintuitive positive entropy of activation. Inset: Explanatory cartoon model citing reduced solvent demand as the root cause of the observed entropy value. Similar conclusions from the Eyring plot with simulated second order rate constants is available in the Experimental ESI (Section 18).†

To estimate thermodynamic activation parameters, we conducted NMR monitoring studies between 50–80 °C. Rate constants were estimated for a simplified first order iodide-led self-immolative degradation pathway at each temperature and an Eyring plot was produced using this data (Fig. 16). The Eyring analysis revealed the activation enthalpy and entropy of the reaction as Δ*H*^‡^ = +127.0 ± 4.0 kJ mol^−1^ and Δ*S*^‡^ = +34.8 ± 11.7 J mol^−1^ K^−1^. Δ*H*^‡^ and Δ*S*^‡^ values for reactions involving changes in charge should be interpreted with caution as the values will include solvation dynamics and selection of appropriate rate constant from the mechanistic model(s). To cover this potential calculation error, we estimated Eyring parameters using both first and second order models, and in all circumstances, a positive entropy of activation was found. The positive activation entropy obtained in this case is hypothesised to be due to a reduced solvation demand as the charged reactants proceed to neutral products (see Computational section).^67^ This model is also consistent with the reaction proceeding through ion pairs wherein the translational entropy penalty of bringing electrophile and nucleophile together has largely been paid. The overall Δ*G*^‡^ = +114.7 kJ mol^−1^ (+27.4 kcal mol^−1^) derived from these calculations is in good agreement with the related DFT-calculated barrier of +116.7 kJ mol^−1^ (27.9 kcal mol^−1^; see below).

To gain further insight into the degradation mechanism, a kinetic isotope effect (KIE) study was conducted. A d_9_-analogue (Ar-N(CD_3_)_3_) of the 3-bromo anilinium iodide salt (**11a**) was synthesised and subjected to an NMR degradation experiment. The rates of degradation were determined by independent experiments. While not conclusive on their own, all data were consistent with there being no primary KIE (approx. 1.18), thus ruling out mechanisms involving direct deprotonation (*i.e.* ylide formation) on the anilinium cation N–Me groups (see Experimental ESI Section 19†). These data are also qualitatively consistent with the ∼90-times molar excess of [CD_3_] *versus* [CH_3_] groups present in solution, and with DFT-calculated KIE range of 1.29–1.47 covering 4 major mechanistic hypotheses (see below).

### Applications of mechanistic analysis in methylation chemistries

**Fig. 17:**
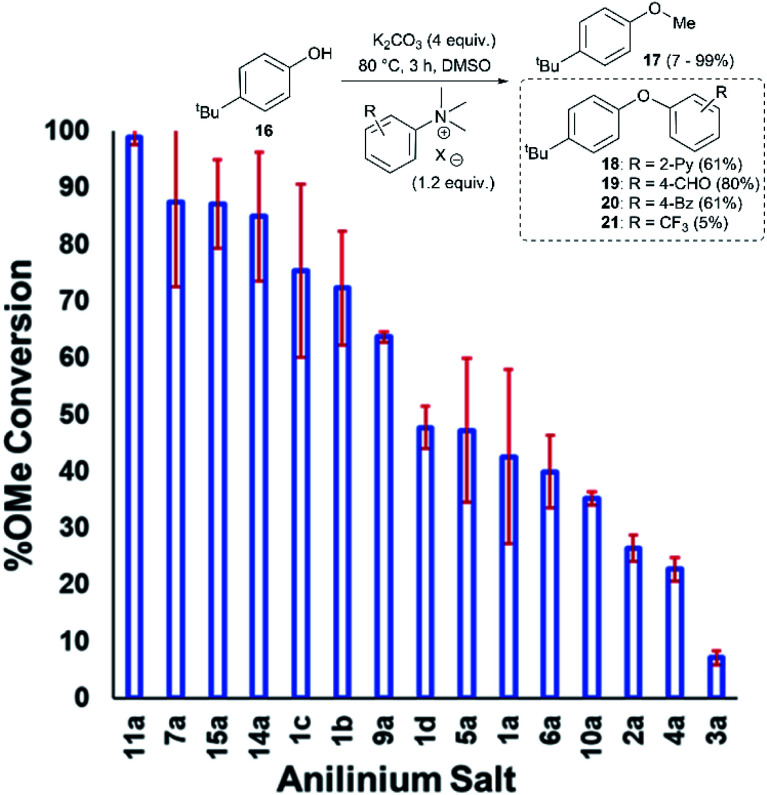
Screen of *O*-methylation capability enabled by anilinium salts.

Applying our new mechanistic data, we examined the broader use of anilinium salts as methylating reagents. Degradation analysis revealed the range of thermal and additive stabilities (knowledge applicable to both cross coupling and process safety strategies). We hypothesised that those anilinium salts presenting as thermally unstable would, in turn, release methyl iodide *in situ* most readily, and also be more susceptible to direct nucleophilic attack, thus behaving as a viable methyl iodide replacement. For a comparative application in methylation, phenol *O*-alkylation was chosen as it remains one of the most used reactions in the pharmaceutical industry.^68,69^ 4-^*t*^Bu-Phenol (**16**) was selected as a suitable substrate for initial exploratory study. The conversion of **16** to 4-^*t*^Bu-anisole (**17**) was calculated from the reaction mixture after 3 h *via*^1^H NMR spectroscopy, and the average conversion of each salt from triplicate experiments is shown in Fig. 17.

From these experiments, the hypothesised trend of thermally unstable anilinium salts leading to more efficient methylation did not hold. Indeed, three of the most thermally unstable anilinium iodides **2a–4a** and **15a** – all carrying strong electron-withdrawing aryl substituents – led to measurable quantities of S_N_Ar products (**18–21**) and poor yields of desired methylation products. Indeed, analysis of thermal degradation *versus O*-methylation efficiency within the anilinium salt library revealed that only those unstable salts bearing no resonance withdrawing aryl substituents served as efficient methylating reagents.

**Fig. 18:**
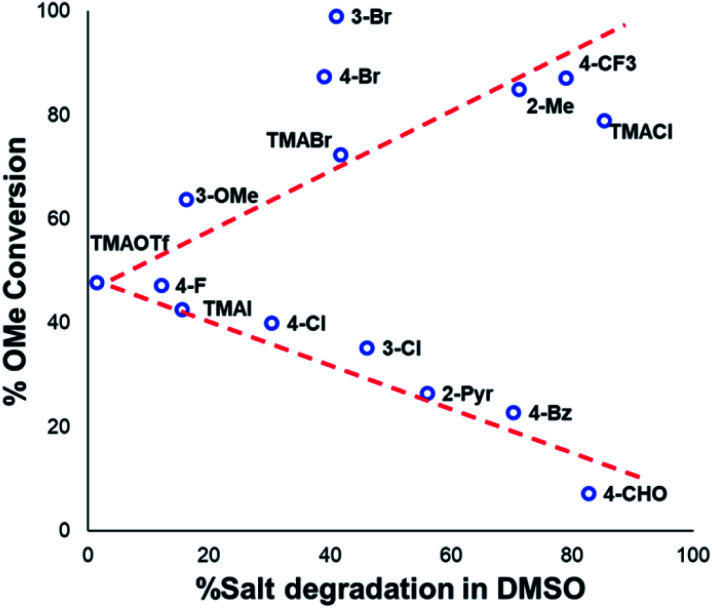
Partition of anilinium salts accordingly to their thermal degradation and *O*-methylation behaviours. Non-iodide salts are indicated where appropriate. The dotted lines are included solely as a guide for the eye.

The anilinium iodide salt most reactive for methylation was shown to be **11a** (3-Br), giving near-quantitative conversion to **16** with minimal variance (Fig. 18). We were surprised to see that salt **10a** (3-Cl), which has a similar degradative profile to **11a** (Fig. 5, **7** and **14**), was considerably less reactive to methyl transfer under the applied conditions. More expectedly, **7a** (4-Br) gave high conversions in comparison to the **5a** (4-F) and **6a** (4-Cl) analogues, the latter two presenting as more stable to thermal degradation. **14a** (2-Me), the only salt identified as prone to degradation due to steric bulk, gave an average of 85 ± 11% conversion to the methylated product under the conditions tested. Salt **14a** presents a viable alternative to **11a** where methylation (or cross-coupling) reactions might be compromised by the presence of the 3-Br substituent in **11a**. Surprisingly, **2a** (2-Py), **3a** (4-CHO), and **4a** (4-Bz) anilinium iodide salts, each gave a relatively low conversion to **17** despite being the most thermally unstable salts in solution-phase. On closer inspection, it was found that S_N_Ar products **18–20**, were formed from use of the most resonance withdrawing anilinium iodide salts (Fig. 17). The S_N_Ar reactivity presumably arises from 4-^*t*^Bu-phenoxide displacing trimethylamine from the respective anilinium salt.^6,65^ Our own calculations remain consistent with the concerted S_N_Ar mechanisms previously reported separately by DiMagno^12^ and Jacobsen.^65^ See the Computational analysis section (Fig. 23).

The trend in methylation ability for the halide salt series **1a–c** correlated with extent of thermal degradation in solution, with the chloride salt giving higher conversions to methylated product than the analogous bromide and iodide salts. The triflate salt (**1e**) was comparative in methylating ability to the iodide salt (**1a**). Our mechanistic degradation studies showed that methyl iodide was produced upon heating the anilinium iodide salts in DMSO, raising the question of whether methylation of **16** occurs from reaction of the phenoxide nucleophile directly with the anilinium cation, from a methyl halide generated *in situ*, or both. The shared trend in anilinium degradation and phenol methylation reactivity (**1c** > **1b** > **1a**) suggests that decomposition to the methyl halide is important for reactivity. However, the comparable methylating ability of **1a** (X = I) and **1e** (X = OTf), despite the large difference in stability (see Fig. 9 and 13), indicates that attack directly onto the anilinium salt also occurs, assuming negligible degradation of the triflate salt to produce methyl triflate *in situ* (Fig. 11). We propose that a combination of direct reaction with the anilinium salts and indirect reaction (*via* methyl iodide generation) in the phenol methylation both occur under the reaction conditions employed. This proposal is further elaborated in discussion below.

To compare the effectiveness of methylating reagent **11a** to common electrophilic methylating reagents, the methylation of phenol **16** was carried out using methyl iodide, methyl triflate, methyl tosylate, dimethylsulfate, and dimethylcarbonate under our chosen reaction conditions (Table 1).

**Table 1:** Comparative methylation with anilinium salt **11a***versus* common electrophilic methylating reagents

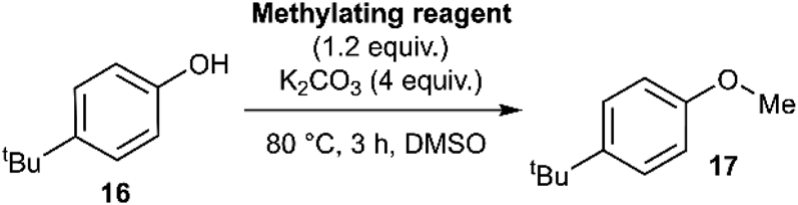		
Entry	Methylating reagent	Yield (%)
1	**11a**	98
2	MeI	79
3	MeOTf	2
4	MeOTs	62
5	Me_2_SO_4_	29
6	Dimethyl carbonate	0
7	Dimethyl carbonate (+10 mol% DBU)	0

**Fig. 19:**
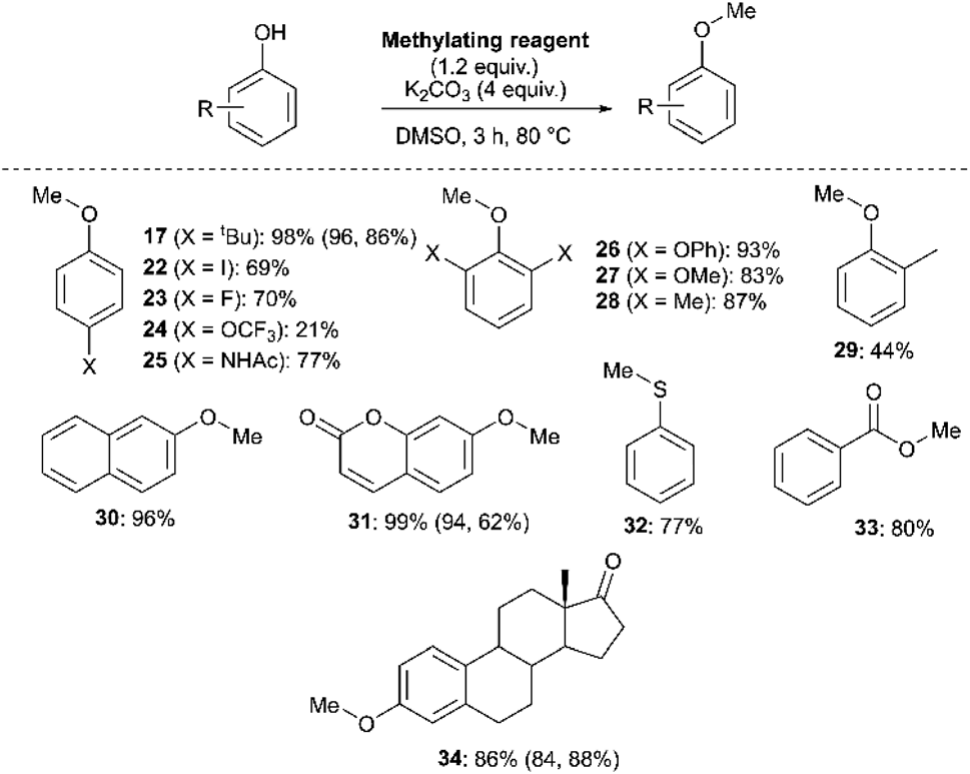
Methylation of phenols, thiophenol and benzoic acid using **11a**. Yields in parentheses obtained with **14a** (2-Me) as the methylating reagent (left number) or for 8 h in NMP as solvent (right number), under otherwise identical conditions.

We next investigated the ability of **11a** (3-Br) to methylate a range of phenols and related nucleophiles under our applied reaction conditions (Fig. 19, see Experimental ESI Section 20† for optimisation). A range of substituents and structural complexities around the phenol ring were tolerated in these methylation reactions, and most with good to excellent isolated yields (**17**, **22–31**, **34**). Additionally, thiophenol and benzoic acid could be methylated under the applied conditions to give thioether **32** and ester **33** with good 77% and 80% yields, respectively. Notably, having used the earlier mechanistic study to guide choice of reagent (Fig. 17), no S_N_Ar product was observed in any of our reactions using **11a** as a methylating reagent. **14a** (2-Me) was explored as an alternative methylating reagent for cases where a 3-Br substituent could cause undesired reactivity, and was shown to be effective in the formation of **17** (86%), **31** (62%) and **34** (84%). Using the same subset of substrates, NMP was shown to be a viable alternative solvent, enabling the synthesis of **17**, **31**, and **34** in 96, 94, and 84% yield, respectively.

**Fig. 20:**
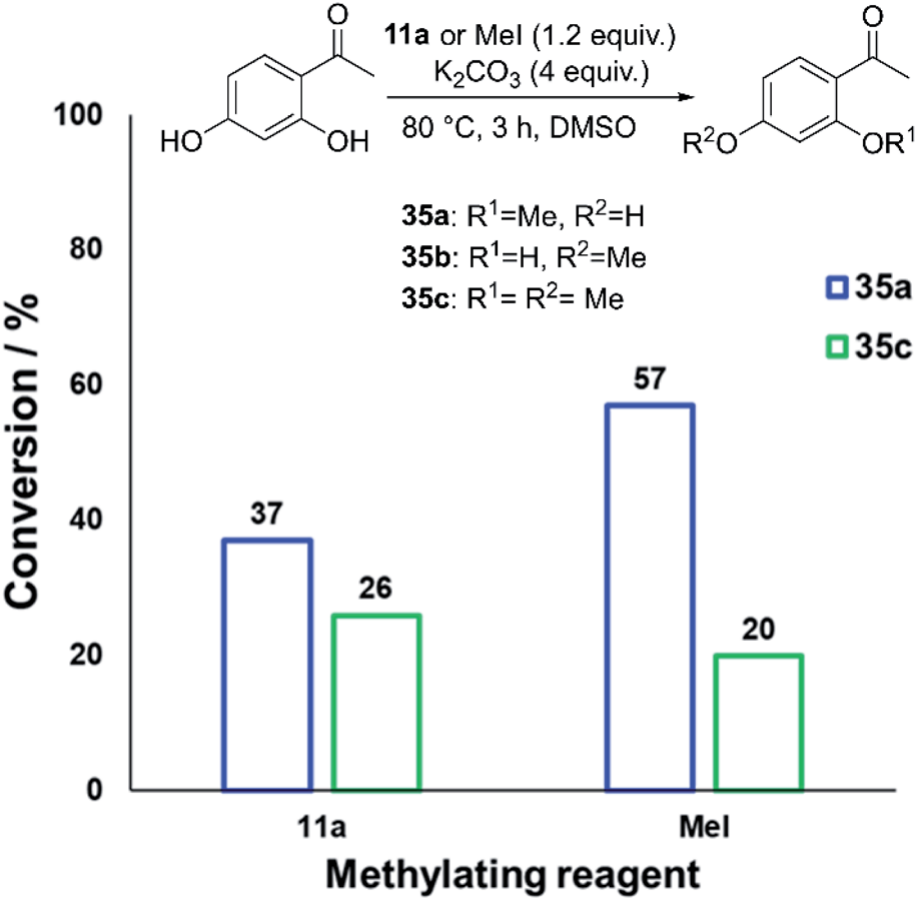
Conversion of 2′,4′-dihydroxyacetophenone to **35a–c** using **11a** or methyl iodide, demonstrating the possibility of exploiting differing methylation product selectivities. In both cases, no **35b** was formed.

We also investigated whether or not **11a** could provide any uniquely applicable regiochemical control when multiple nucleophilic phenol sites are present on a substrate. To probe this, 2′,4′-dihydroxyacetophenone was subjected to methylation with **11a** and methyl iodide under our standard conditions, showing exploitable differences in reactivity and regioselectivity (Fig. 20). A deeper analysis of this synthetic application is the subject of future investigation.

### Experimental mechanistic analysis of active methylation species in solution using anilinium salts

**Fig. 21:**
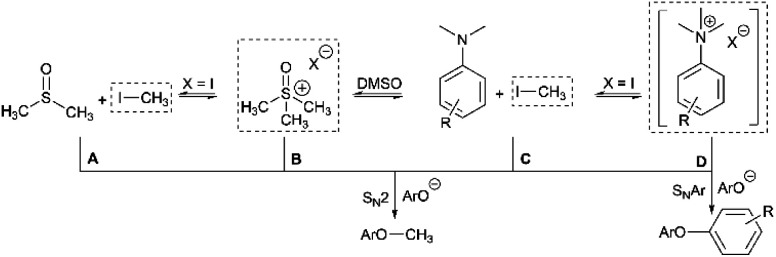
Sources of electrophilic methylation reagent when employing an anilinium iodide in DMSO. The dotted boxes highlight the source of “Me^+^” at each side of the connected equilibria. Competing S_N_Ar reactivity for the anilinium salt is also shown.

Having demonstrated the potential of *N*,*N*,*N*-trimethylanilinium (primarily iodide) salts to act as competent methylating reagents, we next aimed to understand the plausible mechanism(s) by which these *O*-methylation reactions occur. Our understanding of anilinium thermal degradation in DMSO-d_6_ suggested multiple possible methylation pathways: Path A: from methyl iodide generated from TMSO–I degradation, Path B: from TMSO–I that forms *in situ* through methylation of the non-innocent DMSO solvent by methyl iodide, Path C: *via* methyl iodide generated from the anilinium iodide degradation, and Path D: direct attack of the nucleophile onto an *N*-methyl group of the anilinium cation (Fig. 21).

To address the question of whether or not substrate methylation comes solely from the anilinium degradation-led generation of methyl iodide (Path C), we carried out the methylation of **16** under our chosen (loosely-optimised) reaction conditions using 3-bromo-*N*,*N*,*N*-trimethylanilinium PF_6_ (**11d**) and BArF (**11f**) salts (Table 2). In these reactions where methyl iodide could not be generated, **17** was formed in 45% (entry 2) and 50% (entry 3) yield, compared to 98% yield when using iodide salt **11a** (entry 1). This showed that the generation of methyl iodide likely accompanies efficient methylation of phenoxide substrates, but is not a necessity for the reaction to occur under the chosen conditions. Given the knowledge that anilinium PF_6_ and BArF salts can partially decompose in DMSO-d_6_ to give the TMSO cation (see Fig. 11), it is possible that methylation could occur in these reactions from the anilinium directly (Path D) or the TMSO cation itself (Path B).

**Table 2:** Mechanistic experiments investigating the conversion of **16** to **17** with various methylating reagents in DMSO and NMP. Conditions: **16** (1 mmol, 1 equiv.), methylating reagent (1.2 equiv.), K_2_CO_3_ (4 equiv.), additive (various equiv.), solvent (2 mL), 80 °C, 3 h

Entry	Methylating reagent	Anion	Solvent	Additive (equiv.)	*O*-Methylation yield (%)
1	**11a**	I	DMSO	—	98
2	**11d**	PF_6_	DMSO	—	45
3	**11f**	BArF	DMSO	—	50
4	TMSO–I	I	DMSO	—	76
5	TMSO–PF_6_	PF_6_	DMSO	—	57
6	TMSO–BArF	BArF	DMSO	—	53
7	**11a**	I	NMP	—	88
8	**11d**	PF_6_	NMP	—	11
9	**11a**	I	DMSO	TEMPO (3)	65
10	**11a**	I	DMSO	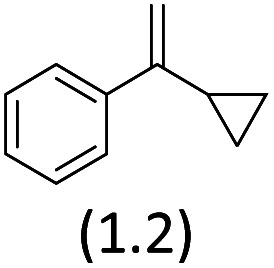	86 (no consumption of additive)

To investigate this further, we ran the same methylation reactions with TMSO salts in the place of anilinium salts. TMSOI formed **17** in 76% yield (entry 4). We were able to demonstrate through NMR analysis that TMSOI decomposes thermally to give DMSO and methyl iodide at 80 °C, the reverse reaction was also shown to occur (Experimental ESI Section 22†). This finding raised the question as to whether TMSO^+^ acts directly as a source of methyl group (Path B), or if the methylation is simply occurring from methyl iodide produced *via* its thermal decomposition (Path A). TMSO–PF_6_ and TMSO–BArF were synthesised and subsequently used in methylation reactions to afford the anisole **17** in 57% (entry 5) and 53% yield (entry 6), respectively. While this suggests that the TMSO^+^ cation is capable of methylating phenols, it does not rule out the possibility that nucleophiles can directly attack the anilinium cation to form the methylated product (Path D). To investigate the ability of the anilinium cation to directly methylate the substrate, the ability to generate TMSO^+^ in the reaction mixture would also have to be removed. Accordingly, we ran the methylation reactions in *N*-methyl-2-pyrrolidone (NMP). Anilinium iodide **11a** was used to methylate **16**, giving 88% of the methylated product (entry 7).

We then repeated the reaction using anilinium PF_6_ salt **11d** as the methylating reagent, which gave only 11% yield of **17**, strongly suggesting that the degradation of the anilinium iodide to release methyl iodide *in situ* is a key contributor to the reaction (Path C); computational studies reveal that the barriers for degradation and direct reaction are close such that reaction flux through each pathway likely depends on the details of concentration, temperature and the identity of the anilinium salt. Furthermore, additive experiments (entries 9 and 10) probing the presence of radical-centred degradation and methylation mechanisms were carried out. These data remained consistent with experimental and theoretical evidence that anilinium iodide degradation was primarily *via* closed shell pathways, and that the principle self-immolative degradation pathway was faster than degradation *via* DMSO participation (see Fig. 9, **11** and **12**).

### Computational analysis of anilinium salt degradation and methylation mechanisms

Further mechanistic insight for both anilinium salt degradation and methylation was sought from computational studies. The computations were all performed at the M06-2X/6-31+G** level of theory and with IEF-PCM for DMSO as the solvation model, all performed in Gaussian09.^70^ M06-2X was designed to be a preferred method for the investigation of reaction barriers, and so is appropriate for this task. Vibrational and standard state corrections were implemented using Goodvibes (with concentration values 1 M and 14.1 M respectively for solutes and DMSO; a frequency cut-off of 100 cm^−1^ was used).^71^ In exploring competition between first and second order reactions, the role of specific interactions between solvent and solute was explored by placing DMSO molecules such that their oxygen was in positions expected to be favoured for such interactions, namely: between the methyl groups of anilinium or TMSO, between the *ortho*-hydrogens and the methyl groups of an anilinium, and, in the case of **3a (4-CHO)**, adjacent to the formyl hydrogen with methyl groups of the DMSO solvent molecules placed so as to interact with the carbonyl group. The lowest free energy guides selection of the preferred solvation geometry (and explicit solvent molecules are excluded when they involve an increase in free energy). The solvation free energy of DMSO itself (in either implicit or explicit solvation regimes) is assumed to be approximately 0 kJ mol^−1^ in line with the thermodynamic analysis of Lai *et al.* and so gas phase values are employed.^72^ The details of all calculations are provided in the Computational ESI.† Generally, the implicit solvation calculations are reported unless noted.

A key first step was to establish the likely speciation of the anilinium salts, as either separated ions and/or as ion pairs. The latter was supported by our DOSY NMR measurements (Experimental ESI Section 13†). Two geometries were considered for the ion-pair, one with the halide placed as an extension of the *ipso*-C_aromatic_–N bond, and the other with the halide between the N-methyl groups and the *ortho* C–H position. The latter was found to be energetically preferred in all cases investigated (see Computational ESI Section 2†). Calculations at 353 K (80 °C) predicted all of the iodide salts to have a small (3–16 kJ mol^−1^, or 0.8–3.8 kcal mol^−1^) preference to be ion-paired (see Computational ESI Section 5†). The bromides are computed to have a mix of preferences, with 4-CHO and 3-Br showing ∼2 kJ mol^−1^ (or ∼0.5 kcal mol^−1^) preferences to be ion pairs, while 2-Me and 2-pyridyl show preferences to be separate ions. Chloride salts were computed to all have an energetic preference of 0.8–11.7 kJ mol^−1^ (or 0.2 to 2.8 kcal mol^−1^) to be separated ions apart from for 3-Cl and 3-Br.

**Fig. 22:**
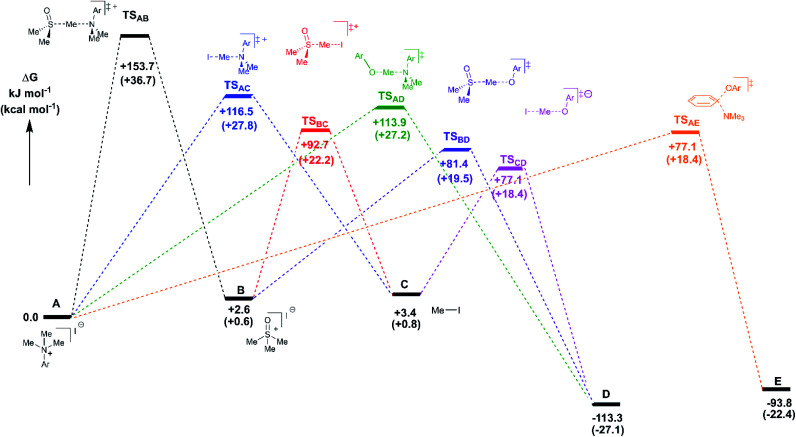
DFT-derived free energies for transition states and intermediates for the reaction of **3a** with the reference state at 0 kJ mol^−1^ being a tight anilinium iodide ion pair (in this case). Experimentally-consistent mechanisms for anilinium iodide degradation and anilinium-mediated *O*-methylation are shown and described further in the text. Energies shown are free energies in kJ mol^−1^, with kcal mol^−1^ values shown in parenthesis. Method: M06-2X/6-31+G**/IEFPCM (DMSO). *T* = 353.15 K (80 °C).

Reaction profiles were computed for each of the anilinium salts in which both degradative reactions (MeI formation and reaction with solvent) and productive reactions (S_N_2 and S_N_Ar with phenolate) are compared. An example is given for **3a** in Fig. 22 where:

State A = the reactant anilinium salt (as an ion-pair)

State B = methyl transfer to solvent DMSO

State C = self-immolative methyl transfer to halide (*i.e.* MeI is formed)

State D = S_N_2 methylation of phenolate, and

State E = S_N_Ar reaction.

Some features are consistent for each of the iodide salts. First, the reaction of the anilinium iodide with DMSO is kinetically and significantly disfavoured (Fig. 22, A → B). The formation of MeI (Fig. 22, A → C) is close to thermoneutral and has a barrier that is readily accessible at the elevated temperatures employed in the experimental studies. Electron donating groups disfavour formation of MeI. The reverse reaction in which anilinium reforms is also accessible. The barrier for methyl iodide to react with DMSO is 89.3 kJ mol^−1^ (or 21.3 kcal mol^−1^). These calculations are consistent with the observed delay in sulfoxonium formation coming only after methyl iodide formation, the intermediate formation of both methyl iodide and sulfoxonium, the induction period in the observed formation of DMSO-d_3_, and the observed positions of H/D scrambling (Fig. 12).

**Fig. 23:**
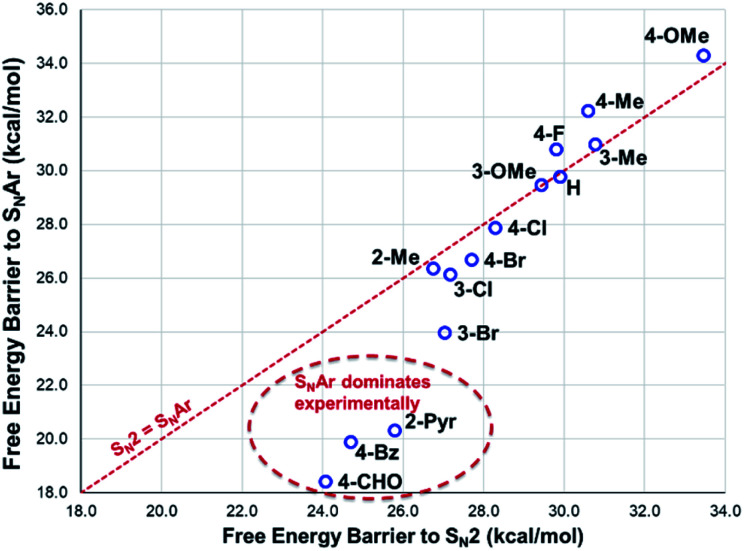
DFT-computed comparison of S_N_Ar and S_N_2 barriers for reaction of phenolate with anilinium cation. Method: M06-2X/6-31+G**/IEFPCM (DMSO). *T* = 353.15 K (80 °C).

The reactions with phenolate, S_N_2 (Fig. 22, C → D) and S_N_Ar (Fig. 22, A → E) have barriers that are low and largely irreversible. For salts **2a** (2-Pyr), **3a** (4-CHO), and 4-Bz (**4a**), the barrier for S_N_Ar is computed to be the most lowered *versus* S_N_2 (Fig. 23), qualitatively corresponding to the observation of S_N_Ar product dominating the product mixture (Fig. 18). More electron-donating substituents are computed to have a preference for S_N_2 but this is accompanied by an overall higher barrier for reaction; thus, iodide salts predicted to be selective for methylation over arylation are also predicted to react more slowly. Although the 4-CHO salt (**3a**) has barriers for direct reaction of phenolate with salt that are significantly lower than those for formation of MeI (77.7 kJ mol^−1^ for S_N_Ar *vs.* 116.5 kJ mol^−1^ for S_N_2), this is not the case for all anilinium salts universally. For instance, the unsubstituted iodide salt, **1a** (Computational ESI Section 3†), is computed to have a barrier to formation of MeI of 125.2 kJ mol^−1^ (29.9 kcal mol^−1^), a barrier to S_N_Ar of 124.6 kJ mol^−1^ (29.8 kcal mol^−1^), and a barrier to S_N_2 with phenoxide of 120.4 kJ mol^−1^ (28.8 kcal mol^−1^), and thus all three processes might be expected to be operating experimentally. While the calculations are not able to explicitly corroborate **11a** (3-Br) as the experimentally optimum methylating reagent, they are suggestive of this salt as having a good balance between the likely degree of degradation and the selectivity between S_N_2 and S_N_Ar reactions, thus providing an effective and safer ‘slow bleed’ alternative to methyl iodide.

**Fig. 24:**
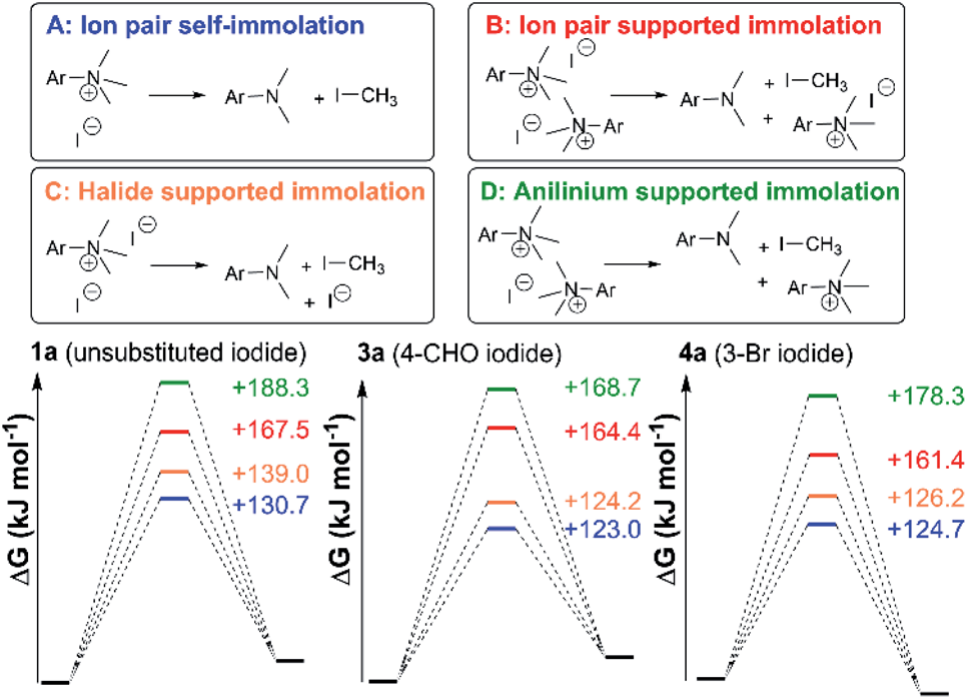
Four anilinium salt degradation mechanisms for which DFT-computed solutions were found.

Fig. 24 shows three alternative modes of anilinium iodide self-immolation in ion-pairs, beyond the simple first order self-reaction, that were all considered computationally. In this case, explicit DMSO molecules were included around the anilinium (or formyl groups in **3a**) for any instances where such explicit solvation was computed to have a negative free energy of association. Of all 4 mechanisms of degradation (A–D), A and C were consistently calculated to be the most accessible across calculations involving salts **1a**, **3a**, and **11a**; thus, at high concentrations and at 80 °C, second order contributions to anilinium iodide degradation are computed to be accessible. This is consistent with the observed deviation from linear concentration dependence reported in Fig. 15. At first sight, this suggestion appears inconsistent with the lack of sensitivity to addition of excess iodide revealed in Fig. 9. However, it should be kept in mind that the temperature dependence of the second order reactions (that involve the entropic penalty of bringing together two reactants) will be markedly different from that for the first order reaction. The calculations support a remarkably fine balance between a number of alternative modes of reactivity that can be selected between with careful choice of concentrations, temperatures, and reagents.

### Limitations of mechanistic analysis

**Fig. 25:**
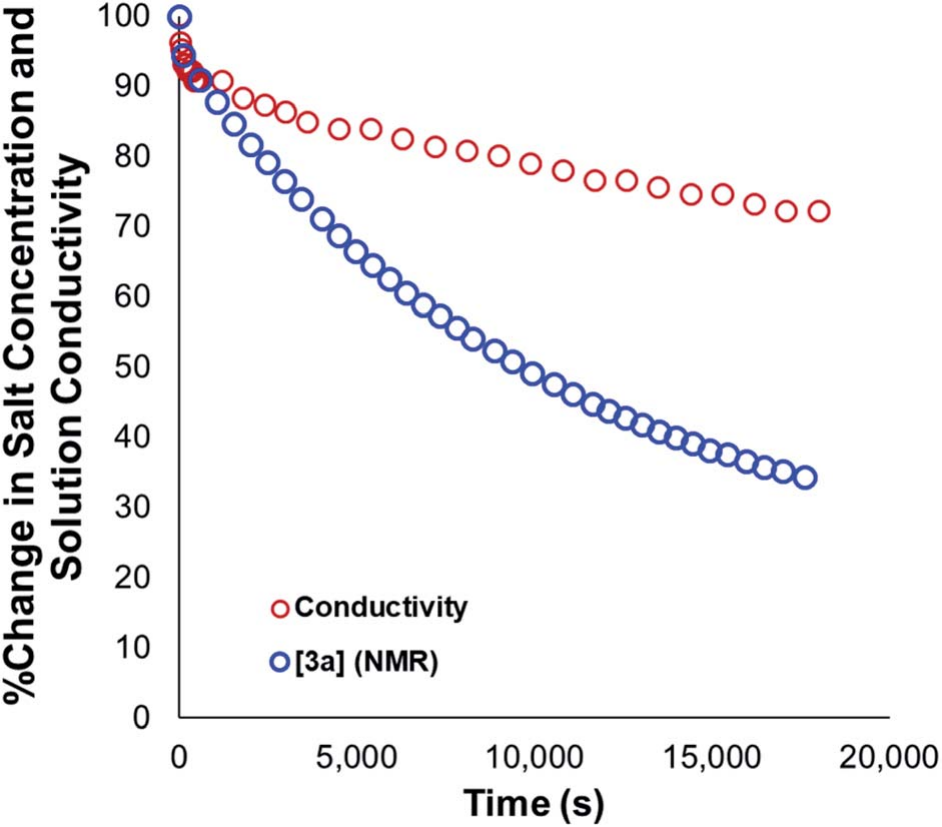
Relative change in measured solution conductivity relative to changes in **3a** salt concentration during degradation. Conditions: **3a** (0.1 M) in DMSO-d_6_ (0.6 mL), 1,2,4,5-tetramethylbenzene as internal standard (0.06 M; not plotted) at 80 °C.

Our mechanistic analysis – both experimental and computational – is consistent with at least two operative mechanisms of anilinium iodide degradation. The first and most dominant is simple first order self-immolation; one ion-pair degrades to one molecule of dimethylaniline and one molecule of methyl iodide. The second mechanism, accessible at higher concentrations of anilinium iodide, is proposed to be (at least in part) second order with respect to the anilinium iodide ion pair. Further microkinetic analysis remains consistent with both first and second order processes being operative (see Experimental ESI Section 17†). The main unavoidable assumption in all mechanistic analysis, is that these reactions proceed in an unchanging medium that presents unchanging physical properties as the main reactions proceed. In reality, an unchanging medium is highly unlikely, especially for such reactions proceeding from charged reactants to neutral products. Conductivity measurements (Fig. 25, and Experimental ESI Section 25†) and scans of solvent model dielectric constant *versus* Δ*G* (Computational ESI, Section 5†) show that the physical properties of the reaction medium change with time. The temperature variation of the nature of both the medium and the solvation thermodynamics are also unlikely to be universally consistent for the different structural variations of anilinium iodide. As a result of the identified subtleties of these very commonly applied DFT methods, this investigation also provides a powerful data set on ionic reactivity that can actively inform new computational approaches to studying the impact of solvent and counterions on reactivity.^73^

## Conclusions

**Fig. 26:**
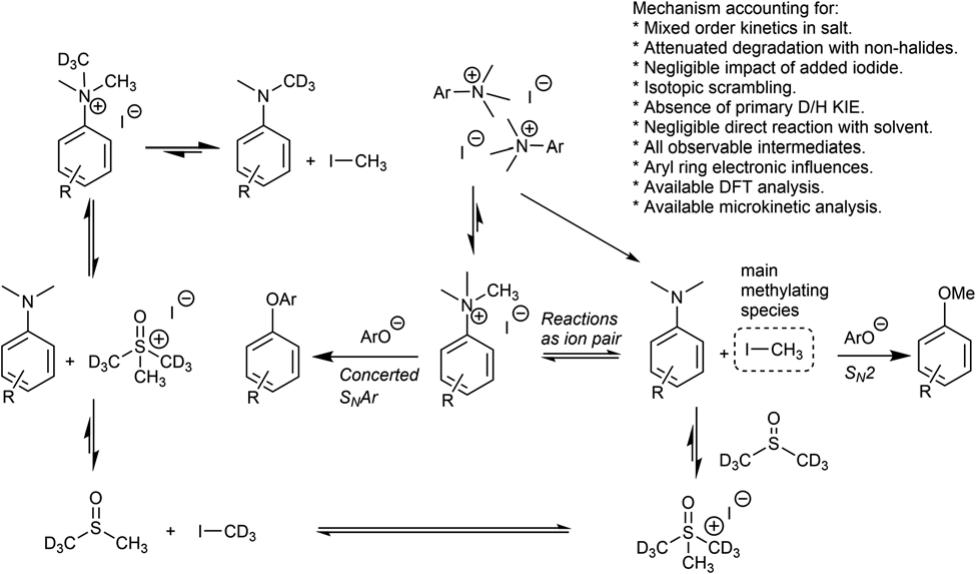
Revised mechanistic model for anilinium iodide degradation, consistent with all available experimental and computational mechanistic evidence.

We have presented solid and solution phase thermal degradation analyses, kinetic studies, isotopic labelling, and computational modelling showing that *N*,*N*,*N*-trimethylanilinium salts are likely to degrade in a self-destructive process. Taken together, the mechanistic experiments forced a partial revision of our initial mechanistic hypothesis from Fig. 13, as displayed in Fig. 26. Increasing the anilinium counter-anion nucleophilicity, solution concentration, electron-withdrawing aryl substituent power, soluble non-iodide halides, and solution temperature were all found to increase the rate of salt degradation. The minimal impact of solvent-led degradation pathways was supported by computational modelling of the two degradation pathways. A range of *N*,*N*,*N*-trimethylanilinium salts was tested as *O*-methylation reagents, with aryl-substitution and counterion species shown to affect methylating ability. 3-Br-*N*,*N*,*N*-Trimethylanilinium iodide (**11a**) was shown to be an effective methylating reagent for a variety of phenols in DMSO and NMP. Mechanistic studies suggest that *in situ* generated methyl iodide is likely to be the main methylating reagent in these reactions. From a practical standpoint, should these anilinium salts be made accessible without first using methyl iodide, we thus envisage that carefully selected anilinium iodide salt could function as a safer, crystalline, and storable ‘slow bleed’ alternative to using carcinogenic and low-boiling methyl iodide directly.^42^

The same studies have highlighted the most stable anilinium salt configurations, and those salts for which concerted S_N_Ar at the ipso carbon of the parent anilinium salts presents dominant reactivity, both key considerations for chemists aiming to exploit these same salts for cross-coupling as opposed to methylation reactivity.

Beyond advancing popular *O*-alkylation strategies,^69^ it is clear that varied applications of trimethylammonium salts is, at the time of writing, on the rise.^74^ As such, the implications of the above results on a broader range of chemistries involving trimethylanilinium salts is also worth delineating. In our estimation, the fundamental understanding of the structural parameters affecting anilinium salt degradation hold implications for: (i) the design of anilinium-derived ionic liquids used as solvents, electrolytes, and in electropolymerisation strategies,^75,76^ (ii) the design and understanding of anilinium-derived compounds used in antibacterial technologies,^77,78^ (iii) understanding the reaction conditions and range of anilinium substitution patterns amenable to metal-catalysed chemistries (including but not limited to arylation, alkynylation, silylation, and *ortho*-methylation),^7,8,23,29,31,79^ (iv) understanding substrate design for ^18^F radiolabelling mediated by S_N_Ar chemistries on anilinium cores,^12,80^ and (v) a fuller understanding of the range of anilinium structures applicable to ion-pair directed regiocontrol of C–H activation methods.^2^

## Author contributions

JBW – data curation; formal analysis; investigation; methodology; validation; visualization; writing – original draft. MA – data curation; formal analysis; investigation; methodology; validation; visualization; writing – original draft. CY – data curation; formal analysis; investigation; methodology; validation; visualization. DM – data curation; formal analysis; investigation; methodology; validation; visualization. VJ – data curation; investigation; visualization. AGL – supervision; data curation; formal analysis; investigation; methodology; validation; writing – original draft; writing – review & editing. SEB – supervision; writing – review & editing. MR – conceptualization; formal analysis; project administration; supervision; validation; writing – original draft; writing – review & editing.

## Conflicts of interest

There are no conflicts to declare.

## Supplementary Material

SC-012-D1SC00757B-s001

SC-012-D1SC00757B-s002
