# Supplementary material for: Trialkylammonium salt degradation: implications for methylation and cross-coupling
Source: Chem Sci. 2021 Apr 13;12(20):6949–63. doi: 10.1039/d1sc00757b (PMC8153232; doi:10.1039/d1sc00757b)
Supplement: SC-012-D1SC00757B-s001 [file SC-012-D1SC00757B-s001.pdf]

Electronic Supplementary Material (ESI) for Chemical Science.
This journal is © The Royal Society of Chemistry 2021

Computational Supporting Information

Trialkylammonium Salt Degradation: Implications for Methylation and Cross-coupling

Jack B. Washington,[a†] Michele Assante,[b†] Chunhui Yan,[a] David McKinney,[a] Vanessa Juba,[a] Andrew G. Leach,[c] Sharon E. Baillie,[d] and Marc Reid*[a]

a. WestCHEM Department of Pure and Applied Chemistry, University of Strathclyde, Thomas Graham Building, 295 Cathedral Street, Glasgow, UK.

b. School of Pharmacy and Biomolecular Sciences, Liverpool John Moores University, Byrom Street, Liverpool, UK.

c. Division of Pharmacy and Optometry, University of Manchester, Stopford Building, Oxford Road, Manchester M13 9PT

d. GlaxoSmithKline, Medicines Research Centre, Gunnels Wood Road, Stevenage, Hertfordshire, UK.

Contents

1. Methods 1
2. Reference State Analysis 2
3. Degradation Mechanistic Analysis 10
4. Higher Order Aggregate Analysis 13
5. Single Point Analysis 14
6. Explicit solvation 16
7. Geometries 17

1. Methods

All calculations were performed with Gaussian09 at the M062X/6-31+G(d,p) level of theory and with IEF-PCM for DMSO as implicit solvation model. LANL2DZ was developed with ECP (effective core potentials) to reduce the burden of calculations on ions with a high number of electrons and to account for some of the effects of relativity in the higher atomic weight elements.[1] It was used for Iodine in combination with 6-31+G(d,p) for the other elements when required. Also, all structures were calculated with the “gen” keyword in G09 to ensure consistent application of the basis sets. M06-2X was specifically developed to account for reaction barriers,[2] moreover it has been found to perform well with charged species.[3] Vibrational and standard state corrections were implemented using the Python package Goodvibes 2.0.[4] Concentration values of 1M and 14.1 M were used for solutes and DMSO respectively and a frequency a cut-off of 100 cm^{-1} was set. Temperature was set at 298.15 K or 353.15 K. Modifications of the dielectric constant were implemented through the ‘scrf=read’ keyword in Gaussian 09 input files. Calculations on cation-anion distances that included a BSSE correction did so through the ‘Counterpoise’ keyword in g09. Kinetic isotope effects were calculated using the Kinisot.py package,[5] reporting the overall value for all three methyl groups’ hydrogen atoms, assuming a temperature of 353.15 K. The method computes the solvation free energy of DMSO in DMSO to be 38.1 kJ mol^{-1}. Several publications cite a study by Lai et al. as supporting the

experimental value being 32.2 kJ mol^{-1}.[6-8]Re-examining the original study reveals that this value is arrived at from assumptions of ideal behaviour of DMSO in the gas and solution phases that are likely implausible. The same study also reveals that the effects of increasing the mole fraction of DMSO support both the enthalpy and entropy of addition of DMSO to DMSO as being approximately 0 kJ mol^{-1}.[6] Agreement of our calculations with experimental observation is significantly enhanced when an assumption is made that ΔG_{solv}(DMSO in DMSO) is approximately 0 kJ mol^{-1}. Notably, this ensures that the barrier of reaction of MeI with DMSO is in line with the observed formation of Me_3SO in the experiment and with tight solvation of the ion pair contrasting with weaker solvation of the self-immolation transition state, agreeing with the experimentally observed positive entropy of activation. We have therefore used the gas phase free energy of DMSO throughout (for both implicit and explicit solvation regimes). Values for Electronic energy (E), zero-point energy (ZPE), Enthalpy (H), entropic contribution TS, TS with a quasi-harmonic correction (T.qH-S), Gibbs Free Energy (G(T)) and corrected Gibbs free energy (qh-G(T)) are reported, as well as optimized geometries.

2. Reference State Analysis

The reference state for each anilinium salt was investigated. In particular, the optimal conformation for the ion pair was studied considering two different positions for the halide around the anilinium cation, namely: 'axial' and 'displaced. The first presente the counterion sitting on top of the three methyl groups and the latter has the halide on the side in between the $-NMe_3$ group and the ortho position of the benzene ring (Fig. S1). The two conformations were studied for unsubstituted trimethylanilinium with three different halides (Cl-, Br- and I-), also on substituted trimethylanilinium iodides (H-TMAI, 3Br-TMAI, 4CHO-TMAI). The 'displaced' conformation appeared to be slightly favoured (Table S1) and was considered the relevant geometry for all ion pairs.

Ion Pair association energy was studied for all the anilinium halides contemplated in the computational study. The energy of formation resolved the appropriate choice of reference state for each anilinium salt, in particular whether the lowest energy state was either the ion pair or the separated ions. For aniliniums showing negative ΔG values for the association of the ion pair, the reference state was considered as the tight ion pair, the contrary was true for the ones showing positives ΔG values. Also, we were able to assess in this way the trend in the formation of the ion pair with respect to the halides considered in the calculations. Iodide appeared to be more prone to form ion pairs compared to Bromide and Chloride respectively (Table S2).

FIGURE S1: Conformations of Ion pair considered: ‘Axial’ and ‘Displaced’

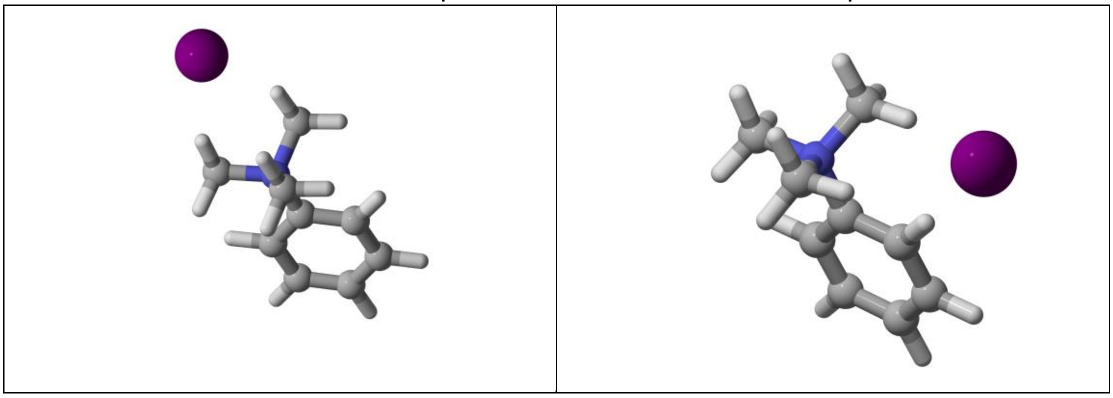

TABLE S1. A) Conformations analysis for trimethylanilinium halides ion pairs. Values are reported in kJ/mol for T = 80°C

Substituents on Ar	Halide	$\Delta G_{(displ\text{-}ax)}$ (kJ/mol)
None	I	-0.7
3-Br	I	-3.4
4-CHO	I	-1.9
None	Cl	-0.6
None	Br	-1.0

TABLE S2: Association energies for halides ion pairs. Values reported in kJ/mol.

T = 298.15 K	I	Cl	Br
H	-8.7	-0.9	-3.2
2Pyr	-3.3	7.4	5.2
4CHO	-12.3	-3.4	-6.7
4Bz	-7.7	0.5	-1.9
4F	-10.7	-1.8	-5.2
4Cl	-11.3	-1.7	-5.6
4Br	-10.3	-2.2	-5.9
4OMe	-10.9	-1.6	-4.5
3OMe	-10.1	-1.4	-4.6
3Cl	-12.6	-4.6	-7.0
3Br	-12.8	-4.8	-7.3
4Me	-8.2	-1.4	-3.0
3Me	-12.4	-2.1	-5.5
2Me	-0.5	1.6	6.1

T = 353.15 K	I	Cl	Br
H	-11.6	3.3	1.3
2Pyr	-6.5	11.7	9.6
4CHO	-15.4	0.8	-2.3
4Bz	-10.1	5.4	3.2
4F	-13.7	2.5	-0.7
4Cl	-14.4	2.7	-1.1
4Br	-13.2	2.1	-1.4
4OMe	-14.0	2.6	0.0
3OMe	-13.2	2.8	-0.2
3Cl	-15.7	-0.3	-2.4
3Br	-15.7	-0.6	-2.6
4Me	-11.1	2.8	1.5
3Me	-15.5	2.1	-1.0
2Me	-3.4	5.7	10.6

FIGURE S2. Set of possible reactions of anilinium salts.

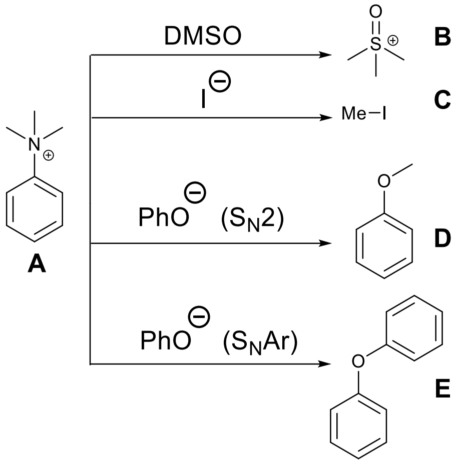

TABLE S3: Barrier and energy difference for S_N2 and S_NAr reaction for each anilinium. Results are reported for Iodide, Chloride and Bromide ion pairs, at 25 and 80°C. Values are in kJ/mol.

IODIDE 80°C	Barrier_S_N2(kJ/mol)	Barrier_S_NAr(kJ/mol)	ΔG_S_NAr(kJ/mol)	ΔG_S_N2(kJ/mol)
**H**	120.8	125.0	-82.5	-89.2
**2Pyr**	110.2	85.2	-96.1	-106.4
**4CHO**	114.3	77.3	-94.1	-113.6
**4Bz**	117.0	83.5	-91.4	-111.0
**4F**	122.2	129.3	-83.1	-89.6
**4Cl**	120.6	117.0	-85.8	-96.0
**4Br**	118.7	112.0	-86.4	-98.4
**4OMe**	125.0	143.9	-77.0	-74.3
**3OMe**	121.1	123.7	-83.2	-91.1
**3Cl**	117.2	109.7	-88.5	-100.7
**3Br**	118.4	100.6	-88.3	-101.2
**4Me**	122.9	135.3	-79.5	-86.3
**3Me**	124.1	130.0	-79.3	-85.5
**2Me**	110.9	110.6	-125.6	-102.4

IODIDE - 25°C	Barrier_S_N2(kJ/mol)	Barrier_S_NAr(kJ/mol)	ΔG_S_NAr(kJ/mol)	ΔG_S_N2(kJ/mol)
**H**	119.2	122.2	-75.6	-81.7
**2Pyr**	108.4	82.1	-89.3	-99.1
**4CHO**	112.6	74.6	-87.3	-106.1
**4Bz**	114.9	80.7	-84.6	-103.2
**4F**	120.4	126.5	-76.2	-82.1
**4Cl**	118.8	114.2	-78.9	-88.4
**4Br**	116.9	109.2	-79.5	-90.7
**4OMe**	123.2	141.2	-70.2	-66.5
**3OMe**	119.2	120.8	-76.3	-83.6

**3Cl**	115.6	106.8	-81.6	-93.1
**3Br**	116.8	97.8	-81.2	-93.4
**4Me**	121.2	132.4	-72.5	-78.4
**3Me**	122.3	127.1	-72.4	-78.0
**2Me**	109.3	108.0	-118.4	-94.8

**CHLORIDE - 80°C**	**Barrier_S_N2(kJ/mol)**	**Barrier_S_NAr(kJ/mol)**	**ΔG_S_NAr(kJ/mol)**	**ΔG_S_N2(kJ/mol)**
**H**	119.2	123.4	-84.1	-90.8
**2Pyr**	113.8	88.8	-92.6	-102.8
**4CHO**	108.9	72.0	-99.5	-119.0
**4Bz**	117.0	83.5	-91.4	-111.0
**4F**	118.5	125.6	-86.8	-93.3
**4Cl**	116.3	112.7	-90.1	-100.3
**4Br**	115.6	108.9	-89.6	-101.5
**4OMe**	121.1	140.0	-80.9	-78.2
**3OMe**	117.9	120.5	-86.3	-94.3
**3Cl**	111.9	104.3	-93.8	-106.0
**3Br**	113.4	95.5	-93.4	-106.3
**4Me**	121.9	134.2	-80.5	-87.3
**3Me**	118.6	124.6	-84.7	-91.0
**2Me**	117.5	117.3	-118.9	-95.8

**CHLORIDE - 25°C**	**Barrier_S_N2(kJ/mol)**	**Barrier_S_NAr(kJ/mol)**	**ΔG_S_NAr(kJ/mol)**	**ΔG_S_N2(kJ/mol)**
**H**	114.0	117.0	-80.9	-86.9
**2Pyr**	107.6	81.3	-90.1	-99.8
**4CHO**	106.2	68.2	-93.6	-112.4
**4Bz**	109.8	75.6	-89.7	-108.4
**4F**	114.0	120.1	-82.6	-88.5
**4Cl**	111.7	107.0	-86.0	-95.5
**4Br**	111.4	103.7	-85.0	-96.2
**4OMe**	116.5	134.4	-76.9	-73.3
**3OMe**	113.0	114.6	-82.4	-89.7
**3Cl**	110.0	101.3	-87.1	-98.6
**3Br**	111.3	92.4	-86.7	-98.8
**4Me**	116.9	128.2	-76.8	-82.7
**3Me**	114.7	119.4	-80.1	-85.6
**2Me**	111.5	110.1	-116.3	-92.7

**BROMIDE - 80°C**	**Barrier_S_N2(kJ/mol)**	**Barrier_S_NAr(kJ/mol)**	**ΔG_S_NAr(kJ/mol)**	**ΔG_S_N2(kJ/mol)**
**H**	119.7	123.8	-83.7	-90.4
**2Pyr**	114.3	89.2	-92.1	-102.4
**4CHO**	111.7	74.7	-96.7	-116.3
**4Bz**	117.4	83.9	-90.9	-110.6
**4F**	119.6	126.8	-85.6	-92.1

**4Cl**	117.8	114.2	-88.7	-98.8
**4Br**	117.4	110.7	-87.8	-99.7
**4OMe**	121.5	140.4	-80.5	-77.8
**3OMe**	118.5	121.2	-85.7	-93.7
**3Cl**	114.4	106.8	-91.3	-103.5
**3Br**	115.8	98.0	-90.9	-103.8
**4Me**	122.3	134.7	-80.1	-86.9
**3Me**	120.1	126.0	-83.3	-89.5
**2Me**	117.9	117.7	-118.5	-95.4

BROMIDE - 25°C	Barrier_S_N2(kJ/mol)	Barrier_S_NAr(kJ/mol)	ΔG_S_NAr(kJ/mol)	ΔG_S_N2(kJ/mol)
**H**	116.7	119.7	-78.1	-84.2
**2Pyr**	108.1	81.8	-89.6	-99.4
**4CHO**	110.0	72.0	-89.9	-108.7
**4Bz**	112.1	77.9	-87.4	-106.1
**4F**	117.8	123.9	-78.7	-84.6
**4Cl**	116.0	111.4	-81.7	-91.2
**4Br**	115.5	107.8	-80.9	-92.1
**4OMe**	119.8	137.7	-73.6	-70.0
**3OMe**	116.7	118.3	-78.8	-86.1
**3Cl**	112.9	104.1	-84.3	-95.8
**3Br**	114.2	95.2	-83.8	-96.0
**4Me**	119.0	130.2	-74.7	-80.6
**3Me**	118.4	123.2	-76.3	-81.9
**2Me**	111.9	110.5	-115.9	-92.3

TABLE S4. Degradation processes for trimethylanilinium halides in DMSO. Calculations have been performed for Iodide, Chloride and Bromide as counterions. Values are reported in kJ/mol. Legend: A = reference state; AB= transition state, DMSO attack on anilinium; B = Methylated DMSO; AC = Halide attack on anilinium; BC = transition state, halide attacking methylated DMSO; C = methylhalide.

I-/80°C	A	TS_AB	B	TS_AC	TS_BC	C
H	0.0	158.8	27.0	125.2	117.0	27.8
2Pyr	0.0	146.3	9.8	114.5	99.9	10.6
4CHO	0.0	153.7	2.6	116.5	92.7	3.4
4Bz	0.0	154.8	5.2	118.4	95.3	6.0
4F	0.0	161.0	26.6	123.6	116.7	27.4
4Cl	0.0	159.3	20.2	121.6	110.3	21.0
4Br	0.0	156.2	17.8	122.8	107.9	18.6
4OMe	0.0	164.1	41.9	127.5	131.9	42.7
3OMe	0.0	162.6	25.0	124.1	115.1	25.8
3Cl	0.0	156.1	15.5	120.3	105.6	16.3
3Br	0.0	157.0	15.0	121.2	105.1	15.8

**I-/25°C**	**A**	**TS_AB**	**B**	**TS_AC**	**TS_BC**	**C**
H	0.0	156.5	33.3	125.9	119.8	36.0
2Pyr	0.0	143.8	16.0	114.8	102.5	18.7
4CHO	0.0	151.4	9.0	117.3	95.5	11.7
4Bz	0.0	152.5	11.8	119.0	98.3	14.5
4F	0.0	158.6	32.9	124.4	119.4	35.6
4Cl	0.0	156.8	26.6	122.3	113.1	29.3
4Br	0.0	153.9	24.3	123.4	110.8	27.0
4OMe	0.0	161.7	48.4	128.2	134.9	51.1
3OMe	0.0	159.5	31.4	124.7	117.9	34.1
3Cl	0.0	153.8	21.9	121.1	108.4	24.7
3Br	0.0	154.9	21.6	122.0	108.1	24.4

4Me	0.0	162.8	29.9	127.0	120.0	30.7
3Me	0.0	NA	30.6	128.9	120.7	31.4
2Me	0.0	147.7	13.8	112.6	103.9	14.6

4Me	0.0	160.4	36.6	127.7	123.1	39.3
3Me	0.0	NA	37.0	129.5	123.5	39.7
2Me	0.0	145.1	20.2	113.0	106.7	22.9

**Cl-/ 80°C**	A	TS_AB	B	TS_AC	TS_BC	C
H	0.0	192.0	60.2	132.1	81.8	15.5
2Pyr	0.0	184.7	48.2	126.1	69.8	3.5
4CHO	0.0	183.2	32.1	120.9	53.7	-12.6
4Bz	0.0	189.7	40.0	127.7	61.7	-4.7
4F	0.0	192.1	57.8	130.3	79.4	13.1
4Cl	0.0	189.8	50.7	127.0	72.4	6.1
4Br	0.0	187.9	49.5	126.8	71.1	4.8
4OMe	0.0	195.0	72.8	133.5	94.4	28.1
3OMe	0.0	194.3	56.7	129.8	78.4	12.0
3Cl	0.0	185.6	45.0	124.0	66.7	0.3
3Br	0.0	186.8	44.7	123.9	66.4	0.0
4Me	0.0	196.6	63.7	135.7	85.3	19.0
3Me	0.0	NA	60.0	131.2	81.7	15.4
2Me	0.0	189.1	55.2	129.4	76.9	10.5

**Cl-/ 25°C**	A	TS_AB	B	TS_AC	TS_BC	C
H	0.0	148.8	25.5	129.1	43.7	20.0
2Pyr	0.0	140.5	12.7	122.1	30.9	7.2
4CHO	0.0	142.5	0.1	120.5	18.3	-5.5
4Bz	0.0	144.8	4.2	123.3	22.4	-1.4
4F	0.0	149.6	24.0	128.2	42.2	18.4
4Cl	0.0	147.2	16.9	124.9	35.1	11.4
4Br	0.0	145.9	16.3	125.2	34.5	10.7
4OMe	0.0	152.4	39.1	131.3	57.3	33.6
3OMe	0.0	150.8	22.7	127.4	40.9	17.2
3Cl	0.0	145.8	13.9	124.6	32.1	8.3
3Br	0.0	146.9	13.6	124.5	31.8	8.1
4Me	0.0	153.6	29.8	133.1	48.0	24.3
3Me	0.0	NA	26.8	129.6	45.0	21.3
2Me	0.0	144.6	19.7	125.3	37.9	14.2

**Br-/ 80°C**	A	TS_AB	B	TS_AC	TS_BC	C
H	0.0	192.0	60.2	129.3	80.9	25.9
2Pyr	0.0	184.7	48.2	122.8	68.9	13.9
4CHO	0.0	185.5	34.4	120.8	55.1	0.1
4Bz	0.0	189.7	40.0	124.4	60.7	5.8
4F	0.0	192.9	58.5	127.3	79.2	24.2
4Cl	0.0	190.8	51.8	125.4	72.5	17.5
4Br	0.0	189.3	50.9	125.4	71.6	16.6
4OMe	0.0	195.0	72.8	130.4	93.5	38.5
3OMe	0.0	194.5	56.9	126.5	77.6	22.7
3Cl	0.0	187.7	47.1	122.3	67.8	12.8
3Br	0.0	188.9	46.8	123.7	67.5	12.5
4Me	0.0	196.6	63.7	131.8	84.4	29.4
3Me	0.0	NA	61.1	129.9	81.8	26.8
2Me	0.0	189.1	55.2	125.8	75.9	21.0

**Br-/ 25°C**	A	TS_AB	B	TS_AC	TS_BC	C
H	0.0	151.1	27.8	128.6	44.9	32.8
2Pyr	0.0	140.5	12.7	118.8	29.7	17.6
4CHO	0.0	145.8	3.4	121.4	20.4	8.4
4Bz	0.0	146.7	6.0	121.8	23.1	11.0
4F	0.0	153.0	27.4	127.9	44.4	32.4
4Cl	0.0	151.1	20.8	126.0	37.9	25.8
4Br	0.0	149.6	19.9	126.0	37.0	24.9
4OMe	0.0	155.3	42.0	131.1	59.0	47.0
3OMe	0.0	154.0	25.9	127.1	42.9	30.9
3Cl	0.0	148.2	16.3	123.2	33.3	21.2
3Br	0.0	149.3	16.1	124.5	33.1	21.0
4Me	0.0	155.2	31.4	130.8	48.4	36.4
3Me	0.0	NA	30.1	130.5	47.2	35.1
2Me	0.0	144.6	19.7	121.7	36.8	24.7

TABLE S5. Sets of all considered reactions occurring for anilinium halide salts in DMSO. Values are reported for Iodide, Bromide and Chloride as halides. Data is reported at 25 and 80 °C. Values are expressed in kJ/mol. Legend: REF = reference state for the reactant (with IP being 'Ion Pair' and SEP 'Separated ions'); A = reference state; AB= transition state, DMSO attack on anilinium; B = Methylated DMSO; AC = Halide attack on anilinium; BC = transition state, halide attacking methylated DMSO; C = methylhalide; AD = phenolate + anilinium S_N2 reaction transition state; BD = methylated DMSO + phenolate S_N2 reaction transition state; CD = methylhalide + anilinium S_N2 reaction transition state; D = S_N2 product (Anisole); AE = anilinium + phenolate S_NAr reaction transition state. In all states reported the spectating species are considered as well, in particular the Phenolate standard state is Potassium Phenolate, when phenolate is involved in a transition structure or a product, potassium ion (K^+) is considered to be forming a salt with the free halide (X^-), hence in these cases the energy of the K^+X^- salt is considered.

I-/80°C	REF	A	TS_AB	B	TS_AC	TS_BC	C	TS_AD	TS_BD	TS_CD	D	TSAE	E
H	IP	0.0	158.8	27.0	125.2	117.0	27.8	120.4	105.8	125.1	-88.9	124.6	-82.3
2Pyr	IP	0.0	146.3	9.8	114.5	99.9	10.6	109.9	88.6	107.9	-106.1	85.0	-95.8
4CHO	IP	0.0	153.7	2.6	116.5	92.7	3.4	113.9	81.4	100.7	-113.3	77.1	-93.8
4Bz	IP	0.0	154.8	5.2	118.4	95.3	6.0	116.6	84.0	103.3	-110.7	83.2	-91.1
4F	IP	0.0	161.0	26.6	123.6	116.7	27.4	121.8	105.4	124.7	-89.3	128.9	-82.8
4Cl	IP	0.0	159.3	20.2	121.6	110.3	21.0	120.2	99.1	118.4	-95.6	116.6	-85.5
4Br	IP	0.0	156.2	17.8	122.8	107.9	18.6	118.4	96.6	115.9	-98.1	111.6	-86.2
4OMe	IP	0.0	164.1	41.9	127.5	131.9	42.7	124.6	120.7	140.0	-74.0	143.5	-76.8
3OMe	IP	0.0	162.6	25.0	124.1	115.1	25.8	120.7	103.9	123.2	-90.8	123.3	-82.9
3Cl	IP	0.0	156.1	15.5	120.3	105.6	16.3	116.9	94.4	113.7	-100.4	109.3	-88.2
3Br	IP	0.0	157.0	15.0	121.2	105.1	15.8	118.1	93.8	113.1	-100.9	100.2	-88.1
4Me	IP	0.0	162.8	29.9	127.0	120.0	30.7	122.5	108.7	128.0	-86.0	134.8	-79.2
3Me	IP	0.0	NA	30.6	128.9	120.7	31.4	123.7	109.5	128.8	-85.2	129.6	-79.0
2Me	IP	0.0	147.7	13.8	112.6	103.9	14.6	110.5	92.6	111.9	-102.1	110.2	-125.2

I-/25°C	REF	A	TS_AB	B	TS_AC	TS_BC	C	TS_AD	TS_BD	TS_CD	D	TSAE	E
H	IP	0.0	156.5	33.3	125.9	119.8	36.0	118.8	106.4	130.1	-81.4	121.8	-75.4
2Pyr	IP	0.0	143.8	16.0	114.8	102.5	18.7	108.1	89.1	112.8	-98.7	81.8	-89.0
4CHO	IP	0.0	151.4	9.0	117.3	95.5	11.7	112.2	82.1	105.8	-105.8	74.4	-87.0
4Bz	IP	0.0	152.5	11.8	119.0	98.3	14.5	114.6	85.0	108.7	-102.9	80.5	-84.3
4F	IP	0.0	158.6	32.9	124.4	119.4	35.6	120.0	106.1	129.8	-81.8	126.1	-75.9
4Cl	IP	0.0	156.8	26.6	122.3	113.1	29.3	118.4	99.8	123.5	-88.1	113.8	-78.6
4Br	IP	0.0	153.9	24.3	123.4	110.8	27.0	116.5	97.5	121.1	-90.4	108.9	-79.3
4OMe	IP	0.0	161.7	48.4	128.2	134.9	51.1	122.8	121.6	145.3	-66.3	140.7	-70.0
3OMe	IP	0.0	159.5	31.4	124.7	117.9	34.1	118.8	104.6	128.3	-83.3	120.4	-76.0
3Cl	IP	0.0	153.8	21.9	121.1	108.4	24.7	115.2	95.1	118.8	-92.8	106.4	-81.3
3Br	IP	0.0	154.9	21.6	122.0	108.1	24.4	116.4	94.8	118.5	-93.1	97.5	-81.0
4Me	IP	0.0	160.4	36.6	127.7	123.1	39.3	120.8	109.7	133.4	-78.2	132.0	-72.3

3Me	IP	0.0	NA	37.0	129.5	123.5	39.7	121.9	110.2	133.9	-77.7	126.7	-72.2
2Me	IP	0.0	145.1	20.2	113.0	106.7	22.9	109.0	93.4	117.1	-94.5	107.7	-118.1

**Cl-/ 80°C**	REF	A	TS_AB	B	TS_AC	TS_BC	C	TS_AD	TS_BD	TS_CD	D	TSAE	E
H	SEP	0.0	192.0	60.2	132.1	81.8	15.5	118.9	141.7	-55.1	-90.5	123.0	-83.8
2Pyr	SEP	0.0	184.7	48.2	126.1	69.8	3.5	113.5	129.7	-67.1	-102.5	88.5	-92.3
4CHO	SEP	0.0	183.2	32.1	120.9	53.7	-12.6	108.6	113.6	-83.2	-118.6	71.7	-99.1
4Bz	SEP	0.0	189.7	40.0	127.7	61.7	-4.7	116.6	121.5	-75.3	-110.7	83.2	-91.1
4F	SEP	0.0	192.1	57.8	130.3	79.4	13.1	118.1	139.2	-57.5	-93.0	125.2	-86.5
4Cl	SEP	0.0	189.8	50.7	127.0	72.4	6.1	115.9	132.2	-64.6	-100.0	112.3	-89.8
4Br	SEP	0.0	187.9	49.5	126.8	71.1	4.8	115.3	130.9	-65.8	-101.2	108.5	-89.3
4OMe	SEP	0.0	195.0	72.8	133.5	94.4	28.1	120.7	154.2	-42.5	-78.0	139.6	-80.7
3OMe	SEP	0.0	194.3	56.7	129.8	78.4	12.0	117.5	138.2	-58.6	-94.0	120.2	-86.0
3Cl	IP	0.0	185.6	45.0	124.0	66.7	0.3	111.5	126.5	-70.3	-105.7	104.0	-93.5
3Br	IP	0.0	186.8	44.7	123.9	66.4	0.0	113.0	126.2	-70.6	-106.0	95.2	-93.1
4Me	SEP	0.0	196.6	63.7	135.7	85.3	19.0	121.5	145.1	-51.6	-87.0	133.8	-80.2
3Me	SEP	0.0	NA	60.0	131.2	81.7	15.4	118.2	141.5	-55.3	-90.7	124.2	-84.4
2Me	SEP	0.0	189.1	55.2	129.4	76.9	10.5	117.1	136.7	-60.1	-95.5	116.9	-118.6

**Cl-/ 25°C**	**REF**	A	TS_AB	B	TS_AC	TS_BC	C	TS_AD	TS_BD	TS_CD	D	TSAE	E
H	IP	0.0	148.8	25.5	129.1	43.7	20.0	113.6	101.2	-53.4	-86.6	116.6	-80.6
2Pyr	SEP	0.0	140.5	12.7	122.1	30.9	7.2	107.3	88.4	-66.2	-99.5	81.1	-89.8
4CHO	IP	0.0	142.5	0.1	120.5	18.3	-5.5	105.9	75.8	-78.8	-112.1	68.0	-93.3
4Bz	SEP	0.0	144.8	4.2	123.3	22.4	-1.4	109.5	79.9	-74.8	-108.0	75.4	-89.4
4F	IP	0.0	149.6	24.0	128.2	42.2	18.4	113.6	99.7	-54.9	-88.2	119.7	-82.3
4Cl	IP	0.0	147.2	16.9	124.9	35.1	11.4	111.3	92.7	-62.0	-95.2	106.7	-85.7
4Br	IP	0.0	145.9	16.3	125.2	34.5	10.7	111.0	92.0	-62.7	-95.9	103.4	-84.8
4OMe	IP	0.0	152.4	39.1	131.3	57.3	33.6	116.1	114.8	-39.8	-73.0	134.0	-76.7
3OMe	IP	0.0	150.8	22.7	127.4	40.9	17.2	112.7	98.4	-56.2	-89.5	114.3	-82.2
3Cl	IP	0.0	145.8	13.9	124.6	32.1	8.3	109.7	89.6	-65.0	-98.3	100.9	-86.8
3Br	IP	0.0	146.9	13.6	124.5	31.8	8.1	111.0	89.4	-65.3	-98.5	92.1	-86.4
4Me	IP	0.0	153.6	29.8	133.1	48.0	24.3	116.6	105.5	-49.1	-82.4	127.8	-76.5
3Me	IP	0.0	NA	26.8	129.6	45.0	21.3	114.3	102.5	-52.1	-85.4	119.0	-79.8
2Me	SEP	0.0	144.6	19.7	125.3	37.9	14.2	111.1	95.5	-59.2	-92.4	109.8	-116.0

**Br-/ 80°C**	**REF**	A	TS_AB	B	TS_AC	TS_BC	C	TS_AD	TS_BD	TS_CD	D	TSAE	E
H	**SEP**	0.0	192.0	60.2	129.3	80.9	25.9	119.3	142.1	-60.9	-90.1	123.4	-83.4
2Pyr	**SEP**	0.0	184.7	48.2	122.8	68.9	13.9	113.9	130.1	-72.9	-102.1	89.0	-91.8
4CHO	**IP**	0.0	185.5	34.4	120.8	55.1	0.1	111.3	116.3	-86.7	-115.9	74.5	-96.4
4Bz	**SEP**	0.0	189.7	40.0	124.4	60.7	5.8	117.1	121.9	-81.1	-110.3	83.6	-90.7
4F	**IP**	0.0	192.9	58.5	127.3	79.2	24.2	119.3	140.4	-62.6	-91.8	126.4	-85.3

4Cl	**IP**	0.0	190.8	51.8	125.4	72.5	17.5	117.4	133.7	-69.3	-98.5	113.8	-88.4
4Br	**IP**	0.0	189.3	50.9	125.4	71.6	16.6	117.1	132.8	-70.2	-99.4	110.3	-87.5
4OMe	**IP**	0.0	195.0	72.8	130.4	93.5	38.5	121.1	154.7	-48.3	-77.5	140.0	-80.2
3OMe	**IP**	0.0	194.5	56.9	126.5	77.6	22.7	118.1	138.8	-64.2	-93.4	120.8	-85.4
3Cl	**IP**	0.0	187.7	47.1	122.3	67.8	12.8	114.0	129.0	-74.0	-103.2	106.5	-91.0
3Br	**IP**	0.0	188.9	46.8	123.7	67.5	12.5	115.5	128.7	-74.3	-103.5	97.7	-90.6
4Me	**SEP**	0.0	196.6	63.7	131.8	84.4	29.4	121.9	145.5	-57.4	-86.6	134.2	-79.8
3Me	**IP**	0.0	NA	61.1	129.9	81.8	26.8	119.7	142.9	-60.0	-89.2	125.6	-83.0
2Me	**SEP**	0.0	189.1	55.2	125.8	75.9	21.0	117.6	137.1	-65.8	-95.1	117.3	-118.1

**Br-/ 25°C**	**REF**	A	TS_AB	B	TS_AC	TS_BC	C	TS_AD	TS_BD	TS_CD	D	TSAE	E
H	IP	0.0	151.1	27.8	128.6	44.9	32.8	116.3	104.0	-57.1	-83.9	119.4	-77.9
2Pyr	SEP	0.0	140.5	12.7	118.8	29.7	17.6	107.7	88.8	-72.3	-99.1	81.5	-89.4
4CHO	IP	0.0	145.8	3.4	121.4	20.4	8.4	109.6	79.6	-81.5	-108.3	71.8	-89.6
4Bz	IP	0.0	146.7	6.0	121.8	23.1	11.0	111.8	82.2	-78.9	-105.7	77.7	-87.1
4F	IP	0.0	153.0	27.4	127.9	44.4	32.4	117.4	103.5	-57.5	-84.3	123.5	-78.5
4Cl	IP	0.0	151.1	20.8	126.0	37.9	25.8	115.6	97.0	-64.1	-90.9	111.0	-81.4
4Br	IP	0.0	149.6	19.9	126.0	37.0	24.9	115.1	96.1	-65.0	-91.8	107.5	-80.7
4OMe	IP	0.0	155.3	42.0	131.1	59.0	47.0	119.4	118.2	-42.9	-69.7	137.3	-73.4
3OMe	IP	0.0	154.0	25.9	127.1	42.9	30.9	116.3	102.1	-59.0	-85.8	117.9	-78.6
3Cl	IP	0.0	148.2	16.3	123.2	33.3	21.2	112.5	92.4	-68.7	-95.5	103.8	-84.0
3Br	IP	0.0	149.3	16.1	124.5	33.1	21.0	113.8	92.2	-68.9	-95.7	94.9	-83.6
4Me	IP	0.0	155.2	31.4	130.8	48.4	36.4	118.6	107.5	-53.5	-80.4	129.8	-74.5
3Me	IP	0.0	NA	30.1	130.5	47.2	35.1	118.1	106.3	-54.8	-81.6	122.8	-76.1
2Me	SEP	0.0	144.6	19.7	121.7	36.8	24.7	111.5	95.9	-65.2	-92.0	110.2	-115.5

3. Degradation Mechanistic Analysis

Four hypotheses for the mechanism of degradation of iodide salts were formulated with associated rate constants (***k***) and activation parameters. Namely one 'intra-ion pair' reaction ($\boldsymbol{k_1}$) and three 'inter-ion pair' reactions: **I-** attacking an ion pair ($\boldsymbol{k_2}$), anilinium attack on an ion pair ($\boldsymbol{k_3}$) and ion pair attacking an ion pair ($\boldsymbol{k_4}$).

Kinetic isotope effects (KIEs) were calculated for the four considered mechanisms of degradation. A *H9* to *D9* change was assumed, as per the experimentally accessible D9 analogues.

FIGURE S3. Transition state geometries for counterion-mediated Ion pair degradation mechanisms.

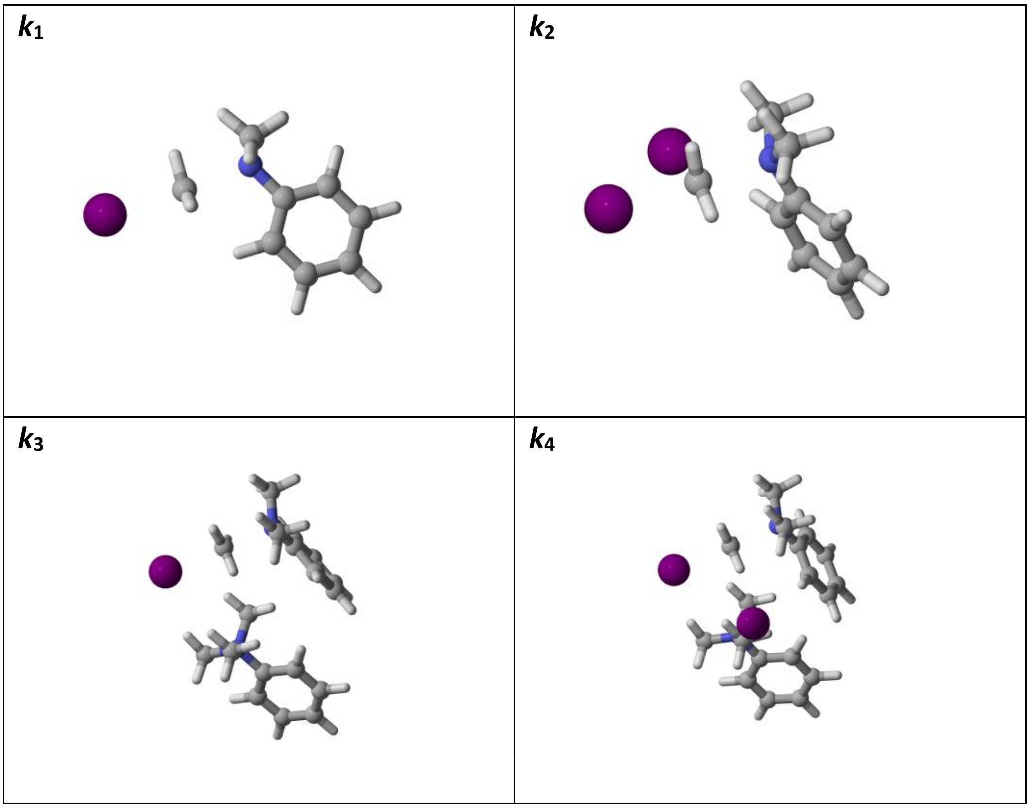

TABLE S6. Kinetic analysis of the four proposed mechanisms for TMAI degradation through counterion attack (implicit solvation only – those reported in the main text include explicit solvation). Kinetic Isotope effects are predicted considering a 9H-9D change. A) Trimethylanilinium Iodide. B) 3-Bromo-trimethylanilinium Iodide. C) 4-formyl-trimethylanilinium Iodide.

**S6A) H**	**K1**	**K2**	**K3**	**K4**
BARRIER (kJ/mol)	125.2	133.0	163.3	153.0
ΔG (kJ/mol)	27.8	27.8	27.8	27.8
KIE	1.40	1.48	1.26	1.30

**S6B) 3Br**	**K1**	**K2**	**K3**	**K4**
BARRIER (kJ/mol)	121.7	132.0	159.1	154.1
ΔG (kJ/mol)	16.2	16.2	16.2	16.2
KIE	1.47	1.42	1.30	1.29

**S6C) 4CHO**	**K1**	**K2**	**K3**	**K4**
BARRIER (kJ/mol)	116.7	127.3	152.4	146.4
ΔG (kJ/mol)	3.6	3.6	3.6	3.6
KIE	1.20	1.28	1.12	1.21

FIGURE S4. Energy diagrams for TMAI degradation reactions. a) H-TMAI b) m-Br-TMAI c) p-CHO-TMAI R1: two separated ion pairs. R2: One separated and one tight ion pair. R3: two tight ion pairs. P1 and P2 respectively stand for an associated ion pair in the products and separated ions. Values reported in kJ/mol.

S4A

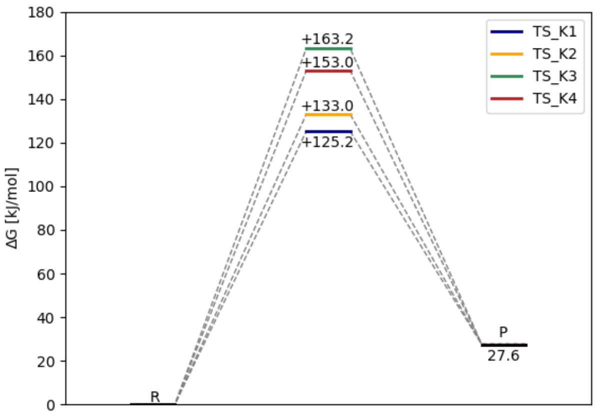

S4B

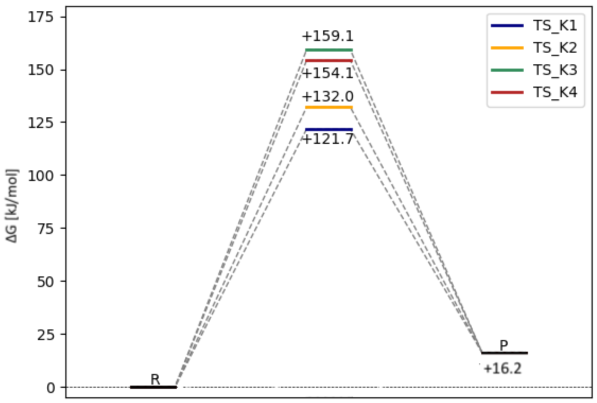

S4C

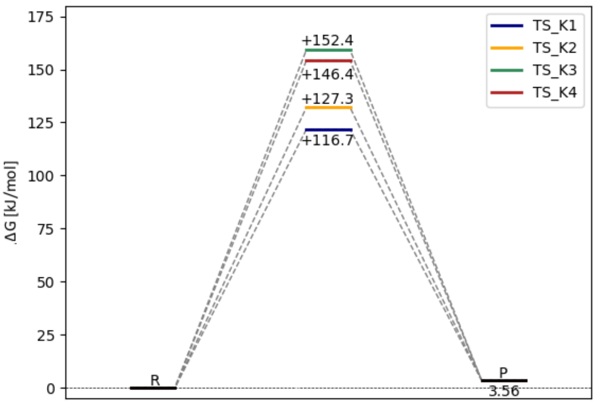

TABLE S7. Summary of the parameters for the four mechanisms proposed. A) Barrier heights for the transition state of each degradation process (data presented in kJ/mol). B) Predicted kinetic Isotope effects (KIE), T= 353.15 K

**S7A**	**$H_{\Delta G‡}$**	**$3Br_{\Delta G‡}$**	**$4CHO_{\Delta G‡}$**
**K1**	125.22	121.67	116.70
**K2**	132.97	131.98	127.31
**K3**	163.24	159.13	152.41
**K4**	153.02	154.09	146.42

**S7B**	**KIE_H**	**KIE_3Br**	**KIE_4CHO**
**K1**	1.40	1.47	1.20
**K2**	1.48	1.42	1.28
**K3**	1.26	1.30	1.12
**K4**	1.30	1.29	1.21

.

4. Higher Order Aggregate Analysis

Starting from the known crystallographic lattices of **H-TMAI**, an initial geometry for the dimer conformation was generated. Input geometries were created for **H-TMAI**, **3-Br-TMAI** and **4-CHO-TMAI**. For each anilinium salt, three different dispositions of the anilinium rings were considered. Moreover for the substituted examples, four different conformers were evaluated.

FIGURE S5. Disposition of the ion pair aggregate studied for the three different trimethylanilinium iodides.

**STACKED**	**ANTI**	**T-SHAPED**
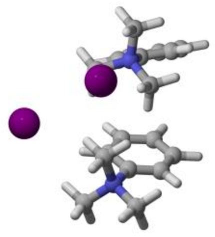	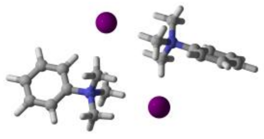	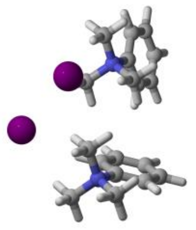

TABLE S8. Thermodynamic data for the three dispositions of dimeric aggregates considered relative to the lowest energy conformation. A) Trimethylanilinium Iodide. B) 4-formyl-trimethylanilinium Iodide. C) 3-Bromo-trimethylanilinium Iodide.

**S8A) H**	ΔG (kJ/mol)	ΔH (kJ/mol)
T-shaped (perpendicular)	11.5	11.3
Anti (displaced)	0.0	0
pi_pi (stacked)	7.4	5.0

**S8B) 4CHO**	ΔG (kJ/mol)	ΔH (kJ/mol)
T-shaped (perpendicular)	14.9	11.7
Anti (displaced)	0.0	0.0
pi_pi (stacked)	8.4	5.9

**S8C) 3Br**	ΔG (kJ/mol)	ΔH (kJ/mol)
T-shaped (perpendicular)	6.7	2.5
Anti (displaced)	0.0	0.0
pi_pi (stacked)	3.0	0.2

TABLE S9. Dimerization free energies for TMAI ion pairs

TMAI	ΔG (kJ/mol)
3Br	23.3
H	21.8
4CHO	23.3

5. Single Point Analysis

The system was analysed at different values for the dielectric constant with single point calculations at m062x/6-31+G* and LANL2DZ. Free energy for the single point files was calculated by adding the free energy correction, from the frequency calculations, to the electronic energy.

TABLE S10. Dielectric Constant values and solvent models considered for the single point analysis.

**Entry**	**Solvent Model for DMSO**	**ε**
**SP1**	SMD	47
**SP2**	IEF-PCM	47
**SP3**	IEF-PCM	52
**SP4**	IEF-PCM	42

TABLE S11. Gibbs Free energy of ion pair association for Trimethylanilium Iodide in different conditions.

T= 353.15 K

ION PAIR ASSOCIATION ΔG (kJ/mol)	SP1	SP2	SP3	SP4
**3Br**	4.7	-8.7	-8.2	-9.4
**4CHO**	5.2	-8.5	-7.9	-9.3
**H**	11.1	-2.4	-1.8	-3.1

FIGURE S6. Free energy of ion pair association with respect to the dielectric constant value.

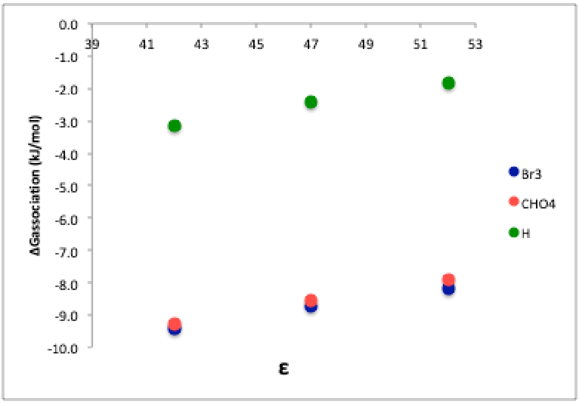

TABLE S12. Kinetic analysis of Iodide mediated degradation of TMAI salts. K1: intramolecular mechanism; K2 = intermolecular mechanism. T = 353.15 K.

**Mechanism Analysis (kJ/mol)**	**SP1**		**SP2**		**SP3**		**SP4**	
	**ΔG** $_{TS\_K1}$	**ΔG** $_{TS\_K2}$	**ΔG** $_{TS\_K1}$	**ΔG** $_{TS\_K2}$	**ΔG** $_{TS\_K1}$	**ΔG** $_{TS\_K2}$	**ΔG** $_{TS\_K1}$	**ΔG** $_{TS\_K2}$
**Br3**	136.7	144.7	120.9	138.5	121.2	138.3	120.6	138.8
**CHO4**	132.6	140.1	116.3	132.9	116.5	132.7	116.0	133.3
**H**	140.5	146.5	123.9	139.3	124.2	139.0	123.7	139.7

FIGURE S7. Free energy of ion pair association with respect to the dielectric constant value for 3-bromo-N,N,N-trimethylanilinium iodide.

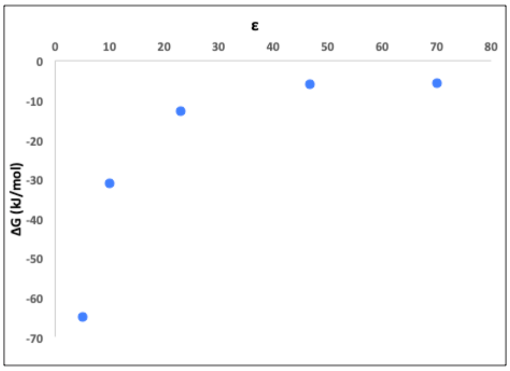

TABLE S13. Free energy of ion pairing data across a range of solvent dielectric constants for 3-bromo-N,N,N-trimethylanilinium iodide.

**ε value**	**5**	**10**	**23**	**46.7**	**70**
**Iodide** (Hartree)	-11.517378	-11.527561	-11.533393	-11.53557	11.536292
**Ion pair** (Hartree)	-2988.032227	-2988.037988	2988.041691	-2988.04311	2988.0443
**Anilinium** (Hartree)	-2976.490099	-2976.498536	2976.503396	2976.505224	2976.5058
**ΔG (kJ/mol)**	-64.98	-31.22	-12.87	-6.08	-5.67

6. Explicit solvation

Given the observation of a positive entropy of activation that is ascribed to tighter solvation of the reactants than the transition state (and also of the products), explicit solvation of each of the species studied for the first and second order degradation processes of the three exemplar anilinium salts. In Table S14, the interaction free energy of a DMSO with each of the relevant minima and transition states is reported at 80 and 120˚C. The resulting free energy of activation when explicit solvation is included whenever appropriate is then given in Table S15.

TABLE S14. Free energy of explicit solvation of species involved in the putative first and second order processes.

Species	Solvation free energy (kJ/mol) at 80˚C [120˚C]		
	H	3-Br	4-CHO
Ion pair (IP)	-18.3 [-4.4]	-18.8 [-4.8]	-18.4 [-4.5]
Anilinium	-17.7 [-4.1]	-20.4 [-6.3]	-20.1 [-6.5]
Self-immolation TS (1st order reaction)	-12.9 [+1.1]	-15.9 [-1.8]	-12.2 [+1.9]
TS(IP + I^-)	-12.8 [+0.9]	-23.1 [-8.9]	-19.9 [-5.6]
TS(IP + Anilinium)	-11.6 [+2.3]	-18.5 [-4.4]	-20.6 [-6.9]
TS(IP + IP)	-22.1 [-7.8]	-30.4 [-16.0]	-18.9 [-4.6]

TABLE S15. Free energy of activation for putative first and second order processes including explicit solvation.

Species	Activation free energy (kJ/mol) at 80˚C [120˚C]		
	H	3-Br	4-CHO
Self-immolation (1st order reaction)	130.7 [129.1]	124.7 [124.2]	122.9 [120.7]
IP + I^-	139.0 [137.3]	126.2 [126.3]	124.2 [124.1]
IP + Anilinium	188.3 [175.2]	178.3 [168.0]	168.7 [158.3]
IP + IP	167.5 [160.8]	161.4 [155.0]	164.4 [158.1]

7. Geometries

The structures below are organized in alphabetical order. The first line is the title of the original calculation that should be descriptive of the relative structure. For clarification it is worth mentioning that:
3Br, Br1, Br2, Br all refer to 3-Bromo substituted species.
4CHO, CHO2, CHO4, CHO, all refer to 4-formyl substituted species
Ph and H refer to unsubstituted species on the aromatic ring.
For calculation where explicit solvation was used, 'DMSO' is included in the title.

Values are reported from GoodVibes output in Hartree for the corrected free energy at 25 and 80°C. For DMSO the concentration was set to 14.1 mol/L and it is reported in the title of the relative structures as well as the indication of gas-phase (GP) rather than implicit solvent (PCM) calculation.

2Me_SnArProduct_GEN
Electronic energy = -577.610189
Thermal correction to Gibbs free energy (25°C) = 0.046805
Thermal correction to Gibbs free energy (80°C) = 0.059898
qh-G(25°C) = -577.429979
qh-G(80°C) = -577.438511

Geometry:

C	-2.493213	-0.408500	1.568880
C	-1.271536	-0.441551	0.893329
C	-1.253932	-0.221696	-0.484087
C	-2.437776	0.022502	-1.183090
C	-3.647142	0.047957	-0.495387
C	-3.682577	-0.165530	0.884920
H	-2.506689	-0.577003	2.641181
H	-0.349485	-0.632609	1.432167
H	-2.391108	0.190200	-2.254218
H	-4.565782	0.238876	-1.041189
H	-4.626555	-0.141835	1.418722
O	-0.104329	-0.251862	-1.238645
C	1.115888	-0.232844	-0.580837
C	1.590752	0.971095	-0.046449
C	1.854469	-1.409461	-0.531587
C	2.851236	0.947914	0.557562
C	3.110324	-1.405193	0.074972
H	1.436480	-2.311340	-0.967537
C	3.606867	-0.223613	0.623273
H	3.246711	1.869424	0.976494
H	3.693575	-2.319162	0.117294
H	4.583512	-0.209970	1.096180
C	0.767292	2.228192	-0.126814
H	0.336472	2.350691	-1.124560

H -0.065867 2.199462 0.584212
H 1.379467 3.102536 0.102301

2Me_TMA_DMSO_tsopt_GEN
Electronic energy = -998.119883
Thermal correction to Gibbs free energy (25°C) = 0.058775
Thermal correction to Gibbs free energy (80°C) = 0.076054
qh-G(25°C) = -997.833767
qh-G(80°C) = -997.844533

Geometry:
C -3.822220 -1.524996 -0.805849
C -3.763058 -0.288042 -1.437753
C -2.818403 0.651187 -1.033304
C -1.918955 0.379225 0.006730
C -1.965471 -0.877071 0.652489
C -2.926755 -1.799670 0.222270
H -4.549086 -2.270506 -1.110606
H -4.445330 -0.043531 -2.245064
H -2.959779 -2.767893 0.714687
N -0.927655 1.369518 0.383340
C -1.064084 1.825712 1.783884
H -0.208765 2.458783 2.031198
H -1.984101 2.410673 1.895326
H -1.094533 0.986306 2.472415
C -0.888645 2.561274 -0.480166
H -0.058034 3.188125 -0.150173
H -0.725685 2.272576 -1.519841
H -1.812267 3.144606 -0.401642
C 0.986335 0.569056 0.042486
H 0.509321 -0.349940 -0.276506
H 1.157756 1.354014 -0.682860
H 1.229656 0.742183 1.083678
S 2.988873 -0.304253 -0.406080
O 3.175374 -0.684939 -1.843364
C 3.267861 -1.733332 0.637468
H 2.508302 -2.472757 0.380512
H 4.267727 -2.115857 0.425083
H 3.172352 -1.430731 1.681832
C 4.312166 0.790320 0.104135
H 5.258602 0.284541 -0.095440
H 4.226040 1.699161 -0.491596
H 4.198990 1.012062 1.166673
H -2.800629 1.606195 -1.540930
C -1.074936 -1.318868 1.794643
H -0.111742 -0.814067 1.832606
H -1.570043 -1.150875 2.757337
H -0.883139 -2.390910 1.711940

2Me_TMA_I_TS
Electronic energy = -456.563468
Thermal correction to Gibbs free energy (25°C) = 0.051261
Thermal correction to Gibbs free energy (80°C) = 0.065529
qh-G(25°C) = -456.357234
qh-G(80°C) = -456.366631

Geometry:

C	-4.117525	-1.633833	-0.669191
C	-3.108617	-1.894117	0.253602
C	-2.138680	-0.945999	0.601219
C	-2.218652	0.330815	-0.001856
C	-3.234130	0.588574	-0.931999
C	-4.174125	-0.382023	-1.269977
H	-4.843944	-2.401569	-0.914383
H	-3.054204	-2.874253	0.719565
H	-4.946869	-0.147469	-1.994669
N	-1.261190	1.368948	0.327603
C	-1.436695	1.892988	1.696648
H	-0.558326	2.485927	1.963979
H	-2.328306	2.530291	1.742351
H	-1.555777	1.083611	2.412850
C	-1.241917	2.504296	-0.605972
H	-0.435855	3.177005	-0.306849
H	-1.056079	2.150314	-1.621988
H	-2.177798	3.073107	-0.577582
C	0.726920	0.635456	0.085494
I	3.117852	-0.295074	-0.230519
H	1.020118	1.595861	-0.307990
H	0.789504	0.456526	1.145533
H	0.329671	-0.116776	-0.578120
C	-1.053398	-1.406465	1.551455
H	-0.177430	-1.763330	0.998739
H	-0.709053	-0.643828	2.248901
H	-1.423962	-2.245748	2.143298
H	-3.308603	1.558467	-1.405897

2Me_TMA_SN2_tsopt_GEN_3
Electronic energy = -751.934310
Thermal correction to Gibbs free energy (25°C) = 0.059616
Thermal correction to Gibbs free energy (80°C) = 0.077115
qh-G(25°C) = -751.636940
qh-G(80°C) = -751.647756

Geometry:

C	4.269360	1.671037	-0.615507

C	3.987087	0.420326	-1.153397
C	2.974407	-0.413615	-0.660573
C	2.214751	0.057585	0.437046
C	2.501361	1.319817	0.975328
C	3.515846	2.121872	0.460428
H	5.062611	2.280222	-1.035710
H	4.570006	0.063309	-1.997837
H	1.940883	1.700045	1.817474
H	3.705780	3.091765	0.907757
N	1.103154	-0.709589	0.989217
C	0.429562	-0.070516	2.138162
H	-0.398805	-0.714751	2.436176
H	1.112994	0.041606	2.984749
H	0.025354	0.901429	1.854423
C	1.474213	-2.081318	1.410789
H	1.908186	-2.646887	0.594790
H	2.187857	-2.032865	2.239270
H	0.569131	-2.591504	1.745781
C	-2.861137	-0.211370	-0.931308
C	-3.114474	1.181314	-0.980170
C	-3.742040	-1.000080	-0.151511
C	-4.198398	1.742771	-0.308548
H	-2.445216	1.803947	-1.569175
C	-4.823158	-0.429561	0.517192
H	-3.560719	-2.070945	-0.097593
C	-5.064092	0.945347	0.445361
H	-4.369765	2.814334	-0.373525
H	-5.485189	-1.064348	1.100812
H	-5.908283	1.385968	0.965919
O	-1.825141	-0.744100	-1.550355
C	2.847191	-1.755672	-1.355286
H	3.482755	-2.503138	-0.868213
H	1.837559	-2.159732	-1.393745
H	3.196133	-1.657264	-2.384889
C	-0.321955	-0.746399	-0.300824
H	-0.912937	-1.404196	0.323503
H	-0.439762	0.327011	-0.231256
H	0.269324	-1.153311	-1.103479

2Me_TMA_SnAr_tsopt_GEN_3
Electronic energy = -751.937539
Thermal correction to Gibbs free energy (25°C) = 0.056652
Thermal correction to Gibbs free energy (80°C) = 0.073696
qh-G(25°C) = -751.637446
qh-G(80°C) = -751.647859

Geometry:

C	-0.685292	2.858686	-0.895609

C	-0.298209	1.839786	-1.760363
C	-0.434284	0.502906	-1.407080
C	-0.935462	0.096701	-0.135303
C	-1.324271	1.152978	0.764338
C	-1.173525	2.474819	0.357826
H	-0.594236	3.904372	-1.166481
H	0.121726	2.072583	-2.735864
H	-0.070029	-0.249882	-2.095678
H	-1.440097	3.245111	1.079534
N	-1.946071	-1.154328	-0.245981
C	-3.259048	-0.587938	-0.679383
H	-3.947907	-1.412759	-0.866816
H	-3.644703	0.055497	0.110843
H	-3.104164	-0.004986	-1.586728
C	-2.169061	-1.944987	1.009775
H	-2.906701	-2.715292	0.779540
H	-1.227363	-2.388682	1.314963
H	-2.560487	-1.301288	1.788136
C	-1.485731	-2.124863	-1.288268
H	-1.557060	-1.666862	-2.271879
H	-0.460376	-2.408862	-1.057985
H	-2.142889	-2.993881	-1.250184
O	0.274192	-0.950232	0.671304
C	1.543125	-0.617025	0.437213
C	2.026968	0.696750	0.605861
C	2.450328	-1.607240	0.009995
C	3.367716	0.992701	0.367357
H	1.336932	1.475887	0.916416
C	3.792836	-1.304434	-0.209109
C	4.263609	-0.000940	-0.034939
H	3.718214	2.012527	0.503201
H	4.473814	-2.090449	-0.524342
H	5.307990	0.236354	-0.210607
H	2.078448	-2.618654	-0.132424
C	-1.747104	0.901644	2.195071
H	-1.597854	1.812912	2.779651
H	-2.804972	0.631774	2.304232
H	-1.144871	0.109460	2.649620

2Me_TMA_bromide_tsopt
Electronic energy = -3016.704681
Thermal correction to Gibbs free energy (25°C) = 0.050273
Thermal correction to Gibbs free energy (80°C) = 0.064361
qh-G(25°C) = -3016.497111
qh-G(80°C) = -3016.506349

Geometry:

C	-3.697141	-1.468583	-0.662343

C -2.679371 -1.824713 0.217557
C -1.643256 -0.950832 0.568984
C -1.662506 0.350357 0.015718
C -2.687272 0.704984 -0.870979
C -3.694915 -0.193185 -1.214105
H -4.476325 -2.181041 -0.912269
H -2.671801 -2.823452 0.645534
H -4.472869 0.115480 -1.904607
N -0.632204 1.317484 0.351462
C -0.742277 1.802856 1.742376
H 0.170338 2.346107 2.000353
H -1.601319 2.477866 1.836460
H -0.876358 0.975775 2.435345
C -0.576949 2.484609 -0.542066
H 0.276531 3.097818 -0.247111
H -0.444603 2.158501 -1.575651
H -1.476317 3.104246 -0.460826
C 1.277971 0.492291 0.030611
H 1.616713 1.451768 -0.325780
H 1.359203 0.259044 1.078531
H 0.826826 -0.206856 -0.656158
C -0.563958 -1.511812 1.471014
H 0.273078 -1.901216 0.881695
H -0.155261 -0.797918 2.185029
H -0.971063 -2.347600 2.043389
H -2.716451 1.694905 -1.306712
Br 3.422376 -0.456047 -0.363373

2Me_TMA_chloride_tsopt
Electronic energy = -905.383174
Thermal correction to Gibbs free energy (25°C) = 0.049151
Thermal correction to Gibbs free energy (80°C) = 0.063028
qh-G(25°C) = -905.174117
qh-G(80°C) = -905.183165

Geometry:
C -3.268977 -1.142409 -0.605908
C -2.242763 -1.682100 0.163707
C -1.086652 -0.964201 0.494099
C -0.987189 0.370486 0.037905
C -2.021066 0.909974 -0.738153
C -3.151286 0.164114 -1.063925
H -4.143948 -1.738337 -0.843588
H -2.326080 -2.705897 0.517978
H -3.932361 0.614606 -1.667421
N 0.173217 1.185117 0.360782
C 0.213183 1.567235 1.787943
H 1.195831 1.989593 2.012700

H	-0.557849	2.318272	1.996176
H	0.037641	0.705534	2.427409
C	0.298797	2.408567	-0.447241
H	1.234188	2.900972	-0.175485
H	0.321976	2.154610	-1.508844
H	-0.516949	3.112223	-0.251702
C	1.934943	0.190565	-0.150223
H	2.364341	1.134313	-0.445499
H	2.047043	-0.145426	0.866395
H	1.369013	-0.388962	-0.863138
C	-0.024516	-1.716990	1.267667
H	0.723146	-2.142575	0.590256
H	0.507366	-1.117333	2.005307
H	-0.488851	-2.549752	1.799327
H	-1.961689	1.928251	-1.098672
Cl	3.819284	-0.882197	-0.727170

2Me_TMA_gen
Electronic energy = -445.082893
Thermal correction to Gibbs free energy (25°C) = 0.043584
Thermal correction to Gibbs free energy (80°C) = 0.056008
qh-G(25°C) = -444.867376
qh-G(80°C) = -444.875470

Geometry:

C	2.224481	0.809502	0.022834
C	0.829664	0.955258	0.010299
C	0.072915	-0.234851	-0.015799
C	0.702251	-1.480885	-0.032144
C	2.089970	-1.580835	-0.024979
C	2.859525	-0.425920	0.004159
H	2.825647	1.713134	0.045460
H	0.137258	-2.400524	-0.053613
H	2.551086	-2.561888	-0.040000
H	3.942744	-0.480006	0.013954
C	0.330075	2.386091	-0.021470
H	1.116503	3.038433	0.359782
H	0.119823	2.698631	-1.048911
H	-0.558704	2.579685	0.577319
N	-1.434932	-0.207976	0.004816
C	-2.048113	-1.565588	-0.214495
H	-1.764045	-2.232167	0.595839
H	-3.128043	-1.431518	-0.206512
H	-1.726887	-1.954275	-1.179562
C	-1.917213	0.262264	1.353274
H	-3.005425	0.315663	1.323842
H	-1.587849	-0.460599	2.098334
H	-1.499712	1.238876	1.575010

C -1.981554 0.672373 -1.091181
H -1.780231 1.713233 -0.870133
H -1.511491 0.378049 -2.028922
H -3.058158 0.516650 -1.138599

2Me_aniline_gen
Electronic energy = -405.355808
Thermal correction to Gibbs free energy (25°C) = 0.041985
Thermal correction to Gibbs free energy (80°C) = 0.053553
qh-G(25°C) = -405.184421
qh-G(80°C) = -405.192174

Geometry:
C 1.837580 1.071492 0.108297
C 0.443731 0.929751 0.071836
C -0.092491 -0.360970 -0.109895
C 0.775130 -1.457102 -0.205851
C 2.156910 -1.300506 -0.144487
C 2.693652 -0.022017 0.002303
H 2.254663 2.066473 0.243577
H 0.334827 -2.441088 -0.340446
H 2.806686 -2.166518 -0.223272
H 3.768619 0.123506 0.042306
C -0.421833 2.153874 0.250406
H 0.169965 2.972022 0.666954
H -0.842959 2.497300 -0.699889
H -1.257034 1.960220 0.928086
N -1.492391 -0.651972 -0.178869
C -2.114825 -0.929367 1.108488
H -2.238774 -0.025841 1.729873
H -3.105442 -1.363921 0.941363
H -1.510979 -1.650899 1.663804
C -2.324229 0.164465 -1.050280
H -3.189260 -0.429421 -1.365101
H -2.705759 1.081240 -0.576536
H -1.759559 0.443015 -1.943533

2Me_bromide_ionpair_gen_2
Electronic energy = -3016.753300
Thermal correction to Gibbs free energy (25°C) = 0.049910
Thermal correction to Gibbs free energy (80°C) = 0.063799
qh-G(25°C) = -3016.541121
qh-G(80°C) = -3016.550200

Geometry:
Br 3.344875 0.277604 -0.078541
N -0.667547 -1.372901 0.083834

C -1.284411 0.007584 0.062646
C -2.657498 0.291167 -0.070856
C -3.007318 1.654786 -0.044596
H -4.060077 1.903155 -0.133221
C -2.084089 2.683033 0.073676
H -2.419586 3.714545 0.086197
C -0.731640 2.370020 0.156840
H 0.022910 3.146026 0.226176
C 0.253049 -1.499865 1.276807
H 0.608635 -2.529325 1.308004
H -0.322003 -1.264975 2.171781
H 1.103635 -0.831521 1.162311
C 0.132794 -1.568254 -1.179026
H 0.546741 -2.577065 -1.160971
H 0.940637 -0.837072 -1.202390
H -0.538628 -1.443619 -2.028295
C -1.635600 -2.511580 0.191171
H -1.047004 -3.425235 0.253872
H -2.261827 -2.550878 -0.693583
H -2.223399 -2.393822 1.098524
C -0.342541 1.038753 0.148744
H 0.719855 0.819605 0.194813
C -3.818444 -0.661151 -0.290560
H -3.717226 -1.220209 -1.223478
H -3.963694 -1.369735 0.524639
H -4.732595 -0.072656 -0.371350

2Me_bromide_ionpair_gen_3
Electronic energy = -3016.753300
Thermal correction to Gibbs free energy (25°C) = 0.049910
Thermal correction to Gibbs free energy (80°C) = 0.063799
qh-G(25°C) = -3016.541121
qh-G(80°C) = -3016.550200

Geometry:
Br 3.344876 0.277604 -0.078541
N -0.667547 -1.372901 0.083834
C -1.284412 0.007584 0.062646
C -2.657498 0.291168 -0.070855
C -3.007317 1.654787 -0.044596
H -4.060076 1.903156 -0.133221
C -2.084088 2.683033 0.073676
H -2.419585 3.714546 0.086197
C -0.731639 2.370020 0.156839
H 0.022911 3.146025 0.226175
C 0.253049 -1.499865 1.276807
H 0.608634 -2.529326 1.308003
H -0.322003 -1.264975 2.171781

H	1.103635	-0.831522	1.162310
C	0.132793	-1.568255	-1.179026
H	0.546740	-2.577066	-1.160971
H	0.940636	-0.837072	-1.202390
H	-0.538629	-1.443619	-2.028295
C	-1.635601	-2.511579	0.191171
H	-1.047005	-3.425235	0.253871
H	-2.261828	-2.550877	-0.693583
H	-2.223399	-2.393822	1.098524
C	-0.342541	1.038753	0.148743
H	0.719856	0.819604	0.194812
C	-3.818444	-0.661151	-0.290559
H	-3.717227	-1.220209	-1.223478
H	-3.963694	-1.369734	0.524640
H	-4.732595	-0.072655	-0.371348

2Me_chloride_ionpair_gen_2
Electronic energy = -905.433556
Thermal correction to Gibbs free energy (25°C) = 0.049409
Thermal correction to Gibbs free energy (80°C) = 0.063282
qh-G(25°C) = -905.221266
qh-G(80°C) = -905.230294

Geometry:

Cl	3.338451	0.976735	-0.702010
N	0.187233	-1.343330	0.334506
C	-0.559301	-0.035775	0.273981
C	-1.788473	0.080410	-0.408309
C	-2.384540	1.349936	-0.415498
H	-3.331525	1.460122	-0.934955
C	-1.819498	2.456408	0.206528
H	-2.323674	3.416200	0.170590
C	-0.603078	2.315787	0.860875
H	-0.126965	3.160428	1.346272
C	1.352065	-1.303053	1.290919
H	1.795703	-2.297309	1.286337
H	0.984648	-1.063343	2.288123
H	2.083165	-0.577770	0.933878
C	0.767239	-1.655099	-1.022409
H	1.237236	-2.637633	-0.968878
H	1.505766	-0.882308	-1.244089
H	-0.023800	-1.657759	-1.766228
C	-0.703405	-2.460802	0.809995
H	-0.073162	-3.322828	1.023197
H	-1.419082	-2.725266	0.041536
H	-1.212128	-2.129180	1.714955
C	0.022520	1.073522	0.889189
H	0.979439	1.003886	1.383737

C -2.572784 -1.007648 -1.114608
H -1.967293 -1.709241 -1.687507
H -3.172736 -1.582042 -0.402332
H -3.264367 -0.539259 -1.815989

2Pyr_SnArProduct_GEN
Electronic energy = -554.353889
Thermal correction to Gibbs free energy (25°C) = 0.043899
Thermal correction to Gibbs free energy (80°C) = 0.055771
qh-G(25°C) = -554.211911
qh-G(80°C) = -554.219888

Geometry:
C 2.880013 0.864103 1.030819
C 1.613756 0.297720 1.160971
C 1.148044 -0.549522 0.159926
C 1.917927 -0.846688 -0.957631
C 3.186652 -0.277125 -1.076654
C 3.668307 0.578682 -0.086458
H 3.252827 1.526592 1.805377
H 0.986509 0.506192 2.021926
H 1.522204 -1.514966 -1.715405
H 3.796634 -0.504521 -1.944992
H 4.654992 1.019811 -0.181910
O -0.079196 -1.182744 0.307677
C -1.222075 -0.464734 0.102846
C -2.422299 -1.114436 0.422985
C -3.598868 -0.417862 0.209363
H -2.402843 -2.121734 0.821753
C -2.294797 1.419435 -0.583580
C -3.542544 0.883007 -0.305702
H -4.553176 -0.878771 0.442807
H -2.199789 2.424444 -0.985399
H -4.441339 1.460868 -0.484648
N -1.141020 0.758365 -0.386034

2Pyr_TMA_DMSO_tsopt_GEN
Electronic energy = -974.873109
Thermal correction to Gibbs free energy (25°C) = 0.056954
Thermal correction to Gibbs free energy (80°C) = 0.073227
qh-G(25°C) = -974.627244
qh-G(80°C) = -974.637607

Geometry:
C 3.483320 -1.963299 0.019700
C 2.601329 -1.655577 1.052642
C 1.954756 0.302627 0.053835

C	2.806799	0.090641	-1.034435
C	3.579972	-1.067297	-1.039875
H	4.071051	-2.872934	0.051136
H	2.487827	-2.323386	1.902177
H	2.886279	0.800120	-1.847023
H	4.255720	-1.258046	-1.867017
N	1.074418	1.428931	0.132147
C	1.178982	2.397311	-0.963383
H	0.415559	3.162675	-0.816119
H	2.163016	2.879324	-0.979061
H	0.996821	1.905834	-1.920767
C	1.082791	2.095709	1.447967
H	0.277401	2.832036	1.468487
H	0.925215	1.356857	2.230874
H	2.039544	2.604131	1.609758
C	-0.817781	0.590432	-0.022866
H	-0.548103	0.226422	-1.008261
H	-0.565659	0.003996	0.854135
H	-1.236560	1.585755	0.076916
S	-2.853388	-0.259908	-0.211038
O	-3.685547	0.397402	-1.269406
C	-3.709098	-0.185447	1.360454
H	-3.781105	0.867000	1.636938
H	-4.701651	-0.617349	1.220430
H	-3.136430	-0.741540	2.104454
C	-2.766572	-2.022439	-0.517518
H	-3.784515	-2.415037	-0.535224
H	-2.286157	-2.152624	-1.487694
H	-2.174312	-2.490614	0.270397
N	1.849533	-0.553113	1.071880

2Pyr_TMA_I_TS
Electronic energy = -433.316693
Thermal correction to Gibbs free energy (25°C) = 0.048703
Thermal correction to Gibbs free energy (80°C) = 0.061834
qh-G(25°C) = -433.149563
qh-G(80°C) = -433.158450

Geometry:

C	3.710856	-2.035335	0.025738
C	3.745434	-1.181827	1.124031
C	3.005736	-0.003381	1.103464
C	2.245034	0.275355	-0.038650
C	2.923901	-1.661305	-1.060278
H	4.275621	-2.959767	0.004967
H	4.347681	-1.423035	1.993921
H	3.038438	0.671163	1.948274
H	2.864713	-2.291723	-1.943644

N 1.401155 1.421756 -0.129540
C 1.441525 2.096965 -1.438267
H 0.655107 2.853514 -1.460839
H 2.413207 2.581625 -1.587911
H 1.272441 1.369281 -2.228748
C 1.495420 2.374939 0.978257
H 0.769522 3.170522 0.805028
H 1.248458 1.883666 1.921058
H 2.496827 2.816636 1.045612
C -0.538788 0.621570 -0.018809
I -2.928936 -0.371046 0.076975
H -0.940582 1.618535 -0.125448
H -0.302836 0.029696 -0.890509
H -0.318842 0.235932 0.965914
N 2.204439 -0.537831 -1.096215

2Pyr_TMA_SN2_tsopt_GEN
Electronic energy = -728.688627
Thermal correction to Gibbs free energy (25°C) = 0.056587
Thermal correction to Gibbs free energy (80°C) = 0.072910
qh-G(25°C) = -728.430289
qh-G(80°C) = -728.440553

Geometry:
C -4.354141 -1.542337 -0.450653
C -3.905746 -0.526730 -1.291348
C -2.396473 0.224516 0.255987
C -2.768107 -0.756560 1.178370
C -3.767880 -1.651817 0.805588
H -5.135933 -2.218953 -0.774613
H -4.331206 -0.398456 -2.282750
H -2.314913 -0.829212 2.157561
H -4.083469 -2.423622 1.499671
N -1.325826 1.157015 0.513997
C 0.123923 0.580674 -0.578244
H 0.759777 1.388560 -0.236996
H 0.177483 -0.384451 -0.089612
H -0.447386 0.687606 -1.490235
C -0.817211 1.148094 1.894431
H -0.020696 1.889623 1.965362
H -1.607592 1.400950 2.607648
H -0.399306 0.168809 2.132999
C -1.646973 2.535260 0.082414
H -1.938675 2.526196 -0.965017
H -2.461425 2.939991 0.690725
H -0.753959 3.148169 0.213736
C 2.662151 -0.215759 -0.914379
C 2.838568 -1.451072 -0.243421

C 3.627409 0.792333 -0.672794
C 3.926489 -1.666791 0.600014
H 2.106622 -2.237206 -0.413861
C 4.713112 0.566634 0.170555
H 3.504673 1.749276 -1.174213
C 4.875232 -0.663165 0.815082
H 4.036387 -2.628954 1.094027
H 5.440586 1.359025 0.327979
H 5.722835 -0.835825 1.470489
O 1.623993 0.000402 -1.698016
N -2.946726 0.336639 -0.950908

2Pyr_TMA_SnAr_tsopt_GEN
Electronic energy = -728.702588
Thermal correction to Gibbs free energy (25°C) = 0.053086
Thermal correction to Gibbs free energy (80°C) = 0.068841
qh-G(25°C) = -728.440277
qh-G(80°C) = -728.450048

Geometry:
C 0.671778 2.916478 0.280762
C 0.361161 2.037931 1.334121
C 0.586946 0.686425 1.208931
C 1.090647 0.193896 -0.041094
C 1.235288 2.352099 -0.852682
H 0.511729 3.984897 0.354843
H -0.056707 2.418930 2.262080
H 0.320758 0.006839 2.009588
H 1.536287 2.982223 -1.687816
N 2.103302 -0.995228 0.068688
C 3.401818 -0.405677 0.526602
H 4.112391 -1.218855 0.676985
H 3.756393 0.280223 -0.240857
H 3.232221 0.129900 1.460551
C 2.333976 -1.655454 -1.257360
H 3.211124 -2.295185 -1.156465
H 1.452471 -2.234900 -1.512223
H 2.507768 -0.875204 -1.994637
C 1.675093 -2.027986 1.059950
H 1.724012 -1.608177 2.063244
H 0.663719 -2.339517 0.811113
H 2.368995 -2.865906 0.987555
O -0.174529 -0.897138 -0.751656
C -1.440165 -0.617509 -0.447275
C -1.993129 0.666084 -0.632441
C -2.277454 -1.628668 0.066554
C -3.328957 0.916160 -0.326384
H -1.357870 1.459696 -1.016683

C -3.616252 -1.373248 0.357612
H -1.857368 -2.619863 0.216753
C -4.153904 -0.098373 0.166281
H -3.731663 1.914042 -0.479014
H -4.242242 -2.174705 0.740737
H -5.195390 0.101354 0.397058
N 1.490774 1.046422 -1.003122

2Pyr_TMA_bromide_tsopt
Electronic energy = -2993.458009
Thermal correction to Gibbs free energy (25°C) = 0.048101
Thermal correction to Gibbs free energy (80°C) = 0.061112
qh-G(25°C) = -2993.290091
qh-G(80°C) = -2993.298867

Geometry:
C -3.324702 -1.875862 -0.030633
C -3.293438 -1.016163 -1.124199
C -2.461683 0.099537 -1.099139
C -1.679274 0.309403 0.042594
C -2.507639 -1.572647 1.055165
H -3.960800 -2.752780 -0.013246
H -3.914806 -1.203002 -1.993917
H -2.442152 0.779471 -1.940095
H -2.495545 -2.211180 1.934395
N -0.743507 1.384599 0.138703
C -0.727716 2.050333 1.453632
H 0.115677 2.742456 1.478080
H -1.657711 2.608295 1.610430
H -0.612833 1.305613 2.237829
C -0.773941 2.356009 -0.958252
H 0.009375 3.094186 -0.780827
H -0.569638 1.859576 -1.908410
H -1.740389 2.870381 -1.012219
C 1.105210 0.451714 0.004916
H 1.580972 1.416245 0.100852
H 0.845903 -0.120102 0.883212
H 0.841677 0.079130 -0.973757
N -1.700526 -0.510549 1.094838
Br 3.231074 -0.611445 -0.123475

2Pyr_TMA_chloride_tsopt
Electronic energy = -882.136735
Thermal correction to Gibbs free energy (25°C) = 0.046846
Thermal correction to Gibbs free energy (80°C) = 0.059627
qh-G(25°C) = -881.967245
qh-G(80°C) = -881.975812

Geometry:
C -2.983388 -1.505054 -0.044885
C -2.827988 -0.619201 -1.106331
C -1.809620 0.328876 -1.067858
C -0.973179 0.348040 0.054050
C -2.096777 -1.398309 1.023349
H -3.765459 -2.254902 -0.038230
H -3.495518 -0.655537 -1.960995
H -1.691804 1.029406 -1.883416
H -2.175718 -2.065733 1.877278
N 0.141911 1.237618 0.159582
C 0.295212 1.843929 1.494964
H 1.244832 2.380950 1.521436
H -0.522694 2.546157 1.690901
H 0.294505 1.062833 2.251680
C 0.253278 2.242657 -0.902002
H 1.151926 2.832968 -0.718295
H 0.353416 1.755787 -1.873610
H -0.614356 2.912164 -0.912734
C 1.782838 0.015006 -0.060097
H 2.422275 0.880031 0.034519
H 1.461108 -0.526705 0.816716
H 1.424257 -0.282803 -1.034358
N -1.112268 -0.498749 1.075296
Cl 3.580205 -1.313081 -0.280128

2pyr_TMA_gen
Electronic energy = -421.834793
Thermal correction to Gibbs free energy (25°C) = 0.041111
Thermal correction to Gibbs free energy (80°C) = 0.052422
qh-G(25°C) = -421.659281
qh-G(80°C) = -421.666865

Geometry:
C 2.031365 1.223916 -0.002478
C 2.819705 0.078874 -0.000259
C 2.186259 -1.161761 -0.001745
C 0.796486 -1.217506 -0.005086
C 0.121111 -0.001174 -0.008088
N 0.692675 1.181836 -0.006210
H 2.762523 -2.080146 0.000640
H 2.477039 2.213361 -0.000308
H 3.900121 0.159256 0.003467
H 0.285869 -2.172699 -0.003175
N -1.379641 0.004983 -0.000312
C -1.927069 1.403779 -0.048961
H -3.012567 1.318401 -0.050076

H -1.580920 1.950063 0.824607
H -1.574087 1.890287 -0.955078
C -1.897229 -0.745961 -1.197388
H -1.577300 -1.784106 -1.140651
H -2.985191 -0.693026 -1.182378
H -1.501543 -0.272798 -2.095089
C -1.869192 -0.652343 1.262043
H -1.461747 -0.104757 2.111084
H -2.957783 -0.611084 1.263767
H -1.534268 -1.687440 1.280613

2pyr_aniline_gen
Electronic energy = -382.112180
Thermal correction to Gibbs free energy (25°C) = 0.039469
Thermal correction to Gibbs free energy (80°C) = 0.049886
qh-G(25°C) = -381.979014
qh-G(80°C) = -381.986250

Geometry:
C -1.800323 -1.206383 0.009883
C -2.600539 -0.072288 0.039424
C -1.950909 1.166208 0.024939
C -0.569501 1.222210 -0.026751
C 0.158784 0.005913 -0.073945
N -0.463831 -1.185819 -0.038963
H -2.522613 2.088844 0.059585
H -2.251975 -2.196082 0.032138
H -3.679977 -0.153815 0.082104
H -0.065308 2.179507 -0.029473
N 1.529920 -0.006631 -0.168467
C 2.261058 1.228267 0.057256
H 2.091690 1.635362 1.064460
H 3.325917 1.030293 -0.061645
H 1.979520 1.988146 -0.676904
C 2.234022 -1.257700 0.069889
H 1.801155 -2.053230 -0.535354
H 3.279177 -1.125237 -0.211393
H 2.184237 -1.563999 1.124325

2pyr_bromide_ionpair_gen
Electronic energy = -2993.505667
Thermal correction to Gibbs free energy (25°C) = 0.047586
Thermal correction to Gibbs free energy (80°C) = 0.060398
qh-G(25°C) = -2993.333375
qh-G(80°C) = -2993.341978

Geometry:

Br -3.240178 -0.473576 0.000115
N 0.770789 1.384414 -0.000102
C 1.623497 0.153171 -0.000244
C 3.009533 0.215499 0.000327
H 3.561299 1.144976 0.000595
C 3.689939 -1.003811 0.000445
H 4.774258 -1.011119 0.000846
C 2.970091 -2.191283 0.000009
H 3.467387 -3.153847 0.000062
C 1.577535 -2.119219 -0.000478
H 0.968727 -3.018022 -0.000923
C -0.105661 1.366319 1.225997
H -0.695957 2.282598 1.221681
H 0.538226 1.326410 2.104576
H -0.758216 0.496677 1.164183
C -0.106293 1.366531 -1.225742
H -0.696277 2.283006 -1.221075
H -0.759087 0.497096 -1.163627
H 0.537095 1.326513 -2.104675
C 1.575251 2.645335 -0.000306
H 0.874495 3.478421 -0.000124
H 2.189776 2.682107 -0.898728
H 2.190184 2.682129 0.897826
N 0.916491 -0.961135 -0.000568

2pyr_chloride_ionpair_gen
Electronic energy = -882.183911
Thermal correction to Gibbs free energy (25°C) = 0.046844
Thermal correction to Gibbs free energy (80°C) = 0.059537
qh-G(25°C) = -882.010927
qh-G(80°C) = -882.019406

Geometry:
Cl -3.650226 -1.127703 0.000119
N -0.089420 1.286820 -0.000043
C 0.948170 0.206461 -0.000073
C 2.306993 0.487624 0.000167
H 2.704584 1.492594 0.000323
C 3.172327 -0.608220 0.000194
H 4.244040 -0.443454 0.000376
C 2.649974 -1.894919 0.000028
H 3.293628 -2.766414 0.000096
C 1.263682 -2.044453 -0.000227
H 0.805329 -3.028501 -0.000294
C -0.953259 1.130082 1.225372
H -1.675082 1.946791 1.225656
H -0.311081 1.184412 2.104222
H -1.466847 0.171952 1.157984

C -0.953520 1.130011 -1.225283
H -1.675111 1.946927 -1.225632
H -1.467320 0.172022 -1.157646
H -0.311467 1.183991 -2.104243
C 0.507006 2.658526 -0.000172
H -0.315638 3.371532 -0.000368
H 1.107954 2.790735 -0.899131
H 1.107683 2.791049 0.898914
N 0.427267 -1.005871 -0.000289

3Br_Ionpair_I_axial
Electronic energy = -2988.204392
Thermal correction to Gibbs free energy (25°C) = 0.053096
Thermal correction to Gibbs free energy (80°C) = 0.067353
qh-G(25°C) = -2988.034381
qh-G(80°C) = -2988.043983

Geometry:
C -3.840439 1.509487 -0.000228
C -3.454777 0.176674 -0.000229
C -2.113652 -0.203282 -0.000114
C -1.147021 0.794962 -0.000011
C -1.504232 2.142769 -0.000023
C -2.850180 2.489997 -0.000131
H -4.889922 1.780264 -0.000333
H -1.866025 -1.255435 -0.000140
H -0.757388 2.928391 0.000035
H -3.130710 3.537301 -0.000155
N 0.304107 0.452140 0.000059
C 0.964014 1.022473 -1.230501
H 2.019127 0.743348 -1.193291
H 0.472500 0.595650 -2.104484
H 0.857609 2.105087 -1.225566
C 0.561566 -1.026981 -0.000066
H 1.645014 -1.158062 0.000019
H 0.130562 -1.464572 0.899359
H 0.130720 -1.464399 -0.899651
C 0.963843 1.022228 1.230826
H 2.018959 0.743104 1.193706
H 0.857442 2.104840 1.226096
H 0.472203 0.595228 2.104651
Br -4.778669 -1.176981 -0.000463
I 4.560186 -0.364742 0.000348

3Br_SnArProduct_GEN
Electronic energy = -3071.059981
Thermal correction to Gibbs free energy (25°C) = 0.046397

Thermal correction to Gibbs free energy (80°C) = 0.059034
qh-G(25°C) = -3070.925216
qh-G(80°C) = -3070.933660

Geometry:

C	-3.440952	-0.148513	1.009475
C	-2.549494	0.354238	0.138759
C	-2.543474	-0.414205	-1.116191
C	-3.449962	-1.401060	-0.995708
C	-4.106707	-1.324539	0.355974
H	-3.963437	-2.249278	0.930039
H	-3.655237	0.225040	2.001928
H	-1.912419	-0.194708	-1.969639
H	-3.697880	-2.143816	-1.743861
H	-5.191884	-1.186510	0.260870
O	-1.802617	1.491601	0.335027
C	-0.448745	1.421289	0.134766
C	0.211330	2.628994	-0.097521
C	1.590533	2.628545	-0.269969
H	-0.365859	3.546027	-0.140156
C	1.633399	0.255437	0.017914
C	2.320582	1.439046	-0.219399
H	2.108657	3.564055	-0.452576
H	3.394821	1.436375	-0.360119
C	0.254351	0.217921	0.202695
H	-0.256790	-0.717205	0.400694
Br	2.603880	-1.375877	0.097938

3Br_TMA
Electronic energy = -2976.672696
Thermal correction to Gibbs free energy (25°C) = 0.045054
Thermal correction to Gibbs free energy (80°C) = 0.057537
qh-G(25°C) = -2976.497204
qh-G(80°C) = -2976.505540

Geometry:

C	-1.203783	0.282387	-0.000024
C	0.010201	-0.393822	-0.000009
C	1.184162	0.357647	-0.000003
C	1.167279	1.745081	-0.000004
C	-0.064230	2.397185	-0.000003
C	-1.252567	1.675735	-0.000014
H	0.079676	-1.472122	-0.000011
H	2.093339	2.307821	0.000001
H	-0.098755	3.480649	0.000007
H	-2.193090	2.213877	0.000002
Br	2.841085	-0.555348	0.000002
N	-2.495564	-0.469586	-0.000001

C -3.287996 -0.110863 1.231922
H -3.511631 0.953202 1.219475
H -4.212837 -0.686485 1.216551
H -2.690087 -0.363329 2.106462
C -3.287879 -0.111190 -1.232095
H -4.212726 -0.686799 -1.216654
H -3.511523 0.952876 -1.219966
H -2.689882 -0.363900 -2.106505
C -2.300761 -1.958295 0.000203
H -1.759781 -2.249770 -0.898493
H -1.760040 -2.249561 0.899123
H -3.288244 -2.415373 0.000115

3Br_TMA_DMSO_tsopt_GEN
Electronic energy = -3529.710027
Thermal correction to Gibbs free energy (25°C) = 0.060997
Thermal correction to Gibbs free energy (80°C) = 0.078460
qh-G(25°C) = -3529.464595
qh-G(80°C) = -3529.475707

Geometry:
C -2.607958 -0.154742 1.736039
C -2.630224 -0.144927 0.348957
C -1.652020 0.494936 -0.407502
C -0.606239 1.154697 0.247277
C -0.568365 1.153109 1.649754
C -1.560459 0.505810 2.376707
H -3.382368 -0.659051 2.301622
H -1.730617 0.478049 -1.485751
H 0.228532 1.651922 2.188146
H -1.516836 0.514237 3.460455
N 0.458288 1.772370 -0.490206
C 0.859682 3.096964 0.023362
H 1.677879 3.471993 -0.592826
H 0.019448 3.797179 -0.024749
H 1.212738 3.024993 1.050657
C 0.270449 1.817592 -1.947151
H 1.150533 2.284220 -2.390906
H 0.177464 0.807190 -2.348598
H -0.615268 2.403327 -2.216718
C 2.099420 0.532467 -0.227762
H 2.018775 0.686732 0.841939
H 1.529627 -0.256825 -0.704044
H 2.684417 1.221872 -0.825803
S 3.880690 -0.758300 0.059494
O 5.000599 -0.099274 0.804477
C 4.464235 -1.330070 -1.533667
H 4.757116 -0.447897 -2.104018

H 5.321713 -1.983500 -1.363722
H 3.654109 -1.862390 -2.035099
C 3.431988 -2.290734 0.871788
H 4.329787 -2.907214 0.943161
H 3.060599 -2.030428 1.863553
H 2.655855 -2.788125 0.287608
Br -4.044012 -1.025615 -0.560800

3Br_TMA_I_TS
Electronic energy = -2988.154345
Thermal correction to Gibbs free energy (25°C) = 0.053161
Thermal correction to Gibbs free energy (80°C) = 0.067557
qh-G(25°C) = -2987.988374
qh-G(80°C) = -2987.998069

Geometry:
C 2.864913 -0.268004 -1.742366
C 2.911941 -0.203675 -0.357449
C 1.970711 0.499415 0.389254
C 0.934706 1.169394 -0.272792
C 0.872960 1.112718 -1.673778
C 1.828308 0.403031 -2.390704
H 3.611453 -0.821293 -2.299681
H 2.067344 0.521999 1.466032
H 0.080353 1.613193 -2.216788
H 1.763758 0.368222 -3.473069
N -0.090928 1.850672 0.454991
C -0.468263 3.160439 -0.106702
H -1.267120 3.580291 0.506101
H 0.388535 3.843215 -0.103887
H -0.842275 3.053330 -1.123523
C 0.117970 1.944340 1.904535
H -0.742467 2.452364 2.341261
H 0.185392 0.947073 2.342406
H 1.024581 2.512882 2.142998
C -1.798721 0.644979 0.260490
Br 4.312540 -1.097190 0.560222
H -2.358519 1.356562 0.849569
H -1.741087 0.756385 -0.811697
H -1.264092 -0.157687 0.745942
I -3.898404 -0.855763 0.014692

3Br_TMA_SN2_tsopt_GEN
Electronic energy = -3283.526699
Thermal correction to Gibbs free energy (25°C) = 0.060329
Thermal correction to Gibbs free energy (80°C) = 0.077798
qh-G(25°C) = -3283.268656

qh-G(80°C) = -3283.279619

Geometry:

C	3.016723	1.781485	0.384437
C	3.226924	0.451713	0.050295
C	2.240465	-0.518961	0.206638
C	0.996034	-0.144954	0.718871
C	0.764397	1.194300	1.059206
C	1.768964	2.140336	0.892781
H	3.800952	2.517747	0.253982
H	2.464744	-1.541589	-0.063351
H	-0.193861	1.513638	1.451372
H	1.576888	3.173915	1.159493
N	-0.080508	-1.101948	0.848297
C	-1.413187	-0.625177	-0.423063
H	-2.034262	-1.472098	-0.158670
H	-1.594132	0.336985	0.037528
H	-0.729472	-0.696113	-1.258424
C	-0.731361	-1.062663	2.177467
H	-1.554049	-1.779327	2.176267
H	-0.012878	-1.327923	2.958086
H	-1.137382	-0.071925	2.374074
C	0.287867	-2.489338	0.515425
H	0.618954	-2.548089	-0.522331
H	1.072759	-2.859473	1.181652
H	-0.600480	-3.110593	0.632149
C	-3.908999	0.094950	-1.084333
C	-4.198872	1.384522	-0.574833
C	-4.855522	-0.927235	-0.829219
C	-5.376314	1.636654	0.126226
H	-3.481859	2.181581	-0.757389
C	-6.030679	-0.665367	-0.128038
H	-4.646731	-1.925046	-1.207647
C	-6.304562	0.617206	0.355650
H	-5.572558	2.639583	0.497010
H	-6.740810	-1.470432	0.043436
H	-7.221700	0.817878	0.900034
O	-2.787865	-0.152023	-1.733806
Br	4.913174	-0.075821	-0.641428

3Br_TMA_SnAr_tsopt_GEN
Electronic energy = -3283.536561
Thermal correction to Gibbs free energy (25°C) = 0.057714
Thermal correction to Gibbs free energy (80°C) = 0.074740
qh-G(25°C) = -3283.275855
qh-G(80°C) = -3283.286404

Geometry:

C	0.908648	-0.962270	1.862234
C	-0.204654	-0.298418	2.395746
C	-1.300167	0.055652	1.630751
C	-1.321878	-0.166208	0.212003
C	-0.236068	-0.945698	-0.306975
C	0.838518	-1.255651	0.505682
H	1.765058	-1.233587	2.465136
H	-0.215821	-0.052693	3.454538
H	-2.114610	0.593821	2.101416
N	-2.726515	-0.574157	-0.359821
C	-3.095337	-1.891667	0.248563
H	-4.037296	-2.218340	-0.192986
H	-2.302195	-2.607227	0.035727
H	-3.199654	-1.764275	1.325082
C	-2.699486	-0.737341	-1.849010
H	-3.722222	-0.912406	-2.183055
H	-2.296241	0.172552	-2.285526
H	-2.088651	-1.600768	-2.104227
C	-3.801040	0.414795	-0.021018
H	-3.840906	0.550093	1.057263
H	-3.579258	1.354042	-0.516872
H	-4.748825	0.001314	-0.366559
O	-1.350852	1.374249	-0.599161
C	-0.190574	2.028663	-0.454291
C	0.870594	1.822201	-1.354874
C	-0.001400	2.921535	0.615924
C	2.082704	2.489192	-1.186232
H	0.722125	1.137395	-2.184751
C	1.211586	3.588645	0.776032
C	2.262084	3.373142	-0.119529
H	2.891665	2.318647	-1.891124
H	1.339620	4.278257	1.605702
H	3.206445	3.892765	0.008346
H	-0.822610	3.079454	1.308734
H	-0.190615	-1.186289	-1.360888
Br	2.319579	-2.154202	-0.305542

3Br_TMA_bromide_tsopt
Electronic energy = -5548.295657
Thermal correction to Gibbs free energy (25°C) = 0.052429
Thermal correction to Gibbs free energy (80°C) = 0.066683
qh-G(25°C) = -5548.128618
qh-G(80°C) = -5548.138188

Geometry:

C	-2.367436	-0.323336	1.730335
C	-2.426627	-0.158979	0.354428
C	-1.425192	0.490196	-0.363053

C	-0.315263	0.997727	0.321083
C	-0.238419	0.836155	1.712907
C	-1.255281	0.185660	2.400835
H	-3.160782	-0.831897	2.265036
H	-1.533343	0.597093	-1.433664
H	0.613038	1.208099	2.270440
H	-1.179718	0.068244	3.476514
N	0.768906	1.622050	-0.378721
C	1.219292	2.893074	0.219510
H	2.094269	3.242236	-0.331039
H	0.427095	3.647166	0.157568
H	1.503643	2.753659	1.260786
C	0.574947	1.779093	-1.825418
H	1.477193	2.226824	-2.243860
H	0.424279	0.804949	-2.293502
H	-0.277780	2.431243	-2.046467
C	2.374325	0.314115	-0.230331
Br	-3.926147	-0.834838	-0.594429
H	2.954227	0.979442	-0.851770
H	2.374304	0.438320	0.841383
H	1.765942	-0.456123	-0.679654
Br	4.220239	-1.180757	-0.081642

3Br_TMA_chloride_tsopt
Electronic energy = -3436.974415
Thermal correction to Gibbs free energy (25°C) = 0.051421
Thermal correction to Gibbs free energy (80°C) = 0.065493
qh-G(25°C) = -3436.806126
qh-G(80°C) = -3436.815531

Geometry:

C	-1.784215	-0.580724	1.653415
C	-1.835827	-0.149298	0.336270
C	-0.759804	0.479579	-0.284932
C	0.418841	0.687873	0.439023
C	0.488294	0.254798	1.771562
C	-0.602969	-0.368612	2.364131
H	-2.635895	-1.067426	2.113576
H	-0.863160	0.803434	-1.311402
H	1.391029	0.392068	2.354932
H	-0.532437	-0.699251	3.394799
N	1.575105	1.277523	-0.172996
C	2.199056	2.345893	0.631461
H	3.107364	2.673570	0.123151
H	1.515395	3.194982	0.736928
H	2.472476	1.978050	1.618769
C	1.388780	1.726294	-1.558876
H	2.339100	2.122244	-1.918827

H 1.102597 0.884632 -2.191881
H 0.630337 2.514402 -1.626115
C 2.974914 -0.224825 -0.313298
Br -3.428323 -0.415475 -0.662642
H 3.624209 0.449068 -0.851589
H 3.023144 -0.276686 0.763376
H 2.253864 -0.826043 -0.846277
Cl 4.506772 -1.857055 -0.481678

3Br_aniline_gen
Electronic energy = -2936.950467
Thermal correction to Gibbs free energy (25°C) = 0.043926
Thermal correction to Gibbs free energy (80°C) = 0.055596
qh-G(25°C) = -2936.818418
qh-G(80°C) = -2936.826453

Geometry:
C 1.511163 0.158590 -0.067162
C 0.231394 -0.443119 -0.046516
C -0.901713 0.356865 -0.011356
C -0.851852 1.744878 0.017068
C 0.414001 2.333148 0.012277
C 1.575009 1.571371 -0.023422
H 0.118482 -1.518678 -0.058591
H -1.755736 2.340667 0.047121
H 0.495712 3.415380 0.042078
H 2.532146 2.076795 -0.018576
Br -2.604729 -0.499165 0.009893
N 2.650005 -0.609879 -0.134539
C 3.940806 0.022075 0.076625
H 4.720158 -0.734716 -0.001599
H 4.133890 0.780835 -0.687835
H 4.013040 0.498445 1.064578
C 2.544463 -2.047741 0.038677
H 2.114516 -2.318138 1.013713
H 1.924918 -2.491043 -0.747202
H 3.538719 -2.486020 -0.035329

3Br_bromide_ionpair1_gen
Electronic energy = -5548.348337
Thermal correction to Gibbs free energy (25°C) = 0.051103
Thermal correction to Gibbs free energy (80°C) = 0.065024
qh-G(25°C) = -5548.176039
qh-G(80°C) = -5548.185319

Geometry:
Br 1.161906 2.852765 -0.000405

N 2.108452 -1.366688 0.000382
C 0.646325 -1.674159 -0.000108
C 0.178450 -2.982675 -0.000702
C -1.200166 -3.204651 -0.001051
C -2.097229 -2.143878 -0.000738
C -1.592786 -0.846344 -0.000161
C 2.454214 -0.563197 1.229453
H 3.531588 -0.397462 1.225802
H 2.155793 -1.138195 2.105311
H 1.933602 0.393449 1.184925
C 2.454915 -0.562981 -1.228346
H 3.532289 -0.397209 -1.224015
H 1.934196 0.393623 -1.183913
H 2.157045 -1.137844 -2.104482
C 2.954442 -2.603398 0.000544
H 3.995333 -2.285826 0.001121
H 2.745221 -3.181211 -0.898226
H 2.744324 -3.181559 0.898885
C -0.229417 -0.589125 0.000057
H 0.124519 0.439824 0.000274
H 0.842240 -3.835997 -0.000834
H -3.166703 -2.319222 -0.000930
H -1.572018 -4.223017 -0.001525
Br -2.794166 0.617518 0.000441

3Br_bromide_ionpair2_gen
Electronic energy = -5548.347490
Thermal correction to Gibbs free energy (25°C) = 0.051312
Thermal correction to Gibbs free energy (80°C) = 0.065270
qh-G(25°C) = -5548.175351
qh-G(80°C) = -5548.184670

Geometry:
Br -4.213401 -0.882195 -0.000340
N -0.751267 1.703816 0.000183
C 0.139601 0.504975 0.000294
C 1.522454 0.645229 -0.000060
C 2.300008 -0.511570 0.000100
C 1.732605 -1.777989 0.000554
C 0.342879 -1.885126 0.000940
C -1.625820 1.676552 1.229335
H -2.227985 2.585057 1.227473
H -0.978816 1.644191 2.105256
H -2.275694 0.802759 1.181283
C -1.625967 1.676157 -1.228851
H -2.228280 2.584561 -1.227080
H -2.275683 0.802263 -1.180503
H -0.979068 1.643700 -2.104844

C 0.008509 2.995845 -0.000058
H -0.723292 3.801552 -0.000037
H 0.620273 3.054929 -0.898967
H 0.620532 3.055090 0.898665
C -0.463021 -0.751774 0.000801
H -1.544744 -0.863674 0.001138
H 2.015090 1.607142 -0.000407
H 2.359147 -2.662298 0.000632
H -0.119608 -2.865960 0.001331
Br 4.185101 -0.333514 -0.000333

3Br_chloride_ionpair1_gen
Electronic energy = -3437.026331
Thermal correction to Gibbs free energy (25°C) = 0.050512
Thermal correction to Gibbs free energy (80°C) = 0.064352
qh-G(25°C) = -3436.853532
qh-G(80°C) = -3436.862729

Geometry:
Cl 0.266731 3.377437 -0.000813
N 2.454201 -0.156640 0.000354
C 1.167312 -0.915755 0.000072
C 1.145173 -2.305081 -0.000604
C -0.088411 -2.959003 -0.000983
C -1.278797 -2.242871 -0.000657
C -1.218436 -0.852000 -0.000014
C 2.522737 0.717074 1.228587
H 3.493858 1.212092 1.229423
H 2.414540 0.079461 2.105130
H 1.727809 1.461362 1.176035
C 2.523355 0.716530 -1.228242
H 3.494392 1.211703 -1.228761
H 1.728190 1.460647 -1.176452
H 2.415732 0.078511 -2.104580
C 3.653644 -1.055679 0.000945
H 4.535974 -0.418512 0.001257
H 3.643769 -1.671094 -0.897476
H 3.642936 -1.670964 0.899458
C -0.010457 -0.168972 0.000316
H -0.002683 0.920044 0.000737
H 2.048616 -2.898709 -0.000857
H -2.235128 -2.752718 -0.000914
H -0.112804 -4.042878 -0.001511
Br -2.828736 0.145876 0.000381

3Br_chloride_ionpair2_gen
Electronic energy = -3437.025594

Thermal correction to Gibbs free energy (25°C) = 0.050526
Thermal correction to Gibbs free energy (80°C) = 0.064373
qh-G(25°C) = -3436.852783
qh-G(80°C) = -3436.861986

Geometry:

Cl	-4.556970	-1.462904	0.000443
N	-1.541354	1.402170	-0.000124
C	-0.519392	0.312739	-0.000254
C	0.838839	0.609047	-0.000011
C	1.741767	-0.452911	-0.000055
C	1.320831	-1.775198	-0.000342
C	-0.047740	-2.039162	-0.000562
C	-2.407116	1.274043	1.229200
H	-3.103737	2.112394	1.232898
H	-1.759318	1.307989	2.104344
H	-2.958191	0.334925	1.175251
C	-2.407530	1.274025	-1.229143
H	-3.104074	2.112440	-1.232684
H	-2.958704	0.334982	-1.174942
H	-1.760027	1.307832	-2.104511
C	-0.933925	2.772211	-0.000246
H	-1.753109	3.488998	-0.000219
H	-0.333589	2.900838	-0.899853
H	-0.333490	2.900967	0.899273
C	-0.976600	-1.004035	-0.000526
H	-2.039910	-1.234804	-0.000733
H	1.219766	1.620574	0.000205
H	2.043494	-2.582809	-0.000402
H	-0.396154	-3.066069	-0.000792
Br	3.595435	-0.065961	0.000203

3Br_dimer_anti_CONF1
Electronic energy = -5976.426360
Thermal correction to Gibbs free energy (25°C) = 0.084895
Thermal correction to Gibbs free energy (80°C) = 0.109557
qh-G(25°C) = -5976.063094
qh-G(80°C) = -5976.078119

Geometry:

C	-4.032101	-0.174886	-0.137324
C	-5.196659	-0.927495	-0.237937
C	-6.410415	-0.249501	-0.339548
C	-6.480977	1.136549	-0.342187
C	-5.296247	1.863428	-0.239709
C	-4.069055	1.217950	-0.137255
H	-5.197797	-2.008247	-0.240762
H	-7.437680	1.639307	-0.422157

H -5.327884 2.947571 -0.239512
N -2.699863 -0.838108 -0.024324
C -2.032971 -0.428398 1.264954
H -1.087091 -0.971578 1.326212
H -2.691054 -0.697507 2.090552
H -1.853129 0.647301 1.244272
C -1.820918 -0.418281 -1.176360
H -0.876095 -0.957422 -1.076023
H -1.650660 0.657450 -1.117599
H -2.326565 -0.682917 -2.104640
C -2.790769 -2.335623 -0.037685
H -1.772518 -2.717674 0.045569
H -3.235786 -2.658032 -0.978037
H -3.384589 -2.665309 0.813995
H -3.162833 1.812267 -0.057507
N 2.768102 1.120368 0.602467
C 3.997931 0.280477 0.689495
C 4.137653 -0.698665 1.664933
H 3.362241 -0.902067 2.389603
C 5.307315 -1.459289 1.697496
H 5.417468 -2.227339 2.454775
C 6.324297 -1.252949 0.773854
H 7.230436 -1.846690 0.800493
C 6.154355 -0.266106 -0.193050
C 5.003243 0.507504 -0.250395
H 4.910775 1.263071 -1.021346
C 2.086322 0.887226 -0.723282
H 1.185089 1.503778 -0.739253
H 1.843417 -0.175049 -0.787058
H 2.758335 1.181575 -1.527125
C 3.125803 2.577675 0.733467
H 2.195861 3.147566 0.688916
H 3.781204 2.864816 -0.085873
H 3.628309 2.719753 1.689853
C 1.773194 0.800406 1.682035
H 0.919908 1.463503 1.529478
H 2.228888 0.990863 2.653138
H 1.470714 -0.242876 1.576942
I -0.859043 3.513911 0.214248
I 1.176504 -2.936982 0.294040
Br -8.004365 -1.262666 -0.475999
Br 7.521163 0.034114 -1.468063

3Br_dimer_anti_CONF2
Electronic energy = -5976.427455
Thermal correction to Gibbs free energy (25°C) = 0.084607
Thermal correction to Gibbs free energy (80°C) = 0.109203
qh-G(25°C) = -5976.063819

qh-G(80°C) = -5976.078782

Geometry:

C	-3.816937	-0.388018	0.010112
C	-4.873511	-1.289708	-0.051481
C	-6.171119	-0.787669	0.033782
C	-6.427667	0.568758	0.176667
C	-5.347688	1.447583	0.235920
C	-4.041055	0.979624	0.153133
H	-4.730628	-2.355380	-0.161662
H	-7.446377	0.932964	0.240212
H	-5.524684	2.511828	0.347297
N	-2.402869	-0.858129	-0.076929
C	-1.657006	-0.467920	1.174969
H	-0.646110	-0.872831	1.089434
H	-2.178398	-0.898155	2.029371
H	-1.625569	0.620182	1.244186
C	-1.723014	-0.218190	-1.261481
H	-0.708706	-0.621393	-1.308040
H	-1.696831	0.862189	-1.113007
H	-2.285723	-0.473555	-2.158969
C	-2.291744	-2.346328	-0.231693
H	-1.228587	-2.582873	-0.290576
H	-2.794086	-2.649241	-1.149593
H	-2.734114	-2.829478	0.638663
H	-3.220171	1.689968	0.202118
N	2.724380	1.867519	-0.230223
C	4.038998	1.207064	-0.475263
C	4.473275	0.172360	0.343540
H	3.894926	-0.189715	1.181060
C	5.687247	-0.440769	0.043209
C	6.468585	-0.039952	-1.031070
H	7.409873	-0.533385	-1.243110
C	6.011665	1.003952	-1.833694
C	4.798880	1.629178	-1.565378
H	4.463863	2.428399	-2.215999
C	1.802351	1.571112	-1.386753
H	0.845887	2.054290	-1.175395
H	1.697867	0.486419	-1.453446
H	2.238766	1.973365	-2.299208
C	2.901133	3.357199	-0.096395
H	1.917318	3.784889	0.104985
H	3.300485	3.761986	-1.023418
H	3.587524	3.544608	0.728684
C	2.052139	1.380192	1.022072
H	1.114557	1.931192	1.111459
H	2.697480	1.590223	1.874725
H	1.854703	0.311814	0.920277
I	-1.188107	3.704773	0.343285

I 1.739496 -2.403111 -0.356854
Br -7.619605 -2.004499 -0.050999
Br 6.259901 -1.878171 1.132949
H 6.604939 1.330512 -2.680225

3Br_dimer_anti_CONF3
Electronic energy = -5976.427225
Thermal correction to Gibbs free energy (25°C) = 0.084939
Thermal correction to Gibbs free energy (80°C) = 0.109609
qh-G(25°C) = -5976.064273
qh-G(80°C) = -5976.079292

Geometry:
C 3.994943 1.714896 -0.390032
C 4.928872 2.713543 -0.639136
C 6.271787 2.361337 -0.786243
C 6.680458 1.037488 -0.687251
C 5.719655 0.061773 -0.437028
C 4.376458 0.377466 -0.286030
H 4.653395 3.755268 -0.723122
H 7.723531 0.766521 -0.801538
N 2.545483 2.030852 -0.223953
C 2.076195 1.593877 1.141455
H 1.028335 1.888970 1.233245
H 2.690123 2.094627 1.889459
H 2.172298 0.510178 1.219801
C 1.738358 1.301269 -1.268549
H 0.697321 1.609519 -1.147345
H 1.837140 0.227081 -1.106138
H 2.117591 1.582373 -2.250569
C 2.255292 3.496974 -0.360582
H 1.180720 3.623613 -0.222955
H 2.547129 3.827985 -1.356286
H 2.799031 4.041944 0.409900
H 3.658848 -0.416081 -0.090879
N -2.275261 -1.147806 0.695849
C -3.676658 -0.637714 0.730505
C -4.070966 0.331654 1.644025
H -3.382843 0.761542 2.357797
C -5.392315 0.780577 1.626138
C -6.309881 0.276224 0.713244
H -7.334823 0.628109 0.701193
C -5.884416 -0.692264 -0.191734
C -4.577315 -1.159397 -0.198334
H -4.287091 -1.911557 -0.922243
C -1.646732 -0.807816 -0.632696
H -0.619191 -1.177378 -0.611756
H -1.677748 0.277592 -0.743937

H -2.207341 -1.295963 -1.427528
C -2.259994 -2.641286 0.890769
H -1.216443 -2.961040 0.879082
H -2.806772 -3.118811 0.080736
H -2.728137 -2.863517 1.849275
C -1.415141 -0.544312 1.769936
H -0.422971 -0.985805 1.663376
H -1.837061 -0.794190 2.742904
H -1.370291 0.534924 1.615313
I 1.839711 -2.571294 0.457112
I -1.712499 3.158779 0.211167
H 7.003738 3.137127 -0.980593
H -5.702105 1.540091 2.335147
Br -7.112892 -1.393518 -1.449895
Br 6.255699 -1.748964 -0.297108

3Br_dimer_anti_CONF4
Electronic energy = -5976.428296
Thermal correction to Gibbs free energy (25°C) = 0.084744
Thermal correction to Gibbs free energy (80°C) = 0.109379
qh-G(25°C) = -5976.064963
qh-G(80°C) = -5976.079955

Geometry:
C 3.593939 2.066594 -0.080590
C 4.332289 3.243303 -0.125319
C 5.724388 3.171145 -0.051013
C 6.373282 1.948511 0.065696
C 5.604289 0.788923 0.107592
C 4.218468 0.825056 0.036635
H 3.866822 4.214568 -0.215761
H 7.454156 1.894663 0.123074
N 2.102985 2.083015 -0.154617
C 1.520044 1.473485 1.095730
H 0.433329 1.555746 1.020135
H 1.895200 2.031097 1.953453
H 1.817025 0.425334 1.151126
C 1.637888 1.282659 -1.345252
H 0.550201 1.373510 -1.391214
H 1.925437 0.239572 -1.206509
H 2.102171 1.699242 -2.238688
C 1.547778 3.470771 -0.289817
H 0.462144 3.377698 -0.336240
H 1.924788 3.918748 -1.208235
H 1.836047 4.056864 0.581624
H 3.656271 -0.105111 0.074749
N -2.069763 -1.996140 -0.230321
C -3.525267 -1.738857 -0.430172

C -4.205843 -0.853632 0.396145
H -3.725138 -0.328259 1.208543
C -5.553152 -0.613576 0.137156
C -6.225276 -1.237474 -0.904186
H -7.274144 -1.033404 -1.084789
C -5.519750 -2.124738 -1.715020
C -4.171532 -2.377995 -1.487302
H -3.646554 -3.062493 -2.143120
C -1.309510 -1.467363 -1.421492
H -0.248287 -1.652055 -1.241148
H -1.521807 -0.399293 -1.498248
H -1.641716 -1.993590 -2.314620
C -1.812749 -3.472620 -0.084020
H -0.742291 -3.602015 0.085205
H -2.110644 -3.986032 -0.995303
H -2.391199 -3.835461 0.764987
C -1.521104 -1.320201 0.994432
H -0.463493 -1.582237 1.051873
H -2.051345 -1.691090 1.871178
H -1.637234 -0.241387 0.879964
I 2.225702 -2.604900 0.201175
I -2.321849 2.371180 -0.419589
H 6.304316 4.086500 -0.085542
Br 6.468938 -0.888567 0.265342
Br -6.472804 0.621562 1.237657
H -6.024610 -2.620810 -2.536158

3Br_dimer_stacked_CONF1
Electronic energy = -5976.426139
Thermal correction to Gibbs free energy (25°C) = 0.084219
Thermal correction to Gibbs free energy (80°C) = 0.108802
qh-G(25°C) = -5976.062630
qh-G(80°C) = -5976.077536

Geometry:
C -1.837387 1.769415 -0.948915
C -1.835498 2.114518 0.396283
C -0.672362 2.514849 1.053522
C 0.521019 2.523610 0.336255
C 0.553457 2.160508 -1.009233
C -0.631108 1.798581 -1.644537
H -2.756624 1.464391 -1.438558
H -0.726874 2.773911 2.102034
H 1.480655 2.115701 -1.568643
H -0.605933 1.520098 -2.693229
N 1.810134 2.827057 1.025893
C 2.471112 1.515997 1.391247
H 3.427066 1.739278 1.866754

H 1.821204 0.982352 2.084235
H 2.628609 0.937900 0.477860
C 1.614062 3.633983 2.277027
H 2.601097 3.882406 2.662340
H 1.062786 4.541061 2.031111
H 1.084307 3.039288 3.017487
C 2.737287 3.600203 0.121857
H 3.601341 3.894834 0.715285
H 3.064942 2.957077 -0.694133
H 2.207531 4.476987 -0.248819
I 3.733493 0.370568 -2.348399
C -3.994709 -1.046082 -2.273498
C -3.990233 -1.267205 -0.898640
C -2.822192 -1.579135 -0.214365
C -1.627687 -1.654380 -0.930995
C -1.602490 -1.445158 -2.304618
C -2.793420 -1.141160 -2.966016
H -4.916882 -0.803488 -2.788599
H -2.867358 -1.752598 0.854082
H -0.687099 -1.498242 -2.877758
H -2.775464 -0.972357 -4.036843
N -0.379415 -1.961537 -0.174295
C 0.858969 -1.815346 -1.016205
H 1.713860 -1.990357 -0.360460
H 0.841801 -2.552346 -1.817775
H 0.907754 -0.800337 -1.412318
C -0.425379 -3.372167 0.349624
H 0.507483 -3.552094 0.887227
H -1.276579 -3.474515 1.020663
H -0.524626 -4.048394 -0.498682
C -0.231364 -1.010400 0.982619
H 0.712621 -1.243428 1.480788
H -0.219204 -0.001502 0.573846
H -1.063422 -1.130788 1.672908
I 3.438845 -2.059072 2.091887
Br -5.614157 -1.162409 0.063762
Br -3.447112 1.994508 1.376657

3Br_dimer_stacked_CONF2
Electronic energy = -5976.426460
Thermal correction to Gibbs free energy (25°C) = 0.084046
Thermal correction to Gibbs free energy (80°C) = 0.108613
qh-G(25°C) = -5976.062886
qh-G(80°C) = -5976.077765

Geometry:
C 2.092165 -1.656997 1.189651
C 2.019270 -0.973727 2.399372

C	0.790778	-0.731068	3.018985
C	-0.376770	-1.158691	2.393858
C	-0.332158	-1.841114	1.179059
C	0.907173	-2.091774	0.602053
H	3.048052	-1.849448	0.713103
H	0.780575	-0.205162	3.963690
H	-1.230862	-2.154033	0.655721
N	-1.722134	-0.861476	2.970305
C	-2.355213	0.259446	2.177087
H	-3.355257	0.433037	2.576602
H	-1.742845	1.152687	2.294663
H	-2.418431	-0.040856	1.128993
C	-1.644231	-0.442653	4.409076
H	-2.664034	-0.306341	4.764406
H	-1.145944	-1.225405	4.980145
H	-1.108771	0.501332	4.483846
C	-2.614414	-2.075554	2.891742
H	-3.531743	-1.843414	3.430556
H	-2.851527	-2.282668	1.848699
H	-2.097158	-2.914381	3.355607
I	-3.359948	-1.716712	-1.242517
C	4.639848	-0.289554	-1.451503
C	4.401507	0.718711	-0.521748
C	3.162244	1.335340	-0.412835
C	2.128372	0.906216	-1.245982
C	2.340151	-0.089288	-2.191636
C	3.600726	-0.680045	-2.286662
H	5.614009	-0.759932	-1.515985
H	3.029150	2.119366	0.320934
H	1.553768	-0.433070	-2.849392
H	3.761950	-1.466055	-3.015951
N	0.789777	1.552191	-1.110981
C	-0.317579	0.698977	-1.675199
H	-1.260687	1.190137	-1.428560
H	-0.211748	0.627418	-2.755828
H	-0.277843	-0.288002	-1.212520
C	0.785256	2.873588	-1.833875
H	-0.204791	3.315536	-1.702680
H	1.553869	3.510892	-1.398247
H	0.995538	2.689288	-2.886766
C	0.457757	1.792176	0.336861
H	-0.574084	2.146398	0.378699
H	0.579902	0.848946	0.869248
H	1.116926	2.551806	0.749139
I	-3.213641	3.133322	-0.164726
Br	5.794889	1.259754	0.638251
H	2.929802	-0.626665	2.875384
Br	0.963101	-3.086345	-1.007529

3Br_dimer_stacked_CONF3
Electronic energy = -5976.425949
Thermal correction to Gibbs free energy (25°C) = 0.083213
Thermal correction to Gibbs free energy (80°C) = 0.107628
qh-G(25°C) = -5976.060815
qh-G(80°C) = -5976.075597

Geometry:

C	-2.368964	1.022270	-1.167555
C	-1.897525	2.322528	-1.045525
C	-0.606735	2.679388	-1.436686
C	0.232191	1.685129	-1.931179
C	-0.209196	0.368083	-2.053415
C	-1.511571	0.050740	-1.680126
H	-3.378017	0.768900	-0.858852
H	-0.292029	3.707520	-1.323088
H	0.444257	-0.425225	-2.400096
N	1.653971	1.993510	-2.267687
C	2.525909	1.532405	-1.120872
H	3.565541	1.731801	-1.383451
H	2.248841	2.093345	-0.228971
H	2.374229	0.460788	-0.973756
C	1.887241	3.460136	-2.486292
H	2.924577	3.582427	-2.792023
H	1.217671	3.813212	-3.270124
H	1.724764	3.998909	-1.555405
C	2.076360	1.270379	-3.522411
H	3.075966	1.617916	-3.779426
H	2.106842	0.198681	-3.329182
H	1.368848	1.513017	-4.314624
I	2.396627	-2.522828	-1.791250
C	-4.458291	-0.772126	1.421892
C	-3.969537	0.248595	2.235438
C	-2.606531	0.369438	2.485677
C	-1.723717	-0.545759	1.914011
C	-2.186128	-1.584381	1.114680
C	-3.555263	-1.671242	0.870760
H	-5.518723	-0.859564	1.216265
H	-2.258106	1.176320	3.119934
H	-1.524542	-2.308547	0.659260
N	-0.262840	-0.366356	2.151863
C	0.579946	-1.332820	1.365832
H	1.624378	-1.083273	1.562145
H	0.366880	-2.349587	1.693919
H	0.371698	-1.212013	0.302540
C	0.055448	-0.552983	3.611536
H	1.131093	-0.411416	3.731849
H	-0.492475	0.185568	4.193672

H -0.241183 -1.561072 3.898839
C 0.145890 1.020175 1.728339
H 1.226158 1.102213 1.869160
H -0.124780 1.135597 0.679051
H -0.374327 1.758692 2.334144
I 4.076172 0.476103 2.153016
Br -4.183308 -3.051233 -0.260394
Br -3.025451 3.652503 -0.314811
H -4.657505 0.961648 2.675433
H -1.856933 -0.973677 -1.782482

3Br_dimer_stacked_CONF4
Electronic energy = -5976.427889
Thermal correction to Gibbs free energy (25°C) = 0.083790
Thermal correction to Gibbs free energy (80°C) = 0.108311
qh-G(25°C) = -5976.063973
qh-G(80°C) = -5976.078816

Geometry:
C 1.726817 -1.152617 2.416799
C 0.896748 -0.927834 3.510502
C -0.468862 -1.213489 3.450971
C -1.004247 -1.716543 2.268852
C -0.195622 -1.949997 1.157568
C 1.163486 -1.673961 1.255190
H 2.788078 -0.933440 2.465508
H -1.076077 -1.027097 4.326105
H -0.594186 -2.308652 0.213903
N -2.470364 -1.965712 2.130605
C -3.080408 -0.811893 1.366668
H -4.146968 -1.007175 1.247250
H -2.926444 0.099992 1.942618
H -2.599304 -0.747110 0.388179
C -3.154281 -2.077555 3.462326
H -4.195218 -2.334310 3.275423
H -2.670248 -2.861085 4.044543
H -3.110154 -1.120471 3.976838
C -2.731372 -3.243472 1.372702
H -3.804553 -3.427048 1.398560
H -2.414709 -3.121892 0.337436
H -2.190615 -4.051970 1.863227
I -1.780447 -1.757246 -2.396856
C 4.560416 1.842814 1.062685
C 3.704285 2.552515 1.903117
C 2.351229 2.670397 1.606207
C 1.848380 2.060742 0.456757
C 2.683597 1.364592 -0.408334
C 4.035588 1.264465 -0.084168

H	5.614497	1.747246	1.295512
H	1.714256	3.236758	2.274775
H	2.325871	0.896614	-1.314626
N	0.392697	2.189833	0.159036
C	-0.057939	1.287663	-0.957838
H	-1.141249	1.392207	-1.039874
H	0.416150	1.595148	-1.888876
H	0.195079	0.255555	-0.711646
C	0.074371	3.610205	-0.229269
H	-0.997422	3.661552	-0.432534
H	0.341450	4.268636	0.595752
H	0.654425	3.858564	-1.117486
C	-0.418274	1.819256	1.371840
H	-1.471474	1.887803	1.090618
H	-0.145785	0.802951	1.655195
H	-0.205499	2.508593	2.185073
I	-3.947011	2.352427	-0.419229
Br	5.177615	0.315476	-1.255781
H	4.096305	3.020350	2.799072
H	1.313001	-0.526199	4.427452
Br	2.271707	-2.061495	-0.229032

3Br_ionpair_displ_conf2
Electronic energy = -2988.206039
Thermal correction to Gibbs free energy (25°C) = 0.052131
Thermal correction to Gibbs free energy (80°C) = 0.066222
qh-G(25°C) = -2988.034974
qh-G(80°C) = -2988.044411

Geometry:

C	-3.194444	-1.143967	0.000175
C	-1.802322	-1.152209	0.000015
C	-1.063203	0.021993	-0.000078
C	-1.749693	1.235652	-0.000004
C	-3.138342	1.280860	0.000173
C	-3.851284	0.080273	0.000261
H	-3.687109	2.212401	0.000249
H	-3.750852	-2.073998	0.000243
N	-0.928000	2.483268	-0.000089
C	-0.054681	2.509876	-1.229859
H	0.493369	3.452002	-1.228454
H	-0.699156	2.438643	-2.105301
H	0.645714	1.675713	-1.187329
C	-0.054609	2.509966	1.229626
H	0.493493	3.452064	1.228088
H	0.645731	1.675753	1.187147
H	-0.699044	2.438863	2.105109
C	-1.764227	3.727734	-0.000116

H -1.081286 4.575175 -0.000151
H -2.378896 3.749869 0.898277
H -2.378924 3.749812 -0.898490
H 0.023405 -0.028953 -0.000186
I 2.917898 -0.019194 0.000063
H -4.935054 0.108684 0.000399
Br -0.880204 -2.806078 -0.000099

3Cl_SnArProduct_GEN
Electronic energy = -997.875256
Thermal correction to Gibbs free energy (25°C) = 0.046743
Thermal correction to Gibbs free energy (80°C) = 0.059603
qh-G(25°C) = -997.732842
qh-G(80°C) = -997.741360

Geometry:
C 3.036188 0.821000 1.308861
C 1.947711 -0.032694 1.144983
C 1.766125 -0.666867 -0.082965
C 2.650843 -0.472239 -1.137488
C 3.742099 0.379297 -0.958331
C 3.935110 1.029050 0.260170
H 3.185292 1.319405 2.261252
H 1.244264 -0.208650 1.953151
H 2.478617 -0.985509 -2.077717
H 4.438306 0.535390 -1.775983
H 4.782705 1.692682 0.394918
O 0.730167 -1.568424 -0.262687
C -0.562143 -1.141885 -0.094273
C -1.513547 -2.129912 0.163073
C -2.850245 -1.770741 0.297263
H -1.191411 -3.161259 0.254681
C -2.279316 0.519417 -0.077016
C -3.251980 -0.438885 0.182653
H -3.592296 -2.535694 0.500056
H -4.291392 -0.152808 0.292681
C -0.933227 0.197056 -0.222867
H -0.199614 0.966979 -0.432115
Cl -2.754207 2.195369 -0.233753

3Cl_TMA_I_TS
Electronic energy = -876.837424
Thermal correction to Gibbs free energy (25°C) = 0.052649
Thermal correction to Gibbs free energy (80°C) = 0.066949
qh-G(25°C) = -876.671019
qh-G(80°C) = -876.680629

Geometry:
C 3.612873 -0.604593 -1.283094
C 3.094960 -0.915166 -0.030574
C 2.143832 -0.122449 0.597709
C 1.679732 1.036084 -0.043624
C 2.192689 1.364104 -1.305264
C 3.146574 0.546806 -1.908703
H 4.356231 -1.241900 -1.747598
H 1.778078 -0.423462 1.571080
H 1.869806 2.254508 -1.827452
H 3.534752 0.820348 -2.884211
N 0.645341 1.811907 0.565986
C 0.386974 3.114590 -0.061671
H -0.414774 3.606741 0.488956
H 1.278961 3.751105 -0.042736
H 0.057397 2.978829 -1.092767
C 0.742163 1.935009 2.031013
H -0.080796 2.560842 2.377707
H 0.644676 0.961720 2.510504
H 1.694601 2.391456 2.321653
C -1.112162 0.721752 0.233631
Cl 3.652037 -2.355478 0.788987
I -3.270563 -0.659223 -0.172263
H -1.680018 1.494978 0.730510
H -0.932463 0.781614 -0.829521
H -0.694431 -0.095022 0.802572

3Cl_TMA_SN2_tsopt_GEN
Electronic energy = -1172.210289
Thermal correction to Gibbs free energy (25°C) = 0.059635
Thermal correction to Gibbs free energy (80°C) = 0.076969
qh-G(25°C) = -1171.951437
qh-G(80°C) = -1171.962282

Geometry:
C 3.669573 1.738707 0.229490
C 3.856291 0.430940 -0.195175
C 2.878298 -0.548725 -0.037855
C 1.666794 -0.207010 0.567371
C 1.458592 1.109899 0.998097
C 2.454248 2.065125 0.829160
H 4.448927 2.479739 0.095320
H 3.087823 -1.552421 -0.382155
H 0.525615 1.405768 1.462394
H 2.279897 3.080658 1.167516
N 0.596337 -1.170532 0.698796
C -0.783598 -0.637600 -0.496425
H -1.400291 -1.489996 -0.239655

H	-0.937863	0.307819	0.007093
H	-0.135333	-0.680771	-1.361546
C	-0.002454	-1.192288	2.052598
H	-0.818330	-1.916600	2.053114
H	0.747728	-1.482327	2.793545
H	-0.409933	-0.215305	2.306184
C	0.950336	-2.541219	0.289404
H	1.230036	-2.554772	-0.764763
H	1.767732	-2.936120	0.900086
H	0.069660	-3.170021	0.422334
C	-3.295386	0.138234	-1.030203
C	-3.566103	1.427573	-0.509885
C	-4.224691	-0.887194	-0.728676
C	-4.711047	1.676851	0.244008
H	-2.860849	2.226457	-0.726748
C	-5.366777	-0.628490	0.026310
H	-4.030439	-1.884829	-1.115342
C	-5.623333	0.654242	0.518968
H	-4.893972	2.679890	0.621271
H	-6.064737	-1.436048	0.232601
H	-6.514925	0.852564	1.105068
O	-2.205611	-0.105365	-1.731955
Cl	5.365606	-0.015959	-0.950315

3Cl_TMA_SnAr_tsopt_GEN
Electronic energy = -1172.216741
Thermal correction to Gibbs free energy (25°C) = 0.056658
Thermal correction to Gibbs free energy (80°C) = 0.073495
qh-G(25°C) = -1171.954773
qh-G(80°C) = -1171.965158

Geometry:

C	-1.387429	-1.892904	1.318305
C	-0.598240	-0.966155	2.002512
C	-0.270221	0.272181	1.471691
C	-0.673381	0.640793	0.152124
C	-1.544481	-0.277475	-0.517375
C	-1.835343	-1.498232	0.058661
H	-1.649440	-2.855512	1.738010
H	-0.226930	-1.214686	2.993066
H	0.370547	0.933315	2.042983
N	-1.091218	2.145379	-0.006085
C	-2.411120	2.313417	0.683168
H	-2.691190	3.365699	0.628488
H	-3.151313	1.693498	0.179307
H	-2.306033	1.996614	1.719849
C	-1.260794	2.561750	-1.436419
H	-1.624151	3.589662	-1.437699

H -0.301677 2.483439 -1.937422
H -2.001739 1.923296 -1.912537
C -0.107913 3.073706 0.638858
H -0.126047 2.924323 1.716342
H 0.876645 2.866713 0.228109
H -0.418353 4.094214 0.414375
O 0.768405 0.887683 -0.890732
C 1.842339 0.165150 -0.564551
C 1.786717 -1.230748 -0.377355
C 3.087151 0.805826 -0.409031
C 2.937941 -1.948428 -0.059416
H 0.832801 -1.740152 -0.481485
C 4.236220 0.077284 -0.106667
C 4.171297 -1.305888 0.074081
H 2.871239 -3.024126 0.080204
H 5.186470 0.594687 -0.006359
H 5.064786 -1.873054 0.314560
H 3.134391 1.882928 -0.546522
H -1.912399 -0.063041 -1.512756
Cl -2.839575 -2.614035 -0.860211

3Cl_TMA_bromide_tsopt
Electronic energy = -3436.979274
Thermal correction to Gibbs free energy (25°C) = 0.051625
Thermal correction to Gibbs free energy (80°C) = 0.065719
qh-G(25°C) = -3436.811415
qh-G(80°C) = -3436.820833

Geometry:
C 2.985639 -1.002104 -1.266185
C 2.962196 -0.560934 0.048969
C 1.981004 0.303688 0.528210
C 0.978152 0.747698 -0.340232
C 0.986436 0.308787 -1.673117
C 1.980363 -0.552550 -2.121788
H 3.762242 -1.674466 -1.611355
H 2.026219 0.619800 1.561536
H 0.220009 0.626979 -2.369484
H 1.970317 -0.882954 -3.154866
N -0.087865 1.586152 0.123206
C -0.400100 2.717901 -0.769522
H -1.259336 3.251408 -0.360255
H 0.453088 3.401549 -0.837483
H -0.660894 2.365786 -1.765911
C 0.031685 2.044085 1.512998
H -0.846947 2.644826 1.750424
H 0.057240 1.189017 2.190558
H 0.928778 2.656719 1.657872

C -1.782909 0.389070 0.135680
Cl 4.207167 -1.095835 1.152264
H -2.341516 1.207515 0.564012
H -1.712764 0.284990 -0.936053
H -1.264432 -0.308037 0.776471
Br -3.730674 -0.978024 0.164386

3Cl_TMA_chloride_tsopt
Electronic energy = -1325.657884
Thermal correction to Gibbs free energy (25°C) = 0.050398
Thermal correction to Gibbs free energy (80°C) = 0.064265
qh-G(25°C) = -1325.488375
qh-G(80°C) = -1325.497594

Geometry:
C 2.753678 0.274732 -1.086478
C 2.226543 -0.450992 -0.022689
C 0.999370 -0.142444 0.547844
C 0.257792 0.937213 0.045319
C 0.772877 1.676598 -1.025286
C 2.008239 1.340412 -1.578142
H 3.715664 0.012628 -1.511158
H 0.638995 -0.750112 1.368774
H 0.234656 2.520789 -1.434529
H 2.394859 1.927340 -2.404380
N -1.037681 1.209589 0.598009
C -1.694100 2.416337 0.076920
H -2.672140 2.502785 0.551312
H -1.110595 3.316800 0.298670
H -1.840486 2.329474 -1.000723
C -1.085483 1.193556 2.072476
H -2.115914 1.370668 2.383943
H -0.776200 0.225043 2.461676
H -0.440723 1.975519 2.487406
C -2.255418 -0.327229 -0.022608
Cl 3.132296 -1.798616 0.621298
H -3.088292 0.154388 0.467373
H -2.013444 -0.084574 -1.046418
H -1.641545 -1.029814 0.519894
Cl -3.598299 -1.992393 -0.702006

3Cl_TMA_gen
Electronic energy = -865.356384
Thermal correction to Gibbs free energy (25°C) = 0.043833
Thermal correction to Gibbs free energy (80°C) = 0.056085
qh-G(25°C) = -865.179595
qh-G(80°C) = -865.187718

Geometry:
C -0.590374 0.237840 -0.000008
C 0.523417 -0.592742 -0.000018
C 1.786409 -0.001688 -0.000005
C 1.952636 1.376326 0.000004
C 0.817576 2.184420 0.000001
C -0.455551 1.625549 -0.000003
H 0.454957 -1.671564 -0.000035
H 2.946663 1.808307 0.000008
H 0.925188 3.263001 -0.000001
H -1.317578 2.282319 0.000000
Cl 3.185637 -1.036373 0.000000
N -1.970493 -0.335624 -0.000005
C -2.705809 0.125672 1.232901
H -2.787724 1.209705 1.218572
H -3.697921 -0.324035 1.222166
H -2.142577 -0.201420 2.105683
C -2.705996 0.126058 -1.232639
H -2.142966 -0.200848 -2.105621
H -3.698154 -0.323548 -1.221830
H -2.787807 1.210097 -1.218020
C -1.977860 -1.836794 -0.000236
H -1.481379 -2.197974 -0.899258
H -1.481302 -2.198245 0.898635
H -3.018458 -2.155923 -0.000238

3Cl_aniline_gen
Electronic energy = -825.634034
Thermal correction to Gibbs free energy (25°C) = 0.042655
Thermal correction to Gibbs free energy (80°C) = 0.054099
qh-G(25°C) = -825.500619
qh-G(80°C) = -825.508444

Geometry:
C -0.897643 0.158093 -0.072748
C 0.288403 -0.610740 -0.046781
C 1.520016 0.028194 -0.006238
C 1.657774 1.410820 0.022819
C 0.483223 2.164387 0.014360
C -0.770189 1.566321 -0.027102
H 0.258999 -1.692203 -0.059324
H 2.635599 1.875729 0.057098
H 0.547612 3.247665 0.045397
H -1.649974 2.196742 -0.024765
Cl 2.964948 -0.966913 0.018139
N -2.131232 -0.449497 -0.147955
C -3.322594 0.351074 0.077202

H -3.410032 1.139912 -0.675727
H -4.198901 -0.289839 -0.010337
H -3.327695 0.818466 1.072229
C -2.221501 -1.885990 0.046954
H -1.659648 -2.420308 -0.725247
H -1.841592 -2.196981 1.030812
H -3.264800 -2.188134 -0.033615

3Cl_bromide_ionpair1_gen
Electronic energy = -3437.031683
Thermal correction to Gibbs free energy (25°C) = 0.050164
Thermal correction to Gibbs free energy (80°C) = 0.063890
qh-G(25°C) = -3436.858322
qh-G(80°C) = -3436.867430

Geometry:
Br 3.043600 0.076706 -0.000101
N -0.768817 -1.977045 0.000092
C -1.450602 -0.647417 0.000017
C -2.836059 -0.541331 -0.000185
C -3.414125 0.729904 -0.000273
C -2.628248 1.875450 -0.000161
C -1.243301 1.730460 0.000006
C 0.097737 -2.097206 1.229211
H 0.537869 -3.094226 1.229255
H -0.534447 -1.954921 2.104870
H 0.885705 -1.345421 1.181674
C 0.097733 -2.097276 -1.229033
H 0.537907 -3.094277 -1.229001
H 0.885655 -1.345429 -1.181562
H -0.534461 -1.955090 -2.104696
C -1.736518 -3.122060 0.000145
H -1.152158 -4.040588 0.000119
H -2.349647 -3.075051 -0.898750
H -2.349574 -3.075030 0.899087
C -0.634883 0.483015 0.000103
H 0.451596 0.416714 0.000275
H -3.482801 -1.407487 -0.000225
H -3.076238 2.862544 -0.000213
H -4.494568 0.818859 -0.000422
Cl -0.234090 3.150751 0.000205

3Cl_bromide_ionpair2_gen
Electronic energy = -3437.031147
Thermal correction to Gibbs free energy (25°C) = 0.050181
Thermal correction to Gibbs free energy (80°C) = 0.063922
qh-G(25°C) = -3436.857758

qh-G(80°C) = -3436.866879

Geometry:

Br	-3.631874	-0.785380	-0.000110
N	-0.067661	1.660538	0.000055
C	0.777003	0.428678	0.000085
C	2.164076	0.515791	-0.000082
C	2.896014	-0.670867	-0.000027
C	2.279681	-1.914789	0.000177
C	0.887152	-1.967377	0.000310
C	-0.942492	1.666142	1.229400
H	-1.506203	2.599021	1.230602
H	-0.297250	1.603915	2.105005
H	-1.627898	0.820055	1.179121
C	-0.942775	1.665902	-1.229044
H	-1.506415	2.598826	-1.230378
H	-1.628257	0.819899	-1.178418
H	-0.297766	1.603406	-2.104816
C	0.740020	2.922941	-0.000149
H	0.038694	3.755297	-0.000108
H	1.353543	2.959496	-0.899006
H	1.353775	2.959632	0.898543
C	0.125904	-0.803461	0.000273
H	-0.959307	-0.873355	0.000342
H	2.696339	1.456940	-0.000286
H	2.874979	-2.820650	0.000231
H	0.386336	-2.929199	0.000466
Cl	4.635362	-0.573324	-0.000204

3Cl_chloride_ionpair1_gen
Electronic energy = -1325.709746
Thermal correction to Gibbs free energy (25°C) = 0.049425
Thermal correction to Gibbs free energy (80°C) = 0.063066
qh-G(25°C) = -1325.535818
qh-G(80°C) = -1325.544827

Geometry:

Cl	0.671368	3.364097	0.000242
N	1.863157	-0.615142	-0.000096
C	0.426023	-1.023503	-0.000064
C	0.051784	-2.361636	0.000188
C	-1.307793	-2.680531	0.000312
C	-2.277978	-1.686341	0.000189
C	-1.865752	-0.356203	-0.000068
C	2.149946	0.211737	1.228979
H	3.215256	0.442156	1.233287
H	1.880227	-0.377269	2.104903
H	1.571990	1.134538	1.175118

C	2.149946	0.211733	-1.229169
H	3.215281	0.442037	-1.233514
H	1.572098	1.134592	-1.175248
H	1.880116	-0.377228	-2.105089
C	2.796492	-1.787810	-0.000123
H	3.811330	-1.394045	-0.000249
H	2.630794	-2.380147	-0.898708
H	2.630994	-2.380033	0.898581
C	-0.523401	-0.002306	-0.000204
H	-0.243463	1.050239	-0.000382
H	0.774311	-3.165732	0.000265
H	-3.333443	-1.933379	0.000296
H	-1.605812	-3.722866	0.000513
Cl	-3.061802	0.911332	-0.000203

3Cl_chloride_ionpair2_gen
Electronic energy = -1325.709231
Thermal correction to Gibbs free energy (25°C) = 0.049578
Thermal correction to Gibbs free energy (80°C) = 0.063244
qh-G(25°C) = -1325.535441
qh-G(80°C) = -1325.544474

Geometry:

Cl	-3.949026	-1.438238	0.000212
N	-0.908930	1.400000	-0.000058
C	0.106042	0.303990	-0.000083
C	1.465806	0.591570	0.000048
C	2.362444	-0.476259	0.000027
C	1.933652	-1.796350	-0.000116
C	0.563348	-2.050754	-0.000276
C	-1.775806	1.277542	1.229001
H	-2.466966	2.120417	1.232488
H	-1.128054	1.307266	2.104349
H	-2.332939	0.342042	1.174791
C	-1.775721	1.277647	-1.229185
H	-2.466925	2.120486	-1.232615
H	-2.332798	0.342109	-1.175126
H	-1.127913	1.307513	-2.104486
C	-0.292268	2.765781	0.000033
H	-1.106379	3.488303	-0.000038
H	0.309180	2.890209	-0.899389
H	0.309006	2.890180	0.899568
C	-0.359352	-1.009961	-0.000276
H	-1.423985	-1.234247	-0.000461
H	1.857221	1.599400	0.000184
H	2.653969	-2.606389	-0.000104
H	0.207870	-3.075220	-0.000407
Cl	4.069164	-0.125956	0.000177

3Me_Aniline_gen
Electronic energy = -405.364795
Thermal correction to Gibbs free energy (25°C) = 0.041667
Thermal correction to Gibbs free energy (80°C) = 0.053008
qh-G(25°C) = -405.193709
qh-G(80°C) = -405.201378

Geometry:

C	-0.554401	0.137974	-0.092493
C	0.507698	-0.792635	-0.046903
C	1.840852	-0.385241	0.011435
C	2.143993	0.980493	0.044185
C	1.106966	1.910265	0.019233
C	-0.224979	1.509733	-0.042279
H	0.299678	-1.856555	-0.057902
H	3.178384	1.307805	0.098694
H	1.332870	2.972286	0.057336
H	-0.999902	2.265842	-0.048996
N	-1.872477	-0.282900	-0.193746
C	-2.924264	0.679579	0.086639
H	-2.893711	1.507641	-0.626712
H	-3.890540	0.187273	-0.021269
H	-2.853029	1.095612	1.103099
C	-2.173130	-1.679633	0.069926
H	-1.673556	-2.328442	-0.654702
H	-1.869625	-1.991770	1.080800
H	-3.246776	-1.834420	-0.034403
C	2.946309	-1.412770	0.019461
H	2.578515	-2.390500	0.339916
H	3.372994	-1.528843	-0.982524
H	3.757771	-1.112210	0.687665

3Me_SnArProduct_GEN
Electronic energy = -577.608382
Thermal correction to Gibbs free energy (25°C) = 0.047560
Thermal correction to Gibbs free energy (80°C) = 0.060742
qh-G(25°C) = -577.429007
qh-G(80°C) = -577.437618

Geometry:

C	2.725932	0.919918	1.282805
C	1.554267	0.226698	0.981865
C	1.503738	-0.547663	-0.177889
C	2.606821	-0.641580	-1.024429
C	3.773484	0.051907	-0.707552
C	3.837771	0.837916	0.443677

H 2.767739 1.523149 2.184232
H 0.691173 0.285273 1.636924
H 2.536558 -1.255122 -1.916593
H 4.632314 -0.020287 -1.367298
H 4.745651 1.380073 0.685774
O 0.402989 -1.307637 -0.508785
C -0.858433 -0.837558 -0.198371
C -1.757358 -1.737626 0.362284
C -3.061900 -1.311646 0.615295
H -1.432294 -2.747843 0.586910
C -2.542013 0.897337 -0.247094
C -3.450853 -0.007907 0.317663
H -3.775808 -2.004361 1.049734
H -4.468766 0.313806 0.519617
C -1.239663 0.468031 -0.509629
H -0.517510 1.145777 -0.957366
C -2.958469 2.314216 -0.552053
H -2.290363 2.775007 -1.283008
H -3.978174 2.347612 -0.943995
H -2.934377 2.925752 0.355918

3Me_TMA_DMSO_tsopt_GEN_3
Electronic energy = -998.158141
Thermal correction to Gibbs free energy (25°C) = 0.062349
Thermal correction to Gibbs free energy (80°C) = 0.080246
qh-G(25°C) = -997.875429
qh-G(80°C) = -997.886628

Geometry:
C 2.824867 -1.726368 -0.929692
C 2.693776 -1.420294 0.429846
C 2.163380 -0.186589 0.808807
C 1.750145 0.768867 -0.146477
C 1.912850 0.448480 -1.511329
C 2.438200 -0.786968 -1.882399
H 3.233666 -2.685443 -1.234510
H 2.074812 0.028810 1.867910
H 1.630901 1.153170 -2.283915
H 2.547559 -1.012879 -2.939311
N 1.161281 1.961014 0.244808
C 0.987733 3.009334 -0.746117
H 0.536841 3.877572 -0.266531
H 1.939501 3.314728 -1.204082
H 0.312841 2.684366 -1.545145
C 1.241858 2.356642 1.640445
H 0.751100 3.321735 1.762228
H 0.721833 1.637113 2.281719
H 2.279918 2.442824 1.992577

C -1.396875 0.401239 0.050364
H -0.906744 0.304877 -0.921271
H -0.830276 -0.118797 0.825961
H -1.556855 1.449639 0.306030
S -3.002712 -0.344391 -0.106638
O -3.823695 0.309869 -1.134727
C -3.754349 -0.270910 1.501196
H -3.896753 0.785523 1.733219
H -4.713233 -0.785250 1.424984
H -3.098157 -0.753913 2.226159
C -2.732869 -2.063839 -0.460126
H -3.716189 -2.534744 -0.500909
H -2.238755 -2.107372 -1.431733
H -2.112303 -2.499896 0.323403
C 3.154457 -2.401178 1.479824
H 2.718424 -2.174760 2.455624
H 2.882725 -3.424523 1.207305
H 4.244082 -2.369086 1.584609

3Me_TMA_I_TS
Electronic energy = -456.571373
Thermal correction to Gibbs free energy (25°C) = 0.053096
Thermal correction to Gibbs free energy (80°C) = 0.067634
qh-G(25°C) = -456.367488
qh-G(80°C) = -456.377108

Geometry:
C -3.848841 1.230325 -0.884315
C -3.110203 1.461054 0.284436
C -2.197757 0.496335 0.705104
C -2.008399 -0.696882 -0.012291
C -2.749556 -0.910322 -1.177629
C -3.664119 0.056918 -1.602933
H -4.564431 1.973904 -1.224278
H -1.623641 0.691952 1.605231
H -2.639234 -1.817359 -1.757603
H -4.237462 -0.122400 -2.507067
N -1.017768 -1.631439 0.436785
C -0.896260 -2.850857 -0.366451
H -0.084561 -3.453065 0.044571
H -1.819513 -3.442398 -0.344322
H -0.651107 -2.597601 -1.399432
C -1.095798 -1.961128 1.869286
H -0.248578 -2.600237 2.124972
H -1.040516 -1.060888 2.478724
H -2.028940 -2.491303 2.093532
C 0.855771 -0.668765 0.170340
I 3.126769 0.486958 -0.165760

H 1.338551 -1.584658 0.476278
H 0.587783 -0.517082 -0.864311
H 0.563010 0.059844 0.910448
C -3.299163 2.737058 1.065015
H -3.071699 3.608406 0.443770
H -4.336043 2.836373 1.399765
H -2.652059 2.765359 1.944130

3Me_TMA_SN2_tsopt_GEN
Electronic energy = -751.944380
Thermal correction to Gibbs free energy (25°C) = 0.060414
Thermal correction to Gibbs free energy (80°C) = 0.078064
qh-G(25°C) = -751.648520
qh-G(80°C) = -751.659453

Geometry:
C -4.017014 -1.711761 0.047240
C -4.218734 -0.409110 -0.405256
C -3.209828 0.543858 -0.205086
C -2.018793 0.206758 0.438199
C -1.827333 -1.108993 0.884382
C -2.825492 -2.054989 0.687668
H -4.790134 -2.460124 -0.100098
H -3.387896 1.553041 -0.558019
H -0.910065 -1.407003 1.378623
H -2.669383 -3.070844 1.036019
N -0.944384 1.165224 0.599492
C 0.482330 0.610161 -0.545826
H 1.087077 1.466521 -0.274000
H 0.609221 -0.327025 -0.019706
H -0.144049 0.646133 -1.427292
C -0.394137 1.196992 1.971479
H 0.429403 1.912775 1.994532
H -1.166327 1.502982 2.683458
H -0.007156 0.218607 2.250885
C -1.271165 2.533209 0.164852
H -1.517034 2.537859 -0.897842
H -2.105823 2.942640 0.742070
H -0.390047 3.156655 0.321048
C 3.005414 -0.157762 -0.984338
C 3.284853 -1.439664 -0.451525
C 3.905152 0.886521 -0.660101
C 4.411832 -1.664492 0.336301
H 2.601165 -2.252284 -0.685401
C 5.029114 0.652542 0.129540
H 3.704080 1.878740 -1.057117
C 5.295525 -0.623355 0.634282
H 4.602899 -2.662559 0.722549

H	5.705177	1.474038	0.352952
H	6.173205	-0.802494	1.246978
O	1.931960	0.062230	-1.719734
C	-5.496774	-0.005562	-1.095889
H	-6.019232	0.768852	-0.526155
H	-5.290948	0.401721	-2.090117
H	-6.168821	-0.858750	-1.207465

3Me_TMA_SnAr_tsopt_GEN
Electronic energy = -751.945646
Thermal correction to Gibbs free energy (25°C) = 0.057265
Thermal correction to Gibbs free energy (80°C) = 0.074409
qh-G(25°C) = -751.646721
qh-G(80°C) = -751.657185

Geometry:

C	1.090453	2.482409	0.988067
C	0.614183	1.472841	1.822264
C	0.556583	0.143221	1.421451
C	0.932152	-0.235021	0.097413
C	1.483425	0.807224	-0.718289
C	1.527841	2.128780	-0.300457
H	1.138435	3.513679	1.323639
H	0.276605	1.717653	2.826820
H	0.144071	-0.595839	2.098740
N	1.739917	-1.599991	0.020902
C	3.102538	-1.333467	0.577980
H	3.654847	-2.273665	0.596474
H	3.605087	-0.606277	-0.058226
H	2.993991	-0.931337	1.584356
C	1.903752	-2.112554	-1.376605
H	2.557779	-2.984382	-1.334905
H	0.927163	-2.375717	-1.769093
H	2.371479	-1.345116	-1.989387
C	1.102588	-2.675338	0.843029
H	1.164101	-2.406018	1.895460
H	0.068497	-2.781786	0.525059
H	1.658625	-3.598656	0.677018
O	-0.430721	-0.963065	-0.759081
C	-1.642871	-0.488998	-0.463167
C	-1.920040	0.887514	-0.333123
C	-2.705288	-1.398558	-0.292319
C	-3.214807	1.322110	-0.055463
H	-1.114027	1.605994	-0.448955
C	-3.998848	-0.951607	-0.030139
C	-4.265404	0.413551	0.093747
H	-3.405651	2.387903	0.039523
H	-4.801482	-1.675301	0.083500

H	-5.271438	0.763006	0.302672
H	-2.492861	-2.460503	-0.383962
H	1.804585	0.574453	-1.728614
C	2.024694	3.195217	-1.247403
H	2.573806	2.761412	-2.086865
H	1.185496	3.768695	-1.656241
H	2.681542	3.902314	-0.732588

3Me_TMA_bromide_tsopt
Electronic energy = -3016.712906
Thermal correction to Gibbs free energy (25°C) = 0.052196
Thermal correction to Gibbs free energy (80°C) = 0.066575
qh-G(25°C) = -3016.507794
qh-G(80°C) = -3016.517277

Geometry:

C	3.435418	-1.005004	-0.869555
C	2.692677	-1.336203	0.272039
C	1.686136	-0.470464	0.694061
C	1.408439	0.721808	0.005189
C	2.155078	1.036235	-1.132952
C	3.163375	0.168162	-1.560171
H	4.222801	-1.671287	-1.211056
H	1.109521	-0.744402	1.571998
H	1.976458	1.945902	-1.691208
H	3.739518	0.424653	-2.443646
N	0.323363	1.549872	0.452664
C	0.113012	2.776692	-0.322349
H	-0.759500	3.291730	0.082479
H	0.978601	3.446693	-0.258730
H	-0.082483	2.532639	-1.368011
C	0.348451	1.853618	1.893898
H	-0.560838	2.401252	2.148846
H	0.373875	0.939005	2.483624
H	1.221923	2.467330	2.142910
C	-1.422943	0.442639	0.120036
H	-1.998608	1.303273	0.425406
H	-1.116497	0.332134	-0.908946
H	-1.090103	-0.272678	0.855706
C	2.982712	-2.608092	1.027876
H	2.266682	-2.761294	1.837968
H	2.939505	-3.474473	0.361697
H	3.987053	-2.579091	1.461551
Br	-3.395588	-0.797338	-0.269362

3Me_TMA_chloride_tsopt
Electronic energy = -905.391701

Thermal correction to Gibbs free energy (25°C) = 0.051154
Thermal correction to Gibbs free energy (80°C) = 0.065337
qh-G(25°C) = -905.185281
qh-G(80°C) = -905.194585

Geometry:

C	3.025267	-0.464600	-0.833428
C	2.331902	-1.029282	0.245659
C	1.143779	-0.435538	0.666510
C	0.635311	0.712373	0.036869
C	1.336230	1.261573	-1.039536
C	2.526937	0.666832	-1.464983
H	3.952800	-0.917391	-1.172826
H	0.610935	-0.888754	1.496363
H	0.982578	2.147653	-1.550149
H	3.064990	1.104566	-2.299909
N	-0.623873	1.250457	0.474962
C	-1.054241	2.469678	-0.218171
H	-2.030440	2.759378	0.173491
H	-0.350027	3.294053	-0.056382
H	-1.152873	2.279061	-1.288202
C	-0.728647	1.429514	1.934011
H	-1.740023	1.766313	2.168977
H	-0.551889	0.489811	2.454394
H	-0.007595	2.177211	2.283272
C	-2.061688	-0.152410	-0.037754
H	-2.819614	0.528399	0.319437
H	-1.714378	-0.091704	-1.058135
H	-1.600378	-0.851938	0.641879
C	2.868357	-2.256585	0.937739
H	2.172771	-2.622014	1.695998
H	3.047392	-3.060942	0.218598
H	3.822175	-2.037613	1.427491
Cl	-3.615210	-1.649331	-0.596501

3Me_TMA_gen
Electronic energy = -445.092991
Thermal correction to Gibbs free energy (25°C) = 0.044793
Thermal correction to Gibbs free energy (80°C) = 0.057383
qh-G(25°C) = -444.879355
qh-G(80°C) = -444.887581

Geometry:

C	0.253550	0.173220	-0.000189
C	-0.741861	-0.793587	-0.000181
C	-2.091931	-0.412010	-0.000086
C	-2.407051	0.945252	-0.000030
C	-1.398758	1.909237	0.000001

C -0.061792 1.532704 -0.000065
H -0.512187 -1.851869 -0.000231
H -3.448087 1.253662 0.000015
H -1.652671 2.963635 0.000077
H 0.704658 2.299290 0.000037
N 1.702246 -0.208094 -0.000020
C 2.368565 0.347975 -1.231525
H 2.297726 1.432787 -1.220201
H 3.413829 0.041213 -1.219673
H 1.857560 -0.057171 -2.103936
C 2.367727 0.346816 1.232484
H 3.412874 0.039638 1.221312
H 2.297258 1.431667 1.221843
H 1.855779 -0.058786 2.104129
C 1.914589 -1.693185 -0.000645
H 1.470483 -2.118541 0.897511
H 1.470276 -2.117834 -0.899037
H 2.989096 -1.868594 -0.000799
C -3.163528 -1.471179 0.000039
H -3.075613 -2.111942 -0.881997
H -3.076117 -2.111148 0.882707
H -4.157654 -1.020817 -0.000430

3Me_bromide_ionpair1_gen
Electronic energy = -3016.766983
Thermal correction to Gibbs free energy (25°C) = 0.051358
Thermal correction to Gibbs free energy (80°C) = 0.065487
qh-G(25°C) = -3016.557128
qh-G(80°C) = -3016.566395

Geometry:
Br -3.366300 -0.678567 -0.000163
N 0.321165 1.600925 0.000100
C 1.120748 0.335463 0.000199
C 2.508649 0.361820 0.000018
C 3.232177 -0.840268 0.000024
C 2.532665 -2.045642 0.000268
H 3.081238 -2.982836 0.000353
C 1.137348 -2.057595 0.000468
H 0.601621 -3.001058 0.000703
C -0.553368 1.641148 1.227407
H -1.082037 2.594602 1.229610
H 0.088021 1.553753 2.103681
H -1.270055 0.821561 1.177484
C -0.552700 1.641232 -1.227688
H -1.081428 2.594651 -1.230089
H -1.269339 0.821559 -1.178242
H 0.089185 1.553973 -2.103620

C 1.176022 2.830781 0.000400
H 0.508150 3.690256 0.000324
H 1.792027 2.842496 -0.897376
H 1.791661 2.842305 0.898434
C 0.417209 -0.869271 0.000425
H -0.669683 -0.896038 0.000699
H 3.064797 1.291304 -0.000121
C 4.738665 -0.801496 -0.000576
H 5.113725 -0.278451 -0.885061
H 5.114453 -0.271423 0.879371
H 5.155503 -1.810304 0.003176

3Me_bromide_ionpair2_gen_2
Electronic energy = -3016.767734
Thermal correction to Gibbs free energy (25°C) = 0.051179
Thermal correction to Gibbs free energy (80°C) = 0.065266
qh-G(25°C) = -3016.557515
qh-G(80°C) = -3016.566751

Geometry:
Br -3.060902 0.239300 -0.000171
N 0.818542 -1.744155 0.000155
C 1.486714 -0.404673 -0.000008
C 2.869605 -0.284900 -0.000375
C 3.424703 0.998671 -0.000496
C 2.612755 2.124604 -0.000226
H 3.059768 3.114944 -0.000294
C 1.217322 1.997485 0.000142
C -0.047057 -1.873804 -1.227095
H -0.477893 -2.875113 -1.228038
H 0.583046 -1.724764 -2.103232
H -0.841702 -1.129323 -1.179259
C -0.046678 -1.873753 1.227658
H -0.477488 -2.875072 1.228782
H -0.841369 -1.129315 1.180032
H 0.583646 -1.724637 2.103624
C 1.796896 -2.878410 0.000027
H 1.223655 -3.804097 0.000280
H 2.410426 -2.823733 0.898175
H 2.409916 -2.823968 -0.898484
C 0.660939 0.719717 0.000233
H -0.422661 0.621775 0.000502
H 3.529186 -1.141771 -0.000563
H 4.504250 1.104199 -0.000807
C 0.330874 3.215789 0.000521
H 0.523772 3.834316 -0.880875
H -0.724027 2.932728 -0.000253
H 0.522804 3.833047 0.883021

3Me_chloride_ionpair1_gen
Electronic energy = -905.445022
Thermal correction to Gibbs free energy (25°C) = 0.050492
Thermal correction to Gibbs free energy (80°C) = 0.064491
qh-G(25°C) = -905.234295
qh-G(80°C) = -905.243430

Geometry:

Cl	-3.670626	-1.383175	0.000207
N	-0.556786	1.389249	-0.000062
C	0.448964	0.280624	-0.000101
C	1.811741	0.545413	-0.000069
C	2.731465	-0.514053	-0.000078
C	2.250245	-1.821985	-0.000221
H	2.952008	-2.650841	-0.000321
C	0.877719	-2.074107	-0.000256
H	0.512568	-3.095764	-0.000370
C	-1.424786	1.277597	1.227614
H	-2.107848	2.127315	1.231657
H	-0.776796	1.300740	2.103099
H	-1.991547	0.347949	1.174738
C	-1.424784	1.277680	-1.227740
H	-2.107837	2.127407	-1.231745
H	-1.991563	0.348035	-1.174918
H	-0.776793	1.300865	-2.103223
C	0.073741	2.747549	-0.000001
H	-0.731952	3.479667	0.000082
H	0.677887	2.864449	-0.898453
H	0.677953	2.864341	0.898416
C	-0.036638	-1.027518	-0.000182
H	-1.103593	-1.238504	-0.000220
H	2.200621	1.556320	-0.000027
C	4.208530	-0.215156	0.000414
H	4.486549	0.372115	-0.879563
H	4.487217	0.365117	0.884844
H	4.794090	-1.136251	-0.003374

3Me_chloride_ionpair2_gen
Electronic energy = -905.445506
Thermal correction to Gibbs free energy (25°C) = 0.050317
Thermal correction to Gibbs free energy (80°C) = 0.064285
qh-G(25°C) = -905.234651
qh-G(80°C) = -905.243758

Geometry:

Cl	3.094818	1.649595	-0.000105

N	0.490883	-1.625802	0.000054
C	-0.680859	-0.694645	0.000057
C	-1.983714	-1.173669	-0.000078
C	-3.031574	-0.247925	-0.000142
C	-2.774780	1.116116	-0.000069
H	-3.600312	1.822617	-0.000133
C	-1.457514	1.594115	0.000070
C	1.330188	-1.377685	1.227479
H	2.140676	-2.106815	1.230163
H	0.695200	-1.505507	2.103607
H	1.739737	-0.368865	1.176130
C	1.329948	-1.377813	-1.227601
H	2.140412	-2.106951	-1.230397
H	1.739493	-0.368973	-1.176413
H	0.694753	-1.505740	-2.103559
C	0.084379	-3.067022	0.000184
H	0.996192	-3.661718	0.000326
H	-0.493954	-3.278214	-0.898055
H	-0.494057	-3.278043	0.898407
C	-0.409948	0.674305	0.000130
H	0.614398	1.043045	0.000195
H	-2.217465	-2.229505	-0.000125
H	-4.053647	-0.611120	-0.000249
C	-1.176023	3.074388	0.000144
H	-1.614195	3.550800	0.882005
H	-0.101812	3.270619	0.000186
H	-1.614139	3.550879	-0.881708

3OMe_SnArProduct_GEN
Electronic energy = -652.793830
Thermal correction to Gibbs free energy (25°C) = 0.048982
Thermal correction to Gibbs free energy (80°C) = 0.062752
qh-G(25°C) = -652.609632
qh-G(80°C) = -652.618555

Geometry:

C	-3.085027	-0.981323	1.283668
C	-2.024179	-0.091783	1.130979
C	-1.879974	0.588784	-0.077637
C	-2.777779	0.402037	-1.123052
C	-3.841317	-0.486292	-0.955529
C	-3.995637	-1.181290	0.243304
H	-3.203672	-1.514875	2.221294
H	-1.311876	0.076613	1.932874
H	-2.636693	0.950897	-2.048329
H	-4.546326	-0.635405	-1.767005
H	-4.821502	-1.873461	0.369321
O	-0.877663	1.526835	-0.244040

C 0.433166 1.142255 -0.084342
C 1.343948 2.162407 0.207746
C 2.688080 1.838098 0.331520
H 0.985605 3.177973 0.332259
C 2.213446 -0.475603 -0.119058
C 3.142726 0.524918 0.175255
H 3.405588 2.619541 0.560741
H 4.196608 0.303492 0.283863
C 0.852283 -0.170740 -0.254081
H 0.153823 -0.966247 -0.488757
O 2.532538 -1.784128 -0.299175
C 3.899107 -2.152507 -0.186438
H 4.281369 -1.937482 0.816798
H 3.936752 -3.225133 -0.369128
H 4.508260 -1.631337 -0.932223

3OMe_TMA_DMSO_tsopt_GEN_2
Electronic energy = -1073.313185
Thermal correction to Gibbs free energy (25°C) = 0.060381
Thermal correction to Gibbs free energy (80°C) = 0.078115
qh-G(25°C) = -1073.023926
qh-G(80°C) = -1073.034998

Geometry:
C 3.196469 -0.776996 1.174897
C 2.282602 -0.189538 2.051904
C 1.289109 0.671558 1.606634
C 1.193371 0.968570 0.235291
C 2.097747 0.389189 -0.652474
C 3.094146 -0.478544 -0.183570
H 3.960580 -1.442578 1.555424
H 2.350947 -0.411869 3.111849
H 0.605702 1.101075 2.327893
H 2.080206 0.588853 -1.715855
N 0.120887 1.794701 -0.240767
C 0.227753 2.220132 -1.642796
H -0.637768 2.839196 -1.880448
H 1.140793 2.800926 -1.813720
H 0.219652 1.352562 -2.304252
C -0.212066 2.934896 0.632177
H -1.028838 3.492612 0.172388
H -0.548137 2.590576 1.609325
H 0.653036 3.594520 0.756948
C -1.549097 0.559708 -0.198551
H -1.026348 -0.097763 -0.884457
H -1.394213 0.453945 0.869361
H -2.138216 1.382897 -0.588158
S -3.317970 -0.759400 -0.211217

O -4.006146 -0.843436 -1.538505
C -4.488267 -0.257107 1.047902
H -4.822192 0.749037 0.792391
H -5.323508 -0.959175 1.027297
H -3.989967 -0.263689 2.018555
C -2.847333 -2.398189 0.333873
H -3.748200 -3.012604 0.375945
H -2.146496 -2.790144 -0.403982
H -2.372425 -2.322337 1.313416
O 3.918540 -0.975411 -1.139669
C 4.954980 -1.855042 -0.726746
H 5.491762 -2.131455 -1.632518
H 5.638093 -1.355382 -0.032300
H 4.541200 -2.752752 -0.256128

3OMe_TMA_I_TS
Electronic energy = -531.757089
Thermal correction to Gibbs free energy (25°C) = 0.054527
Thermal correction to Gibbs free energy (80°C) = 0.069664
qh-G(25°C) = -531.548462
qh-G(80°C) = -531.558407

Geometry:
C -3.480739 -0.949395 1.126998
C -2.535322 -0.429480 2.015278
C -1.571809 0.480964 1.608109
C -1.536096 0.902648 0.266049
C -2.472138 0.391846 -0.631175
C -3.438214 -0.529209 -0.201379
H -4.219970 -1.657950 1.478096
H -2.553856 -0.750131 3.052061
H -0.855599 0.848716 2.332454
H -2.500919 0.686369 -1.672325
N -0.501839 1.793215 -0.166125
C -0.255531 2.927932 0.739422
H 0.575067 3.513301 0.341383
H -1.144452 3.564917 0.812844
H 0.020916 2.579201 1.733067
C -0.599314 2.247777 -1.556122
H 0.258736 2.886940 -1.768369
H -0.568447 1.394138 -2.235116
H -1.519333 2.819287 -1.728625
C 1.278454 0.650688 -0.126465
H 1.820063 1.521673 -0.464616
H 1.122704 0.485146 0.928704
H 0.830476 -0.020306 -0.844331
O -4.296945 -0.950075 -1.165469
C -5.297935 -1.886740 -0.795469

H -5.862741 -2.096058 -1.702525
H -4.848801 -2.812557 -0.421121
H -5.965856 -1.467201 -0.035852
I 3.448157 -0.741313 -0.075294

3OMe_TMA_SN2_tsopt_GEN
Electronic energy = -827.129914
Thermal correction to Gibbs free energy (25°C) = 0.061516
Thermal correction to Gibbs free energy (80°C) = 0.079692
qh-G(25°C) = -826.828859
qh-G(80°C) = -826.840046

Geometry:
C -3.694767 -1.534196 0.273216
C -3.858140 -0.231677 -0.195652
C -2.826375 0.709841 -0.063354
C -1.626449 0.351261 0.540569
C -1.450485 -0.959753 1.013070
C -2.481599 -1.877594 0.874502
H -4.479773 -2.273787 0.180696
H -3.016823 1.706854 -0.438634
H -0.524238 -1.272256 1.479480
H -2.341359 -2.889530 1.240121
N -0.524733 1.285477 0.635656
C 0.846950 0.652981 -0.533030
H 1.481616 1.503813 -0.317761
H 0.971370 -0.265163 0.025569
H 0.193883 0.669943 -1.395457
C 0.069401 1.345386 1.988628
H 0.915453 2.034148 1.961013
H -0.670134 1.700580 2.712269
H 0.432715 0.364358 2.289598
C -0.830575 2.646909 0.166259
H -1.114807 2.622135 -0.886576
H -1.632150 3.098229 0.758844
H 0.072023 3.250173 0.269520
C 3.325423 -0.235465 -1.018643
C 3.475670 -1.460016 -0.322430
C 4.365274 0.715847 -0.882408
C 4.609582 -1.721009 0.444239
H 2.686873 -2.203376 -0.413563
C 5.496823 0.444727 -0.116149
H 4.263678 1.664274 -1.404213
C 5.631746 -0.774339 0.554591
H 4.697691 -2.674050 0.959784
H 6.281627 1.193103 -0.039575
H 6.514810 -0.982106 1.150244
O 2.245994 0.021941 -1.732345

O -4.983923 0.231094 -0.794719
C -6.068297 -0.673416 -0.951169
H -6.421460 -1.031897 0.021141
H -6.859497 -0.110245 -1.442847
H -5.780032 -1.524749 -1.576178

3OMe_TMA_SnAr_tsopt_GEN
Electronic energy = -827.132005
Thermal correction to Gibbs free energy (25°C) = 0.058771
Thermal correction to Gibbs free energy (80°C) = 0.076510
qh-G(25°C) = -826.828257
qh-G(80°C) = -826.839040

Geometry:
C -1.689886 -1.688983 1.141372
C -0.825877 -0.890453 1.897983
C -0.299117 0.303116 1.435853
C -0.575064 0.753041 0.104680
C -1.512387 -0.024487 -0.636458
C -2.015117 -1.221059 -0.139601
H -2.086126 -2.611498 1.542700
H -0.552031 -1.216144 2.898469
H 0.396220 0.852563 2.059453
N -0.813563 2.315072 -0.009928
C -2.152673 2.600501 0.594212
H -2.301192 3.680889 0.599966
H -2.919003 2.110512 -0.004231
H -2.166698 2.204845 1.609039
C -0.829915 2.804242 -1.425120
H -1.121437 3.855083 -1.408743
H 0.161183 2.680131 -1.848572
H -1.565431 2.238632 -1.992971
C 0.213051 3.098228 0.746545
H 0.091212 2.912606 1.811723
H 1.198191 2.791933 0.404390
H 0.042050 4.156190 0.545478
O 0.935417 0.889186 -0.814673
C 1.895794 0.015350 -0.505306
C 1.653717 -1.365278 -0.353469
C 3.215154 0.479764 -0.337690
C 2.699719 -2.236293 -0.055444
H 0.641372 -1.741184 -0.467491
C 4.256556 -0.402232 -0.055147
C 4.007916 -1.768437 0.091905
H 2.489918 -3.296751 0.057381
H 5.266954 -0.018196 0.056387
H 4.817278 -2.455643 0.316753
H 3.404192 1.544491 -0.447141

H -1.789802 0.251634 -1.647031
O -2.847786 -1.890542 -0.995319
C -3.421465 -3.103875 -0.540242
H -4.048790 -3.465470 -1.354246
H -2.649146 -3.847776 -0.316926
H -4.037449 -2.940361 0.350604

3OMe_TMA_bromide_tsopt
Electronic energy = -3091.898482
Thermal correction to Gibbs free energy (25°C) = 0.053734
Thermal correction to Gibbs free energy (80°C) = 0.068726
qh-G(25°C) = -3091.688756
qh-G(80°C) = -3091.698569

Geometry:
C -3.008401 -0.897290 1.087791
C -1.995471 -0.538174 1.981086
C -0.956413 0.301516 1.608655
C -0.912746 0.813453 0.298978
C -1.914507 0.463402 -0.602805
C -2.956965 -0.388439 -0.208797
H -3.805106 -1.555502 1.409831
H -2.020857 -0.930769 2.992503
H -0.188515 0.543559 2.333267
H -1.937608 0.834857 -1.619118
N 0.197071 1.630438 -0.100193
C 0.526418 2.708484 0.848487
H 1.425955 3.216382 0.496099
H -0.295470 3.430528 0.912265
H 0.727361 2.305122 1.839230
C 0.133069 2.153671 -1.468757
H 1.043984 2.722588 -1.659971
H 0.082238 1.331299 -2.184396
H -0.731239 2.813883 -1.606535
C 1.853774 0.359717 -0.123406
H 2.444700 1.171203 -0.520410
H 1.756028 0.234161 0.943743
H 1.309597 -0.291984 -0.790116
O -3.876564 -0.653276 -1.171850
C -4.963662 -1.501295 -0.830894
H -5.575604 -1.579648 -1.727823
H -4.608288 -2.495330 -0.540461
H -5.556543 -1.069511 -0.017972
Br 3.735194 -1.077656 -0.163600

3OMe_TMA_chloride_tsopt
Electronic energy = -980.577315

Thermal correction to Gibbs free energy (25°C) = 0.052430
Thermal correction to Gibbs free energy (80°C) = 0.067176
qh-G(25°C) = -980.365863
qh-G(80°C) = -980.375457

Geometry:

C	-2.499142	-0.740154	1.067120
C	-1.434804	-0.589891	1.959790
C	-0.276245	0.087482	1.608876
C	-0.160529	0.644530	0.322441
C	-1.212647	0.501883	-0.578602
C	-2.375886	-0.188165	-0.206829
H	-3.389546	-1.274713	1.372280
H	-1.515677	-1.017931	2.953726
H	0.525535	0.169451	2.332291
H	-1.183926	0.917375	-1.577484
N	1.065137	1.287309	-0.059918
C	1.591084	2.230965	0.942880
H	2.547567	2.616120	0.585249
H	0.896340	3.065316	1.089383
H	1.759794	1.731431	1.895207
C	1.060251	1.905216	-1.390875
H	2.046724	2.334127	-1.571652
H	0.866497	1.150904	-2.155272
H	0.309401	2.700531	-1.461175
C	2.467785	-0.224136	-0.212730
H	3.189132	0.506775	-0.545980
H	2.347978	-0.425701	0.840593
H	1.823349	-0.714645	-0.926720
O	-3.332910	-0.258388	-1.167161
C	-4.536428	-0.943160	-0.850986
H	-5.158071	-0.884174	-1.742683
H	-4.337666	-1.992591	-0.610369
H	-5.050633	-0.463255	-0.011930
Cl	3.987640	-1.851206	-0.392662

3OMe_TMA_gen
Electronic energy = -520.277836
Thermal correction to Gibbs free energy (25°C) = 0.046148
Thermal correction to Gibbs free energy (80°C) = 0.059330
qh-G(25°C) = -520.059356
qh-G(80°C) = -520.067900

Geometry:

C	-0.654274	0.198760	-0.000012
C	0.405050	-0.691156	0.000016
C	1.718390	-0.193266	-0.000089
C	1.952208	1.181262	-0.000205

C 0.861146 2.051289 -0.000212
C -0.444631 1.580189 -0.000120
H 0.285819 -1.766055 0.000096
H 2.957909 1.581654 -0.000292
H 1.036482 3.121432 -0.000295
H -1.264602 2.287956 -0.000115
N -2.066413 -0.296235 0.000100
C -2.774224 0.206387 1.231628
H -2.794645 1.293264 1.216282
H -3.790210 -0.187004 1.222634
H -2.229798 -0.151383 2.104457
C -2.774231 0.205746 -1.231683
H -3.790211 -0.187651 -1.222486
H -2.794687 1.292631 -1.216913
H -2.229810 -0.152460 -2.104338
C -2.160013 -1.793355 0.000491
H -1.683567 -2.182148 -0.897765
H -1.683664 -2.181671 0.899002
H -3.217013 -2.053319 0.000511
O 2.685899 -1.136889 -0.000099
C 4.039807 -0.699543 0.000035
H 4.257800 -0.109970 0.895945
H 4.644479 -1.604247 0.000204
H 4.258043 -0.110149 -0.895935

3OMe_aniline_gen
Electronic energy = -480.551041
Thermal correction to Gibbs free energy (25°C) = 0.044834
Thermal correction to Gibbs free energy (80°C) = 0.057182
qh-G(25°C) = -480.375807
qh-G(80°C) = -480.384039

Geometry:
C -0.969175 0.135844 -0.092153
C 0.155315 -0.707573 -0.064073
C 1.448689 -0.179504 -0.017746
C 1.666485 1.199446 0.016241
C 0.544198 2.032850 0.007210
C -0.749423 1.534524 -0.040295
H 0.060527 -1.785971 -0.079026
H 2.661454 1.623059 0.055949
H 0.689486 3.108634 0.043302
H -1.581108 2.226822 -0.038314
N -2.249219 -0.384718 -0.180431
C -2.433412 -1.805994 0.054949
H -2.093706 -2.112764 1.055739
H -3.491878 -2.045819 -0.042679
H -1.889571 -2.395431 -0.688587

C -3.373341 0.487378 0.116763
H -4.296481 -0.082619 0.014864
H -3.326292 0.900629 1.135265
H -3.418661 1.319141 -0.591339
O 2.448671 -1.106523 -0.002775
C 3.784478 -0.633481 0.054573
H 3.958429 -0.054707 0.967989
H 4.418105 -1.519224 0.058531
H 4.021981 -0.017476 -0.819300

3OMe_bromide_ionpair1_gen
Electronic energy = -3091.951879
Thermal correction to Gibbs free energy (25°C) = 0.052750
Thermal correction to Gibbs free energy (80°C) = 0.067444
qh-G(25°C) = -3091.737115
qh-G(80°C) = -3091.746674

Geometry:
Br 3.011210 0.889886 0.000108
N -0.088452 -2.124542 -0.000126
C -1.140397 -1.060539 0.000008
C -2.495262 -1.384045 0.000210
C -3.418333 -0.339912 0.000336
C -3.017648 0.992817 0.000240
C -1.650431 1.288368 0.000015
C 0.773525 -1.979213 1.228387
H 1.489755 -2.801004 1.232586
H 0.126360 -2.027802 2.103352
H 1.302499 -1.027604 1.178103
C 0.773614 -1.978891 -1.228539
H 1.489806 -2.800711 -1.232956
H 1.302660 -1.027356 -1.177960
H 0.126514 -2.027185 -2.103569
C -0.665503 -3.507291 -0.000340
H 0.168708 -4.206719 -0.000472
H -1.264713 -3.647176 -0.898891
H -1.264593 -3.647532 0.898241
C -0.705790 0.257141 -0.000038
H 0.347685 0.528633 -0.000033
H -2.853848 -2.403313 0.000248
H -4.477045 -0.575388 0.000534
H -3.763798 1.777363 0.000359
O -1.139344 2.541242 -0.000131
C -2.051729 3.631741 -0.000319
H -2.680457 3.614625 0.895592
H -1.439742 4.531711 -0.000555
H -2.680520 3.614261 -0.896183

3OMe_bromide_ionpair2_gen
Electronic energy = -3091.951899
Thermal correction to Gibbs free energy (25°C) = 0.052737
Thermal correction to Gibbs free energy (80°C) = 0.067454
qh-G(25°C) = -3091.737182
qh-G(80°C) = -3091.746760

Geometry:
Br -3.610077 -0.951578 0.000095
N -0.199287 1.719422 -0.000006
C 0.732850 0.549919 -0.000235
C 2.104088 0.737614 -0.000126
C 2.947476 -0.385048 -0.000137
C 2.410082 -1.671563 -0.000229
C 1.022077 -1.822224 -0.000329
C -1.071533 1.662497 1.228503
H -1.704616 2.549972 1.228573
H -0.423175 1.651488 2.104029
H -1.690736 0.766915 1.180797
C -1.072711 1.662146 -1.227685
H -1.705569 2.549773 -1.227526
H -1.692091 0.766730 -1.179008
H -0.425174 1.650622 -2.103813
C 0.516036 3.035333 -0.000577
H -0.241356 3.817236 -0.000615
H 1.126401 3.112560 -0.899004
H 1.126839 3.113120 0.897490
C 0.168068 -0.727501 -0.000332
H -0.907827 -0.878841 -0.000470
H 2.577103 1.710313 0.000012
H 3.045896 -2.547850 -0.000281
H 0.598466 -2.820949 -0.000416
O 4.271554 -0.104793 -0.000136
C 5.179965 -1.198864 0.000492
H 5.048997 -1.814115 -0.895222
H 6.175609 -0.759418 0.000902
H 5.048135 -1.813803 0.896288

3OMe_chloride_ionpair1_gen
Electronic energy = -980.629873
Thermal correction to Gibbs free energy (25°C) = 0.051742
Thermal correction to Gibbs free energy (80°C) = 0.066277
qh-G(25°C) = -980.414035
qh-G(80°C) = -980.423445

Geometry:
Cl 1.295360 3.294950 0.000030

N 1.824768 -0.840475 -0.000012
C 0.341098 -1.034614 -0.000027
C -0.219795 -2.309722 0.000018
C -1.609417 -2.414394 0.000046
C -2.429122 -1.289588 0.000011
C -1.838647 -0.021637 -0.000049
C 2.231188 -0.065763 1.227645
H 3.318607 0.011300 1.227391
H 1.883981 -0.613456 2.103025
H 1.789641 0.929532 1.177873
C 2.231231 -0.065679 -1.227587
H 3.318649 0.011395 -1.227278
H 1.789690 0.929607 -1.177777
H 1.884069 -0.613311 -2.103024
C 2.574554 -2.137104 -0.000052
H 3.636882 -1.899707 -0.000064
H 2.321100 -2.697987 -0.898240
H 2.321138 -2.698022 0.898124
C -0.445852 0.108376 -0.000054
H -0.022565 1.111137 -0.000035
H 0.375969 -3.211096 0.000034
H -2.061700 -3.400157 0.000095
H -3.505059 -1.410069 0.000044
O -2.529157 1.142231 -0.000075
C -3.949259 1.072843 0.000025
H -4.314896 0.561014 0.895869
H -4.297601 2.103925 -0.000006
H -4.315010 0.560921 -0.895719

3OMe_chloride_ionpair2_gen
Electronic energy = -980.629923
Thermal correction to Gibbs free energy (25°C) = 0.051823
Thermal correction to Gibbs free energy (80°C) = 0.066391
qh-G(25°C) = -980.414378
qh-G(80°C) = -980.423808

Geometry:
Cl -3.882265 -1.681526 0.000316
N -1.061140 1.390967 -0.000063
C 0.048046 0.388032 -0.000158
C 1.370236 0.796943 0.000076
C 2.385457 -0.172932 0.000002
C 2.064748 -1.529775 -0.000254
C 0.719881 -1.905090 -0.000507
C -1.913474 1.192565 1.228070
H -2.676790 1.970970 1.232479
H -1.271077 1.278542 2.103743
H -2.385260 0.211416 1.174130

C -1.914062 1.192307 -1.227732
H -2.677479 1.970611 -1.231823
H -2.385726 0.211107 -1.173499
H -1.272107 1.278328 -2.103731
C -0.568450 2.805456 -0.000419
H -1.443045 3.453387 -0.000658
H 0.021235 2.981487 -0.899003
H 0.021044 2.982065 0.898161
C -0.301556 -0.964381 -0.000489
H -1.339572 -1.286999 -0.000549
H 1.677297 1.833982 0.000272
H 2.835015 -2.290614 -0.000277
H 0.465448 -2.959662 -0.000723
O 3.646206 0.319624 0.000307
C 4.721143 -0.611197 0.000287
H 4.692324 -1.239480 -0.895582
H 5.631523 -0.014820 0.000417
H 4.692183 -1.239699 0.895996

4Br_SnArProduct_GEN
Electronic energy = -3109.191413
Thermal correction to Gibbs free energy (25°C) = 0.047523
Thermal correction to Gibbs free energy (80°C) = 0.060509
qh-G(25°C) = -3109.049730
qh-G(80°C) = -3109.058368

Geometry:
C -4.038420 -1.262084 1.058016
C -2.919064 -0.432773 1.053583
C -2.787594 0.519005 0.043417
C -3.752567 0.661039 -0.947538
C -4.873313 -0.170437 -0.927657
C -5.017346 -1.134041 0.069916
H -4.148464 -2.005887 1.840597
H -2.153850 -0.518028 1.819155
H -3.617807 1.415108 -1.715909
H -5.631172 -0.064397 -1.697147
H -5.888215 -1.780928 0.080525
O -1.722032 1.402342 0.038829
C -0.443594 0.903098 0.024454
C 0.555663 1.747951 0.508012
C 1.884218 1.337206 0.478816
H 0.282575 2.720132 0.903861
C 2.198115 0.081493 -0.033833
H 2.664852 1.988664 0.855212
C -0.123870 -0.351641 -0.495043
H -0.899732 -1.004676 -0.879142
C 1.207115 -0.764058 -0.519272

H 1.464372 -1.737349 -0.921865
Br 4.010504 -0.486855 -0.070661

4Br_TMA_DMSO_tsopt_GEN
Electronic energy = -3529.710083
Thermal correction to Gibbs free energy (25°C) = 0.061287
Thermal correction to Gibbs free energy (80°C) = 0.078745
qh-G(25°C) = -3529.464917
qh-G(80°C) = -3529.476025

Geometry:
C -2.795414 -0.086536 -0.002662
C -2.404129 0.653312 -1.109374
C -1.208298 1.369684 -1.072651
C -0.399654 1.347216 0.068908
C -0.814747 0.590724 1.175078
C -2.006393 -0.123161 1.142788
H -3.022992 0.683978 -1.998921
H -0.935920 1.947034 -1.946382
H -0.217687 0.542216 2.078255
H -2.313624 -0.702777 2.005702
N 0.866624 2.021056 0.111593
C 1.112548 2.771013 1.358620
H 2.109301 3.211457 1.305635
H 0.370159 3.566713 1.479823
H 1.077722 2.111022 2.223833
C 1.172029 2.853963 -1.059475
H 2.163414 3.287416 -0.921930
H 1.183766 2.243996 -1.964228
H 0.444213 3.664944 -1.173087
C 2.301072 0.518902 0.053164
H 1.896764 0.131867 0.981016
H 1.876005 0.204670 -0.892849
H 3.064190 1.288044 0.082433
S 3.858019 -1.052870 0.047378
O 4.787677 -0.963751 1.218187
C 4.798390 -1.015142 -1.476385
H 5.300314 -0.048117 -1.518385
H 5.526147 -1.827701 -1.441170
H 4.114326 -1.131318 -2.318543
C 3.106803 -2.677424 -0.006113
H 3.909007 -3.416959 -0.036155
H 2.514919 -2.786349 0.903213
H 2.471062 -2.746107 -0.890326
Br -4.422869 -1.058023 -0.047829

4Br_TMA_I_TS

Electronic energy = -2988.154414
Thermal correction to Gibbs free energy (25°C) = 0.053061
Thermal correction to Gibbs free energy (80°C) = 0.067402
qh-G(25°C) = -2987.987795
qh-G(80°C) = -2987.997456

Geometry:
C 3.070297 -0.152018 0.004236
C 2.259326 -0.173666 1.135209
C 1.106945 0.601266 1.165074
C 0.751589 1.406751 0.072026
C 1.581214 1.411777 -1.055311
C 2.737771 0.633213 -1.090178
H 2.519246 -0.790721 1.987796
H 0.487543 0.560314 2.053460
H 1.352656 2.021606 -1.919780
H 3.371614 0.649742 -1.969458
N -0.473831 2.144228 0.110844
C -0.730314 2.999423 -1.052557
H -1.704155 3.472375 -0.920029
H 0.032026 3.781004 -1.153309
H -0.760129 2.397851 -1.962518
C -0.702931 2.884257 1.364157
H -1.685970 3.354965 1.311178
H -0.694235 2.212060 2.220383
H 0.062433 3.656098 1.502129
C -2.000868 0.696924 0.024896
I -3.862614 -1.095934 -0.078480
H -2.735399 1.489431 0.021908
H -1.582380 0.343094 -0.905600
H -1.646690 0.290618 0.959966
Br 4.645161 -1.206032 -0.035655

4Br_TMA_SN2_tsopt_GEN
Electronic energy = -3283.527498
Thermal correction to Gibbs free energy (25°C) = 0.059842
Thermal correction to Gibbs free energy (80°C) = 0.077216
qh-G(25°C) = -3283.268605
qh-G(80°C) = -3283.279488

Geometry:
C 2.687824 -0.294907 0.037401
C 1.661849 -0.420314 0.969048
C 0.687778 0.567846 1.047817
C 0.731184 1.683327 0.200694
C 1.768770 1.790917 -0.727649
C 2.748924 0.802146 -0.809874
H -0.105841 0.444931 1.775783

H 1.843410 2.637727 -1.397025
H 3.551739 0.897741 -1.531825
N -0.340189 2.653526 0.261817
C -1.903726 1.753982 -0.332192
H -2.513881 2.646371 -0.295055
H -1.441573 1.463028 -1.264689
H -1.892934 1.071124 0.507020
C -0.180401 3.800444 -0.649046
H -1.050319 4.446988 -0.530802
H 0.722837 4.370291 -0.411657
H -0.142153 3.452158 -1.682140
C -0.609111 3.138981 1.633751
H -0.852487 2.307080 2.292238
H 0.262741 3.672085 2.023477
H -1.465784 3.813594 1.595179
C -3.631726 -0.403674 -0.523232
C -2.520498 -1.287296 -0.533857
C -4.863830 -0.932787 -0.058931
C -2.644138 -2.608888 -0.106280
H -1.562013 -0.928655 -0.900141
C -4.975673 -2.255694 0.355275
H -5.726497 -0.271835 -0.040289
C -3.867477 -3.110327 0.342004
H -1.771040 -3.256579 -0.131583
H -5.938742 -2.625550 0.698650
H -3.957917 -4.140631 0.670253
O -3.544215 0.848508 -0.914712
H 1.616059 -1.280165 1.627636
Br 4.017741 -1.640713 -0.073989

4Br_TMA_SnAr_tsopt_GEN
Electronic energy = -3283.532462
Thermal correction to Gibbs free energy (25°C) = 0.057631
Thermal correction to Gibbs free energy (80°C) = 0.074649
qh-G(25°C) = -3283.271506
qh-G(80°C) = -3283.282058

Geometry:
C 2.003119 -0.272941 0.030866
C 1.165426 -0.124195 1.128785
C -0.151215 -0.562679 1.082388
C -0.713476 -1.116105 -0.108354
C 0.206839 -1.329980 -1.186957
C 1.515707 -0.888444 -1.122793
H 1.532618 0.337321 2.040401
H -0.777582 -0.396386 1.950962
H 2.161729 -1.026544 -1.984347
N -1.703228 -2.314072 0.139210

C	-0.880380	-3.488688	0.571414
H	-1.557233	-4.313530	0.796482
H	-0.203083	-3.759353	-0.237392
H	-0.308797	-3.208190	1.455024
C	-2.463866	-2.710789	-1.090127
H	-3.054063	-3.592590	-0.839815
H	-3.100687	-1.884431	-1.387733
H	-1.762909	-2.965751	-1.881826
C	-2.690982	-2.013303	1.224126
H	-2.166954	-1.926809	2.173696
H	-3.205318	-1.089814	0.971843
H	-3.390910	-2.847305	1.277592
O	-2.035413	-0.087852	-0.708831
C	-1.937567	1.213186	-0.420077
C	-0.767614	1.959167	-0.666252
C	-3.044501	1.879491	0.140097
C	-0.722225	3.320246	-0.371012
H	0.099798	1.459941	-1.088398
C	-2.994936	3.244666	0.416345
C	-1.832269	3.976464	0.166213
H	0.190315	3.875959	-0.569778
H	-3.868265	3.737556	0.834675
H	-1.791312	5.038459	0.385983
H	-3.946827	1.306576	0.337139
H	-0.140631	-1.776375	-2.111083
Br	3.809818	0.326172	0.108708

4Br_TMA_bromide_tsopt
Electronic energy = -5548.295693
Thermal correction to Gibbs free energy (25°C) = 0.052419
Thermal correction to Gibbs free energy (80°C) = 0.066634
qh-G(25°C) = -5548.128477
qh-G(80°C) = -5548.138019

Geometry:

C	2.579770	-0.065458	0.006707
C	1.772428	-0.188196	1.133367
C	0.541672	0.455720	1.165951
C	0.104956	1.229208	0.079606
C	0.932816	1.337335	-1.043289
C	2.167777	0.690585	-1.081077
H	2.094884	-0.782798	1.980485
H	-0.071842	0.337205	2.051439
H	0.642859	1.928282	-1.902080
H	2.799754	0.787077	-1.956639
N	-1.194758	1.832032	0.120511
C	-1.529204	2.675485	-1.033611
H	-2.545686	3.047781	-0.900684

H -0.847264 3.529549 -1.117095
H -1.494788 2.088109 -1.952563
C -1.496200 2.537332 1.379967
H -2.521253 2.907942 1.330614
H -1.419449 1.863364 2.231420
H -0.811786 3.380757 1.522412
C -2.553766 0.262496 0.008580
H -3.359016 0.981232 -0.001235
H -2.091025 -0.046914 -0.916289
H -2.180657 -0.118417 0.946636
Br 4.260265 -0.943623 -0.039966
Br -4.116062 -1.523075 -0.127383

4Br_TMA_chloride_tsopt
Electronic energy = -3436.974484
Thermal correction to Gibbs free energy (25°C) = 0.051258
Thermal correction to Gibbs free energy (80°C) = 0.065258
qh-G(25°C) = -3436.805801
qh-G(80°C) = -3436.815148

Geometry:
C -2.001367 -0.020218 0.010247
C -1.212663 0.281150 1.116356
C 0.105980 -0.155423 1.154732
C 0.649658 -0.896552 0.094753
C -0.161120 -1.186005 -1.007936
C -1.484464 -0.748451 -1.051364
H -1.617518 0.854092 1.942804
H 0.701607 0.099119 2.023629
H 0.211271 -1.760062 -1.846075
H -2.101660 -0.984306 -1.910812
N 2.031549 -1.279756 0.137487
C 2.481951 -2.111684 -0.985718
H 3.545345 -2.314513 -0.853708
H 1.941354 -3.064271 -1.020802
H 2.347171 -1.577990 -1.928022
C 2.452679 -1.876281 1.419184
H 3.525186 -2.071382 1.372011
H 2.267213 -1.192031 2.245256
H 1.919723 -2.816050 1.599389
C 3.113692 0.462118 -0.062778
H 4.020259 -0.123617 -0.089317
H 2.574556 0.670583 -0.974631
H 2.721397 0.808347 0.881013
Br -3.802469 0.571873 -0.045849
Cl 4.302564 2.348406 -0.289605

4Br_TMA_gen
Electronic energy = -2976.673296
Thermal correction to Gibbs free energy (25°C) = 0.045282
Thermal correction to Gibbs free energy (80°C) = 0.057759
qh-G(25°C) = -2976.498154
qh-G(80°C) = -2976.506479

Geometry:

C	-1.395866	0.032751	-0.004191
C	-0.700683	1.235974	-0.004100
C	0.694184	1.223115	-0.002748
C	1.369190	0.010647	-0.001745
C	0.675817	-1.196612	-0.002193
C	-0.713171	-1.183379	-0.003025
H	-1.200462	2.194740	-0.004561
H	1.207713	-2.140566	-0.001450
H	-1.235090	-2.133405	-0.002155
N	-2.889120	-0.000899	0.000362
C	-3.383465	-0.739399	-1.217309
H	-3.032010	-1.767895	-1.183924
H	-4.472479	-0.718381	-1.204416
H	-2.996565	-0.233497	-2.100781
C	-3.371654	-0.697871	1.246788
H	-4.460893	-0.691629	1.236366
H	-3.004126	-1.721288	1.251740
H	-2.989266	-0.152880	2.108817
C	-3.499884	1.370307	-0.020499
H	-3.192603	1.913681	0.871328
H	-3.188925	1.888214	-0.926261
H	-4.581067	1.243417	-0.020315
H	1.239225	2.159527	-0.002085
Br	3.259531	-0.004769	0.000837

4Br_aniline_gen
Electronic energy = -2936.949859
Thermal correction to Gibbs free energy (25°C) = 0.043720
Thermal correction to Gibbs free energy (80°C) = 0.055331
qh-G(25°C) = -2936.817380
qh-G(80°C) = -2936.825370

Geometry:

C	1.737152	0.000008	-0.081483
C	1.003108	1.207891	-0.055110
C	-0.387075	1.205203	-0.027953
C	-1.079752	-0.000002	-0.019289
C	-0.387057	-1.205190	-0.027629
C	1.003134	-1.207871	-0.054786
H	1.512166	2.163364	-0.054827

H -0.924715 -2.147243 -0.009334
H 1.512194 -2.163344 -0.054180
N 3.114375 0.000032 -0.142028
C 3.825244 -1.245915 0.085825
H 3.564743 -1.987801 -0.674953
H 4.896002 -1.059397 0.013582
H 3.609908 -1.673764 1.075791
C 3.825222 1.245898 0.086417
H 3.565513 1.987859 -0.674568
H 3.609067 1.673742 1.076198
H 4.896017 1.059223 0.015156
H -0.924754 2.147248 -0.009945
Br -2.981618 -0.000007 0.024438

4Br_bromide_ionpair_gen
Electronic energy = -5548.348274
Thermal correction to Gibbs free energy (25°C) = 0.051546
Thermal correction to Gibbs free energy (80°C) = 0.065506
qh-G(25°C) = -5548.176474
qh-G(80°C) = -5548.185791

Geometry:
Br -3.381939 -1.896287 -0.000030
N -1.454551 1.982170 0.000012
C -0.123216 1.306397 0.000015
C 1.060051 2.035436 -0.000085
C 2.280610 1.359391 -0.000083
C 2.294393 -0.028372 0.000044
C 1.109448 -0.758897 0.000123
C -2.226193 1.569933 1.229064
H -3.167462 2.119539 1.231796
H -1.629661 1.823097 2.104745
H -2.423843 0.499054 1.178749
C -2.226395 1.569558 -1.228789
H -3.167615 2.119242 -1.231600
H -2.424124 0.498709 -1.178078
H -1.629970 1.822369 -2.104647
C -1.342345 3.476355 -0.000222
H -2.353623 3.879077 -0.000223
H -0.817478 3.797193 -0.898792
H -0.817389 3.797479 0.898190
C -0.107117 -0.087619 0.000137
H -1.026412 -0.669485 0.000229
H 1.076486 3.116730 -0.000185
H 3.206567 1.922132 -0.000173
H 1.128183 -1.842621 0.000185
Br 3.950876 -0.944021 -0.000013

4Br_chloride_ionpair_gen
Electronic energy = -3437.026400
Thermal correction to Gibbs free energy (25°C) = 0.050591
Thermal correction to Gibbs free energy (80°C) = 0.064387
qh-G(25°C) = -3436.853488
qh-G(80°C) = -3436.862652

Geometry:

Cl	3.267451	2.831098	0.000055
N	2.273513	-1.211980	-0.000029
C	0.825037	-0.849884	-0.000036
C	-0.164972	-1.825279	0.000088
C	-1.505841	-1.439769	0.000111
C	-1.829633	-0.090288	0.000019
C	-0.838467	0.887319	-0.000096
C	2.933108	-0.638194	1.229211
H	3.974181	-0.961046	1.229657
H	2.410568	-1.023683	2.104017
H	2.883240	0.449641	1.179695
C	2.933181	-0.638157	-1.229192
H	3.974250	-0.961021	-1.229585
H	2.883366	0.449677	-1.179644
H	2.410706	-1.023613	-2.104053
C	2.498575	-2.693030	-0.000068
H	3.574394	-2.859214	-0.000138
H	2.059072	-3.122926	-0.898803
H	2.059246	-3.122951	0.898734
C	0.497908	0.505797	-0.000107
H	1.266363	1.276882	-0.000105
H	0.061065	-2.882814	0.000144
H	-2.282479	-2.195367	0.000201
H	-1.100043	1.939145	-0.000166
Br	-3.649388	0.430897	-0.000008

4Bz_SnArProduct_GEN
Electronic energy = -882.574521
Thermal correction to Gibbs free energy (25°C) = 0.057563
Thermal correction to Gibbs free energy (80°C) = 0.074388
qh-G(25°C) = -882.336307
qh-G(80°C) = -882.346717

Geometry:

C	5.456358	-0.481656	1.424901
C	4.263648	-1.007701	0.929138
C	3.805110	-0.579753	-0.312232
C	4.505199	0.354272	-1.068256
C	5.696964	0.873412	-0.562570

C	6.172591	0.458126	0.682069
H	5.825604	-0.808743	2.391446
H	3.689469	-1.739642	1.488288
H	4.115347	0.662123	-2.033283
H	6.253469	1.601609	-1.143557
H	7.100616	0.863886	1.070982
O	2.643917	-1.137048	-0.831691
C	1.449321	-0.536230	-0.554399
C	0.318307	-1.153343	-1.098456
C	-0.938478	-0.619222	-0.856487
H	0.445276	-2.044058	-1.704074
C	-1.082071	0.544762	-0.086213
H	-1.809702	-1.098511	-1.291485
C	1.327926	0.624149	0.216671
H	2.204075	1.102371	0.639005
C	0.063050	1.158603	0.432317
H	-0.047053	2.064640	1.019654
C	-2.402570	1.193735	0.146175
O	-2.474620	2.401693	0.336810
C	-3.649677	0.365562	0.148120
C	-3.668787	-0.943922	0.642610
C	-4.837877	0.955332	-0.299745
C	-4.867056	-1.654826	0.686768
H	-2.756074	-1.399420	1.014550
C	-6.028641	0.236062	-0.275808
H	-4.812283	1.975300	-0.670319
C	-6.043996	-1.069805	0.219852
H	-4.880762	-2.664207	1.084649
H	-6.944398	0.691200	-0.638786
H	-6.973902	-1.629042	0.245257

4Bz_TMA_DMSO_tsopt_GEN
Electronic energy = -1303.092173
Thermal correction to Gibbs free energy (25°C) = 0.071157
Thermal correction to Gibbs free energy (80°C) = 0.092444
qh-G(25°C) = -1302.750132
qh-G(80°C) = -1302.763012

Geometry:

C	-1.863557	-0.895581	-0.577864
C	-1.572904	-0.974098	0.785115
C	-0.305878	-1.352631	1.223772
C	0.702638	-1.646353	0.298780
C	0.411182	-1.565284	-1.074022
C	-0.857531	-1.206749	-1.500444
H	-2.341860	-0.768394	1.522791
H	-0.130528	-1.426078	2.288760
H	1.167147	-1.780598	-1.820011

H -1.078816 -1.156532 -2.561530
N 2.035769 -1.962607 0.716388
C 2.651877 -3.090385 -0.010876
H 3.669573 -3.223362 0.358339
H 2.082090 -4.009713 0.157819
H 2.700785 -2.886009 -1.078878
C 2.222231 -2.119131 2.165861
H 3.278057 -2.316331 2.354539
H 1.944425 -1.200212 2.684001
H 1.629470 -2.954269 2.554653
C 3.172535 -0.298066 0.237687
H 2.894773 -0.436700 -0.800641
H 2.537522 0.285979 0.893802
H 4.042435 -0.810104 0.633320
S 4.417362 1.448155 -0.344440
O 5.431707 1.124104 -1.398175
C 5.230001 2.182083 1.072779
H 5.911286 1.431550 1.474541
H 5.779619 3.059147 0.726780
H 4.474472 2.453419 1.811868
C 3.363836 2.781278 -0.910610
H 4.004991 3.620146 -1.187308
H 2.814626 2.411622 -1.777332
H 2.677721 3.055508 -0.107689
C -3.226794 -0.565580 -1.093090
O -3.600658 -1.021074 -2.165232
C -4.130892 0.318048 -0.295072
C -3.637897 1.365325 0.492393
C -5.512927 0.118309 -0.399764
C -4.522374 2.203482 1.169182
H -2.568777 1.542327 0.558366
C -6.392742 0.942666 0.294121
H -5.882294 -0.690681 -1.022156
C -5.897294 1.987546 1.077889
H -4.137995 3.023272 1.767009
H -7.462498 0.775353 0.223359
H -6.583848 2.634926 1.614156

4Bz_TMA_I_TS
Electronic energy = -761.536720
Thermal correction to Gibbs free energy (25°C) = 0.063371
Thermal correction to Gibbs free energy (80°C) = 0.081616
qh-G(25°C) = -761.274152
qh-G(80°C) = -761.285640

Geometry:
C 2.219585 0.969343 0.546917
C 1.860523 0.896298 -0.800929

C 0.603010 1.311513 -1.228722
C -0.334947 1.798180 -0.307449
C 0.025137 1.868856 1.050016
C 1.286915 1.470259 1.462734
H 2.571838 0.540734 -1.539538
H 0.378281 1.261719 -2.285790
H -0.670366 2.238194 1.793066
H 1.559997 1.539499 2.510838
N -1.652657 2.158201 -0.715757
C -1.839712 2.330321 -2.163861
H -2.876409 2.615139 -2.343038
H -1.178369 3.109049 -2.559685
H -1.652057 1.390565 -2.684606
C -2.259226 3.269744 0.039182
H -3.242958 3.473798 -0.384118
H -2.395159 3.000504 1.085887
H -1.639406 4.170003 -0.028953
C -2.843056 0.504900 -0.262263
H -3.695709 1.023197 -0.675220
H -2.222574 -0.110022 -0.897381
H -2.598058 0.611991 0.784410
C 3.581540 0.605818 1.036566
O 4.037806 1.140525 2.038529
C 4.392328 -0.409619 0.295697
C 5.785205 -0.268572 0.291945
C 3.804604 -1.514537 -0.331273
C 6.581746 -1.209506 -0.352592
H 6.229746 0.587401 0.790166
C 4.606210 -2.468012 -0.956552
H 2.726688 -1.644130 -0.311465
C 5.992047 -2.311665 -0.975749
H 7.659962 -1.087625 -0.367788
H 4.148322 -3.331007 -1.428757
H 6.613500 -3.049769 -1.473168
I -4.362690 -1.540203 0.276514

4Bz_TMA_SN2_tsopt_GEN
Electronic energy = -1056.909669
Thermal correction to Gibbs free energy (25°C) = 0.069618
Thermal correction to Gibbs free energy (80°C) = 0.090802
qh-G(25°C) = -1056.554009
qh-G(80°C) = -1056.566627

Geometry:
C -1.746638 -1.609626 -0.144613
C -0.990611 -0.980941 0.850356
C 0.339824 -1.325874 1.048906
C 0.948974 -2.293438 0.238298

C	0.194858	-2.926823	-0.755413
C	-1.144659	-2.593911	-0.929869
H	0.892231	-0.820396	1.831971
H	0.628342	-3.684013	-1.395035
H	-1.731165	-3.096907	-1.691820
N	2.363343	-2.546333	0.393585
C	3.219365	-0.959675	-0.187437
H	4.217105	-1.361979	-0.073126
H	2.741850	-0.993494	-1.156072
H	2.780985	-0.368783	0.605637
C	2.895816	-3.621670	-0.462739
H	3.966403	-3.703048	-0.273194
H	2.417592	-4.578824	-0.235267
H	2.746469	-3.369751	-1.513280
C	2.770781	-2.776719	1.798452
H	2.511997	-1.920188	2.417693
H	2.284143	-3.674314	2.189943
H	3.853571	-2.905979	1.822058
C	3.451980	1.793030	-0.479203
C	4.125028	2.958379	-0.027466
C	2.039819	1.881449	-0.604899
C	3.434627	4.129410	0.265368
H	5.205806	2.912143	0.078504
C	1.359085	3.059368	-0.297972
H	1.483123	1.020298	-0.965814
C	2.042446	4.195424	0.138683
H	3.988162	5.002582	0.602055
H	0.277909	3.090110	-0.410621
H	1.506714	5.109732	0.372014
O	4.122330	0.697301	-0.757639
H	-1.440418	-0.227935	1.489425
C	-3.195845	-1.306817	-0.357149
O	-3.953165	-2.184025	-0.747304
C	-3.706549	0.072776	-0.092466
C	-2.910898	1.206201	-0.299207
C	-5.037892	0.222545	0.314647
C	-3.444960	2.477798	-0.098322
H	-1.884987	1.098401	-0.638612
C	-5.562095	1.492186	0.535243
H	-5.646605	-0.663985	0.462140
C	-4.765772	2.620857	0.326724
H	-2.830241	3.354699	-0.273003
H	-6.589318	1.604508	0.866240
H	-5.176505	3.611668	0.493079

4Bz_TMA_SnAr_tsopt_GEN
Electronic energy = -1056.924587
Thermal correction to Gibbs free energy (25°C) = 0.068020

Thermal correction to Gibbs free energy (80°C) = 0.088893
qh-G(25°C) = -1056.566999
qh-G(80°C) = -1056.579369

Geometry:

C	-0.768311	-0.428379	0.845877
C	-0.419125	-0.734750	-0.484887
C	0.883476	-0.984708	-0.860257
C	1.955262	-0.849287	0.075627
C	1.585694	-0.658973	1.446072
C	0.281188	-0.398224	1.789647
H	-1.183928	-0.761740	-1.255391
H	1.096865	-1.177916	-1.904288
H	2.348597	-0.621478	2.214219
H	0.039630	-0.181891	2.826434
N	3.164657	-1.770502	-0.164463
C	2.752226	-3.163642	0.214777
H	3.588697	-3.832193	0.009562
H	2.500380	-3.178335	1.274187
H	1.882743	-3.442509	-0.378524
C	4.358157	-1.396727	0.667261
H	5.146689	-2.114967	0.444856
H	4.656634	-0.386445	0.406504
H	4.100608	-1.466824	1.721221
C	3.588981	-1.784936	-1.605175
H	2.819898	-2.271639	-2.200692
H	3.744055	-0.757662	-1.922609
H	4.510453	-2.362494	-1.671079
O	2.904739	0.658690	-0.411764
C	2.110492	1.725646	-0.426472
C	1.923449	2.505128	0.733978
C	1.411768	2.093435	-1.595045
C	1.071438	3.607184	0.721744
H	2.461760	2.225266	1.634911
C	0.562269	3.197567	-1.599992
H	1.555197	1.495960	-2.491044
C	0.381313	3.958294	-0.441864
H	0.942405	4.195723	1.626045
H	0.035240	3.465390	-2.511764
H	-0.281916	4.817592	-0.448023
C	-2.123752	-0.092620	1.276757
O	-2.346760	0.465794	2.356023
C	-3.295764	-0.422538	0.393908
C	-3.414729	-1.657933	-0.252446
C	-4.329589	0.514069	0.283108
C	-4.552465	-1.948867	-1.004702
H	-2.627927	-2.399718	-0.150210
C	-5.455544	0.232100	-0.486304
H	-4.237145	1.463699	0.801509

C -5.568977 -1.001816 -1.130252
H -4.644646 -2.914352 -1.491861
H -6.245919 0.969995 -0.580885
H -6.449550 -1.225965 -1.724064

4Bz_TMA_bromide_tsopt
Electronic energy = -3321.677988
Thermal correction to Gibbs free energy (25°C) = 0.062546
Thermal correction to Gibbs free energy (80°C) = 0.080637
qh-G(25°C) = -3321.414213
qh-G(80°C) = -3321.425564

Geometry:
C 1.615673 -0.929779 -0.536070
C 1.281391 -0.873187 0.818352
C -0.012299 -1.159688 1.246734
C -1.007927 -1.492553 0.319366
C -0.671793 -1.547629 -1.044857
C 0.623786 -1.282498 -1.458872
H 2.036299 -0.631789 1.559836
H -0.220871 -1.128926 2.307691
H -1.414396 -1.794770 -1.793560
H 0.877344 -1.337313 -2.512589
N -2.362262 -1.704810 0.723457
C -2.579185 -1.811284 2.173363
H -3.645833 -1.953667 2.348556
H -2.030435 -2.660138 2.596010
H -2.270981 -0.891049 2.671021
C -3.056863 -2.792598 0.008506
H -4.072959 -2.863245 0.397826
H -3.119215 -2.580857 -1.057608
H -2.541284 -3.746170 0.162366
C -3.358551 0.022978 0.186748
H -4.272760 -0.386943 0.589190
H -2.695737 0.593582 0.819744
H -3.095561 -0.149785 -0.845713
C 3.004464 -0.699229 -1.033050
O 3.394004 -1.264893 -2.045957
C 3.917321 0.223545 -0.290307
C 5.293567 -0.031825 -0.331445
C 3.440808 1.356161 0.380546
C 6.182931 0.823577 0.310866
H 5.650666 -0.907416 -0.864436
C 4.335829 2.223582 1.004754
H 2.377528 1.575089 0.395174
C 5.703935 1.953542 0.978095
H 7.247423 0.614155 0.290113
H 3.964718 3.108402 1.511283

H 6.398065 2.624913 1.473643
Br -4.516192 2.019145 -0.432613

4Bz_TMA_chloride_tsopt
Electronic energy = -1210.356666
Thermal correction to Gibbs free energy (25°C) = 0.061284
Thermal correction to Gibbs free energy (80°C) = 0.079139
qh-G(25°C) = -1210.091354
qh-G(80°C) = -1210.102491

Geometry:
C 0.971499 -0.856698 -0.446514
C 0.625512 -0.573677 0.875919
C -0.702821 -0.624531 1.292218
C -1.717554 -0.944346 0.382172
C -1.369716 -1.224775 -0.950775
C -0.043343 -1.193137 -1.350406
H 1.393704 -0.337458 1.605337
H -0.924127 -0.423103 2.331748
H -2.127300 -1.466040 -1.686610
H 0.218149 -1.420641 -2.378725
N -3.097560 -0.923720 0.761777
C -3.350623 -0.751622 2.199443
H -4.429665 -0.733559 2.354795
H -2.920208 -1.574072 2.781239
H -2.937994 0.197760 2.543570
C -3.897527 -2.041753 0.224465
H -4.931473 -1.907845 0.545182
H -3.878682 -2.045638 -0.863786
H -3.521264 -2.999142 0.599478
C -3.876208 0.769940 -0.091682
H -4.827764 0.553233 0.370331
H -3.140911 1.361359 0.432920
H -3.648779 0.379632 -1.071963
C 2.387737 -0.885679 -0.919235
O 2.713157 -1.635108 -1.830215
C 3.405186 0.001771 -0.276329
C 4.734482 -0.436584 -0.233999
C 3.075696 1.269280 0.218366
C 5.720726 0.374285 0.318247
H 4.978374 -1.416955 -0.631078
C 4.069517 2.088770 0.751076
H 2.051784 1.626425 0.166129
C 5.388528 1.639137 0.809441
H 6.747085 0.025087 0.363715
H 3.813483 3.076452 1.120038
H 6.159000 2.274776 1.234227
Cl -4.742488 2.630256 -1.018923

4Bz_TMA_gen
Electronic energy = -750.054463
Thermal correction to Gibbs free energy (25°C) = 0.056411
Thermal correction to Gibbs free energy (80°C) = 0.072957
qh-G(25°C) = -749.783820
qh-G(80°C) = -749.794148

Geometry:

C	2.394440	-0.022305	-0.042210
C	1.438445	-0.824510	-0.652323
C	0.099566	-0.432077	-0.618643
C	-0.275602	0.757762	0.002176
C	0.703360	1.559835	0.597381
C	2.034112	1.170136	0.588741
H	1.693368	-1.745894	-1.157034
H	0.414217	2.491945	1.070525
H	2.768749	1.805940	1.069198
N	3.840934	-0.399186	-0.050743
C	4.634162	0.670470	-0.757659
H	4.531247	1.609254	-0.218667
H	5.678117	0.359720	-0.771145
H	4.246271	0.768347	-1.770619
C	4.337199	-0.539433	1.365800
H	5.384411	-0.837052	1.325560
H	4.241874	0.415397	1.877103
H	3.737210	-1.300518	1.862604
C	4.100063	-1.699685	-0.754493
H	3.556814	-2.496270	-0.248658
H	3.796312	-1.611515	-1.796339
H	5.170316	-1.889721	-0.698161
H	-0.641061	-1.058197	-1.104824
C	-1.691101	1.258441	-0.015139
O	-1.897948	2.460953	-0.063866
C	-2.823414	0.287558	0.019224
C	-4.034681	0.663459	-0.575104
C	-2.721584	-0.945742	0.674165
C	-5.128010	-0.194934	-0.533119
H	-4.101260	1.626075	-1.072089
C	-3.825602	-1.794275	0.733097
H	-1.793939	-1.233851	1.159095
C	-5.024076	-1.423805	0.123426
H	-6.060685	0.091602	-1.007708
H	-3.749288	-2.742921	1.253878
H	-5.879291	-2.091017	0.161495

4Bz_aniline_gen

Electronic energy = -710.335264
Thermal correction to Gibbs free energy (25°C) = 0.054320
Thermal correction to Gibbs free energy (80°C) = 0.069855
qh-G(25°C) = -710.106803
qh-G(80°C) = -710.116645

Geometry:

C	2.756174	-0.104997	-0.043658
C	1.705286	-0.972931	-0.437623
C	0.387106	-0.549409	-0.410289
C	0.043161	0.751807	-0.010006
C	1.084750	1.620328	0.357045
C	2.404839	1.213269	0.355081
H	1.922461	-1.979743	-0.770936
H	0.836325	2.633606	0.658097
H	3.170838	1.914106	0.662275
N	4.054470	-0.517680	-0.049789
C	5.113619	0.402250	0.328636
H	4.988752	0.751709	1.360040
H	6.070681	-0.111217	0.256499
H	5.141031	1.276321	-0.332868
C	4.386580	-1.866903	-0.473899
H	3.893963	-2.614702	0.158145
H	4.091882	-2.044744	-1.515174
H	5.462822	-2.008712	-0.393776
H	-0.384892	-1.240443	-0.734724
C	-1.342083	1.263846	-0.026580
O	-1.574292	2.469956	-0.081336
C	-2.494442	0.304091	0.023728
C	-3.646405	0.610097	-0.710138
C	-2.473185	-0.838577	0.831638
C	-4.755336	-0.229724	-0.658386
H	-3.657575	1.506403	-1.322632
C	-3.592983	-1.666336	0.900902
H	-1.592076	-1.069055	1.422950
C	-4.730014	-1.368502	0.149713
H	-5.639682	0.003650	-1.242545
H	-3.576901	-2.542984	1.540342
H	-5.596982	-2.019946	0.196526

4Bz_bromide_ionpair_gen
Electronic energy = -3321.729362
Thermal correction to Gibbs free energy (25°C) = 0.061346
Thermal correction to Gibbs free energy (80°C) = 0.079137
qh-G(25°C) = -3321.460606
qh-G(80°C) = -3321.471694

Geometry:

Br 3.109217 2.706499 -0.347205
N 2.903649 -1.561183 0.323323
C 1.424316 -1.516734 0.119038
C 0.650822 -2.672169 0.176912
C -0.723077 -2.574113 -0.030460
C -1.322596 -1.337633 -0.267452
C -0.527231 -0.188861 -0.321552
C 3.277125 -0.670486 1.482602
H 4.350130 -0.770494 1.645865
H 2.721464 -1.003319 2.358582
H 3.037338 0.361825 1.227887
C 3.593132 -1.065785 -0.924182
H 4.667887 -1.164402 -0.771739
H 3.335183 -0.017711 -1.077396
H 3.264675 -1.682578 -1.760097
C 3.408521 -2.941272 0.618086
H 4.485196 -2.868316 0.760634
H 3.192755 -3.592746 -0.227189
H 2.939499 -3.309834 1.529193
C 0.848044 -0.274396 -0.140036
H 1.448589 0.630177 -0.203043
H 1.077267 -3.646075 0.374243
H -1.337212 -3.467815 -0.001066
C -2.800526 -1.297627 -0.523979
O -3.338569 -2.236395 -1.090758
H -0.971788 0.779627 -0.526976
C -3.594629 -0.112648 -0.084311
C -3.229925 0.646745 1.034047
C -4.767003 0.198010 -0.784934
C -4.033527 1.708169 1.446101
H -2.335824 0.398182 1.597462
C -5.555742 1.270895 -0.383723
H -5.041996 -0.404504 -1.644850
C -5.189866 2.025368 0.733835
H -3.756071 2.286634 2.320947
H -6.455180 1.519311 -0.937429
H -5.808521 2.859233 1.049996

4Bz_chloride_ionpair_gen
Electronic energy = -1210.407383
Thermal correction to Gibbs free energy (25°C) = 0.060694
Thermal correction to Gibbs free energy (80°C) = 0.078378
qh-G(25°C) = -1210.138099
qh-G(80°C) = -1210.149076

Geometry:
Cl 2.948702 3.386539 -0.625062
N 3.447152 -0.646730 0.261668

C 1.976298 -0.854477 0.101281
C 1.401430 -2.110102 0.273403
C 0.025757 -2.249583 0.106163
C -0.770435 -1.147771 -0.204927
C -0.172792 0.105247 -0.372873
C 3.693484 0.361290 1.356885
H 4.771845 0.451933 1.487444
H 3.225790 -0.008638 2.268686
H 3.275214 1.320964 1.052819
C 4.022779 -0.122373 -1.030762
H 5.102165 -0.038915 -0.903526
H 3.596906 0.860640 -1.232987
H 3.782525 -0.832993 -1.820719
C 4.174974 -1.906957 0.617979
H 5.229104 -1.655235 0.718272
H 4.044132 -2.636743 -0.179744
H 3.794589 -2.285674 1.565374
C 1.201801 0.256020 -0.230353
H 1.649336 1.237046 -0.377355
H 1.984410 -2.983999 0.529660
H -0.435287 -3.224303 0.224532
C -2.239735 -1.365949 -0.417579
O -2.629650 -2.425874 -0.883127
H -0.772795 0.970199 -0.636604
C -3.206891 -0.286246 -0.060695
C -2.955379 0.617073 0.979063
C -4.421044 -0.226768 -0.756394
C -3.912776 1.571394 1.318132
H -2.026968 0.562882 1.539183
C -5.365734 0.740227 -0.429532
H -4.606122 -0.938652 -1.554566
C -5.112315 1.638766 0.609843
H -3.721211 2.261545 2.132993
H -6.299035 0.794253 -0.980282
H -5.851932 2.389873 0.868411

4CHO_Ionpair_I_axial
Electronic energy = -530.611799
Thermal correction to Gibbs free energy (25°C) = 0.052952
Thermal correction to Gibbs free energy (80°C) = 0.067431
qh-G(25°C) = -530.421702
qh-G(80°C) = -530.431244

Geometry:
C 4.534612 0.126233 -0.000199
C 3.854327 1.339975 -0.000226
C 2.459136 1.370665 -0.000119
C 1.761273 0.169308 0.000018

C 2.433665 -1.057645 0.000047
C 3.818940 -1.075742 -0.000063
H 1.958983 2.328902 -0.000141
H 1.895799 -1.998612 0.000155
H 4.355340 -2.018736 -0.000043
N 0.270286 0.145257 0.000144
C -0.228151 -0.570023 -1.231098
H -1.319296 -0.546134 -1.203010
H 0.157172 -0.045165 -2.104736
H 0.127795 -1.597751 -1.218194
C -0.340959 1.517225 0.000042
H -1.423438 1.379503 0.000125
H -0.029695 2.047317 0.898988
H -0.029814 2.047135 -0.899052
C -0.227934 -0.569741 1.231634
H -1.319084 -0.545806 1.203797
H 0.127955 -1.597491 1.218867
H 0.157603 -0.044725 2.105084
I -4.065163 -0.092838 0.000029
H 4.406502 2.275157 -0.000330
C 6.018966 0.121922 -0.000312
O 6.686715 -0.891303 -0.000251
H 6.498850 1.117371 -0.000388

4CHO_SnArProduct_GEN
Electronic energy = -651.601226
Thermal correction to Gibbs free energy (25°C) = 0.048163
Thermal correction to Gibbs free energy (80°C) = 0.061529
qh-G(25°C) = -651.440205
qh-G(80°C) = -651.448953

Geometry:
C -3.640153 -0.671696 1.210187
C -2.505581 0.139249 1.218438
C -1.979574 0.569903 0.005113
C -2.555717 0.215791 -1.209797
C -3.690763 -0.594978 -1.205621
C -4.232438 -1.039812 0.001128
H -4.061361 -1.013435 2.150010
H -2.027836 0.438834 2.145862
H -2.115910 0.573712 -2.135192
H -4.151303 -0.877070 -2.146786
H -5.115892 -1.669610 -0.000374
O -0.878883 1.417914 0.007354
C 0.370665 0.871725 0.004717
C 1.430641 1.785096 0.030193
C 2.733619 1.312512 0.026799
H 1.209503 2.846491 0.052290

C	2.989842	-0.064731	-0.001858
H	3.562815	2.015109	0.046727
C	0.608254	-0.509109	-0.024585
H	-0.218101	-1.210023	-0.044884
C	1.919284	-0.965623	-0.027584
H	2.125421	-2.031304	-0.050429
C	4.382238	-0.541140	-0.004518
O	4.711124	-1.713997	-0.028660
H	5.152827	0.252842	0.017553

4CHO_TMA_DMSO
Electronic energy = -1072.184852
Thermal correction to Gibbs free energy (25°C) = 0.061449
Thermal correction to Gibbs free energy (80°C) = 0.079182
qh-G(25°C) = -1071.916708
qh-G(80°C) = -1071.927803

Geometry:

C	-1.364823	-1.037512	0.016274
C	-2.750529	-1.066114	0.116284
C	-3.447001	0.142907	0.136732
C	-2.763713	1.352787	0.058486
C	-1.368091	1.362342	-0.039942
C	-0.662157	0.169521	-0.061688
H	-3.308918	-1.989647	0.178895
H	-0.844894	2.311388	-0.099528
H	0.422694	0.180340	-0.137821
N	-0.566426	-2.299549	-0.013666
C	0.217756	-2.365989	-1.301992
H	0.938926	-1.549427	-1.317636
H	0.739749	-3.322777	-1.320054
H	-0.485657	-2.296162	-2.131032
C	0.396188	-2.311948	1.148844
H	0.924705	-3.265074	1.128312
H	1.103791	-1.492208	1.028955
H	-0.180898	-2.212669	2.067448
C	-1.419868	-3.528379	0.076443
H	-1.969950	-3.517040	1.016149
H	-2.096342	-3.561487	-0.776209
H	-0.753328	-4.388297	0.050878
H	-4.530238	0.131655	0.214263
C	-3.529868	2.623396	0.079908
O	-3.013924	3.720364	0.019734
H	-4.627499	2.517396	0.155020
O	2.500219	-0.348693	-0.279464
S	3.593337	0.711948	-0.468545
C	2.751269	2.296304	-0.275814
H	2.080582	2.411007	-1.128233

H 2.186117 2.281487 0.659120
H 3.491174 3.098924 -0.274484
C 4.511108 0.731821 1.084036
H 5.039147 -0.219122 1.158019
H 5.227128 1.555804 1.064718
H 3.803322 0.843405 1.908032

4CHO_TMA_DMSO_tsopt_GEN
Electronic energy = -1072.118391
Thermal correction to Gibbs free energy (25°C) = 0.061628
Thermal correction to Gibbs free energy (80°C) = 0.079439
qh-G(25°C) = -1071.853437
qh-G(80°C) = -1071.864631

Geometry:
C 3.421746 -0.636847 -0.191798
C 2.662881 -0.636916 0.984669
C 1.573213 0.207175 1.113703
C 1.216189 1.075596 0.064116
C 1.973318 1.071240 -1.114081
C 3.068079 0.218979 -1.233444
H 2.935649 -1.306056 1.794502
H 1.003077 0.181310 2.034348
H 1.735391 1.729298 -1.938911
H 3.653433 0.228721 -2.149053
N 0.044812 1.887983 0.194321
C -0.180060 2.850370 -0.894982
H -1.099481 3.396785 -0.683291
H 0.647077 3.563994 -0.970971
H -0.301301 2.325862 -1.843951
C -0.107642 2.544359 1.508672
H -1.048198 3.096453 1.509962
H -0.143884 1.809810 2.310890
H 0.720677 3.237222 1.687535
C -1.542978 0.572288 0.031655
H -1.128988 0.237643 -0.912613
H -1.227761 0.104520 0.956620
H -2.218245 1.419997 0.050148
S -3.272251 -0.811265 -0.220868
O -4.080464 -0.533177 -1.451482
C -4.335958 -0.774622 1.220534
H -4.728122 0.239500 1.303889
H -5.144730 -1.489429 1.058161
H -3.748331 -1.034486 2.102817
C -2.714889 -2.513707 -0.236000
H -3.593919 -3.154934 -0.321127
H -2.065828 -2.631007 -1.104385
H -2.166084 -2.714668 0.685618

C 4.588815 -1.528627 -0.344654
O 4.968510 -2.306807 0.509488
H 5.121248 -1.452050 -1.310935

4CHO_TMA_I_TS
Electronic energy = -530.563002
Thermal correction to Gibbs free energy (25°C) = 0.054113
Thermal correction to Gibbs free energy (80°C) = 0.068918
qh-G(25°C) = -530.377681
qh-G(80°C) = -530.387524

Geometry:
C 3.723992 -0.732207 -0.215973
C 2.986371 -0.690292 0.973640
C 1.920057 0.182188 1.104863
C 1.563535 1.039535 0.045370
C 2.298518 0.991095 -1.147200
C 3.368904 0.109830 -1.268902
H 3.258440 -1.348298 1.792994
H 1.365023 0.189393 2.034836
H 2.061879 1.638615 -1.980866
H 3.937423 0.087156 -2.195036
N 0.431585 1.895461 0.187986
C 0.234766 2.870439 -0.893474
H -0.667495 3.442257 -0.675969
H 1.084834 3.557791 -0.968529
H 0.092924 2.356905 -1.845082
C 0.308739 2.539827 1.510010
H -0.574709 3.178797 1.499150
H 0.176756 1.796866 2.294746
H 1.193754 3.147186 1.726470
C -1.243854 0.659982 0.055426
H -1.871777 1.520158 0.233005
H -0.968288 0.387716 -0.953056
H -0.881337 0.073653 0.886623
C 4.869062 -1.649233 -0.372501
O 5.256417 -2.415024 0.490092
H 5.380343 -1.604436 -1.352450
I -3.374354 -0.835826 -0.084548

4CHO_TMA_SN2_tsopt_GEN
Electronic energy = -825.935259
Thermal correction to Gibbs free energy (25°C) = 0.060996
Thermal correction to Gibbs free energy (80°C) = 0.078862
qh-G(25°C) = -825.657773
qh-G(80°C) = -825.668860

Geometry:

C	-3.026856	-1.366885	-0.142151
C	-3.326077	-0.288455	-0.972023
C	-2.656774	0.925285	-0.828687
C	-1.679068	1.066092	0.159734
C	-1.375209	-0.023229	0.995687
C	-2.043390	-1.226703	0.844107
H	-2.917239	1.743588	-1.486249
H	-0.613222	0.056901	1.762005
H	-1.807630	-2.069309	1.486281
N	-0.911528	2.282184	0.296078
C	0.845788	1.851411	-0.257144
H	1.204977	2.866850	-0.157438
H	0.991878	1.145328	0.549533
H	0.517251	1.497291	-1.223675
C	-0.822135	2.766686	1.693298
H	-0.180843	3.648941	1.706561
H	-1.816668	3.028209	2.065098
H	-0.379326	2.007539	2.334800
C	-1.339773	3.384417	-0.584958
H	-1.247453	3.084187	-1.629305
H	-2.369988	3.682013	-0.369391
H	-0.678532	4.232551	-0.406505
C	3.093465	0.224473	-0.473873
C	2.247777	-0.911488	-0.579106
C	4.407544	-0.002286	0.012590
C	2.695901	-2.182584	-0.221487
H	1.239043	-0.786955	-0.964388
C	4.844972	-1.276913	0.356129
H	5.070680	0.854097	0.104644
C	3.995038	-2.383522	0.248372
H	2.019570	-3.028411	-0.319433
H	5.861445	-1.410235	0.718405
H	4.339010	-3.375720	0.521586
O	2.697611	1.434179	-0.801027
H	-4.089936	-0.388979	-1.738301
C	-3.752843	-2.642958	-0.316272
O	-3.560727	-3.632926	0.362597
H	-4.507559	-2.653235	-1.124623

4CHO_TMA_SnAr_tsopt_GEN
Electronic energy = -825.952816
Thermal correction to Gibbs free energy (25°C) = 0.058473
Thermal correction to Gibbs free energy (80°C) = 0.075872
qh-G(25°C) = -825.672191
qh-G(80°C) = -825.682892

Geometry:

C	-0.958103	2.015921	0.281900
C	-0.370925	1.411637	1.408406
C	0.720032	0.577151	1.302304
C	1.224955	0.209743	0.017045
C	0.707061	0.924189	-1.112636
C	-0.378963	1.753726	-0.978146
H	-0.779916	1.616461	2.396161
H	1.123793	0.121168	2.197370
H	1.110235	0.740503	-2.101015
H	-0.798704	2.232526	-1.858812
N	2.724531	-0.118297	-0.036752
C	3.474903	1.177967	0.077296
H	4.541191	0.950601	0.085588
H	3.223140	1.804824	-0.777087
H	3.179815	1.671314	1.002141
C	3.136416	-0.780769	-1.320988
H	4.213346	-0.938255	-1.272381
H	2.601107	-1.720330	-1.410311
H	2.911145	-0.123213	-2.156746
C	3.150964	-1.006620	1.097031
H	3.084060	-0.454057	2.031356
H	2.502748	-1.878592	1.104480
H	4.189250	-1.286324	0.922146
O	0.744457	-1.570020	-0.255907
C	-0.574090	-1.709402	-0.169213
C	-1.394450	-1.564780	-1.308172
C	-1.196178	-1.978120	1.068206
C	-2.778560	-1.682066	-1.208888
H	-0.918425	-1.360845	-2.262959
C	-2.581234	-2.095595	1.159238
H	-0.567517	-2.093753	1.946723
C	-3.382542	-1.941548	0.024789
H	-3.392013	-1.568075	-2.098550
H	-3.039975	-2.305009	2.121821
H	-4.461681	-2.032174	0.098932
C	-2.099680	2.889864	0.435880
O	-2.687242	3.472870	-0.473595
H	-2.448676	3.020726	1.480111

4CHO_TMA_bromide_tsopt
Electronic energy = -3090.704160
Thermal correction to Gibbs free energy (25°C) = 0.052885
Thermal correction to Gibbs free energy (80°C) = 0.067481
qh-G(25°C) = -3090.517311
qh-G(80°C) = -3090.526963

Geometry:

C	3.191588	-0.578777	-0.216044

C 2.598607 -0.365328 1.033136
C 1.465385 0.423519 1.147577
C 0.896309 1.021719 0.006783
C 1.492725 0.807186 -1.244586
C 2.629659 0.013082 -1.347647
H 3.035678 -0.823559 1.914740
H 1.032877 0.570474 2.128775
H 1.091252 1.256516 -2.142813
H 3.084086 -0.142433 -2.322597
N -0.315188 1.766915 0.120550
C -0.611082 2.666441 -1.007599
H -1.536277 3.198160 -0.786572
H 0.196852 3.390429 -1.155974
H -0.762391 2.094145 -1.922935
C -0.513708 2.446565 1.414725
H -1.429481 3.034032 1.354661
H -0.634633 1.720289 2.217819
H 0.328549 3.108123 1.641033
C -1.799351 0.347319 0.042375
H -2.545407 1.118557 0.164070
H -1.472301 0.060282 -0.945669
H -1.380665 -0.142909 0.908685
C 4.398789 -1.415744 -0.353678
O 4.958336 -1.967101 0.575589
H 4.786310 -1.523382 -1.384126
Br -3.535667 -1.307035 -0.056226

4CHO_TMA_chloride_tsopt
Electronic energy = -979.382914
Thermal correction to Gibbs free energy (25°C) = 0.051819
Thermal correction to Gibbs free energy (80°C) = 0.066201
qh-G(25°C) = -979.194808
qh-G(80°C) = -979.204262

Geometry:
C 2.713020 -0.244780 -0.207076
C 1.967966 -0.505655 0.949516
C 0.692086 0.012201 1.091427
C 0.128202 0.807968 0.075253
C 0.872608 1.063323 -1.083433
C 2.155309 0.538333 -1.216374
H 2.399023 -1.120005 1.733642
H 0.137674 -0.212537 1.994466
H 0.477035 1.675316 -1.882645
H 2.726075 0.749116 -2.116895
N -1.215507 1.278386 0.217671
C -1.668486 2.213067 -0.823457
H -2.702980 2.482991 -0.610326

H	-1.057501	3.122044	-0.834573
H	-1.634005	1.733520	-1.802449
C	-1.542456	1.801269	1.559583
H	-2.589860	2.105033	1.562201
H	-1.411001	1.033455	2.319467
H	-0.912698	2.664165	1.798773
C	-2.403578	-0.368007	-0.036718
H	-3.278612	0.256702	0.063672
H	-1.961791	-0.531006	-1.008347
H	-1.963033	-0.826883	0.835355
C	4.076108	-0.785810	-0.373799
O	4.643686	-1.474551	0.452953
H	4.577077	-0.524586	-1.324727
Cl	-3.728883	-2.173593	-0.322615

4CHO_TMA_gen
Electronic energy = -519.080021
Thermal correction to Gibbs free energy (25°C) = 0.045561
Thermal correction to Gibbs free energy (80°C) = 0.058352
qh-G(25°C) = -518.884933
qh-G(80°C) = -518.893313

Geometry:

C	-0.666091	0.066497	-0.000538
C	-0.057239	1.315245	-0.000571
C	1.336515	1.384863	-0.000371
C	2.102016	0.223050	-0.000217
C	1.474815	-1.027040	-0.000353
C	0.091636	-1.109138	-0.000473
H	-0.624496	2.235279	-0.000681
H	2.077724	-1.928890	-0.000297
H	-0.374785	-2.087355	-0.000503
N	-2.154820	-0.064823	0.000058
C	-2.594966	-0.816954	-1.230440
H	-2.173081	-1.818883	-1.211691
H	-3.682785	-0.870770	-1.218863
H	-2.243863	-0.271297	-2.105114
C	-2.593876	-0.811491	1.234235
H	-3.681542	-0.868346	1.222134
H	-2.168927	-1.812198	1.221114
H	-2.244894	-0.260197	2.106225
C	-2.856269	1.262660	-0.002564
H	-2.583308	1.814254	0.895385
H	-2.583258	1.810790	-0.902638
H	-3.926454	1.063246	-0.002074
H	1.819439	2.357617	-0.000278
C	3.583735	0.323538	0.000199
O	4.319881	-0.640835	0.000381

H 3.993266 1.349809 0.000382

4CHO_aniline_gen
Electronic energy = -479.362805
Thermal correction to Gibbs free energy (25°C) = 0.044207
Thermal correction to Gibbs free energy (80°C) = 0.056167
qh-G(25°C) = -479.210757
qh-G(80°C) = -479.218827

Geometry:
C -0.993793 0.015003 -0.000087
C -0.347784 1.280063 -0.000003
C 1.033228 1.361604 0.000047
C 1.833716 0.210230 0.000044
C 1.201139 -1.044602 -0.000003
C -0.174741 -1.150411 -0.000057
H -0.929937 2.192540 0.000031
H 1.812853 -1.942228 0.000015
H -0.627218 -2.133874 -0.000075
N -2.350759 -0.082639 -0.000204
C -2.990585 -1.388035 0.000126
H -2.716768 -1.966550 0.889925
H -4.070416 -1.252040 0.000007
H -2.716659 -1.967016 -0.889326
C -3.166781 1.120070 0.000049
H -4.217249 0.835349 -0.000264
H -2.975780 1.731296 0.889732
H -2.975432 1.731833 -0.889178
H 1.506375 2.341165 0.000104
C 3.287323 0.335388 0.000115
O 4.075775 -0.600392 -0.000107
H 3.669007 1.375274 -0.000075

4CHO_bromide_ionpair_gen
Electronic energy = -3090.754806
Thermal correction to Gibbs free energy (25°C) = 0.052053
Thermal correction to Gibbs free energy (80°C) = 0.066372
qh-G(25°C) = -3090.563562
qh-G(80°C) = -3090.572968

Geometry:
Br -3.190293 -1.392294 0.000173
N -0.408256 1.919627 -0.000090
C 0.733170 0.955424 -0.000172
C 2.053378 1.402414 0.000347
C 3.081858 0.465201 0.000364
C 2.790743 -0.898821 -0.000142

C	1.462432	-1.328005	-0.000678
C	-1.254133	1.693813	1.229213
H	-2.042153	2.446933	1.231547
H	-0.615895	1.801343	2.105606
H	-1.694487	0.698035	1.177228
C	-1.254484	1.693884	-1.229161
H	-2.042593	2.446910	-1.231134
H	-1.694764	0.698081	-1.177229
H	-0.616528	1.801626	-2.105733
C	0.038450	3.350558	-0.000156
H	-0.857804	3.967950	-0.000231
H	0.621062	3.546887	-0.899085
H	0.620940	3.547033	0.898813
C	0.424272	-0.403880	-0.000678
H	-0.605080	-0.755080	-0.001117
H	2.307436	2.453363	0.000708
H	4.116337	0.792186	0.000762
C	3.881634	-1.905089	-0.000105
O	5.061256	-1.619308	0.000386
H	3.554828	-2.960964	-0.000540
H	1.232785	-2.389935	-0.001093

4CHO_chloride_ionpair_gen
Electronic energy = -979.432977
Thermal correction to Gibbs free energy (25°C) = 0.051069
Thermal correction to Gibbs free energy (80°C) = 0.065257
qh-G(25°C) = -979.240705
qh-G(80°C) = -979.249978

Geometry:

Cl	3.073418	2.469943	0.000458
N	1.388915	-1.334644	-0.000127
C	0.026821	-0.720538	-0.000143
C	-1.119901	-1.513048	0.000511
C	-2.366010	-0.894016	0.000508
C	-2.460681	0.497475	-0.000108
C	-1.301364	1.275326	-0.000786
C	2.139751	-0.884699	1.229386
H	3.103177	-1.394331	1.234340
H	1.553753	-1.161995	2.104830
H	2.291591	0.193432	1.174482
C	2.139865	-0.884780	-1.229598
H	3.103239	-1.394504	-1.234472
H	2.291828	0.193335	-1.174733
H	1.553901	-1.162048	-2.105072
C	1.349955	-2.832864	-0.000097
H	2.380139	-3.184514	-0.000154
H	0.841844	-3.179606	-0.898605

H 0.841982 -3.179603 0.898483
C -0.049058 0.671579 -0.000833
H 0.846592 1.289723 -0.001479
H -1.075979 -2.593291 0.000986
H -3.271067 -1.492283 0.000998
C -3.786368 1.164384 -0.000074
O -4.841721 0.564676 0.000332
H -3.763738 2.269294 -0.000451
H -1.372069 2.359550 -0.001301

4CHO_dimer_Tshape_CONF1
Electronic energy = -1061.236976
Thermal correction to Gibbs free energy (25°C) = 0.083972
Thermal correction to Gibbs free energy (80°C) = 0.108899
qh-G(25°C) = -1060.833821
qh-G(80°C) = -1060.848732

Geometry:
C 1.701817 3.176142 -0.927251
C 0.991379 3.516595 -2.074387
C -0.389108 3.318181 -2.136402
C -1.039058 2.773437 -1.035747
C -0.339365 2.429793 0.125938
C 1.029294 2.640223 0.177746
H -0.913793 3.592888 -3.040859
H -0.841343 1.991046 0.985293
H 1.585436 2.397998 1.079457
N -2.506306 2.509689 -1.056588
C -2.726934 1.014165 -1.005934
H -3.801054 0.828242 -1.030244
H -2.245079 0.567502 -1.874553
H -2.304423 0.623576 -0.078793
C -3.175886 3.038987 -2.288943
H -4.237603 2.816275 -2.201296
H -3.024228 4.116255 -2.346559
H -2.770671 2.534943 -3.164428
C -3.163472 3.150886 0.140268
H -4.238151 2.997590 0.048015
H -2.803462 2.670141 1.049367
H -2.923903 4.213795 0.133681
I -2.027235 0.265732 2.962887
C 5.445054 -1.228312 0.241823
C 4.955768 -2.512654 0.023769
C 3.597982 -2.722278 -0.219920
C 2.740759 -1.628057 -0.240058
C 3.222381 -0.333405 -0.021657
C 4.572697 -0.135148 0.219326
H 3.250040 -3.731730 -0.386951

H 2.562161 0.526352 -0.036582
H 4.951109 0.867646 0.387069
N 1.280594 -1.800663 -0.493686
C 0.485555 -1.331104 0.702632
H -0.572215 -1.485417 0.477966
H 0.790107 -1.914531 1.571344
H 0.680314 -0.272998 0.870885
C 0.901484 -3.229743 -0.757445
H -0.174836 -3.245626 -0.933260
H 1.433586 -3.583723 -1.639528
H 1.144013 -3.831204 0.117254
C 0.860772 -0.991093 -1.694139
H -0.200335 -1.189250 -1.861813
H 1.018573 0.067165 -1.487071
H 1.458583 -1.305668 -2.548906
I -2.993267 -2.560694 -1.224244
H 1.507814 3.937318 -2.932046
H 5.629701 -3.364237 0.040476
C 6.894316 -1.033105 0.495970
O 7.401528 0.051767 0.692095
H 7.506871 -1.952998 0.497048
C 3.173179 3.364149 -0.891181
O 3.867154 2.981165 0.029042
H 3.618221 3.869026 -1.766665

4CHO_dimer_Tshape_CONF2
Electronic energy = -1061.236907
Thermal correction to Gibbs free energy (25°C) = 0.085546
Thermal correction to Gibbs free energy (80°C) = 0.110869
qh-G(25°C) = -1060.834241
qh-G(80°C) = -1060.849389

Geometry:
C 1.511673 3.395650 -0.964365
C 0.730985 3.722888 -2.068864
C -0.632476 3.422655 -2.086158
C -1.194082 2.793194 -0.982213
C -0.425666 2.472863 0.141918
C 0.925360 2.780210 0.148153
H -1.212031 3.685332 -2.960288
H -0.861642 1.978184 1.007091
H 1.534167 2.550247 1.018468
N -2.633729 2.406724 -0.959256
C -2.727562 0.897048 -0.924763
H -3.782244 0.621847 -0.935709
H -2.223517 0.502530 -1.806179
H -2.260398 0.531690 -0.008779
C -3.385908 2.893119 -2.161139

H	-4.421427	2.579055	-2.044028
H	-3.328990	3.979933	-2.204764
H	-2.967078	2.439472	-3.057725
C	-3.302556	2.973790	0.268118
H	-4.361596	2.724165	0.211331
H	-2.867485	2.518716	1.157433
H	-3.160655	4.053923	0.267564
I	-1.873749	0.201375	3.030735
C	5.404185	-0.876784	0.219276
C	4.997527	-2.202120	0.069814
C	3.665450	-2.503008	-0.196380
C	2.744073	-1.463266	-0.312037
C	3.141135	-0.134153	-0.169262
C	4.473479	0.156516	0.098626
H	3.378216	-3.539184	-0.305977
H	2.433875	0.682561	-0.259436
H	4.776718	1.193442	0.212093
N	1.303173	-1.741557	-0.581159
C	0.460801	-1.252020	0.574834
H	-0.581768	-1.484743	0.345802
H	0.785251	-1.764858	1.479888
H	0.592938	-0.176871	0.684888
C	1.016023	-3.204728	-0.760779
H	-0.054120	-3.296848	-0.950718
H	1.584834	-3.579615	-1.610864
H	1.277773	-3.734374	0.154017
C	0.859510	-1.034837	-1.836589
H	-0.182936	-1.310507	-2.011030
H	0.944319	0.041907	-1.693291
H	1.495601	-1.361880	-2.658608
I	-2.922980	-2.683964	-1.234498
H	1.180130	4.208448	-2.930204
H	5.727092	-2.999672	0.163573
C	6.822907	-0.549085	0.508025
O	7.694796	-1.384958	0.626232
H	7.051761	0.526937	0.613222
C	2.968049	3.677368	-0.985132
O	3.728765	3.298813	-0.117118
H	3.337826	4.248555	-1.854837

4CHO_dimer_Tshape_CONF3
Electronic energy = -1061.235440
Thermal correction to Gibbs free energy (25°C) = 0.086285
Thermal correction to Gibbs free energy (80°C) = 0.111712
qh-G(25°C) = -1060.833629
qh-G(80°C) = -1060.848871

Geometry:

C	1.827363	3.511231	-0.550200
C	1.148917	3.883894	-1.710127
C	-0.202020	3.585272	-1.861516
C	-0.863365	2.905209	-0.840571
C	-0.195893	2.513761	0.319991
C	1.151878	2.824268	0.461372
H	-0.703886	3.893775	-2.768191
H	-0.704027	1.973977	1.116256
H	1.674873	2.533840	1.369642
N	-2.312503	2.566858	-0.950652
C	-2.472749	1.063836	-0.981070
H	-3.533998	0.837222	-1.083346
H	-1.919757	0.678680	-1.837285
H	-2.097217	0.645361	-0.045858
C	-2.946235	3.123936	-2.189784
H	-3.999084	2.849338	-2.166322
H	-2.844462	4.208314	-2.190014
H	-2.473837	2.682590	-3.065703
C	-3.057123	3.118879	0.239876
H	-4.116286	2.910006	0.092557
H	-2.707916	2.620800	1.143957
H	-2.879112	4.192609	0.287517
I	-1.973985	0.183600	2.982349
C	5.284405	-1.307606	0.245444
C	4.799545	-2.610557	0.140741
C	3.452718	-2.840403	-0.122442
C	2.596533	-1.752127	-0.280030
C	3.069486	-0.444236	-0.177353
C	4.415993	-0.226339	0.086622
H	3.104130	-3.860770	-0.198094
H	2.409842	0.407694	-0.298676
H	4.791143	0.789295	0.169770
N	1.146211	-1.949436	-0.563352
C	0.317970	-1.333282	0.540780
H	-0.732783	-1.525360	0.312930
H	0.602791	-1.796594	1.484816
H	0.505958	-0.260853	0.575581
C	0.759824	-3.397416	-0.658008
H	-0.310246	-3.425331	-0.868226
H	1.315828	-3.864368	-1.469910
H	0.966259	-3.883817	0.294254
C	0.783805	-1.296868	-1.873066
H	-0.275011	-1.496400	-2.052494
H	0.960418	-0.224617	-1.801713
H	1.403752	-1.733572	-2.655453
I	-3.101121	-2.497569	-1.361797
H	1.679839	4.415943	-2.492541
H	5.479981	-3.446236	0.266010
C	6.720445	-1.055307	0.526014

O 7.539697 -1.937557 0.676764
H 7.014163 0.008055 0.592098
C 3.262804 3.847844 -0.374249
O 3.922010 4.434748 -1.206791
H 3.713864 3.527372 0.582864

4CHO_dimer_Tshape_CONF4
Electronic energy = -1061.235656
Thermal correction to Gibbs free energy (25°C) = 0.085921
Thermal correction to Gibbs free energy (80°C) = 0.111260
qh-G(25°C) = -1060.833208
qh-G(80°C) = -1060.848378

Geometry:
C 2.238578 3.223999 -0.561767
C 1.634884 3.632700 -1.750629
C 0.264117 3.475007 -1.934465
C -0.492911 2.898697 -0.916369
C 0.098501 2.468939 0.271399
C 1.467293 2.638831 0.445149
H -0.177557 3.810036 -2.862713
H -0.484680 2.007288 1.065258
H 1.931769 2.317415 1.374415
N -1.968158 2.720922 -1.055336
C -2.299479 1.246226 -1.041304
H -3.376491 1.140069 -1.168143
H -1.773415 0.769663 -1.867737
H -2.000341 0.820980 -0.081785
C -2.508316 3.302506 -2.327314
H -3.584599 3.139204 -2.325284
H -2.294871 4.370240 -2.355312
H -2.062549 2.787748 -3.176833
C -2.671319 3.393580 0.097493
H -3.743807 3.293481 -0.066567
H -2.394593 2.894238 1.025704
H -2.379132 4.443048 0.111195
I -1.966608 0.444288 2.961394
C 5.095109 -1.905740 0.279025
C 4.476613 -3.151244 0.235438
C 3.108192 -3.254236 -0.018060
C 2.374046 -2.092904 -0.225083
C 2.984732 -0.834951 -0.183712
C 4.343993 -0.744651 0.067943
H 2.655851 -4.235512 -0.047668
H 2.415215 0.073584 -0.346805
H 4.836346 0.221285 0.102200
N 0.910382 -2.146130 -0.501982
C 0.154715 -1.405228 0.577178

H -0.909654 -1.470219 0.340385
H 0.369454 -1.877808 1.535145
H 0.476312 -0.364808 0.591361
C 0.372266 -3.547118 -0.545468
H -0.695913 -3.469156 -0.752949
H 0.871880 -4.096378 -1.342193
H 0.531632 -4.021107 0.421957
C 0.619622 -1.508015 -1.836537
H -0.452227 -1.611067 -2.019415
H 0.897869 -0.455805 -1.799670
H 1.200047 -2.029415 -2.597234
I -3.368623 -2.224607 -1.309885
H 2.240130 4.083928 -2.529860
H 5.057108 -4.054658 0.398282
C 6.552066 -1.823044 0.550378
O 7.165593 -0.777543 0.605618
H 7.064358 -2.790027 0.704060
C 3.695916 3.415379 -0.349811
O 4.436819 3.899848 -1.179574
H 4.082550 3.086266 0.632196

4CHO_dimer_anti_CONF1
Electronic energy = -1061.240902
Thermal correction to Gibbs free energy (25°C) = 0.086500
Thermal correction to Gibbs free energy (80°C) = 0.111950
qh-G(25°C) = -1060.839138
qh-G(80°C) = -1060.854420

Geometry:
C -4.000244 -0.808610 -0.038777
C -5.042116 -1.725808 -0.080936
C -6.358659 -1.254643 -0.085698
C -6.631907 0.112836 -0.048581
C -5.576377 1.016675 -0.007138
C -4.259610 0.560997 -0.002576
H -4.876238 -2.796115 -0.110264
H -7.663392 0.449436 -0.052766
H -5.765369 2.084159 0.021956
N -2.574725 -1.249662 -0.031274
C -1.890679 -0.750816 1.217028
H -0.869087 -1.137532 1.203081
H -2.439333 -1.128737 2.079170
H -1.883508 0.339944 1.205528
C -1.857490 -0.687783 -1.233106
H -0.834264 -1.069782 -1.207337
H -1.858473 0.401155 -1.168621
H -2.377342 -1.025806 -2.129172
C -2.431208 -2.742359 -0.067156

H -1.362620 -2.961086 -0.058398
H -2.883845 -3.122603 -0.982012
H -2.907664 -3.167265 0.815446
H -3.452213 1.288792 0.029910
N 2.535431 1.564031 0.050646
C 3.887898 0.937554 -0.014464
C 4.308304 0.034165 0.959495
H 3.672849 -0.255321 1.784509
C 5.576179 -0.528803 0.857905
H 5.918327 -1.237241 1.605078
C 6.413055 -0.193343 -0.205916
C 5.978268 0.710504 -1.176967
H 6.625716 0.973216 -2.008496
C 4.714383 1.280488 -1.084242
H 4.394248 1.979201 -1.848564
C 1.737712 1.170335 -1.168333
H 0.753763 1.635293 -1.076434
H 1.665129 0.081213 -1.177207
H 2.245702 1.533648 -2.059407
C 2.664657 3.064263 0.101659
H 1.656116 3.476190 0.169747
H 3.153868 3.415799 -0.803884
H 3.255454 3.325033 0.979243
C 1.752207 1.136567 1.259569
H 0.789505 1.647852 1.207174
H 2.290837 1.439670 2.156842
H 1.612330 0.055033 1.219811
I -1.450429 3.335340 0.103198
I 1.597257 -2.698386 0.016037
C -7.462431 -2.245940 -0.130704
O -8.637560 -1.943621 -0.138747
H -7.151050 -3.306407 -0.157389
C 7.766660 -0.790519 -0.321232
O 8.224661 -1.578918 0.479857
H 8.355314 -0.471141 -1.200457

4CHO_dimer_anti_CONF2
Electronic energy = -1061.241549
Thermal correction to Gibbs free energy (25°C) = 0.086548
Thermal correction to Gibbs free energy (80°C) = 0.112011
qh-G(25°C) = -1060.839767
qh-G(80°C) = -1060.855063

Geometry:
C -4.048058 -0.705164 -0.032891
C -5.103612 -1.600219 -0.061623
C -6.411979 -1.099281 -0.058806
C -6.655270 0.270947 -0.027505

C	-5.579519	1.157759	0.000842
C	-4.276961	0.674417	-0.002300
H	-4.976571	-2.675257	-0.086248
H	-7.676253	0.641909	-0.025541
H	-5.748035	2.228640	0.025288
N	-2.631104	-1.173551	-0.033933
C	-1.928553	-0.683448	1.207291
H	-0.914195	-1.088440	1.186187
H	-2.476696	-1.048939	2.075108
H	-1.901783	0.406992	1.192770
C	-1.912089	-0.629939	-1.242893
H	-0.896132	-1.031092	-1.222862
H	-1.890944	0.459016	-1.182676
H	-2.444886	-0.961043	-2.133896
C	-2.517678	-2.668905	-0.065420
H	-1.453629	-2.908881	-0.063612
H	-2.985583	-3.042832	-0.975123
H	-2.996816	-3.080847	0.821811
H	-3.453083	1.383747	0.020169
N	2.533386	1.555540	0.053110
C	3.874400	0.904155	-0.003242
C	4.268727	-0.012436	0.969333
H	3.620134	-0.294781	1.786568
C	5.526846	-0.598315	0.876348
H	5.848626	-1.317235	1.622558
C	6.379884	-0.272456	-0.177576
C	5.971052	0.644605	-1.147520
H	6.631063	0.899547	-1.971555
C	4.717139	1.237491	-1.063320
H	4.417169	1.946028	-1.826781
C	1.739930	1.184345	-1.175619
H	0.764548	1.668272	-1.090785
H	1.645743	0.096920	-1.191534
H	2.263745	1.542513	-2.059580
C	2.690367	3.052640	0.114817
H	1.689094	3.483408	0.172885
H	3.197509	3.400187	-0.782386
H	3.274760	3.296631	1.001472
C	1.731098	1.135846	1.252195
H	0.779431	1.666909	1.194959
H	2.267867	1.422402	2.155978
H	1.570127	0.057616	1.203605
I	-1.421472	3.398198	0.086589
I	1.517589	-2.688589	-0.008509
C	-7.554116	-2.045979	-0.089227
O	-7.427141	-3.252915	-0.118431
H	-8.556688	-1.581791	-0.083736
C	7.723377	-0.893664	-0.283683
O	8.159019	-1.695584	0.516452

H 8.326378 -0.579170 -1.154930

4CHO_dimer_anti_CONF3
Electronic energy = -1061.241549
Thermal correction to Gibbs free energy (25°C) = 0.086547
Thermal correction to Gibbs free energy (80°C) = 0.112009
qh-G(25°C) = -1060.839766
qh-G(80°C) = -1060.855061

Geometry:
C -4.048004 -0.705138 -0.032705
C -5.103724 -1.599977 -0.061683
C -6.411992 -1.098763 -0.059024
C -6.655002 0.271511 -0.027619
C -5.579074 1.158105 0.000995
C -4.276620 0.674491 -0.001999
H -4.976895 -2.675044 -0.086370
H -7.675912 0.642683 -0.025777
H -5.747375 2.229019 0.025564
N -2.631131 -1.173766 -0.033662
C -1.928488 -0.683477 1.207397
H -0.914107 -1.088416 1.186372
H -2.476595 -1.048873 2.075283
H -1.901757 0.406957 1.192847
C -1.912145 -0.630743 -1.242946
H -0.896185 -1.031838 -1.222590
H -1.891195 0.458254 -1.183419
H -2.445048 -0.962516 -2.133638
C -2.517958 -2.669160 -0.064716
H -1.453950 -2.909314 -0.062932
H -2.985944 -3.043273 -0.974297
H -2.997154 -3.080762 0.822639
H -3.452615 1.383644 0.020601
N 2.533120 1.555627 0.052817
C 3.874202 0.904372 -0.003426
C 4.268567 -0.012179 0.969178
H 3.619966 -0.294564 1.786396
C 5.526735 -0.597963 0.876270
H 5.848525 -1.316857 1.622503
C 6.379812 -0.272057 -0.177606
C 5.970964 0.644968 -1.147575
H 6.631010 0.899969 -1.971564
C 4.717000 1.237755 -1.063451
H 4.417028 1.946251 -1.826953
C 1.739736 1.184234 -1.175922
H 0.764264 1.667966 -1.091114
H 1.645777 0.096799 -1.191829
H 2.263543 1.542546 -2.059823

C	2.689887	3.052774	0.114320
H	1.688532	3.483353	0.172318
H	3.196984	3.400270	-0.782928
H	3.274239	3.296982	1.000943
C	1.730862	1.136073	1.252002
H	0.779153	1.667026	1.194654
H	2.267756	1.422889	2.155626
H	1.569971	0.057822	1.203600
I	-1.421328	3.397922	0.086716
I	1.517752	-2.688958	-0.008404
C	-7.554320	-2.045224	-0.089701
O	-7.427583	-3.252182	-0.119050
H	-8.556799	-1.580833	-0.084335
C	7.723348	-0.893181	-0.283646
O	8.158998	-1.695077	0.516508
H	8.326350	-0.578682	-1.154893

4CHO_dimer_anti_CONF4
Electronic energy = -1061.240902
Thermal correction to Gibbs free energy (25°C) = 0.086502
Thermal correction to Gibbs free energy (80°C) = 0.111952
qh-G(25°C) = -1060.839139
qh-G(80°C) = -1060.854421

Geometry:

C	-4.000290	-0.808819	-0.038295
C	-5.042314	-1.725806	-0.081128
C	-6.358760	-1.254356	-0.085738
C	-6.631734	0.113161	-0.047805
C	-5.576037	1.016775	-0.005680
C	-4.259369	0.560806	-0.001255
H	-4.876631	-2.796125	-0.111072
H	-7.663156	0.449948	-0.051896
H	-5.764808	2.084276	0.024088
N	-2.574827	-1.250062	-0.031074
C	-1.890473	-0.751513	1.217153
H	-0.868792	-1.137964	1.202732
H	-2.438723	-1.129929	2.079340
H	-1.883516	0.339258	1.206313
C	-1.857767	-0.688263	-1.232983
H	-0.834532	-1.070263	-1.207304
H	-1.858702	0.400681	-1.168653
H	-2.377566	-1.026345	-2.129063
C	-2.431475	-2.742796	-0.067101
H	-1.362894	-2.961543	-0.058574
H	-2.884230	-3.122899	-0.981951
H	-2.907883	-3.167728	0.815505
H	-3.451813	1.288408	0.031679

N 2.535298 1.564155 0.050665
C 3.887772 0.937686 -0.014463
C 4.308290 0.034500 0.959637
H 3.672899 -0.254868 1.784738
C 5.576196 -0.528396 0.858078
H 5.918439 -1.236677 1.605352
C 6.412989 -0.193067 -0.205856
C 5.978091 0.710583 -1.177036
H 6.625472 0.973202 -2.008643
C 4.714170 1.280504 -1.084339
H 4.393954 1.979084 -1.848744
C 1.737526 1.170471 -1.168270
H 0.753668 1.635665 -1.076458
H 1.665168 0.081332 -1.177221
H 2.245463 1.533714 -2.059424
C 2.664616 3.064376 0.101751
H 1.656141 3.476446 0.169959
H 3.153796 3.415894 -0.803813
H 3.255510 3.325033 0.979300
C 1.752026 1.136610 1.259516
H 0.789326 1.647908 1.207099
H 2.290487 1.439676 2.156909
H 1.612212 0.055058 1.219726
I -1.450553 3.335067 0.102866
I 1.597616 -2.698369 0.015920
C -7.462739 -2.245384 -0.131457
O -8.637802 -1.942804 -0.139463
H -7.151595 -3.305905 -0.158728
C 7.766626 -0.790167 -0.321135
O 8.224727 -1.578386 0.480075
H 8.355206 -0.470919 -1.200455

4CHO_dimer_stacked_CONF1
Electronic energy = -1061.239335
Thermal correction to Gibbs free energy (25°C) = 0.084759
Thermal correction to Gibbs free energy (80°C) = 0.109983
qh-G(25°C) = -1060.835639
qh-G(80°C) = -1060.850699

Geometry:
C -2.349196 -1.599399 -1.806023
C -1.661715 -1.473570 -3.010798
C -0.285770 -1.700988 -3.069320
C 0.391112 -2.039173 -1.901808
C -0.285322 -2.161709 -0.683669
C -1.656434 -1.949846 -0.642290
H 0.216468 -1.599938 -4.021478
H 0.239997 -2.392681 0.238712

H	-2.196913	-2.047602	0.294749
N	1.872970	-2.220109	-1.901060
C	2.501031	-0.994742	-1.276197
H	3.583961	-1.123663	-1.286125
H	2.221274	-0.123277	-1.867022
H	2.145807	-0.904170	-0.246981
C	2.434601	-2.386036	-3.283285
H	3.500575	-2.579493	-3.178841
H	1.944482	-3.229725	-3.768175
H	2.290761	-1.467477	-3.848179
C	2.259618	-3.439155	-1.100582
H	3.332112	-3.584038	-1.221713
H	2.035488	-3.268839	-0.048313
H	1.709621	-4.294606	-1.491064
I	1.740502	-1.790584	2.681861
C	-4.951108	1.165949	0.591699
C	-4.493333	2.044217	-0.390269
C	-3.132103	2.294834	-0.525198
C	-2.234184	1.657914	0.331087
C	-2.679783	0.780912	1.319608
C	-4.043650	0.537923	1.443254
H	-2.796701	2.983986	-1.291668
H	-1.997410	0.279152	1.992762
H	-4.407434	-0.152743	2.196674
N	-0.779709	1.943901	0.161263
C	0.081080	1.142408	1.097725
H	1.120222	1.390260	0.872055
H	-0.153526	1.412288	2.126756
H	-0.093952	0.080838	0.928498
C	-0.504458	3.402757	0.420474
H	0.570785	3.555485	0.306732
H	-1.052582	4.004258	-0.302044
H	-0.828239	3.634231	1.434577
C	-0.351452	1.602858	-1.241871
H	0.719838	1.804826	-1.314464
H	-0.570388	0.548034	-1.410884
H	-0.899688	2.218770	-1.951005
I	3.514520	2.507385	-0.422665
H	-2.194236	-1.198401	-3.916549
H	-5.197328	2.532972	-1.057591
C	-6.403299	0.880914	0.696390
O	-6.881234	0.118858	1.510995
H	-7.046848	1.410562	-0.029550
C	-3.814685	-1.357012	-1.769249
O	-4.497379	-1.545499	-0.784370
H	-4.261924	-0.987200	-2.709734

4CHO_dimer_stacked_CONF2

Electronic energy = -1061.239594
Thermal correction to Gibbs free energy (25°C) = 0.085081
Thermal correction to Gibbs free energy (80°C) = 0.110370
qh-G(25°C) = -1060.836462
qh-G(80°C) = -1060.851573

Geometry:

C	-2.382362	-1.698314	-1.665206
C	-1.751952	-1.543709	-2.897497
C	-0.372715	-1.723473	-3.016262
C	0.365115	-2.042247	-1.880734
C	-0.252889	-2.187849	-0.634545
C	-1.627507	-2.024909	-0.533562
H	0.083895	-1.604452	-3.989062
H	0.319519	-2.403376	0.263243
H	-2.123402	-2.145624	0.425109
N	1.849882	-2.184327	-1.946300
C	2.475722	-0.948635	-1.340774
H	3.560030	-1.054028	-1.394603
H	2.153532	-0.081461	-1.916088
H	2.160043	-0.869535	-0.297926
C	2.353230	-2.326791	-3.353246
H	3.427745	-2.490903	-3.298062
H	1.865639	-3.181276	-3.821484
H	2.158400	-1.409094	-3.904125
C	2.301626	-3.398455	-1.172908
H	3.370983	-3.514952	-1.342304
H	2.120319	-3.240666	-0.110462
H	1.756286	-4.264988	-1.544963
I	1.904603	-1.767601	2.641482
C	-4.921249	0.961653	0.826547
C	-4.541378	1.886863	-0.149568
C	-3.201218	2.196038	-0.327570
C	-2.239844	1.573144	0.474849
C	-2.605828	0.659967	1.457631
C	-3.956851	0.357752	1.626513
H	-2.923543	2.916910	-1.088116
H	-1.877835	0.170713	2.090941
H	-4.249673	-0.368504	2.379231
N	-0.806473	1.911948	0.240856
C	0.124367	1.121082	1.117242
H	1.142702	1.409111	0.848611
H	-0.070250	1.360691	2.162049
H	-0.024660	0.058226	0.932259
C	-0.565076	3.373413	0.516353
H	0.496498	3.564784	0.346593
H	-1.172632	3.970660	-0.160595
H	-0.838283	3.572923	1.551863
C	-0.439773	1.611191	-1.188960

H 0.621791 1.840112 -1.308995
H -0.642215 0.554876 -1.370758
H -1.036227 2.228570 -1.856817
I 3.443346 2.586186 -0.533520
H -2.331769 -1.286526 -3.779118
H -5.299370 2.353135 -0.770169
C -6.344324 0.577054 0.992058
O -7.247440 1.041471 0.327335
H -6.544643 -0.182126 1.769855
C -3.854044 -1.518737 -1.565568
O -4.486332 -1.748238 -0.555760
H -4.356156 -1.157665 -2.481305

4CHO_dimer_stacked_CONF3
Electronic energy = -1061.239158
Thermal correction to Gibbs free energy (25°C) = 0.085268
Thermal correction to Gibbs free energy (80°C) = 0.110574
qh-G(25°C) = -1060.835960
qh-G(80°C) = -1060.851100

Geometry:
C -2.358307 -1.855371 -1.434398
C -1.773562 -1.769865 -2.698436
C -0.399768 -1.935460 -2.849976
C 0.385195 -2.174250 -1.721788
C -0.186375 -2.265607 -0.452694
C -1.562551 -2.115288 -0.317322
H 0.023852 -1.863278 -3.842226
H 0.416964 -2.424693 0.436729
H -2.012468 -2.194079 0.669369
N 1.870111 -2.280030 -1.830848
C 2.478627 -0.991825 -1.323969
H 3.563518 -1.074088 -1.404350
H 2.116234 -0.169483 -1.940581
H 2.191118 -0.855914 -0.278615
C 2.330319 -2.499175 -3.242931
H 3.410946 -2.626151 -3.214742
H 1.855500 -3.397720 -3.636168
H 2.087421 -1.627030 -3.846019
C 2.383440 -3.430650 -1.000384
H 3.448942 -3.530826 -1.202542
H 2.236274 -3.209613 0.056214
H 1.848794 -4.332384 -1.297178
I 2.141580 -1.566539 2.684394
C -4.955778 0.851481 1.007066
C -4.623486 1.674684 -0.073387
C -3.296519 1.990268 -0.322769
C -2.297519 1.473441 0.508626

C -2.614201 0.653931 1.587064
C -3.953661 0.348155 1.831712
H -3.058537 2.636528 -1.159645
H -1.858416 0.245798 2.244884
H -4.208073 -0.290922 2.672628
N -0.882044 1.838760 0.213598
C 0.097711 1.130790 1.108400
H 1.098996 1.426065 0.787678
H -0.068008 1.434905 2.141511
H -0.028535 0.054685 0.996718
C -0.685945 3.321552 0.400272
H 0.363498 3.538349 0.189669
H -1.334009 3.857611 -0.290763
H -0.938452 3.569931 1.430596
C -0.537513 1.470056 -1.206184
H 0.514314 1.720993 -1.362083
H -0.711937 0.400382 -1.325833
H -1.163092 2.033903 -1.894459
I 3.280686 2.671813 -0.755521
H -2.396074 -1.568880 -3.564186
H -5.409566 2.062853 -0.712669
C -6.373114 0.503095 1.282651
O -7.300742 0.886669 0.601518
H -6.543726 -0.142589 2.162948
C -3.820122 -1.658055 -1.256441
O -4.569879 -1.347718 -2.157653
H -4.198312 -1.808052 -0.228042

4CHO_dimer_stacked_CONF4
Electronic energy = -1061.239065
Thermal correction to Gibbs free energy (25°C) = 0.085676
Thermal correction to Gibbs free energy (80°C) = 0.111028
qh-G(25°C) = -1060.836694
qh-G(80°C) = -1060.851853

Geometry:
C -2.401531 -1.934349 -1.168018
C -1.874746 -1.961378 -2.459958
C -0.510044 -2.153783 -2.659139
C 0.324901 -2.300992 -1.551074
C -0.187624 -2.270976 -0.254218
C -1.555711 -2.100058 -0.069817
H -0.132399 -2.176010 -3.672116
H 0.454899 -2.348551 0.617737
H -1.959730 -2.087251 0.939645
N 1.802938 -2.437952 -1.717060
C 2.450579 -1.120539 -1.352234
H 3.529967 -1.231122 -1.463250

H	2.079981	-0.352904	-2.030568
H	2.204683	-0.882408	-0.314887
C	2.194803	-2.784988	-3.123922
H	3.273119	-2.932137	-3.134685
H	1.687080	-3.702493	-3.419516
H	1.939193	-1.961227	-3.786808
C	2.342125	-3.516917	-0.810633
H	3.399366	-3.640045	-1.041138
H	2.234549	-3.204904	0.227464
H	1.794874	-4.437913	-1.008119
I	2.162281	-1.294224	2.728734
C	-5.033423	1.113110	0.621597
C	-4.608666	1.828774	-0.498476
C	-3.257159	2.091910	-0.688924
C	-2.332906	1.630109	0.248121
C	-2.744882	0.923730	1.377710
C	-4.101167	0.668859	1.559102
H	-2.948631	2.651394	-1.564253
H	-2.042697	0.563914	2.117778
H	-4.439037	0.113740	2.428317
N	-0.889303	1.916072	0.007268
C	0.011092	1.283237	1.032647
H	1.038389	1.513915	0.744212
H	-0.205200	1.701723	2.014779
H	-0.139300	0.204423	1.027504
C	-0.645620	3.403368	0.036447
H	0.422326	3.560344	-0.128430
H	-1.226573	3.875969	-0.753139
H	-0.951977	3.779321	1.012062
C	-0.479735	1.375082	-1.338045
H	0.587700	1.574569	-1.457399
H	-0.688127	0.304828	-1.345065
H	-1.045677	1.873472	-2.121700
I	3.363832	2.460379	-0.826103
H	-2.535844	-1.833714	-3.310723
H	-5.331100	2.177289	-1.230651
C	-6.477735	0.812511	0.792643
O	-6.922054	0.173514	1.723458
H	-7.144412	1.205919	0.004188
C	-3.855261	-1.723304	-0.942666
O	-4.638476	-1.450642	-1.827685
H	-4.194367	-1.826594	0.104837

4CHO_ionpair_displ_conf2
Electronic energy = -530.612467
Thermal correction to Gibbs free energy (25°C) = 0.053019
Thermal correction to Gibbs free energy (80°C) = 0.067525
qh-G(25°C) = -530.422402

qh-G(80°C) = -530.431972

Geometry:

C	-3.232106	-1.040968	-0.000089
C	-1.855223	-1.288406	0.000274
C	-0.957974	-0.232049	0.000366
C	-1.447406	1.077780	0.000069
C	-2.811745	1.342295	-0.000354
C	-3.703190	0.268732	-0.000420
H	-3.207999	2.348187	-0.000597
H	-4.771698	0.464011	-0.000744
H	-1.494470	-2.311883	0.000511
N	-0.444925	2.185466	0.000120
C	0.421207	2.075789	-1.230599
H	1.110980	2.919687	-1.227875
H	-0.227012	2.109963	-2.105494
H	0.981383	1.141803	-1.190054
C	0.422114	2.074965	1.230127
H	1.110998	2.919585	1.228093
H	0.983326	1.141675	1.187807
H	-0.225547	2.107235	2.105504
C	-1.077302	3.544810	0.000838
H	-0.270825	4.275853	0.001042
H	-1.680113	3.661494	0.900160
H	-1.680304	3.662391	-0.898233
H	0.109443	-0.440247	0.000591
I	3.006763	-0.843941	-0.000076
C	-4.203585	-2.162813	-0.000102
O	-3.880193	-3.332528	0.000296
H	-5.269345	-1.869737	-0.000549

4CHO_ionpair_displ_conf2_DMSO
Electronic energy = -1083.717484
Thermal correction to Gibbs free energy (25°C) = 0.068371
Thermal correction to Gibbs free energy (80°C) = 0.087672
qh-G(25°C) = -1083.453681
qh-G(80°C) = -1083.465830

Geometry:

C	-4.493047	-0.737664	0.085290
C	-3.535849	0.282959	0.065727
C	-2.187017	-0.029578	0.008090
C	-1.797924	-1.372738	-0.030648
C	-2.735179	-2.398332	-0.010437
C	-4.090282	-2.069493	0.047836
H	-2.451052	-3.441070	-0.038482
H	-4.830801	-2.864035	0.063848
H	-3.857164	1.319137	0.095832

N -0.337859 -1.661758 -0.094359
C 0.346109 -1.083543 1.121233
H 1.395843 -1.375320 1.060113
H -0.133042 -1.501949 2.006354
H 0.251062 0.002486 1.098681
C 0.246083 -1.038181 -1.339288
H 1.299210 -1.320812 -1.368840
H 0.144850 0.045130 -1.274093
H -0.296484 -1.432744 -2.198360
C -0.022604 -3.127599 -0.133424
H 1.063161 -3.208807 -0.183644
H -0.477162 -3.568193 -1.019905
H -0.396624 -3.598245 0.774945
H -1.452632 0.772386 -0.006021
I 0.675895 2.829325 -0.059954
C -5.940196 -0.417650 0.145514
O -6.378582 0.713702 0.182031
H -6.620754 -1.288535 0.155445
O 3.079122 -2.147474 -0.200057
S 4.263469 -1.193956 -0.416094
C 3.915713 0.241748 0.619177
H 4.769809 0.921301 0.583106
H 3.030854 0.738817 0.212268
H 3.728113 -0.096083 1.641415
C 5.616182 -1.881916 0.558368
H 6.453187 -1.180976 0.551207
H 5.259523 -2.059638 1.575082
H 5.910896 -2.819384 0.086185

4Cl_SnArProduct_GEN
Electronic energy = -997.874970
Thermal correction to Gibbs free energy (25°C) = 0.046711
Thermal correction to Gibbs free energy (80°C) = 0.059556
qh-G(25°C) = -997.732516
qh-G(80°C) = -997.741019

Geometry:
C -3.283523 -1.332388 0.957303
C -2.122145 -0.563760 0.911132
C -2.059598 0.514520 0.028761
C -3.136331 0.838043 -0.791438
C -4.295709 0.065127 -0.729312
C -4.372352 -1.022834 0.140093
H -3.337755 -2.173027 1.641620
H -1.274777 -0.794582 1.549121
H -3.054466 1.686126 -1.463146
H -5.137729 0.313880 -1.367219
H -5.273955 -1.624573 0.182922

O -0.959664 1.350024 -0.014216
C 0.300383 0.799736 -0.021573
C 1.302878 1.513574 0.631472
C 2.614120 1.046174 0.606356
H 1.048507 2.429292 1.154047
C 2.901370 -0.135824 -0.069404
H 3.401744 1.592988 1.112860
C 0.595127 -0.379393 -0.706582
H -0.188459 -0.925779 -1.220673
C 1.904786 -0.851732 -0.726253
H 2.147471 -1.766967 -1.254831
Cl 4.544276 -0.734412 -0.094370

4Cl_TMA_DMSO_tsopt_GEN
Electronic energy = -1418.393687
Thermal correction to Gibbs free energy (25°C) = 0.059767
Thermal correction to Gibbs free energy (80°C) = 0.076979
qh-G(25°C) = -1418.146858
qh-G(80°C) = -1418.157737

Geometry:
C -3.364989 -0.636178 -0.024665
C -3.061119 0.153800 -1.124516
C -1.955117 1.001612 -1.079424
C -1.149962 1.062512 0.063097
C -1.476001 0.253787 1.162176
C -2.577950 -0.591640 1.122295
H -3.681066 0.119199 -2.013368
H -1.749501 1.614727 -1.946966
H -0.877459 0.267373 2.065693
H -2.819706 -1.211801 1.978106
N 0.030566 1.877662 0.113564
C 0.181439 2.646690 1.363833
H 1.115876 3.207626 1.313751
H -0.653520 3.343944 1.488968
H 0.228907 1.982273 2.225192
C 0.244342 2.741787 -1.054648
H 1.183572 3.278357 -0.915443
H 0.322526 2.138763 -1.960918
H -0.566437 3.470122 -1.167356
C 1.635175 0.556951 0.062739
H 1.244774 0.089572 0.959335
H 1.284956 0.235065 -0.911111
H 2.299947 1.409115 0.148185
S 3.350766 -0.838104 0.066186
O 4.076607 -0.893291 1.374911
C 4.494980 -0.423203 -1.246030
H 4.887944 0.569569 -1.024667

H 5.295648 -1.164772 -1.244154
H 3.957119 -0.421844 -2.195442
C 2.814063 -2.482433 -0.398205
H 3.691703 -3.131122 -0.406190
H 2.097893 -2.810420 0.355903
H 2.345033 -2.438626 -1.382590
Cl -4.750379 -1.696287 -0.076474

4Cl_TMA_I_TS
Electronic energy = -876.837741
Thermal correction to Gibbs free energy (25°C) = 0.052525
Thermal correction to Gibbs free energy (80°C) = 0.066762
qh-G(25°C) = -876.671277
qh-G(80°C) = -876.680816

Geometry:
C 3.675569 -0.731835 0.022509
C 3.462692 0.156924 1.066470
C 2.380744 1.035164 1.012570
C 1.509055 1.028039 -0.081497
C 1.741991 0.116205 -1.122381
C 2.818984 -0.759679 -1.074602
H 4.134097 0.175631 1.917834
H 2.243974 1.725931 1.834371
H 1.084521 0.071790 -1.983417
H 2.987195 -1.461559 -1.883822
N 0.363390 1.885500 -0.140984
C 0.224551 2.617342 -1.412437
H -0.714348 3.173617 -1.391883
H 1.056766 3.317948 -1.546151
H 0.195448 1.928625 -2.255234
C 0.202768 2.795616 0.997610
H -0.729825 3.346237 0.865918
H 0.140885 2.226868 1.927080
H 1.029597 3.513271 1.060218
C -1.340362 0.646004 -0.026737
Cl 5.031145 -1.831719 0.082079
I -3.463920 -0.820140 0.091331
H -1.949531 1.536213 -0.056012
H -1.038871 0.166311 -0.944958
H -0.981272 0.264589 0.918034

4Cl_TMA_SN2_tsopt_GEN
Electronic energy = -1172.210279
Thermal correction to Gibbs free energy (25°C) = 0.059241
Thermal correction to Gibbs free energy (80°C) = 0.076489
qh-G(25°C) = -1171.950909

qh-G(80°C) = -1171.961668

Geometry:

C	-4.156511	-0.623496	0.061313
C	-3.935175	0.325624	-0.926542
C	-2.738648	1.041864	-0.936837
C	-1.766397	0.810817	0.038856
C	-2.007508	-0.155806	1.024006
C	-3.198291	-0.871550	1.039716
H	-2.595741	1.780874	-1.713956
H	-1.273584	-0.369437	1.792207
H	-3.376105	-1.617376	1.806198
N	-0.492325	1.497046	0.024435
C	0.842101	0.211886	-0.392535
H	1.625983	0.959646	-0.380819
H	0.646591	-0.375774	0.491990
H	0.369550	-0.068786	-1.323741
C	-0.126278	2.070329	1.339333
H	0.864307	2.519755	1.251588
H	-0.851607	2.833493	1.635401
H	-0.085065	1.290490	2.097718
C	-0.368963	2.531894	-1.017339
H	-0.482759	2.082278	-2.004578
H	-1.113027	3.321872	-0.877899
H	0.628945	2.965477	-0.944633
C	3.422563	-0.845870	-0.419739
C	3.640109	-0.342586	0.889486
C	4.576396	-1.081734	-1.211960
C	4.927720	-0.098081	1.365647
H	2.785875	-0.164819	1.537301
C	5.855454	-0.841125	-0.721938
H	4.432482	-1.463919	-2.219310
C	6.049261	-0.343173	0.571521
H	5.054147	0.283203	2.376028
H	6.714333	-1.040221	-1.358278
H	7.048761	-0.153874	0.949138
O	2.221719	-1.088569	-0.896071
H	-4.686811	0.514835	-1.684574
Cl	-5.654632	-1.517791	0.079402

4Cl_TMA_SnAr_tsopt_GEN
Electronic energy = -1172.214786
Thermal correction to Gibbs free energy (25°C) = 0.056568
Thermal correction to Gibbs free energy (80°C) = 0.073386
qh-G(25°C) = -1171.952673
qh-G(80°C) = -1171.963042

Geometry:

C	-2.034136	-1.492753	0.086226
C	-1.292566	-1.038218	1.169221
C	-0.592025	0.158975	1.098271
C	-0.546877	0.930386	-0.103267
C	-1.387990	0.466851	-1.167874
C	-2.080605	-0.727541	-1.079516
H	-1.257035	-1.616224	2.088036
H	-0.000744	0.459171	1.955480
H	-2.666907	-1.064008	-1.929447
N	-0.604034	2.489200	0.126599
C	-2.004155	2.824108	0.539052
H	-2.044733	3.888038	0.774078
H	-2.678883	2.587604	-0.282560
H	-2.261981	2.227561	1.413075
C	-0.282321	3.280246	-1.105164
H	-0.469590	4.330436	-0.879070
H	0.758103	3.115148	-1.363837
H	-0.935727	2.968219	-1.916736
C	0.321672	2.928625	1.218271
H	-0.033997	2.538598	2.169749
H	1.318641	2.560955	0.989043
H	0.306629	4.018287	1.249833
O	1.116843	1.038282	-0.710354
C	1.908959	0.001218	-0.421717
C	1.524526	-1.334570	-0.652708
C	3.185748	0.241011	0.121505
C	2.395278	-2.381284	-0.356223
H	0.539720	-1.538649	-1.063066
C	4.055580	-0.811862	0.399594
C	3.666754	-2.132556	0.166337
H	2.078056	-3.403967	-0.542020
H	5.040531	-0.598724	0.805936
H	4.341586	-2.953227	0.387574
H	3.483739	1.269836	0.305766
H	-1.430451	1.018181	-2.099863
Cl	-2.930618	-3.000806	0.191919

4Cl_TMA_bromide_tsopt
Electronic energy = -3436.979251
Thermal correction to Gibbs free energy (25°C) = 0.051364
Thermal correction to Gibbs free energy (80°C) = 0.065399
qh-G(25°C) = -3436.810988
qh-G(80°C) = -3436.820357

Geometry:

C	-3.157378	-0.574669	-0.014731
C	-2.831411	0.230722	-1.096598
C	-1.680178	1.016163	-1.049065

C -0.850530 0.998777 0.077661
C -1.198971 0.173035 1.157435
C -2.346397 -0.609105 1.116021
H -3.468359 0.255528 -1.973750
H -1.456715 1.641495 -1.903415
H -0.578757 0.121880 2.044694
H -2.603735 -1.243622 1.956760
N 0.372466 1.745020 0.127539
C 0.586542 2.476502 1.389512
H 1.568887 2.949944 1.351023
H -0.182205 3.245228 1.524632
H 0.570181 1.797340 2.240062
C 0.616865 2.622180 -1.023423
H 1.582861 3.108541 -0.882488
H 0.657538 2.034785 -1.942250
H -0.157379 3.392887 -1.112959
C 1.900800 0.338198 0.015751
Cl -4.599806 -1.557054 -0.070852
H 2.619209 1.143859 0.001270
H 1.575803 -0.079188 0.956280
H 1.470464 -0.021818 -0.906392
Br 3.650160 -1.260654 -0.127918

4Cl_TMA_chloride_tsopt
Electronic energy = -1325.658048
Thermal correction to Gibbs free energy (25°C) = 0.050234
Thermal correction to Gibbs free energy (80°C) = 0.064053
qh-G(25°C) = -1325.488356
qh-G(80°C) = -1325.497530

Geometry:
C -2.639732 -0.314151 -0.017022
C -2.183298 0.466873 -1.069243
C -0.903422 1.017804 -1.013316
C -0.076248 0.790725 0.091837
C -0.558454 -0.005599 1.141605
C -1.833449 -0.555289 1.091597
H -2.816747 0.654544 -1.929131
H -0.577658 1.630772 -1.843293
H 0.052580 -0.214631 2.011947
H -2.194155 -1.169473 1.909124
N 1.267214 1.292612 0.146853
C 1.626477 1.912667 1.436100
H 2.676540 2.206488 1.396471
H 1.008604 2.797548 1.623242
H 1.501952 1.205754 2.254511
C 1.650250 2.170079 -0.966557
H 2.692654 2.459873 -0.828741

H 1.563418 1.636152 -1.914318
H 1.031788 3.074361 -0.995081
C 2.498722 -0.348408 -0.059368
Cl -4.243248 -1.002218 -0.080535
H 3.352486 0.312442 -0.056533
H 2.117637 -0.743237 0.869897
H 1.997015 -0.583673 -0.985936
Cl 3.845502 -2.122688 -0.293225

4Cl_TMA_gen
Electronic energy = -865.357117
Thermal correction to Gibbs free energy (25°C) = 0.044098
Thermal correction to Gibbs free energy (80°C) = 0.056363
qh-G(25°C) = -865.180795
qh-G(80°C) = -865.188917

Geometry:
C -0.722846 0.032406 -0.004518
C -0.026189 1.234883 -0.004134
C 1.368430 1.221276 -0.002439
C 2.042127 0.007623 -0.001318
C 1.347638 -1.199491 -0.001901
C -0.041063 -1.184366 -0.003015
H -0.525452 2.193922 -0.004712
H 1.881529 -2.142558 -0.000997
H -0.564357 -2.133630 -0.002094
N -2.216181 -0.000007 0.000223
C -2.711945 -0.739796 -1.216024
H -2.361980 -1.768738 -1.181037
H -3.800943 -0.717358 -1.202895
H -2.324566 -0.235943 -2.100459
C -2.699238 -0.694759 1.247658
H -3.788488 -0.687762 1.237437
H -2.332278 -1.718385 1.253892
H -2.316264 -0.148859 2.108860
C -2.825774 1.371620 -0.022524
H -2.518480 1.915793 0.868818
H -2.513793 1.888301 -0.928648
H -3.907073 1.245727 -0.022767
H 1.917353 2.155672 -0.001591
Cl 3.781248 -0.008383 0.001407

4Cl_aniline_gen
Electronic energy = -825.633147
Thermal correction to Gibbs free energy (25°C) = 0.042550
Thermal correction to Gibbs free energy (80°C) = 0.053956
qh-G(25°C) = -825.499539

qh-G(80°C) = -825.507331

Geometry:

C	-1.051152	-0.000015	-0.088672
C	-0.317580	1.207317	-0.054761
C	1.072645	1.205296	-0.018917
C	1.765104	-0.000004	-0.004782
C	1.072656	-1.205308	-0.018907
C	-0.317571	-1.207342	-0.054750
H	-0.826802	2.162646	-0.057293
H	1.612855	-2.146077	0.003713
H	-0.826777	-2.162678	-0.057276
N	-2.430519	0.000001	-0.161761
C	-3.139468	-1.243276	0.088232
H	-4.210380	-1.060640	0.007104
H	-2.874441	-1.998664	-0.657151
H	-2.927550	-1.651806	1.087363
C	-3.139414	1.243304	0.088247
H	-2.927491	1.651801	1.087390
H	-2.874333	1.998693	-0.657116
H	-4.210332	1.060721	0.007098
H	1.612839	2.146068	0.003694
Cl	3.517336	0.000006	0.044157

4Cl_bromide_ionpair_gen
Electronic energy = -3437.031941
Thermal correction to Gibbs free energy (25°C) = 0.050470
Thermal correction to Gibbs free energy (80°C) = 0.064214
qh-G(25°C) = -3436.858982
qh-G(80°C) = -3436.868101

Geometry:

Br	-3.136476	-1.482932	-0.000079
N	-0.517362	1.961053	0.000047
C	0.668347	1.053176	0.000019
C	1.965177	1.553485	-0.000261
C	3.041400	0.665956	-0.000251
C	2.800912	-0.701262	-0.000007
C	1.502037	-1.203385	0.000255
C	-1.351273	1.697984	1.229223
H	-2.176519	2.410028	1.229689
H	-0.719020	1.840816	2.104969
H	-1.740725	0.681010	1.181157
C	-1.351732	1.697615	-1.228724
H	-2.177024	2.409608	-1.229035
H	-1.741139	0.680646	-1.180272
H	-0.719856	1.840282	-2.104764
C	-0.136997	3.410375	-0.000259

H -1.060132 3.987319 -0.000201
H 0.436103 3.632128 -0.899308
H 0.436360 3.632437 0.898543
C 0.429322 -0.320591 0.000292
H -0.581314 -0.723459 0.000548
H 2.179220 2.613590 -0.000489
H 4.056243 1.046147 -0.000432
H 1.325937 -2.273078 0.000462
Cl 4.145699 -1.807197 -0.000010

4Cl_chloride_ionpair_gen
Electronic energy = -1325.710119
Thermal correction to Gibbs free energy (25°C) = 0.049305
Thermal correction to Gibbs free energy (80°C) = 0.062900
qh-G(25°C) = -1325.535910
qh-G(80°C) = -1325.544884

Geometry:
Cl 2.908781 2.627283 0.000046
N 1.523251 -1.288174 -0.000010
C 0.117008 -0.785926 -0.000028
C -0.964867 -1.658820 0.000029
C -2.261215 -1.143324 0.000036
C -2.450108 0.231876 -0.000023
C -1.367105 1.107378 -0.000109
C 2.236681 -0.781624 1.228887
H 3.240792 -1.205977 1.229618
H 1.678591 -1.112450 2.104020
H 2.295031 0.305799 1.178706
C 2.236524 -0.782062 -1.229161
H 3.240639 -1.206400 -1.229861
H 2.294856 0.305372 -1.179365
H 1.678320 -1.113198 -2.104105
C 1.602171 -2.784113 0.000273
H 2.656575 -3.054147 0.000403
H 1.122989 -3.169556 -0.898399
H 1.122820 -3.169206 0.899006
C -0.075266 0.595533 -0.000110
H 0.765536 1.287106 -0.000213
H -0.845247 -2.733637 0.000104
H -3.111166 -1.815766 0.000093
H -1.527212 2.179549 -0.000160
Cl -4.068676 0.874259 0.000040

4F_SnAr_Product_gen_2
Electronic energy = -637.519605
Thermal correction to Gibbs free energy (25°C) = 0.045544

Thermal correction to Gibbs free energy (80°C) = 0.058110
qh-G(25°C) = -637.374990
qh-G(80°C) = -637.383287

Geometry:

C	2.812477	1.426750	-0.786786
C	1.621617	0.709401	-0.668337
C	1.628243	-0.502788	0.021849
C	2.804252	-1.002327	0.580789
C	3.985888	-0.277048	0.449371
C	3.996042	0.941702	-0.231601
H	2.809461	2.370240	-1.323445
H	0.704158	1.086866	-1.107222
H	2.775918	-1.949636	1.109089
H	4.900603	-0.665477	0.885818
H	4.917084	1.506431	-0.328832
O	0.509311	-1.298272	0.143596
C	-0.737562	-0.706250	0.100504
C	-1.083059	0.299320	1.003516
C	-2.368679	0.832325	0.978015
H	-0.350081	0.661528	1.717285
C	-3.274866	0.332990	0.053215
H	-2.670709	1.614827	1.664977
C	-1.659605	-1.194070	-0.819742
H	-1.361978	-1.978295	-1.507218
C	-2.952528	-0.671318	-0.844571
H	-3.695349	-1.030430	-1.547817
F	-4.526323	0.848666	0.030141

4F_TMA_DMSO_tsopt_GEN
Electronic energy = -1058.038673
Thermal correction to Gibbs free energy (25°C) = 0.058623
Thermal correction to Gibbs free energy (80°C) = 0.075566
qh-G(25°C) = -1057.789699
qh-G(80°C) = -1057.800385

Geometry:

C	3.553394	-1.189905	0.070530
C	3.398157	-0.312283	1.127027
C	2.402505	0.661934	1.051537
C	1.574751	0.749374	-0.072849
C	1.759795	-0.161603	-1.124328
C	2.751041	-1.133642	-1.058865
H	4.045949	-0.382576	1.993399
H	2.298326	1.349256	1.880692
H	1.134791	-0.128426	-2.009653
H	2.899119	-1.839343	-1.868301
N	0.501608	1.703906	-0.152071

C 0.466104 2.463543 -1.415795
H -0.425286 3.093586 -1.417513
H 1.356347 3.095165 -1.507799
H 0.413870 1.791326 -2.270748
C 0.391164 2.613712 0.994555
H -0.477515 3.255327 0.840220
H 0.243749 2.044359 1.914181
H 1.281054 3.245655 1.092752
C -1.261482 0.597396 -0.080583
H -0.975997 0.138184 -1.019112
H -0.902958 0.181651 0.854080
H -1.813288 1.530682 -0.088869
S -3.131569 -0.567670 -0.042549
O -4.041830 -0.310261 -1.202941
C -4.009469 -0.293350 1.493815
H -4.289391 0.760015 1.519675
H -4.896009 -0.929687 1.491822
H -3.348613 -0.537998 2.327104
C -2.739446 -2.312158 0.050240
H -3.677752 -2.865744 0.114494
H -2.200944 -2.568984 -0.862440
H -2.115379 -2.487656 0.928361
F 4.517705 -2.133937 0.138305

4F_TMA_I_TS
Electronic energy = -516.482520
Thermal correction to Gibbs free energy (25°C) = 0.051367
Thermal correction to Gibbs free energy (80°C) = 0.065342
qh-G(25°C) = -516.313983
qh-G(80°C) = -516.323337

Geometry:
C 3.816379 -1.289803 -0.051165
C 3.005314 -1.204898 1.070144
C 2.046807 -0.200134 1.125795
C 1.902029 0.717489 0.072953
C 2.738467 0.599627 -1.042875
C 3.700558 -0.408373 -1.109221
H 3.120089 -1.916798 1.879817
H 1.410392 -0.148051 2.001897
H 2.665920 1.290210 -1.872592
H 4.354054 -0.500882 -1.969503
N 0.869203 1.709901 0.141348
C 0.812103 2.633359 -0.995722
H -0.037819 3.301974 -0.851446
H 1.725712 3.235575 -1.072018
H 0.661535 2.080074 -1.924490
C 0.828656 2.451337 1.413142

H -0.022971 3.133562 1.388998
H 0.697003 1.771973 2.253962
H 1.750628 3.027309 1.554626
C -0.967055 0.661823 0.033874
F 4.748760 -2.267746 -0.109589
I -3.200243 -0.615386 -0.086788
H -1.484089 1.608382 0.088785
H -0.652748 0.269424 -0.922000
H -0.697657 0.140530 0.939056

4F_TMA_SN2_tsopt_GEN
Electronic energy = -811.855475
Thermal correction to Gibbs free energy (25°C) = 0.058065
Thermal correction to Gibbs free energy (80°C) = 0.075044
qh-G(25°C) = -811.593817
qh-G(80°C) = -811.604384

Geometry:
C -3.769259 -1.625518 0.061524
C -4.093588 -0.495245 -0.665177
C -3.239490 0.606434 -0.611132
C -2.078454 0.565948 0.165243
C -1.778116 -0.598425 0.885583
C -2.623945 -1.700369 0.839087
H -3.508402 1.487162 -1.178990
H -0.881988 -0.666252 1.491297
H -2.399407 -2.604187 1.393728
N -1.142723 1.671524 0.199007
C 0.456138 1.095649 -0.657341
H 0.916922 2.068301 -0.558252
H 0.643862 0.331875 0.086032
H -0.058383 0.839033 -1.573097
C -0.766037 2.066697 1.574433
H -0.033068 2.872678 1.511152
H -1.646181 2.411959 2.124345
H -0.312080 1.228517 2.100937
C -1.576395 2.858589 -0.557351
H -1.704410 2.601898 -1.609952
H -2.509320 3.264650 -0.154757
H -0.795155 3.615017 -0.475844
C 3.000582 -0.022270 -0.795556
C 3.756775 -1.152140 -1.202970
C 3.305826 0.523721 0.478564
C 4.754941 -1.687263 -0.396035
H 3.536926 -1.588673 -2.173718
C 4.303435 -0.028183 1.281599
H 2.764465 1.400779 0.822855
C 5.039787 -1.135900 0.858340

H 5.316340 -2.549734 -0.746940
H 4.511253 0.420848 2.249792
H 5.817105 -1.558516 1.486499
O 2.067567 0.475701 -1.576596
H -4.998721 -0.471501 -1.261110
F -4.594938 -2.693387 0.014762

4F_TMA_SnAr_tsopt_GEN
Electronic energy = -811.855379
Thermal correction to Gibbs free energy (25°C) = 0.055460
Thermal correction to Gibbs free energy (80°C) = 0.072037
qh-G(25°C) = -811.591497
qh-G(80°C) = -811.601680

Geometry:
C 1.039090 2.604257 0.152918
C 0.655357 1.829647 1.231474
C 0.654753 0.441494 1.128809
C 0.984767 -0.220788 -0.093914
C 1.437192 0.629009 -1.156927
C 1.432735 2.010480 -1.038471
H 0.355269 2.303356 2.161398
H 0.310744 -0.137365 1.978035
H 1.738291 2.628470 -1.877369
N 1.878531 -1.526258 0.101720
C 3.247415 -1.064470 0.488283
H 3.860683 -1.942826 0.692508
H 3.668902 -0.486695 -0.333325
H 3.165826 -0.438153 1.375795
C 1.999445 -2.348537 -1.143701
H 2.720628 -3.143044 -0.947928
H 1.025150 -2.758017 -1.388780
H 2.369206 -1.724106 -1.954185
C 1.355029 -2.402828 1.194773
H 1.452567 -1.885492 2.147182
H 0.315437 -2.636427 0.979172
H 1.960497 -3.309472 1.217652
O -0.356124 -1.187887 -0.664983
C -1.580440 -0.724001 -0.391710
C -1.949943 0.622277 -0.582307
C -2.554175 -1.619726 0.089650
C -3.250921 1.040858 -0.308632
H -1.211920 1.331204 -0.945304
C -3.856202 -1.193995 0.344992
C -4.215504 0.141430 0.151205
H -3.514996 2.083564 -0.463586
H -4.591946 -1.909110 0.702598
H -5.227918 0.475374 0.354370

H -2.269081 -2.656945 0.244316
H 1.718950 0.193397 -2.108379
F 1.051267 3.969840 0.263889

4F_TMA_bromide_tsopt
Electronic energy = -3076.624084
Thermal correction to Gibbs free energy (25°C) = 0.050273
Thermal correction to Gibbs free energy (80°C) = 0.064033
qh-G(25°C) = -3076.453858
qh-G(80°C) = -3076.463020

Geometry:
C 3.388824 -1.102770 -0.054052
C 2.554918 -1.123140 1.053621
C 1.504847 -0.214845 1.114316
C 1.291836 0.710729 0.080783
C 2.152859 0.700454 -1.022207
C 3.207379 -0.210480 -1.093619
H 2.723621 -1.839933 1.849224
H 0.852345 -0.243897 1.979503
H 2.027634 1.399877 -1.838289
H 3.879671 -0.220627 -1.944055
N 0.164270 1.597279 0.155317
C 0.028102 2.528021 -0.970025
H -0.880903 3.112764 -0.821852
H 0.881124 3.214153 -1.031380
H -0.064801 1.974963 -1.906395
C 0.052890 2.320268 1.434685
H -0.876632 2.892492 1.429695
H 0.021672 1.624563 2.271574
H 0.898910 3.004432 1.565606
C -1.538779 0.395121 0.018715
F 4.411346 -1.985335 -0.116884
H -2.142644 1.289731 0.013021
H -1.149859 -0.003496 -0.906040
H -1.270806 -0.068645 0.955033
Br -3.474531 -0.956270 -0.143548

4F_TMA_chloride_tsopt
Electronic energy = -965.302919
Thermal correction to Gibbs free energy (25°C) = 0.048870
Thermal correction to Gibbs free energy (80°C) = 0.062378
qh-G(25°C) = -965.130861
qh-G(80°C) = -965.139801

Geometry:
C 2.942877 -0.739757 -0.058985

C 2.112323 -0.963769 1.028464
C 0.905392 -0.278041 1.097977
C 0.532791 0.627985 0.093005
C 1.395484 0.826956 -0.990388
C 2.607623 0.140481 -1.070254
H 2.404757 -1.664656 1.802059
H 0.258368 -0.465450 1.947326
H 1.149679 1.520157 -1.783965
H 3.282067 0.293544 -1.905144
N -0.747040 1.277962 0.173420
C -1.034284 2.219046 -0.915160
H -2.040475 2.614080 -0.768177
H -0.324927 3.054713 -0.922234
H -1.001956 1.703233 -1.876418
C -1.014510 1.910452 1.478049
H -2.032955 2.302880 1.469931
H -0.934800 1.183319 2.284481
H -0.311313 2.730851 1.659644
C -2.177101 -0.199744 -0.057119
F 4.117840 -1.404951 -0.129879
H -2.939065 0.564185 -0.021982
H -1.715133 -0.463775 -0.996400
H -1.833662 -0.661874 0.855264
Cl -3.730319 -1.780826 -0.318997

4F_TMA_gen
Electronic energy = -505.002912
Thermal correction to Gibbs free energy (25°C) = 0.043040
Thermal correction to Gibbs free energy (80°C) = 0.055050
qh-G(25°C) = -504.824524
qh-G(80°C) = -504.832464

Geometry:
C -0.311941 0.031757 -0.003124
C 0.385845 1.234767 -0.002686
C 1.780257 1.221396 -0.001282
C 2.434669 0.003181 -0.000428
C 1.755924 -1.206764 -0.000884
C 0.367398 -1.187528 -0.001864
H -0.114203 2.193313 -0.003073
H 2.304182 -2.141304 -0.000114
H -0.159492 -2.134775 -0.001173
N -1.805732 0.001019 0.000031
C -2.301452 -0.731104 -1.220556
H -1.949350 -1.759555 -1.192969
H -3.390522 -0.710642 -1.207136
H -1.915085 -0.220728 -2.101697
C -2.292795 -0.698705 1.242831

H -3.382051 -0.689072 1.231274
H -1.928804 -1.723397 1.244816
H -1.909620 -0.158305 2.107382
C -2.413540 1.373404 -0.016478
H -2.105011 1.913379 0.877010
H -2.101306 1.893569 -0.920502
H -3.495030 1.249088 -0.016894
H 2.346128 2.145358 -0.000548
F 3.779344 -0.011832 0.001136

4F_aniline_gen
Electronic energy = -465.276443
Thermal correction to Gibbs free energy (25°C) = 0.041260
Thermal correction to Gibbs free energy (80°C) = 0.052373
qh-G(25°C) = -465.140635
qh-G(80°C) = -465.148208

Geometry:
C -0.618202 0.000009 -0.104664
C 0.112729 1.207231 -0.060152
C 1.504263 1.207734 -0.006035
C 2.179416 -0.000003 0.016322
C 1.504256 -1.207730 -0.006037
C 0.112717 -1.207213 -0.060154
H -0.397645 2.161730 -0.069427
H 2.058054 -2.140036 0.025251
H -0.397665 -2.161710 -0.069433
N -2.004573 0.000003 -0.203237
C -2.706784 -1.236536 0.097490
H -2.496703 -1.603379 1.113891
H -3.778675 -1.063995 0.003773
H -2.435763 -2.020010 -0.615265
C -2.706826 1.236519 0.097488
H -3.778710 1.063938 0.003775
H -2.496758 1.603374 1.113889
H -2.435840 2.020002 -0.615269
H 2.058078 2.140030 0.025256
F 3.538248 -0.000003 0.073408

4F_bromide_ionpair_gen
Electronic energy = -3076.677711
Thermal correction to Gibbs free energy (25°C) = 0.049288
Thermal correction to Gibbs free energy (80°C) = 0.062792
qh-G(25°C) = -3076.502579
qh-G(80°C) = -3076.511524

Geometry:

Br -3.118162 -0.965459 0.000167
N 0.187447 1.816407 -0.000126
C 1.149792 0.673959 -0.000063
C 2.525061 0.883041 0.000519
C 3.385739 -0.214687 0.000579
C 2.841844 -1.486326 0.000007
C 1.473048 -1.711323 -0.000598
C -0.684381 1.740262 1.228221
H -1.338988 2.611915 1.226831
H -0.036716 1.745773 2.104193
H -1.282045 0.829905 1.181123
C -0.684172 1.740351 -1.228596
H -1.338847 2.611948 -1.227176
H -1.281807 0.829977 -1.181692
H -0.036387 1.746011 -2.104476
C 0.872128 3.148945 -0.000050
H 0.096287 3.912449 -0.000292
H 1.480390 3.241154 -0.898537
H 1.479878 3.241315 0.898767
C 0.618113 -0.616317 -0.000611
H -0.456003 -0.789241 -0.001148
H 2.961713 1.872439 0.000961
H 4.460936 -0.080434 0.001043
H 1.086254 -2.723842 -0.001047
F 3.671760 -2.546733 0.000003

4F_chloride_ionpair_gen
Electronic energy = -965.355821
Thermal correction to Gibbs free energy (25°C) = 0.048352
Thermal correction to Gibbs free energy (80°C) = 0.061708
qh-G(25°C) = -965.179686
qh-G(80°C) = -965.188488

Geometry:
Cl -3.010844 -2.201639 -0.000014
N -0.923199 1.392333 0.000016
C 0.369179 0.643481 -0.000078
C 1.590403 1.309219 -0.000059
C 2.774372 0.572090 -0.000051
C 2.697145 -0.808698 -0.000003
C 1.487376 -1.487716 -0.000010
C -1.718139 1.024719 1.228048
H -2.629959 1.622288 1.226113
H -1.111650 1.250396 2.104406
H -1.970967 -0.034555 1.178701
C -1.718379 1.024556 -1.227781
H -2.630237 1.622065 -1.225699
H -1.971158 -0.034721 -1.178283

H -1.112100 1.250191 -2.104301
C -0.727979 2.877728 -0.000125
H -1.714902 3.336384 -0.000205
H -0.186045 3.169241 -0.898551
H -0.186088 3.169426 0.898257
C 0.309185 -0.751016 -0.000070
H -0.643050 -1.278335 -0.000024
H 1.661469 2.388056 -0.000049
H 3.738926 1.065795 -0.000063
H 1.470494 -2.571448 0.000037
F 3.839225 -1.522159 0.000061

4Me_SnAr_Product_gen_2
Electronic energy = -577.607800
Thermal correction to Gibbs free energy (25°C) = 0.047566
Thermal correction to Gibbs free energy (80°C) = 0.060736
qh-G(25°C) = -577.428394
qh-G(80°C) = -577.436995

Geometry:
C -2.816073 1.466240 -0.758232
C -1.636036 0.731435 -0.637356
C -1.667082 -0.495870 0.025828
C -2.858996 -0.990957 0.556069
C -4.029131 -0.247863 0.423252
C -4.014155 0.985346 -0.231425
H -2.792795 2.421037 -1.274147
H -0.707235 1.106726 -1.053432
H -2.851140 -1.949824 1.063956
H -4.955075 -0.633878 0.837768
H -4.926459 1.563878 -0.330061
O -0.562284 -1.307474 0.148585
C 0.696032 -0.733309 0.101625
C 1.601572 -1.207477 -0.837456
C 2.899961 -0.692337 -0.855481
H 1.288546 -1.972417 -1.540696
C 3.300565 0.295099 0.047052
H 3.608675 -1.066419 -1.589285
C 1.069757 0.245796 1.022642
H 0.350799 0.600927 1.754985
C 2.364008 0.753157 0.985098
H 2.656692 1.515509 1.702670
C 4.697547 0.862756 0.022390
H 4.682488 1.927456 -0.230513
H 5.177730 0.766188 1.000633
H 5.318249 0.348523 -0.714598

4Me_TMA_DMSO_tsopt_GEN
Electronic energy = -998.127504
Thermal correction to Gibbs free energy (25°C) = 0.060620
Thermal correction to Gibbs free energy (80°C) = 0.078172
qh-G(25°C) = -997.843791
qh-G(80°C) = -997.854783

Geometry:

C	3.599177	-1.133777	0.069037
C	3.377806	-0.236236	1.111711
C	2.363525	0.722755	1.052063
C	1.536792	0.804831	-0.070491
C	1.747238	-0.098488	-1.124492
C	2.760879	-1.044735	-1.050029
H	4.008611	-0.274201	1.995593
H	2.243884	1.400255	1.887999
H	1.123633	-0.075555	-2.011962
H	2.901428	-1.731747	-1.880466
N	0.443056	1.736377	-0.147153
C	0.387603	2.497267	-1.408162
H	-0.523113	3.099421	-1.412658
H	1.257760	3.157252	-1.496375
H	0.360470	1.825571	-2.264650
C	0.311212	2.639106	1.001627
H	-0.571125	3.262781	0.849435
H	0.176558	2.063289	1.919248
H	1.187404	3.289587	1.104042
C	-1.295630	0.581484	-0.080181
H	-0.997847	0.142013	-1.024481
H	-0.917915	0.167622	0.847908
H	-1.867334	1.502930	-0.076195
S	-3.133336	-0.619233	-0.044851
O	-4.047947	-0.373873	-1.204119
C	-4.010675	-0.352641	1.492780
H	-4.306148	0.696559	1.515487
H	-4.887876	-1.001737	1.496189
H	-3.343658	-0.584210	2.324925
C	-2.719759	-2.358479	0.048997
H	-3.651868	-2.922694	0.111552
H	-2.176343	-2.608727	-0.862519
H	-2.095700	-2.526009	0.928545
C	4.698828	-2.162356	0.126977
H	5.448825	-1.974669	-0.647589
H	5.201213	-2.147573	1.096487
H	4.302714	-3.168489	-0.038896

4Me_TMA_I_TS
Electronic energy = -456.571013

Thermal correction to Gibbs free energy (25°C) = 0.053304
Thermal correction to Gibbs free energy (80°C) = 0.067871
qh-G(25°C) = -456.367509
qh-G(80°C) = -456.377149

Geometry:

C	3.875782	-1.234781	-0.051755
C	3.025049	-1.123062	1.056138
C	2.041421	-0.145333	1.121299
C	1.873533	0.770661	0.069788
C	2.713422	0.665134	-1.041877
C	3.696152	-0.326919	-1.093266
H	3.130844	-1.819085	1.884416
H	1.402000	-0.108330	1.996728
H	2.626675	1.349386	-1.876416
H	4.336307	-0.382480	-1.969644
N	0.818362	1.739686	0.139152
C	0.729408	2.650417	-1.004714
H	-0.134011	3.301345	-0.858962
H	1.627885	3.272943	-1.096850
H	0.584100	2.083405	-1.926050
C	0.769454	2.490765	1.403729
H	-0.116887	3.128170	1.396200
H	0.697089	1.814065	2.253558
H	1.662924	3.116282	1.517004
C	-1.005040	0.647506	0.045906
I	-3.212070	-0.656007	-0.085778
H	-1.531298	1.590089	0.045260
H	-0.655196	0.220970	-0.882114
H	-0.745170	0.176278	0.981167
C	4.942214	-2.298544	-0.100094
H	5.685719	-2.142122	0.687705
H	4.511296	-3.292775	0.050611
H	5.460553	-2.292657	-1.061391

4Me_TMA_SN2_tsopt_GEN
Electronic energy = -751.944292
Thermal correction to Gibbs free energy (25°C) = 0.060287
Thermal correction to Gibbs free energy (80°C) = 0.077892
qh-G(25°C) = -751.648290
qh-G(80°C) = -751.659178

Geometry:

C	-4.240387	-1.084950	-0.112625
C	-4.235238	0.224246	-0.589653
C	-3.138506	1.069101	-0.401444
C	-2.009984	0.611864	0.279026
C	-2.000115	-0.705549	0.759618

C	-3.099002	-1.532267	0.565375
H	-3.192658	2.078540	-0.788499
H	-1.137963	-1.101476	1.285034
H	-3.066563	-2.549361	0.947051
N	-0.827945	1.432578	0.445090
C	0.561548	0.651607	-0.615753
H	1.248757	1.453143	-0.373610
H	0.567716	-0.257048	-0.028511
H	-0.034233	0.700883	-1.517465
C	-0.331148	1.460993	1.836770
H	0.592665	2.042027	1.860354
H	-1.072603	1.923408	2.495194
H	-0.113904	0.452499	2.184846
C	-0.965195	2.807626	-0.060752
H	-1.177947	2.790258	-1.130695
H	-1.757988	3.346239	0.467574
H	-0.017512	3.323531	0.098864
C	3.007818	-0.371522	-0.923355
C	3.117124	-1.584858	-0.201449
C	4.037814	0.582858	-0.742373
C	4.204938	-1.833231	0.633174
H	2.333319	-2.328522	-0.325265
C	5.122494	0.325325	0.093405
H	3.966946	1.522350	-1.285100
C	5.218646	-0.883583	0.788405
H	4.263370	-2.778218	1.167386
H	5.901381	1.075582	0.204152
H	6.065712	-1.081475	1.437352
O	1.970611	-0.124445	-1.701530
H	-5.103247	0.605723	-1.120128
C	-5.424804	-1.995225	-0.309841
H	-6.202688	-1.506401	-0.900161
H	-5.129616	-2.913951	-0.825218
H	-5.858234	-2.284839	0.652289

4Me_TMA_SnAr_tsopt_GEN
Electronic energy = -751.942798
Thermal correction to Gibbs free energy (25°C) = 0.057337
Thermal correction to Gibbs free energy (80°C) = 0.074484
qh-G(25°C) = -751.644018
qh-G(80°C) = -751.654485

Geometry:

C	1.128483	2.608782	0.132228
C	0.740260	1.801958	1.202182
C	0.687806	0.412306	1.126116
C	0.974112	-0.275075	-0.090580
C	1.436268	0.553645	-1.165853

C	1.475260	1.932689	-1.048773
H	0.464443	2.267820	2.147181
H	0.337620	-0.144709	1.988277
H	1.687800	0.101212	-2.118945
H	1.791378	2.508233	-1.917827
N	1.832334	-1.609059	0.104949
C	3.217986	-1.185202	0.471740
H	3.808813	-2.079333	0.674483
H	3.645078	-0.624824	-0.358975
H	3.164871	-0.550493	1.355399
C	1.914854	-2.443143	-1.134751
H	2.614243	-3.257798	-0.942050
H	0.926319	-2.825587	-1.366273
H	2.292965	-1.834754	-1.953664
C	1.295448	-2.460211	1.210450
H	1.411741	-1.934284	2.155997
H	0.248477	-2.669332	1.005130
H	1.877916	-3.381855	1.239832
O	-0.398379	-1.207593	-0.635199
C	-1.607589	-0.703261	-0.371267
C	-1.927670	0.659406	-0.538449
C	-2.620322	-1.574838	0.073945
C	-3.218678	1.115191	-0.276664
H	-1.159501	1.351058	-0.870932
C	-3.911151	-1.110235	0.318037
H	-2.372888	-2.624175	0.210877
C	-4.221656	0.240530	0.147813
H	-3.444531	2.169565	-0.412873
H	-4.676517	-1.807390	0.648091
H	-5.225319	0.604995	0.341960
C	1.193200	4.113427	0.232831
H	0.526648	4.600346	-0.487924
H	0.897559	4.447190	1.231659
H	2.203911	4.492450	0.043473

4Me_TMA_bromide_tsopt
Electronic energy = -3016.712602
Thermal correction to Gibbs free energy (25°C) = 0.052276
Thermal correction to Gibbs free energy (80°C) = 0.066647
qh-G(25°C) = -3016.507662
qh-G(80°C) = -3016.517132

Geometry:

C	3.431785	-1.046142	-0.051428
C	2.561789	-1.033829	1.047022
C	1.491237	-0.152321	1.115743
C	1.251877	0.762765	0.077847
C	2.110646	0.756565	-1.023989

C 3.181982 -0.138921 -1.078892
H 2.722326 -1.731666 1.864852
H 0.842018 -0.190194 1.983858
H 1.970295 1.444672 -1.848025
H 3.835162 -0.118438 -1.947111
N 0.105827 1.625942 0.148996
C -0.050605 2.548393 -0.979231
H -0.971358 3.115458 -0.833525
H 0.788636 3.251175 -1.045552
H -0.132000 1.989474 -1.913246
C -0.024949 2.348726 1.425388
H -0.968149 2.898474 1.418825
H -0.039692 1.654348 2.263867
H 0.803827 3.054085 1.556649
C -1.574646 0.377931 0.016505
H -2.196334 1.260280 0.007930
H -1.170932 -0.010490 -0.906227
H -1.289193 -0.071798 0.954508
C 4.590265 -2.008484 -0.106140
H 5.287324 -1.828218 0.717973
H 4.244250 -3.042959 -0.020886
H 5.140411 -1.909014 -1.044361
Br -3.469247 -1.018139 -0.140270

4Me_TMA_chloride_tsopt
Electronic energy = -905.391455
Thermal correction to Gibbs free energy (25°C) = 0.050807
Thermal correction to Gibbs free energy (80°C) = 0.064922
qh-G(25°C) = -905.184603
qh-G(80°C) = -905.193847

Geometry:
C 2.981319 -0.677333 -0.055535
C 2.108765 -0.882661 1.021393
C 0.884184 -0.232423 1.098118
C 0.485954 0.660464 0.090380
C 1.346150 0.869808 -0.989949
C 2.574055 0.205822 -1.053177
H 2.390896 -1.568784 1.815910
H 0.241831 -0.432868 1.948906
H 1.086566 1.551945 -1.789568
H 3.224751 0.391498 -1.903459
N -0.809372 1.280015 0.167415
C -1.118951 2.209268 -0.923896
H -2.135387 2.579247 -0.780622
H -0.430739 3.062698 -0.933529
H -1.070738 1.691565 -1.883519
C -1.095949 1.907645 1.469133

H	-2.125256	2.271277	1.460190
H	-0.995051	1.185177	2.277410
H	-0.416889	2.748732	1.649880
C	-2.205303	-0.241332	-0.059122
H	-2.984000	0.505405	-0.021416
H	-1.735372	-0.489104	-0.998922
H	-1.841381	-0.688726	0.852658
C	4.306634	-1.391761	-0.120203
H	4.166223	-2.476949	-0.122194
H	4.857501	-1.117847	-1.022570
H	4.926950	-1.144342	0.746573
Cl	-3.711497	-1.858472	-0.313558

4Me_TMA_gen
Electronic energy = -445.093213
Thermal correction to Gibbs free energy (25°C) = 0.045225
Thermal correction to Gibbs free energy (80°C) = 0.057876
qh-G(25°C) = -444.880277
qh-G(80°C) = -444.888549

Geometry:

C	-0.354036	0.033881	-0.001030
C	0.343745	1.233721	-0.000725
C	1.740477	1.209432	0.000071
C	2.450513	0.010544	0.000432
C	1.719386	-1.184855	0.000185
C	0.331564	-1.182148	-0.000490
H	-0.155960	2.192955	-0.000777
H	2.243210	-2.136325	0.000814
H	-0.192274	-2.131682	-0.000196
N	-1.849730	-0.000294	-0.000090
C	-2.340073	-0.722792	-1.227716
H	-1.977245	-1.747694	-1.211087
H	-3.429390	-0.712140	-1.214533
H	-1.958140	-0.199305	-2.103106
C	-2.337484	-0.712881	1.234322
H	-3.426836	-0.705070	1.222005
H	-1.971508	-1.736775	1.226448
H	-1.956110	-0.180561	2.104635
C	-2.459850	1.370433	-0.005159
H	-2.149566	1.904052	0.891519
H	-2.148235	1.898037	-0.904970
H	-3.541330	1.245216	-0.005400
H	2.276744	2.153366	0.000640
C	3.955930	-0.014778	-0.000282
H	4.367733	0.995881	0.019797
H	4.332495	-0.522472	-0.893023
H	4.333486	-0.558769	0.870218

4Me_aniline_gen
Electronic energy = -405.363719
Thermal correction to Gibbs free energy (25°C) = 0.044058
Thermal correction to Gibbs free energy (80°C) = 0.055913
qh-G(25°C) = -405.194026
qh-G(80°C) = -405.202040

Geometry:
C -0.665456 0.000465 -0.112667
C 0.069066 1.203391 -0.070748
C 1.461209 1.191352 -0.020478
C 2.192810 0.001679 0.004716
C 1.461781 -1.189586 -0.023126
C 0.070766 -1.202677 -0.073443
H -0.439187 2.159808 -0.082781
H 1.990169 -2.140316 -0.000415
H -0.437004 -2.159344 -0.087795
N -2.054638 -0.000837 -0.209117
C -2.752527 -1.234451 0.114347
H -3.825357 -1.066829 0.019848
H -2.480482 -2.028992 -0.585166
H -2.538990 -1.583746 1.136479
C -2.753885 1.233027 0.109752
H -2.542452 1.585289 1.131360
H -2.480582 2.025776 -0.591351
H -3.826440 1.064619 0.013596
H 1.988540 2.142470 0.004338
C 3.698688 -0.000915 0.095010
H 4.117274 0.934578 -0.285242
H 4.128071 -0.825471 -0.480873
H 4.034194 -0.115693 1.131643

4Me_bromide_ionpair_gen
Electronic energy = -3016.767355
Thermal correction to Gibbs free energy (25°C) = 0.051416
Thermal correction to Gibbs free energy (80°C) = 0.065541
qh-G(25°C) = -3016.557490
qh-G(80°C) = -3016.566756

Geometry:
Br -3.115721 -1.062432 0.000123
N 0.098518 1.840683 -0.000413
C 1.111397 0.740720 0.000034
C 2.474324 1.005381 0.000938
C 3.373848 -0.063927 0.001773
C 2.935289 -1.386760 0.001425

C	1.553389	-1.619848	0.000910
C	-0.768811	1.725475	1.226969
H	-1.461492	2.567387	1.227891
H	-0.121771	1.757799	2.102805
H	-1.325393	0.789548	1.178872
C	-0.768510	1.724665	-1.227930
H	-1.461313	2.566471	-1.229478
H	-1.324991	0.788702	-1.179447
H	-0.121278	1.756590	-2.103639
C	0.723452	3.201609	-0.000807
H	-0.084860	3.930874	-0.001486
H	1.327807	3.319750	-0.898991
H	1.327044	3.320650	0.897768
C	0.641031	-0.573364	0.000153
H	-0.423936	-0.794733	0.000089
H	2.869095	2.012599	0.001448
H	4.438199	0.151053	0.002926
H	1.181087	-2.640653	0.001543
C	3.905861	-2.538107	-0.001838
H	3.728229	-3.197103	0.852827
H	4.937711	-2.184717	0.044292
H	3.788838	-3.138950	-0.908598

4Me_chloride_ionpair_gen
Electronic energy = -905.445338
Thermal correction to Gibbs free energy (25°C) = 0.050943
Thermal correction to Gibbs free energy (80°C) = 0.064991
qh-G(25°C) = -905.235287
qh-G(80°C) = -905.244459

Geometry:

Cl	-2.955814	-2.324711	0.000051
N	-1.008930	1.376722	-0.000265
C	0.315148	0.682326	-0.000131
C	1.508334	1.392154	0.000336
C	2.716562	0.690379	0.001115
C	2.750985	-0.702870	0.001245
C	1.529394	-1.389658	0.001005
C	-1.787240	0.975607	1.226824
H	-2.721803	1.537089	1.229127
H	-1.188430	1.221733	2.103020
H	-1.998125	-0.092708	1.175216
C	-1.787140	0.975275	-1.227291
H	-2.721933	1.536377	-1.229540
H	-1.997615	-0.093116	-1.175649
H	-1.188481	1.221623	-2.103528
C	-0.875648	2.868103	-0.000456
H	-1.880480	3.286739	-0.000822

H	-0.344657	3.181157	-0.898179
H	-0.345208	3.181433	0.897497
C	0.316540	-0.713456	0.000171
H	-0.612503	-1.279809	0.000274
H	1.538689	2.473447	0.000583
H	3.645368	1.252771	0.001909
H	1.524741	-2.476220	0.001827
C	4.053370	-1.458875	-0.001380
H	4.109680	-2.136148	0.855611
H	4.905686	-0.778055	0.041119
H	4.144589	-2.067187	-0.906113

4OMe_SnAr_Product_gen_2
Electronic energy = -652.792401
Thermal correction to Gibbs free energy (25°C) = 0.048799
Thermal correction to Gibbs free energy (80°C) = 0.062523
qh-G(25°C) = -652.607977
qh-G(80°C) = -652.616860

Geometry:

C	3.045773	-1.735417	0.031048
C	1.911260	-0.921471	-0.014595
C	2.073836	0.461963	-0.092340
C	3.350778	1.028439	-0.126395
C	4.469447	0.203256	-0.081148
C	4.324592	-1.184502	-0.001375
H	2.918408	-2.811805	0.091458
H	0.920231	-1.360472	0.009122
H	3.445069	2.107755	-0.187622
H	5.459609	0.647350	-0.107160
H	5.198893	-1.825517	0.034770
O	1.024866	1.348594	-0.154573
C	-0.262439	0.853310	-0.010270
C	-1.034970	0.621234	-1.137286
C	-2.351136	0.168942	-1.003325
H	-0.609969	0.793622	-2.120990
C	-2.876998	-0.049701	0.272296
H	-2.943071	-0.004751	-1.893415
C	-0.783264	0.640403	1.266494
H	-0.162940	0.828801	2.137288
C	-2.087457	0.188958	1.406315
H	-2.517237	0.017305	2.387660
O	-4.141294	-0.489046	0.513368
C	-4.974890	-0.745475	-0.606311
H	-4.547518	-1.527727	-1.242554
H	-5.926881	-1.084639	-0.201357
H	-5.130351	0.164063	-1.196206

4OMe_TMA_DMSO_tsopt_GEN
Electronic energy = -1073.312062
Thermal correction to Gibbs free energy (25°C) = 0.062140
Thermal correction to Gibbs free energy (80°C) = 0.080288
qh-G(25°C) = -1073.023781
qh-G(80°C) = -1073.035091

Geometry:

C	3.344184	-0.694673	-0.232395
C	3.130992	0.102259	0.890136
C	2.027427	0.961085	0.937892
C	1.126801	1.037361	-0.123030
C	1.349856	0.222586	-1.246885
C	2.441295	-0.626659	-1.302297
H	3.807291	0.078285	1.735751
H	1.900758	1.569771	1.824235
H	0.670817	0.240882	-2.092742
H	2.610349	-1.252765	-2.172061
N	-0.050311	1.864907	-0.074662
C	-0.232385	2.719847	-1.260713
H	-1.204533	3.212110	-1.189998
H	0.555433	3.479889	-1.311383
H	-0.212756	2.124848	-2.172515
C	-0.200118	2.656080	1.150897
H	-1.137018	3.212312	1.089333
H	-0.240470	1.997639	2.020967
H	0.622675	3.370396	1.270956
C	-1.682092	0.554729	-0.029938
H	-1.473088	0.311144	-1.064822
H	-1.161449	0.028556	0.762151
H	-2.311834	1.407671	0.198104
S	-3.416162	-0.784167	-0.000844
O	-4.510153	-0.374193	-0.936530
C	-4.058198	-0.916951	1.665154
H	-4.380274	0.080774	1.965091
H	-4.903694	-1.606756	1.642701
H	-3.266817	-1.281949	2.321990
C	-2.910082	-2.464938	-0.352273
H	-3.789225	-3.105364	-0.264200
H	-2.522608	-2.476949	-1.371590
H	-2.135186	-2.756289	0.358647
O	4.381714	-1.558110	-0.380709
C	5.315736	-1.652957	0.684493
H	4.827189	-1.991029	1.604430
H	6.054878	-2.388564	0.372042
H	5.807358	-0.690478	0.860712

4OMe_TMA_I_TS
Electronic energy = -531.755402
Thermal correction to Gibbs free energy (25°C) = 0.054981
Thermal correction to Gibbs free energy (80°C) = 0.070181
qh-G(25°C) = -531.547774
qh-G(80°C) = -531.557765

Geometry:

C	3.655386	-0.792554	-0.228000
C	3.501859	0.059726	0.862415
C	2.422875	0.949819	0.906281
C	1.486693	1.002166	-0.125081
C	1.648386	0.127822	-1.214362
C	2.715463	-0.751973	-1.266737
H	4.206535	0.055743	1.685076
H	2.342740	1.603308	1.765726
H	0.936111	0.123945	-2.032949
H	2.836264	-1.425237	-2.109220
N	0.345622	1.872917	-0.083991
C	0.178668	2.697079	-1.291299
H	-0.776501	3.222762	-1.225876
H	0.989672	3.430920	-1.374794
H	0.168715	2.074753	-2.185065
C	0.240308	2.701203	1.118760
H	-0.687095	3.273888	1.062413
H	0.206459	2.068425	2.007873
H	1.079483	3.403498	1.201891
C	-1.390892	0.634870	0.005824
H	-1.969155	1.530108	0.176799
H	-1.179844	0.316027	-1.002780
H	-0.933824	0.118057	0.836867
O	4.667883	-1.687946	-0.373221
C	5.649427	-1.739705	0.650594
H	6.368220	-2.496814	0.340897
H	6.155536	-0.774161	0.756838
H	5.202200	-2.027964	1.608091
I	-3.497301	-0.819525	0.104746

4OMe_TMA_SN2_tsopt_GEN
Electronic energy = -827.128826
Thermal correction to Gibbs free energy (25°C) = 0.061668
Thermal correction to Gibbs free energy (80°C) = 0.079855
qh-G(25°C) = -826.827991
qh-G(80°C) = -826.839190

Geometry:

C	-4.039265	-0.504629	-0.150522
C	-3.908957	0.812675	-0.597688

C -2.729417 1.517046 -0.392241
C -1.649558 0.918199 0.267603
C -1.784840 -0.400736 0.706273
C -2.966039 -1.112579 0.506141
H -2.675102 2.537287 -0.749919
H -0.969619 -0.905117 1.213235
H -3.027924 -2.132684 0.864309
N -0.384040 1.600981 0.452158
C 0.930626 0.684620 -0.601347
H 1.686454 1.424189 -0.365998
H 0.853860 -0.213458 -0.002891
H 0.339197 0.778433 -1.502367
C 0.094162 1.563915 1.849788
H 1.082247 2.026759 1.888454
H -0.593758 2.111345 2.501220
H 0.180203 0.534875 2.195453
C -0.366032 2.987503 -0.039442
H -0.575897 3.005321 -1.110089
H -1.094938 3.606414 0.493128
H 0.632536 3.393433 0.128059
C 3.294963 -0.512760 -0.910419
C 3.367438 -1.727499 -0.186771
C 4.362847 0.402206 -0.745797
C 4.457162 -2.015933 0.632367
H 2.553386 -2.440021 -0.296731
C 5.448538 0.105245 0.075465
H 4.320772 1.342433 -1.290394
C 5.508499 -1.105463 0.771183
H 4.487051 -2.961424 1.168022
H 6.256807 0.825542 0.173648
H 6.356563 -1.334503 1.408468
O 2.258028 -0.227581 -1.676273
H -4.746110 1.279742 -1.105626
O -5.230793 -1.107885 -0.394452
C -5.393097 -2.449804 0.041316
H -5.289337 -2.523844 1.128910
H -6.401277 -2.739991 -0.249302
H -4.666889 -3.109908 -0.444341

4OMe_TMA_SnAr_tsopt_GEN
Electronic energy = -827.123943
Thermal correction to Gibbs free energy (25°C) = 0.059233
Thermal correction to Gibbs free energy (80°C) = 0.077020
qh-G(25°C) = -826.821180
qh-G(80°C) = -826.832000

Geometry:
C -0.228983 2.563513 0.182027

C	-0.148695	1.678068	1.251350
C	0.538171	0.473488	1.146479
C	1.132290	0.057919	-0.086209
C	1.104373	1.028963	-1.142139
C	0.412156	2.222245	-1.010602
H	-0.632523	1.933358	2.191372
H	0.540612	-0.199030	1.996817
H	0.368298	2.909156	-1.852531
N	2.552620	-0.647677	0.092952
C	3.526512	0.418555	0.480718
H	4.491530	-0.051530	0.674306
H	3.607187	1.134420	-0.336268
H	3.156840	0.919568	1.374499
C	3.048176	-1.294555	-1.162721
H	4.066285	-1.639921	-0.978811
H	2.394235	-2.123980	-1.411023
H	3.061004	-0.560792	-1.965766
C	2.531560	-1.679407	1.175924
H	2.377950	-1.189789	2.135477
H	1.731518	-2.383968	0.963071
H	3.499627	-2.181531	1.181696
O	0.427998	-1.429104	-0.653559
C	-0.869602	-1.613899	-0.384686
C	-1.840769	-0.605998	-0.549328
C	-1.293052	-2.878905	0.065057
C	-3.181973	-0.870318	-0.277726
H	-1.536134	0.379958	-0.887392
C	-2.639011	-3.136744	0.318004
C	-3.595708	-2.133110	0.152857
H	-3.914052	-0.077835	-0.410481
H	-2.939966	-4.125811	0.652398
H	-4.643401	-2.330885	0.355431
H	-0.544416	-3.655182	0.199164
H	1.561939	0.795181	-2.096722
O	-0.908743	3.768688	0.302070
C	-2.285812	3.660095	-0.035817
H	-2.729894	4.649329	0.088786
H	-2.404935	3.332295	-1.075995
H	-2.791878	2.944589	0.624076

4OMe_TMA_bromide_tsopt
Electronic energy = -3091.897170
Thermal correction to Gibbs free energy (25°C) = 0.053819
Thermal correction to Gibbs free energy (80°C) = 0.068797
qh-G(25°C) = -3091.687565
qh-G(80°C) = -3091.697367

Geometry:

C -3.152242 -0.638038 0.243262
C -2.904702 0.156851 -0.873239
C -1.752811 0.949888 -0.925234
C -0.835397 0.960773 0.124450
C -1.095081 0.148126 1.242349
C -2.234768 -0.634913 1.302536
H -3.590406 0.181493 -1.711289
H -1.601023 1.559852 -1.806772
H -0.404606 0.115646 2.078202
H -2.430211 -1.259995 2.167610
N 0.384590 1.719212 0.071909
C 0.635034 2.535240 1.271339
H 1.620313 2.996517 1.178579
H -0.123808 3.320187 1.371373
H 0.630229 1.916574 2.167592
C 0.560980 2.524314 -1.139354
H 1.534451 3.014501 -1.087281
H 0.542190 1.882961 -2.022611
H -0.215581 3.293621 -1.230102
C 1.953020 0.322694 -0.023141
H 2.641406 1.147587 -0.127171
H 1.675134 -0.025740 0.959249
H 1.471197 -0.095938 -0.893449
O -4.238781 -1.440378 0.395425
C -5.188589 -1.465228 -0.659022
H -5.968435 -2.157380 -0.345983
H -5.621815 -0.472243 -0.819486
H -4.732653 -1.822221 -1.588599
Br 3.710830 -1.236147 -0.152580

4OMe_TMA_chloride_tsopt
Electronic energy = -980.576018
Thermal correction to Gibbs free energy (25°C) = 0.052504
Thermal correction to Gibbs free energy (80°C) = 0.067250
qh-G(25°C) = -980.364794
qh-G(80°C) = -980.374387

Geometry:
C -2.624778 -0.440532 0.226478
C -2.273113 0.397626 -0.828734
C -1.002302 0.982811 -0.862618
C -0.069967 0.742097 0.144976
C -0.435989 -0.111746 1.200309
C -1.692307 -0.690869 1.242426
H -2.966780 0.614630 -1.631718
H -0.769799 1.634542 -1.695381
H 0.261168 -0.336811 2.000369
H -1.970069 -1.349136 2.058955

N 1.261980 1.286529 0.109920
C 1.654020 1.971752 1.353442
H 2.703961 2.261407 1.276018
H 1.042087 2.867678 1.510132
H 1.542642 1.308450 2.209990
C 1.550783 2.135514 -1.049381
H 2.595197 2.447347 -0.994787
H 1.403571 1.571750 -1.972632
H 0.917391 3.030616 -1.062306
C 2.565818 -0.321981 -0.112248
H 3.375679 0.385462 -0.207129
H 2.270272 -0.673333 0.863925
H 1.995058 -0.614736 -0.980534
O -3.830025 -1.054816 0.356518
C -4.799511 -0.817956 -0.652927
H -5.680000 -1.389928 -0.365177
H -5.054689 0.245588 -0.708873
H -4.441099 -1.162047 -1.628893
Cl 3.961519 -2.028762 -0.368463

4OMe_TMA_gen
Electronic energy = -520.278266
Thermal correction to Gibbs free energy (25°C) = 0.046247
Thermal correction to Gibbs free energy (80°C) = 0.059394
qh-G(25°C) = -520.059730
qh-G(80°C) = -520.068250

Geometry:
C 0.747440 0.074987 -0.000790
C 0.154919 1.337066 -0.000748
C -1.228648 1.444775 -0.000376
C -2.033489 0.300751 -0.000036
C -1.434016 -0.963379 -0.000148
C -0.046252 -1.067566 -0.000428
H 0.738048 2.247914 -0.001082
H -2.025161 -1.870118 0.000040
H 0.387887 -2.061359 -0.000321
N 2.232701 -0.092237 -0.000008
C 2.658957 -0.845494 1.232414
H 2.204389 -1.833337 1.223628
H 3.744796 -0.934167 1.217739
H 2.328085 -0.283065 2.104265
C 2.660091 -0.854511 -1.226512
H 3.746164 -0.940145 -1.211899
H 2.208752 -1.843755 -1.209427
H 2.327156 -0.300160 -2.102733
C 2.959322 1.220038 -0.004478
H 2.694483 1.772741 -0.904275

H 2.695671 1.778092 0.892327
H 4.025989 1.002305 -0.004219
H -1.701857 2.420323 -0.000328
O -3.367507 0.510154 0.000437
C -4.222137 -0.627391 -0.000023
H -5.237771 -0.236710 0.000028
H -4.061212 -1.234585 -0.896198
H -4.061392 -1.235202 0.895768

4OMe_aniline_gen
Electronic energy = -480.544176
Thermal correction to Gibbs free energy (25°C) = 0.045492
Thermal correction to Gibbs free energy (80°C) = 0.057899
qh-G(25°C) = -480.369996
qh-G(80°C) = -480.378278

Geometry:
C 1.061926 -0.000019 -0.153811
C 0.329220 -1.206368 -0.185501
C -1.060216 -1.201150 -0.272786
C -1.761957 0.000086 -0.323091
C -1.060138 1.201263 -0.272570
C 0.329303 1.206376 -0.185285
H 0.838630 -2.161043 -0.147248
H -1.607507 2.139162 -0.303225
H 0.838778 2.161012 -0.146837
N 2.452276 -0.000051 -0.112566
C 3.119472 1.235391 0.262898
H 4.195722 1.064304 0.275908
H 2.919943 2.022485 -0.469074
H 2.808394 1.597309 1.255040
C 3.119462 -1.235448 0.263099
H 2.808078 -1.597395 1.255132
H 2.920271 -2.022568 -0.468937
H 4.195689 -1.064252 0.276486
H -1.607644 -2.139009 -0.303623
O -3.138562 0.000154 -0.440650
C -3.809297 -0.000160 0.816276
H -4.879391 -0.000077 0.606633
H -3.547549 -0.894381 1.393540
H -3.547499 0.893747 1.394006

4OMe_bromide_ionpair_gen_3
Electronic energy = -3091.952568
Thermal correction to Gibbs free energy (25°C) = 0.052580
Thermal correction to Gibbs free energy (80°C) = 0.067243
qh-G(25°C) = -3091.737503

qh-G(80°C) = -3091.747040

Geometry:

Br	3.019376	-1.586195	-0.000040
N	0.586834	1.999452	0.000021
C	-0.650175	1.161121	-0.000001
C	-1.923079	1.729974	-0.000113
C	-3.039682	0.904536	-0.000124
C	-2.897473	-0.486937	-0.000024
C	-1.616697	-1.049248	0.000144
C	1.406340	1.691319	-1.226860
H	2.275968	2.348827	-1.223233
H	0.786594	1.876479	-2.103486
H	1.729718	0.651336	-1.182314
C	1.406311	1.691273	1.226904
H	2.275948	2.348770	1.223317
H	1.729681	0.651286	1.182306
H	0.786543	1.876417	2.103516
C	0.286719	3.466527	0.000077
H	1.238480	3.995129	0.000147
H	-0.275316	3.717870	0.898231
H	-0.275207	3.717957	-0.898114
C	-0.497750	-0.221328	0.000139
H	0.487473	-0.682785	0.000249
H	-2.078611	2.800609	-0.000138
H	-4.036662	1.331012	-0.000197
H	-1.470492	-2.122102	0.000266
O	-4.043996	-1.203908	-0.000127
C	-3.941552	-2.622558	0.000079
H	-3.420974	-2.975646	0.895829
H	-4.964007	-2.995373	0.000087
H	-3.420933	-2.975929	-0.895532

4OMe_chloride_ionpair_gen_2
Electronic energy = -980.630556
Thermal correction to Gibbs free energy (25°C) = 0.051831
Thermal correction to Gibbs free energy (80°C) = 0.066373
qh-G(25°C) = -980.414817
qh-G(80°C) = -980.424231

Geometry:

Cl	-2.617091	2.851071	0.000350
N	-1.638890	-1.211203	-0.000147
C	-0.185549	-0.864564	-0.000125
C	0.802301	-1.848270	0.000421
C	2.139242	-1.473857	0.000552
C	2.500597	-0.122745	0.000135
C	1.503217	0.858177	-0.000446

C -2.295348 -0.632970 -1.227321
H -3.341086 -0.941298 -1.225815
H -1.779539 -1.026023 -2.102719
H -2.230336 0.454115 -1.180527
C -2.295362 -0.632920 1.227021
H -3.341237 -0.940794 1.225233
H -2.229808 0.454152 1.180465
H -1.779889 -1.026392 2.102430
C -1.876503 -2.689472 -0.000147
H -2.953580 -2.848148 -0.000276
H -1.439530 -3.123013 0.898106
H -1.439248 -3.123035 -0.898256
C 0.162649 0.482237 -0.000562
H -0.596939 1.261709 -0.000916
H 0.567191 -2.904204 0.000747
H 2.919510 -2.226858 0.000968
H 1.748061 1.912926 -0.000808
O 3.827353 0.139039 0.000291
C 4.237870 1.500662 -0.000294
H 3.877673 2.016942 0.895113
H 5.326075 1.484035 -0.000117
H 3.877940 2.016111 -0.896285

Anisole_DMSO_TS
Electronic energy = -899.656547
Thermal correction to Gibbs free energy (25°C) = 0.051304
Thermal correction to Gibbs free energy (80°C) = 0.065465
qh-G(25°C) = -899.479958
qh-G(80°C) = -899.489240

Geometry:
S -2.777470 -0.019689 0.039047
O -3.894883 -0.811112 -0.563049
C -2.975746 0.065036 1.815125
H -2.168419 0.670915 2.229669
H -3.950731 0.508571 2.024161
H -2.924261 -0.956702 2.192376
C -2.916669 1.699198 -0.437766
H -3.904200 2.052695 -0.136395
H -2.126091 2.266125 0.056927
H -2.803027 1.743286 -1.521332
O 1.029436 -1.450699 -0.869515
C 2.003866 -0.677859 -0.448961
C 3.042727 -1.184281 0.374691
C 2.069013 0.701055 -0.781206
C 4.077524 -0.368612 0.821031
H 3.011171 -2.236768 0.645495
C 3.106141 1.510136 -0.320846

H 1.296942 1.116831 -1.425138
C 4.121901 0.988390 0.483207
H 4.858972 -0.794992 1.445703
H 3.123827 2.561016 -0.600093
H 4.929614 1.621408 0.836140
C -0.819190 -0.717621 -0.431352
H -0.307658 0.050246 0.139418
H -0.949868 -0.590437 -1.497912
H -0.980570 -1.695325 0.003211

Br1_ionpair_displ_conf2_DMSO1
Electronic energy = -3541.311034
Thermal correction to Gibbs free energy (25°C) = 0.067470
Thermal correction to Gibbs free energy (80°C) = 0.086345
qh-G(25°C) = -3541.066434
qh-G(80°C) = -3541.078423

Geometry:
C -3.825736 2.108900 -0.110088
C -3.137923 0.899167 -0.063211
C -1.753316 0.845987 0.002617
C -1.044414 2.047098 0.023217
C -1.698899 3.271686 -0.022258
C -3.093753 3.289565 -0.089341
H -1.164557 4.211539 -0.007509
H -3.609701 4.242450 -0.125431
N 0.440935 1.957899 0.093815
C 0.962622 1.216950 -1.113210
H 2.051678 1.234471 -1.047441
H 0.607443 1.735042 -2.003856
H 0.594941 0.190559 -1.084745
C 0.847048 1.219138 1.345644
H 1.937045 1.235941 1.382952
H 0.484384 0.192848 1.285079
H 0.410818 1.739490 2.198104
C 1.111141 3.299408 0.125020
H 2.182838 3.108218 0.178367
H 0.778299 3.844839 1.007277
H 0.867488 3.842431 -0.787201
H -1.252975 -0.119314 0.035230
I 0.217130 -2.643261 0.078079
O 3.873207 1.583639 0.209656
S 4.795806 0.374457 0.422051
C 4.024313 -0.979337 -0.487888
H 4.676538 -1.854103 -0.445601
H 3.070577 -1.205676 -0.004026
H 3.860076 -0.664803 -1.521516
C 6.199479 0.644750 -0.677450

H 6.831621 -0.245334 -0.674381
H 5.822113 0.856893 -1.680030
H 6.755203 1.498313 -0.288640
H -4.908185 2.125599 -0.161363
Br -4.110058 -0.726348 -0.087958

Br2_TMA_DMSO_2
Electronic energy = -3529.778111
Thermal correction to Gibbs free energy (25°C) = 0.059644
Thermal correction to Gibbs free energy (80°C) = 0.076679
qh-G(25°C) = -3529.529312
qh-G(80°C) = -3529.540138

Geometry:
C -1.974573 -0.792821 0.023097
C -3.305823 -0.403561 0.107685
C -3.608219 0.958485 0.161131
C -2.603201 1.917045 0.130290
C -1.280925 1.490651 0.043456
C -0.943170 0.145768 -0.012142
H -4.117422 -1.117193 0.133273
H 0.101065 -0.150498 -0.082594
N -1.587007 -2.234131 -0.039308
C -0.854648 -2.500587 -1.331384
H 0.079150 -1.938888 -1.328012
H -0.645982 -3.569326 -1.377879
H -1.501119 -2.198788 -2.154634
C -0.679947 -2.564986 1.120253
H -0.455724 -3.630651 1.070520
H 0.239398 -1.988427 1.025095
H -1.209548 -2.325668 2.041741
C -2.770451 -3.151536 0.023990
H -3.296131 -2.995385 0.964899
H -3.421160 -2.960772 -0.827923
H -2.392636 -4.171213 -0.022880
O 1.883794 -1.197583 -0.255028
S 3.374183 -0.909083 -0.477799
C 3.571010 0.857492 -0.171570
H 3.056850 1.383502 -0.976580
H 3.116564 1.098794 0.792049
H 4.634481 1.104971 -0.181233
C 4.198226 -1.510924 1.009461
H 4.104535 -2.597133 1.011957
H 5.251765 -1.227348 0.973770
H 3.703187 -1.080858 1.882591
H -2.840113 2.973777 0.172561
Br 0.108572 2.777651 -0.000285
H -4.645093 1.268068 0.227815

Br3_dimer_Tshape_CONF1
Electronic energy = -5976.425781
Thermal correction to Gibbs free energy (25°C) = 0.083232
Thermal correction to Gibbs free energy (80°C) = 0.107652
qh-G(25°C) = -5976.061046
qh-G(80°C) = -5976.075815

Geometry:

C	-0.071863	-3.672088	-1.605721
C	0.682779	-3.546936	-2.765688
C	1.858106	-2.792553	-2.784328
C	2.268597	-2.158562	-1.618219
C	1.527879	-2.261277	-0.440587
C	0.363341	-3.016785	-0.455899
H	-0.980491	-4.263467	-1.593049
H	2.417492	-2.725002	-3.706951
H	1.831375	-1.763122	0.478680
N	3.512430	-1.335889	-1.571272
C	3.151195	0.101622	-1.272584
H	4.069975	0.687639	-1.257638
H	2.486771	0.458742	-2.059009
H	2.669255	0.153326	-0.295123
C	4.272415	-1.367091	-2.863031
H	5.162908	-0.755366	-2.729890
H	4.556133	-2.394626	-3.087150
H	3.654782	-0.947466	-3.655176
C	4.423980	-1.850805	-0.485390
H	5.345339	-1.270506	-0.523578
H	3.942709	-1.711986	0.482463
H	4.623497	-2.904059	-0.678715
I	2.418071	-0.132457	2.735434
C	-4.837541	-1.044662	-0.594028
C	-4.905525	0.290906	-0.222034
C	-3.779828	1.112625	-0.211262
C	-2.560205	0.561791	-0.587003
C	-2.462437	-0.775448	-0.966755
C	-3.602371	-1.570755	-0.966555
H	-5.727428	-1.662993	-0.593225
H	-3.886652	2.146013	0.086775
H	-1.514911	-1.211695	-1.263771
H	-3.527113	-2.612701	-1.258008
N	-1.316256	1.382828	-0.581646
C	-0.348564	0.831831	0.439653
H	0.550421	1.451796	0.414601
H	-0.821470	0.868420	1.420950
H	-0.109751	-0.199304	0.180375
C	-1.562097	2.823859	-0.239533

H -0.594760 3.326910 -0.279735
H -2.243524 3.252571 -0.973462
H -1.973122 2.889856 0.766648
C -0.669373 1.350102 -1.942867
H 0.217788 1.985449 -1.896184
H -0.387634 0.326624 -2.184449
H -1.386776 1.727859 -2.670687
I 2.415231 3.624214 -0.600292
Br -6.571023 1.031133 0.288409
Br -0.650814 -3.153803 1.138642
H 0.360366 -4.045819 -3.672594

Br3_dimer_Tshape_CONF3
Electronic energy = -5976.427183
Thermal correction to Gibbs free energy (25°C) = 0.083397
Thermal correction to Gibbs free energy (80°C) = 0.107817
qh-G(25°C) = -5976.062643
qh-G(80°C) = -5976.077408

Geometry:
C 2.322338 -2.453065 1.298218
C 1.761676 -2.787872 2.524758
C 0.376675 -2.821301 2.700494
C -0.445226 -2.511745 1.624012
C 0.089260 -2.164178 0.383475
C 1.470448 -2.135365 0.243045
H 3.397130 -2.439688 1.162155
H -0.018069 -3.093172 3.669518
H -0.541610 -1.923293 -0.470659
N -1.932340 -2.546629 1.739294
C -2.493312 -1.169587 1.466801
H -3.574722 -1.213544 1.594475
H -2.054294 -0.472797 2.180560
H -2.260479 -0.883955 0.439947
C -2.396013 -2.973988 3.099066
H -3.484453 -2.974362 3.084626
H -2.027786 -3.977966 3.305992
H -2.039341 -2.261534 3.840934
C -2.494230 -3.516764 0.730448
H -3.573661 -3.556671 0.873858
H -2.270162 -3.157053 -0.273659
H -2.043631 -4.492459 0.908676
I -2.234851 -1.128030 -2.624685
C 4.502629 2.631830 -0.523169
C 3.688472 3.676833 -0.940966
C 2.310512 3.646947 -0.722477
C 1.751663 2.547812 -0.081358
C 2.546015 1.482166 0.341925

C 3.914483 1.544087 0.115397
H 5.573254 2.659231 -0.688369
H 1.712777 4.482633 -1.057915
H 2.128932 0.614165 0.840918
N 0.283204 2.457723 0.158321
C -0.300079 1.348811 -0.684374
H -1.375203 1.314554 -0.494072
H -0.096220 1.565199 -1.732906
H 0.170985 0.408154 -0.399721
C -0.442906 3.723938 -0.195292
H -1.494353 3.562372 0.046294
H -0.036574 4.543561 0.396211
H -0.333817 3.914514 -1.261434
C 0.002641 2.172161 1.611349
H -1.081922 2.169106 1.738188
H 0.410094 1.197723 1.874222
H 0.468152 2.955276 2.208938
I -3.989737 2.127399 0.940870
Br 5.010330 0.121687 0.709964
H 2.406737 -3.035275 3.360237
H 4.126745 4.533298 -1.440594
Br 2.200193 -1.671016 -1.444124

Br3_dimer_Tshape_CONF4
Electronic energy = -5976.422849
Thermal correction to Gibbs free energy (25°C) = 0.084825
Thermal correction to Gibbs free energy (80°C) = 0.109488
qh-G(25°C) = -5976.059648
qh-G(80°C) = -5976.074660

Geometry:
C 2.452848 1.804128 0.629646
C 1.977101 2.736226 -0.283209
C 0.643068 3.140321 -0.294864
C -0.228993 2.570903 0.627518
C 0.212993 1.614951 1.541322
C 1.557087 1.252706 1.544334
H 3.497202 1.511756 0.627654
H 0.327628 3.877406 -1.020194
H -0.465275 1.131753 2.237332
N -1.676072 2.934277 0.619393
C -2.442288 1.826244 -0.066550
H -3.500337 2.089810 -0.063621
H -2.078134 1.742632 -1.089624
H -2.284745 0.896934 0.484858
C -1.945217 4.216694 -0.112540
H -3.004466 4.438965 0.001675
H -1.343239 5.011749 0.326077

H -1.720664 4.087559 -1.168881
C -2.194546 3.093954 2.026569
H -3.219223 3.456513 1.958151
H -2.188932 2.126253 2.526671
H -1.564600 3.815089 2.546177
I -2.444242 -0.916737 2.993140
C 4.077010 -3.331622 -0.649424
C 3.295488 -3.976542 -1.600418
C 1.992322 -3.555165 -1.873016
C 1.478356 -2.468550 -1.176409
C 2.242333 -1.805410 -0.218646
C 3.533118 -2.246719 0.031949
H 5.086958 -3.664100 -0.440008
H 1.418121 -4.086097 -2.619267
H 1.852459 -0.958410 0.330505
N 0.099119 -1.959365 -1.422772
C -0.697059 -1.960554 -0.138033
H -1.707311 -1.619600 -0.375426
H -0.708437 -2.975092 0.259840
H -0.237619 -1.280031 0.577012
C -0.651283 -2.789018 -2.424289
H -1.646269 -2.351565 -2.517431
H -0.132720 -2.751385 -3.381330
H -0.722618 -3.811283 -2.054825
C 0.161217 -0.548176 -1.948426
H -0.864989 -0.231942 -2.149897
H 0.617697 0.093568 -1.193132
H 0.759352 -0.544353 -2.859073
I -4.044351 -0.683289 -2.086292
Br 4.561728 -1.349350 1.343711
H 3.700214 -4.824321 -2.141488
Br 3.163249 3.487163 -1.549565
H 1.908515 0.525504 2.270763

Br_gen
Electronic energy = -2571.662914
Thermal correction to Gibbs free energy (25°C) = 0.015517
Thermal correction to Gibbs free energy (80°C) = 0.018664
qh-G(25°C) = -2571.676071
qh-G(80°C) = -2571.678782

Geometry:
Br 0.000000 0.000000 0.000000

Cl_gen
Electronic energy = -460.342477
Thermal correction to Gibbs free energy (25°C) = 0.014364

Thermal correction to Gibbs free energy (80°C) = 0.017298
qh-G(25°C) = -460.354481
qh-G(80°C) = -460.356979

Geometry:
Cl 0.000000 0.000000 0.000000

DMSO_GP (c=14.1)
Electronic energy = -553.079007
Thermal correction to Gibbs free energy (25°C) = 0.029252
Thermal correction to Gibbs free energy (80°C) = 0.036535
qh-G(25°C) = -553.021482
qh-G(80°C) = -553.026841

Geometry:

S	-0.000008	0.228718	-0.449229
O	0.000008	1.483617	0.388160
C	-1.351061	-0.801466	0.186069
H	-2.283025	-0.290321	-0.058108
H	-1.328260	-1.785502	-0.287987
H	-1.247068	-0.879602	1.270898
C	1.351068	-0.801469	0.186060
H	1.247045	-0.879564	1.270888
H	1.328300	-1.785518	-0.287967
H	2.283031	-0.290316	-0.058104

DMSO_MeBr_TS
Electronic energy = -3164.455181
Thermal correction to Gibbs free energy (25°C) = 0.043184
Thermal correction to Gibbs free energy (80°C) = 0.054002
qh-G(25°C) = -3164.366501
qh-G(80°C) = -3164.374254

Geometry:

C	-0.359252	-0.021409	0.250910
H	0.062706	-0.013126	-0.754264
H	-0.078516	-0.928996	0.786610
H	-0.056360	0.865521	0.808825
S	-2.134294	-0.000206	0.151405
O	-2.777770	-0.004480	1.472764
C	-2.568848	1.430068	-0.808041
H	-2.263373	2.304516	-0.232158
H	-2.051571	1.383877	-1.767291
H	-3.651677	1.406847	-0.937017
C	-2.604363	-1.403893	-0.830978
H	-2.120404	-1.336116	-1.806221
H	-2.284250	-2.293769	-0.287771

H -3.690933 -1.372416 -0.922060
Br 3.020001 -0.000165 -0.021845

DMSO_MeCl_TS
Electronic energy = -1053.133926
Thermal correction to Gibbs free energy (25°C) = 0.042437
Thermal correction to Gibbs free energy (80°C) = 0.053127
qh-G(25°C) = -1053.044467
qh-G(80°C) = -1053.052095

Geometry:
C 0.462163 -0.020140 0.273282
H 0.899222 -0.012722 -0.725497
H 0.735276 -0.927138 0.813859
H 0.755926 0.867724 0.834547
S -1.310691 -0.000194 0.150989
O -1.970736 -0.004713 1.464195
C -1.735438 1.429347 -0.813765
H -1.439896 2.304258 -0.233448
H -1.205469 1.384816 -1.766155
H -2.816484 1.403554 -0.956840
C -1.768321 -1.404336 -0.836370
H -1.275276 -1.335690 -1.806989
H -1.452715 -2.293984 -0.290231
H -2.854045 -1.374097 -0.937790
Cl 3.743528 -0.000302 -0.047043

DMSO_MeI_TS
Electronic energy = -604.282205
Thermal correction to Gibbs free energy (25°C) = 0.043733
Thermal correction to Gibbs free energy (80°C) = 0.054686
qh-G(25°C) = -604.196707
qh-G(80°C) = -604.204619

Geometry:
C -0.282643 -0.002928 0.105367
H -0.275392 -0.059180 -0.973847
H -0.234354 -0.907519 0.695175
H -0.233026 0.958025 0.598047
S -2.569288 -0.001609 0.171996
O -3.227207 -0.005717 1.520888
C -3.171935 1.396164 -0.777684
H -2.880582 2.296966 -0.236561
H -2.708101 1.378066 -1.766019
H -4.258652 1.321895 -0.848318
C -3.188076 -1.383899 -0.790385
H -2.771246 -1.330572 -1.797946

H -2.860786 -2.295384 -0.289234
H -4.277839 -1.322508 -0.808946
I 2.401550 -0.000459 -0.013494

DMSO_PCM (c=14.1)
Electronic energy = -553.093206
Thermal correction to Gibbs free energy (25°C) = 0.029326
Thermal correction to Gibbs free energy (80°C) = 0.036613
qh-G(25°C) = -553.035735
qh-G(80°C) = -553.041101

Geometry:
S 0.000004 0.211441 -0.451414
O 0.000138 1.494154 0.380569
C -1.357316 -0.790665 0.192305
H -2.284971 -0.276096 -0.059675
H -1.337159 -1.774304 -0.281087
H -1.249357 -0.872812 1.276005
C 1.357199 -0.790865 0.192289
H 1.336762 -1.774606 -0.280871
H 2.284943 -0.276555 -0.059884
H 1.249307 -0.872733 1.276018

H_Ionpair_I_axial
Electronic energy = -417.325011
Thermal correction to Gibbs free energy (25°C) = 0.048307
Thermal correction to Gibbs free energy (80°C) = 0.061340
qh-G(25°C) = -417.141659
qh-G(80°C) = -417.150376

Geometry:
C 5.255964 -0.067650 -0.000355
C 4.601173 1.158968 -0.000402
C 3.205870 1.221255 -0.000326
C 2.475837 0.038084 -0.000203
C 3.120426 -1.199420 -0.000151
C 4.510474 -1.246784 -0.000229
H 6.339806 -0.108048 -0.000414
H 2.728401 2.191520 -0.000366
H 2.562563 -2.129251 -0.000043
H 5.007288 -2.210729 -0.000190
N 0.982196 0.050354 -0.000091
C 0.464279 -0.651434 -1.229191
H -0.626112 -0.602946 -1.201252
H 0.860673 -0.135898 -2.103442
H 0.797639 -1.686796 -1.217436
C 0.407046 1.436716 -0.000189

H -0.678795 1.328468 -0.000105
H 0.733216 1.958821 0.898192
H 0.733108 1.958651 -0.898709
C 0.464491 -0.651180 1.229241
H -0.625908 -0.602799 1.201424
H 0.797960 -1.686510 1.217697
H 0.860944 -0.135394 2.103319
I -3.369198 -0.015215 0.000251
H 5.168006 2.083487 -0.000497

H_SnAr_product_GEN
Electronic energy = -538.309517
Thermal correction to Gibbs free energy (25°C) = 0.043514
Thermal correction to Gibbs free energy (80°C) = 0.055449
qh-G(25°C) = -538.155349
qh-G(80°C) = -538.163278

Geometry:
C -2.598296 1.235003 -0.876236
C -1.350020 0.616939 -0.827358
C -1.189835 -0.525094 -0.042034
C -2.257068 -1.056029 0.678222
C -3.502194 -0.431861 0.614304
C -3.677221 0.716457 -0.158250
H -2.726776 2.123927 -1.485677
H -0.510417 1.013399 -1.389357
H -2.099510 -1.947690 1.276033
H -4.334913 -0.843935 1.175334
H -4.645968 1.202684 -0.202746
O 0.000081 -1.221423 -0.000485
C 1.189878 -0.524990 0.041595
C 2.257671 -1.056285 -0.677537
C 1.349459 0.617578 0.826321
C 3.502732 -0.432000 -0.613131
H 2.100653 -1.948330 -1.274917
C 2.597651 1.235715 0.875700
H 0.509459 1.014333 1.387514
C 3.677156 0.716777 0.158835
H 4.335841 -0.844428 -1.173319
H 2.725684 2.125014 1.484692
H 4.645817 1.203137 0.203739

H_TMA_DMSO_2
Electronic energy = -958.897781
Thermal correction to Gibbs free energy (25°C) = 0.057088
Thermal correction to Gibbs free energy (80°C) = 0.073434
qh-G(25°C) = -958.636725

qh-G(80°C) = -958.647051

Geometry:

C	-1.878837	-0.001391	0.026267
C	-3.189706	0.450557	0.128805
C	-3.429870	1.825982	0.171579
C	-2.375779	2.730978	0.111822
C	-1.066882	2.259129	0.007581
C	-0.809356	0.892431	-0.035812
H	-4.032521	-0.225461	0.177092
H	-0.237129	2.956488	-0.040298
H	0.214391	0.534377	-0.116617
N	-1.561722	-1.462491	-0.022931
C	-0.853549	-1.779479	-1.315876
H	0.102178	-1.256465	-1.330262
H	-0.688760	-2.856607	-1.347921
H	-1.494437	-1.464331	-2.138502
C	-0.660036	-1.822036	1.130893
H	-0.488697	-2.898165	1.095148
H	0.284861	-1.291775	1.017337
H	-1.166007	-1.543712	2.054764
C	-2.785014	-2.322240	0.060398
H	-3.291074	-2.136401	1.006594
H	-3.437420	-2.103829	-0.783613
H	-2.457703	-3.359313	0.014349
H	-4.452388	2.178277	0.252372
O	1.999129	-0.693432	-0.283981
S	3.386760	-0.065469	-0.468091
C	3.132629	1.712162	-0.286809
H	2.535468	2.040912	-1.138316
H	2.601896	1.898106	0.649860
H	4.101580	2.215291	-0.295457
C	4.247954	-0.348620	1.091432
H	4.425057	-1.421405	1.171098
H	5.199455	0.186433	1.076956
H	3.612942	-0.002435	1.909625
H	-2.570158	3.797579	0.145939

H_TMA_DMSO_scan_tsopt_GEN
Electronic energy = -958.828629
Thermal correction to Gibbs free energy (25°C) = 0.057298
Thermal correction to Gibbs free energy (80°C) = 0.073709
qh-G(25°C) = -958.571030
qh-G(80°C) = -958.581435

Geometry:

C	-3.670225	-1.869556	-0.136880
C	-3.538898	-0.986097	-1.203585

C	-2.685896	0.115640	-1.118374
C	-1.949049	0.346702	0.048847
C	-2.080274	-0.548121	1.121913
C	-2.934194	-1.642205	1.026289
H	-4.336275	-2.722750	-0.207481
H	-4.105308	-1.142650	-2.115923
H	-2.618989	0.785992	-1.965471
H	-1.520866	-0.404870	2.039473
H	-3.020364	-2.320078	1.869256
N	-1.014552	1.434675	0.148461
C	-1.086356	2.181615	1.417850
H	-0.314677	2.953113	1.410325
H	-2.068479	2.653188	1.530989
H	-0.902891	1.524979	2.266678
C	-1.006052	2.358797	-0.992347
H	-0.241398	3.116226	-0.814714
H	-0.755928	1.823827	-1.910114
H	-1.975124	2.856150	-1.112041
C	0.873769	0.557505	0.095839
H	0.628285	0.034586	1.012889
H	0.592594	0.129424	-0.859258
H	1.300907	1.552510	0.145343
S	2.878708	-0.352651	0.067490
O	3.653945	-0.162497	1.334211
C	3.820277	0.274787	-1.320900
H	3.956022	1.344624	-1.158253
H	4.783175	-0.238973	-1.334427
H	3.260891	0.092541	-2.240011
C	2.751833	-2.098072	-0.310309
H	3.762717	-2.507765	-0.350317
H	2.179539	-2.558788	0.495496
H	2.237529	-2.217590	-1.265256

H_TMA_Iodide_TS
Electronic energy = -417.272374
Thermal correction to Gibbs free energy (25°C) = 0.049282
Thermal correction to Gibbs free energy (80°C) = 0.062613
qh-G(25°C) = -417.093966
qh-G(80°C) = -417.102939

Geometry:

C	3.917568	-1.958772	-0.115286
C	3.135941	-1.741107	1.020227
C	2.310422	-0.625400	1.109509
C	2.252976	0.304284	0.058913
C	3.036080	0.082380	-1.080309
C	3.859597	-1.042166	-1.160255
H	3.162505	-2.445931	1.845053

H 1.709062 -0.492797 2.001897
H 3.025698 0.777859 -1.909655
H 4.461133 -1.190606 -2.051381
N 1.353161 1.416279 0.151375
C 1.394309 2.353905 -0.974436
H 0.638437 3.122554 -0.807603
H 2.375315 2.836310 -1.062120
H 1.159263 1.835408 -1.905483
C 1.409109 2.140134 1.432279
H 0.641905 2.916272 1.425691
H 1.207492 1.470469 2.266480
H 2.392258 2.604479 1.571759
C -0.597456 0.594502 0.048495
H -0.993743 1.598718 0.064199
H -0.319852 0.135986 -0.888708
H -0.405438 0.074939 0.974552
I -2.969769 -0.395738 -0.083728
H 4.560342 -2.830044 -0.181556

H_TMA_SN2_tsopt_GEN
Electronic energy = -712.645402
Thermal correction to Gibbs free energy (25°C) = 0.056499
Thermal correction to Gibbs free energy (80°C) = 0.072909
qh-G(25°C) = -712.374834
qh-G(80°C) = -712.385095

Geometry:
C -4.421401 -1.665271 -0.249153
C -4.558886 -0.354824 -0.695406
C -3.557973 0.588917 -0.457003
C -2.402447 0.223171 0.238576
C -2.262066 -1.098414 0.683708
C -3.265664 -2.030896 0.441408
H -5.202853 -2.394161 -0.435435
H -5.451198 -0.050832 -1.232844
H -3.703536 1.600415 -0.813169
H -1.373721 -1.415310 1.217972
H -3.138237 -3.048875 0.794730
N -1.316595 1.156644 0.455795
C 0.152054 0.576665 -0.620650
H 0.755525 1.426122 -0.325176
H 0.242322 -0.359649 -0.085521
H -0.431090 0.616193 -1.531115
C -0.831888 1.171182 1.852825
H 0.003932 1.869743 1.918638
H -1.630789 1.489792 2.528746
H -0.478141 0.183882 2.144946
C -1.595224 2.532930 0.012846

H	-1.790151	2.546612	-1.060354
H	-2.448082	2.956405	0.552019
H	-0.710486	3.138117	0.213844
C	2.691427	-0.189600	-0.930085
C	2.953656	-1.453371	-0.347578
C	3.568140	0.870578	-0.594387
C	4.042040	-1.645740	0.500813
H	2.287439	-2.277942	-0.589887
C	4.653458	0.669104	0.255887
H	3.380080	1.849131	-1.029848
C	4.903074	-0.589220	0.810810
H	4.220738	-2.630540	0.925158
H	5.312425	1.502197	0.487266
H	5.750709	-0.743348	1.470772
O	1.651434	0.001783	-1.719620

H_TMA_SnAr_tsopt_GEN
Electronic energy = -712.647013
Thermal correction to Gibbs free energy (25°C) = 0.053513
Thermal correction to Gibbs free energy (80°C) = 0.069474
qh-G(25°C) = -712.373671
qh-G(80°C) = -712.383510

Geometry:

C	0.774336	2.993279	0.297225
C	0.480196	2.096474	1.325018
C	0.638727	0.723625	1.177045
C	1.053810	0.155149	-0.066306
C	1.416628	1.093298	-1.087930
C	1.247042	2.455627	-0.907301
H	0.655832	4.062640	0.429031
H	0.117161	2.466671	2.280985
H	0.361538	0.072087	1.998374
H	1.758789	0.729117	-2.050098
H	1.495015	3.115744	-1.735041
N	2.070947	-1.054064	0.084311
C	3.378266	-0.473769	0.523290
H	4.077247	-1.293337	0.693976
H	3.748178	0.188563	-0.258167
H	3.217307	0.091290	1.440436
C	2.299130	-1.794383	-1.197270
H	3.083796	-2.530575	-1.018868
H	1.372469	-2.275537	-1.492112
H	2.632148	-1.096818	-1.962750
C	1.625883	-2.036817	1.120890
H	1.654637	-1.563049	2.099980
H	0.619595	-2.364586	0.872852
H	2.322591	-2.875601	1.108451

O	-0.185921	-0.926994	-0.705951
C	-1.451016	-0.613082	-0.414625
C	-1.961902	0.695067	-0.537016
C	-2.324867	-1.629977	0.016852
C	-3.298989	0.959269	-0.246362
H	-1.300975	1.495360	-0.856566
C	-3.664003	-1.357346	0.290090
H	-1.931890	-2.638122	0.119047
C	-4.162766	-0.059161	0.163975
H	-3.672026	1.975044	-0.347835
H	-4.319328	-2.163391	0.608868
H	-5.204196	0.155387	0.381431

H_TMA_bromide_tsopt
Electronic energy = -2977.413899
Thermal correction to Gibbs free energy (25°C) = 0.048259
Thermal correction to Gibbs free energy (80°C) = 0.061403
qh-G(25°C) = -2977.234189
qh-G(80°C) = -2977.242996

Geometry:

C	3.506810	-1.796855	-0.122770
C	2.696274	-1.659166	1.004780
C	1.788536	-0.609727	1.101010
C	1.675985	0.331346	0.065565
C	2.488262	0.190119	-1.065459
C	3.394506	-0.868333	-1.152720
H	4.213859	-2.616374	-0.194545
H	2.764661	-2.374728	1.817848
H	1.167955	-0.537555	1.987123
H	2.437008	0.897611	-1.882974
H	4.017114	-0.955558	-2.037385
N	0.691743	1.371806	0.164558
C	0.679341	2.330395	-0.945374
H	-0.132292	3.039305	-0.775544
H	1.623819	2.883265	-1.010889
H	0.493651	1.812915	-1.888103
C	0.687970	2.079599	1.456654
H	-0.132003	2.799801	1.454469
H	0.528436	1.384580	2.279237
H	1.634807	2.609835	1.609643
C	-1.166479	0.423982	0.028028
H	-1.638406	1.394236	0.063368
H	-0.851905	0.007914	-0.917012
H	-0.947268	-0.103964	0.943102
Br	-3.272254	-0.639203	-0.137672

H_TMA_chloride_tsopt
Electronic energy = -866.092733
Thermal correction to Gibbs free energy (25°C) = 0.047095
Thermal correction to Gibbs free energy (80°C) = 0.060020
qh-G(25°C) = -865.911511
qh-G(80°C) = -865.920121

Geometry:

C	3.125919	-1.424502	0.134131
C	2.270839	-1.479017	-0.967359
C	1.197702	-0.600829	-1.074799
C	0.960056	0.357823	-0.077755
C	1.816460	0.408654	1.027827
C	2.889765	-0.478869	1.126763
H	3.962230	-2.110555	0.215074
H	2.434496	-2.212150	-1.750535
H	0.547208	-0.676635	-1.938908
H	1.670639	1.136294	1.815791
H	3.543633	-0.418514	1.990854
N	-0.189527	1.213617	-0.186330
C	-0.340448	1.854273	-1.505154
H	-1.275380	2.417354	-1.511294
H	0.494552	2.536540	-1.699837
H	-0.386410	1.107845	-2.296146
C	-0.330045	2.209523	0.881779
H	-1.249687	2.769745	0.706942
H	-0.405886	1.713903	1.851104
H	0.513347	2.909697	0.892281
C	-1.840035	-0.015110	0.049513
H	-2.471697	0.859481	0.008042
H	-1.568502	-0.529266	-0.859459
H	-1.426750	-0.342055	0.991796
Cl	-3.620963	-1.332275	0.312878

H_TMA_gen
Electronic energy = -405.793570
Thermal correction to Gibbs free energy (25°C) = 0.041140
Thermal correction to Gibbs free energy (80°C) = 0.052553
qh-G(25°C) = -405.605865
qh-G(80°C) = -405.613462

Geometry:

C	-2.873922	-0.004522	0.000026
C	-2.188304	1.205072	-0.000153
C	-0.791894	1.232263	-0.000472
C	-0.093499	0.030382	-0.000563
C	-0.768368	-1.190579	-0.000298
C	-2.159105	-1.202302	-0.000085

H	-3.958387	-0.017315	0.000294
H	-2.731190	2.143750	0.000009
H	-0.291199	2.190676	-0.000573
H	-0.236265	-2.135265	-0.000152
H	-2.680003	-2.153343	0.000066
N	1.402834	0.003131	-0.000016
C	1.895130	-0.715493	-1.229243
H	2.984356	-0.698633	-1.217271
H	1.538316	-1.742489	-1.213762
H	1.509464	-0.193139	-2.103745
C	1.893511	-0.709008	1.233601
H	2.982774	-0.694320	1.221903
H	1.508558	-0.180660	2.104826
H	1.534315	-1.735247	1.224069
C	2.008860	1.375752	-0.003356
H	1.696748	1.903741	-0.902777
H	1.697589	1.907713	0.893985
H	3.090635	1.253228	-0.003498

H_aniline_gen
Electronic energy = -366.066276
Thermal correction to Gibbs free energy (25°C) = 0.039306
Thermal correction to Gibbs free energy (80°C) = 0.049797
qh-G(25°C) = -365.921062
qh-G(80°C) = -365.928275

Geometry:

C	2.649010	-0.000010	0.049710
C	1.934786	1.197740	0.020633
C	0.543972	1.207710	-0.041808
C	-0.187825	0.000029	-0.089719
C	0.543942	-1.207675	-0.041809
C	1.934747	-1.197747	0.020633
H	3.732455	-0.000033	0.101586
H	2.462901	2.146455	0.052913
H	0.030861	2.161324	-0.054765
H	0.030779	-2.161265	-0.054771
H	2.462845	-2.146472	0.052912
N	-1.570483	0.000007	-0.192263
C	-2.280373	-1.239068	0.076124
H	-3.350627	-1.061376	-0.025960
H	-2.002763	-2.010674	-0.647267
H	-2.083058	-1.625541	1.087524
C	-2.280463	1.239038	0.076129
H	-2.083143	1.625536	1.087516
H	-2.002946	2.010652	-0.647286
H	-3.350705	1.061255	-0.025923

H_bromide_ionpair_gen
Electronic energy = -2977.467795
Thermal correction to Gibbs free energy (25°C) = 0.047378
Thermal correction to Gibbs free energy (80°C) = 0.060259
qh-G(25°C) = -2977.283164
qh-G(80°C) = -2977.291752

Geometry:
Br -3.055116 -0.446577 -0.000002
N 0.828887 1.477045 0.000009
C 1.498882 0.139158 0.000039
C 2.884533 0.024174 0.000033
C 3.457604 -1.249764 0.000006
H 4.538415 -1.339417 -0.000003
C 2.656528 -2.386734 -0.000020
H 3.108941 -3.372609 -0.000045
C 1.267990 -2.251489 -0.000009
H 0.631525 -3.129981 -0.000024
C -0.036521 1.604302 1.227868
H -0.471487 2.603803 1.227809
H 0.594833 1.458941 2.103691
H -0.828620 0.857134 1.180565
C -0.036430 1.604288 -1.227923
H -0.471345 2.603812 -1.227919
H -0.828559 0.857145 -1.180658
H 0.595001 1.458899 -2.103687
C 1.804066 2.614337 0.000007
H 1.227427 3.537858 0.000009
H 2.417318 2.562476 -0.898522
H 2.417337 2.562468 0.898524
C 0.680666 -0.990273 0.000024
H -0.403646 -0.905234 0.000090
H 3.535817 0.887601 0.000043

H_chloride_ionpair_gen
Electronic energy = -866.145875
Thermal correction to Gibbs free energy (25°C) = 0.046630
Thermal correction to Gibbs free energy (80°C) = 0.059411
qh-G(25°C) = -865.960700
qh-G(80°C) = -865.969176

Geometry:
Cl -3.328917 -1.265421 0.000147
N -0.118598 1.383594 -0.000056
C 0.841576 0.236278 -0.000050
C 2.216620 0.443931 0.000201
C 3.067866 -0.663584 0.000230

H	4.140245	-0.501844	0.000429
C	2.550567	-1.954641	0.000008
H	3.218197	-2.809595	0.000023
C	1.168250	-2.143412	-0.000236
H	0.751864	-3.145108	-0.000409
C	-0.990444	1.308431	1.227847
H	-1.639809	2.184135	1.229281
H	-0.342353	1.308581	2.103620
H	-1.593170	0.401539	1.176762
C	-0.990448	1.308433	-1.227949
H	-1.639765	2.184170	-1.229416
H	-1.593243	0.401591	-1.176827
H	-0.342368	1.308511	-2.103730
C	0.568695	2.714092	-0.000056
H	-0.205096	3.479794	-0.000125
H	1.177641	2.804820	-0.898218
H	1.177495	2.804905	0.898197
C	0.305570	-1.051555	-0.000266
H	-0.770033	-1.216116	-0.000437
H	2.652666	1.433783	0.000386

H_ionpair_Br_axial
Electronic energy = -2977.467146
Thermal correction to Gibbs free energy (25°C) = 0.047421
Thermal correction to Gibbs free energy (80°C) = 0.060304
qh-G(25°C) = -2977.282808
qh-G(80°C) = -2977.291381

Geometry:

C	4.617613	-0.075180	-0.000082
C	3.966908	1.153627	-0.000062
C	2.571755	1.220575	-0.000011
C	1.837427	0.040022	0.000017
C	2.478320	-1.199504	0.000001
C	3.868164	-1.251833	-0.000048
H	5.701323	-0.119224	-0.000120
H	2.097238	2.192327	0.000006
H	1.917027	-2.127291	0.000023
H	4.361652	-2.217498	-0.000061
N	0.344377	0.056946	0.000068
C	-0.177011	-0.642870	-1.229066
H	-1.266985	-0.590638	-1.196115
H	0.220028	-0.127638	-2.103243
H	0.154670	-1.678823	-1.218242
C	-0.228116	1.444677	0.000144
C	-0.176930	-0.642967	1.229179
H	-1.266902	-0.590623	1.196386
H	0.154631	-1.678957	1.218187

H 0.220272 -0.127885 2.103369
H -1.313765 1.335956 0.000185
H 0.100090 1.965847 0.898377
H 0.100004 1.965910 -0.898083
H 4.536959 2.076184 -0.000086
Br -3.733590 -0.027302 -0.000043

H_ionpair_Cl_axial
Electronic energy = -866.145694
Thermal correction to Gibbs free energy (25°C) = 0.046564
Thermal correction to Gibbs free energy (80°C) = 0.059314
qh-G(25°C) = -865.960492
qh-G(80°C) = -865.968934

Geometry:
C 3.860613 -0.057759 -0.001501
C 3.201199 1.166384 -0.001153
C 1.805580 1.223358 -0.000236
C 1.079527 0.037692 0.000324
C 1.729261 -1.197221 -0.000006
C 3.119500 -1.239664 -0.000908
H 4.944616 -0.094054 -0.002206
H 1.324321 2.191755 0.000034
H 1.174706 -2.129013 0.000419
H 3.619836 -2.201800 -0.001152
N -0.413659 0.044347 0.001284
C -0.931724 -0.658542 -1.227428
H -2.021986 -0.613492 -1.191495
H -0.539466 -0.140091 -2.101877
H -0.592193 -1.691995 -1.217654
C -0.996161 1.428137 0.002430
C -0.930178 -0.659809 1.229921
H -2.020466 -0.614158 1.195994
H -0.591378 -1.693481 1.218312
H -0.536213 -0.142723 2.104406
H -2.080823 1.309693 0.003452
H -0.670208 1.951086 0.900461
H -0.672025 1.951815 -0.895829
H 3.764646 2.092997 -0.001591
Cl -4.343145 -0.043672 -0.001701

H_ionpair_displ
Electronic energy = -417.325562
Thermal correction to Gibbs free energy (25°C) = 0.048203
Thermal correction to Gibbs free energy (80°C) = 0.061227
qh-G(25°C) = -417.141914
qh-G(80°C) = -417.150632

Geometry:
C 3.056306 -2.488855 0.000004
C 1.678030 -2.272722 -0.000348
C 1.166441 -0.978950 -0.000372
C 2.048973 0.101034 -0.000010
C 3.425369 -0.095427 0.000357
C 3.922524 -1.400799 0.000353
H 4.126878 0.727744 0.000669
H 4.996120 -1.553913 0.000632
H 3.450317 -3.499448 0.000001
H 0.990588 -3.111935 -0.000592
N 1.460646 1.476805 -0.000049
C 0.606061 1.657029 1.228568
H 0.222528 2.677397 1.223067
H 1.231119 1.484210 2.104112
H -0.223811 0.951599 1.191491
C 0.606628 1.657116 -1.229077
H 0.223656 2.677687 -1.223979
H -0.223593 0.952124 -1.192062
H 1.231955 1.483732 -2.104304
C 2.501737 2.554372 0.000184
H 1.980301 3.510023 0.000270
H 3.110654 2.467001 -0.898376
H 3.110468 2.466768 0.898845
H 0.088831 -0.832237 -0.000630
I -2.804018 -0.247834 0.000061

H_ionpair_displ_conf2_DMSO1
Electronic energy = -970.430232
Thermal correction to Gibbs free energy (25°C) = 0.063637
Thermal correction to Gibbs free energy (80°C) = 0.081477
qh-G(25°C) = -970.173131
qh-G(80°C) = -970.184435

Geometry:
C 5.072292 -0.504486 -0.184673
C 4.076048 0.472470 -0.166392
C 2.735293 0.111321 -0.081150
C 2.396958 -1.240747 -0.013400
C 3.376816 -2.226665 -0.031618
C 4.718758 -1.847888 -0.117726
H 3.134144 -3.279553 0.018929
H 5.482554 -2.617772 -0.131583
H 4.337899 1.523955 -0.218530
N 0.948742 -1.587640 0.079691
C 0.218600 -1.054198 -1.127339
H -0.817611 -1.385466 -1.042804

H 0.696459 -1.464852 -2.016768
H 0.271559 0.034705 -1.120889
C 0.360715 -0.975690 1.326514
H -0.682737 -1.290711 1.374011
H 0.426866 0.109578 1.252789
H 0.928379 -1.346133 2.180106
C 0.695803 -3.063837 0.140714
H -0.384517 -3.191220 0.209792
H 1.183407 -3.473836 1.024244
H 1.075939 -3.529443 -0.767818
H 1.972562 0.886564 -0.068097
I -0.329899 2.832074 0.053763
O -2.446599 -2.233589 0.267336
S -3.682466 -1.339561 0.442223
C -3.419154 0.060858 -0.663989
H -4.311681 0.690123 -0.659796
H -2.564667 0.627561 -0.283603
H -3.212388 -0.316793 -1.668443
C -4.995445 -2.149740 -0.491562
H -5.869123 -1.495796 -0.518469
H -4.629149 -2.359788 -1.498658
H -5.238672 -3.076360 0.028751
H 6.116388 -0.217887 -0.250980

I_DMSO
Electronic energy = -564.619618
Thermal correction to Gibbs free energy (25°C) = 0.040254
Thermal correction to Gibbs free energy (80°C) = 0.049905
qh-G(25°C) = -564.570338
qh-G(80°C) = -564.577550

Geometry:
I -1.845992 0.000001 -0.057511
H 0.804009 -1.307632 1.023255
C 1.857046 -1.353253 0.737175
H 2.517583 -1.247755 1.600993
H 2.064853 -2.284633 0.209177
S 2.192754 -0.000001 -0.408524
O 3.711888 -0.000077 -0.615988
C 1.857165 1.353314 0.737093
H 2.064523 2.284741 0.209005
H 0.804206 1.307467 1.023587
H 2.517981 1.248031 1.600730

I_gen
Electronic energy = -11.518911
Thermal correction to Gibbs free energy (25°C) = 0.016190

Thermal correction to Gibbs free energy (80°C) = 0.019460
qh-G(25°C) = -11.532741
qh-G(80°C) = -11.535576

Geometry:
I 0.000000 0.000000 0.000000

K
Electronic energy = -599.824281
Thermal correction to Gibbs free energy (25°C) = 0.014518
Thermal correction to Gibbs free energy (80°C) = 0.017480
qh-G(25°C) = -599.836438
qh-G(80°C) = -599.838965

Geometry:
K 0.000000 0.000000 0.000000

K1_TS_3Br_trimethylanilinium_iodide
Electronic energy = -2988.154345
Thermal correction to Gibbs free energy (25°C) = 0.053161
Thermal correction to Gibbs free energy (80°C) = 0.067557
qh-G(25°C) = -2987.988374
qh-G(80°C) = -2987.998069

Geometry:
C 2.864913 -0.268004 -1.742366
C 2.911941 -0.203675 -0.357449
C 1.970711 0.499415 0.389254
C 0.934706 1.169394 -0.272792
C 0.872960 1.112718 -1.673778
C 1.828308 0.403031 -2.390704
H 3.611453 -0.821293 -2.299681
H 2.067344 0.521999 1.466032
H 0.080353 1.613193 -2.216788
H 1.763758 0.368222 -3.473069
N -0.090928 1.850672 0.454991
C -0.468263 3.160439 -0.106702
H -1.267120 3.580291 0.506101
H 0.388535 3.843215 -0.103887
H -0.842275 3.053330 -1.123523
C 0.117970 1.944340 1.904535
H -0.742467 2.452364 2.341261
H 0.185392 0.947073 2.342406
H 1.024581 2.512882 2.142998
C -1.798721 0.644979 0.260490
Br 4.312540 -1.097190 0.560222
H -2.358519 1.356562 0.849569

H -1.741087 0.756385 -0.811697
H -1.264092 -0.157687 0.745942
I -3.898404 -0.855763 0.014692

K1_TS_4CHO_trimethylanilinium_iodide
Electronic energy = -530.563002
Thermal correction to Gibbs free energy (25°C) = 0.054113
Thermal correction to Gibbs free energy (80°C) = 0.068918
qh-G(25°C) = -530.377681
qh-G(80°C) = -530.387524

Geometry:
C 3.723992 -0.732207 -0.215973
C 2.986371 -0.690292 0.973640
C 1.920057 0.182188 1.104863
C 1.563535 1.039535 0.045370
C 2.298518 0.991095 -1.147200
C 3.368904 0.109830 -1.268902
H 3.258440 -1.348298 1.792994
H 1.365023 0.189393 2.034836
H 2.061879 1.638615 -1.980866
H 3.937423 0.087156 -2.195036
N 0.431585 1.895461 0.187986
C 0.234766 2.870439 -0.893474
H -0.667495 3.442257 -0.675969
H 1.084834 3.557791 -0.968529
H 0.092924 2.356905 -1.845082
C 0.308739 2.539827 1.510010
H -0.574709 3.178797 1.499150
H 0.176756 1.796866 2.294746
H 1.193754 3.147186 1.726470
C -1.243854 0.659982 0.055426
H -1.871777 1.520158 0.233005
H -0.968288 0.387716 -0.953056
H -0.881337 0.073653 0.886623
C 4.869062 -1.649233 -0.372501
O 5.256417 -2.415024 0.490092
H 5.380343 -1.604436 -1.352450
I -3.374354 -0.835826 -0.084548

K1_TS_Br1_DMSO2_OPT
Electronic energy = -3541.258514
Thermal correction to Gibbs free energy (25°C) = 0.068109
Thermal correction to Gibbs free energy (80°C) = 0.087262
qh-G(25°C) = -3541.018750
qh-G(80°C) = -3541.030945

Geometry:
C -1.623658 1.819828 1.652516
C -0.737335 1.033033 2.388570
C -0.034920 -0.007952 1.792317
C -0.196465 -0.285305 0.424634
C -1.076939 0.504829 -0.325282
C -1.779892 1.527935 0.304784
H -0.595093 1.234225 3.445025
H 0.641657 -0.595344 2.401540
H -1.250581 0.317304 -1.376341
N 0.580599 -1.313336 -0.186107
C 0.316184 -1.546597 -1.611855
H 1.010206 -2.313012 -1.960630
H -0.712574 -1.893278 -1.760867
H 0.502163 -0.633357 -2.180710
C 0.593009 -2.588537 0.556070
H 1.274560 -3.272366 0.046483
H 0.958529 -2.441283 1.571385
H -0.416418 -3.012534 0.575297
C 2.579356 -0.615468 -0.155582
H 2.863548 -1.502733 -0.701210
H 2.252011 0.265436 -0.687274
H 2.551486 -0.630786 0.923070
I 5.017157 0.213140 -0.134013
S -3.632580 -1.542613 -0.317124
O -2.519128 -2.587226 -0.430549
C -3.754094 -1.155717 1.441401
H -4.561671 -0.436977 1.597482
H -3.933392 -2.079252 1.996424
H -2.799415 -0.715183 1.735315
C -5.180271 -2.459456 -0.470041
H -5.158430 -3.301305 0.225243
H -6.014935 -1.790202 -0.252043
H -5.246009 -2.815329 -1.498560
H -2.181006 2.624293 2.117304
Br -3.014677 2.540247 -0.722238

K1_TS_CHO2_DMSO2_OPT
Electronic energy = -1083.665724
Thermal correction to Gibbs free energy (25°C) = 0.068976
Thermal correction to Gibbs free energy (80°C) = 0.088534
qh-G(25°C) = -1083.406671
qh-G(80°C) = -1083.419009

Geometry:
C -1.867517 2.485016 -0.288482
C -1.189024 2.166061 0.895429
C -0.560153 0.940442 1.033700

C	-0.585511	-0.001655	-0.015868
C	-1.259400	0.324042	-1.201949
C	-1.895398	1.555456	-1.328749
H	-0.042188	0.722732	1.959889
H	-1.317890	-0.380653	-2.020809
N	0.134772	-1.219242	0.120453
C	-0.000073	-2.169768	-0.993226
H	0.636971	-3.029227	-0.779579
H	-1.040025	-2.498454	-1.088601
H	0.346140	-1.710164	-1.920754
C	-0.036170	-1.901854	1.418392
H	0.610315	-2.781018	1.426553
H	0.265718	-1.254548	2.240248
H	-1.081143	-2.204887	1.537572
C	2.161977	-0.667439	0.051621
H	2.424343	-1.713043	0.112997
H	1.988438	-0.207856	-0.909803
H	2.031926	-0.089066	0.953834
I	4.668297	-0.019567	-0.045395
S	-4.078575	-1.333103	-0.355284
O	-2.977245	-2.274794	0.137864
C	-4.270321	-0.097897	0.945592
H	-5.102197	0.564721	0.698095
H	-4.441890	-0.610773	1.894659
H	-3.338621	0.469363	0.983655
C	-5.630892	-2.213873	-0.081107
H	-5.657668	-2.563247	0.953243
H	-6.465414	-1.542103	-0.291675
H	-5.649281	-3.058840	-0.769930
H	-2.427638	1.787839	-2.247689
H	-1.162813	2.888823	1.704891
C	-2.552526	3.780422	-0.450278
O	-2.587606	4.647999	0.402707
H	-3.054177	3.932465	-1.424492

K1_TS_H_DMSO2_OPT_B
Electronic energy = -970.374865
Thermal correction to Gibbs free energy (25°C) = 0.064589
Thermal correction to Gibbs free energy (80°C) = 0.082760
qh-G(25°C) = -970.123105
qh-G(80°C) = -970.134668

Geometry:

C	-1.850378	3.055876	-0.311353
C	-1.130780	2.695229	0.829468
C	-0.591717	1.418496	0.952854
C	-0.763883	0.467587	-0.067147
C	-1.478065	0.836341	-1.213998

C	-2.016654	2.119359	-1.327765
H	-0.984980	3.410915	1.632244
H	-0.034429	1.171260	1.849818
H	-1.645316	0.127076	-2.014899
H	-2.578808	2.376070	-2.220267
N	-0.155560	-0.818928	0.064616
C	-0.366215	-1.735126	-1.061741
H	0.200079	-2.647550	-0.864826
H	-1.428464	-1.982002	-1.167177
H	0.014919	-1.286715	-1.981860
C	-0.438985	-1.495175	1.343026
H	0.154791	-2.410898	1.386606
H	-0.153110	-0.864824	2.184180
H	-1.505297	-1.740010	1.403335
C	1.955884	-0.481480	0.036221
H	2.080894	-1.553568	0.054439
H	1.815233	0.031171	-0.903399
H	1.855300	0.064100	0.961707
I	4.483503	-0.108771	-0.018896
S	-4.407759	-0.567513	-0.326255
O	-3.423494	-1.697277	-0.014639
C	-4.310695	0.562013	1.077678
H	-5.074862	1.335060	0.972171
H	-4.452655	-0.010855	1.997111
H	-3.317010	1.013473	1.057211
C	-6.046018	-1.242289	0.024769
H	-6.040525	-1.672202	1.028717
H	-6.788005	-0.445544	-0.058176
H	-6.243440	-2.014423	-0.719382
H	-2.273931	4.050261	-0.403253

K1_TS_H_trimethylanilinium_iodide
Electronic energy = -417.272374
Thermal correction to Gibbs free energy (25°C) = 0.049282
Thermal correction to Gibbs free energy (80°C) = 0.062613
qh-G(25°C) = -417.093966
qh-G(80°C) = -417.102939

Geometry:

C	3.917568	-1.958772	-0.115286
C	3.135941	-1.741107	1.020227
C	2.310422	-0.625400	1.109509
C	2.252976	0.304284	0.058913
C	3.036080	0.082380	-1.080309
C	3.859597	-1.042166	-1.160255
H	3.162505	-2.445931	1.845053
H	1.709062	-0.492797	2.001897
H	3.025698	0.777859	-1.909655

H	4.461133	-1.190606	-2.051381
N	1.353161	1.416279	0.151375
C	1.394309	2.353905	-0.974436
H	0.638437	3.122554	-0.807603
H	2.375315	2.836310	-1.062120
H	1.159263	1.835408	-1.905483
C	1.409109	2.140134	1.432279
H	0.641905	2.916272	1.425691
H	1.207492	1.470469	2.266480
H	2.392258	2.604479	1.571759
C	-0.597456	0.594502	0.048495
H	-0.993743	1.598718	0.064199
H	-0.319852	0.135986	-0.888708
H	-0.405438	0.074939	0.974552
I	-2.969769	-0.395738	-0.083728
H	4.560342	-2.830044	-0.181556

K2_TS_4CHO_DMSO2_OPT
Electronic energy = -1095.200073
Thermal correction to Gibbs free energy (25°C) = 0.074159
Thermal correction to Gibbs free energy (80°C) = 0.095046
qh-G(25°C) = -1094.943370
qh-G(80°C) = -1094.956552

Geometry:

C	3.198167	2.136065	-0.526283
C	3.634789	1.224297	0.440875
C	2.750409	0.713760	1.380597
C	1.398776	1.106286	1.369144
C	0.963577	2.028586	0.402152
C	1.857055	2.532263	-0.533518
H	4.676153	0.917000	0.450109
H	3.115763	0.001166	2.107373
H	-0.077448	2.329086	0.347701
H	1.503239	3.234871	-1.284249
N	0.454308	0.544655	2.270867
C	-0.527793	1.501769	2.812545
H	-1.189443	0.962578	3.492017
H	-0.022090	2.303745	3.362369
H	-1.136439	1.924544	2.012263
C	1.012063	-0.320648	3.317913
H	0.183056	-0.723177	3.901431
H	1.551417	-1.148999	2.854826
H	1.676470	0.240512	3.986234
C	-0.698293	-0.796057	1.116000
H	-1.328656	-0.972169	1.977580
H	-0.963501	0.007019	0.437209
H	0.199963	-1.385899	0.975217

C	4.124211	2.680448	-1.535186
O	5.305040	2.392823	-1.610475
H	3.676277	3.393460	-2.252843
I	-2.053109	-2.513640	-0.282617
I	-2.879235	2.280933	-0.608397
O	2.047154	-2.343181	0.861601
S	2.767208	-2.186646	-0.483120
C	1.678866	-1.187041	-1.523005
H	1.652924	-0.179866	-1.102609
H	2.091212	-1.156362	-2.533825
H	0.678474	-1.630456	-1.517294
C	2.514953	-3.757461	-1.336491
H	2.901670	-3.680708	-2.354454
H	3.066064	-4.518160	-0.782966
H	1.445436	-3.981720	-1.335938

K2_TS_4CHO_Ionpair_Iodide
Electronic energy = -542.090324
Thermal correction to Gibbs free energy (25°C) = 0.060735
Thermal correction to Gibbs free energy (80°C) = 0.077091
qh-G(25°C) = -541.909004
qh-G(80°C) = -541.919869

Geometry:

C	-1.408141	2.921862	0.217150
C	-1.142657	2.195240	1.379915
C	-0.632312	0.905306	1.311723
C	-0.382785	0.310613	0.064516
C	-0.637404	1.050737	-1.106570
C	-1.144781	2.336953	-1.026919
H	-0.456395	0.367068	2.233431
H	-0.462852	0.621611	-2.084415
H	-1.351177	2.898576	-1.932716
N	0.188454	-0.987378	-0.015369
C	-0.029033	-1.847843	1.160781
H	0.403134	-2.826360	0.951114
H	0.479435	-1.437503	2.033918
H	-1.100380	-1.953984	1.362537
C	2.255130	-0.650306	-0.016690
H	2.415106	-1.717100	-0.069699
H	2.153044	-0.072449	-0.923424
H	2.167286	-0.162708	0.943099
C	-0.055085	-1.712035	-1.275506
H	0.385519	-2.705049	-1.186707
H	-1.130671	-1.801525	-1.461915
H	0.430931	-1.204365	-2.109113
I	-3.939408	-1.428905	-0.027732
I	4.822289	-0.252254	-0.018330

H -1.344115 2.638327 2.351871
C -1.959025 4.283449 0.319520
O -2.218752 4.994724 -0.634564
H -2.134160 4.646857 1.349884

K2_TS_Br1_Ionpair_Iodide
Electronic energy = -2999.684711
Thermal correction to Gibbs free energy (25°C) = 0.059417
Thermal correction to Gibbs free energy (80°C) = 0.075282
qh-G(25°C) = -2999.522365
qh-G(80°C) = -2999.533013

Geometry:
C 3.471868 -1.914676 -0.934541
C 2.971467 -3.115807 -0.444656
C 1.876889 -3.144028 0.419917
C 1.258683 -1.950254 0.809168
C 1.748694 -0.730665 0.314542
C 2.840690 -0.738404 -0.539826
H 4.324720 -1.890808 -1.602585
H 1.527732 -4.100898 0.784872
H 1.276165 0.213596 0.570622
N 0.100374 -1.935490 1.647450
C -0.335722 -3.246762 2.135925
H -1.241906 -3.107010 2.726792
H -0.568480 -3.902953 1.295136
H 0.427643 -3.716265 2.767923
C -1.492313 -1.299756 0.413686
H -2.176631 -1.556666 1.209024
H -1.064739 -0.304802 0.373880
H -1.194728 -2.051970 -0.301712
C 0.154280 -0.955308 2.746874
H -0.810326 -0.961464 3.258395
H 0.944015 -1.220679 3.459613
H 0.324614 0.049339 2.360321
I -0.369801 2.671254 0.807871
I -3.463172 -0.586008 -1.095298
H 3.443140 -4.049177 -0.733566
Br 3.493328 0.920116 -1.199821

K2_TS_Br2_DMSO2_OPT
Electronic energy = -3552.792554
Thermal correction to Gibbs free energy (25°C) = 0.073641
Thermal correction to Gibbs free energy (80°C) = 0.094175
qh-G(25°C) = -3552.555544
qh-G(80°C) = -3552.568628

Geometry:

C	2.600490	2.172629	-1.166589
C	3.121991	1.372510	-0.158573
C	2.359105	0.933542	0.919181
C	1.006832	1.295688	0.992338
C	0.462903	2.105046	-0.017629
C	1.255597	2.532645	-1.077139
H	2.819198	0.304185	1.668004
H	-0.586988	2.378118	-0.005248
N	0.166617	0.807003	2.034502
C	-0.779893	1.798340	2.573530
H	-1.367460	1.316807	3.356967
H	-0.244793	2.655525	2.999278
H	-1.467046	2.136879	1.796837
C	0.850076	0.067506	3.101055
H	0.095812	-0.288003	3.804509
H	1.365004	-0.794898	2.672978
H	1.562026	0.706575	3.638613
C	-1.054658	-0.681761	1.135047
H	-1.582338	-0.780189	2.074292
H	-1.410609	0.048140	0.416443
H	-0.146905	-1.248565	0.964931
I	-2.462218	-2.561786	0.067414
I	-3.478698	2.177932	-0.659496
O	1.726239	-2.174601	0.775154
S	2.314980	-2.120747	-0.639782
C	1.096151	-1.266386	-1.664141
H	1.063689	-0.223405	-1.343337
H	1.416744	-1.323707	-2.706665
H	0.120485	-1.741733	-1.524604
C	2.056741	-3.773523	-1.319000
H	2.340299	-3.778930	-2.373268
H	2.692248	-4.457936	-0.756565
H	1.003542	-4.035851	-1.191888
H	3.218088	2.499790	-1.994574
Br	4.951293	0.861822	-0.239369
H	0.814399	3.149955	-1.853181

K2_TS_CHO2_Ionpair_Iodide
Electronic energy = -542.092672
Thermal correction to Gibbs free energy (25°C) = 0.060496
Thermal correction to Gibbs free energy (80°C) = 0.076802
qh-G(25°C) = -541.911318
qh-G(80°C) = -541.922141

Geometry:

C	4.073743	0.041032	-0.834089
C	4.149014	-1.087570	-0.013991

C 3.143213 -1.360290 0.903890
C 2.038483 -0.498983 1.018357
C 1.960153 0.632794 0.189554
C 2.970666 0.894116 -0.724250
H 3.236000 -2.236659 1.531631
H 1.104512 1.299732 0.236449
N 0.963641 -0.779782 1.912583
C 1.140350 -1.957015 2.769938
H 0.238681 -2.079176 3.371071
H 1.272181 -2.851786 2.159257
H 1.998630 -1.838978 3.441615
C -0.664285 -1.284468 0.685409
H -1.208456 -1.596149 1.565086
H -0.734184 -0.257916 0.345372
H -0.032273 -1.990956 0.167833
C 0.498753 0.379753 2.697640
H -0.378802 0.074707 3.270670
H 1.283586 0.712737 3.386275
H 0.205955 1.198618 2.041361
I -1.514244 2.710390 -0.028884
I -2.679700 -1.958408 -0.809319
H 2.897762 1.769787 -1.364575
H 5.005887 -1.748942 -0.096500
C 5.132709 0.342518 -1.814640
O 6.126550 -0.340061 -1.983701
H 4.974831 1.257549 -2.416196

K2_TS_H_DMSO2_OPT
Electronic energy = -981.903725
Thermal correction to Gibbs free energy (25°C) = 0.071671
Thermal correction to Gibbs free energy (80°C) = 0.091442
qh-G(25°C) = -981.656211
qh-G(80°C) = -981.668878

Geometry:
C -1.119869 3.391287 -1.709397
C 0.113608 3.321072 -1.057891
C 0.331508 2.395211 -0.042351
C -0.696627 1.521545 0.348599
C -1.931975 1.585635 -0.307788
C -2.133379 2.516705 -1.328997
H 0.918412 3.991682 -1.345315
H 1.305836 2.336957 0.434624
H -2.751848 0.927741 -0.039564
H -3.100849 2.550851 -1.821625
N -0.435936 0.544679 1.359648
C -1.574558 -0.303373 1.714606
H -1.242480 -1.033979 2.454479

H -2.400390 0.282059 2.139757
H -1.942164 -0.835024 0.834441
C 0.234981 1.067173 2.560170
H 0.459476 0.226430 3.220505
H 1.174722 1.550193 2.294148
H -0.414811 1.775427 3.089559
C 1.007349 -0.807463 0.500524
H 0.784532 -1.487051 1.310469
H 0.390282 -0.825155 -0.386117
H 1.751986 -0.030813 0.637801
I 2.709098 -2.419818 -0.503710
I -5.315348 -0.669588 -0.076582
O 3.241476 1.457610 1.246308
S 4.422182 1.844585 0.345364
C 3.862049 1.519897 -1.340444
H 3.071675 2.237573 -1.564422
H 4.698281 1.659464 -2.028659
H 3.478360 0.496597 -1.387150
C 5.596525 0.481482 0.475263
H 6.405177 0.638108 -0.241541
H 5.991175 0.492994 1.491732
H 5.064153 -0.452465 0.276051
H -1.283109 4.114898 -2.501260

K2_TS_H_Ionpair_Iodide
Electronic energy = -428.801547
Thermal correction to Gibbs free energy (25°C) = 0.055859
Thermal correction to Gibbs free energy (80°C) = 0.070723
qh-G(25°C) = -428.627165
qh-G(80°C) = -428.637158

Geometry:
C -4.220987 -0.109536 -1.972592
C -4.517980 0.980373 -1.160653
C -3.737240 1.269232 -0.039077
C -2.640281 0.461482 0.282425
C -2.335990 -0.633056 -0.542801
C -3.123037 -0.912011 -1.654834
H -4.831200 -0.331345 -2.841799
H -4.004140 2.120240 0.574603
H -1.475018 -1.261738 -0.332091
H -2.868837 -1.762061 -2.280710
N -1.785150 0.753643 1.394182
C -2.163465 1.924584 2.186998
H -1.413297 2.068232 2.966294
H -2.183935 2.815552 1.556105
H -3.142643 1.792160 2.664122
C 0.102458 1.285481 0.558785

H 0.398527 1.630827 1.538546
H 0.258508 0.248768 0.285013
H -0.428876 1.951125 -0.105129
C -1.507959 -0.400668 2.264897
H -0.760557 -0.103623 3.004163
H -2.420644 -0.720525 2.782859
H -1.100488 -1.229675 1.686720
I 1.155829 -2.682818 0.067855
I 2.366856 1.978934 -0.434031
H -5.366775 1.617133 -1.389480

K3_TS_3Br_Ionpair_anilinium
Electronic energy = -5964.841496
Thermal correction to Gibbs free energy (25°C) = 0.078532
Thermal correction to Gibbs free energy (80°C) = 0.101856
qh-G(25°C) = -5964.478493
qh-G(80°C) = -5964.492635

Geometry:
C 4.264254 0.265814 1.096895
C 3.901127 -0.709911 0.180124
C 2.683171 -1.382243 0.259534
C 1.808029 -1.035348 1.281418
C 2.137207 -0.046955 2.207945
C 3.368269 0.592086 2.112517
H 5.219305 0.771422 1.015202
H 2.452646 -2.137987 -0.477905
H 1.457804 0.236454 3.004048
H 3.628737 1.358008 2.834666
N 0.482807 -1.712258 1.414569
C 0.375531 -2.361247 2.771281
H -0.587386 -2.868827 2.823398
H 1.193970 -3.072541 2.873508
H 0.435217 -1.597129 3.542509
C 0.265680 -2.781116 0.382236
H -0.724396 -3.201596 0.554371
H 0.299474 -2.333176 -0.609881
H 1.028327 -3.549975 0.498183
C -0.618012 -0.697678 1.253507
H -1.568863 -1.217082 1.380770
H -0.505019 0.088109 1.999739
H -0.539510 -0.279114 0.249636
C 0.459259 2.832469 1.245283
C 0.272603 2.403640 -0.064969
C -0.989383 2.259193 -0.624861
C -2.126721 2.525620 0.155240
C -1.955054 2.940007 1.482767
C -0.673265 3.094930 2.009721

H 1.455873 2.952831 1.653616
H -1.070990 1.923646 -1.651233
H -2.804601 3.161941 2.114327
H -0.560152 3.430359 3.035185
N -3.423131 2.293050 -0.398242
C -3.609105 0.199079 -0.462857
H -4.605502 0.299472 -0.868374
H -2.759758 0.162378 -1.128808
H -3.466274 0.188188 0.607882
C -3.594113 2.746686 -1.790540
H -4.607903 2.501018 -2.108502
H -3.441301 3.828538 -1.866027
H -2.900464 2.235437 -2.455980
C -4.551026 2.723229 0.438432
H -5.475544 2.469642 -0.080714
H -4.537735 2.193744 1.392155
H -4.526535 3.804046 0.617708
I -3.784944 -2.381610 -0.507967
Br 1.789138 1.972630 -1.121532
Br 5.073267 -1.132561 -1.242569

K3_TS_4CHO_DMSO3_OPT_C
Electronic energy = -1602.763666
Thermal correction to Gibbs free energy (25°C) = 0.095115
Thermal correction to Gibbs free energy (80°C) = 0.124022
qh-G(25°C) = -1602.288004
qh-G(80°C) = -1602.304990

Geometry:
C -0.050324 2.080843 1.183146
C -1.080122 1.611369 2.008581
C -2.402706 1.771844 1.634219
C -2.729174 2.406346 0.418736
C -1.698356 2.883391 -0.399569
C -0.369925 2.714916 -0.015958
H -0.830029 1.103491 2.935155
H -3.178620 1.382635 2.283128
H -1.910457 3.377558 -1.338200
H 0.423629 3.077195 -0.664671
N -4.095253 2.460442 0.010989
C -4.349051 3.118867 -1.275752
H -5.415830 3.045464 -1.490230
H -4.066586 4.177401 -1.247209
H -3.799040 2.610918 -2.069776
C -5.034560 2.942252 1.040330
H -6.050835 2.814700 0.664326
H -4.934221 2.367391 1.958875
H -4.856412 4.001675 1.254455

C	-4.610298	0.459882	-0.342765
H	-5.553691	0.773899	-0.766162
H	-3.733779	0.362173	-0.969396
H	-4.544980	0.263294	0.716956
C	1.363592	1.860552	1.540199
O	1.731510	1.300760	2.557550
H	2.104227	2.230709	0.804877
I	-5.189436	-2.038140	-0.771650
H	4.000161	0.540473	2.181301
C	4.657716	-0.070781	1.561667
H	5.647886	-0.163079	2.008865
H	4.743183	0.325928	0.549571
N	4.060915	-1.457172	1.486003
C	4.155275	-2.074363	2.847846
H	5.209191	-2.102554	3.118686
H	3.607013	-1.457038	3.558400
H	3.757272	-3.087450	2.814744
C	4.867832	-2.297754	0.529170
H	4.394155	-3.276169	0.454918
H	4.900798	-1.793031	-0.436341
H	5.874722	-2.389373	0.937476
C	2.654961	-1.354460	0.995261
C	1.590947	-1.805838	1.765315
C	2.451847	-0.755109	-0.251845
C	0.291978	-1.631963	1.285473
H	1.729975	-2.269942	2.731606
C	1.155540	-0.586088	-0.714511
H	3.287562	-0.408883	-0.853163
C	0.071471	-1.017299	0.057357
H	-0.548849	-1.963981	1.888192
H	0.972550	-0.103574	-1.669436
C	-1.318663	-0.772122	-0.396681
O	-1.592932	-0.197355	-1.431473
H	-2.119877	-1.138885	0.274599
O	5.143676	0.101972	-1.677344
S	6.475169	0.449790	-2.355613
C	6.901852	2.094876	-1.750410
H	7.908910	2.349336	-2.086846
H	6.179684	2.792215	-2.175608
H	6.838967	2.094374	-0.660011
C	7.728268	-0.477571	-1.446198
H	7.623048	-0.262977	-0.380181
H	7.554062	-1.535797	-1.644443
H	8.717366	-0.189970	-1.807664

K3_TS_4CHO_Ionpair_anilinium
Electronic energy = -1049.655596
Thermal correction to Gibbs free energy (25°C) = 0.079710

Thermal correction to Gibbs free energy (80°C) = 0.103751
qh-G(25°C) = -1049.253404
qh-G(80°C) = -1049.267751

Geometry:

C	1.083864	2.984514	-0.316292
C	0.239410	3.084436	-1.423012
C	-1.135437	2.922252	-1.285962
C	-1.693036	2.663591	-0.023806
C	-0.836414	2.552499	1.090739
C	0.531792	2.713417	0.942587
H	-1.757548	3.018692	-2.165412
H	-1.230649	2.354661	2.079270
H	1.184951	2.643187	1.808040
H	0.655369	3.296800	-2.404236
N	-3.089970	2.447581	0.129694
C	-3.289732	0.367702	0.077718
H	-4.353912	0.457099	0.243666
H	-2.902464	0.373167	-0.930800
H	-2.618100	0.302460	0.921640
C	-3.933250	2.918499	-0.981145
H	-4.973255	2.713508	-0.729475
H	-3.805211	3.993741	-1.144758
H	-3.694787	2.377924	-1.897383
C	-3.647994	2.835345	1.439289
H	-4.726926	2.686948	1.407449
H	-3.246912	2.205665	2.232796
H	-3.432058	3.885794	1.658814
C	5.159009	-1.028062	0.012023
C	4.666216	-2.266699	-0.387228
C	3.292253	-2.508905	-0.411905
C	2.425664	-1.492279	-0.029147
C	2.908534	-0.243340	0.373420
C	4.275093	-0.012742	0.391643
H	2.940970	-3.480657	-0.729252
H	2.238140	0.555080	0.673274
H	4.656749	0.956646	0.694235
N	0.946744	-1.699969	-0.030184
C	0.410250	-1.521087	1.367807
H	-0.658505	-1.740585	1.342036
H	0.931578	-2.216735	2.024297
H	0.577685	-0.494088	1.688368
C	0.551001	-3.071933	-0.496231
H	-0.537564	-3.118940	-0.461401
H	0.898610	-3.216035	-1.518078
H	0.977377	-3.814399	0.176214
C	0.290789	-0.701626	-0.950560
H	-0.775769	-0.931740	-0.973627
H	0.453501	0.305340	-0.566994

H 0.730048 -0.809094 -1.941839
I -3.483521 -2.223329 0.008928
H 5.349663 -3.056542 -0.684764
C 2.538888 3.164525 -0.483744
O 3.345214 3.022864 0.417427
H 2.874508 3.434887 -1.501451
C 6.625816 -0.797538 0.027819
O 7.136051 0.252212 0.359078
H 7.247298 -1.655667 -0.286523

K3_TS_4CHO_trimethylanilinium_iodide_CONF7_OPT
Electronic energy = -1049.658998
Thermal correction to Gibbs free energy (25°C) = 0.078977
Thermal correction to Gibbs free energy (80°C) = 0.102952
qh-G(25°C) = -1049.256030
qh-G(80°C) = -1049.270319

Geometry:
C -1.161360 2.330510 -0.188596
C -0.402550 1.953844 -1.304374
C 0.979795 1.999518 -1.256334
C 1.640076 2.424421 -0.085609
C 0.879177 2.810310 1.024303
C -0.511511 2.757466 0.968133
H -0.909053 1.607606 -2.200329
H 1.542015 1.684935 -2.127845
H 1.350859 3.145684 1.938180
H -1.092404 3.047032 1.840193
N 3.064285 2.363226 -0.031348
C 3.671536 2.812956 1.226986
H 4.749883 2.667864 1.154618
H 3.469070 3.873791 1.412829
H 3.295652 2.214000 2.058223
C 3.757241 2.958159 -1.189207
H 4.823141 2.743645 -1.098996
H 3.398345 2.527372 -2.121814
H 3.605348 4.042779 -1.208746
C 3.499537 0.314543 -0.085714
H 4.542279 0.525259 0.105441
H 2.811052 0.165079 0.735171
H 3.146672 0.271338 -1.105388
C -2.631781 2.223691 -0.203122
O -3.279882 1.835149 -1.158974
H -3.139615 2.509805 0.738194
I 3.973557 -2.242482 -0.138757
H -5.427609 1.152751 -0.330809
C -5.958478 0.428187 0.287729
H -7.029997 0.447601 0.092112

H	-5.772846	0.614075	1.343041
N	-5.448949	-0.945982	-0.074757
C	-5.879221	-1.223637	-1.485311
H	-6.965136	-1.155240	-1.511101
H	-5.442883	-0.472741	-2.142830
H	-5.567792	-2.227930	-1.766804
C	-6.087002	-1.978800	0.817302
H	-5.703982	-2.958103	0.532800
H	-5.840006	-1.759648	1.853637
H	-7.165400	-1.928204	0.670775
C	-3.965354	-0.984014	0.091874
C	-3.133441	-1.307537	-0.971920
C	-3.442372	-0.650495	1.344916
C	-1.751616	-1.271776	-0.782102
H	-3.516051	-1.564924	-1.949499
C	-2.067661	-0.608656	1.519238
H	-4.084694	-0.406423	2.183101
C	-1.218083	-0.912258	0.450808
H	-1.093616	-1.505216	-1.614356
H	-1.643650	-0.327019	2.477602
C	0.253268	-0.795498	0.604153
O	0.789473	-0.412426	1.624382
H	0.856625	-1.071696	-0.282704

K3_TS_Br1_DMSO3_FIX_OPT
Electronic energy = -6517.949126
Thermal correction to Gibbs free energy (25°C) = 0.093290
Thermal correction to Gibbs free energy (80°C) = 0.121356
qh-G(25°C) = -6517.512417
qh-G(80°C) = -6517.529036

Geometry:

C	-1.370584	-0.974987	1.378356
C	-0.376247	-0.399775	2.172014
C	0.922129	-0.889842	2.164270
C	1.261744	-1.985940	1.354438
C	0.273525	-2.576119	0.560602
C	-1.019069	-2.058050	0.588524
H	-0.614199	0.450241	2.803464
H	1.666744	-0.407750	2.787840
H	0.483867	-3.427678	-0.072244
N	2.614725	-2.453399	1.336181
C	2.877508	-3.591034	0.449227
H	3.948882	-3.795759	0.466141
H	2.340872	-4.489650	0.776340
H	2.588604	-3.344134	-0.574006
C	3.177373	-2.697146	2.677086
H	4.218427	-3.004620	2.565511

H	3.150462	-1.789593	3.278122
H	2.620166	-3.489412	3.189591
C	3.793777	-0.898616	0.515991
H	4.674075	-1.467269	0.776383
H	3.305757	-1.067890	-0.433208
H	3.356566	-0.216537	1.229509
I	5.249093	0.989448	-0.462532
H	-2.146915	2.392465	2.691453
C	-2.850725	2.530734	1.871357
H	-3.742251	3.052865	2.221968
H	-3.126960	1.585132	1.402494
N	-2.190611	3.391917	0.825599
C	-1.900471	4.737553	1.425153
H	-2.856183	5.202581	1.659967
H	-1.328195	4.614177	2.341640
H	-1.354493	5.338545	0.698698
C	-3.163550	3.599437	-0.309500
H	-2.647975	4.127775	-1.110667
H	-3.538135	2.631164	-0.638221
H	-3.988565	4.198122	0.074541
C	-0.947268	2.712591	0.342301
C	0.301257	3.085004	0.833694
C	-1.079039	1.682578	-0.586736
C	1.438852	2.435578	0.354517
H	0.422338	3.872624	1.564705
C	0.075666	1.060683	-1.046152
H	-2.046278	1.333453	-0.930370
C	1.338456	1.432157	-0.602625
H	2.418540	2.728500	0.718979
O	-3.830677	0.404227	-0.320820
S	-5.116441	-0.415896	-0.492874
C	-5.449053	-1.103967	1.141989
H	-6.436759	-1.568888	1.142725
H	-4.683720	-1.856695	1.336995
H	-5.391837	-0.296207	1.875437
C	-6.442710	0.808112	-0.519191
H	-6.336162	1.455985	0.353965
H	-6.328276	1.383603	-1.438380
H	-7.406784	0.296115	-0.516307
H	-2.380028	-0.579598	1.347208
H	2.230499	0.960882	-1.000292
Br	-2.342983	-2.889778	-0.490848
Br	-0.082839	-0.306397	-2.347654

K3_TS_H_DMSO3_OPT
Electronic energy = -1376.179911
Thermal correction to Gibbs free energy (25°C) = 0.086931
Thermal correction to Gibbs free energy (80°C) = 0.113124

qh-G(25°C) = -1375.719278
qh-G(80°C) = -1375.734756

Geometry:

C	1.620027	1.920515	0.635987
C	0.724469	1.480332	1.611674
C	-0.630925	1.773144	1.513036
C	-1.120278	2.527655	0.434349
C	-0.221859	2.959939	-0.548292
C	1.136495	2.653381	-0.442248
H	1.076485	0.901336	2.459797
H	-1.301997	1.411555	2.284820
H	-0.558306	3.546384	-1.393872
H	1.817160	2.996404	-1.215751
N	-2.524027	2.811867	0.360280
C	-2.941464	3.620374	-0.788402
H	-4.029706	3.700233	-0.773534
H	-2.511905	4.628920	-0.749989
H	-2.643385	3.134533	-1.718891
C	-3.083416	3.359823	1.608223
H	-4.154225	3.518658	1.469579
H	-2.945262	2.662332	2.432886
H	-2.607289	4.315213	1.857736
C	-3.553621	0.971239	0.061824
H	-4.471473	1.502802	0.263528
H	-3.151086	0.952374	-0.940000
H	-2.985177	0.540038	0.872288
I	-4.848321	-1.214715	-0.309798
H	2.465651	-1.217383	2.334228
C	3.219105	-1.407153	1.570393
H	4.150332	-1.742965	2.028655
H	3.400429	-0.527140	0.951986
N	2.714175	-2.514430	0.683024
C	2.611537	-3.763077	1.503875
H	3.610778	-3.998254	1.866313
H	1.947790	-3.586582	2.348480
H	2.242501	-4.573885	0.877163
C	3.722142	-2.761097	-0.410650
H	3.319640	-3.523046	-1.077234
H	3.907093	-1.824992	-0.936232
H	4.642609	-3.108325	0.059536
C	1.403245	-2.108110	0.084175
C	0.226445	-2.777295	0.407104
C	1.399494	-1.023569	-0.791704
C	-0.971342	-2.358364	-0.175223
H	0.207210	-3.614901	1.091113
C	0.198139	-0.624202	-1.370116
H	2.309743	-0.475182	-1.016431
C	-0.987227	-1.292736	-1.069971

H	-1.893986	-2.873204	0.072470
H	0.195295	0.222089	-2.049281
O	4.221365	0.439830	-0.949954
S	5.604386	1.094366	-1.060059
C	5.698850	2.229260	0.339177
H	6.709538	2.636774	0.403121
H	4.985102	3.032558	0.151625
H	5.435513	1.685789	1.249778
C	6.767474	-0.143714	-0.450555
H	6.427120	-0.496041	0.525877
H	6.775214	-0.961799	-1.171640
H	7.761480	0.302718	-0.383045
H	2.676021	1.676029	0.694304
H	-1.923746	-0.991742	-1.528599

K3_TS_H_Ionpair_anilinium
Electronic energy = -823.078087
Thermal correction to Gibbs free energy (25°C) = 0.071397
Thermal correction to Gibbs free energy (80°C) = 0.092729
qh-G(25°C) = -822.690646
qh-G(80°C) = -822.703515

Geometry:

C	-1.607479	3.172805	0.994830
C	-0.580505	2.879423	1.892759
C	0.715708	2.649612	1.440432
C	1.015422	2.710639	0.068971
C	-0.020316	2.997659	-0.831988
C	-1.315573	3.231235	-0.365288
H	-2.616634	3.351961	1.350336
H	1.485487	2.421606	2.168271
H	0.165799	3.063868	-1.896065
H	-2.096960	3.467247	-1.081722
N	2.341065	2.421045	-0.382387
C	2.541237	0.312629	-0.180484
H	3.550669	0.385282	-0.558494
H	2.351015	0.436607	0.875435
H	1.717622	0.222391	-0.873851
C	3.414937	2.990185	0.449468
H	4.373899	2.703150	0.016379
H	3.344392	4.083167	0.481390
H	3.373286	2.595860	1.463698
C	2.590875	2.666671	-1.806952
H	3.622444	2.389316	-2.025996
H	1.933545	2.048055	-2.420244
H	2.442768	3.722192	-2.064045
H	-6.220270	-2.017784	0.615472
C	-5.726348	-0.137691	-0.309922

C	-5.427211	-1.376374	0.247231
C	-4.104200	-1.814122	0.343688
C	-3.087303	-0.993063	-0.129364
C	-3.373183	0.249829	-0.693569
C	-4.694338	0.674797	-0.780219
H	-6.756327	0.194974	-0.378820
H	-3.906389	-2.781615	0.784722
H	-2.585199	0.896894	-1.062369
H	-4.911312	1.643648	-1.217090
N	-1.651808	-1.401395	-0.039009
C	-1.038902	-1.431035	-1.414857
H	-0.005487	-1.767350	-1.311995
H	-1.610850	-2.126760	-2.027630
H	-1.070538	-0.431539	-1.844346
C	-1.466649	-2.761522	0.566974
H	-0.395231	-2.963882	0.577835
H	-1.854957	-2.756729	1.584257
H	-1.980596	-3.499187	-0.047413
C	-0.906791	-0.411495	0.818620
H	0.119392	-0.769296	0.922821
H	-0.928006	0.565889	0.336938
H	-1.397319	-0.363454	1.790558
I	2.721498	-2.232778	0.074954
H	-0.784736	2.824100	2.957155

K4_TS_3Br_Ionpair_ionpair
Electronic energy = -5976.374717
Thermal correction to Gibbs free energy (25°C) = 0.084484
Thermal correction to Gibbs free energy (80°C) = 0.109219
qh-G(25°C) = -5976.015090
qh-G(80°C) = -5976.030132

Geometry:

C	0.232805	3.047157	1.442534
C	-0.288218	3.534400	0.250993
C	-1.534684	3.146748	-0.230824
C	-2.292115	2.211582	0.490670
C	-1.764665	1.683821	1.680727
C	-0.525143	2.110566	2.146134
H	1.199020	3.379150	1.805424
H	-1.893522	3.579953	-1.154530
H	-2.304202	0.935174	2.247880
H	-0.143917	1.703796	3.078402
N	-3.545491	1.755178	-0.006265
C	-3.074370	0.004083	-1.089010
H	-4.123295	-0.116110	-1.320648
H	-2.457816	0.619719	-1.728167
H	-2.676401	-0.432403	-0.178615

C -4.187222 2.628327 -0.998796
H -5.152027 2.195800 -1.262520
H -4.340802 3.637565 -0.600178
H -3.586028 2.682919 -1.907454
C -4.496667 1.299193 1.023243
H -5.425906 1.018485 0.526642
H -4.109579 0.417330 1.536148
H -4.699192 2.095870 1.747913
I -2.657734 -2.154029 2.403027
C 5.002619 0.157713 1.408246
C 5.022226 -0.736747 0.347101
C 3.857858 -1.125065 -0.313981
C 2.649194 -0.591500 0.117533
C 2.598926 0.307134 1.181105
C 3.776783 0.677485 1.819410
H 5.922266 0.443514 1.905246
H 3.929653 -1.824364 -1.134883
H 1.658744 0.724907 1.519335
H 3.737965 1.377379 2.646703
N 1.368444 -0.951483 -0.556759
C 0.386411 -1.507202 0.448123
H -0.510575 -1.810515 -0.093949
H 0.844925 -2.362486 0.943881
H 0.129020 -0.735963 1.172212
C 1.547076 -1.979420 -1.635434
H 0.559053 -2.185323 -2.047182
H 2.196411 -1.577292 -2.411553
H 1.965481 -2.885249 -1.198720
C 0.768784 0.279590 -1.185551
H -0.132171 -0.031637 -1.717622
H 0.520286 0.994849 -0.400699
H 1.496736 0.705680 -1.875161
I -2.482040 -2.096697 -2.458327
Br 6.673081 -1.459197 -0.230739
Br 0.725938 4.779902 -0.758951

K4_TS_4CHO_Ionpair_ionpair
Electronic energy = -1061.191556
Thermal correction to Gibbs free energy (25°C) = 0.085660
Thermal correction to Gibbs free energy (80°C) = 0.111128
qh-G(25°C) = -1060.792923
qh-G(80°C) = -1060.808175

Geometry:
C 1.583168 3.076909 -0.761689
C 0.863994 3.326287 -1.931139
C -0.515906 3.149289 -1.969593
C -1.202805 2.719507 -0.823741

C	-0.472923	2.452032	0.353159
C	0.899058	2.635511	0.380198
H	-1.040195	3.367868	-2.890087
H	-0.968804	2.087264	1.246189
H	1.454021	2.445519	1.295720
N	-2.604264	2.489390	-0.846290
C	-2.785755	0.406867	-1.037812
H	-3.860817	0.514369	-1.071152
H	-2.215590	0.475148	-1.952948
H	-2.292033	0.279746	-0.080071
C	-3.317465	3.017206	-2.017453
H	-4.375410	2.779115	-1.909067
H	-3.200537	4.103708	-2.096968
H	-2.953966	2.542448	-2.929861
C	-3.314353	2.800351	0.409854
H	-4.374170	2.592075	0.259608
H	-2.958884	2.165080	1.221715
H	-3.185737	3.855660	0.673795
I	-1.944815	-0.164424	2.983893
C	5.523036	-1.101030	0.142591
C	5.058125	-2.346175	-0.270317
C	3.704156	-2.542520	-0.544852
C	2.827908	-1.473894	-0.396357
C	3.283477	-0.219066	0.018912
C	4.630497	-0.033753	0.286514
H	3.374988	-3.521089	-0.864934
H	2.605003	0.618124	0.138243
H	4.989650	0.939895	0.602693
N	1.370022	-1.627435	-0.674696
C	0.572348	-1.315953	0.570644
H	-0.481851	-1.483007	0.343703
H	0.902897	-1.978899	1.369930
H	0.727834	-0.275043	0.849626
C	1.005512	-3.015017	-1.116243
H	-0.071760	-3.025510	-1.281651
H	1.528251	-3.243887	-2.043803
H	1.266584	-3.721818	-0.330224
C	0.953771	-0.677020	-1.768316
H	-0.098507	-0.870946	-1.984152
H	1.085019	0.349217	-1.424063
H	1.570199	-0.869836	-2.645786
I	-3.008444	-2.155118	-1.332016
H	5.748896	-3.176686	-0.383532
H	1.382363	3.670246	-2.822207
C	3.043693	3.276652	-0.743916
O	3.752551	3.008877	0.209843
H	3.482485	3.687442	-1.671575
C	6.968882	-0.920382	0.425483
O	7.456744	0.131104	0.784495

H 7.597624 -1.818836 0.287971

K4_TS_4CHO_Ionpair_ionpair_DMSO2_OPT
Electronic energy = -1614.298098
Thermal correction to Gibbs free energy (25°C) = 0.099704
Thermal correction to Gibbs free energy (80°C) = 0.129829
qh-G(25°C) = -1613.824508
qh-G(80°C) = -1613.842187

Geometry:

C	1.077838	1.930496	-2.395584
C	0.759350	0.879628	-3.257867
C	-0.549267	0.420698	-3.367474
C	-1.572440	1.016568	-2.611495
C	-1.246016	2.066734	-1.728620
C	0.060769	2.516823	-1.630541
H	-0.758005	-0.387413	-4.055837
H	-2.003602	2.528752	-1.108029
H	0.302910	3.336534	-0.959407
N	-2.906852	0.535034	-2.683738
C	-3.031322	-0.897407	-1.155411
H	-4.067532	-1.072755	-1.409771
H	-2.260150	-1.460705	-1.661260
H	-2.783871	-0.133789	-0.425288
C	-3.244312	-0.213564	-3.905148
H	-4.297886	-0.487632	-3.858327
H	-3.067396	0.393559	-4.799566
H	-2.661025	-1.132557	-3.967297
C	-3.952031	1.515678	-2.334059
H	-4.923043	1.041724	-2.477470
H	-3.868405	1.804485	-1.285143
H	-3.883344	2.401526	-2.974816
I	-3.273432	2.077996	1.700681
C	4.556132	1.225165	1.738165
C	4.473645	-0.099861	2.159584
C	3.277307	-0.808149	2.042118
C	2.170273	-0.171942	1.492568
C	2.241089	1.158453	1.065718
C	3.433716	1.855611	1.191432
H	3.244821	-1.840344	2.362056
H	1.381859	1.658420	0.631562
H	3.501942	2.883373	0.852020
N	0.873950	-0.887350	1.329811
C	-0.215728	-0.139328	2.054768
H	-1.139988	-0.709125	1.943720
H	0.066491	-0.053115	3.104273
H	-0.349286	0.844665	1.609929
C	0.909176	-2.290802	1.865871

H -0.084026 -2.711541 1.704958
H 1.661079 -2.851239 1.309534
H 1.128800 -2.256340 2.932631
C 0.528414 -0.972118 -0.136632
H -0.401755 -1.538408 -0.210862
H 0.390932 0.036233 -0.527584
H 1.345219 -1.493236 -0.639089
I -3.150075 -2.704143 0.708085
H 5.344902 -0.594804 2.579350
H 1.537691 0.417357 -3.859502
C 2.469644 2.408480 -2.306165
O 2.840867 3.287996 -1.550601
H 3.189957 1.909792 -2.981953
C 5.843017 1.953590 1.868861
O 6.000318 3.105645 1.521855
H 6.675277 1.375831 2.310354
S 4.523697 -2.363615 -0.626858
O 3.088682 -2.849505 -0.392952
C 5.144347 -3.345147 -2.007375
H 5.234714 -4.372780 -1.654480
H 4.429591 -3.284130 -2.830586
H 6.123638 -2.964825 -2.304545
C 4.351558 -0.793419 -1.501856
H 3.900053 -0.082965 -0.805080
H 5.336708 -0.435478 -1.808227
H 3.699883 -0.949419 -2.365177

K4_TS_Br3_Ionpair_ionpair_DMSO2_OPT
Electronic energy = -6529.485731
Thermal correction to Gibbs free energy (25°C) = 0.098242
Thermal correction to Gibbs free energy (80°C) = 0.127560
qh-G(25°C) = -6529.051157
qh-G(80°C) = -6529.068536

Geometry:
C 0.740045 -2.194944 2.205510
C 0.426668 -1.409247 3.311380
C -0.844881 -0.861437 3.473408
C -1.839682 -1.087222 2.511204
C -1.527815 -1.855731 1.377420
C -0.254949 -2.395080 1.252453
H -1.046256 -0.277320 4.361304
H -2.251677 -2.024903 0.587312
N -3.139980 -0.515928 2.628311
C -3.129107 1.150415 1.329397
H -4.144175 1.368000 1.630303
H -2.308191 1.544428 1.910985
H -2.951892 0.479263 0.495754

C	-3.458911	0.057140	3.941770
H	-4.472697	0.456038	3.903578
H	-3.401196	-0.699321	4.733070
H	-2.779736	0.879204	4.172806
C	-4.236222	-1.364155	2.124331
H	-5.177899	-0.842074	2.297296
H	-4.135282	-1.526565	1.050337
H	-4.253916	-2.326469	2.648169
I	-3.532399	-1.265231	-2.000796
C	4.632284	-0.627714	-1.658490
C	4.337600	0.585993	-2.269307
C	3.119418	1.229844	-2.045473
C	2.190239	0.640675	-1.196170
C	2.462277	-0.575886	-0.572276
C	3.685032	-1.190767	-0.808432
H	2.935598	2.181190	-2.524836
H	1.754492	-1.039267	0.103523
N	0.868572	1.273765	-0.927226
C	-0.237114	0.389087	-1.446947
H	-1.187177	0.890347	-1.253564
H	-0.092505	0.245268	-2.517835
H	-0.209613	-0.569672	-0.929129
C	0.732820	2.618508	-1.585877
H	-0.252543	3.000966	-1.319290
H	1.521219	3.265896	-1.200788
H	0.795494	2.490056	-2.665619
C	0.681670	1.474751	0.556421
H	-0.264173	2.003448	0.689281
H	0.637064	0.502942	1.050374
H	1.519387	2.074263	0.915669
I	-3.099498	3.206358	-0.219522
S	4.616617	3.253696	0.223251
O	3.106148	3.503964	0.297289
C	5.359280	4.461282	1.339306
H	5.206176	5.445679	0.896467
H	4.860602	4.395130	2.308532
H	6.427363	4.253961	1.429494
C	4.903129	1.784293	1.230195
H	4.427296	0.945340	0.719144
H	5.977121	1.606711	1.313917
H	4.448904	1.938220	2.211630
H	1.722040	-2.640795	2.090357
H	5.582132	-1.121119	-1.828182
Br	4.076219	-2.818907	0.071615
H	5.066286	1.047419	-2.926385
H	1.180488	-1.232100	4.071141
Br	0.126407	-3.465905	-0.272325

K4_TS_H_Ionpair_ionpair
Electronic energy = -834.612791
Thermal correction to Gibbs free energy (25°C) = 0.077590
Thermal correction to Gibbs free energy (80°C) = 0.100366
qh-G(25°C) = -834.229202
qh-G(80°C) = -834.242982

Geometry:

C	1.859729	3.430132	-0.678945
C	1.262746	3.466601	-1.936327
C	-0.075054	3.105134	-2.104075
C	-0.841596	2.690688	-1.004887
C	-0.229742	2.629971	0.258467
C	1.101396	3.006221	0.414106
H	2.896656	3.724752	-0.551688
H	-0.505543	3.167952	-3.095174
H	-0.777139	2.280344	1.126850
H	1.543587	2.966615	1.406338
N	-2.200891	2.283153	-1.154851
C	-2.135784	0.152309	-1.205767
H	-3.211276	0.149804	-1.313198
H	-1.517075	0.252741	-2.085554
H	-1.699738	0.171062	-0.212422
C	-2.832333	2.648879	-2.426970
H	-3.863107	2.293881	-2.412820
H	-2.828895	3.734945	-2.578933
H	-2.320368	2.165648	-3.260686
C	-3.071417	2.592168	-0.007723
H	-4.084704	2.270286	-0.251589
H	-2.749411	2.044087	0.878836
H	-3.074614	3.668480	0.200947
I	-1.622203	-0.053689	2.900934
C	6.141374	-0.016923	0.570800
C	5.899669	-1.317380	0.141130
C	4.607719	-1.729632	-0.193646
C	3.561255	-0.819892	-0.090511
C	3.790082	0.485965	0.340209
C	5.081330	0.884582	0.668275
H	7.147871	0.294663	0.828219
H	4.456728	-2.747654	-0.525432
H	2.978667	1.199875	0.417971
H	5.252745	1.902661	1.001020
N	2.160029	-1.201164	-0.441884
C	1.250529	-0.992159	0.744910
H	0.251597	-1.332938	0.467534
H	1.636380	-1.573592	1.581995
H	1.218014	0.067143	0.995332
C	2.040533	-2.636405	-0.860023
H	0.990226	-2.819853	-1.086349

H 2.648907 -2.803136 -1.747706
H 2.360699 -3.274438 -0.037584
C 1.668864 -0.346144 -1.580858
H 0.670795 -0.699057 -1.847069
H 1.629638 0.695397 -1.258740
H 2.354495 -0.462356 -2.419637
I -2.040843 -2.400587 -1.327180
H 1.832189 3.789347 -2.802003
H 6.714379 -2.028645 0.060744

K4_TS_H_Ionpair_ionpair_DMSO2_OPT_B
Electronic energy = -1387.720752
Thermal correction to Gibbs free energy (25°C) = 0.091440
Thermal correction to Gibbs free energy (80°C) = 0.118847
qh-G(25°C) = -1387.262032
qh-G(80°C) = -1387.278221

Geometry:
C -1.694020 0.419406 2.827417
C -1.438511 -0.950698 2.802981
C -0.130478 -1.430251 2.775148
C 0.955427 -0.539209 2.778746
C 0.692676 0.838289 2.802409
C -0.619666 1.305411 2.822764
H 0.027267 -2.501763 2.752950
H 1.497369 1.560073 2.785012
H -0.795170 2.377325 2.834597
N 2.294335 -1.029647 2.692305
C 2.612352 -1.431587 0.618709
H 3.617445 -1.744762 0.863210
H 1.804968 -2.147423 0.683567
H 2.407659 -0.384858 0.420838
C 2.529506 -2.316974 3.361754
H 3.587192 -2.565534 3.268917
H 2.261962 -2.264135 4.423565
H 1.957297 -3.113335 2.885710
C 3.343009 -0.052050 3.011037
H 4.310677 -0.546756 2.919405
H 3.312087 0.775664 2.299230
H 3.232354 0.332534 4.032378
I 2.387669 2.619991 -0.098621
C -4.172763 2.880092 -0.730564
C -4.465660 1.863013 -1.633894
C -3.491479 0.927825 -1.991769
C -2.221180 1.026293 -1.434766
C -1.909631 2.047210 -0.535239
C -2.892329 2.967933 -0.182345
H -3.751277 0.141416 -2.688239

H -0.912139 2.143113 -0.113245
H -2.649403 3.756910 0.521598
N -1.137146 0.069706 -1.801468
C -0.066348 0.806502 -2.560334
H 0.720845 0.091011 -2.805480
H -0.516143 1.220357 -3.462927
H 0.342547 1.593689 -1.927953
C -1.617672 -1.062993 -2.660205
H -0.753808 -1.699750 -2.851877
H -2.383504 -1.610064 -2.108431
H -1.991330 -0.665238 -3.602655
C -0.556038 -0.535895 -0.546912
H 0.189919 -1.267126 -0.862265
H -0.077285 0.245925 0.046262
H -1.376676 -1.009290 -0.006001
I 2.976133 -1.933298 -1.868626
H -5.457786 1.779672 -2.064670
H -2.257831 -1.663512 2.796019
S -4.838493 -1.505972 -0.115379
O -3.391511 -2.003584 -0.199042
C -5.738237 -2.787035 0.782289
H -5.800803 -3.657793 0.129247
H -5.182350 -3.028560 1.690909
H -6.740169 -2.423009 1.018037
C -4.838802 -0.251502 1.180761
H -4.142676 0.527327 0.860092
H -5.844089 0.163496 1.281150
H -4.505453 -0.704602 2.117038
H -2.710903 0.796051 2.855217
H -4.935624 3.599340 -0.452476

KBr_gen
Electronic energy = -3171.496472
Thermal correction to Gibbs free energy (25°C) = 0.026073
Thermal correction to Gibbs free energy (80°C) = 0.031511
qh-G(25°C) = -3171.518270
qh-G(80°C) = -3171.522928

Geometry:
Br 0.000000 0.000000 1.117628
K 0.000000 0.000000 -2.058788

KCl_gen
Electronic energy = -1060.176322
Thermal correction to Gibbs free energy (25°C) = 0.024811
Thermal correction to Gibbs free energy (80°C) = 0.030030
qh-G(25°C) = -1060.196843

qh-G(80°C) = -1060.201284

Geometry:

K	0.000000	0.000000	1.420058
Cl	0.000000	0.000000	-1.587124

KI
Electronic energy = -611.353462
Thermal correction to Gibbs free energy (25°C) = 0.026883
Thermal correction to Gibbs free energy (80°C) = 0.032462
qh-G(25°C) = -611.376076
qh-G(80°C) = -611.380874

Geometry:

K	0.000000	0.000000	-2.527259
I	0.000000	0.000000	0.905998

K_phenolate_2
Electronic energy = -906.716854
Thermal correction to Gibbs free energy (25°C) = 0.038698
Thermal correction to Gibbs free energy (80°C) = 0.048290
qh-G(25°C) = -906.654241
qh-G(80°C) = -906.661211

Geometry:

C	1.059743	-1.207344	-0.000161
C	0.297753	-0.000053	0.000061
C	1.059468	1.207254	0.000098
C	2.451007	1.200262	0.000023
C	3.170814	0.000168	-0.000114
C	2.451162	-1.200151	-0.000245
H	0.516356	-2.149744	-0.000242
H	0.516181	2.149703	0.000216
H	2.985058	2.148288	0.000094
H	4.256002	0.000172	-0.000151
H	2.985548	-2.147989	-0.000378
O	-0.997838	-0.000279	0.000074
K	-3.485059	0.000052	0.000099

Methyliodide
Electronic energy = -51.222469
Thermal correction to Gibbs free energy (25°C) = 0.025795
Thermal correction to Gibbs free energy (80°C) = 0.031396
qh-G(25°C) = -51.207137
qh-G(80°C) = -51.211787

Geometry:
C 0.000000 0.000000 -1.834578
H 0.000000 1.036890 -2.158542
H 0.897973 -0.518445 -2.158542
H -0.897973 -0.518445 -2.158542
I 0.000000 0.000000 0.329870

PhOMe_GEN
Electronic energy = -346.636734
Thermal correction to Gibbs free energy (25°C) = 0.035433
Thermal correction to Gibbs free energy (80°C) = 0.044637
qh-G(25°C) = -346.530039
qh-G(80°C) = -346.536568

Geometry:
C -0.500552 -1.305147 0.000009
C 0.454048 -0.279625 -0.000025
C 0.045623 1.056545 -0.000031
C -1.320984 1.353195 0.000001
C -2.275145 0.340885 0.000012
C -1.854016 -0.992910 0.000022
H -0.158272 -2.335291 0.000025
H 0.767664 1.864281 -0.000063
H -1.632001 2.393567 -0.000011
H -3.332791 0.582362 0.000009
H -2.585296 -1.795618 0.000049
O 1.755538 -0.679168 -0.000076
C 2.755002 0.328377 0.000064
H 2.681210 0.955518 0.895001
H 3.709935 -0.194997 0.000117
H 2.681386 0.955600 -0.894834

PhO_MeBr_TS
Electronic energy = -2918.303531
Thermal correction to Gibbs free energy (25°C) = 0.043258
Thermal correction to Gibbs free energy (80°C) = 0.054183
qh-G(25°C) = -2918.201660
qh-G(80°C) = -2918.209404

Geometry:
O -1.399968 2.047368 -0.000021
C -0.005419 2.315412 -0.000150
H 0.093356 3.400046 -0.000284
H 0.475858 1.897521 -0.890883
H 0.476004 1.897631 0.890567
C -1.783675 0.740986 -0.000001
C -3.165681 0.508820 0.000235

C -0.888904 -0.332760 -0.000216
C -3.649424 -0.793663 0.000174
H -3.838410 1.360719 0.000416
C -1.395577 -1.636394 -0.000215
H 0.185797 -0.177275 -0.000329
C -2.766354 -1.878690 -0.000061
H -4.721801 -0.963694 0.000324
H -0.696082 -2.467604 -0.000374
H -3.145740 -2.895094 -0.000107
Br 2.980028 -0.342099 0.000064

PhO_MeCl_TS
Electronic energy = -806.980241
Thermal correction to Gibbs free energy (25°C) = 0.042533
Thermal correction to Gibbs free energy (80°C) = 0.053353
qh-G(25°C) = -806.877776
qh-G(80°C) = -806.885399

Geometry:
O 0.012224 -1.445902 -0.000032
C -1.305193 -0.912244 -0.000175
H -1.979796 -1.766724 -0.000193
H -1.486064 -0.305819 -0.893381
H -1.486266 -0.305751 0.892958
C 1.049601 -0.566876 -0.000031
C 2.332145 -1.132814 0.000082
C 0.899708 0.823038 -0.000116
C 3.453359 -0.312969 0.000130
H 2.423548 -2.214375 0.000140
C 2.039242 1.633794 -0.000069
H -0.082170 1.280745 -0.000186
C 3.315495 1.079263 0.000053
H 4.441631 -0.762530 0.000225
H 1.915153 2.712441 -0.000132
H 4.192373 1.717866 0.000090
Cl -4.631903 0.443776 0.000088

PhO_MeI_TS
Electronic energy = -358.086708
Thermal correction to Gibbs free energy (25°C) = 0.043355
Thermal correction to Gibbs free energy (80°C) = 0.054458
qh-G(25°C) = -357.989090
qh-G(80°C) = -357.996959

Geometry:
O 1.403522 -1.459571 0.791427
I -2.830356 0.166930 -0.147545

C -0.515171 -0.674005 0.388909
H -0.665369 -1.625029 -0.092377
H -0.014576 0.120650 -0.142847
H -0.639220 -0.594939 1.455899
C 2.372865 -0.674370 0.400674
C 3.381994 -1.128744 -0.491962
C 2.476861 0.675780 0.835262
C 4.421922 -0.299220 -0.900221
H 3.322003 -2.154860 -0.846828
C 3.520382 1.497685 0.416327
H 1.722570 1.054654 1.521667
C 4.506704 1.024014 -0.453774
H 5.176131 -0.688362 -1.580533
H 3.566752 2.522582 0.777604
H 5.319030 1.667756 -0.775428

Phenolate_GEN
Electronic energy = -306.876071
Thermal correction to Gibbs free energy (25°C) = 0.031899
Thermal correction to Gibbs free energy (80°C) = 0.039889
qh-G(25°C) = -306.809811
qh-G(80°C) = -306.815680

Geometry:
C -1.822749 0.000000 0.000075
C -1.100507 -1.199840 -0.000014
C 0.290338 -1.207941 0.000060
C 1.061924 0.000007 0.000544
C 0.290339 1.207944 0.000060
C -1.100514 1.199833 -0.000029
H -2.907996 -0.000007 -0.000004
H -1.634697 -2.148296 -0.000168
H 0.831241 -2.152239 -0.000173
H 0.831220 2.152254 -0.000170
H -1.634698 2.148292 -0.000184
O 2.350244 -0.000003 -0.000435

TMAI_dimer_Tshape
Electronic energy = -834.662505
Thermal correction to Gibbs free energy (25°C) = 0.077613
Thermal correction to Gibbs free energy (80°C) = 0.100261
qh-G(25°C) = -834.274874
qh-G(80°C) = -834.288575

Geometry:
C 2.093560 3.709026 -0.663054
C 1.501514 3.899946 -1.906965

C	0.190333	3.478948	-2.143933
C	-0.515119	2.855664	-1.120803
C	0.068735	2.645692	0.128762
C	1.371317	3.081432	0.353429
H	3.110209	4.043306	-0.484154
H	-0.241811	3.652789	-3.119993
H	-0.468970	2.143694	0.930206
H	1.819975	2.923597	1.329740
N	-1.918830	2.382975	-1.319001
C	-1.956222	0.876201	-1.222540
H	-2.984309	0.548666	-1.379122
H	-1.308007	0.468006	-1.997273
H	-1.622404	0.573100	-0.228831
C	-2.479302	2.775855	-2.651849
H	-3.504149	2.411562	-2.693686
H	-2.465859	3.861301	-2.742406
H	-1.893926	2.308007	-3.441513
C	-2.810132	2.966326	-0.252401
H	-3.832388	2.655226	-0.465889
H	-2.506119	2.579096	0.719656
H	-2.721076	4.051572	-0.288699
I	-1.743340	0.317473	2.808496
C	5.968054	-0.188227	0.585744
C	5.689317	-1.550594	0.565341
C	4.402609	-2.011533	0.276259
C	3.398871	-1.087778	0.008095
C	3.666248	0.280724	0.024072
C	4.950743	0.727044	0.313796
H	6.969861	0.160770	0.811444
H	4.220902	-3.077524	0.268902
H	2.889924	1.008321	-0.183986
H	5.150974	1.793222	0.325169
N	2.004386	-1.521100	-0.302702
C	1.052884	-0.988957	0.741948
H	0.046721	-1.324268	0.479857
H	1.353650	-1.380708	1.713243
H	1.097133	0.099223	0.746179
C	1.852052	-3.013337	-0.334311
H	0.806980	-3.221811	-0.567777
H	2.501106	-3.421848	-1.107878
H	2.105129	-3.418690	0.644291
C	1.588568	-0.994150	-1.651389
H	0.585912	-1.376257	-1.855700
H	1.581188	0.095507	-1.624496
H	2.303460	-1.349811	-2.392805
I	-2.073977	-2.765107	-1.231355
H	2.050058	4.384777	-2.706969
H	6.470561	-2.273015	0.774725

TMAI_dimer_anti
Electronic energy = -834.666927
Thermal correction to Gibbs free energy (25°C) = 0.077734
Thermal correction to Gibbs free energy (80°C) = 0.100377
qh-G(25°C) = -834.279243
qh-G(80°C) = -834.292948

Geometry:

C	-3.953764	-1.223542	-0.122857
C	-4.933628	-2.207755	-0.195925
C	-6.275917	-1.825091	-0.248699
C	-6.631345	-0.480543	-0.228466
C	-5.635517	0.493673	-0.154641
C	-4.294194	0.128341	-0.102016
H	-4.692836	-3.261830	-0.213530
H	-7.039021	-2.593632	-0.305971
H	-7.676112	-0.191741	-0.269684
H	-5.896425	1.546685	-0.137781
N	-2.502206	-1.576221	-0.064352
C	-1.892883	-1.036296	1.204378
H	-0.847392	-1.352817	1.224046
H	-2.442729	-1.451880	2.048438
H	-1.958787	0.052335	1.193915
C	-1.779340	-0.971595	-1.240415
H	-0.736127	-1.291100	-1.180970
H	-1.848215	0.115094	-1.177158
H	-2.247615	-1.338194	-2.153471
C	-2.264069	-3.056425	-0.091491
H	-1.185164	-3.208893	-0.042886
H	-2.659343	-3.464973	-1.020556
H	-2.746232	-3.510032	0.773294
H	-3.535854	0.904750	-0.044157
N	2.460431	1.522708	0.048146
C	3.851379	0.984443	-0.038864
C	4.350038	0.117553	0.926299
H	3.753034	-0.209155	1.766340
C	5.654872	-0.363084	0.798968
H	6.039731	-1.042350	1.551823
C	6.448655	0.016469	-0.277872
H	7.460923	-0.362076	-0.370354
C	5.934180	0.884449	-1.241260
H	6.540395	1.187245	-2.087956
C	4.636402	1.371602	-1.125443
H	4.256827	2.043195	-1.887553
C	1.666105	1.067955	-1.149733
H	0.655478	1.468582	-1.043989
H	1.664399	-0.023624	-1.148986
H	2.132696	1.454487	-2.053803

C	2.490699	3.027302	0.084921
H	1.459522	3.374964	0.170570
H	2.938334	3.401356	-0.833051
H	3.081727	3.334444	0.947233
C	1.731237	1.054974	1.274827
H	0.736017	1.501553	1.239900
H	2.267908	1.397947	2.158887
H	1.661390	-0.033566	1.245458
I	-1.624353	3.070361	0.155306
I	1.747719	-2.805582	0.096300

TMAI_dimer_stacked
Electronic energy = -834.664922
Thermal correction to Gibbs free energy (25°C) = 0.076926
Thermal correction to Gibbs free energy (80°C) = 0.099497
qh-G(25°C) = -834.276502
qh-G(80°C) = -834.290123

Geometry:

C	3.052689	1.840191	-1.346628
C	2.578083	1.681349	-2.645087
C	1.213978	1.800488	-2.925362
C	0.329350	2.070665	-1.885525
C	0.791119	2.237416	-0.579697
C	2.154179	2.128333	-0.318851
H	4.112027	1.738414	-1.132454
H	0.881040	1.671329	-3.946324
H	0.109937	2.421186	0.246404
H	2.508596	2.263024	0.698090
N	-1.145749	2.135993	-2.121660
C	-1.780612	0.882503	-1.566826
H	-2.857562	0.942766	-1.730959
H	-1.366719	0.023871	-2.094214
H	-1.569711	0.824773	-0.496706
C	-1.489666	2.233768	-3.577912
H	-2.570805	2.335224	-3.651775
H	-0.999283	3.109860	-4.001170
H	-1.178222	1.323965	-4.087085
C	-1.738737	3.336170	-1.428575
H	-2.789752	3.394157	-1.708666
H	-1.659057	3.206796	-0.349974
H	-1.201866	4.223716	-1.761409
I	-1.724717	1.703604	2.439678
C	5.585486	-0.526996	0.969228
C	5.262336	-1.422873	-0.050034
C	3.945241	-1.830705	-0.236149
C	2.947276	-1.332074	0.601327
C	3.257327	-0.449759	1.630274

C	4.583429	-0.049564	1.807361
H	6.612568	-0.207579	1.108952
H	3.719776	-2.529108	-1.034130
H	2.500368	-0.054654	2.294875
H	4.820391	0.643701	2.607096
N	1.539252	-1.765116	0.355567
C	0.550021	-1.041550	1.225763
H	-0.445656	-1.384780	0.938345
H	0.741905	-1.282243	2.270746
H	0.636508	0.030518	1.053111
C	1.395045	-3.239299	0.621329
H	0.354324	-3.507575	0.427729
H	2.060379	-3.787452	-0.043237
H	1.660444	-3.424818	1.661495
C	1.161127	-1.485241	-1.074829
H	0.109989	-1.757357	-1.194533
H	1.324010	-0.423679	-1.263118
H	1.779968	-2.084272	-1.739097
I	-2.655113	-2.665017	-0.588070
H	3.262772	1.460962	-3.456634
H	6.034098	-1.808931	-0.706838

TMSO_GEN
Electronic energy = -592.787048
Thermal correction to Gibbs free energy (25°C) = 0.034734
Thermal correction to Gibbs free energy (80°C) = 0.043681
qh-G(25°C) = -592.692477
qh-G(80°C) = -592.698896

Geometry:

S	0.000143	0.000213	0.147507
O	0.001335	0.002036	1.615727
C	-1.117943	1.203319	-0.528518
H	-1.080138	1.157416	-1.617693
H	-2.111520	0.955000	-0.152771
H	-0.793432	2.176631	-0.158134
C	-0.485085	-1.569846	-0.526221
H	-0.469488	-1.513423	-1.615402
H	0.229257	-2.305492	-0.154223
H	-1.488464	-1.776644	-0.151562
C	1.601595	0.364498	-0.529381
H	2.282470	-0.400308	-0.153102
H	1.545305	0.347428	-1.618663
H	1.881640	1.351877	-0.159657

methylbromide
Electronic energy = -2611.366366

Thermal correction to Gibbs free energy (25°C) = 0.024862
Thermal correction to Gibbs free energy (80°C) = 0.030256
qh-G(25°C) = -2611.349619
qh-G(80°C) = -2611.354094

Geometry:

Br	0.000000	0.000000	0.421625
C	0.000000	0.000000	-1.531319
H	0.000000	1.036528	-1.856319
H	0.897659	-0.518264	-1.856319
H	-0.897659	-0.518264	-1.856319

methylchloride
Electronic energy = -500.050615
Thermal correction to Gibbs free energy (25°C) = 0.023566
Thermal correction to Gibbs free energy (80°C) = 0.028685
qh-G(25°C) = -500.032024
qh-G(80°C) = -500.036258

Geometry:

Cl	0.000000	0.000000	0.661068
C	0.000000	0.000000	-1.136197
H	0.000000	1.033697	-1.473658
H	0.895208	-0.516849	-1.473658
H	-0.895208	-0.516849	-1.473658

trimethylamine_GEN
Electronic energy = -174.390711
Thermal correction to Gibbs free energy (25°C) = 0.030404
Thermal correction to Gibbs free energy (80°C) = 0.037915
qh-G(25°C) = -174.293445
qh-G(80°C) = -174.299024

Geometry:

N	-0.000184	-0.000057	-0.398027
C	0.644240	1.220330	0.063857
H	1.670379	1.263181	-0.311542
H	0.100858	2.092037	-0.311183
H	0.675898	1.279822	1.168229
C	0.734983	-1.167991	0.063861
H	0.258670	-2.078372	-0.310516
H	1.761173	-1.133064	-0.312227
H	0.771706	-1.224479	1.168245
C	-1.379176	-0.052351	0.063922
H	-1.929006	0.815256	-0.311206
H	-1.862128	-0.958712	-0.311727
H	-1.446545	-0.055194	1.168278

1. P. J. Hay and W. R. Wadt, *The Journal of Chemical Physics*, 1985, **82**, 299-310.
2. Y. Zhao and D. G. Truhlar, *Theoretical Chemistry Accounts*, 2008, **120**, 215-241.
3. M. Walker, A. J. A. Harvey, A. Sen and C. E. H. Dessent, *The Journal of Physical Chemistry A*, 2013, **117**, 12590-12600.
4. P. L. J. R.-G. J. C. IFunes, *Journal*, 2017, DOI: 10.5281/zenodo.884527.
5. R. Paton, *Journal*.
6. J. T. W. Lai, F. W. Lau, D. Robb, P. Westh, G. Nielsen, C. Trandum, A. Hvidt and Y. Koga, *Journal of Solution Chemistry*, 1995, **24**, 89-102.
7. J. R. Pliego Jr and J. M. Riveros, *Phys. Chem. Chem. Phys.*, 2002, **4**, 1622-1627.
8. J. Li, T. Zhu, G. D. Hawkins, P. Winget, D. A. Liotard, C. J. Cramer and D. G. Truhlar, *Theoretical Chemistry Accounts*, 1999, **103**, 9-63.
