# Supplementary material for: Trialkylammonium salt degradation: implications for methylation and cross-coupling
Source: Chem Sci. 2021 Apr 13;12(20):6949–63. doi: 10.1039/d1sc00757b (PMC8153232; doi:10.1039/d1sc00757b)
Supplement: SC-012-D1SC00757B-s002 [file SC-012-D1SC00757B-s002.pdf]

Electronic Supplementary Material (ESI) for Chemical Science.
This journal is © The Royal Society of Chemistry 2021

Experimental Supporting Information

Trialkylammonium Salt Degradation: Implications for Methylation and Cross-coupling

Jack B. Washington,[a†] Michele Assante,[b†] Chunhui Yan,[a] David McKinney,[a] Vanessa Juba,[a] Andrew G. Leach,[c] Sharon E. Baillie,[d] and Marc Reid*[a]

a. WestCHEM Department of Pure and Applied Chemistry, University of Strathclyde, Thomas Graham Building, 295 Cathedral Street, Glasgow, UK.
b. School of Pharmacy and Biomolecular Sciences, Liverpool John Moores University, Byrom Street, Liverpool, UK.
c. Division of Pharmacy and Optometry, University of Manchester, Stopford Building, Oxford Road, Manchester M13 9PT
d. GlaxoSmithKline, Medicines Research Centre, Gunnels Wood Road, Stevenage, Hertfordshire, UK.

Contents

1. General Information 4

2. General Procedures 5

General Procedure A – Preparation of N,N,N-trimethylanilinium iodide salts from N,N-dimethylaniline with methyl iodide 5

General Procedure B – Preparation of N,N,N-trimethylanilinium iodide salts from aniline with methyl iodide 5

General Procedure C – Preparation of N,N,N-trimethylanilinium hexafluorophosphate salts from N,N,N-trimethylanilinium iodide with ammonium hexafluorophosphate 5

General Procedure D – Preparation of N,N,N-trimethylanilinium triflate salts from N,N-dimethyl aniline with methyl triflate 5

General Procedure E – Preparation of N,N,N-trimethylanilinium tetrakis(3,5-bis(trifluoromethyl)phenyl) borate salts from N,N,N-trimethylanilinium iodide with sodium tetrakis(3,5-bis(trifluoro)phenyl borate 5

3. Synthesis of N,N,N-trimethylanilinium salts 6

*Synthesis of N,N,N-trimethylpyridinaminium iodide, **2a*** 6

*Synthesis of 4-formyl-N,N,N-trimethyanilinium iodide, **3a*** 6

*Synthesis of 4-benzoyl-N,N,N-trimethylanilinium iodide, **4a*** 7

*Synthesis of 4-fluoro-N,N,N-trimethylanilinium iodide, **5a*** 7

*Synthesis of 4-chloro-N,N,N-trimethylanilinium iodide, **6a*** 8

*Synthesis of 4-bromo-N,N,N-trimethylanilinium iodide, **7a*** 8

*Synthesis of 4-methoxy-N,N,N-trimethylanilinium iodide, **8a*** 9

*Synthesis of 3-methoxy-N,N,N-trimethylanilinium iodide, **9a*** 9

*Synthesis of 3-chloro-N,N,N-trimethylanilinium iodide, **10a*** 10

*Synthesis of 3-bromo-N,N,N-trimethylanilinium iodide, **11a*** 10

*Synthesis of N,N,N,4-tetramethylanilinium iodide, **12a*** 11

*Synthesis of N,N,N,3-tetramethylanilinium iodide, **13a*** 11

*Synthesis of N,N,N,2-tetramethylanilinium iodide, **14a*** 12

Synthesis of 4-trifluoromethyl-N,N,N-trimethylanilinium iodide, **15a** 12

*Synthesis of N,N,N-trimethylanilinium hexafluorophosphate, **1d*** 13

*Synthesis of N,N,N-trimethylpyridinaminium hexafluorophosphate, **2d*** 13

*Synthesis of 4-formyl-N,N,N-trimethylanilinium hexafluorophosphate, **3d*** 14
*Synthesis of 4-benzoyl-N,N,N-trimethylanilinium hexafluorophosphate, **4d*** 14
*Synthesis of 4-benzoyl-N,N,N-trimethylanilinium hexafluorophosphate, **11d*** 15
*Synthesis of N,N,N,2-tetramethylanilinium hexafluorophosphate, **14d*** 15
*Synthesis of N,N,N-trimethylanilinium triflate, **1e*** 16
*Synthesis of N,N,N-trimethylpyridinaminium triflate, **2e*** 16
*Synthesis of 4-formyl-N,N,N-trimethylanilinium triflate, **3e*** 17
*Synthesis of 4-benzoyl-N,N,N-trimethylanilinium triflate, **4e*** 17
*Synthesis of N,N,N,2-tetramethylanilinium triflate, **14e*** 18
Synthesis of sodium tetrakis(3,5-bis(trifluoromethyl)phenyl)borate (NaBArF) 18
*Synthesis of N,N,N-trimethylanilinium tetrakis(3,5-bis-(trifluoromethyl)phenyl) borate, **1f*** 19
*Synthesis of N,N,N,-trimethylpyridinaminium tetrakis(3,5-bis-(trifluoromethyl)phenyl) borate, **2f*** 20
*Synthesis of 4-formyl-N,N,N-trimethylanilinium tetrakis(3,5-bis-(trifluoromethyl)phenyl) borate, **3f*** 20
*Synthesis of 4-benzoyl-N,N,N-trimethylanilinium tetrakis(3,5-bis-(trifluoromethyl)phenyl) borate, **4f*** 21
*Synthesis of 4-benzoyl-N,N,N,-trimethylanilinium tetrakis(3,5-bis-(trifluoromethyl)phenyl) borate, **4f*** 21
*Synthesis of N,N,N,2-tetramethylanilinium tetrakis(3,5-bis-(trifluoromethyl)phenyl) borate, 1**4f*** 22
4. Thermal Gravimetric Analysis (TGA) 23
5. Thermal Volumetric Analysis – Sub-ambient Distillation (TVA-SAD) 25
6. Degradation of *N,N,N*-Trimethylanilinium iodide salts in DMSO-d_6 27
7. Effect of Halide Counterion of Degradation 28
8. Effect of halide additive on the degradation of *N,N,N*-trimethylanilinium iodide in DMSO-d_6 29
9. Effect of water on the degradation of *N,N,N*-trimethylanilinium iodide 30
10. Effect of non-nucleophilic counterion on degradation of *N,N,N*-trimethylanilinium salts 31
11. NMR Degradation Monitoring of *N*,*N*,*N*-trimethylanilinium iodides 33
Determination of T1 Relaxation Times for N,N,N-trimethylanilinium Iodides in DMSO-d_6 36
12. NMR Degradation Monitoring of 4-formyl-*N*,*N*,*N*-trimethylanilinium BArF 38
13. Diffusion ordered spectroscopy (DOSY) to probe ion pairing of *N,N,N*-trimethylanilinium salts in DMSO-d_6 39
14. Probing Iodide displacement of trimethylamine 41
15. Effect of UV radiation on the degradation of 4-formyl-*N,N,N*-trimethylanilinium iodide 42
16. Effect of concentration on the degradation of 4-formyl-*N,N,N*-trimethylanilinium iodide 42
17. In-depth Microkinetic Analysis Using COPASI 46
18. Effect of Temperature on the Degradation of 4-formyl-*N,N,N*-trimethylanilinium iodide 53
19. Kinetic isotope Effect Studies 56
20. Methylation of 4-^{t}Bu-phenol using *N,N,N*-trimethylanilinium salts 59
21. Methylation of phenols in DMSO using 3-Br-*N,N,N*-trimethylanilinium iodide 63
General procedure F - Methylation of phenols in DMSO using 3-Br-N,N,N-trimethylanilinium iodide 63
*Synthesis of 4-^{t}Bu-anisole **17*** 63

*Synthesis of 4-I-anisole **21*** 64
*Synthesis of 4-F-anisole **22*** 64
*Synthesis of 4-trifluoromethoxy-anisole **23*** 64
*Synthesis of 2,6-diphenylanisole **24*** 65
*Synthesis of 2,6-diphenylanisole **25*** 65
*Synthesis of 1,2,3-trimethoxybenzene **26*** 66
Synthesis of 2,6-dimethylanisole **27** 66
Synthesis of 2-methylanisole **28** 67
*Synthesis of 2-methoxynaphthalene **29*** 67
*Synthesis of estrone 3-methyl ether **30*** 67
*Synthesis of 7-methoxycoumarin **31*** 68
*Synthesis of thioanisole **32*** 68
*Synthesis of methylbenzoate **33*** 69
22. Methylation of phenols in NMP using 3-Br-*N,N,N*-trimethylanilinium iodide 69
23. Thermal degradation of trimethylsulfoxonium iodide in DMSO-d_6 72
24. Mechanistic experiments on Speciation of Active Methylating Species 74
*Synthesis of trimethylsulfoxonium hexafluorophosphate, **35d*** 74
*Synthesis of trimethylsulfoxonium tetrakis(3,5-bis-(trifluoromethyl)phenyl) borate, **35f*** 75
*General procedure for methylation of **16** for mechanistic experiments* 75
25. Conductivity Measurements 76
Single Point Measurements 76
Kinetic Analysis 77
26. ^{1}H and ^{13}C NMR spectra of *N,N,N*-trimethylanilinium salts 80

1. General Information

All reagents and solvents were purchased from commercial suppliers (Sigma Aldrich, Thermo Fisher Scientific, Fluorochem, VWR, or Alfa Aesar) and used without further purification unless stated otherwise.

Thin layer chromatography was carried out using Merck TLC Silica gel 60 F_{254} plates. A Mineralight UVGL-25 lamp was used at 254 nm to visualise spots; when necessary, vanillin or potassium permanganate dip were

used to develop TLC plates.

Column chromatography was carried out using VWR silica gel (40-60 μm mesh).

Infrared (IR) spectra were recorded on a Perkin Elmer Spectrometer 1. All spectra were taken from neat samples and absorptions are listed in cm^{-1}.

^{1}H, ^{13}C, ^{19}F, ^{31}P, ^{11}B NMR spectra for structural analysis and single point degradation studies were recorded on a Bruker AV3-400 spectrometer at 400 MHz and 101 MHz, 376 MHz, 162 MHz and 128 MHz, respectively. All spectra were recorded at ambient temperature using standard pulse methods. Chemical shifts are recorded in ppm with respect to residual solvent peak, and multiplicities are denoted as: singlet (s), doublet (d), triplet (t), quartet (q), quintet (quin) or multiplet (m).

^{1}H NMR spectra for elevated temperature studies were recorded on a Bruker AV3-500HD spectrometer at 500 MHz. Chemical shifts are reported in ppm with respect to 1,2,4,5-tetramethylbenzene internal standard.

2. General Procedures

General Procedure A – Preparation of N,N,N-trimethylanilinium iodide salts from N,N-dimethylaniline with methyl iodide

The relevant aryl-substituted *N,N*-dimethylaniline (1 equiv.) was dissolved in acetonitrile to a concentration of 2 M in a sealed 25 mL round bottom flask equipped with a stirrer bar. Methyl iodide (3 equiv.) was added dropwise to the stirred solution. The reaction mixture was stirred at 60 °C for 16 h and a white precipitate formed. Upon cooling, the solid was collected *via* vacuum filtration, and washed with diethyl ether (3 x 10 mL). The solid residue was then dried under high vacuum to give a white powder.

General Procedure B – Preparation of N,N,N-trimethylanilinium iodide salts from aniline with methyl iodide

The relevant aryl-substituted aniline (1 equiv.) was dissolved in dichloromethane to a concentration of 1 M and potassium carbonate (4 equiv.) were added to a sealed 50 mL round bottom flask equipped with a stirrer bar. Methyl iodide (4 equiv.) was added dropwise to the stirred solution. The reaction mixture was stirred at room temperature for 16 h. The suspension was then separated *via* vacuum filtration and washed with diethyl ether (3 x 10 mL). The solid residue was then added to absolute ethanol (50 mL) and stirred at 50 °C for 10 min, vacuum filtration of the warm suspension was used to remove the solid. The filtrate was then transferred to a round bottom flask and the solvent was removed *via* rotary evaporation to give a white powder.

General Procedure C – Preparation of N,N,N-trimethylanilinium hexafluorophosphate salts from N,N,N-trimethylanilinium iodide with ammonium hexafluorophosphate

The relevant aryl-substituted *N,N,N*-trimethylanilinium iodide (1 equiv.) was dissolved in the minimum amount of deionised water to achieve dissolution. Potassium hexafluorophosphate (2 equiv.) was added to the solution causing a white precipitation to form. The solid was collected *via* vacuum filtration and washed with deionised water (3 x 10 mL), then dried in a vacuum oven overnight.

General Procedure D – Preparation of N,N,N-trimethylanilinium triflate salts from N,N-dimethyl aniline with methyl triflate

The relevant substituted *N,N*-dimethylaniline (1 equiv.) was dissolved in dichloromethane to a concentration of 1 M in a 50 mL round bottom flask equipped with a stirrer bar. Methyl triflate (1.5 equiv.) was added dropwise to the stirred solution. The reaction mixture was stirred at room temperature for 18 h. Diethyl ether (20 mL) was added to the resulting solution causing a precipitate to form. The solid was collected *via* vacuum filtration and washed with diethyl ether (3 x 10 mL) and dried under vacuum.

General Procedure E – Preparation of N,N,N-trimethylanilinium tetrakis(3,5-bis(trifluoromethyl)phenyl) borate salts from N,N,N-trimethylanilinium iodide with sodium tetrakis(3,5-bis(trifluoro)phenyl borate

The relevant aryl-substituted *N,N,N*-trimethylanilinium iodide (1 equiv.) and sodium tetrakis(3,5-bis(trifluoromethyl)phenyl borate (1 equiv.) were stirred in a 1:1 biphasic mixture

of deionised water and dichloromethane. The organic layer was then collected and dried over sodium sulfate. The solvent was removed *in vacuo* to give a white powder.

3. Synthesis of N,N,N-trimethylanilinium salts

*Synthesis of N,N,N-trimethylpyridinaminium iodide, **2a***

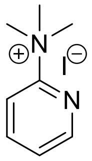

Prepared according to *General Procedure A*.

Amount of *N,N*-dimethylpyridinamine: 1.22 g, 10.0 mmol, 1 equiv.
Amount of methyl iodide: 1.87 mL, 4.26 g, 30.0 mmol, 3 equiv.
Volume of acetonitrile: 5.0 mL
Product yield: 2.46 g, 9.3 mmol, 93 %

Melting point: 203-205 °C

IR: ν_{max} 1490, 1466, 1151, 1060, 849 cm^{-1}

^{1}H NMR (400 MHz, DMSO-d$_6$): δ 3.60 (s, 9H, $N(CH_3)_3$), δ 7.73 (m, 1H, ArH), δ 8.11 (m, 1H, ArH), δ 8.25 (m, 1H, ArH), δ 8.68 (m, 1H, ArH)

^{13}C NMR (101 MHz, DMSO-d$_6$): δ 54.41, 115.28, 126.23, 141.07, 148.59, 156.67

*Synthesis of 4-formyl-N,N,N-trimethyanilinium iodide, **3a***

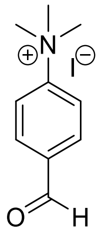

Prepared according to *General Procedure A*.

Amount of 4-formyl-*N,N*-dimethylanilinine: 2.98 g, 20.0 mmol, 1 equiv.
Amount of methyl iodide: 3.74 mL, 8.53 g, 60.1 mmol, 3 equiv.
Volume of acetonitrile: 10.0 mL
Product yield: 2.90 g, 10 mmol, 50 %

Melting point: 164-164 °C

IR: ν_{max} 3015, 1699, 1306, 1177, 827 cm^{-1}

^{1}H NMR (400 MHz, DMSO-d$_6$): δ 3.74 (s, 9H, $N(CH_3)_3$), δ 8.18 (m, 2H, ArH), δ 8.28 (m, 2H, ArH), δ 10.17 (s, 1H, CHO)

^{13}C NMR (101 MHz, DMSO-d$_6$): δ 56.40, 121.71, 130.84, 136.75, 151.01, 192.08

Synthesis of 4-benzoyl-N,N,N-trimethylanilinium iodide, ***4a***

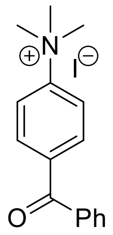

Prepared according to *General Procedure A*.

Amount of 4-dimethylaminobenzophenone: 2.25 g, 10.0 mmol, 1 equiv.
Amount of methyl iodide: 1.87 mL, 4.26 g, 30.0 mmol, 3 equiv.
Volume of acetonitrile: 5.0 mL
Product yield: 1.90 g, 5.2 mmol, 52 %

Melting point: 181-183 °C

IR: v_{max} 3007, 1655, 1597, 1288, 928 cm^{-1}

^{1}H NMR (400 MHz, DMSO-d_6): δ 3.70 (s, 9H, $N(CH_3)_3$), δ 7.61 (m, 2H, ArH), δ 7.72-7.79 (m, 3H, ArH), δ 7.95 (m, 2H, ArH), δ 8.19 (m, 2H, ArH)

^{13}C NMR (101 MHz, DMSO-d_6): δ 56.43, 121.14, 128.83, 129.78, 130.89, 133.46, 136.08, 138.47, 149.62, 194.47

Synthesis of 4-fluoro-N,N,N-trimethylanilinium iodide, ***5a***

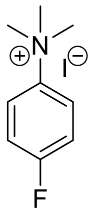

Prepared according to *General Procedure A*.

Amount of 4-fluoro-*N,N*-dimethylaniline: 1.39 g, 10.0 mmol, 1 equiv.
Amount of methyl iodide: 1.87 mL, 4.26 g, 30.0 mmol, 3 equiv.
Volume of acetonitrile: 5.0 mL
Product yield: 2.60 g, 9.2 mmol, 92%

Melting point: 203-205 °C

IR: v_{max} 3003, 1600, 1510, 1232, 1120, 948, 844 cm^{-1}

^{1}H NMR (400 MHz, DMSO-d_6): δ 3.66 (s, 9H, $N(CH_3)_3$), δ 7.54 (m, 2H, ArH), δ 8.09 (m, 2H, ArH)

^{13}C NMR (101 MHz, DMSO-d_6): δ 56.74, 116.65 (d, J_{CF}^2 = 23.6 Hz), 123.44 (d, J_{CF}^3 = 8.6 Hz), 143.32, 161.80 (d, J_{CF}^1 = 248 Hz)

^{19}F NMR (376 MHz, DMSO-d_6): δ -111.5 (m)

*Synthesis of 4-chloro-N,N,N-trimethylanilinium iodide, **6a***

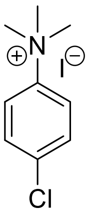

Prepared according to *General Procedure B*.

Amount of 4-chloro-*N,N*-dimethylaniline: 2.33 g, 15.0 mmol, 1 equiv.
Amount of methyl iodide: 3.74 mL, 8.52 g, 60.1 mmol, 4 equiv.
Amount of potassium carbonate: 8.29 g, 60.0 mmol, 4 equiv.
Volume of dichloromethane: 15.0 mL
Product yield: 1.61 g, 5.4 mmol, 36%

Melting point: 229-230 °C

IR: ν_{max} 1483, 1394, 1292, 1122, 1008, 945, 840 cm^{-1}

^{1}H NMR (400 MHz, DMSO-d_6): δ 3.66 (s, 9H, $N(CH_3)_3$), δ 7.75 (m, 2H, ArH), δ 8.05 (m, 2H, ArH)

^{13}C NMR (101 MHz, DMSO-d_6): δ 56.50, 122.83, 129.78, 134.62, 145.93

*Synthesis of 4-bromo-N,N,N-trimethylanilinium iodide, **7a***

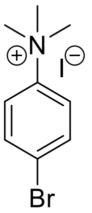

Prepared according to *General Procedure A*.

Amount of 4-bromo-*N,N*-dimethylaniline: 1.0 g, 5.0 mmol, 1 equiv.
Amount of methyl iodide: 0.94 mL, 2.14 g, 15.1 mmol, 3 equiv.
Volume of acetonitrile: 2.5 mL
Product yield: 1.27 g, 3.7 mmol, 74%

Melting point: 201-203 °C

IR: ν_{max} 2361, 1479, 1464, 1408, 1395, 1123, 1078, 1005, 945, 926, 839, 814, 735, 710 cm^{-1}

^{1}H NMR (400 MHz, DMSO-d_6): δ 3.65 (s, 9H, $N(CH_3)_3$), δ 7.88 (m, 2H, ArH), δ 7.98 (m, 2H, ArH)

^{13}C NMR (101 MHz, DMSO-d_6): δ 56.44, 123.01, 123.26, 132.75, 146.42

Synthesis of 4-methoxy-N,N,N-trimethylanilinium iodide, ***8a***

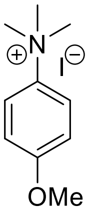

Prepared according to *General Procedure B*.

Amount of 4-methoxy-*N,N*-dimethylaniline: 0.63 g, 5.0 mmol, 1 equiv.
Amount of methyl iodide: 1.3 mL, 2.96 g, 20.0 mmol, 4 equiv.
Amount of potassium carbonate: 2.71 g, 19.9 mmol, 4 equiv.
Volume of dichloromethane: 5.0 mL
Product yield: 0.99 g, 3.4 mmol, 68%

Melting point: 236-238 °C

IR: ν_{max} 1605, 1512, 1261, 1188, 1026, 854 cm^{-1}

^{1}H NMR (400 MHz, DMSO-d_6): δ 3.64 (s, 9H, $N(CH_3)_3$), δ 3.83 (s, 3H, OCH_3), δ 7.12 (m, 2H, ArH), δ 7.93 (m, 2H, ArH)

^{13}C NMR (101 MHz, DMSO-d_6): δ 56.00, 56.67, 114.67, 121.98, 140.08, 159.53

Synthesis of 3-methoxy-N,N,N-trimethylanilinium iodide, ***9a***

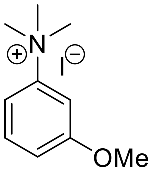

Prepared according to *General Procedure A*.

Amount of 3-methoxy-*N,N*-dimethylaniline: 3.02 g, 20.0 mmol, 1 equiv.
Amount of methyl iodide: 3.74 mL, 8.53 g, 60.1 mmol, 3 equiv.
Volume of acetonitrile: 10.0 mL
Product yield: 4.55 g, 15.5 mmol, 78%

Melting point: 184-185 °C

IR: ν_{max} 3003, 1610, 1469, 1255, 1126, 1026, 904, 840 cm^{-1}

^{1}H NMR (400 MHz, DMSO-d_6): δ 3.67 (s, 9H, $N(CH_3)_3$), δ 3.88 (s, 3H, OCH_3), δ 7.17 (m, 1H, ArH), δ 7.49-7.59 (m, 3H, ArH)

^{13}C NMR (101 MHz, DMSO-d_6): δ 56.26, 56.45, 107.17, 112.22, 115.35, 130.90, 148.30, 159.96

Synthesis of 3-chloro-N,N,N-trimethylanilinium iodide, ***10a***

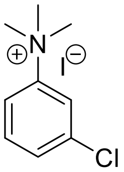

Prepared according to *General Procedure B*.

Amount of 3-chloro-*N,N*-dimethylaniline: 2.55 g, 20.0 mmol, 1 equiv.
Amount of methyl iodide: 5.0 mL, 11.4 g, 80.0 mmol, 4 equiv.
Amount of potassium carbonate: 11.1 g, 80.4 mmol, 4 equiv.
Volume of dichloromethane: 40.0 mL
Product yield: 1.95 g, 6.5 mmol, 33%

Melting point: 199-201 °C

IR: ν_{max} 3004, 1591, 1481, 1178, 1097, 1082, 947, 866, 792 cm^{-1}

^{1}H NMR (400 MHz, DMSO-d_6): δ 3.63 (s, 9H, $N(CH_3)_3$), δ 7.69 (m, 2H, ArH), δ 7.97 (m, 1H, ArH), δ 8.17 (m, 1H, ArH)

^{13}C NMR (101 MHz, DMSO-d_6): δ 56.46, 119.60, 121.24, 130.24, 131.62, 134.23, 148.18

Synthesis of 3-bromo-N,N,N-trimethylanilinium iodide, ***11a***

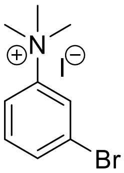

Prepared according to *General Procedure A*.

Amount of 3-bromo-*N,N*-dimethylaniline: 4.10 g, 20.5 mmol, 1 equiv.

Amount of methyl iodide: 3.8 mL, 8.73 g, 61.5 mmol, 3 equiv.

Volume of acetonitrile: 10.0 mL

Product yield: 6.59 g, 19.3 mmol, 94%

Melting point: 197-200 °C

IR: ν_{max} 3003, 2922, 2361, 1584, 1449, 1422, 1180, 1078, 995, 947, 856, 792 cm^{-1}

^{1}H NMR (400 MHz, DMSO-d_6): δ 3.66 (s, 9H, $N(CH_3)_3$), δ 7.63 (m, 1H, ArH), δ 7.83 (m, 1H, ArH), δ 8.04 (m, 1H, ArH), δ 8.23 (m, 1H, ArH)

^{13}C NMR (101 MHz, DMSO-d_6): δ 56.46, 119.93, 122.54, 123.82, 131.82, 133.17, 148.21

Synthesis of N,N,N,4-tetramethylanilinium iodide, ***12a***

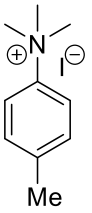

N,N,4-trimethylaniline (1.35, 10.0 mmol, 1 equiv.) was dissolved in dichloromethane to a concentration of 1 M in a 25 mL round bottom flask equipped with a stirrer bar. Methyl iodide (3 equiv.) was added dropwise to the stirred solution. The reaction mixture was stirred at room temperature for 16 h and a white precipitate formed. The precipitate was collected *via* vacuum filtration, and washed with diethyl ether (3 x 10 mL). The solid residue was then dried under high vacuum to give a white powder (2.13 g, 77%).

Melting point: 219-221 °C

IR: v_{max} 3009, 1512, 1394, 1122, 1018, 947, 813 cm^{-1}

^{1}H NMR (400 MHz, DMSO-d_6): δ 2.39 (s, 3H, Ar-CH_3), δ 3.62 (s, 9H, $N(CH_3)_3$), δ 7.45 (m, 2H, ArH, δ 7.78 (m, 2H, ArH)

^{13}C NMR (101 MHz, DMSO-d_6): δ 20.26, 56.40, 120.16, 130.23, 139.83, 144.93

Synthesis of N,N,N,3-tetramethylanilinium iodide, ***13a***

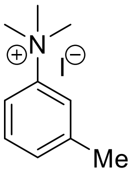

*N,N,*3-trimethylaniline (1.35, 10.0 mmol, 1 equiv.) was dissolved in dichloromethane to a concentration of 1 M in a 25 mL round bottom flask equipped with a stirrer bar. Methyl iodide (3 equiv.) was added dropwise to the stirred solution. The reaction mixture was stirred at room temperature for 16 h and a white precipitate formed. The precipitate was collected *via* vacuum filtration, and washed with diethyl ether (3 x 10 mL). The solid residue was then dried under high vacuum to give a white powder (2.30 g, 83%).

Melting point: 184-185 °C

IR: v_{max} 3009, 1614, 1487, 1296, 1182, 1095, 948, 896, 783 cm^{-1}

^{1}H NMR (400 MHz, DMSO-d_6): δ 2.44 (s, 3H, Ar-CH_3), δ 3.64 (s, 9H, $N(CH_3)_3$), δ 7.42 (m, 1H, ArH), δ 7.53 (m, 1H, ArH), δ 7.79 (m, 1H, ArH), δ 7.89 (m, 1H, ArH)

^{13}C NMR (101 MHz, DMSO-d_6): δ 21.04, 56.36, 117.36, 120.82, 129.74, 130.53, 139.95, 147.22

*Synthesis of N,N,N,2-tetramethylanilinium iodide, **14a***

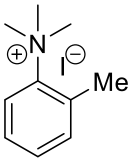

Prepared according to *General Procedure A*.

Amount of *N,N*,2-trimethylaniline: 2.70 g, 20.0 mmol, 1 equiv.
Amount of methyl iodide: 3.74 mL, 8.53 g, 60.1 mmol, 3 equiv.
Volume of acetonitrile: 10.0 mL
Product yield: 4.58 g, 16.5 mmol, 83%

Melting point: Decomposes >187 °C

IR: ν_{max} 1490, 1151, 1060, 948, 849, 756 cm^{-1}

^{1}H NMR (400 MHz, DMSO-d_6): δ 2.76 (s, 3H, Ar-CH_3), δ 3.73 (s, 9H, $N(CH_3)_3$), δ 7.43-7.55 (m, 3H, ArH), δ 7.88 (m, 1H, ArH)

^{13}C NMR (101 MHz, DMSO-d_6): δ 22.77, 56.47, 121.28, 127.85, 130.21, 130.30, 135.64, 146.16

*Synthesis of 4-trifluoromethyl-N,N,N-trimethylanilinium iodide, **15a***

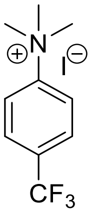

Prepared according to *General Procedure B*.

Amount of 4-trifluoromethyl-*N,N*-dimethylaniline: 1.74 g, 9.21 mmol, 1 equiv.
Amount of methyl iodide: 1.71 mL, 3.89 g, 27.6 mmol, 3 equiv.
Volume of acetonitrile: 5.0 mL
Product yield: 2.25 g, 6.8 mmol, 74%

Melting point: 190-192 °C

IR: ν_{max} 3015, 2359, 1616, 1329, 1163, 1126, 1070, 1013, 926, 839 cm^{-1}

^{1}H NMR (400 MHz, DMSO-d_6): δ 3.68 (s, 9H, N-$(CH_3)_3$), δ 8.08 (m, 2H, ArH), δ 8.25 (m, 2H, ArH)

^{13}C NMR (101 MHz, DMSO-d_6): δ 56.44, 122.14, 126.09 (q, J_{CF}^1 = 273.53 Hz), 127.23 (m), 130.66 (q, J_{CF}^2 = 30.08 Hz), 150.09

^{19}F NMR (376 MHz, DMSO-d_6): δ -61.36

Synthesis of N,N,N-trimethylanilinium hexafluorophosphate, ***1d***

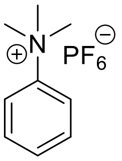

Prepared according to *General Procedure C*.

Amount of *N,N,N*-trimethylanilinium iodide: 0.25 g, 0.95 mmol, 1 equiv.
Amount of potassium hexafluorophosphate: 0.35 g, 1.9 mmol, 2 equiv.
Product yield: 0.20 g, 0.71 mmol, 73%

Melting point: 176-179 °C

IR: ν_{max} 2990, 1786, 1263, 838 cm^{-1}

^{1}H NMR (400 MHz, DMSO-d_6): δ 3.61 (s, 9H, $N(CH_3)_3$), δ 7.65 (m, 3H, ArH), δ 7.96 (m, 2H, ArH)

^{13}C NMR (101 MHz, DMSO-d_6): δ56.39, 120.32, 130.03, 131.10, 147.21

^{19}F NMR (376 MHz, DMSO-d_6): δ -70.15 (d, PF_6^-, J = 710 Hz)

^{31}P NMR (162 MHz, DMSO-d_6): δ -144.15 (hep, J = 711 Hz)

Synthesis of N,N,N-trimethylpyridinaminium hexafluorophosphate, ***2d***

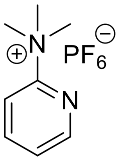

Prepared according to *General Procedure C*.

Amount of *N,N,N*-trimethylpyridinaminium iodide: 0.14 g, 0.54 mmol, 1 equiv.
Amount of potassium hexafluorophosphate: 0.20 g, 1.1 mmol, 2 equiv.
Product yield: 0.09 g, 0.33 mmol, 62%

Melting point: 204-206 °C

IR: ν^{max} 3008, 1738, 1252, 1162 cm^{-1}

^{1}H NMR (400 MHz, DMSO-d_6): δ3.59 (s, 9H, $N(CH_3)_3$), δ 7.73 (m, 1H, ArH), δ 8.09 (m, 1H, ArH), δ 8.25 (m, 1H, ArH), δ 8.68 (m, 1H, ArH)

^{13}C NMR (101 MHz, DMSO-d_6): δ 54.53, 115.11, 126.18, 141.03, 148.59, 156.67

^{19}F NMR (376 MHz, DMSO-d_6): δ -70.15 (d, PF_6^-, J = 710 Hz)

^{31}P NMR (162 MHz, DMSO-d_6): δ -144.17 (hep, J = 711 Hz)

Synthesis of 4-formyl-N,N,N-trimethylanilinium hexafluorophosphate, ***3d***

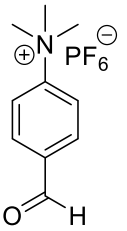

Prepared according to *General Procedure C*.

Amount of 4-formyl-*N,N,N*-trimethylanilinium iodide: 0.21 g, 0.73 mmol, 1 equiv.
Amount of potassium hexafluorophosphate: 0.28 g, 1.5 mmol, 2 equiv.
Product yield: 0.16 g, 0.51 mmol, 70%

Melting point: 184-186 °C

IR: ν_{max} 941, 1180, 1217, 1492, 1605, 1703 cm^{-1}

^{1}H NMR (400 MHz, DMSO-d_6): δ 3.65 (s, 9H, $N(CH_3)_3$), δ 8.16 (d, 2H, ArH, J = 9.1 Hz), δ 8.22 (d, 2H, ArH, J = 9.1 Hz), δ 10.12 (s, 1H, CHO)

^{13}C NMR (101 MHz, DMSO-d_6): δ 56.43, 121.64, 130.86, 136.80, 151.01, 192.02

^{19}F NMR (376 MHz, DMSO-d_6): δ -70.15 (d, PF_6^-, J = 710 Hz)

^{31}P NMR (162 MHz, DMSO-d_6): δ -146.34 (hep, J = 709 Hz)

Synthesis of 4-benzoyl-N,N,N-trimethylanilinium hexafluorophosphate, ***4d***

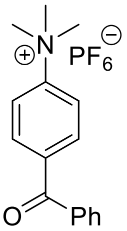

Prepared according to *General Procedure C*.

Amount of 4-benzoyl-*N,N,N*-trimethylanilinium iodide: 95.5 mg, 0.26 mmol, 1 equiv.
Amount of potassium hexafluorophosphate: 95.7 mg, 0.52 mmol, 2 equiv.
Product yield: 67.1 mg, 0.17 mmol, 67%

Melting point: 238-240 °C

IR: ν_{max} 2998, 1783, 1675, 1302, 832 cm^{-1}

^{1}H NMR (400 MHz, DMSO-d_6): δ 3.68 (s, 9H, $N(CH_3)_3$), δ 7.61 (m, 2H, ArH), δ 7.76 (m, 3H, ArH), δ 7.96 (m, 2H, ArH), δ 8.16 (m, 2H ArH)

^{13}C NMR (101 MHz, DMSO-d_6): δ 56.44, 120.99, 128.79, 129.74, 130.86, 133.41, 136.21, 138.56, 149.59, 194.42

^{19}F NMR (376 MHz, DMSO-d_6): δ -70.16 (d, PF_6^-, J = 711 Hz)

^{31}P NMR (162 MHz, DMSO-d_6): δ -144.16 (hep, J = 711 Hz)

Synthesis of 4-benzoyl-N,N,N-trimethylanilinium hexafluorophosphate, ***11d***

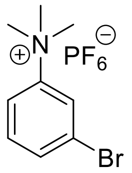

Prepared according to *General Procedure C*.

Amount of 3-bromo-*N,N,N*-trimethylanilinium iodide: 684.1 mg, 2.00 mmol, 1 equiv.
Amount of potassium hexafluorophosphate: 73.6 mg, 4.00 mmol, 2 equiv.
Product yield: 64.9 mg, 0.17 mmol, 95%

Melting point: 172-174 °C

IR: v_{max} 2359, 1587, 1468, 1435, 1082, 949, 829, 785, 681 cm^{-1}

^{1}H NMR (400 MHz, DMSO-d6): δ 3.63 (s, 9H, $N(CH_3)_3$), δ 7.61 (m, 1H, ArH), δ 7.82 (m, 1H, ArH), δ 8.01 (m, 1H, ArH), δ 8.26 (m, 1H, ArH)

^{13}C NMR (101 MHz, DMSO-d_6): δ 56.46, 119.93, 122.54, 123.82, 131.82, 133.17, 148.21

^{19}F NMR (376 MHz, DMSO-d_6): δ -70.15 (d, PF_6^-, J = 711 Hz)

^{31}P NMR (162 MHz, DMSO-d_6): δ -144.19 (hep, J = 711 Hz)f

Synthesis of N,N,N,2-tetramethylanilinium hexafluorophosphate, ***14d***

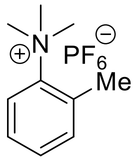

Prepared according to *General Procedure C*.

Amount of *N,N,N*,2-tetramethylaniliniumiodide: 0.33 g, 1.19 mmol, 1 equiv.
Amount of ammonium hexafluorophosphate: 0.39 g, 2.38 mmol, 2 equiv.
Product yield: 52.7 mg, 0.18 mmol, 15%

Melting point: Decomposes >190 °C

IR: v_{max} 1492, 1062, 943, 871 cm^{-1}

^{1}H NMR (400 MHz, DMSO-d_6): δ 2.73 (s, 3H, Ar-CH_3), δ 3.67 (s, 9H, $N(CH_3)_3$), δ 7.49 (m, 3H, ArH), δ 7.85 (d, 1H, J = 7.3 Hz)

^{13}C NMR (101 MHz, DMSO-d_6): δ 22.53, 56.52, 121.08, 127.82, 130.21, 130.36, 135.67, 145.22

^{19}F NMR (376 MHz, DMSO-d_6): δ -70.15 (d, PF_6^-, J = 710 Hz)

^{31}P NMR (162 MHz, DMSO-d_6): δ -144.15 (hep, J = 710 Hz)

*Synthesis of N,N,N-trimethylanilinium triflate, **1e***

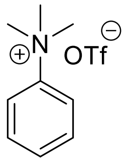

Prepared according to *General Procedure D*.

Amount of *N,N*-dimethylaniline: 2.42 g, 20.0 mmol, 1 equiv.
Amount of methyl triflate: 3.30 mL, 4.94 g, 30.0 mmol, 1.5 equiv.
Volume of dichloromethane: 20.0 mL
Product yield: 4.12 g, 14.4 mmol, 72%

Melting point: 82-84 °C

IR: ν_{max} 3554, 3503, 2359, 1641, 1499, 1254, 1223, 1169, 1153, 1024, 951, 847, 756, 689, 624 cm^{-1}

^{1}H NMR (400 MHz, DMSO-d_6): δ 3.61 (s, 3H, $N(CH_3)_3$), δ 7.56-7.68 (m, 3H, Ar-H), δ 7.97 (m, 2H, ArH)

^{13}C NMR (101 MHz, DMSO-d_6): δ 56.38, 119.07, 120.27, 122.27, 130.03, 147.19

^{19}F NMR (376 MHz, DMSO-d_6): δ -77.75

*Synthesis of N,N,N-trimethylpyridinaminium triflate, **2e***

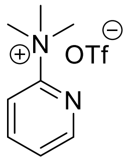

Prepared according to *General Procedure D*.

Amount of *N,N*-dimethylpyridinamine: 2.44 g, 20.0 mmol, 1 equiv.
Amount of methyl triflate: 3.71 mL, 5.55 g, 33.8 mmol, 1.7 equiv.
Volume of dichloromethane: 20.0 mL
Product yield: 5.04 g, 17.6 mmol, 88%

Melting point: 108-109 °C

IR: ν_{max} 3003, 1473, 1259, 1226, 1147, 945 cm^{-1}

^{1}H NMR (400 MHz, DMSO-d_6): δ 3.59 (s, 9H, $N(CH_3)_3$), δ 7.73 (m, 1H, ArH), δ 8.09 (m, 1H, ArH), δ 8.24 (m, 1H, ArH), δ 8.68 (m, 1H, ArH)

^{13}C NMR (101 MHz, DMSO-d_6): δ 54.50, 115.08, 119.08, 126.17, 141.01, 148.58, 156.68

Synthesis of 4-formyl-N,N,N-trimethylanilinium triflate, ***3e***

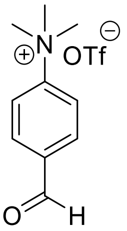

Prepared according to *General Procedure D*.

Amount of 4-formyl-*N,N*-dimethylaniline: 1.49 g, 10.0 mmol, 1 equiv.
Amount of methyl triflate: 1.7 mL, 2.54 g, 15.5 mmol, 1.5 equiv.
Volume of dichloromethane: 10.0 mL
Product yield: 2.13 g, 6.8 mmol, 68%

Melting point: 107-119 °C

IR: v_{max} 1259, 1147, 1026 cm^{-1}

^{1}H NMR (400 MHz, DMSO-d_6): δ 3.66 (s, 9H, $N(CH_3)_3$), δ 8.17 (d, 2H, ArH, J = 9.1 Hz), δ 8.22 (d, 2H, ArH, J = 9.1 Hz), δ 10.12 (s, 1H, CHO)

^{13}C NMR (101 MHz, DMSO-d_6): δ 56.43, 119.06, 121.66, 130.84, 136.80, 151.03, 192.03

^{19}F NMR (376 MHz, DMSO-d_6): δ -77.75

Synthesis of 4-benzoyl-N,N,N-trimethylanilinium triflate, ***4e***

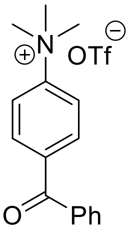

Prepared according to *General Procedure D*.

Amount of 4-benzoyl-*N,N*-dimethylaniline: 1.13 g, 5.0 mmol, 1 equiv.
Amount of methyl triflate: 0.85 mL, 1.27 g, 7.75 mmol, 1.55 equiv.
Volume of dichloromethane: 5 mL
Product yield: 1.57 g, 3.0 mmol, 59%

Melting point: 128-130 °C

IR: v_{max} 1259, 1226, 1139, 1033 cm^{-1}

^{1}H NMR (400 MHz, DMSO-d_6): δ 3.68 (s, 9H, $N(CH_3)_3$), δ 7.62 (m, 2H, ArH), δ 7.76 (m, 3H, ArH), δ 7.96 (m, 2H, ArH), δ 8.17 (m, 2H, ArH)

^{13}C NMR (101 MHz, DMSO-d_6): δ 56.44, 119.08, 120.99, 128.77, 129.74, 130.86, 133.40, 136.12, 138.54, 149.60, 194.42

^{19}F NMR (376 MHz, DMSO-d_6): δ -77.74

Synthesis of N,N,N,2-tetramethylanilinium triflate, ***14e***

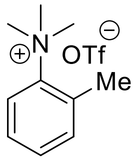

2-methylaniline (2.14 g, 20 mmol, 1 equiv.) dissolved in dichloromethane (10 mL) and potassium carbonate (11.0 g, 80.0 mmol, 4 equiv.) were added to a 50 mL round bottom flask equipped with a stirrer bar. Methyl triflate (9.0 mL, 13.5 g, 80.0, 4 equiv.) was added dropwise to the stirred solution. The reaction mixture was stirred at room temperature for 18 h. The suspension was separated *via* vacuum filtration. Diethyl ether (20 mL) was added to the filtrate causing a precipitate to form. The solid was collected *via* vacuum filtration and washed with diethyl ether (3 x 10 mL) and dried under vacuum to give a white crystalline solid (1.62 g, 5.4 mmol, 27%).

Melting point: 88-90 °C

IR: ν_{max} 2361, 1254, 1234, 1167, 1034, 762, 637, 559 cm^{-1}

^{1}H NMR (400 MHz, DMSO-d$_6$): δ 2.73 (s, 3H, Ar-CH_3), δ 3.67 (s, 9H, $N(CH_3)_3$), δ 7.49 (m, 3H, ArH), δ 7.85 (m, 1H, ArH)

^{13}C NMR (101 MHz, DMSO-d$_6$): δ 22.52, 56.52, 119.06, 121.08, 122.26, 127.81, 130.18, 130.34, 135.65

^{19}F NMR (376 MHz, DMSO-d$_6$): δ -77.75

Synthesis of sodium tetrakis(3,5-bis(trifluoromethyl)phenyl)borate (NaBArF)

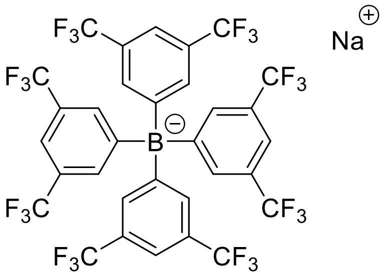

Magnesium turnings (5.00 g, 206 mmol) was added to a flame dried 3-neck round bottom flask (250 mL) and ground under vacuum with a stir bar for 12 h. Iodine (0.500 g, 3.94 mmol) was added under argon, the system was then returned to vacuum and heated with a heat gun. Upon formation of iodine vapour, the mixture was allowed to cool to room temperature. Dry diethyl ether (25 mL) was added and the mixture was stirred vigorously under argon for 25 mins giving a colourless liquid. 1,3-bis(trifluoromethyl)-5-bromobenzene (14 mL, 81 mmol) in dry diethyl ether (25 mL) was added dropwise *via* a dropping funnel over the course of 65 mins, resulting in a dark brown reaction mixture. The contents of the flask were stirred under argon at room temperature for a further 4 h. Boron trifluoride diethyl etherate (2 mL, 16.3 mmol) in dry diethyl ether (12 mL) was added to the Grignard reagent over the course of 1 h, once the addition was complete, the reaction mixture was refluxed under argon for 16 h. The reaction was then cooled to room temperature and transferred *via* cannula into an air-exposed aqueous sodium carbonate solution (1.5 M, 250 mL). The resulting suspension was stirred at room temperature for 1 h. The brown solid was collected *via* vacuum filtration, then extracted

with diethyl ether (3 x 40 mL). The organic extracts were dried over sodium sulfate and the solvent was removed *via* rotary evaporation, giving the crude $NaBAr^F$ salt as a brown oil.

The crude oil was dissolved in a 1:1 mixture of tetrahydrofuran and dichloromethane (15 mL), the solution was placed in a freezer for 48 h and a pale brown solid was filtered. A second recrystallization of this solid from tetrahydrofuran and dichloromethane (15 mL) was performed yielding an off-white solid. The solid was dried under vacuum at 80 °C for 50 h to give $NaBAr^F$ as a white solid (8.375 g, 9.45 mmol, 58%)

Melting point: Decomposed at 330°C

IR: ν_{max} 2360, 1628, 1356, 1281, 1167, 1063, 931, 887, 837 710, 681, 670 cm^{-1}

^{1}H NMR (400 MHz, DMSO-d_6): δ 7.63 (s, 8H, ArH), δ 7.69 (s, 4H, ArH)

^{13}C NMR (101 MHz, DMSO-d_6): δ 117.59 (m), 123.95 (q, $J_{CF}{}^1$ = 273 Hz), 128.60 (q, $J_{CF}{}^2$ = 32 Hz), 134.00, 160.90 (q, $J_{CB}{}^1$ = 50.5 Hz)

^{19}F NMR (376 MHz, DMSO-d_6): δ -61.75

^{11}B NMR (128 MHz, DMSO-d_6): δ -6.83

Synthesis of N,N,N-trimethylanilinium tetrakis(3,5-bis-(trifluoromethyl)phenyl) borate, ***1f***

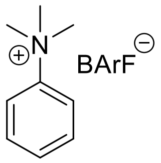

Prepared according to *General Procedure E*.

Amount of *N,N,N*-trimethylanilinium iodide: 0.263 g, 1.00 mmol, 1 equiv.
Amount of tetrakis(3,5-bis(trifluoromethyl)phenyl) borate: 0.886 g, 1 mmol, 1 equiv.
Product yield: 0.849 g, 0.85 mmol, 85%

Melting point: 150-152 °C

IR: ν_{max} 1610, 1491, 1334, 1277, 1161, 1112 cm^{-1}

^{1}H NMR (400 MHz, DMSO-d_6): δ 3.61 (s, 9H, $N(CH_3)_3$), δ 7.63 (m, 11H, ArH anilinium and ArH BArF), δ 7.73 (s, 4H, ArH BArF), δ 7.97 (m, 2H, ArH anilinium)

^{13}C NMR (101 MHz, DMSO-d_6): δ 56.34, 117.45, 120.33, 123.93 (q, $J_{CF}{}^1$ = 274 Hz), 128.43 ($J_{CF}{}^2$ = 31 Hz), 129.94, 134.00, 147.21, 160.93 (q, $J_{CB}{}^1$ = 51 Hz)

^{19}F NMR (376 MHz, DMSO-d_6): δ -61.60

^{11}B NMR (128 MHz, DMSO-d_6): δ -6.83

Synthesis of N,N,N,-trimethylpyridinaminium tetrakis(3,5-bis-(trifluoromethyl)phenyl) borate, ***2f***

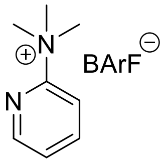

Prepared according to *General Procedure E*.

Amount of *N,N,N*-pyridinaminium iodide: 0.264 g, 1.00 mmol, 1 equiv.
Amount of tetrakis(3,5-bis(trifluoromethyl)phenyl) borate: 0.886 g, 1 mmol, 1 equiv.
Product yield: 0.911 g, 0.91 mmol, 91%

Melting point: 156-158 °C

IR: ν_{max} 1610, 1490, 1472, 1437, 1354, 1275, 1161, 1112 cm^{-1}

^{1}H NMR (400 MHz, DMSO-d$_6$): δ 3.59 (s, 9H, $N(CH_3)_3$), δ 7.62 (m, 8H, ArH BArF), δ 7.70 (m, 5H, ArH BArF and ArH anilinium), δ 8.09 (m, 1H, ArH anilinium), δ 8.25 (m, 1H, ArH anilinium), δ 8.68 (m, 1H, ArH anilinium)

^{13}C NMR (101 MHz, DMSO-d$_6$): δ 54.47, 115.14, 117.50 (m), 119.87, 123.94 (q, $J_{CF}{}^1$ = 274 Hz), 126.12, 128.43 (q, $J_{CF}{}^2$ = Hz 32 Hz), 134.00, 140.98, 148.53, 160.92 (q, $J_{CB}{}^1$ = 50.5 Hz)

^{19}F NMR (376 MHz, DMSO-d$_6$): δ -61.91

^{11}B NMR (128 MHz, DMSO-d$_6$): δ -6.82

Synthesis of 4-formyl-N,N,N-trimethylanilinium tetrakis(3,5-bis-(trifluoromethyl)phenyl) borate, ***3f***

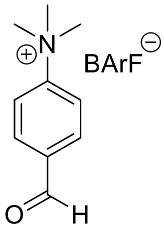

Prepared according to *General Procedure E*.

Amount of 4-formyl-*N,N,N*-trimethylanilinium iodide: 0.146 g, 0.50 mmol, 1 equiv.
Amount of tetrakis(3,5-bis(trifluoromethyl)phenyl) borate: 0.443 g, 0.50 mmol, 1 equiv.
Product yield: 0.246 g, 0.24 mmol, 48%

Melting point: 159-161 °C

IR: ν_{max} 1709, 1607, 1491, 1470, 1354, 1279, 1109, 1088 cm^{-1}

^{1}H NMR (400 MHz, DMSO-d$_6$): δ 3.66 (s, 9H, $N(CH_3)_3$), δ 7.63 (s, 8H, ArH BArF), δ 7.74 (s, 4H, ArH BArF), δ 8.19 (m, 4H, ArH anilinium), δ 10.12 (s, 1H, formyl)

^{13}C NMR (101 MHz, DMSO-d$_6$): δ 56.38, 117.56 (m), 121.68, 123.94 (q, $J_{CF}{}^1$ = 273 Hz), 128.37 (q, $J_{CF}{}^2$ = 33 Hz), 130.82, 134.00, 136.77, 151.01, 160.91 (q, $J_{CB}{}^1$ = 50 Hz), 192.00

^{19}F NMR (376 MHz, DMSO-d$_6$): δ -61.90

^{11}B NMR (128 MHz, DMSO-d$_6$): δ -6.83

Synthesis of 4-benzoyl-N,N,N-trimethylanilinium tetrakis(3,5-bis-(trifluoromethyl)phenyl) borate, ***4f***

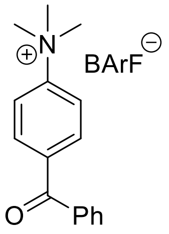

Prepared according to *General Procedure E*.

Amount of 4-benzoyl-*N,N,N*-trimethylanilinium iodide: 0.367 g, 1 mmol, 1 equiv.
Amount of tetrakis(3,5-bis(trifluoromethyl)phenyl) borate: 0.886 g, 1.00 mmol, 1 equiv.
Product yield: 0.871 g, 0.79 mmol, 79%

Melting point: 151-152 °C

IR: ν_{max} 1665, 1607, 1354, 1273, 1113 cm^{-1}

^{1}H NMR (400 MHz, DMSO-d$_6$): δ 3.68 (s, 9H, N(CH$_3$)$_3$), δ 7.62 (m, 10H, ArH BArF and ArH anilnium), δ 7.75 (m, 7H, ArH BArF and ArH anilinium), δ 7.96 (m, 2H, ArH anilinium), δ 8.17 (m, 2H, ArH anilinium)

^{13}C NMR (101 MHz, DMSO-d$_6$): δ 56.40, 117.60 (m), 121.05, 123.96 (q, $J_{CF}{}^1$ = 273 Hz), 128.31 (m), 128.74, 129.74, 130.85, 133.37, 134.02, 136.09, 138.52, 149.59, 160.88 (q, $J_{CB}{}^1$ = 50 Hz), 194.41

^{19}F NMR (376 MHz, DMSO-d$_6$): δ -61.61

^{11}B NMR (128 MHz, DMSO-d$_6$): δ -6.83

Synthesis of 4-benzoyl-N,N,N,-trimethylanilinium tetrakis(3,5-bis-(trifluoromethyl)phenyl) borate, ***4f***

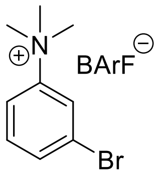

Prepared according to *General Procedure E*.

Amount of 3-bromo-*N,N,N*-trimethylanilinium iodide: 0.367 g, 1 mmol, 1 equiv.
Amount of tetrakis(3,5-bis(trifluoromethyl)phenyl) borate: 0.886 g, 1.00 mmol, 1 equiv.
Product yield: 0.871 g, 0.79 mmol, 79%

Melting point: 154-155 °C

IR: ν_{max} 2359, 1611, 1354, 1273, 1111, 885, 839, 710, 669 cm^{-1}

^{1}H NMR (400 MHz, DMSO-d$_6$): δ 3.62 (s, 9H, $N(CH_3)_3$), δ 7.57-7.65 (m, 9H, ArH BArF and ArH anilnium), δ 7.81 (m, 4H, ArH BArF), δ 8.01 (m, 2H, ArH anilinium), δ 8.27 (m, 2H, ArH anilinium)

^{13}C NMR (101 MHz, DMSO-d$_6$): δ 56.44, 117.53 (m), 119.81, 122.48, 123.85, 123.96 (q, J_{CF}^1 = 271 Hz), 128.42 (q, J_{CF}^2 = 30 Hz), 131.73, 133.09, 133.99, 148.21, 160.91 (q, J_{CB}^1 = 50 Hz)

^{19}F NMR (376 MHz, DMSO-d$_6$): δ -61.84

^{11}B NMR (128 MHz, DMSO-d$_6$): δ -6.83

*Synthesis of N,N,N,2-tetramethylanilinium tetrakis(3,5-bis-(trifluoromethyl)phenyl) borate, 14**f***

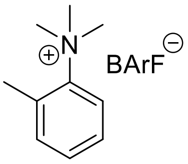

Prepared according to *General Procedure E*.

Amount of *N,N,N,2*-tetramethylanilinium iodide: 0.277 g, 1.00 mmol, 1 equiv.
Amount of tetrakis(3,5-bis(trifluoromethyl)phenyl) borate: 0.886 g, 1 mmol, 1 equiv.
Product yield: 0.902 g, 0.89 mmol, 89%

Melting point: 132-134 °C

IR: ν_{max} 1610, 1490, 1355, 1276, 1136, 1112, 1088 cm^{-1}

^{1}H NMR (400 MHz, DMSO-d$_6$): δ 2.73 (s, 3H, Ar-CH_3), δ 3.68 (s, 9H, $N(CH_3)_3$), δ 7.47 (m, 3H, ArH anilinium), δ 7.62 (s, 8H, ArH BArF), δ 7.70 (s, 4H, ArH BArF), δ 7.85 (m, 1H, ArH anilinium)

^{13}C NMR (101 MHz, DMSO-d$_6$): δ 22.48, 56.46, 117.44 (m), 121.11, 123.84 (q, J_{CF}^1 = 274 Hz), 127.99, 128.44 (q, J_{CF}^2 = 31 Hz), 130.09, 130.32, 134.00, 135.57, 145.19, 160.94 (q, J_{CB}^1 = 50.5 Hz)

^{19}F NMR (376 MHz, DMSO-d$_6$): δ -61.91

^{11}B NMR (128 MHz, DMSO-d$_6$): δ -6.83

4. Thermal Gravimetric Analysis (TGA)

All compound numbers in this section and throughout refer to the structures summarised in manuscript ***Figure 3****.*

The *N,N,N*-trimethylanilinium salt of interest (approx. 10 mg) was loaded into the TGA instrument. The sample was heated from 40-140 °C at a rate of 5 °Cmin^{-1} then 140-300 °C at a rate of 10 °Cmin^{-1} under an argon atmosphere.

The temperature vs. weight% trace for each sample is shown in **figure S1**. Onset and peak temperatures for each sample are given in **table S1**.

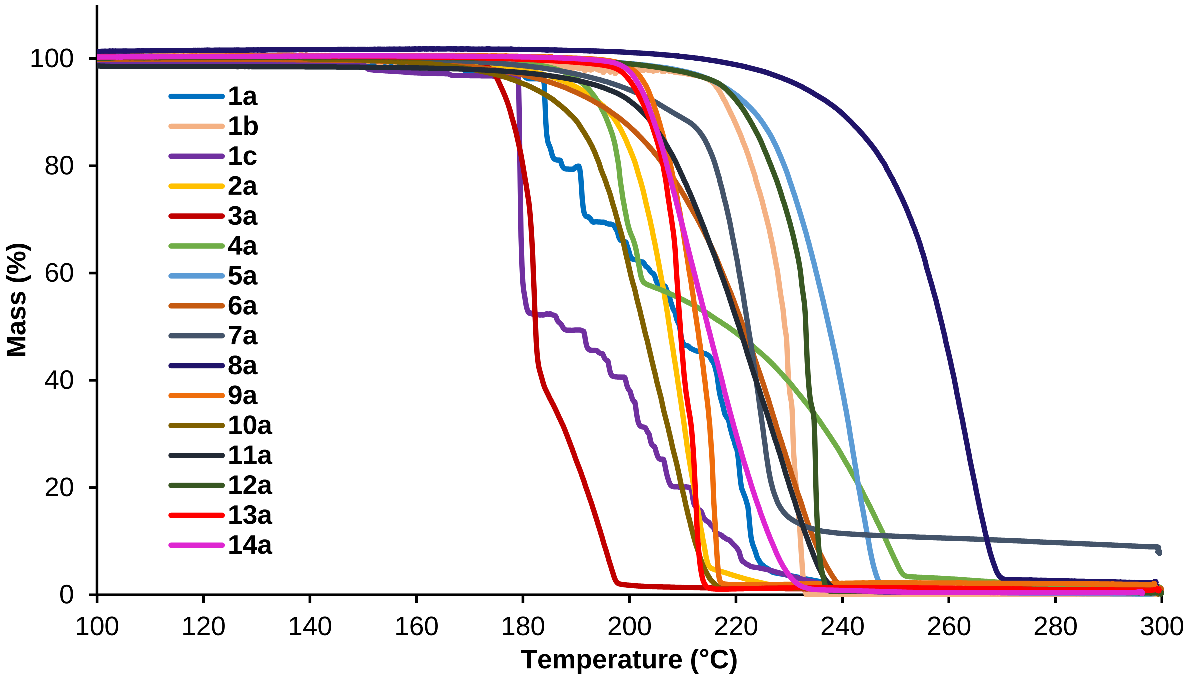

Figure S1. Weight vs. temperature trace determined via thermal gravimetric analysis under argon for salts **1a-14a**, **1b,** and **1c**, temperatures between 40-100 °C have been omitted as no change in weight was observed for any of the salts in this temperature range.

Anilinium salt	Onset (°C)	Peak (°C)
**1a**	184	184
**1b**	221	229
**1c**	179	179
**2a**	199	210
**3a**	180	182
**4a**	195	198
**5a**	227	242
**6a**	204	229
**7a**	214	225
**8a**	248	261
**9a**	205	212
**10a**	191	210
**11a**	204	227
**12a**	230	235
**13a**	209	223
**14a**	187	203

Table S1. Onset and Peak temperatures for each of the anilinium salts subjected to thermal gravimetric analysis under argon.

The *N,N,N*-trimethylanilinium salt of interest (approx. 10 mg) was loaded into the TGA instrument. The sample was heated from 40-140 °C at a rate of 5 °Cmin^{-1} then 140-300 °C at a rate of 10 °Cmin^{-1} in air.

The temperature vs. weight% trace for each sample is shown in **figure S2**. Onset and peak temperatures for each sample are given in **table S2**.

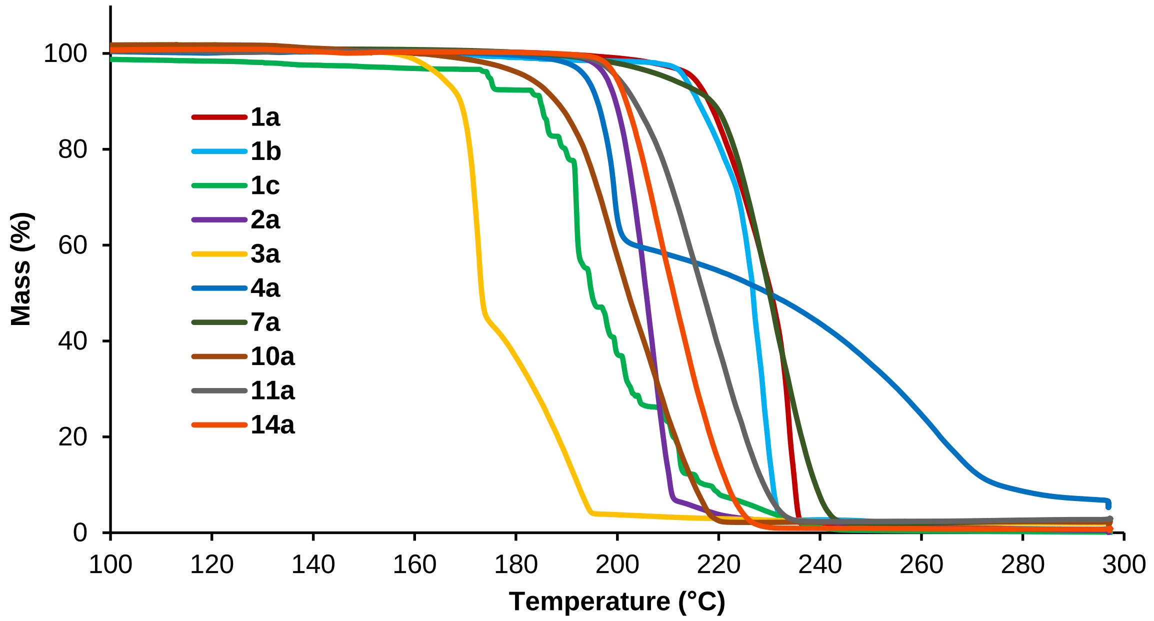

Figure S2. Weight vs. temperature trace determined via thermal gravimetric analysis in air for salts **1a-4a**, **7a**, **10a**, **11a**, **14a**, **1b,** and **1c**, temperatures between 40-100 °C have been omitted as no change in weight was observed for any of the salts in this temperature range.

Anilinium salt	Onset (°C)	Peak (°C)
1a	216	234
1b	212	229
1c	174	192
2a	199	207
3a	169,188	173
4a	196,237	199
7a	220	231
10a	188	198
11a	203	217
14a	200	208

Table S2. Onset and Peak temperatures for each of the anilinium salts subjected to thermal gravimetric analysis in air.

5. Thermal Volumetric Analysis – Sub-ambient Distillation (TVA-SAD)

TVA-SAD analyses were carried out using equipment built at the University of Strathclyde. The sample chamber was heated by a programmable heating furnace tube, and connected to a primary liquid nitrogen cooled sub-ambient trap. A two-stage rotary pump, and oil diffusion pumping system, was used to keep the system at a pressure of 1 x 10^{-3} Pa. A water jacket cooled cold ring was used to condense high boiling point degradants. Volatile condensable products were collected by the primary liquid nitrogen-cooled sub-ambient trap; a linear response Pirani gauge monitored volatiles entering the sub-ambient trap a function of pressure vs. temperature. Non-condensable volatiles were measured by a second Pirani gauge when exiting the sub-ambient trap as a function of pressure vs. temperature. The sub-ambient trap was then warmed slowly to ambient temperature, the volatised species were distilled in secondary cold traps and submitted to gas-phase FTIR analysis.

TVA-SAD experiments were carried out by placing the *N,N,N*-trimethylanilinium salt of interest (100 mg) in the sample chamber. The sample was then heated from ambient temperature to 400 °C at a rate of 30 °Cmin^{-1} under vacuum.

Compounds **1a**, **6a**, and **12a** were tested using TVA-SAD analysis, the pressure change over time as the samples were subjected to the heating programme is shown in **figure S3**. The three curves reach a peak around 240 °C and compound **12a** has an additional peak evolving around 180 °C.

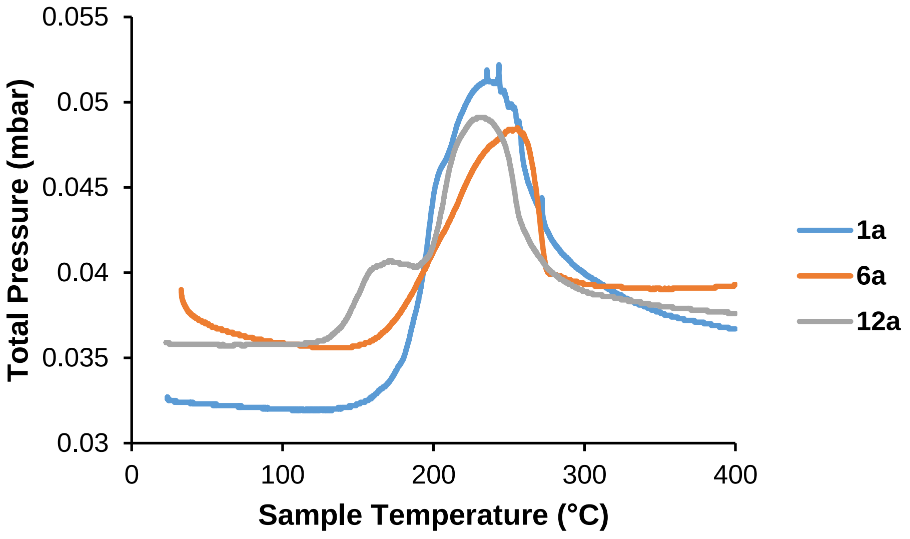

Figure S3. Pressure change vs. temperature at sample furnace for salts **1a**, **6a**, and **12a**, determined by thermal volumetric analysis.

The cold trap containing volatile compounds was then slowly heated from -200 °C to ambient temperature, the pressure change vs temperature during the secondary heating process is shown in **figure S4**. The pressure changes at -150 °C and -50 °C correspond to the sublimation of CO_2 and H_2O peaks emerging, respectively. The pressure change around 0 °C is suspected to be due to the volatilisation of starting compounds. The peak occurring around -120 °C is believed to be due to the evolution of methyl iodide, the FTIR spectrum of the sample taken at for this peak in each sample is shown in **figure S5**.

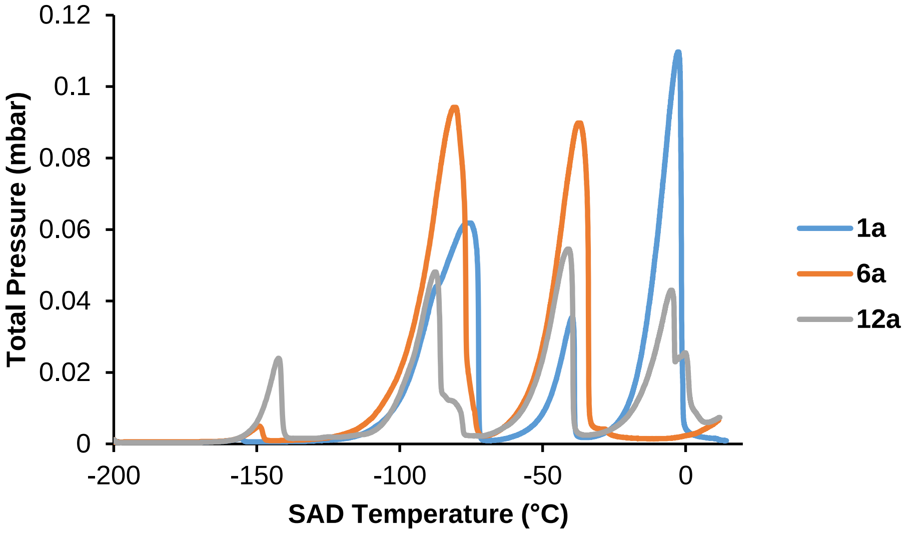

Figure S4. Pressure change vs. temperature when heating cold trap to ambient temperature for salts **1a**, **6a**, and **12a**, determined by thermal volumetric analysis.

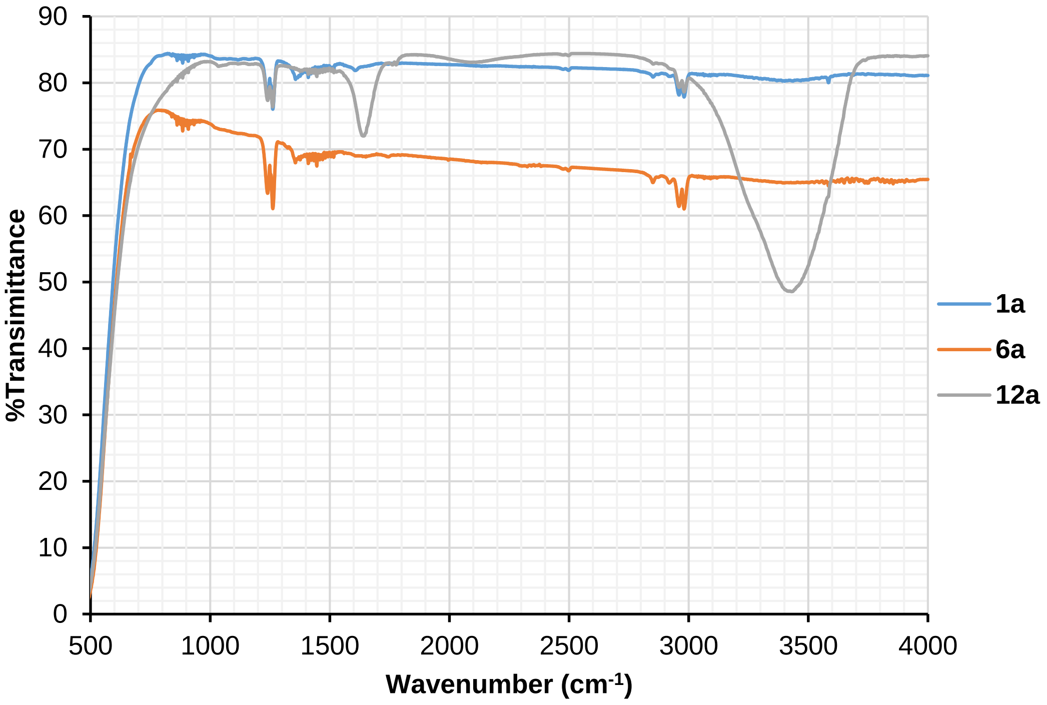

Figure S5. FTIR spectra for volatile compounds released from the heating of **1a**, **6a**, and **12a**. The peaks correspond to methyl iodide.

6. Degradation of *N,N,N*-Trimethylanilinium iodide salts in DMSO-d_6

The relevant substituted *N,N,N*-trimethylanilinium iodide (36.0 μmol) and 1,2,4,5-tetramethylbenzene internal standard (0.8 mg, 6.0 μmol) wa dissolved in DMSO-d_6 (0.6 mL, 0.7140 g), to give an anilinium concentration of 0.06 M and internal standard concentration of 0.01 M. The solution was transferred to an NMR tube and a ^{1}H NMR spectrum was recorded. The solution was heated to 120 °C for 20 min, then the NMR tube was placed in ice to halt the degradation. A ^{1}H NMR spectrum was recorded of the degradation mixture.

The concentration of the anilinium salt was calculated *via* the integration of its aromatic protons against those of the internal standard. The average %degradation value and error from a triplicate of experiments is shown in **figure S6** and **table S3**.

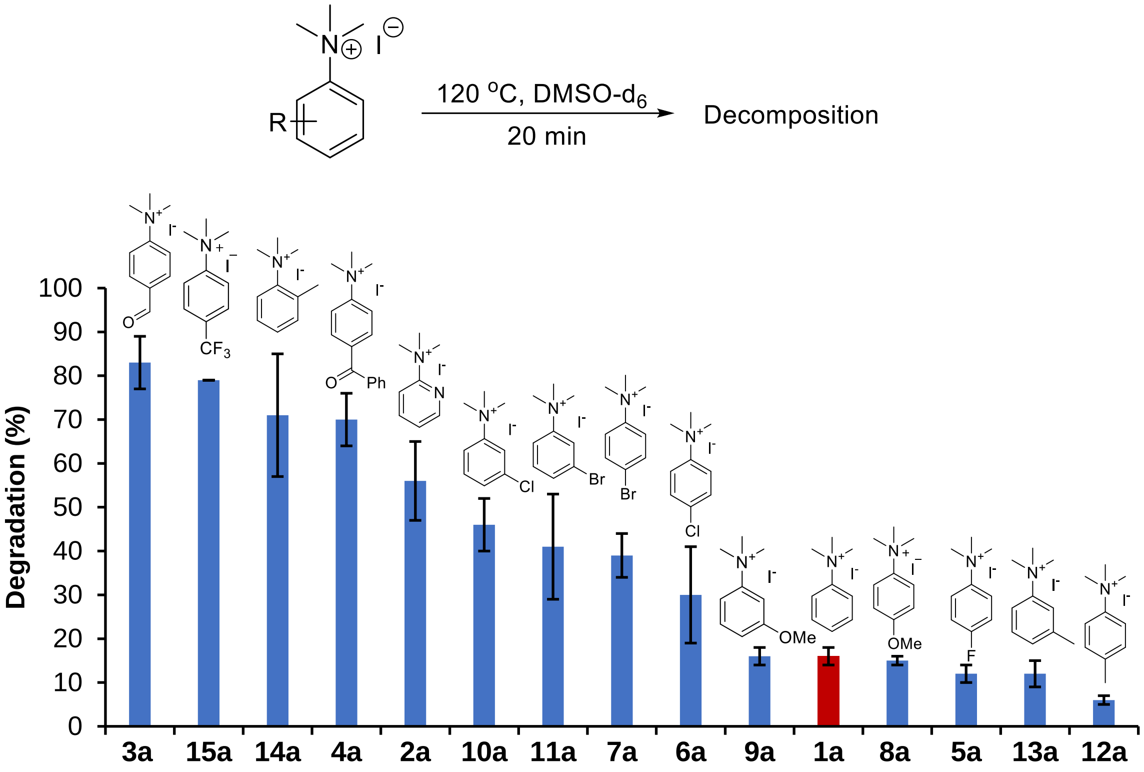

Figure S6. %Degradation of a range of *N,N,N*-trimethylanilinium iodides upon heating in DMSO-d_6 at 120 °C for 20 min, 1,2,4,5-tetramethylbenzene was used as an internal standard to calculate the concentration of the anilinium salt before and after heating.

Anilinium salt	Degradation (%)	Error (%)
**1a**	15.50	2.16
**2a**	56.08	8.67
**3a**	82.78	5.81
**4a**	70.27	6.33
**5a**	12.08	2.04
**6a**	30.36	10.99
**7a**	39.01	5.45
**8a**	15.11	1.42
**9a**	16.28	2.14
**10a**	46.12	5.75
**11a**	10.96	11.92
**12a**	5.88	0.83
**13a**	11.81	2.77
**14a**	71.26	14.41
**15a**	78.97	0.17

Table S3. %Degradation of a range of *N,N,N*-trimethylanilinium iodides upon heating in DMSO-d_6 at 120 °C for 20 min.

7. Effect of Halide Counterion of Degradation

The relevant *N,N,N*-trimethylanilinium halide (36.0 μmol) and 1,2,4,5-tetramethylbenzene internal standard (4.8 mg, 36 μmol) was dissolved in DMSO-d_6 (0.6 mL, 0.7140 g), to give an anilinium concentration of 0.06 M and internal standard concentration of 0.06 M. The solution was transferred to an NMR tube and a ^{1}H NMR spectrum was recorded. The solution was heated to 120 °C for 20 min, then the NMR tube was placed in ice to halt the degradation. A ^{1}H NMR spectrum was recorded of the degradation mixture.

The concentration of the anilinium salt was calculated *via* the integration of its aromatic protons against those of the internal standard. The average %degradation value and error from a triplicate of experiments is shown in **figure S7** and **table S4**.

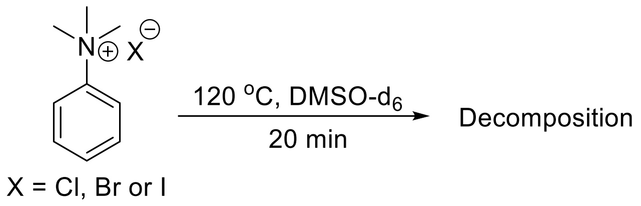

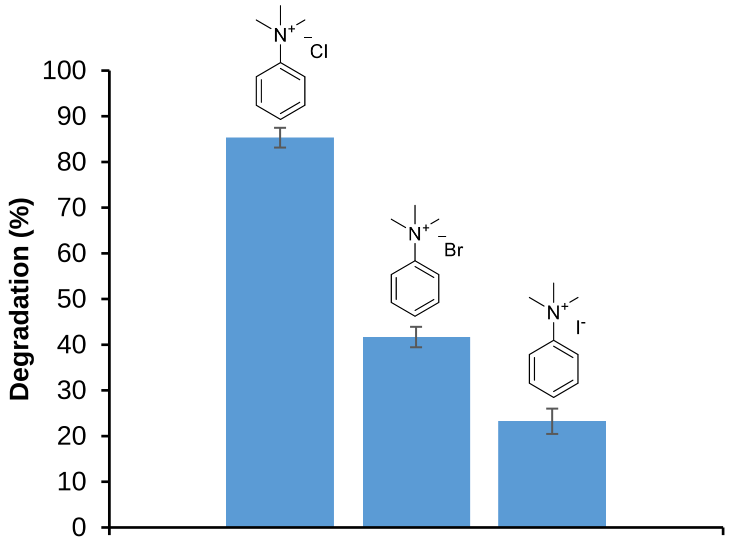

Figure S7. %Degradation of **1a**, **1b**, and **1c** upon heating in DMSO-d_6 at 120 °C for 20 min.

Anilinium salt	Degradation (%)	Error (%)
**1a**	23.25	2.77
**1b**	41.69	2.23
**1c**	85.32	2.16

Table S4. %Degradation of **1a**, **1b**, and **1c** upon heating in DMSO-d_6 at 120 °C for 20 min.

8. Effect of halide additive on the degradation of *N,N,N*-trimethylanilinium iodide in DMSO-d_6

N,N,N-trimethylanilinium iodide (9.5 mg, 36 μmol, 1 equiv.), the relevant halide salt (36 μmol, 1 equiv.), and 1,2,4,5-tetramethylbenzene internal standard (4.8 mg, 36 μmol) was dissolved in DMSO-d_6 (0.6 mL, 0.7140 g), to give an anilinium, additive, and internal standard concentration each of 0.06 M. The solution was transferred to an NMR tube and a ^{1}H NMR spectrum was recorded. The solution was heated to 120 °C for 20 min, then the NMR tube was placed in ice to halt the degradation. A ^{1}H NMR spectrum was recorded of the degradation mixture.

The concentration of the anilinium salt was calculated *via* the integration of its aromatic protons against those of the internal standard. The average %degradation value and error from a triplicate of experiments is shown in **figure S8** and **table S5**.

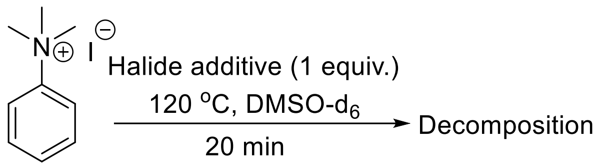

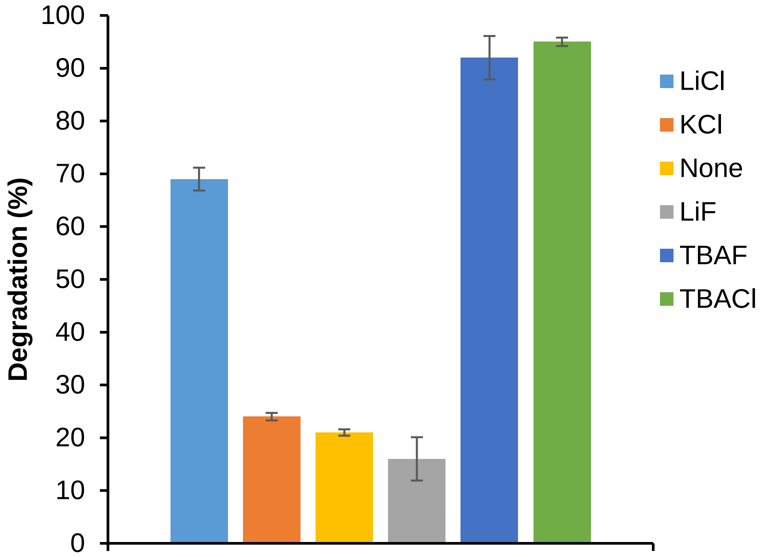

Figure S8. %Degradation of N,N,N-trimethylanilinium iodide upon heating in DMSO-d_6 at 120 °C for 20 min in the presence of 1 equiv. halide additive.

Halide salt	Degradation (%)	Error (%)
None	21.4	0.6
LiCl	69.4	2.2
KCl	24.0	0.7
TBACl	94.6	0.8
LiF	15.7	4.1
TBAF	92.3	7.6

Table S5. %Degradation of *N,N,N*-trimethylanilinium iodide upon heating in DMSO-d_6 at 120 °C for 20 min in the presence of 1 equiv. halide additive.

9. Effect of water on the degradation of *N,N,N*-trimethylanilinium iodide

Stock solutions of maleic acid (7.0 mg, 36 μmol) in DMSO-d_6 (1.19 g, 1.0 mL), and maleic acid (7.0 mg, 36 μmol) in D_2O (1,11 g, 1 mL) were prepared.

To *N,N,N*-trimethylanilinium iodide (9.5 mg, 36 μmol) and tetrabutylammonium fluoride (10.0 mg, 36 μmol), either 0.6 mL DMSO-d_6 stock solution, 0.6 mL D_2O stock solution, or 0.3 mL of each stock solution, were added. The solution was transferred to an NMR tube and a ^1H NMR spectrum was recorded. The solution was heated to 90 °C for 20 min, then the NMR tube was placed in ice to halt the degradation. A ^1H NMR spectrum was recorded of the degradation mixture.

The concentration of the anilinium salt was calculated *via* the integration of its aromatic protons against the olefinic protons of the internal standard. The %degradation value in DMSO-d_6, 1:1 DMSO-d_6:D_2O, and D_2O is shown in **figure S9**.

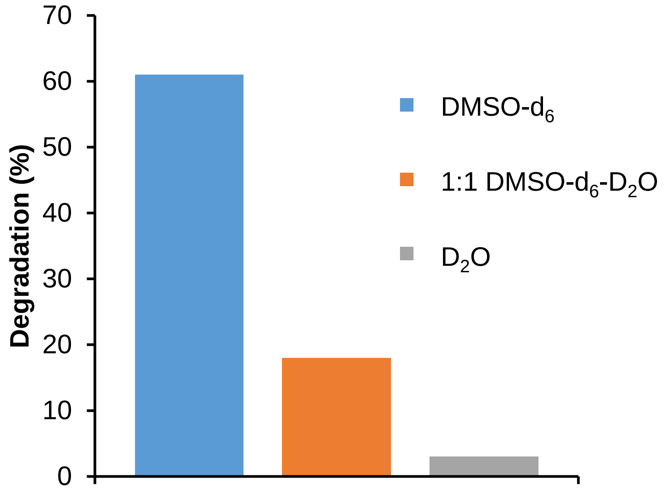

Figure S9. Degradation of *N,N,N*-trimethylanilinium iodide with tetrabutylammonium fluoride in either DMSO-d_6, D_2O, or 1:1 DMSO-d_6:D_2O after heating at 90 °C for 20 min.

10. Effect of non-nucleophilic counterion on degradation of *N,N,N*-trimethylanilinium salts

The relevant *N,N,N*-trimethylanilinium salt (36 μmol) and 1,2,4,5-tetramethylbenzene internal standard (4.8 mg, 36 μmol) was dissolved in DMSO-d_6 (0.6 mL, 0.7140 g), to give an anilinium, and internal standard concentration each of 0.06 M. The solution was transferred to an NMR tube and a ^{1}H NMR spectrum was recorded. The solution was heated to 120 °C for 1 h, then the NMR tube was placed in ice to halt the degradation. A ^{1}H NMR spectrum was recorded of the degradation mixture.

The concentration of the anilinium salt was calculated *via* the integration of its aromatic protons against those of the internal standard. Each experiment was performed in triplicate. The average %degradation value for each of the salts is shown in **figure S10** and **table S6**.

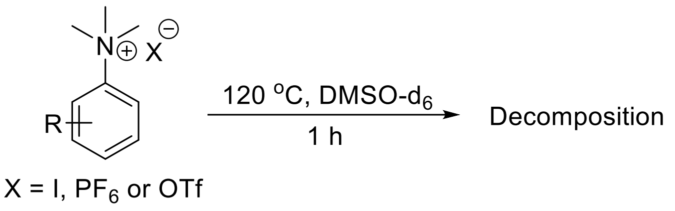

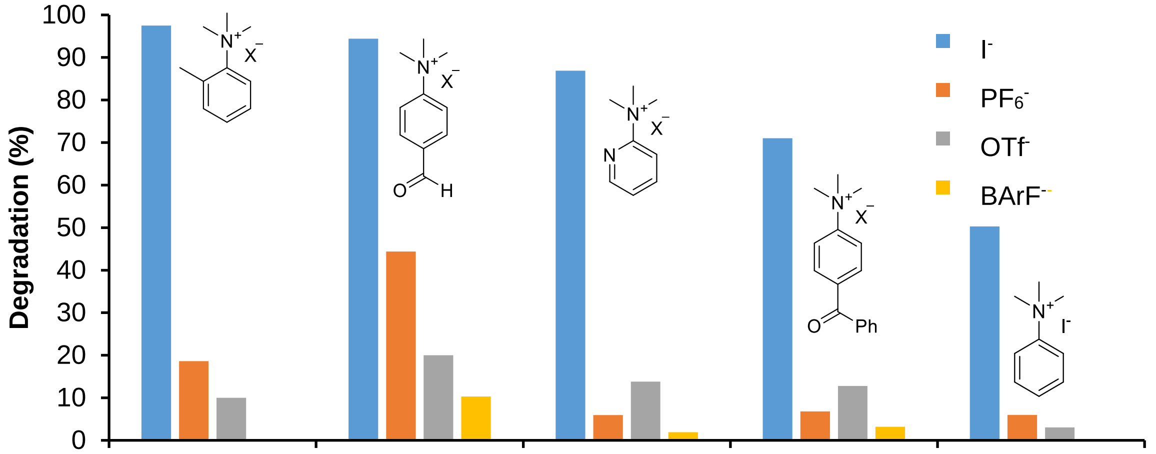

Figure S10. %Degradation of **1-4a, d**, **e** and **f**, and **14a, d**, **e** and **f**, upon heating in DMSO-d_6 at 120 °C for 1 h.

Anilinium salt	Degradation (%)
**1a**	97.5
**2a**	94.4
**3a**	86.9
**4a**	71.0
**14a**	50.3
**1d**	18.6
**2d**	44.4
**3d**	5.95
**4d**	6.80
**14d**	5.9_6
**1e**	10.0
**2e**	20.0
**3e**	13.8
**4e**	12.8
**14e**	3.03
**1f**	0.0
**2f**	10.3
**3f**	1.9
**4f**	3.2
**14f**	0.3

Table S6. %Degradation of **1-4a, d** and **e**, and **14a, d** and **e**, upon heating in DMSO-d_6 at 120 °C for 1 h.

11. NMR Degradation Monitoring of *N*,*N*,*N*-trimethylanilinium iodides

The relevant *N,N,N*-trimethylanilinium iodide (60 μmol) and 1,2,4,5-tetramethylbenzene internal standard (4.8 mg, 36 μmol) was dissolved in DMSO-d_6 (0.6 mL, 0.7140 g), to give an anilinium concentration of 0.1 M, and an internal standard concentration of 0.06 M, then transferred to an NMR tube. A ^{1}H NMR spectrum (4 scans, 25 s relaxation time) was recorded every 5 min whilst the sample was heated to 80 °C in the NMR spectrometer probe.

The concentration of the anilinium salt, aniline, methyl iodide, trimethylsulfoxonium-d_6 cation (TMSO-d_6), and dimethylsulfoxide-d_3 (DMSO-d_3) was calculated against the internal standard at each time point. The anilinium and aniline concentration was calculated from both aromatic protons and N-methyl protons.

An exemplar stacked spectrum is shown in **figure S11** for the degradation of **3a**, and a graph of the calculated species concentrations vs time is shown in **figure S12**. The process was repeated on a range of *N,N,N*-trimethylanilinium iodides, the anilinium concentration calculated *via* aromatic protons vs time is shown for all of the salts tested in **figure S13**.

According to the first proposed degradation pathway (**figure S14**), three separate reversible reactions were identified to occur in the degradation mixtures: iodide-led anilinium degradation, DMSO-led anilinium degradation, and reaction between methyl iodide and DMSO. The three reactions shown in **figure S14** have been assigned rate constants k_1, k_{-1}, k_2, k_{-2}, k_3, and k_{-3}. Using COPASI v4.22, values for each rate constant in this reaction model were estimated. The modelling was programmed in such a way that priority would be given to correlating simulated data to the obtained anilinium concentration vs time curve. A summary of the rate constants obtained is shown in **table S7**. Rate constants were determined for each step of this first proposed degradation pathway (**figure S14**) using COPASI, the estimated values are shown in **table S10**. Deeper microkinetic analysis using COPASI is reported in Supporting Information **Section 17**, below.

A Swain-Lupton analysis was performed using the derived k_1 values for each salt to reveal the resonance (R) and field (F) contributions of aryl-substituents to be R = 51% and F = 49% (**figure S15**).

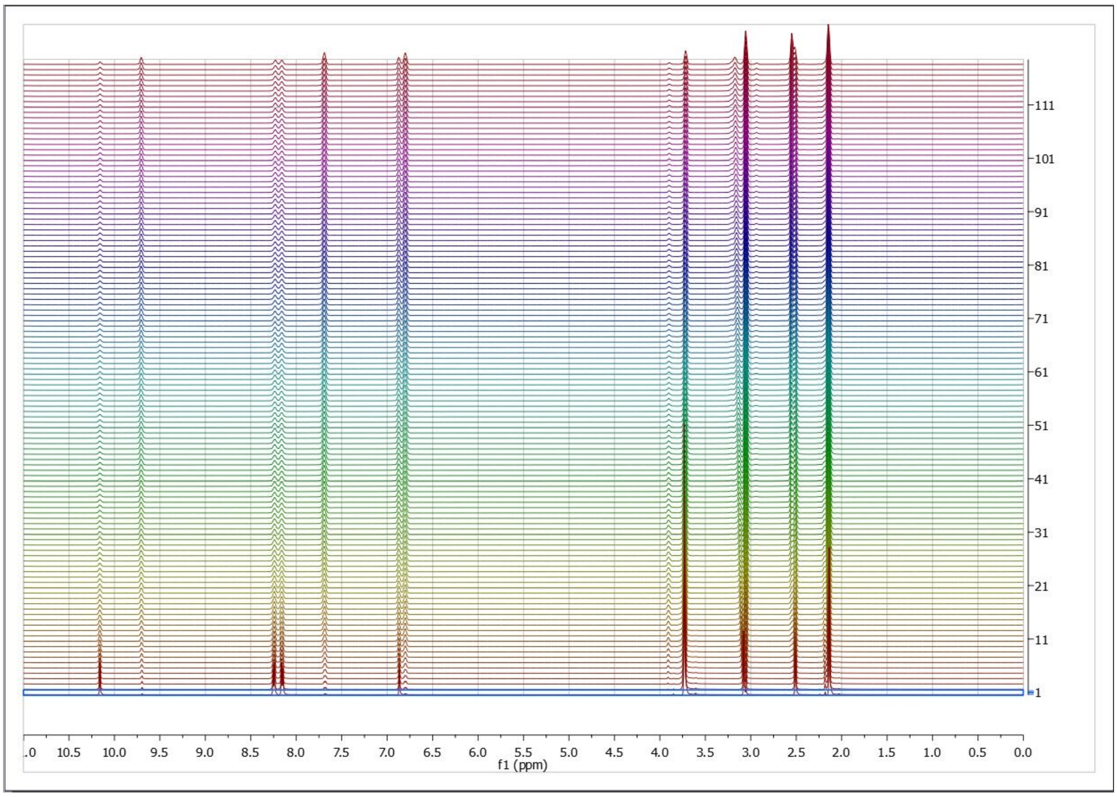

Figure S11. Stacked NMR spectra monitoring the degradation of 0.1 M **3a** in DMSO-d_6 at 80 °C.

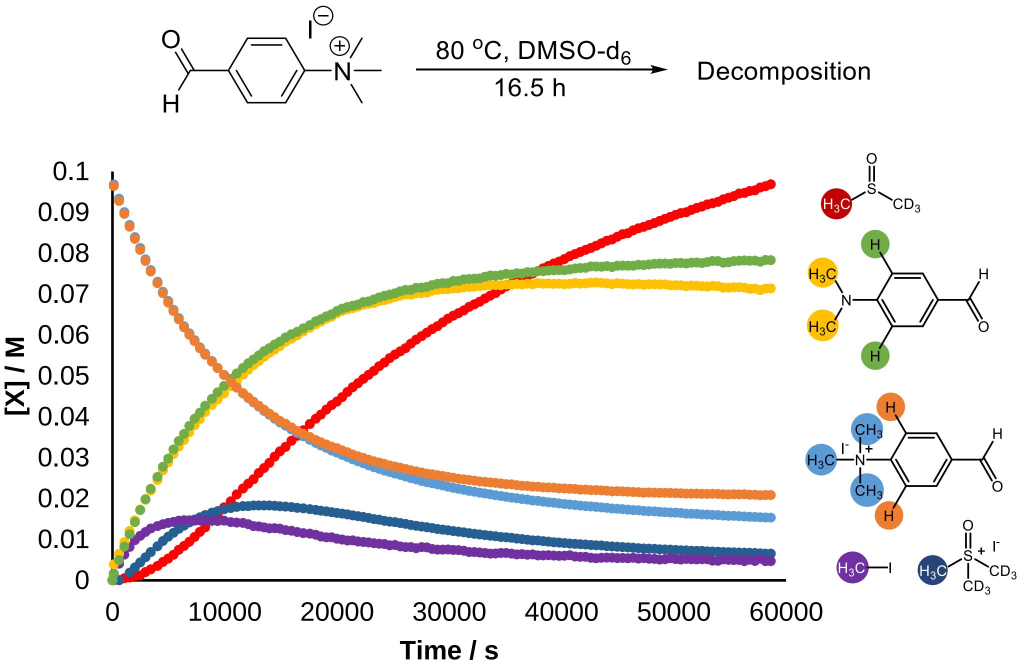

Figure S12. Real-time ^{1}H NMR monitoring of the degradation of **3a** *via* -$N^+(CH_3)_3$ (light blue) and *o*-aryl (orange) protons, evolution of 4-formyl-aniline via N-$(CH_3)_2$ (yellow) and *o*-aryl (green) protons, methyl iodide (purple), DMSO-d_3 (red) and intermediate TMSO-d_6 iodide (dark blue).

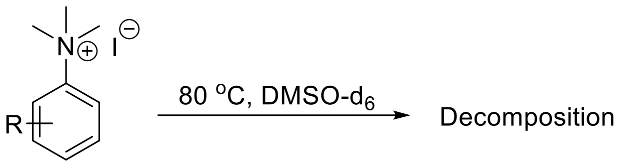

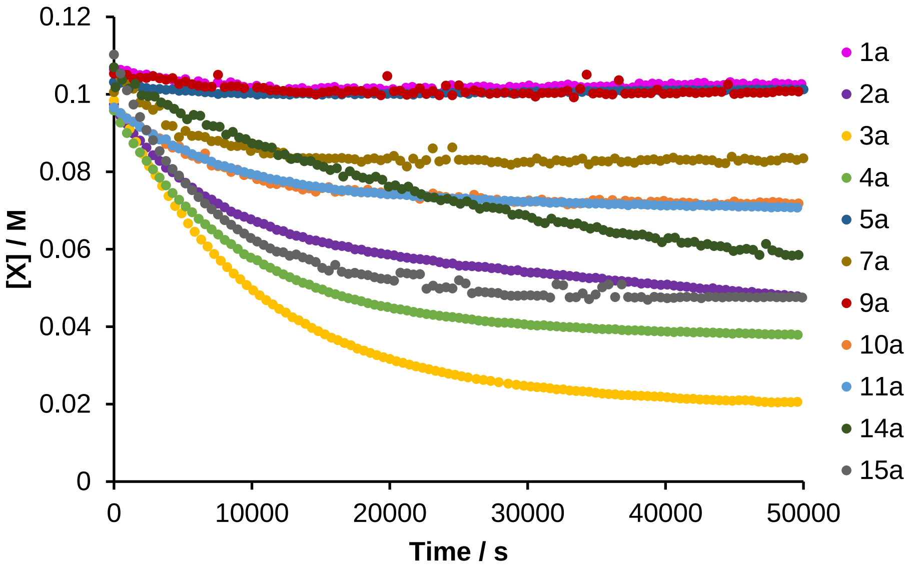

Figure S13. Plots of anilinium degradation vs time for a range of substituted *N,N,N*-trimethylanilinium iodides in DMSO-d_6 at 80 °C determined by real-time ^{1}H NMR spectroscopic analysis and the relative initial degradation rates calculated for each of these salts.

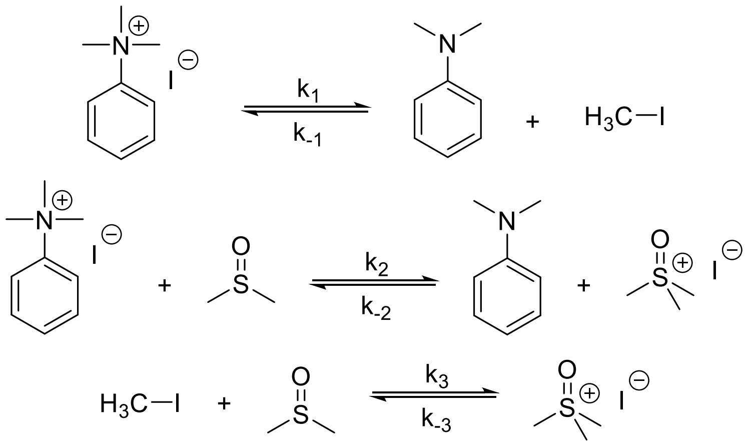

Figure S14. Reaction model used to estimate rate constants in anilinium degradation models.

Salt	k_1 (s^{-1})	k_{-1} ($M^{-1}s^{-1}$)	k_2 ($M^{-1}s^{-1}$)	k_{-2} ($M^{-1}s^{-1}$)	k_3 ($M^{-1}s^{-1}$)	k_{-3} (s^{-1})
1a	1.11E-05	1.80E-95	1.595E-315	1.63E+07	1.81E-05	1.82E-04
2a	4.47E-05	1.95E-03	3.74E-208	1.36E-172	1.94E-162	0.00E+00
3a	7.95E-05	5.46E-04	1.71E-283	1.30E-13	5.53E-06	5.27E-05
4a	6.41E-05	7.94E-276	6.96E-134	1.10E+08	2.95E-06	5.84E-23
5a	9.45E-06	1.69E-02	2.85e-315	1.56E-02	1.79E-316	0.00E+00
7a	1.85E-05	6.06E-03	1.577E-315	1.13E-306	6.341E-314	7.78E-04
9a	4.46E-06	5.00E-259	1.82E-180	3.43E-02	1.66E-05	7.47E-212

10a	2.53E-05	0.00E+00	4.74E-302	1.71E-02	7.70E-06	1.14E-04
11a	3.09E-05	1.36E-145	4.75E-48	3.46E-02	6.39E-06	7.05E-05
14a	1.89E-05	1.12E-85	9.96E-134	7.70E+11	1.59E-05	3.10E+11
15a	8.08E-05	3.09E-81	1.23E-44	8.66E+09	4.40E-06	1.79E-04

Table S7. Estimated rate constants k_1, k_{-1}, k_2, k_{-2}, k_3 and k_{-3}.

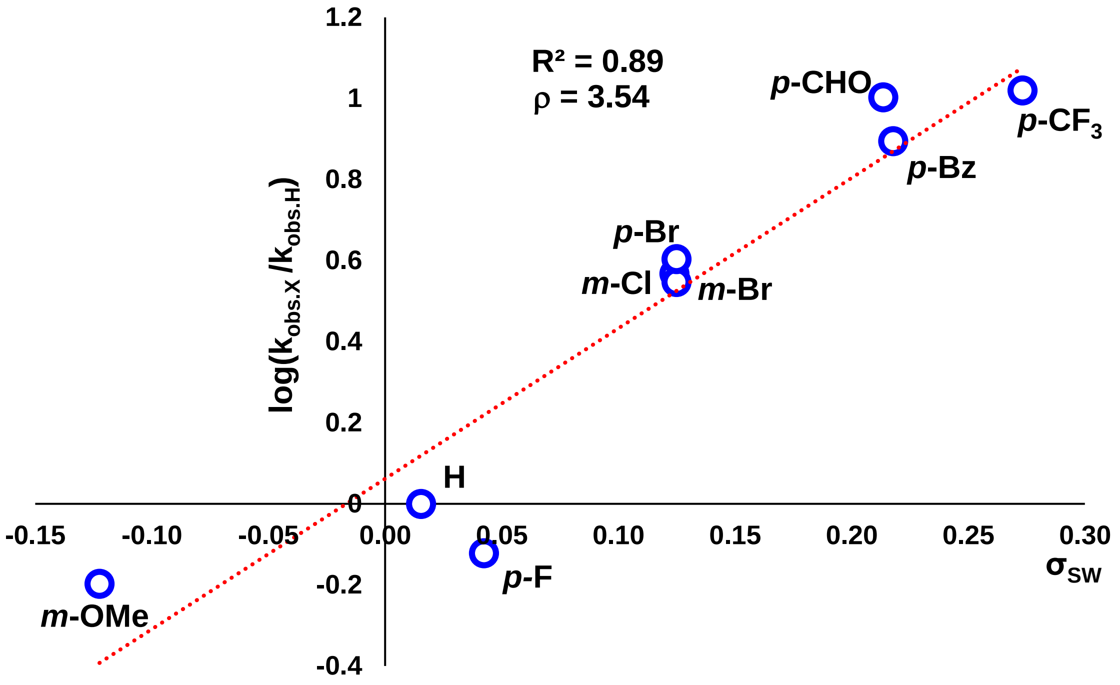

Figure S155. Swain-Lupton analysis using estimated k_1 values to give R = 51% and F = 49%.

Determination of T1 Relaxation Times for N,N,N-trimethylanilinium Iodides in DMSO-d6

In order to gain reliable kinetic degradation data, it was necessary to calculate the T1 relaxation times for protons of interest on the *N,N,N*-trimethylanilinium salts investigated. A sample of 0.1 M of each *N,N,N*-trimethylanilinium iodide in DMSO-d_6 was prepared and subjected to a T1 inversion recovery (T1IR) sequence. The T1IR experiment uses a 180°-τ-90° pulse sequence which simultaneously inverts and measures peaks with a varied delay time (τ) to create a pseudo-2D NMR spectrum. Processing the spectrum on Bruker Topspin 4.0.7 allows the user to create a plot of τ *vs* area for each peak of interest, an exemplar plot is shown below in **figure S16**.

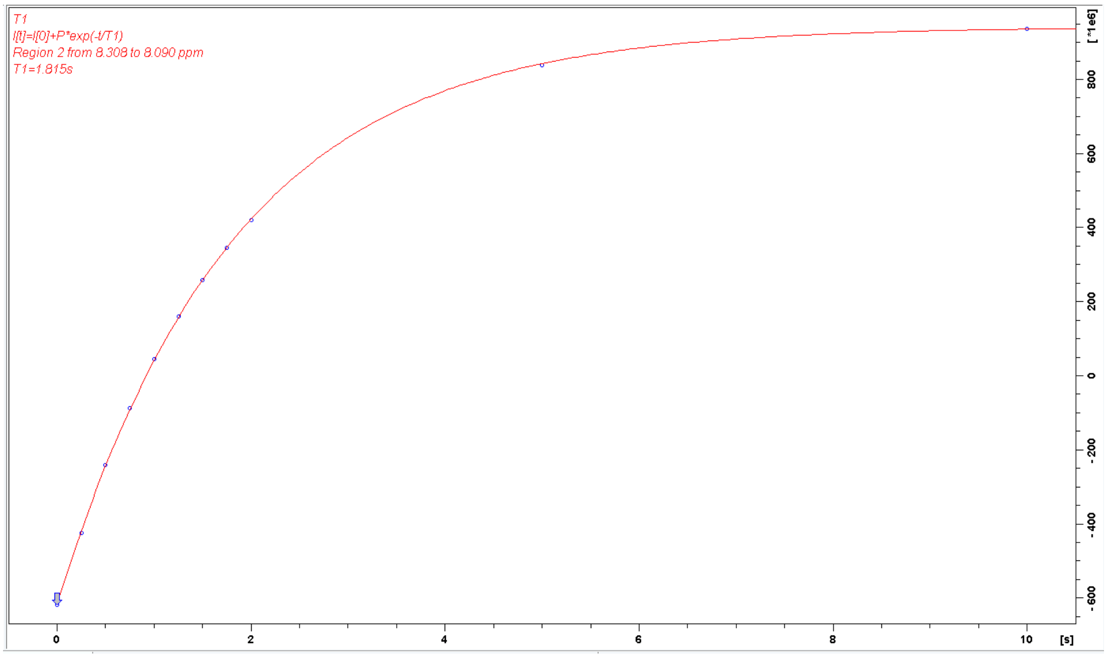

Figure S166. T1 inversion recovery plot in Bruker Topspin.

The following equation is fitted to the data where M_z is the magnetisation at t = τ s, and M_0 is the magnetisation at t = 0 s (maximum magnetisation).

$$M_z = M_0\left(1 - e^{-\frac{\tau}{T1}}\right)$$

Fitting the data to this equation is used to calculate T1, which corresponds to the time when magnetisation reaches 1 – 1/e (~63%) of its maximum value. Kinetic experiments were carried out with a delay time (D1) of at least 5x the longest calculated T1 time on each substrate. This ensured full relaxation of each nucleus, giving accurate peak area integrations for quantitative interpretation of spectra.

The calculated T1 relaxation times are displayed next to the relevant proton environment for each of the *N*,*N*,*N*-trimethylanilinium iodides investigated below. Values denoted with an asterix represent proton signals that were too close to be integrated separately; therefore, the value will represent that T1 of proton with the longest relaxation time.

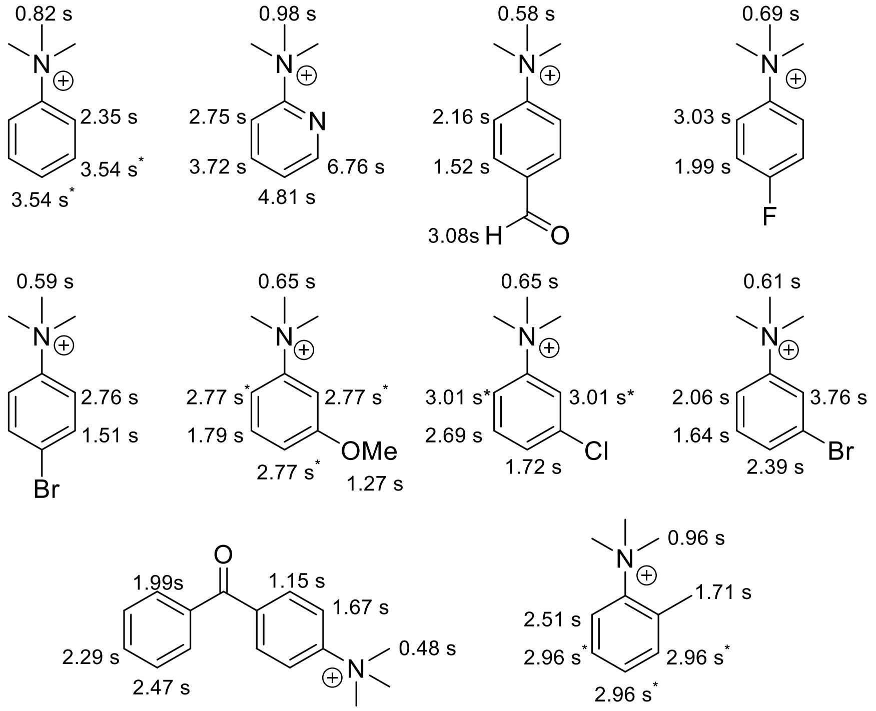

Figure S177. T1 data for selected anilinium salts, enabling a suitable, globally applicable relaxation delay to be set for kinetic experiments.

12. NMR Degradation Monitoring of 4-formyl-*N*,*N*,*N*-trimethylanilinium BArF

The relevant 4-formyl-*N,N,N*-trimethylanilinium BArF (61.6 mg, 60 µmol) and 1,2,4,5-tetramethylbenzene internal standard (4.8 mg, 36 µmol) was dissolved in DMSO-d_6 (0.6 mL, 0.7140 g), to give an anilinium concentration of 0.1 M, and an internal standard concentration of 0.06 M, then transferred to an NMR tube. A ^{1}H NMR spectrum (4 scans, 25 s relaxation time) was recorded every 5 min whilst the sample was heated to 80 °C in the NMR spectrometer probe.

The concentration of the anilinium salt and aniline was calculated against the internal standard at each time point. A graph of the calculated species concentrations vs time is shown in **figure S18**.

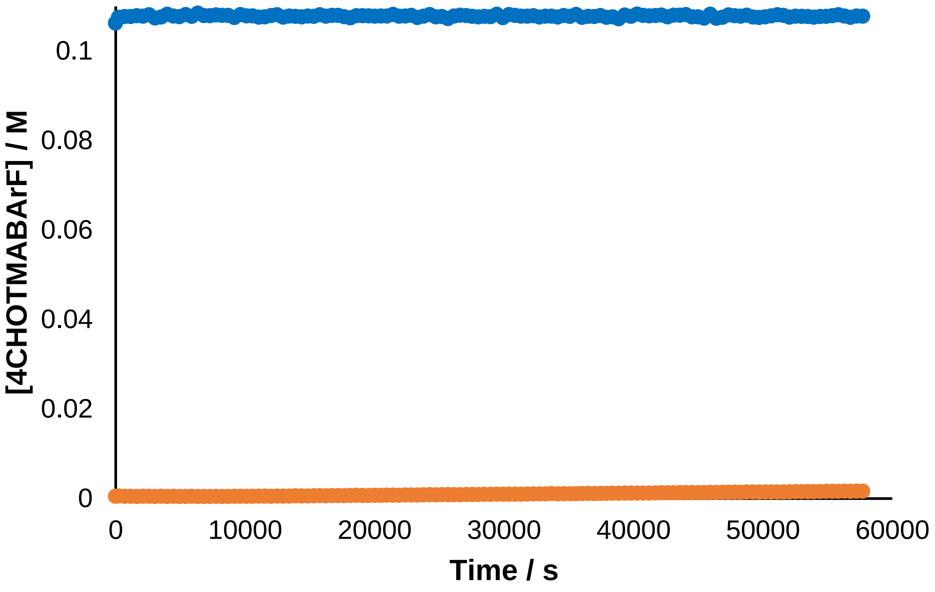

Figure S18. Real-time ^{1}H NMR monitoring of the degradation of **3f**, with anilinium concentration in blue and aniline concentration in orange.

13. Diffusion ordered spectroscopy (DOSY) to probe ion pairing of *N,N,N*-trimethylanilinium salts in DMSO-d_6

DOSY NMR was used.

The relevant *N,N,N*-trimethylanilinium salt was dissolved in DMSO-d_6 along with 1,2,4,5-tetramethylbenzene, 1,3,5-trimethoxybenzene and maleic acid as internal standards. A DOSY experiment using the parameters D20 = 0.4, P30 = 1500 and D21 = 0.005 was used to determine the diffusion coefficient (D) of the anilinium cation and internal standards. For each experiment a calibration plot of log(MW) versus log(D) was produced. A predicted MW for the anilinium species could be predicted from its calculated D value and the calibration plot. Below are the calculated D values for each analyte of each experiment, the calibration plot and the estimated anilinium molecular weight. These plots are collected and subtitled in **figure S19**, below.

4-formyl-N,N,N-trimethylanilinium iodide, ***3a*** *(0.05 M). D = 3.04 x 10^{-10} m^{2}s^{-1}. Predicted MW = 291 gmol^{-1}*

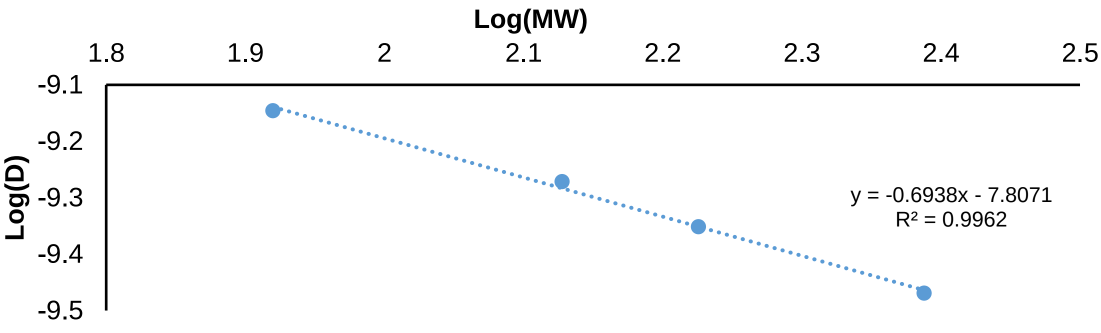

*4-formyl-N,N,N-trimethylanilinium iodide, **3a** (0.2 M). D = 2.73 x 10^{-10} m^2s^{-1}. Predicted MW = 284 $gmol^{-1}$*

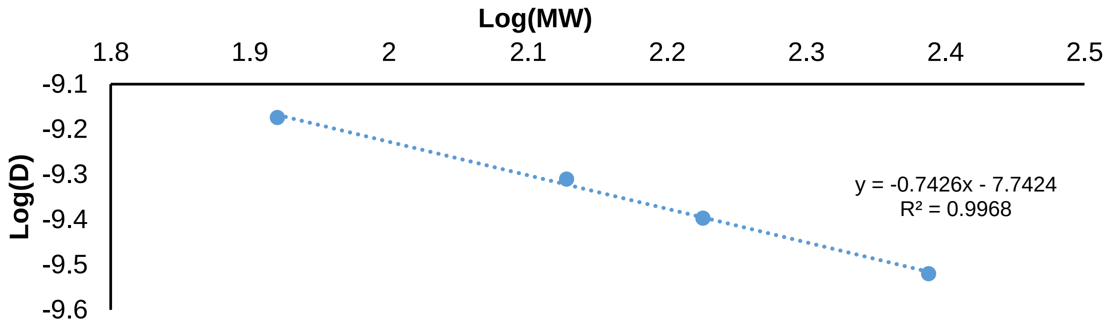

*N,N,N-trimethylanilinium chloride, **1c** (0.1 M). D = 3.36 x 10^{-10} m^2s^{-1}. Predicted MW = 252 $gmol^{-1}$*

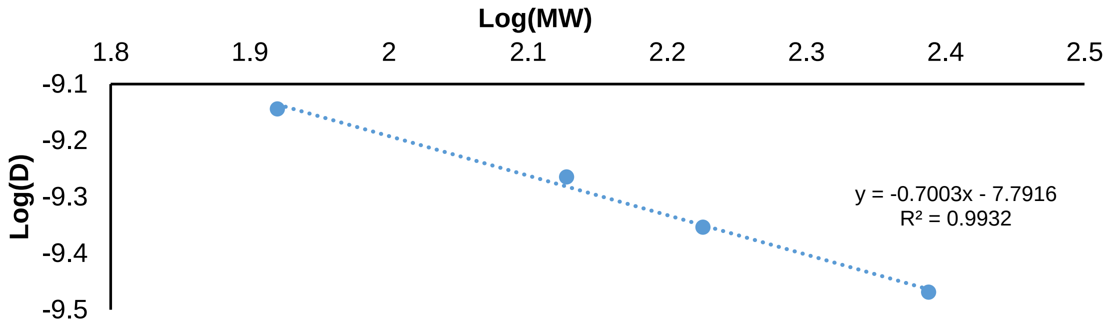

*N,N,N-trimethylanilinium bromide, **1b** (0.1 M). D = 3.37 x 10^{-10} m^2s^{-1}. Predicted MW = 246 $gmol^{-1}$*

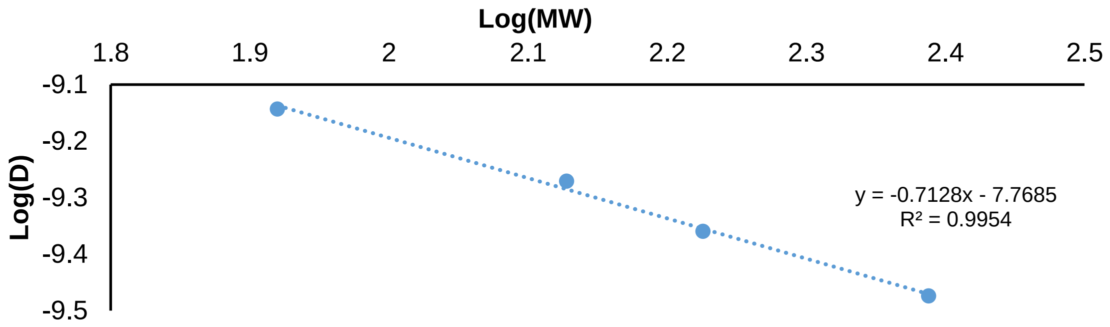

*N,N,N-trimethylanilinium iodide, **1a** (0.1 M). D = 3.41 x 10^{-10} m^2s^{-1}. Predicted MW = 241 $gmol^{-1}$*

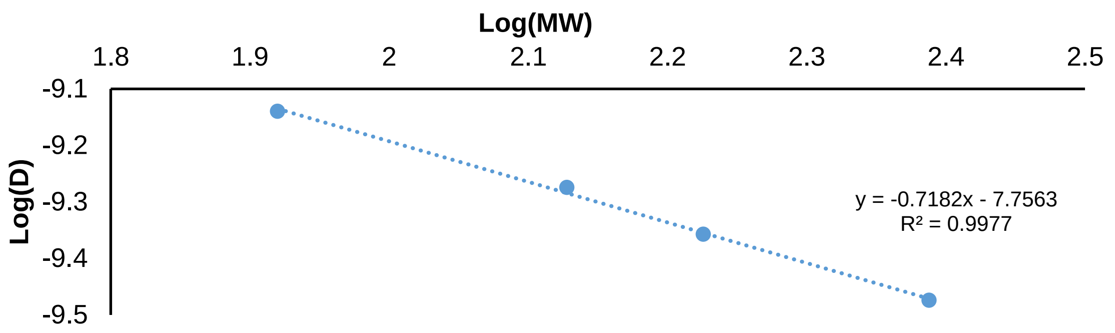

Figure S19. Collected ^{1}H DOSY-NMR calibration plots for various anilinium salts.

The calculated molecular weights of each anilinium species is consistent with no high-order aggregates being present in observable concentrations, on the NMR timescale.

14. Probing Iodide displacement of trimethylamine

To probe the possibility of an S_NAr type displacement of trimethylamine by iodide, **3a** (0.103 M) was heated in DMSO-d_6 at 120 °C for 20 min in the presence of 4-I-benzaldehyde (0.011 M) and 1,2,4,5-tetramethylbenzene (0.06 M) as an internal standard. The concentration of each species was measured at the start and end of the heating period to see if there was any increase in the concentration of 4-I-benzaldehyde, which would be the displacement product (**table S8**). The concentration of 4-I-benzaldehyde was shown not to increase in this experiment, ruling out this reactive pathway.

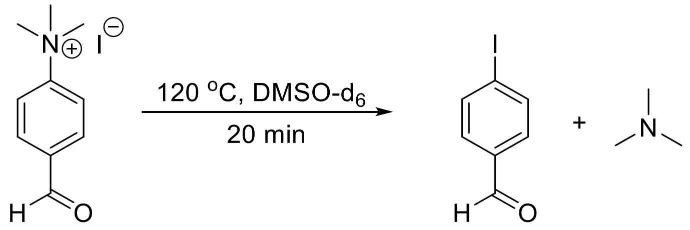

Analyte	Time (min)	Average Concentration (M)	Concentration change (%)
**3a**	0	0.103	-89.4 ± 0.8
	20	0.011	
**4-I-benzaldehyde**	0	0.011	-0.3 ± 0.3
	20	0.011	

Table S8. Reaction probing the possibility of trimethylamine displacement in the degradation of **176a**.

15. Effect of UV radiation on the degradation of 4-formyl-*N,N,N*-trimethylanilinium iodide

4-formyl-*N,N,N*-trimethylanilinium iodide (**3a**) (10.5 mg, 36 µmol) and 1,2,4,5-tetramethylbenzene internal standard (4.8 mg, 36 µmol) was dissolved in DMSO-d_6 (0.6 mL, 0.7140 g), to give an anilinium, and internal standard concentration each of 0.06 M. The solution was transferred to an NMR tube and a ^{1}H NMR spectrum was recorded. The solution was heated to 80 °C for 1 h either in darkness, or exposed to UV and visible radiation, then the NMR tube was placed in ice to halt the degradation. A ^{1}H NMR spectrum was recorded of the degradation mixture.

The concentration of the anilinium salt was calculated *via* the integration of its aromatic protons against those of the internal standard. Each experiment was performed in triplicate. The average %degradation value for each of the salts is shown in **table S9**.

Light conditions	Anilinium salt	Degradation (%)
Darkness	**3a**	18.8 ± 1.5
UV lamp	**3a**	20.6 ± 1.0

Table S9. %Degradation of **3a** upon heating in DMSO-d_6 at 80 °C for 1 h in either darkness, or exposed to UV radiation.

16. Effect of concentration on the degradation of 4-formyl-*N,N,N*-trimethylanilinium iodide

4-formyl-*N,N,N*-trimethylanilinium iodide (**3a**) and 1,2,4,5-tetramethylbenzene internal standard (4.8 mg, 36 µmol) were dissolved in DMSO-d_6 (0.6 mL, 0.7140 g), to give an anilinium concentration of 0.025, 0.05, 0.10, 0.15, 0.20 or 0.25 M, and an internal standard concentration of 0.06 M, then transferred to an NMR tube. A ^{1}H NMR spectrum (4 scans, 25 s relaxation time) was recorded every 5 min whilst the sample was heated to 80 °C in the NMR spectrometer probe.

The concentration of the anilinium salt and aniline were calculated *via* their aromatic protons at each time point against the internal standard. The individual graphs for each of the experiments are shown in **figure S20a-d**. The anilinium degradation for each of the experiments in terms of fraction degradation is plotted on one graph for comparison in **figure S21**.

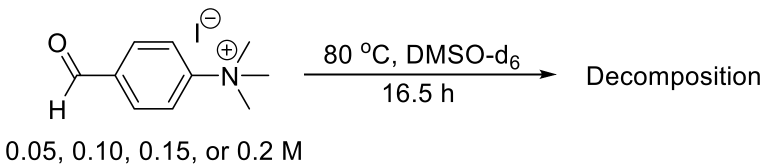

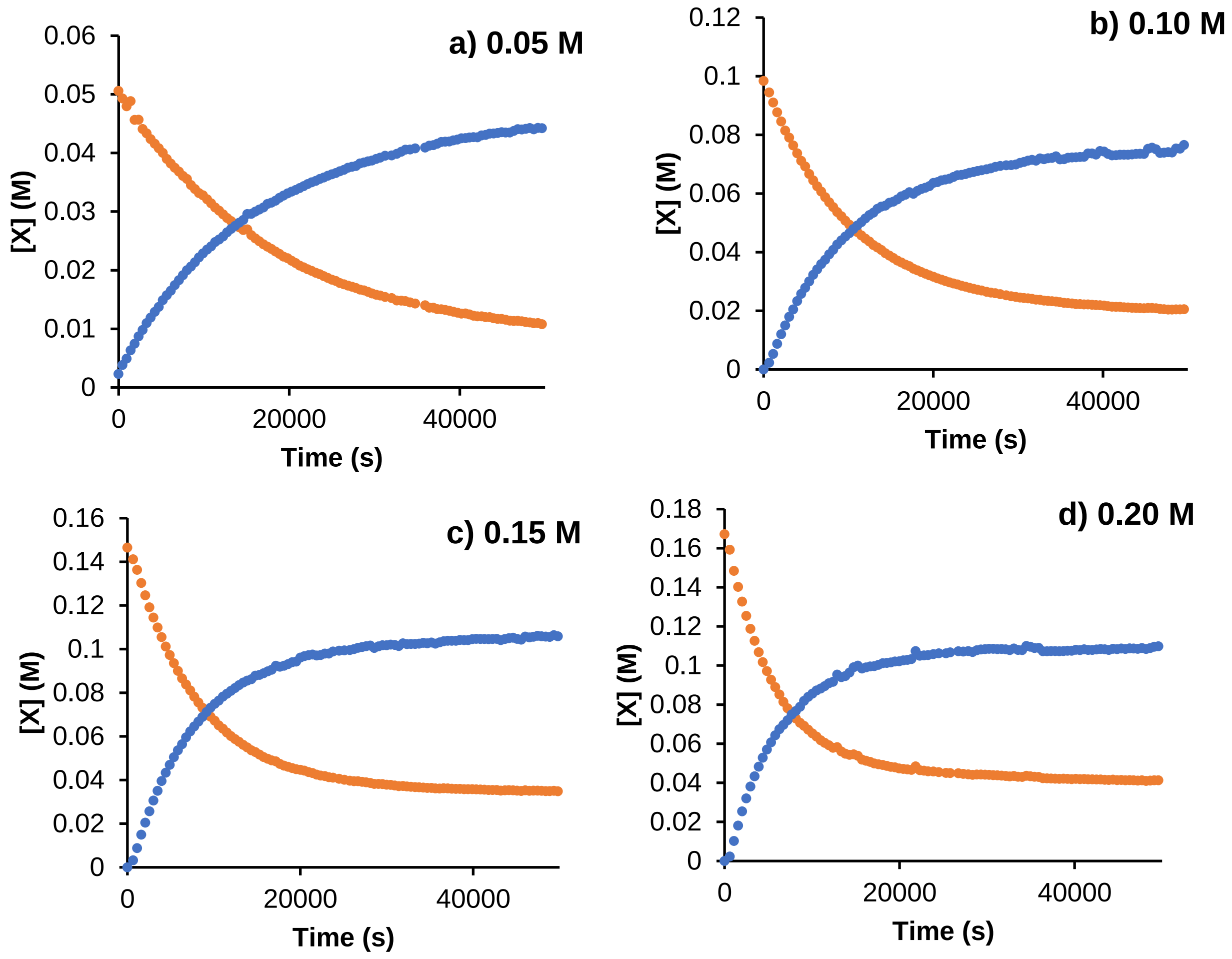

Figure S20. Degradation of **3a** and evolution of 4-formy-*N*,*N*-dimethylaniline determined by ^{1}H NMR monitoring at 80 °C with initial [**3a**] of a) 0.05M b) 0.10 M, c) 0.15M, d) 0.20 M.

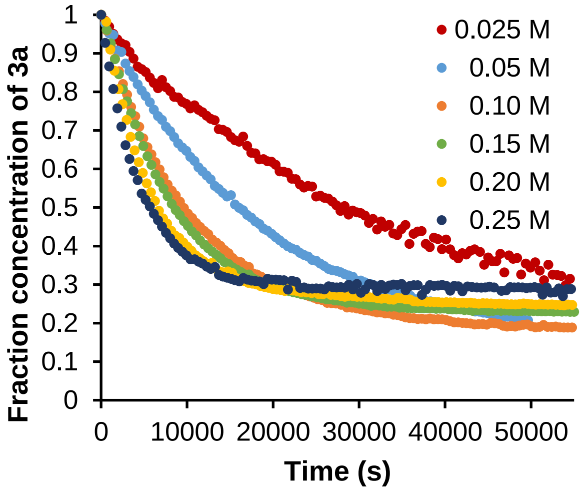

Figure S21. Fraction concentration of **3a** over time at initial [**3a**] of 0.05 M, 0.10 M, 0.15 M, 0.20 M.

The initial rate of degradation was calculated from this data at each concentration and a plot of initial anilinium concentration vs initial degradation rate was constructed (**figure S22** and **table S10**). The degradation rate increases with an increase in initial concentration though the relationship is non-linear.

$[\mathbf{3a}]_0$ (M)	Initial degradation rate (μMs^{-1})
0.025	0.73
0.05	1.96
0.10	5.58
0.15	9.54
0.20	15.3
0.25	25.5

Table S10. Degradation rate of **3a** determined at different values of $[\mathbf{3a}]_0$.

Using the Solver routine in Microsoft Excel, the experimental data were fitted to three initial mechanistic models for anilinium salt degradation:

a. First order: Rate = $k_1[\mathbf{3a}]_o$
b. Second order: Rate = $k_2[\mathbf{3a}]_o^2$
c. Mixed First and Second Order: Rate = $k_1[\mathbf{3a}]_o + k_2[\mathbf{3a}]_o^2$

Figure S22 shows the real data coplotted with all three regression-modelled simulations. Using Solver in Excel, the values of k_1 and/or k_2 were varied in order to minimise the sum of squares (and therefore root mean squared) error between the real and simulated absolute rate values.

Whilst first order is insufficient to explain all the observations, both second and mixed order models evidence lower root mean square errors (RMSEs) than the first order model. This analysis is consistent with the log-log plot which suggested a reaction order of approximately 1.7 in anilinium iodide salt concentration, $[\mathbf{3a}]_0$ (**figure S23**).

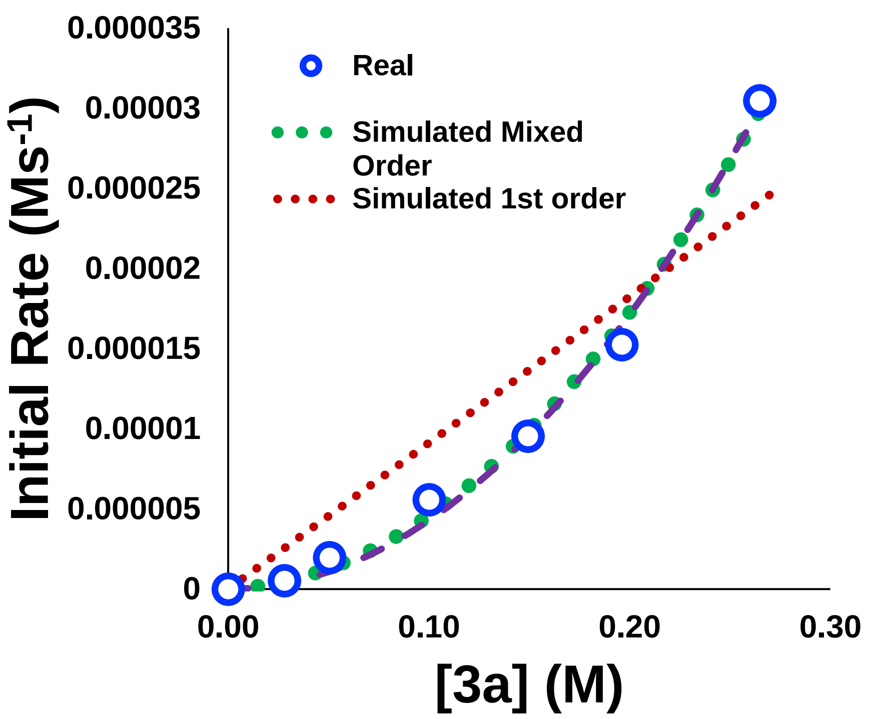

Figure S22. Degradation rate of **3a** determined via degradation monitoring (blue) at different values of [**3a**]$_0$.

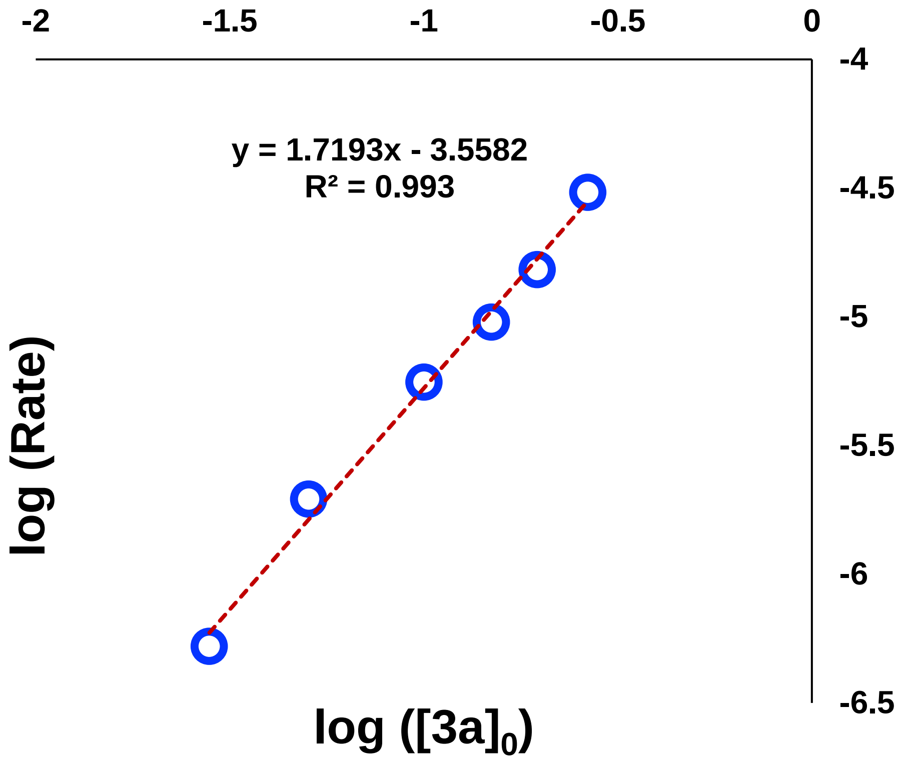

Figure S23. Log-log plot reporting the apparent order of reaction in [**3a**]$_0$. Replotted from manuscript Figure 15.

17. In-depth Microkinetic Analysis Using COPASI

Following on from the initial mechanistic hypothesis and microkinetic analysis presented Supporting Information **Section 11**, comparative analysis of 4 further mechanistic models were analysed using COPASI. This was done to enable a more wholistic mechanistic assessment alongside 4 parallel computational (DFT) mechanisms considered (see separate Computational Chemistry Supporting Information document).

In summary, the 4 mechanisms modelled in COPASI (below) are labelled as:

1. **First order**: reversible initial unimolecular degradation of anilinium iodide followed by reversible capture of methyl iodide by DMSO solvent.
2. **True Second Order**: reversible initial bimolecular degradation of anilinium iodide followed by reversible capture of methyl iodide by DMSO solvent.
3. **Pseudo Second Order**: reversible initial bimolecular degradation of anilinium iodide, wherein only one ion pair is degraded, followed by reversible capture of methyl iodide by DMSO solvent.
4. **First and True Second Order Mix**: mechanisms 1 and 2 (above) operate simultaneously.

In each case, all available experimental ^{1}H NMR kinetic data were used as a combined input.

Mechanism	Objective Function Value	Standard Deviation	R^2 (Experimental Absolute Rate vs k_1)
**1**	0.002794	0.001131	0.9666
**2**	0.002017	0.000962	0.9428
**3**	0.002356	0.001039	0.0738
**4**	0.00217317	0.00100004	0.0694 and 0.5603

Table S11. Summarised COPASI metrics and correlation of predicted k_1 rate constants for each mechanism versus observed absolute initial rate.

Mechanism 1 - First Order

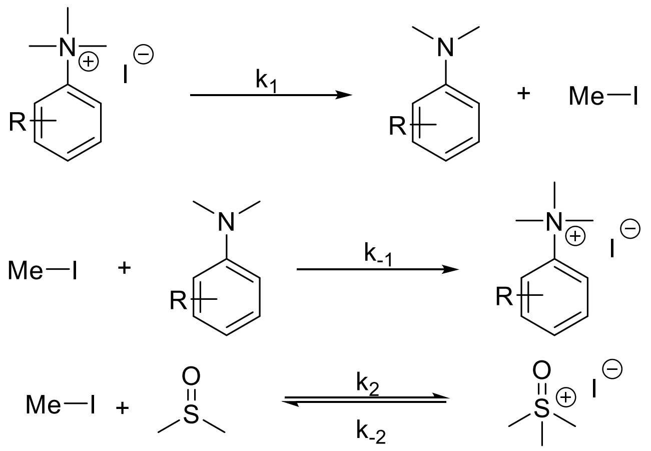

Objective Function Value:	0.002794
Standard Deviation:	0.001131

Salt	k_1	k_{-1}	k_2	k_{-2}
**4-F**	5.87E-07	4.12E-09	1.00E-05	3.00E-04
**3-OMe**	1.18E-06	5.46E-06	1.00E-05	3.00E-04
**TMAI**	1.19E-06	1.07E-09	1.00E-05	3.00E-04
**2-Me**	1.81E-05	6.64E-04	1.00E-05	3.00E-04
**3-Cl**	2.03E-05	3.09E-03	1.00E-05	3.00E-04
**3-Br**	2.47E-05	3.86E-03	1.00E-05	3.00E-04
**2-Pyr**	3.99E-05	1.19E-03	1.00E-05	3.00E-04
**4-CF_3**	6.18E-05	1.00E-03	1.00E-05	3.00E-04
**4-Bz**	6.22E-05	9.85E-04	1.00E-05	3.00E-04
**4-CHO**	7.18E-05	3.35E-04	1.00E-05	3.00E-04

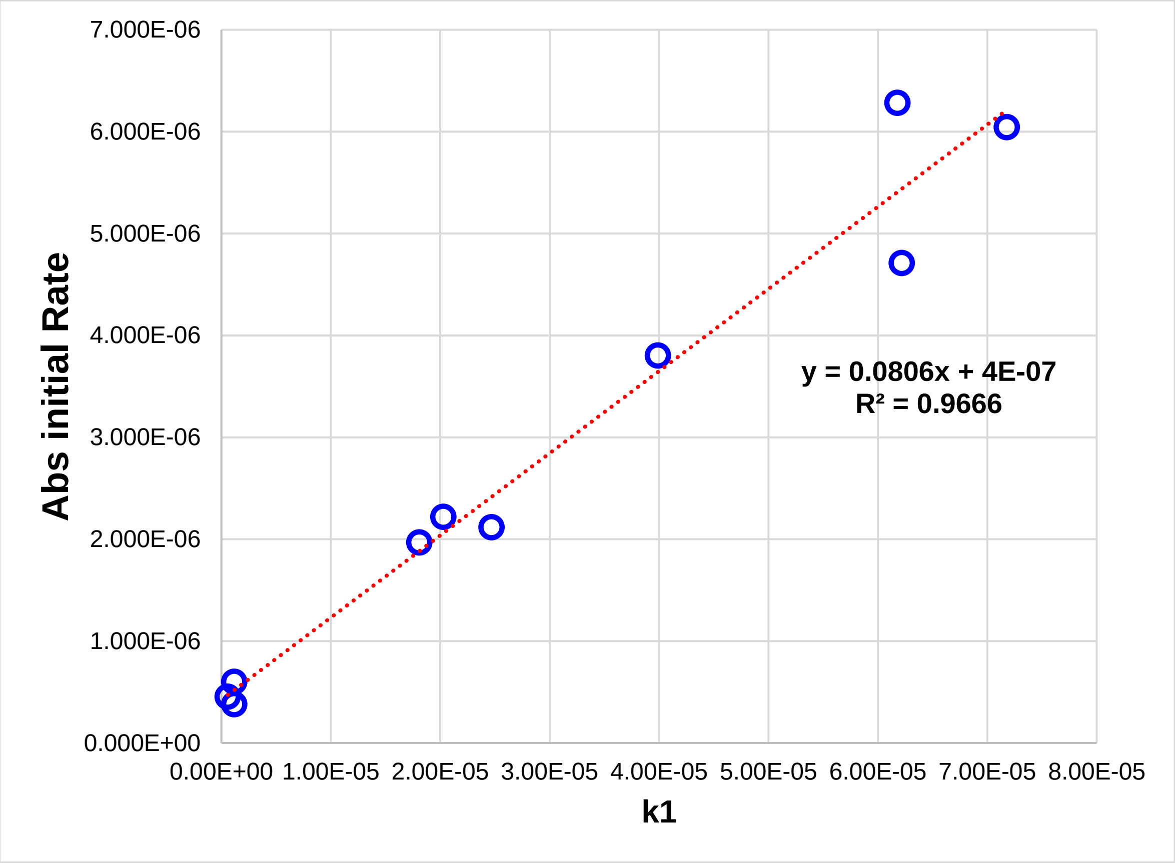

Mechanism 2 - True Second Order

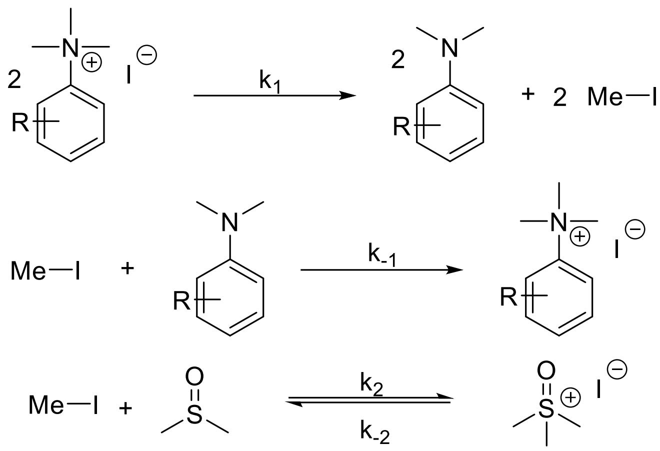

Objective Function Value:	0.002017
Standard Deviation:	0.000962

**Salt**	**k_1**	**k_{-1}**	**k_2**	**k_{-2}**
**4-F**	2.94E-06	2.26E-05	1.05E-05	2.47E-04
**3-OMe**	5.11E-06	1.39E-07	1.05E-05	2.47E-04
**TMAI**	6.59E-06	3.91E-06	1.05E-05	2.47E-04
**2-Me**	1.03E-04	4.19E-04	1.05E-05	2.47E-04
**3-Cl**	1.24E-04	3.01E-03	1.05E-05	2.47E-04
**3-Br**	1.47E-04	3.80E-03	1.05E-05	2.47E-04
**2-Pyr**	2.45E-04	9.81E-04	1.05E-05	2.47E-04
**4-CF_3**	3.64E-04	4.39E-04	1.05E-05	2.47E-04
**4-Bz**	3.88E-04	7.06E-04	1.05E-05	2.47E-04
**4-CHO**	5.50E-04	4.21E-05	1.05E-05	2.47E-04

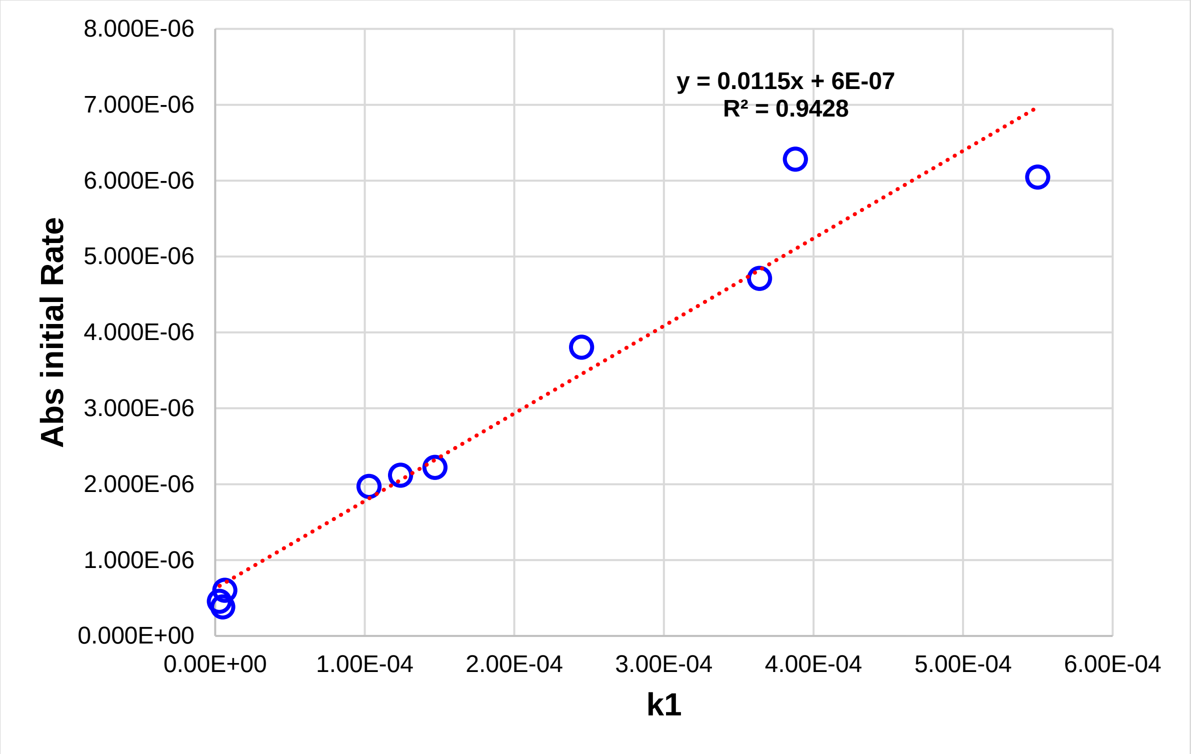

Mechanism 3 - Pseudo Second order

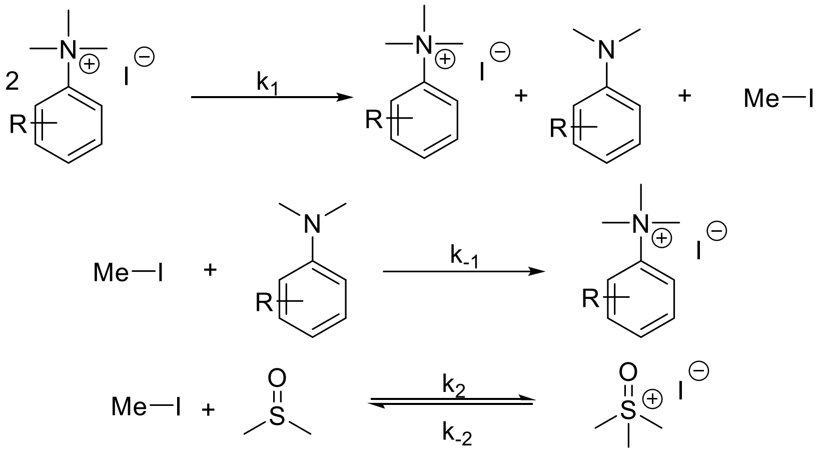

Objective Function Value:	0.002356
Standard Deviation:	0.001039

Salt	k_1	k_{-1}	k_2	k_{-2}
**4-CHO**	6.24E-06	8.51E-07	1.12E-05	3.00E-04
**4-F**	9.78E-06	8.34E-04	1.12E-05	3.00E-04
**3-Cl**	1.03E-05	2.93E-03	1.12E-05	3.00E-04
**TMAI**	1.94E-04	4.78E-05	1.12E-05	3.00E-04
**2-Pyr**	2.46E-04	1.15E-03	1.12E-05	3.00E-04
**3-Br**	2.63E-04	2.69E-03	1.12E-05	3.00E-04
**2-Me**	5.19E-04	3.65E-04	1.12E-05	3.00E-04
**4-Bz**	7.41E-04	5.04E-04	1.12E-05	3.00E-04

**3-OMe**	7.86E-04	4.83E-06	1.12E-05	3.00E-04
**4-CF$_3$**	1.00E-03	5.99E-04	1.12E-05	3.00E-04

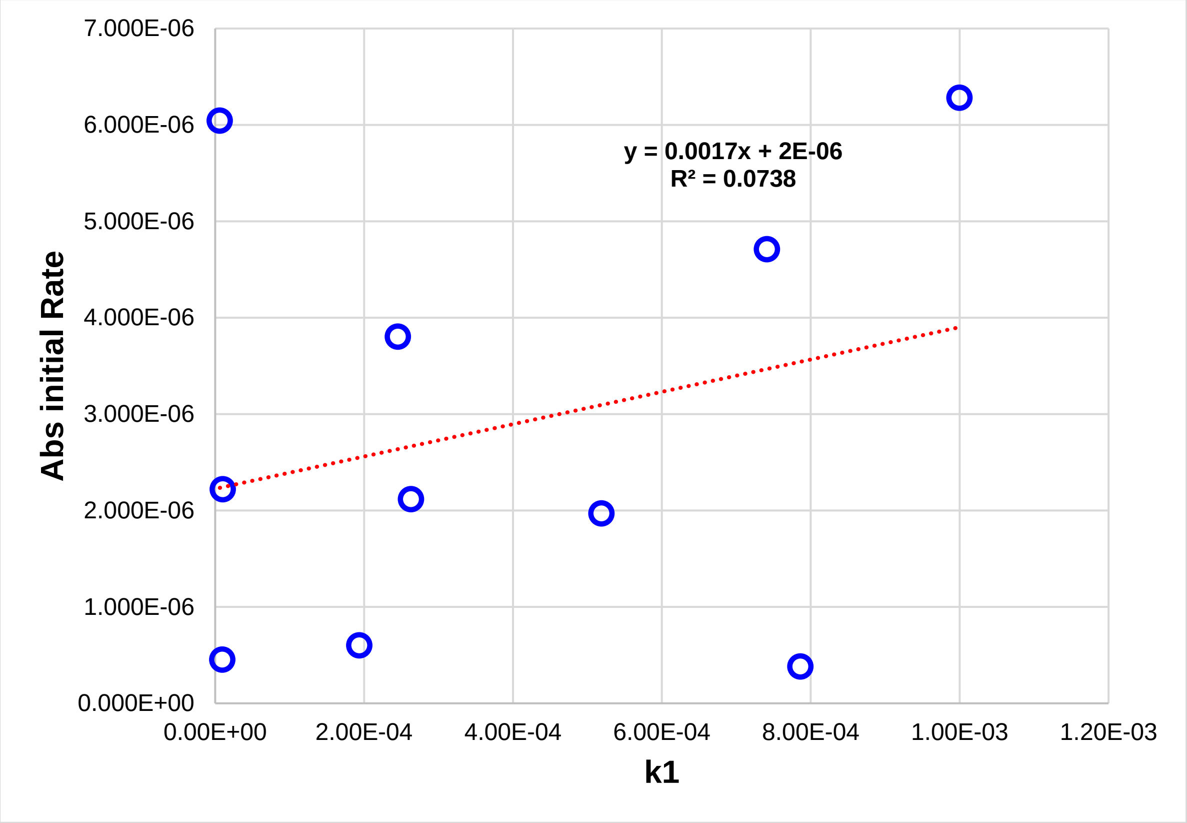

Mechanism 4 - First and True Second Order Mix

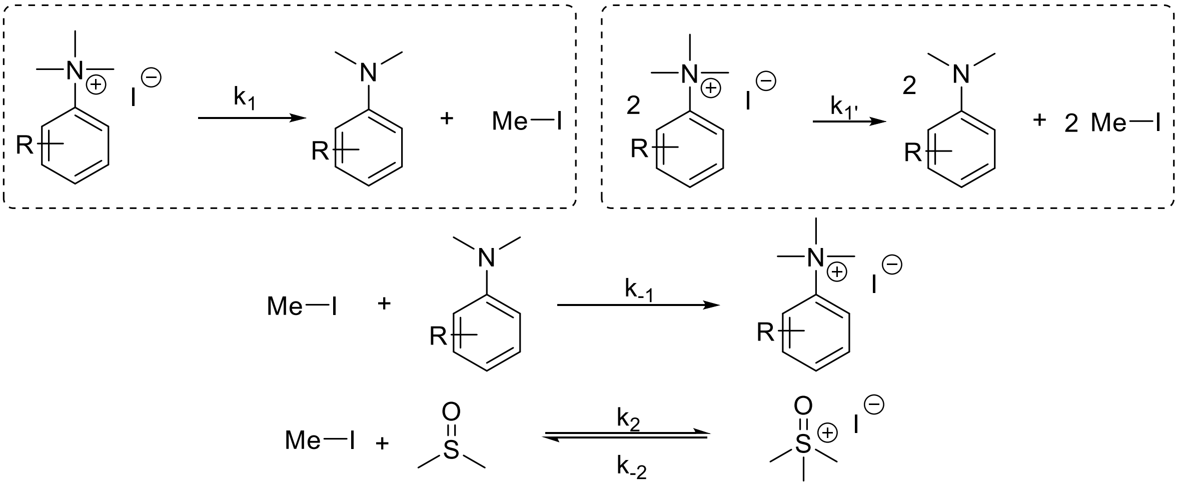

Objective Function Value:	0.00217317
Standard Deviation:	0.00100004

Salt	k_1	k_1'	k_{-1}	k_2	k_{-2}
**4-CF$_3$**	1.23E-09	3.95E-04	7.11E-04	1.13E-05	2.54E-04
**2-Me**	1.36E-08	9.59E-05	3.10E-04	1.13E-05	2.54E-04
**TMAI**	3.12E-07	4.46E-06	3.01E-08	1.13E-05	2.54E-04
**4-F**	5.18E-07	1.42E-09	2.22E-08	1.13E-05	2.54E-04
**4-CHO**	1.00E-06	5.60E-04	5.56E-05	1.13E-05	2.54E-04
**3-OMe**	1.04E-06	5.46E-07	1.04E-04	1.13E-05	2.54E-04
**3-Br**	2.10E-05	8.00E-07	3.67E-03	1.13E-05	2.54E-04
**3-Cl**	2.43E-05	8.05E-07	4.74E-03	1.13E-05	2.54E-04
**2-Pyr**	4.98E-05	1.04E-08	2.66E-03	1.13E-05	2.54E-04
**4-Bz**	5.24E-05	4.26E-07	9.31E-04	1.13E-05	2.54E-04

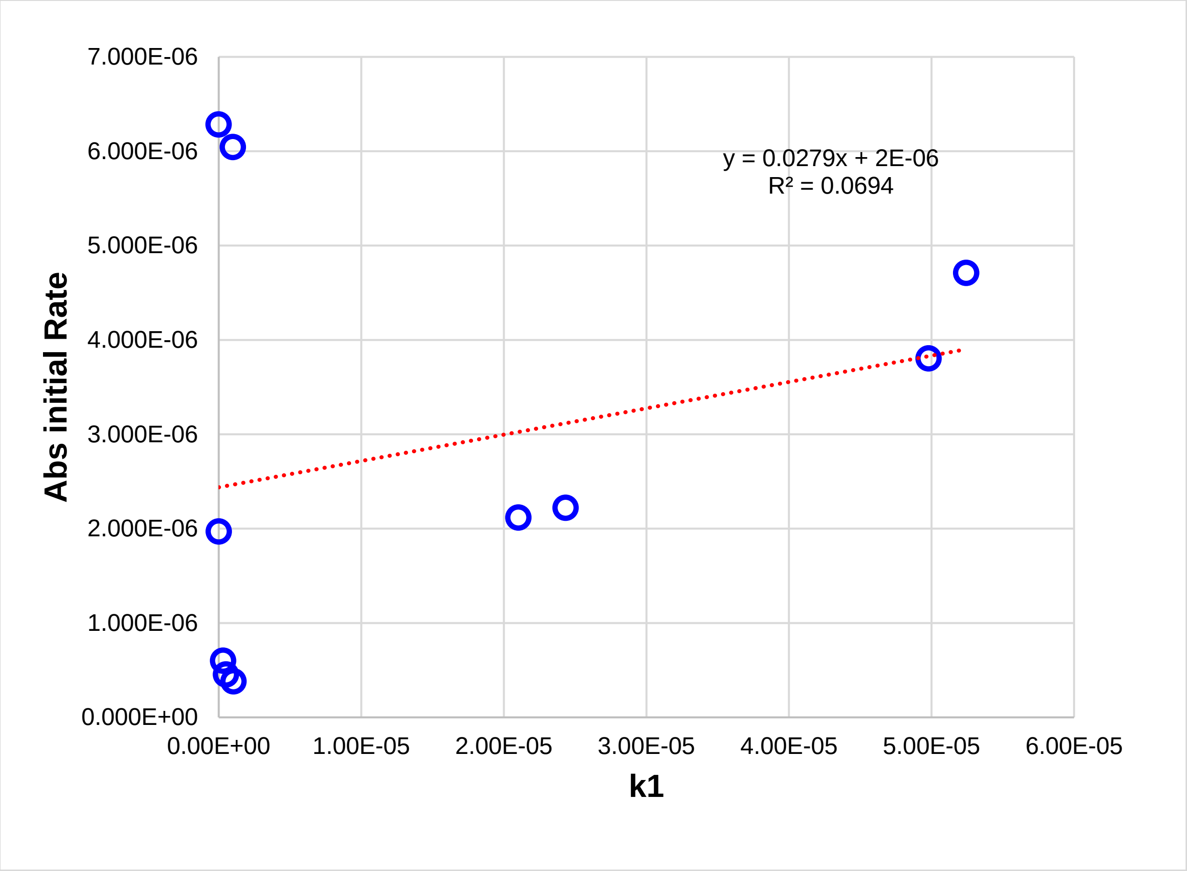

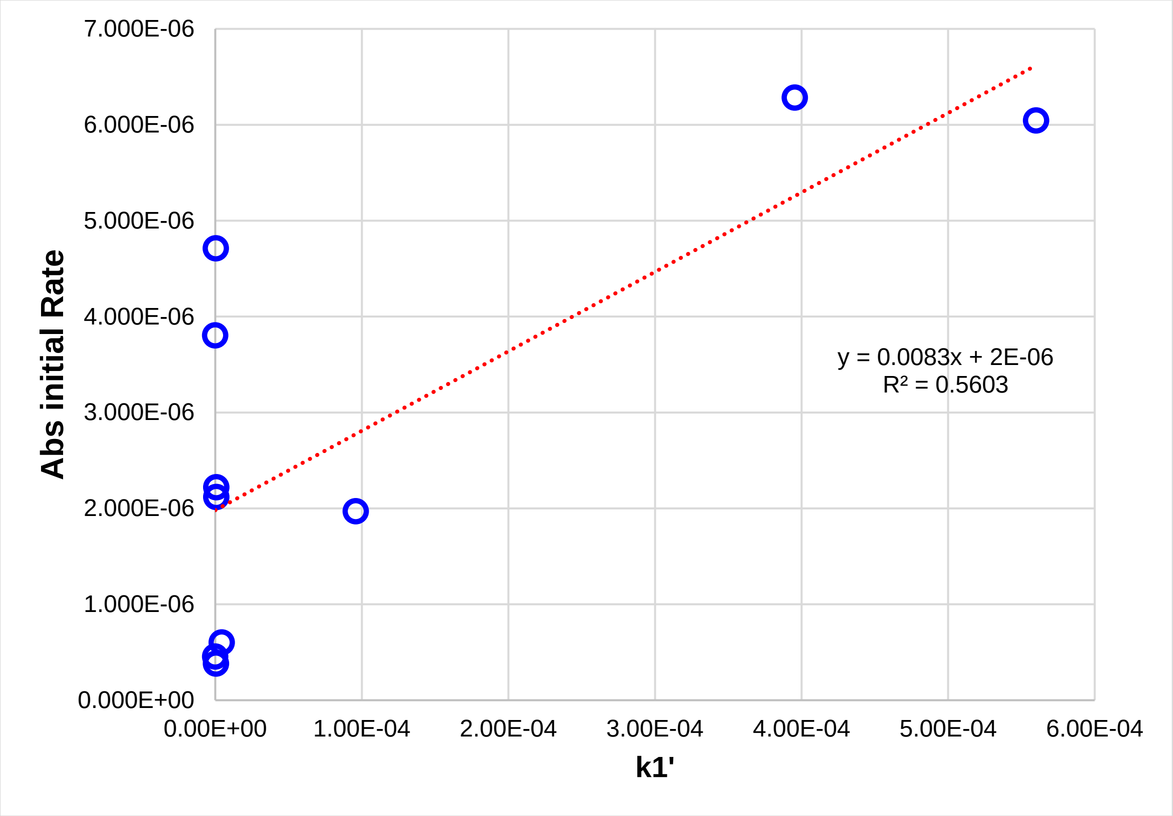

3a Concentration Data

First Order

Objective Function Value:	0.002038
Standard Deviation:	0.001199

[3a]	k_1	k_1'	k_{-1}	k_2
**0.025**	2.71E-05	2.30E-06	1.21E-05	2.27E-04
**0.05**	4.19E-05	9.35E-06	1.21E-05	2.27E-04
**0.1**	7.44E-05	4.05E-04	1.21E-05	2.27E-04
**0.15**	8.23E-05	3.90E-04	1.21E-05	2.27E-04
**0.2**	1.19E-04	5.81E-04	1.21E-05	2.27E-04
**0.25**	1.46E-04	5.30E-04	1.21E-05	2.27E-04

True Second Order

Objective Function Value:	0.002867
Standard Deviation:	0.001421

[3a]	k_1	k_1'	k_{-1}	k_2
**0.025**	6.01E-04	8.01E-06	1.14E-05	2.55E-04
**0.05**	7.57E-04	1.25E-06	1.14E-05	2.55E-04
**0.1**	5.50E-04	2.05E-05	1.14E-05	2.55E-04

**0.15**	4.29E-04	1.08E-04	1.14E-05	2.55E-04
**0.2**	5.08E-04	1.57E-04	1.14E-05	2.55E-04
**0.25**	3.64E-04	1.93E-04	1.14E-05	2.55E-04

First and True Second Mix

**[3a]**	**k_1**	**k_1'**	**k_{-1}**	**k_2**	**k_{-2}**
**0.025**	8.50E-06	3.52E-04	1.06E-06	1.12E-05	2.99E-04
**0.05**	4.68E-05	7.04E-06	3.00E-04	1.12E-05	2.99E-04
**0.1**	1.20E-06	5.40E-04	3.35E-06	1.12E-05	2.99E-04
**0.15**	6.35E-05	1.16E-04	2.97E-04	1.12E-05	2.99E-04
**0.2**	4.00E-05	3.39E-04	2.82E-04	1.12E-05	2.99E-04
**0.25**	6.42E-05	1.98E-04	3.05E-04	1.12E-05	2.99E-04

18. Effect of Temperature on the Degradation of 4-formyl-*N,N,N*-trimethylanilinium iodide

4-formyl-*N,N,N*-trimethylanilinium iodide, **3a** (17.5 mg, 60 µmol) and 1,2,4,5-tetramethylbenzene internal standard (4.8 mg, 36 µmol) were dissolved in DMSO-d_6 (0.6 mL, 0.7140 g), to give an anilinium concentration of 0.10 M, and an internal standard concentration of 0.06 M, then transferred to an NMR tube. A ^{1}H NMR spectrum (4 scans, 25 s relaxation time) every 5 min whilst the sample was heated to either 50, 60, 70, 75, or 80 °C in the NMR spectrometer probe.

The concentration of the anilinium salt and aniline were calculated *via* their aromatic protons at each time point against the internal standard. The individual graphs for each of the experiment are shown in **figure S24a-e**. Rate constants for each step of the degradation pathway were estimated and are shown in **table S12**.

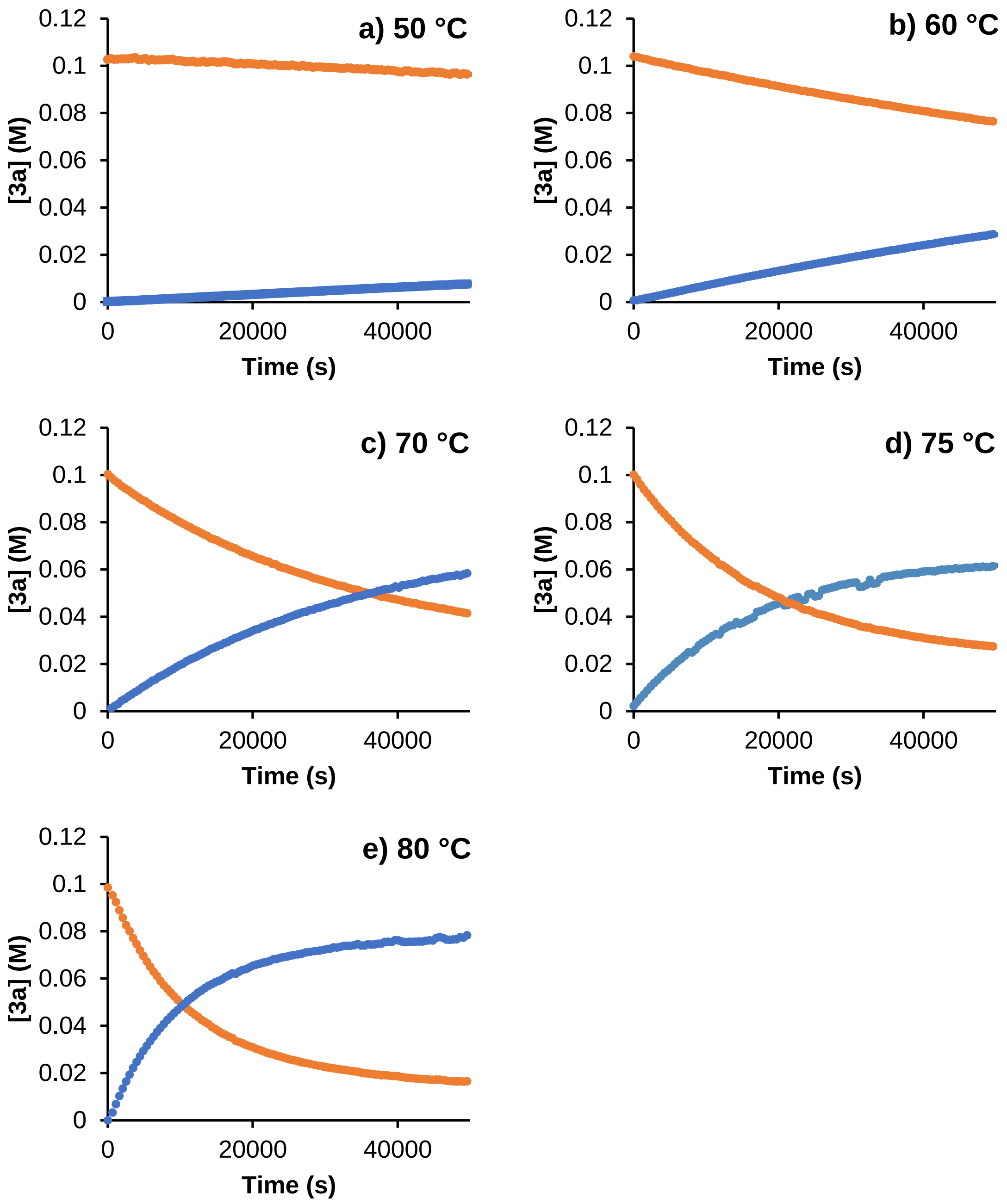

Figure S24. Degradation of **3a** and evolution of 4-formyl-N,N-dimethylaniline determined *via* ^{1}H NMR monitoring in DMSO-d_6 at 50, 60, 70, 75, and 80 °C.

Temperature (^oC)	Initial Rate (Ms^{-1})	k (s^{-1})	Temperature (K)	1/T (K^{-1})	ln(kh/k_bT)
50	0.000000129	1.17E-06	323	0.003096	-43.19547975
60	0.000000673	6.28E-06	333	0.003003	-41.54686697
70	0.000002107	2.21E-05	343	0.002915	-40.31622986
75	0.000003600	4.06E-05	348	0.002874	-39.72396661
80	0.000006224	7.42E-05	353	0.002833	-39.13503788

Table S12. First order rate constants determined for degradation of **3a** at 50, 60, 70, 75, and 80 °C.

A linear form of the Eyring equation was plotted to calculate values for ΔH‡ and ΔS‡ (**figure S23**). Linear regression analysis revealed a gradient of -15273 ± 480 K, and y-intercept of 4.18 ± 1.41. Using these values, the calculated activation parameters for the degradation were ΔH‡ = +127.0 ± 4.0 $kJmol^{-1}$ and ΔS‡ = +34.8 ± 11.7 $Jmol^{-1}K^{-1}$.

$$\ln\left(\frac{kh}{k_bT}\right) = \left(\frac{-\Delta H^{\ddagger}}{R}\right)\frac{1}{T} + \frac{\Delta S^{\ddagger}}{R}$$

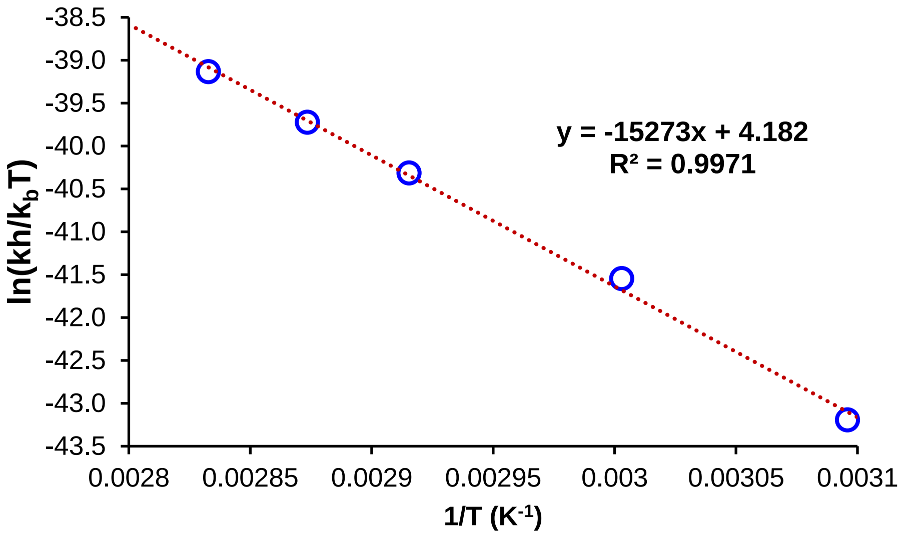

Figure S25. Eyring plot produced with k_1 value calculated for the degradation of **3a** between 50-80 °C.

To cover any potential errors of interpretation in the Eyring analysis, a linear form of the Eyring equation was plotted to calculate values for ΔH‡ and ΔS‡ (**figure SX**) using derived second order rather than derived first order rate constants (**table SX**). Linear regression analysis revealed calculated activation parameters for the degradation were ΔH‡ = +139.8 ± 3.2 $kJmol^{-1}$ and ΔS‡ = +87.5 ± 9.3 $Jmol^{-1}K^{-1}$.

In both cases, the magnitude and direction of both activation parameters remained the same. Most dioagnostically, ΔS‡ was positive (and of similar magnitude) using either first or second

order rate constants. Both Eyring treatments remain consistent with a combination of first and second order processes occurring. The data also remain consistent with the solvation disorder argument depicted in **Figure 16** and surrounding text in the manuscript.

Temperature	Initial Rate (Ms^{-1})	k (M^{-1} s^{-1})	Temperature (K)	1/T (K^{-1})	ln(k_h/k_bT)
50	0.000000129	5.81E-06	323	0.003096	-41.595
60	0.000000673	3.40E-05	333	0.003003	-39.8576
70	0.000002107	1.36E-04	343	0.002915	-38.499
75	0.000003600	2.65E-04	348	0.002874	-37.8465
80	0.000006224	5.68E-04	353	0.002833	-37.0995

Table S13. Second order rate constants and thermal data determined for degradation of **3a** at 50, 60, 70, 75, and 80 °C.

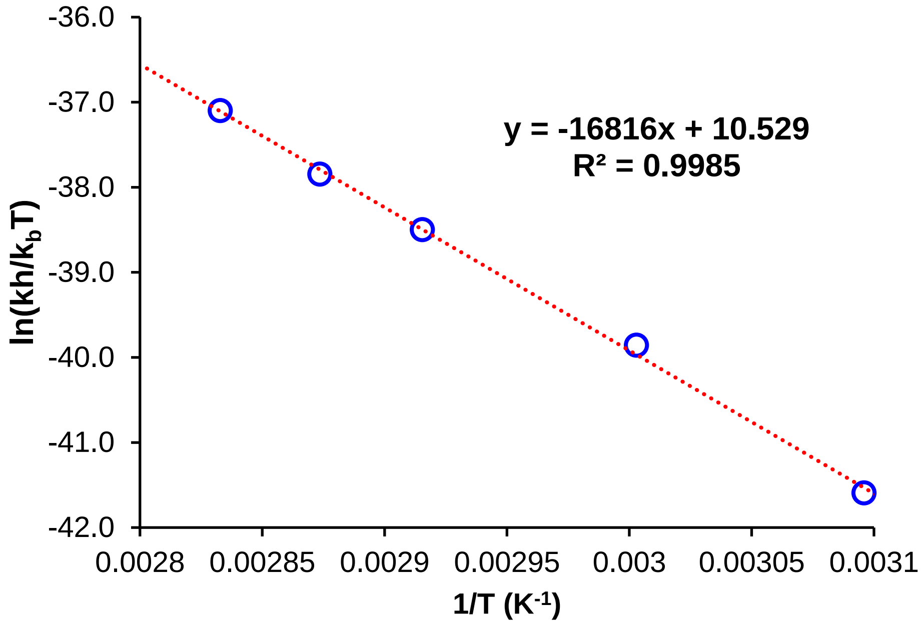

Figure S25. Eyring plot produced with using second order rate constants calculated for the degradation of **3a** between 50-80 °C.

19. Kinetic isotope Effect Studies

In separate NMR tubes, for independent rate experiments, 0.1 M of **11a** and **11a-d9** were prepared in dry DMSO-d9. A ^{1}H NMR spectrum (4 scans, 25 s relaxation time) was recorded every 5 min whilst the sample was heated to 80 °C in the NMR spectrometer probe.

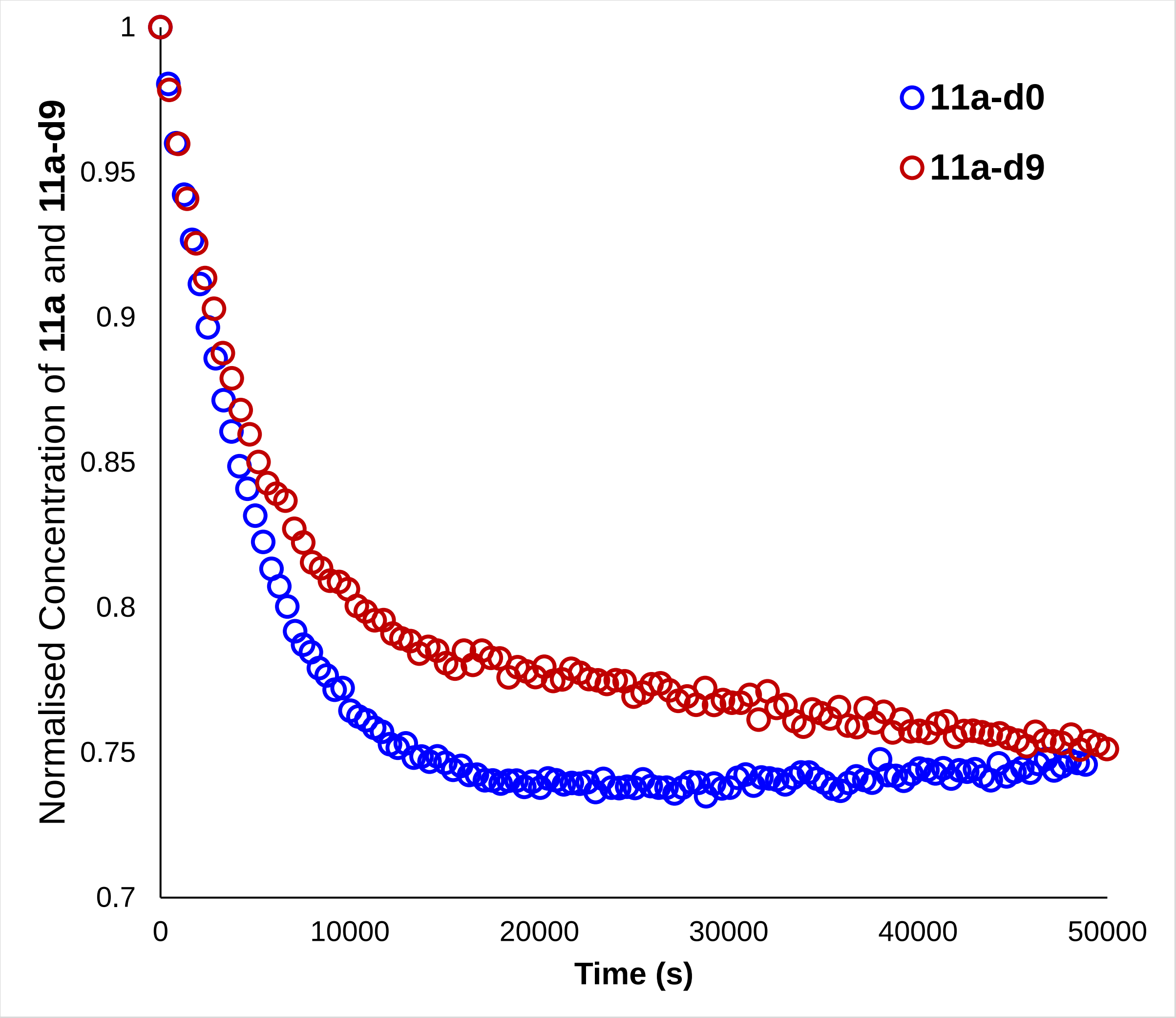

Figure S26. Independent rate experiments for **11a** and **11a-d9** at 0.1 M in dry DMSO-d6.

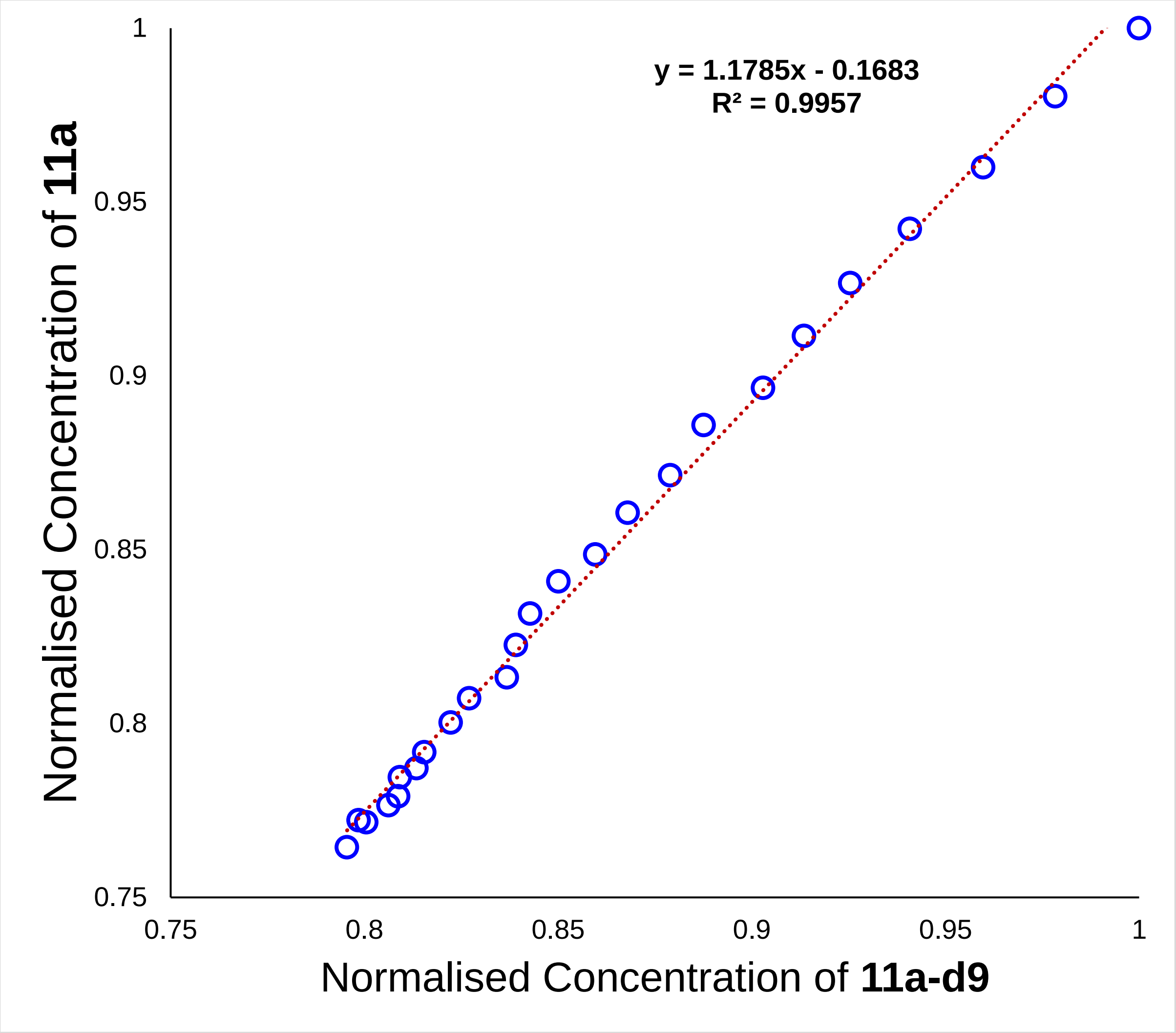

Figure S27. Estimated H/D kinetic isotope effect in the degradation of N,N,N-3-bromo-anilinium iodide.

20. Methylation of 4-^{t}Bu-phenol using *N,N,N*-trimethylanilinium salts

Methylation in Toluene

4-^{t}Bu-phenol (0.300 g, 2 mmol, 1 eq.), the relevant *N,N,N*-trimethylanilinium salt (2.4 mmol, 1.2 eq.), potassium carbonate (1.106 g, 8 mmol, 4 eq.), and Toluene (3 mL) were added to an Asynt DrySyn Octo reaction carousel tube equipped with a stirrer bar. The reaction mixture was heated to 80 °C with stirring for 3 h. After cooling, an aliquot of the supernatant was taken and the conversion of 4-^{t}Bu-phenol to 4-^{t}Bu-anisole was calculated *via* ^{1}H NMR analysis of the crude reaction mixture. Each experiment was conducted in triplicate. A range of *N,N,N*-trimethylanilinium salts were all screened for their ability to methylate 4-^{t}Bu-phenol in this manner. A chart showing the average conversion to the anisole product of each anilinium salt tested is shown below. A table showing the values for each of the three runs, the average and the error is also provided.

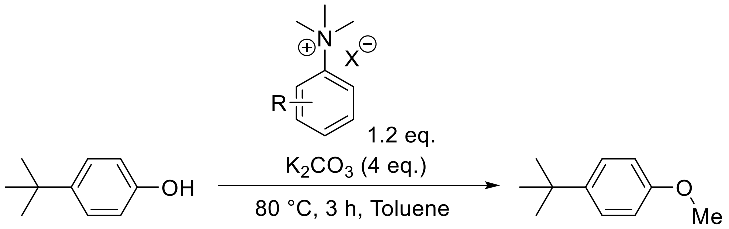

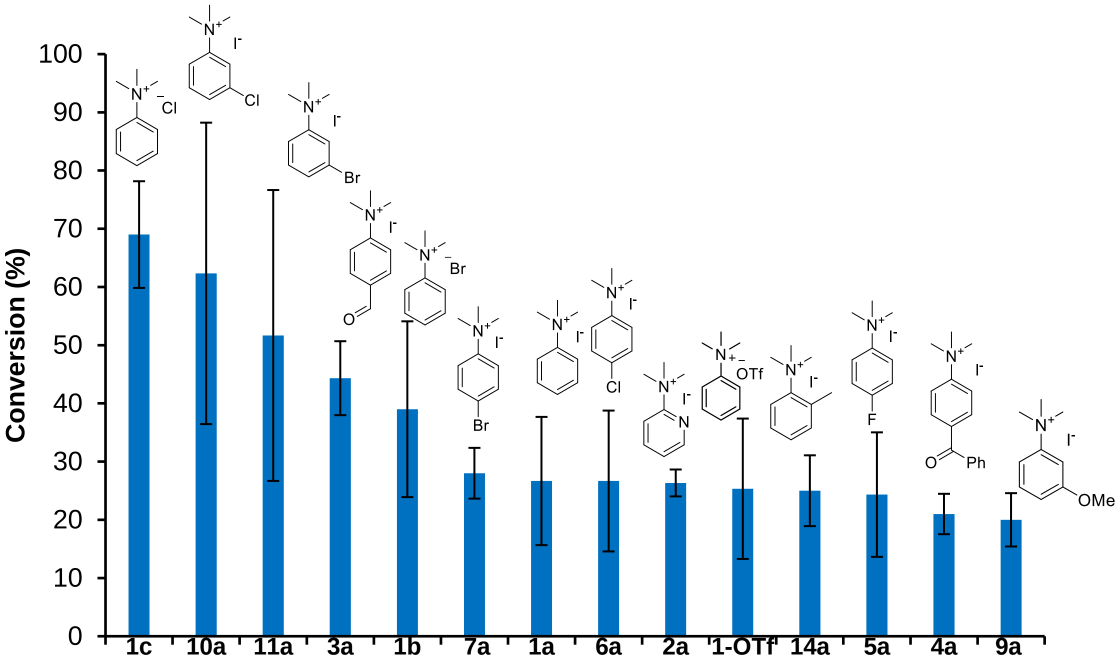

Figure S28. Conversion of **16** to **17** in toluene with a range of substituted *N,N,N*-trimethylanilinium salts (1.2 equiv.) and potassium carbonate (4 equiv.).

Anilinium salt	Conversion (%)			Average Conversion (%)	Error (%)
	Run 1	Run 2	Run 3		
**1a**	16	26	38	26.67	11.02
**1b**	53	41	23	39.00	15.10
**1c**	61	67	79	69.00	9.17
**1-OTf**	38	24	14	25.33	12.06
**2a**	25	25	29	26.33	2.31
**3a**	37	48	48	44.33	6.35
**4a**	19	25	19	21.00	3.46
**5a**	31	30	12	24.33	10.69
**6a**	36	31	13	26.67	12.10
**7a**	33	26	25	28.00	4.36
**9a**	25	16	19	20.00	4.58
**10a**	72	82	33	62.33	25.89
**11a**	79	30	46	51.67	24.99
**14a**	22	32	21	25.00	6.08

Table S14. Conversion of **16** to **17** in toluene with a range of substituted *N,N,N*-trimethylanilinium salts (1.2 equiv.) and potassium carbonate (4 equiv.).

An investigation into the effect of temperature on the ability of a selection of *N,N,N*-trimethylanilinium salts to methylate 4-^{t}Bu-phenol in toluene was then investigated. The experimental procedure was identical to the procedure mentioned above, with the exception that the reaction was carried out at either 30 or 50 °C. Anilinium salts **1a**, **1c**, **3a**, and **14a** were screened for their methylating ability at these temperatures. A graph showing the conversion to the anisole product for each salt at each temperature is shown below. A table containing the conversion to anisole at 30 and 50 °C is also provided.

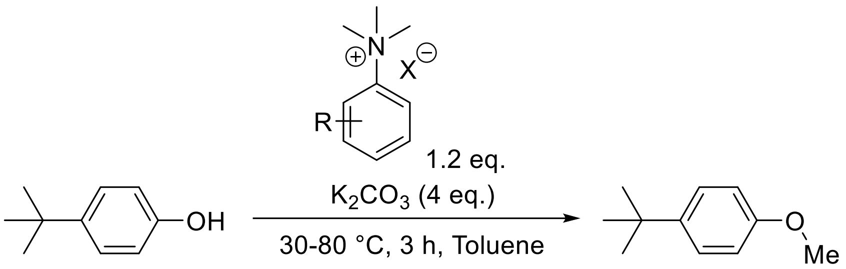

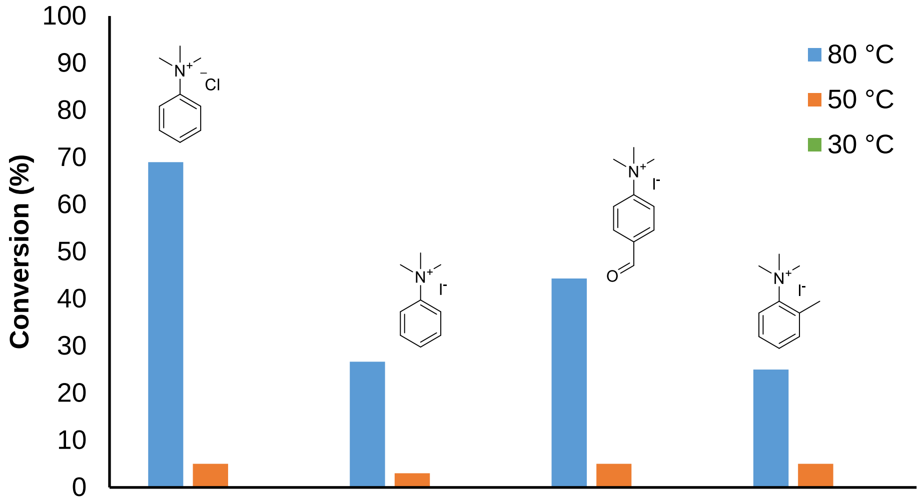

Figure S29. Conversion of **16** to **17** in toluene with a range of substituted *N,N,N*-trimethylanilinium salts (1.2 equiv.) and potassium carbonate (4 equiv.) at various temperatures.

Anilinium salt	Conversion (%)		
	30 °C	50 °C	80 °C
**1a**	0	3	27
**1a**	0	5	69
**3a**	0	5	44
**14a**	0	5	25

Table S15. Conversion of **15** to **16** in toluene with a range of substituted *N,N,N*-trimethylanilinium salts (1.2 equiv.) and potassium carbonate (4 equiv.).

Methylation in DMSO

4-tBu-phenol (0.150 g, 1 mmol, 1 equiv.), the relevant *N,N,N*-trimethylanilinium salt (1.2 mmol, 1.2 equiv.), potassium carbonate (0.553 g, 4 mmol, 4 equiv.), and DMSO (2.5 mL) were added to a 25 mL round bottom flask equipped with a stir bar. The reaction mixture was heated to 80 °C with stirring for 3 h open to air.

After cooling, an aliquot of the supernatant was taken and the conversion of 4-tBu-phenol to 4-tBu-anisole was calculated *via* ^{1}H NMR analysis of the crude reaction mixture. The average

conversion from a triplicate of experiments for a range of *N,N,N*-trimethylanilinium salts is shown in **figure S28** and **table S16**. Compounds **17**, **18**, and **19**, were identified in reaction mixtures using salts **2a**, **3a**, and **4a** arising from S_NAr reactivity, the conversion to these compounds is also shown in **table S16**.

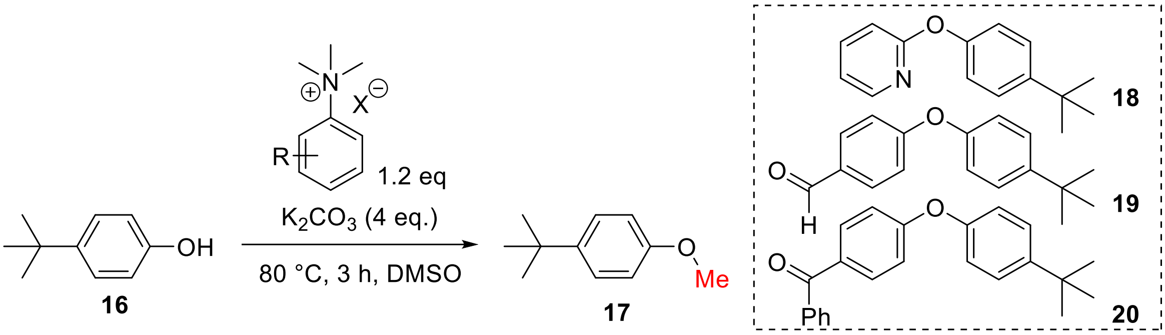

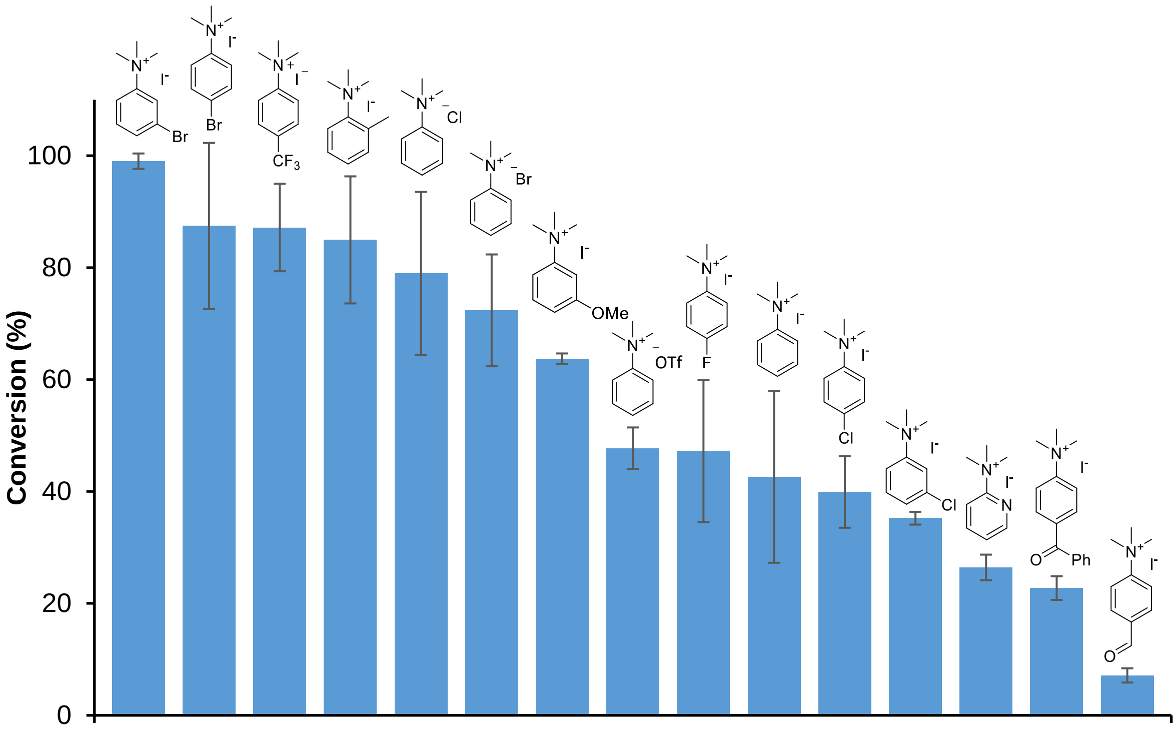

Figure S30. Conversion of **15** to **16** in DMSO with a range of substituted *N,N,N*-trimethylanilinium salts (1.2 equiv.) and potassium carbonate (4 equiv.).

Anilinium salt	4-^{t}Bu-anisole (%)	S_NAr Product (%)
1a	43	0
1b	72	0
1c	79	0
1e	48	0
2a	26	61
3a	7	80
4a	23	61
5a	47	0
6a	40	0
7a	87	0
9a	64	0
10a	35	0
11a	99	0
14a	85	0

Table S16. Conversion of **15** to **16** in DMSO with a range of substituted *N,N,N*-trimethylanilinium salts (1.2 equiv.) and potassium carbonate (4 equiv.).

21. Methylation of phenols in DMSO using 3-Br-*N,N,N*-trimethylanilinium iodide

General procedure F - Methylation of phenols in DMSO using 3-Br-N,N,N-trimethylanilinium iodide

The relevant phenol (1 mmol, 1 equiv.), 3-Br-*N,N,N*-trimethylanilinium iodide (0.4104 g, 1.2 mmol, 1.2 equiv.), potassium carbonate (0.553 g, 4 mmol, 4 equiv.), and DMSO (2 mL) were added to a 25 mL round bottom flask equipped with a stir bar. The reaction mixture was heated to 80 °C with stirring for 3 h open to air. After cooling, the reaction was diluted with water (10 mL) and extracted with dichloromethane (3 x 15 mL). The organic layers were then combined, washed with 1M HCl (3 x 20 mL), dried over $MgSO_4$, and filtered. The solvent was then removed *in vacuo* to give the crude methylated product. Column chromatography on silica gel was used to deliver the anisole product.

*Synthesis of 4-^{t}Bu-anisole **17***

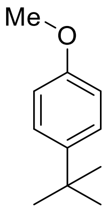

Prepared according to *General Procedure F*

Amount of 4-^{t}Bu-phenol: 0.1502 g, 1.00 mmol

Product yield: 0.1608 g, 0.979 mmol, 98%

IR: ν_{max} 2959, 2361, 1612, 1514, 1464, 1364, 1298, 1246, 1183, 1036, 827, 793 cm^{-1}

^{1}H NMR (400 MHz, $CDCl_3$): δ 1.33 (s, 9H, $C(CH_3)_3$), δ 3.82 (s, 3H, OCH_3), δ 6.87 (m, 2H, ArH), δ 7.33 (m, 2H, ArH)

^{13}C NMR (101 MHz, $CDCl_3$): δ 30.98, 33.54, 54.70, 112.84, 125.71, 142.83, 156.79

Synthesis of 4-I-anisole ***21***

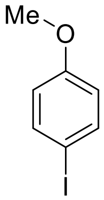

Prepared according to *General Procedure F*

Amount of 4-I-phenol: 0.2340 g, 1.00 mmol

Product yield: 0.1615 g, 0.691 mmol, 69%

Melting point: 47-48 °C

IR: v_{max} 2361, 1584, 1481, 1285, 1238, 1172, 1026, 997, 831, 808, 583 cm^{-1}

^{1}H NMR (400 MHz, $CDCl_3$): δ 3.79 (s, 3H, OCH_3), δ 6.71 (m, 2H, ArH), δ 7.58 (m, 2H, ArH)

^{13}C NMR (101 MHz, $CDCl_3$): δ 54.88, 82.33, 115.94, 137.73, 158.99

Synthesis of 4-F-anisole ***22***

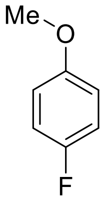

Prepared according to *General Procedure F*

Amount of 4-F-phenol: 0.1122 g, 1.00 mmol

Product yield: 0.0877 g, 0.696 mmol, 70%

IR: v_{max} 3055, 3001, 2953, 2934, 2837, 2355, 2335, 1721, 1668, 1601, 1296, 1250, 1210, 1120, 1034, 887, 723cm^{-1}

^{1}H NMR (400 MHz, $CDCl_3$): δ 3.81 (s, 3H, OCH_3), δ 6.86 (m, 2H, ArH), δ 7.00 (m, 2H, ArH)

^{13}C NMR (101 MHz, $CDCl_3$): δ 55.23, 114.25 (d, J_{CF}^3 = 7.51 Hz), 115.25 (d, J_{CF}^2 = 22.89), 155.37 (d, J_{CF}^1 = 34.30), 157.91

^{19}F NMR (376 MHz, $CDCl_3$): δ -124.40

Synthesis of 4-trifluoromethoxy-anisole ***23***

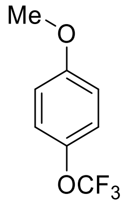

Prepared according to *General Procedure F*

Amount of 4-trifluoromethoxy-phenol: 0.1781 g, 1.00 mmol

Product yield: 0.0403 g, 0.210 mmol, 21%

IR: v_{max} 2361, 1584, 1481, 1285, 1238, 1172, 1026, 997, 831, 808, 583 cm^{-1}

^{1}H NMR (400 MHz, $CDCl_3$): δ 3.83 (s, 3H, OCH_3), δ 6.92 (m, 2H, ArH), δ 7.18 (m, 2H, ArH)

^{13}C NMR (101 MHz, $CDCl_3$): δ 54.97, 114.11, 120.19 (q, $J_{CF}{}^1$ = 255.7 Hz), 121.86, 142.23, 157.63

^{19}F NMR (376 MHz, $CDCl_3$): δ -58.53

Synthesis of 2,6-diphenylanisole ***24***

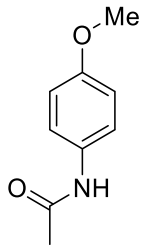

Prepared according to *General Procedure F*

Amount of 4-acetamidophenol: 0.1548 g, 1.01 mmol

Product yield: 0.1279 g, 0.774 mmol, 77%

Melting point: 129-131 °C

IR: v_{max} 2359, 1650, 1609, 1560, 1056, 1437, 1370, 1327, 1257, 1225, 1171, 835, 806, 683 cm^{-1}

^{1}H NMR (400 MHz, $CDCl_3$): δ 2.12 (s, 3H, CH_3), δ 3.78 (s, 3H, OCH_3), δ 6.83 (d, 2H, ArH, J = 8.77 Hz), δ 7.39 (d, 2H, ArH, J = 8.77 Hz), δ 7.99 (broad s, 1H, NH)

^{13}C NMR (101 MHz, $CDCl_3$): δ 23.88, 54.98, 113.66, 121.39, 130.42, 155.98, 167.59

Synthesis of 2,6-diphenylanisole ***25***

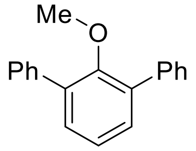

Prepared according to *General Procedure F*

Amount of 2,6-diphenylphenol: 0.2460 g, 1.00 mmol

Product yield: 0.2411 g, 0.926 mmol, 93%

IR: ν_{max} 1497, 1462, 1441, 1406, 1227, 1177, 1084, 1028, 1005, 804, 762, 748, 696, 611, 582 cm^{-1}

^{1}H NMR (400 MHz, $CDCl_3$): δ 3.34 (s, 3H, OCH_3), δ 7.39 (dd, 1H, ArH, *J* = 7.00, 8.14 Hz), δ 7.52 (m, 4H, ArH), δ 7.60 (m, 4H, ArH), δ 7.80 (m, 4H, ArH)

^{13}C NMR (101 MHz, $CDCl_3$): δ 60.07, 123.90, 126.78, 127.84, 129.00, 130.03, 135.42, 138.39, 154.63

Synthesis of 1,2,3-trimethoxybenzene ***26***

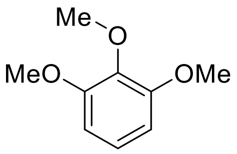

Prepared according to *General Procedure F*

Amount of 2,6-methoxyphenol: 0.1542 g, 1.00 mmol

Product yield: 0.1395 g, 0.831 mmol, 83%

Melting point: 41-42 °C

IR: ν_{max} 2940, 2835, 2359, 1593, 1498, 1475, 1435, 1296, 1254, 1229, 1172, 1103, 1094, 1001, 901, 777, 739, 696 cm^{-1}

^{1}H NMR (400 MHz, $CDCl_3$): δ 3.85 (s, 3H, O-CH_3) δ 3.89 (s, 6H, O-CH_3), δ 6.61 (d, 2H, ArH, *J* = 8.39 Hz), δ 7.02 (t, 1H, ArH, *J* = 8.39 Hz)

^{13}C NMR (101 MHz, $CDCl_3$): δ 55.58, 60.33, 104.75, 123.12, 137.68, 153.05

Synthesis of 2,6-dimethylanisole ***27***

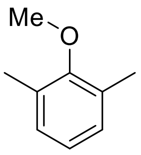

Prepared according to *General Procedure F*

Amount of 2,6-methylphenol: 0.1222 g, 1.00 mmol

Product yield: 0.1185 g, 0.870 mmol, 87%

IR: ν_{max} 2943, 1475, 1416, 1263, 1213, 1170, 1091, 1018, 811, 769 cm^{-1}

^{1}H NMR (400 MHz, $CDCl_3$): δ 3.80 (s, 6H, Ar-CH_3) δ 3.81 (s, 3H, O-CH_3), δ 6.53 (d, 2H, ArH, *J* = 8.43 Hz), δ 6.93 (t, 1H, ArH, *J* = 8.43 Hz)

^{13}**C NMR (101 MHz, CDCl$_3$):** δ 55.45, 60.16, 104.73, 123.11, 137.61, 152.97

Synthesis of 2-methylanisole ***28***

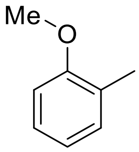

Prepared according to *General Procedure F*

Amount of 2-methylphenol: 0.1082 g, 1.00 mmol

Product yield: 0.0541 g, 0.442 mmol, 44%

IR: v_{max} 2999, 2941, 2916, 2833, 2357, 1601, 1585, 1489, 1460, 1435, 1288, 1260, 1190, 1165, 1151, 1043, 897, 843 cm^{-1}

^{1}**H NMR (400 MHz, CDCl$_3$):** δ 2.32 (s, 3H, Ar-CH$_3$) δ 3.90 (s, 3H, O-CH$_3$), δ 6.93 (m, 2H, ArH), δ 7.23 (m, 2H, ArH)

^{13}**C NMR (101 MHz, CDCl$_3$):** δ 15.74, 54.73, 109.44, 119.81, 126.12, 126.34, 130.15, 157.28

Synthesis of 2-methoxynaphthalene ***29***

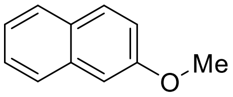

Prepared according to *General Procedure F*

Amount of 2-naphthol: 0.1444 g, 1.00 mmol

Product yield: 0.1525 g, 0.964 mmol, 96%

Melting point: 69-70 °C

IR: $\mathbf{v_{max}}$ 2361, 1628, 1589, 1473, 1439, 1389, 1258, 1213, 1169, 1115, 949, 897, 835, 816, 741, 698, 621 cm^{-1}

^{1}**H NMR (400 MHz, CDCl$_3$):** δ 3.90 (s, 3H, O-CH$_3$), δ 7.14 (d, 1H, ArH, *J* = 2.57 Hz), δ 7.18 (dd, 1H, ArH, *J* = 8.86 Hz), δ 7.35 (ddd, 1H, ArH, *J* = 8.15, 6.83, 1.24), δ 7.46 (ddd, 1H, ArH, *J* = 8.15, 6.86, 1.24), δ 7.72 (m, 3H, ArH)

^{13}**C NMR (101 MHz, CDCl$_3$):** δ 54.82, 105.37, 118.31, 123.18, 126.34, 127.26, 128.57, 128.99, 134.19, 157.20

Synthesis of estrone 3-methyl ether ***30***

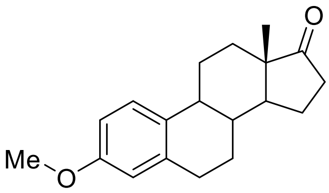

Prepared according to *General Procedure F*

Amount of α-estrone: 0.2704 g, 1.00 mmol

Product yield: 0. g, 0.856 mmol, 86%

Melting point: 169-170 °C

IR: $ν_{max}$ 2913, 2361, 1736, 1503, 1452, 1316, 1236, 1165, 1107, 910, 245, 822, 575 cm^{-1}

^{1}H NMR (400 MHz, $CDCl_3$): δ 0.93 (s, 3H, C-CH_3), δ 1.38-1.72 (m, 6H), δ 1.93-2.58 (m, 7H), δ 2.93 (m, 2H), δ 3.80 (s, 3H, O-CH_3), δ 6.67 (d, 1H, ArH, *J* = 2.78 Hz), δ 6.75 (dd, 1H, ArH, *J* = 2.78, 8.59 Hz), δ 7.22 (d, 1H, ArH, *J* = 8.59 Hz)

^{13}C NMR (101 MHz, $CDCl_3$): δ 13.39, 21.12, 25.48, 26.10, 29.21, 31.14, 35.37, 37.91, 43.48, 47.48, 49.92, 54.68, 111.08, 113.41, 125.83, 131.51, 137.22, 157.13, 220.19

Synthesis of 7-methoxycoumarin ***31***

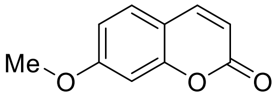

Prepared according to *General Procedure F*

Amount of 7-hydroxycoumarin: 0.1619 g, 1.00 mmol

Product yield: 0.174 g, 0.986 mmol, 99%

Melting point: 115-116 °C

IR: $ν_{max}$ 2361, 1701, 1611, 1504, 1398, 1350, 1281, 1231, 1123, 1024, 980, 891, 826, 750, 615 cm^{-1}

^{1}H NMR (400 MHz, $CDCl_3$): δ 3.79 (s, 3H, O-CH_3), δ 6.16 (d, 1H, C=C-H, *J* = 9.49 Hz), δ 6.69 (d, 1H, ArH, *J* = 2.47 Hz), δ 6.76 (dd, 1H, ArH, *J* = 8.60, 2.47 Hz), δ 7.30 (d, 1H, ArH, *J* = 8.60 Hz), δ 7.58 (d, 1H, C=C-H, *J* = 9.49 Hz)

^{13}C NMR (101 MHz, $CDCl_3$): δ 55.18, 100.25, 111.88, 111.95, 112.37, 128.30, 142.96, 155.27, 160.56, 162.26

Synthesis of thioanisole ***32***

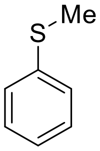

Prepared according to *General Procedure F*

Amount of thiophenol: 0.1100 g, 1.00 mmol

Product yield: 0.0958 g, 0.773 mmol, 77%

IR: ν_{max} 3057, 2918, 2359, 1582, 1479, 1439, 1315, 1088, 1024, 966, 893, 135, 669 cm^{-1}

^{1}H NMR (400 MHz, CDCl$_3$): δ 2.54 (s, 3H, S-CH$_3$), δ 7.20 (m, 1H, ArH), δ 7.35 (m, 4H, ArH)

^{13}C NMR (101 MHz, CDCl$_3$): δ 15.39, 124.56, 126.18, 128.36, 138.01

*Synthesis of methylbenzoate **33***

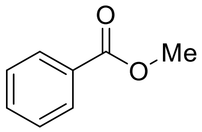

Prepared according to *General Procedure F*

Amount of benzoic acid: 0.1222 g, 1.00 mmol

Product yield: 0.1085 g, 0.797 mmol, 80%

IR: ν_{max} 2951, 2361, 1719, 1600, 1452, 1435, 1315, 1273, 1176, 1109, 1023, 964, 822, 708 cm^{-1}

^{1}H NMR (400 MHz, CDCl$_3$): δ 3.90 (s, 3H, O-CH$_3$), δ 7.43 (m, 2H, ArH), δ 7.53 (m, 1H, ArH), δ 8.05 (m, 2H, ArH)

^{13}C NMR (101 MHz, CDCl$_3$): δ 51.50, 127.82, 129.03, 129.66, 132.36, 166.51

22. Methylation of phenols in NMP using 3-Br-*N,N,N*-trimethylanilinium iodide

Optimization of methylation in NMP

Based on the standard conditions used to methylate phenols in DMSO, a brief screen of reaction conditions was monitored for the methylation of 4-^{t}Bu-phenol in NMP. The investigated conditions are shown in **table S14**.

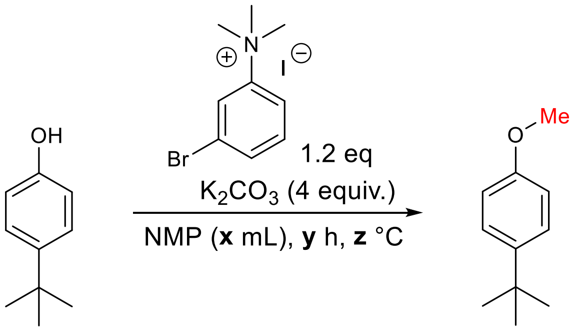

Entry	x (mL)	y (h)	z (°C)	Yield (%)
1	2	3	80	88
2	2	3	**50**	62
3	**5**	3	80	83
4	2	**8**	80	96

Table S17. Investigation of reaction conditions for the methylation of 4-tBu-phenol using 3-Br-*N*,*N*,*N*-trimethylanilinium iodide in NMP.

^{19}F NMR kinetics of trimethylanilinium iodide salt degradation in NMR versus DMSO

With NMP proving to be a suitable alternative solvent to DMSO for the methylation of phenols, the following questions about the behaviour of anilinium salts in NMP were raised: Do *N,N,N*-trimethylanilinium iodides still decompose to *N,N*-dimethylanilines and methyl iodide in NMP? And if so, does the rate of degradation compare to that observed in DMSO-d_6?

To begin answering these questions, a suitable method to monitor the degradation of *N,N,N*-trimethylanilinium salts in NMP needed to be developed. Ideally ^{1}H NMR kinetic experiments analogous to those already carried out in DMSO-d_6 would be used. However, NMP-d_9 is less widely available than DMSO-d_6 which was a prohibiting factor in its use. It was thought that NMR spectroscopy could be used to follow an alternative nucleus to ^{1}H, allowing the non-deuterated solvent to be used in a degradation experiment. To put this hypothesis to the test, 4-trifluoromethyl-*N,N,N*-trimethylanilinium iodide (**5a**) was chosen as a substrate to monitor with ^{19}F NMR spectroscopy.

A sequence was developed that allowed the NMR spectrometer to lock onto the N-CH_3 protons of NMP and carry out the shimming process, before obtaining a ^{19}F NMR spectrum of the sample. With this sequence set up, the T1 delay times of each fluorine nucleus in **5a**, 4-trifluoromethyl-*N,N*-dimethylaniline, and α,α,α-trifluorotoluene (which would be used as an internal standard) were calculated *via* a T1ir experiment. A mixture of **5a** (0.1 M) and α,α,α-trifluorotoluene (0.1 M) was prepared in NMP and heated at 80 °C for 16.5 h whilst ^{19}F NMR spectra were recorded at regular time intervals. The resulting spectra were stacked, which could be processed to calculate the concentrations of **5a** and 4-trifluoromethyl-*N,N*-dimethylaniline over the time course (**Figure**).

Unfortunately, the nature of the experiment meant that any non-innocence of NMP would not be observed (unless it involved the generation of a new fluorine-containing species). Though

the assumption could not be tested, estimation of degradation rate constants were based solely on the reversible iodide-led degradation of **5a** (**Figure**). Using COPASI the estimated rate constants for this reaction were $k_1 = 4.44 \times 10^{-4}\ s^{-1}$ and $k_{-1} = 4.78 \times 10^{-4}\ M^{-1}s^{-1}$.

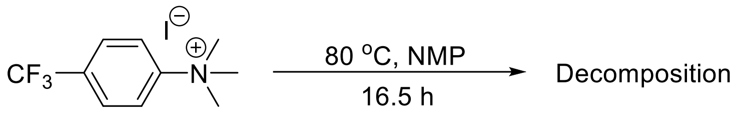

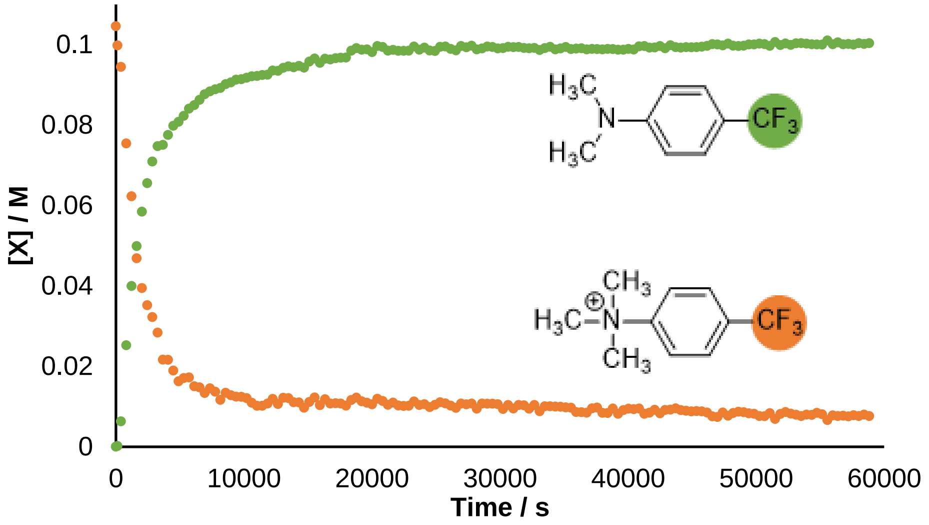

Figure S31. Degradation time course of **5a** (0.1 M) in NMP at 80 °C monitored in real-time by ^{19}F NMR spectroscopy. The concentrations of **5a** and 4-trifluoromethyl-*N*,*N*-dimethylaniline were determined from their trifluoromethyl groups with respect to the internal standard, α,α,α-trifluorotoluene.

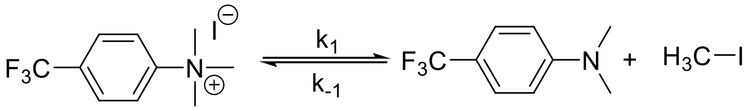

Figure S32. Proposed degradation reaction of **5a** in NMP with assigned rate constants k_1 and k_{-1}.

The monitoring of **5a** in NMP at 80 °C revealed that the *N,N,N*-trimethylanilinium iodide salt does thermally degrade. The rate constant for iodide-led degradation (k_1) was an order of magnitude higher than that observed for any of the anilinium salts tested in DMSO-d_6. The concentration *vs* time plots for **5a** in both DMSO-d_6 and NMP are overlaid in **Figure S33**. The first observation here is the noticeable difference in degradation rate: $k_1 = 8.08 \times 10^{-5}\ s^{-1}$ in DMSO-d_6 and $k_1 = 4.44 \times 10^{-4}\ s^{-1}$ in NMP. The second point to consider is that, **5a** appears to reach an equilibrium concentration of about 0.05 M in DMSO-d_6. In NMP, the salt almost completely degrades. Though DMSO and NMP largely have similar properties, differences in their physical parameters such as dielectric constant (74 vs 42) or viscosity (2.00 vs 1.66

mPas), may contribute to differences in *N,N,N*-trimethylanilinium salt stability in solution.[178] Alternatively, the ability of methyl iodide to react with DMSO to form TMSOI may act as a methyl iodide "sponge" to keep the volatile methylating reagent in solution. Without this mechanism in place for NMP, the methyl iodide could exist mainly in the NMR headspace which limits the rate of the backwards reaction. This cannot be probed with ^{19}F NMR.

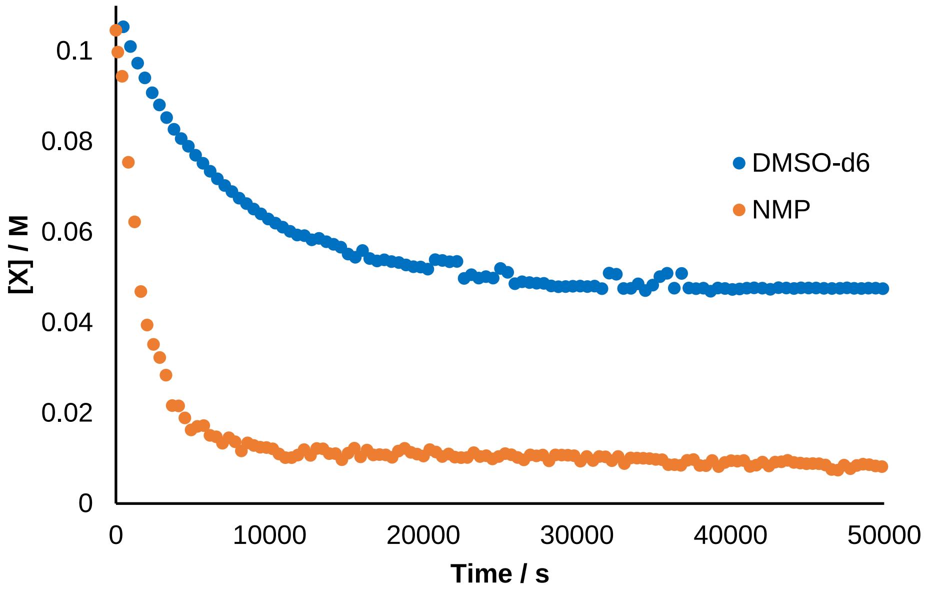

Figure S33. Comparison of the degradation of **5a** in DMSO-d_6 (blue) and NMP (orange).

23. Thermal degradation of trimethylsulfoxonium iodide in DMSO-d_6

Trimethylsulfoxonium iodide (13.2 mg, 60 μmol) and 1,2,4,5-tetramethylbenzene internal standard (4.8 mg, 36 μmol) were dissolved in DMSO-d_6 (0.6 mL, 0.7140 g), to give trimethylsulfoxonium iodide concentration of 0.10 M, and an internal standard concentration of 0.06 M, then transferred to an NMR tube. A ^{1}H NMR spectrum (4 scans, 25 s relaxation time) was taken every 5 min whilst the sample was heated to 80 °C in the NMR spectrometer probe.

The concentration of the trimethylsulfoxonium salt and methyl iodide were calculated *via* their CH_3 protons at each time point against the internal standard. The concentration of each species *vs* time is shown in **figure S34**.

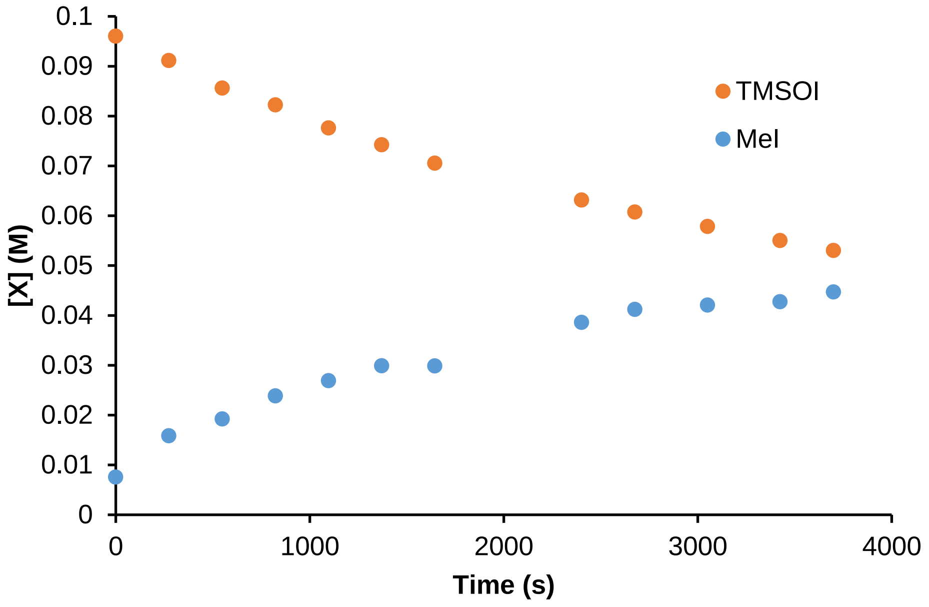

Figure S34. Degradation of trimethylsulfoxium iodide and evolution of methyl iodide determined *via* ^{1}H NMR monitoring in DMSO-d_6 80 °C.

Methyl iodide (8.5 mg, 60 μmol) and 1,2,4,5-tetramethylbenzene internal standard (4.8 mg, 36 μmol) were dissolved in DMSO-d_6 (0.6 mL, 0.7140 g), to give methyl iodide concentration of 0.10 M, and an internal standard concentration of 0.06 M, then transferred to an NMR tube. A ^{1}H NMR spectrum (4 scans, 25 s relaxation time) was taken every 5 min whilst the sample was heated to 80 °C in the NMR spectrometer probe.

The concentration of the trimethylsulfoxonium salt and methyl iodide were calculated *via* their CH_3 protons at each time point against the internal standard. The concentration of each species *vs* time is shown in **figure S35**.

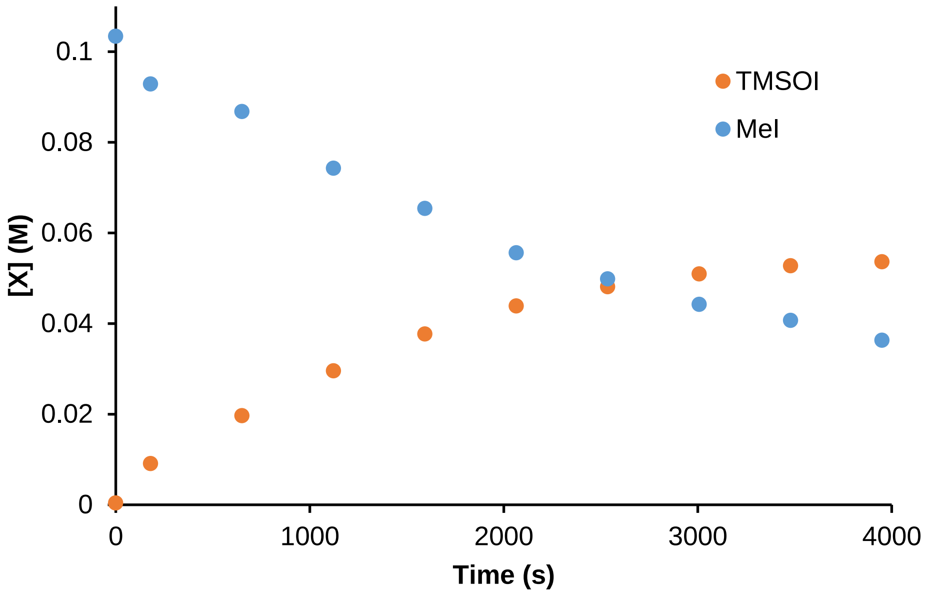

Figure S35. Degradation of trimethylsulfoxium iodide and evolution of methyl iodide determined *via* ^{1}H NMR monitoring in DMSO-d_6 80 °C

24. Mechanistic experiments on Speciation of Active Methylating Species

*Synthesis of trimethylsulfoxonium hexafluorophosphate, **35d***

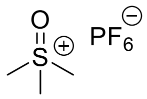

Trimethylsulfoxonium iodide (0.6602 g, 3 mmol, 1 equiv.) was dissolved in the minimum amount of deionised water to achieve dissolution. Potassium hexafluorophosphate (0.5522 g, 3 mmol, 1 equiv.) was added and the resulting mixture was stirred at room temperature for 3 h causing a white precipitation to form. The solid was collected *via* vacuum filtration and washed with deionised water (3 x 5 mL), then dried in a vacuum oven overnight to give a white powder (0.5671 g, 79%).

Melting point: Decomposed at 256 °C

IR: ν_{max} 3003, 2914, 2359, 1412, 1233, 1040, 845, 822 cm^{-1}

^{1}H NMR (400 MHz, DMSO-d_6): δ 3.83 (s, 9H, S-$(CH_3)_3$)

^{13}C NMR (101 MHz, DMSO-d_6): δ 38.95

^{19}F NMR (376 MHz, DMSO-d_6): δ -70.09 (d, PF_6^-, J = 711 Hz)

^{31}P NMR (162 MHz, DMSO-d_6): δ -146.39 (hep, J = 711 Hz)

*Synthesis of trimethylsulfoxonium tetrakis(3,5-bis-(trifluoromethyl)phenyl) borate, **35f***

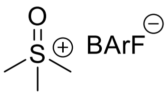

The trimethylsulfoxonium iodide (0.3756 g, 1.707 mmol, 1 equiv.) and sodium tetrakis(3,5-bis(trifluoromethyl)phenyl borate (1 equiv.) were stirred in a 1:1 biphasic mixture of deionised water and dichloromethane. The organic layer was then collected and dried over sodium sulfate. The solvent was removed *in vacuo* to give a white powder.

Melting point: Decomposed at 237 °C

IR: ν_{max} 2631, 2331, 1609, 1352, 1271 1144, 1113, 1028, 885, 837, 726, 710, 669 cm^{-1}

^{1}H NMR (400 MHz, DMSO-d_6): δ 3.83 (s, 9H, S-$(CH_3)_3$), δ 7.62 (m, 8H, ArH BArF), δ 7.69 (s, 4H, ArH BArF)

^{13}C NMR (101 MHz, DMSO-d_6): δ 38.91, 117.66, 123.97j (q, $J_{CF}{}^1$ = 271.9 Hz), 128.43 ($J_{CF}{}^2$ = 30 Hz), 134.02, 147.21, 160.89 (q, $J_{CB}{}^1$ = 49 Hz)

^{19}F NMR (376 MHz, DMSO-d_6): δ -61.75

^{11}B NMR (MHz, DMSO-d_6): δ -6.82

*General procedure for methylation of **16** for mechanistic experiments*

4-^{t}Bu-phenol (0.1502 g, 1 mmol, 1 equiv.), methylating reagent (1.2 mmol, 1.2 equiv.), potassium carbonate (0.553 g, 4 mmol, 4 equiv.), and solvent (2 mL) were added to a 25 mL round bottom flask equipped with a stir bar. The reaction mixture was heated to 80 °C with stirring for 3 h open to air. After cooling, the reaction was diluted with water (10 mL) and extracted with dichloromethane (3 x 15 mL). The organic layers were then combined, washed with 1M HCl (3 x 20 mL), dried over $MgSO_4$, and filtered. The solvent was then removed *in vacuo* to give the crude methylated product. Column chromatography on silica gel was used to deliver **17** as a brown oil.

Entry	Methylating reagent	Solvent	Yield 17 (%)
1	**11d**	DMSO	45
2	**11f**	DMSO	50
3	**35a**	DMSO	76
4	**35d**	DMSO	57
5	**35f**	DMSO	53
6	**11a**	NMP	88
7	**11d**	NMP	11

Table S18. Conversion of **16** to **17** with various methylating reagents according to general procedure for mechanistic experiments.

25. Conductivity Measurements

Single Point Measurements

Salt **3a** and DMSO were added to a 50 mL round bottom flask, with a stirrer bar. A Jenway 4510 Bench Conductivity Meter was used to measure conductivity of each solution at room temperature. AS expected, the conductivity was observed to increase with increasing concentration of anilinium iodide salt added to DMSO. The data are shown in **figure S36** and **table S19** below.

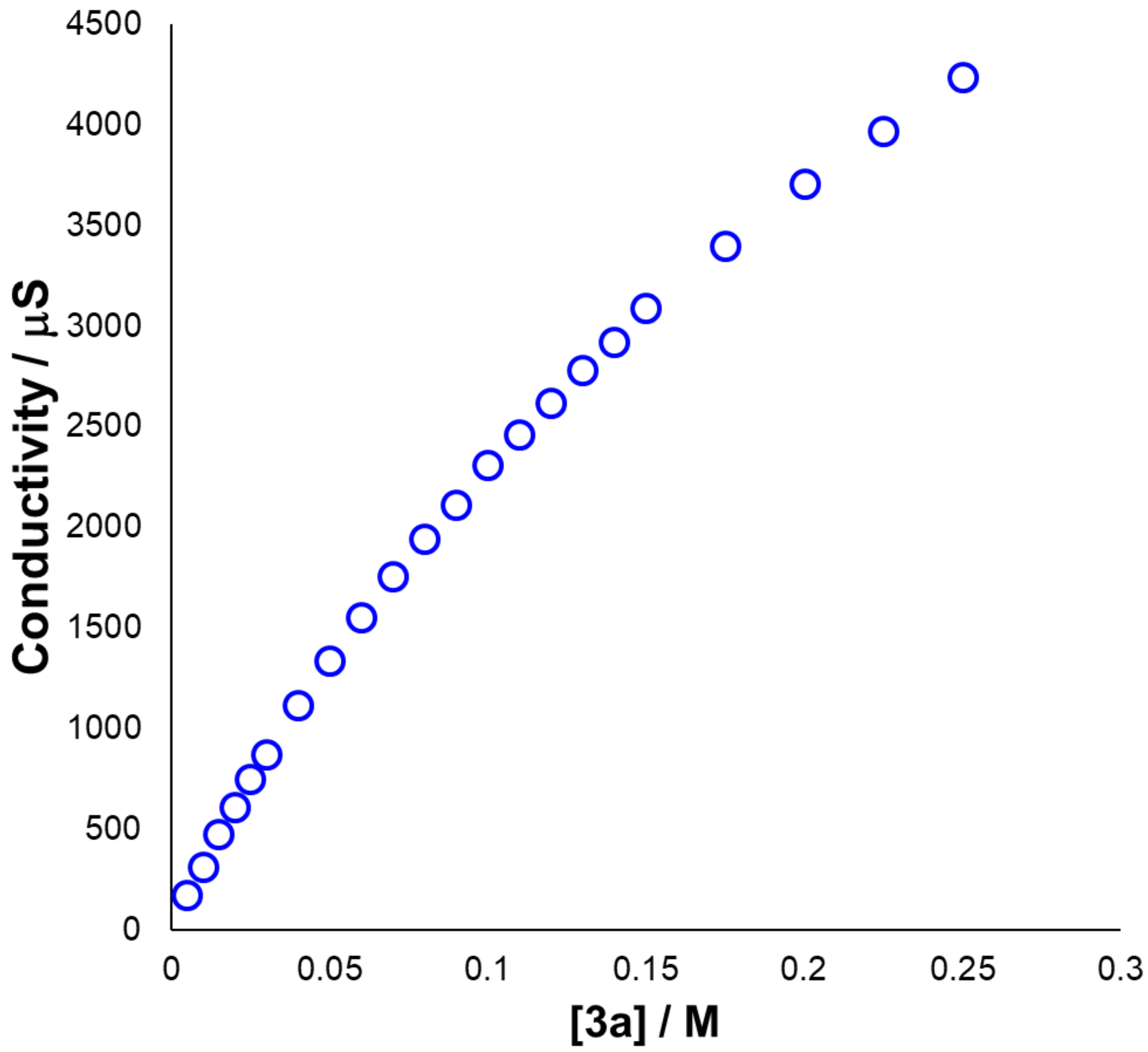

Figure S36. Single point experimental measurements of solution conductivity for a range of salt **3a** (4-CHO iodide) in DMSO at room temperature.

[3a] / M	Conductivity / µS
0.005	170.8
0.01	313
0.015	477
0.02	610
0.025	748
0.03	873
0.04	1116
0.05	1336
0.06	1552
0.07	1755
0.08	1944
0.09	2110
0.1	2310
0.11	2460
0.12	2620
0.13	2780
0.14	2920
0.15	3090
0.175	3400
0.2	3710
0.225	3970
0.25	4240

Table S19. *Single point experimental measurements of solution conductivity for a range of salt* ***3a*** *(4-CHO iodide) in DMSO.*

Kinetic Analysis

4-formyl-*N,N,N*-trimethylanilinium iodide (**3a**, 291 mg, 1 mmol) was dissolved in DMSO-d_6 (10 mL, 11 g) in a 25 mL round bottom flask, with a stirrer bar. The solution was heated to 80 ºC. The conductivity meter was submerged in the stirred solution and used to periodically note the solution phase conductivity. The results are displayed in **figure S37** and **table S20**. The noted NMR concentrations relate to a comparable experiment in which concentration was the main measurement quantity versus time. The concentration and conductivity data are coplotted to more intuitively contextualise the latter.

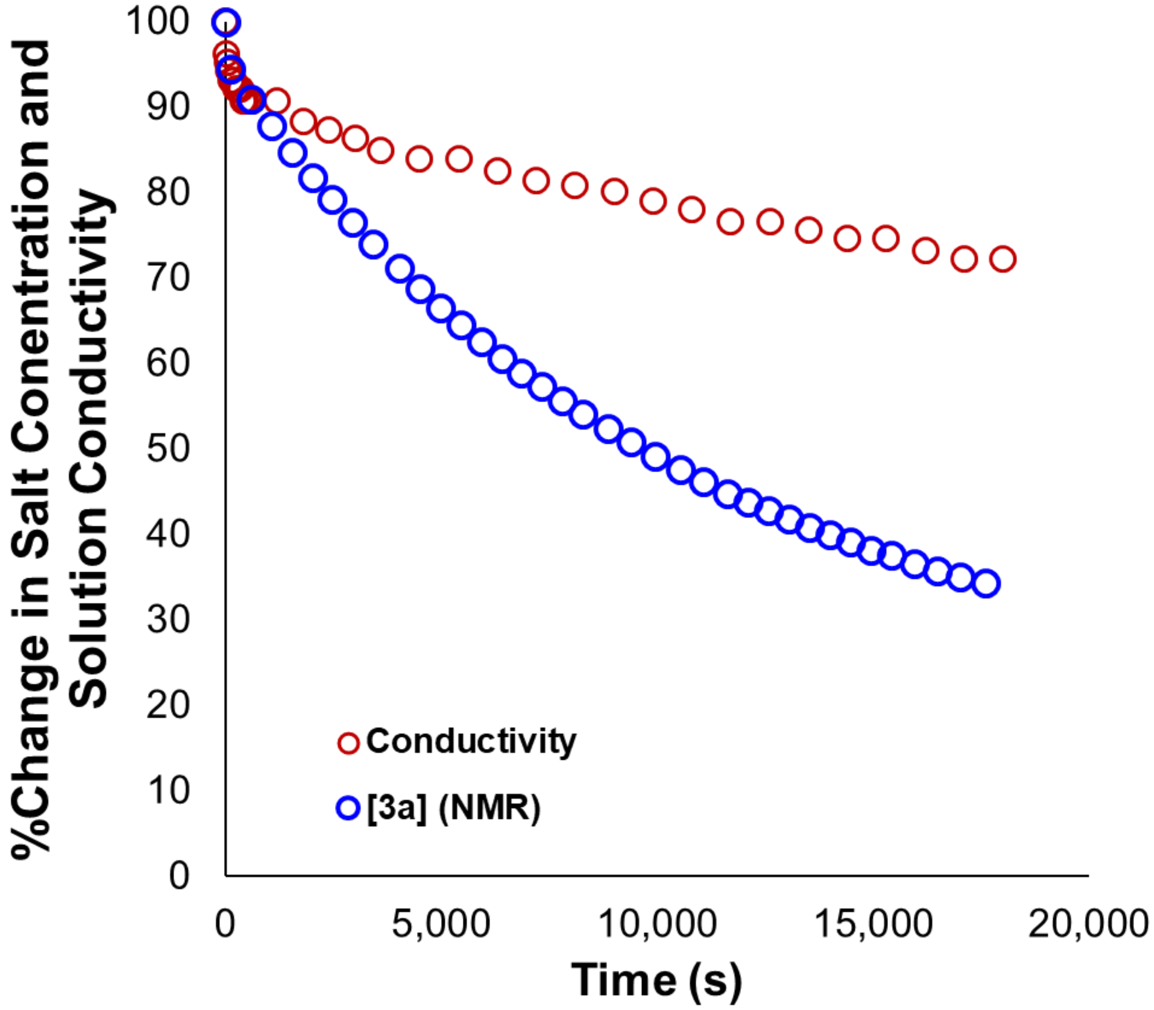

Figure S37. Kinetic analysis of relative salt concentration and conductivity changes during the course of a controlled degradation of anilinium salt **3a**.

NMR Time / s	Concentration / M	% Concentration vs t_0	Conductivity Time / s	Conductivity / mS	% Conductivity vs t_0
0	0.102	100.000	6000.000	2.910	100.000
120	0.096	94.454	5667.261	2.800	96.220
592	0.093	90.880	5452.801	2.770	95.189
1063	0.090	87.757	5265.429	2.740	94.158
1534	0.086	84.716	5082.984	2.710	93.127
2005	0.083	81.794	4907.615	2.710	93.127
2476	0.081	79.164	4749.837	2.680	92.096
2948	0.078	76.515	4590.894	2.680	92.096
3419	0.076	73.990	4439.409	2.680	92.096
4020	0.073	71.159	4269.533	2.640	90.722
4501	0.070	68.750	4125.014	2.640	90.722
4973	0.068	66.546	3992.776	2.640	90.722
5444	0.066	64.498	3869.864	2.640	90.722
5915	0.064	62.492	3749.550	2.640	90.722
6386	0.062	60.574	3634.427	2.570	88.316
6857	0.060	58.845	3530.689	2.540	87.285
7329	0.058	57.284	3437.060	2.510	86.254
7800	0.057	55.555	3333.292	2.470	84.880
8271	0.055	54.045	3242.679	2.440	83.849
8860	0.053	52.378	3142.667	2.440	83.849
9374	0.052	50.808	3048.472	2.400	82.474
9942	0.050	49.164	2949.851	2.370	81.443
10540	0.049	47.531	2851.833	2.350	80.756
11070	0.047	46.173	2770.377	2.330	80.069
11622	0.046	44.814	2688.826	2.300	79.038
12095	0.045	43.783	2626.978	2.270	78.007
12567	0.044	42.742	2564.491	2.230	76.632
13038	0.043	41.816	2508.934	2.230	76.632
13510	0.042	40.852	2451.121	2.200	75.601
13981	0.041	39.929	2395.759	2.170	74.570
14453	0.040	39.079	2344.770	2.170	74.570
14927	0.039	38.178	2290.694	2.130	73.196
15420	0.038	37.494	2249.669	2.100	72.165
15955	0.037	36.640	2198.426	2.100	72.165
16463	0.037	35.786			
17005	0.036	35.092			
17594	0.035	34.317			

Table S20. *Raw data for the kinetic co-analysis of salt degradation and solution conductivity.*

26. ^{1}H and ^{13}C NMR spectra of *N,N,N*-trimethylanilinium salts

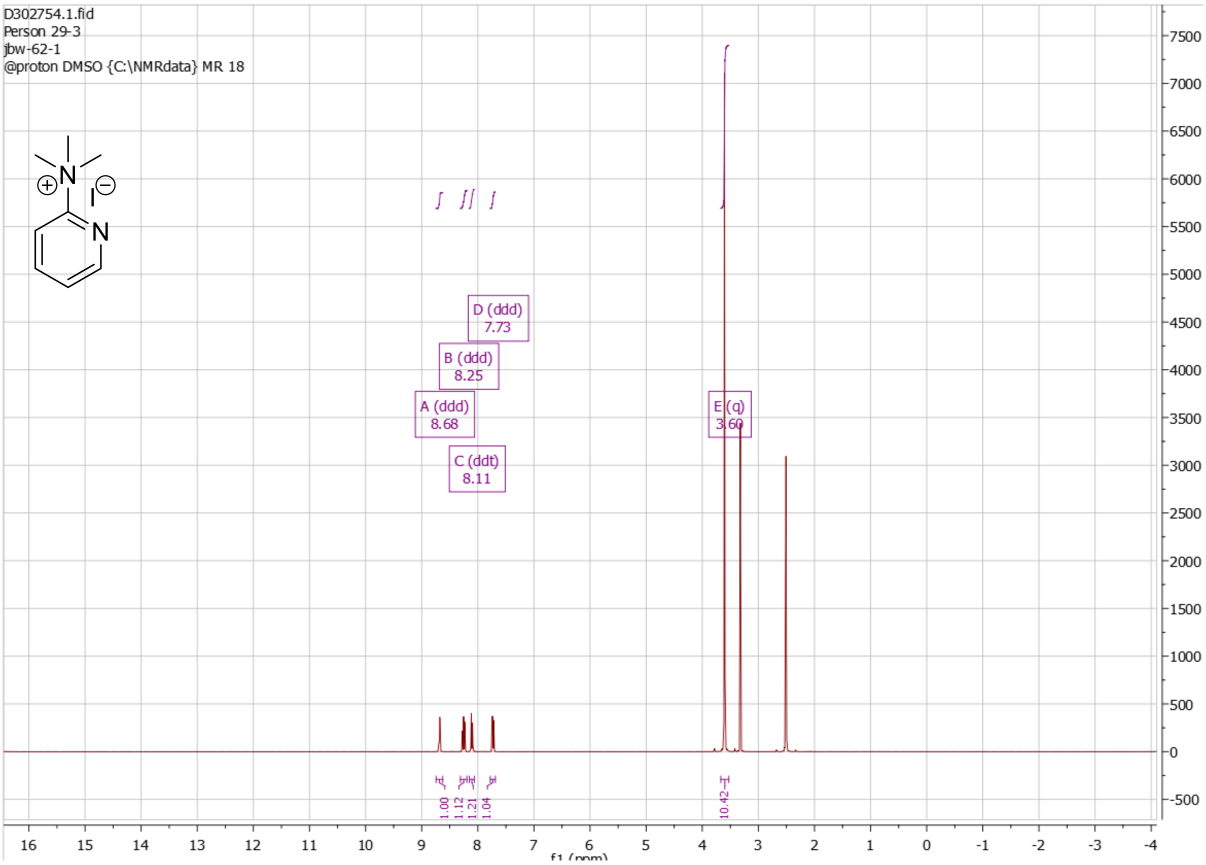

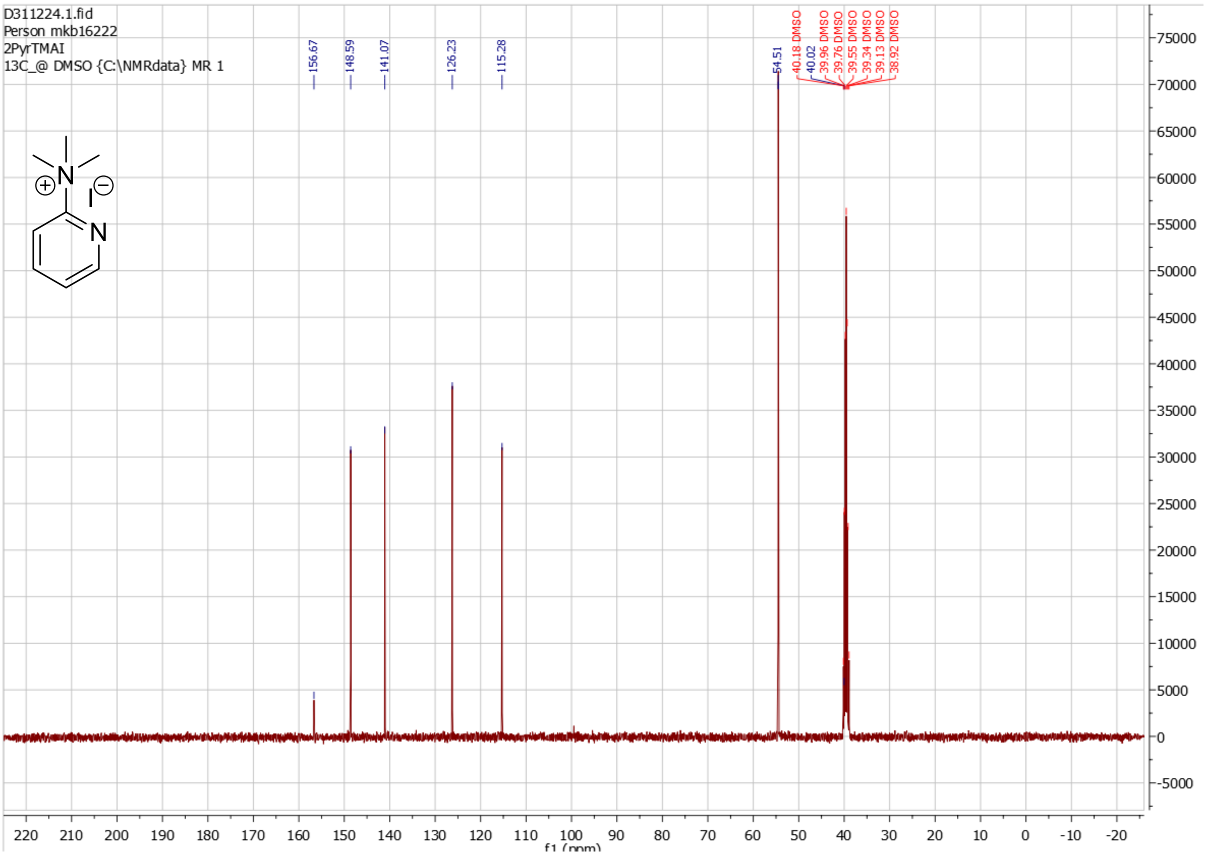

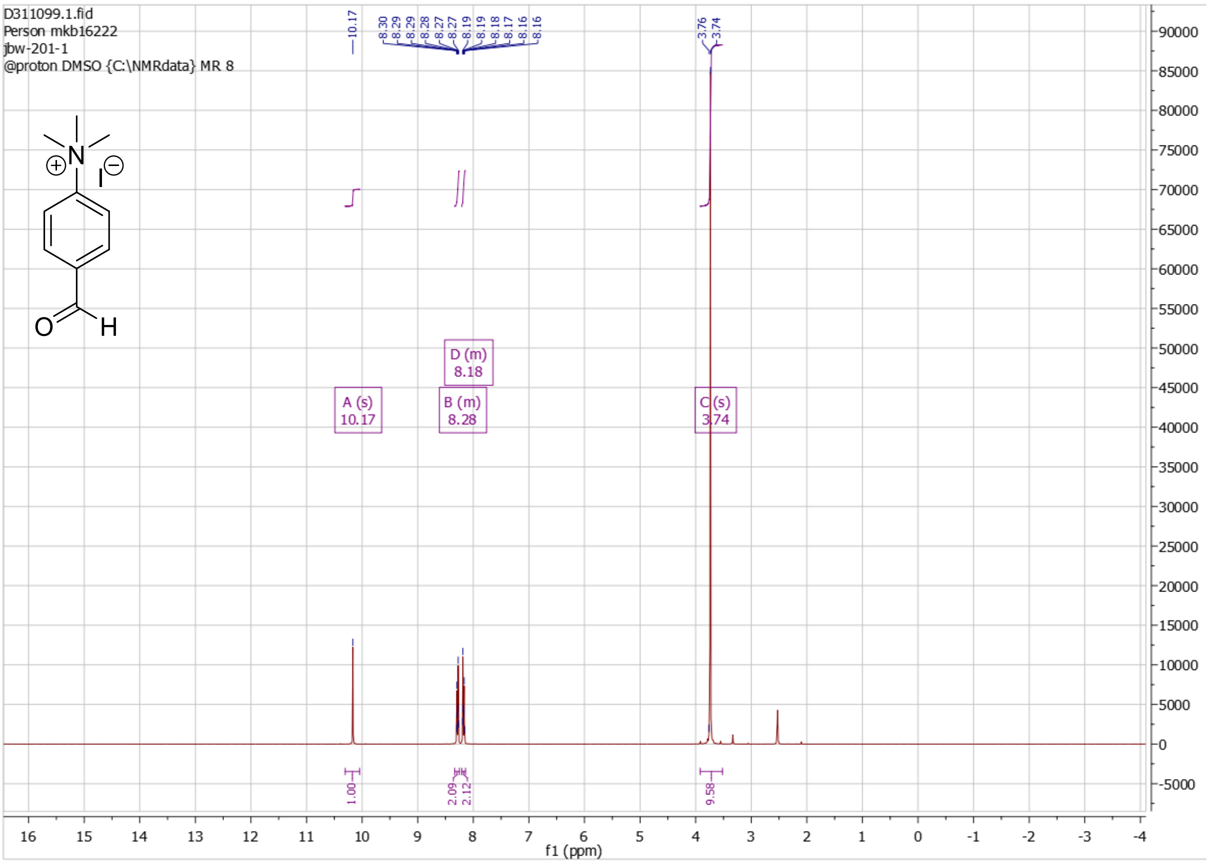
D311099.1.fid
Person mkb16222
jbw-201-1
@proton DMSO {C:\NMRdata} MR 8
A (s)
10.17
B (m)
8.28
D (m)
8.18
C (s)
3.74
f1 (ppm)

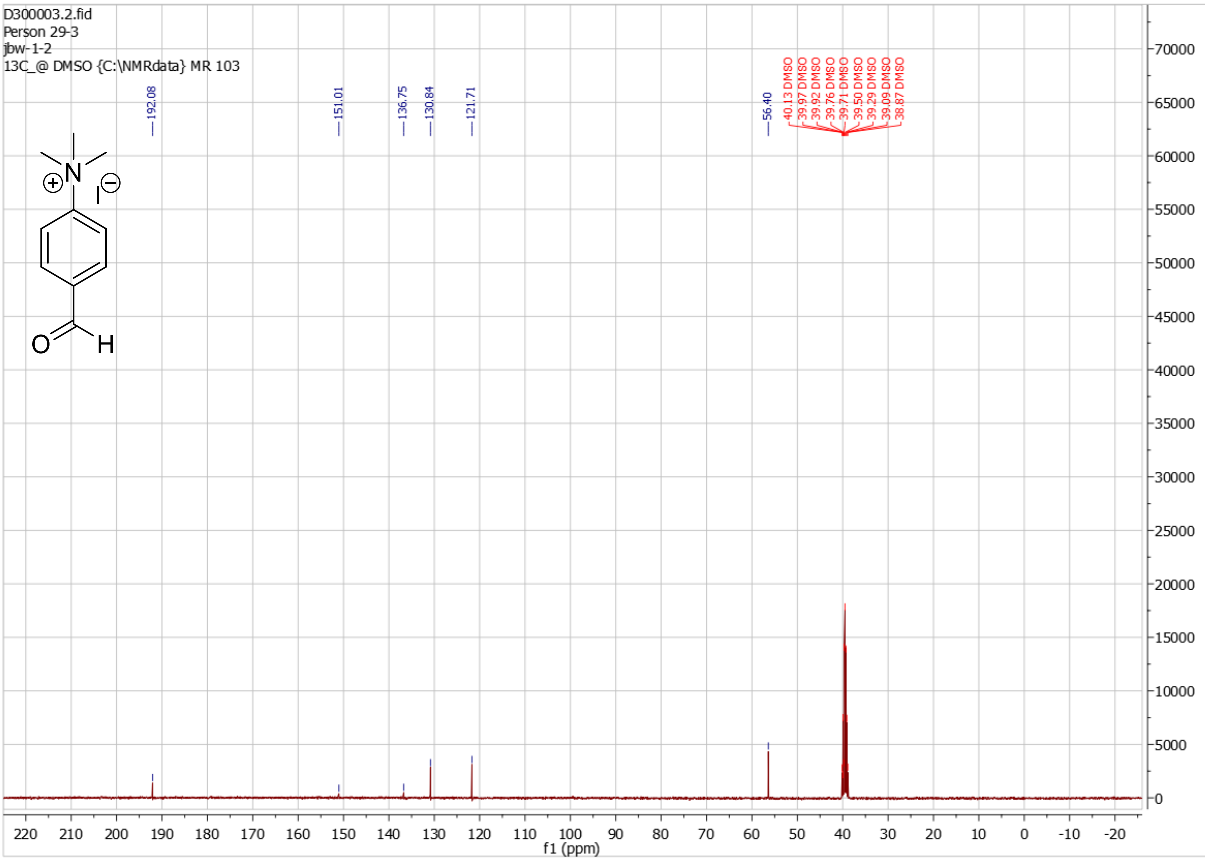
D300003.2.fid
Person 29-3
jbw-1-2
13C_@ DMSO {C:\NMRdata} MR 103
192.08
151.01
136.75
130.84
121.71
56.40
f1 (ppm)

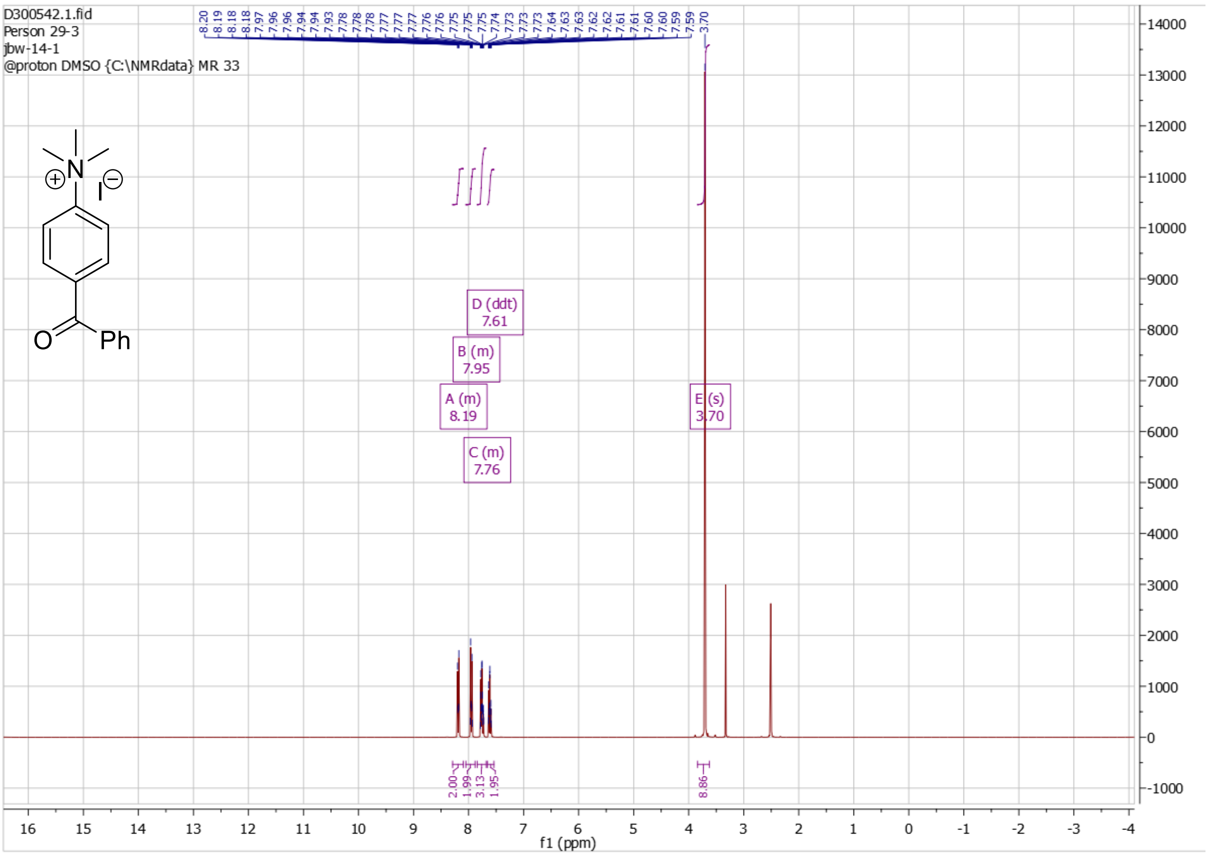
D300542.1.fid
Person 29-3
jbw-14-1
@proton DMSO {C:\NMRdata} MR 33
8.20 8.19 8.18 8.18 7.97 7.96 7.96 7.94 7.94 7.93 7.78 7.78 7.78 7.77 7.77 7.77 7.76 7.76 7.75 7.75 7.75 7.74 7.73 7.73 7.73 7.64 7.63 7.62 7.62 7.61 7.61 7.60 7.59 7.59 3.70
N
I
O
Ph
D (ddt)
7.61
B (m)
7.95
A (m)
8.19
E (s)
3.70
C (m)
7.76
2.00
1.99
3.13
1.95
8.86
f1 (ppm)

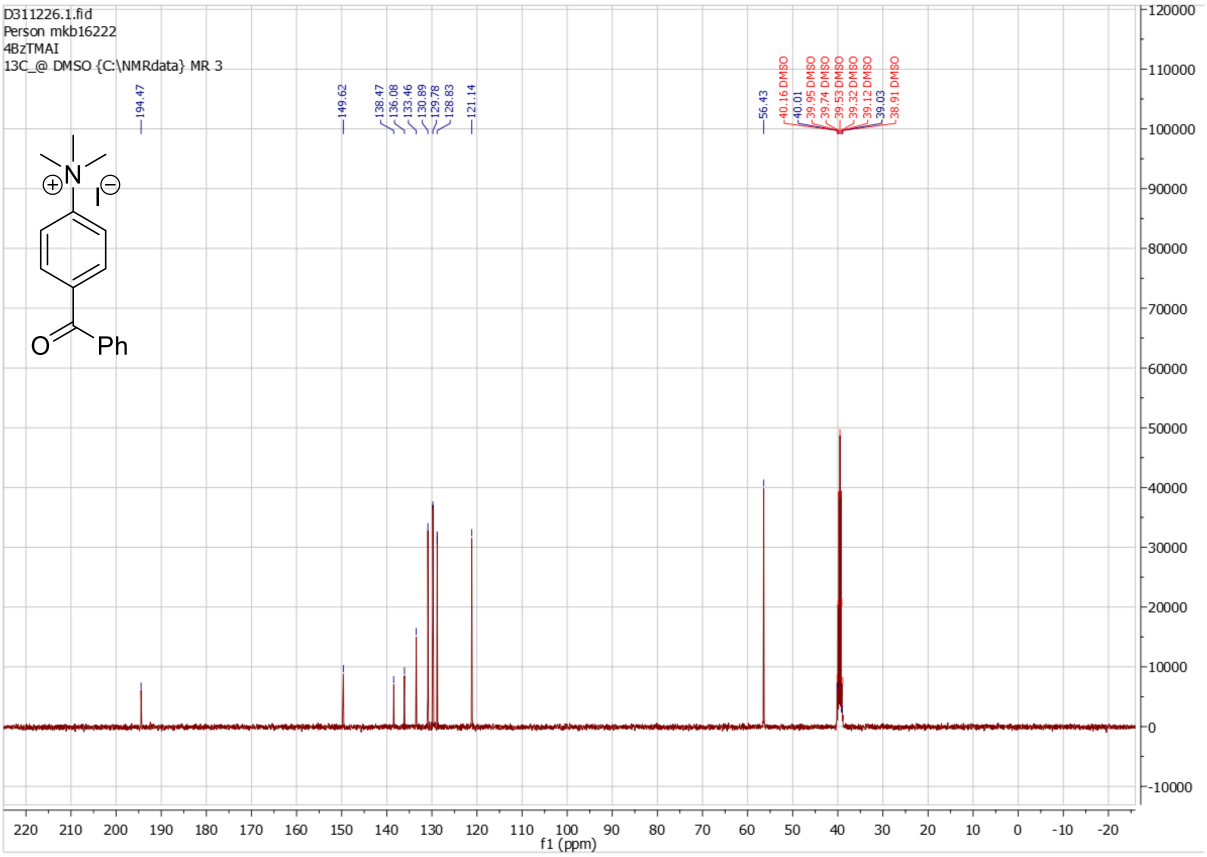
D311226.1.fid
Person mkb16222
4BzTMAI
13C_@ DMSO {C:\NMRdata} MR 3
194.47
149.62
138.47 136.08 133.46 130.89 129.78 128.83
121.14
56.43
40.16 DMSO 40.01 39.95 DMSO 39.74 DMSO 39.53 DMSO 39.32 DMSO 39.12 DMSO 39.03 38.91 DMSO
N
I
O
Ph
f1 (ppm)

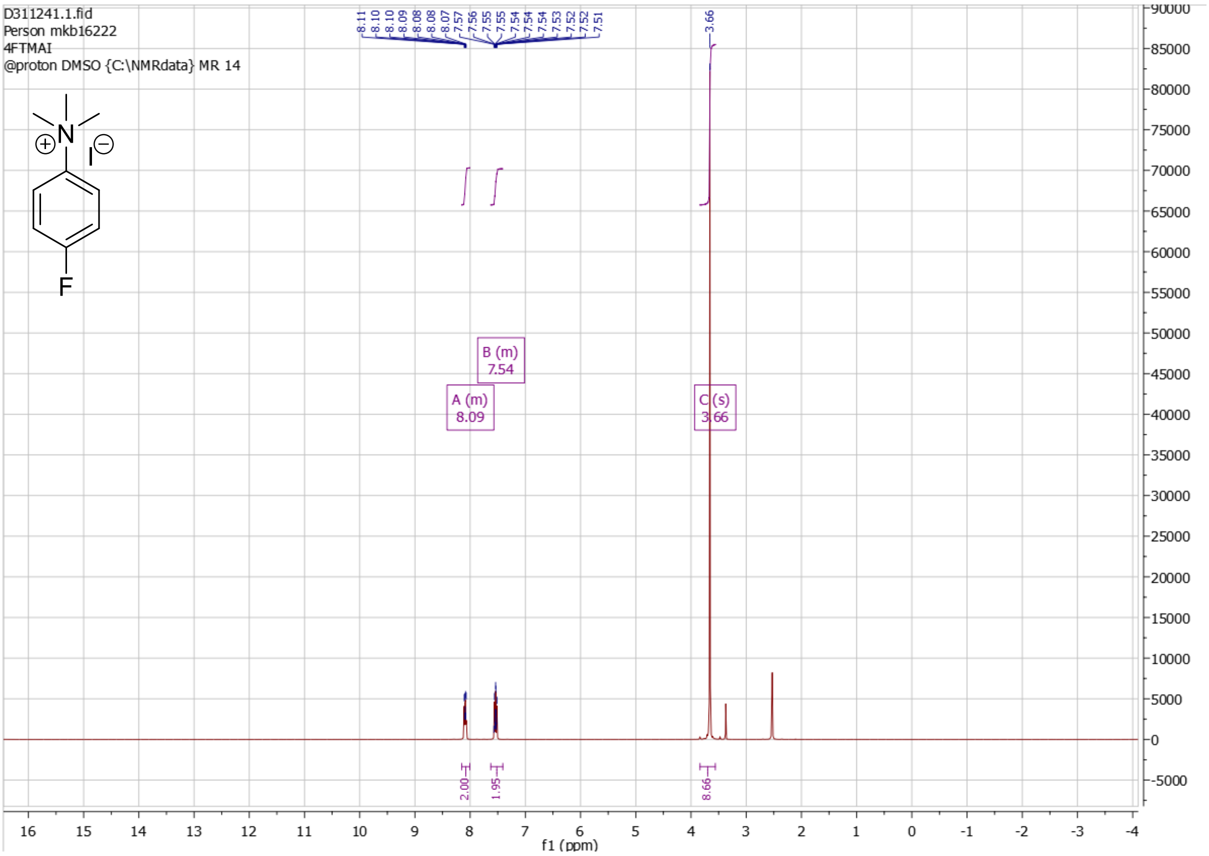
D311241.1.fid
Person mkb16222
4FTMAI
@proton DMSO {C:\NMRdata} MR 14
A (m)
8.09
B (m)
7.54
C (s)
3.66
2.00
1.95
8.66
f1 (ppm)

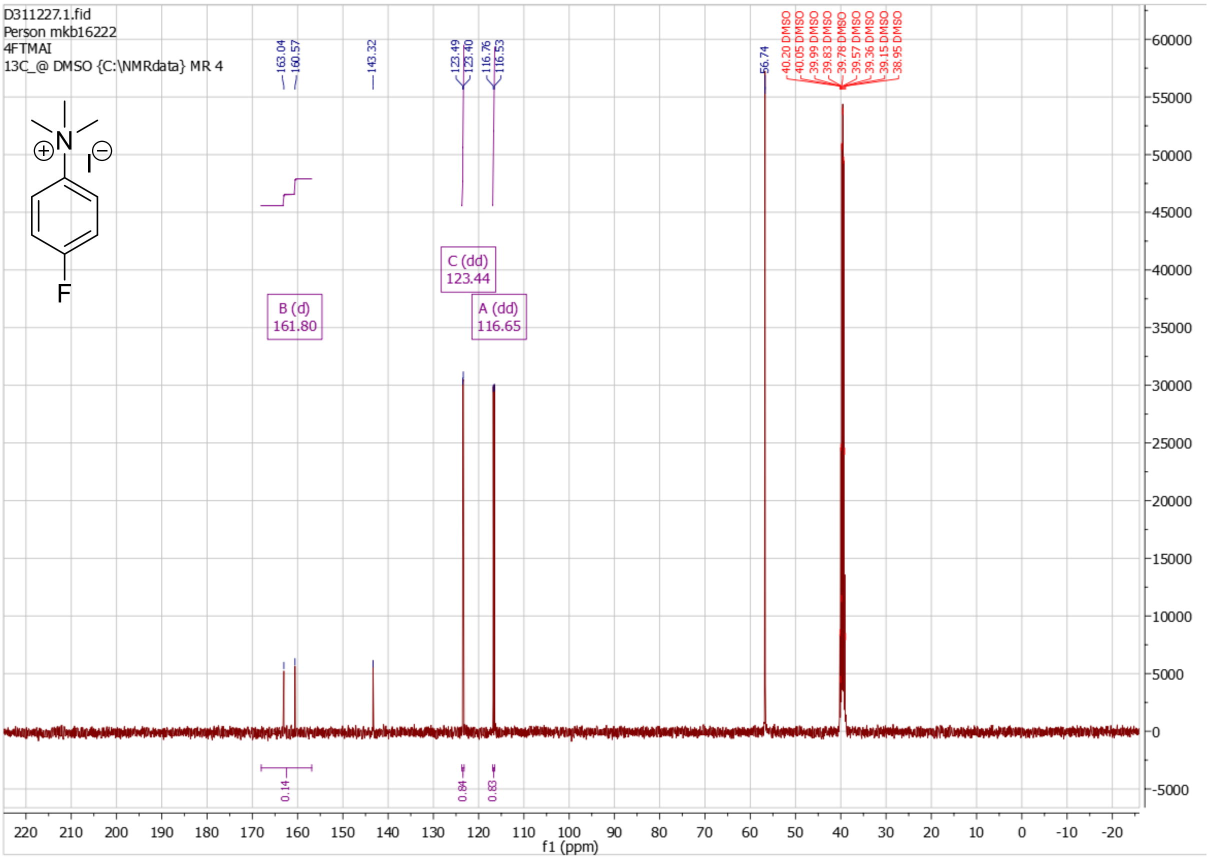
D311227.1.fid
Person mkb16222
4FTMAI
13C_@ DMSO {C:\NMRdata} MR 4
B (d)
161.80
C (dd)
123.44
A (dd)
116.65
163.04
160.57
143.32
123.49
123.40
116.76
116.53
56.74
0.14
0.84
0.83
f1 (ppm)

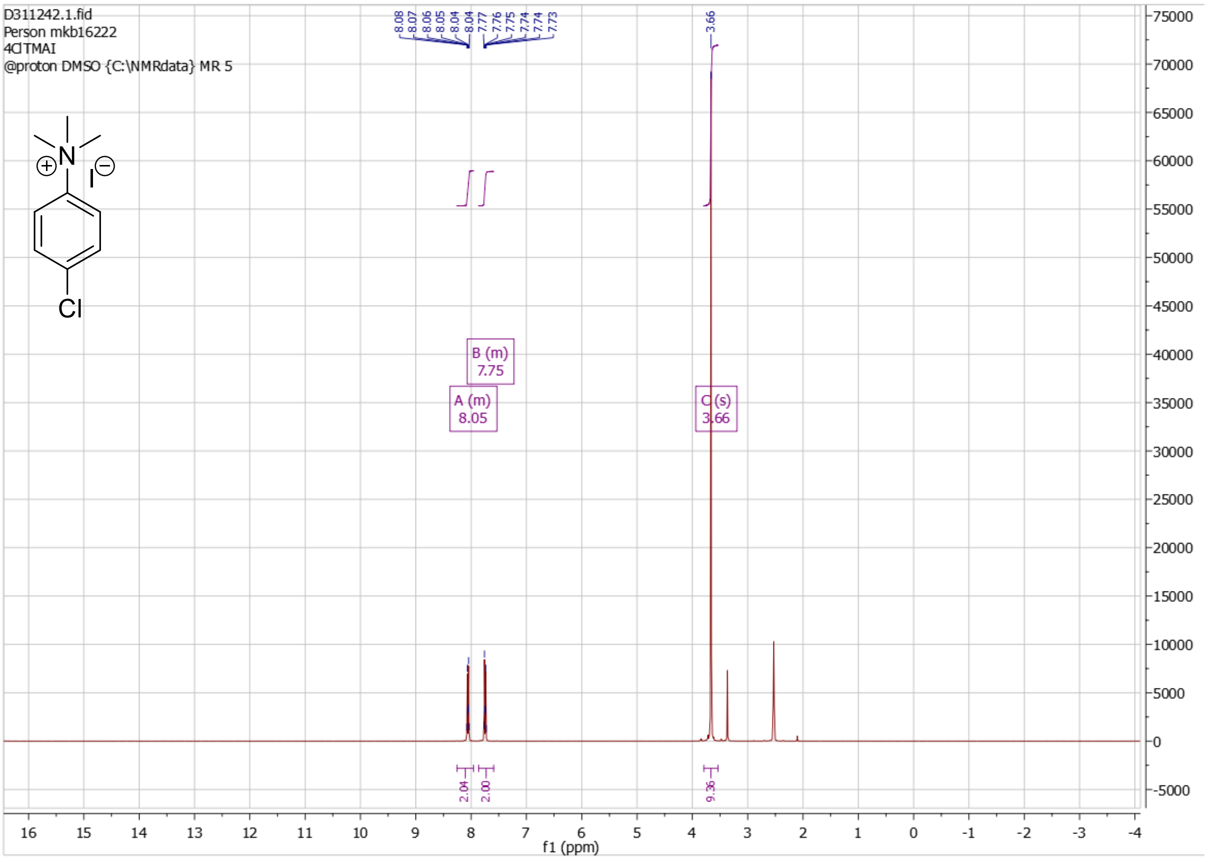
D311242.1.fid
Person mkb16222
4ClTMAI
@proton DMSO {C:\NMRdata} MR 5
8.08
8.07
8.06
8.05
8.04
8.04
7.77
7.76
7.75
7.74
7.74
7.73
3.66
B (m)
7.75
A (m)
8.05
C (s)
3.66
2.04
2.00
9.36
f1 (ppm)

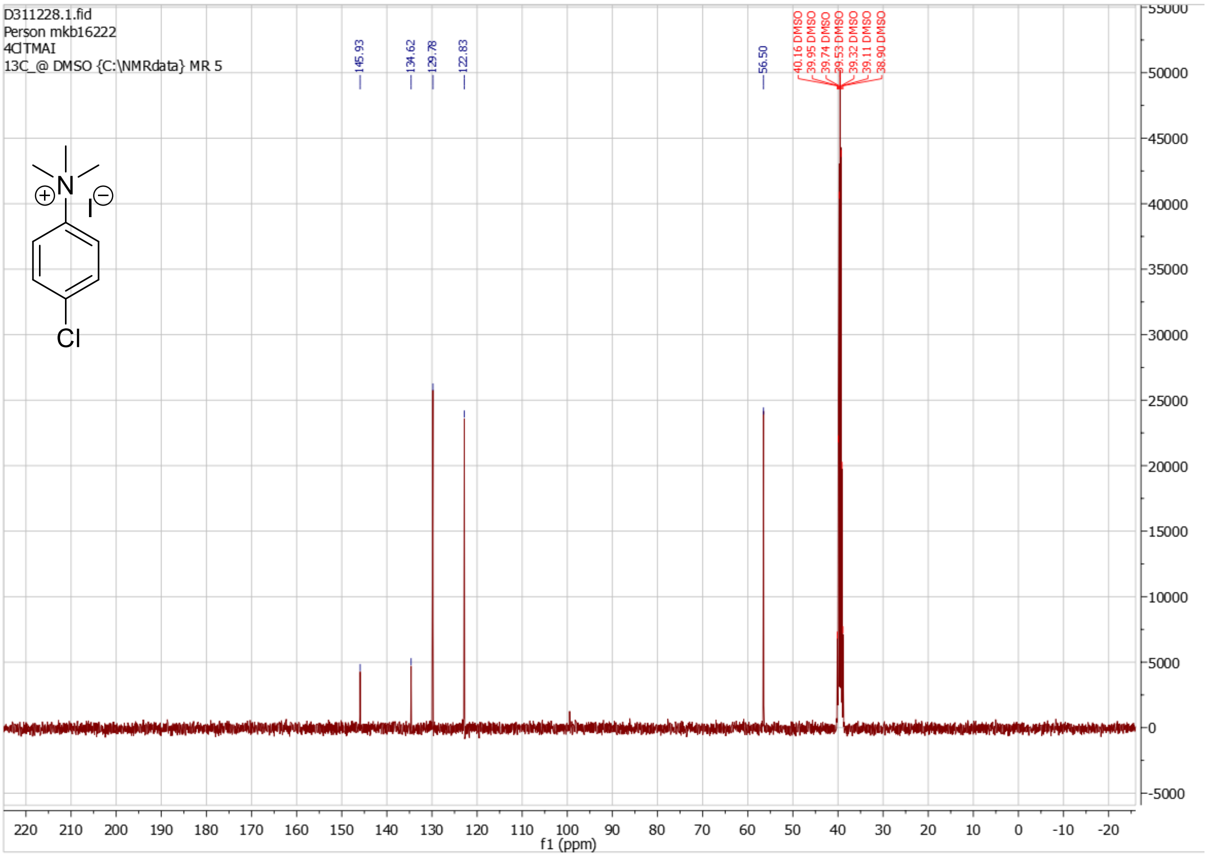
D311228.1.fid
Person mkb16222
4ClTMAI
13C_@ DMSO {C:\NMRdata} MR 5
145.93
134.62
129.78
122.83
56.50
40.16 DMSO
39.95 DMSO
39.74 DMSO
39.53 DMSO
39.32 DMSO
39.11 DMSO
38.90 DMSO
f1 (ppm)

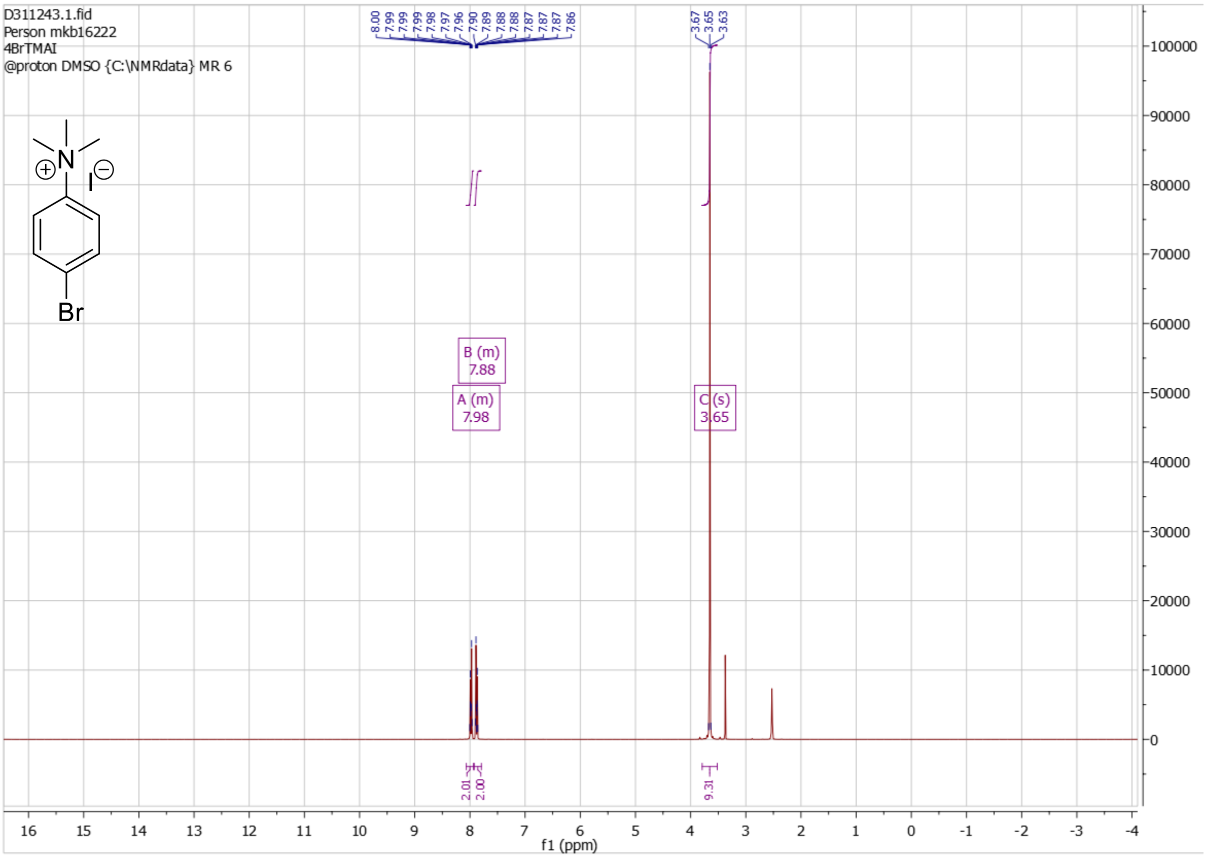
D311243.1.fid
Person mkb16222
4BrTMAI
@proton DMSO {C:\NMRdata} MR 6
N
I
Br
B (m)
7.88
A (m)
7.98
C (s)
3.65
2.01
2.00
9.31
f1 (ppm)

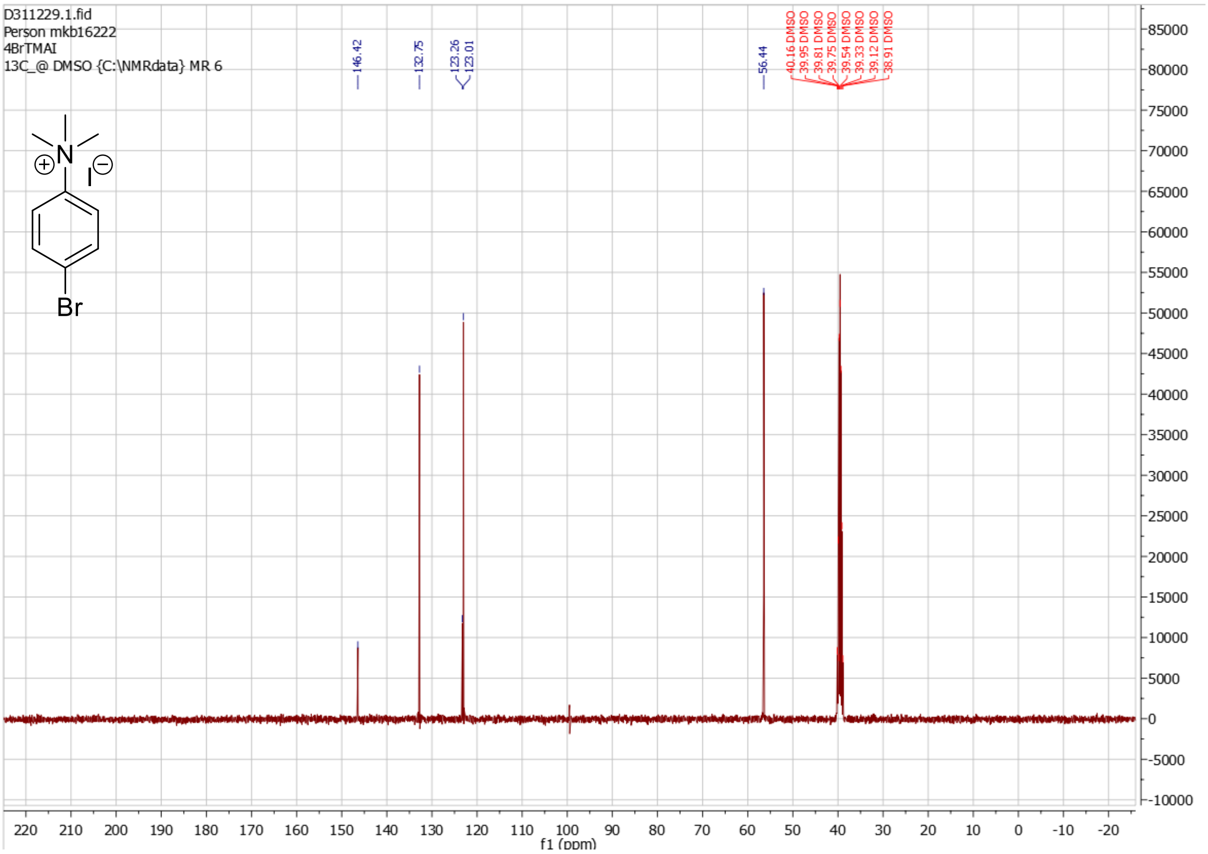
D311229.1.fid
Person mkb16222
4BrTMAI
13C_@ DMSO {C:\NMRdata} MR 6
N
I
Br
146.42
132.75
123.26
123.01
56.44
f1 (ppm)

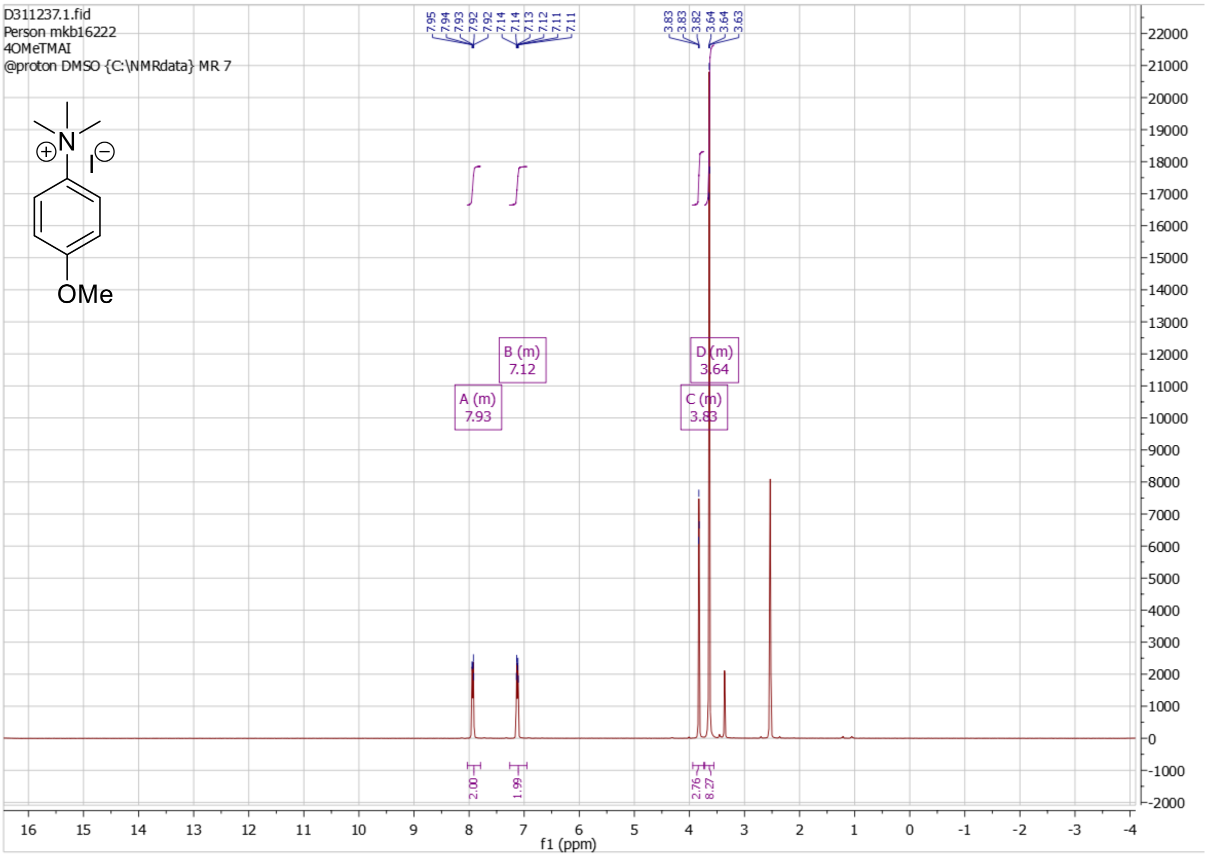
D311237.1.fid
Person mkb16222
4OMeTMAI
@proton DMSO {C:\NMRdata} MR 7
OMe
A (m)
7.93
B (m)
7.12
C (m)
3.83
D (m)
3.64
2.00
1.99
2.76
8.27
f1 (ppm)

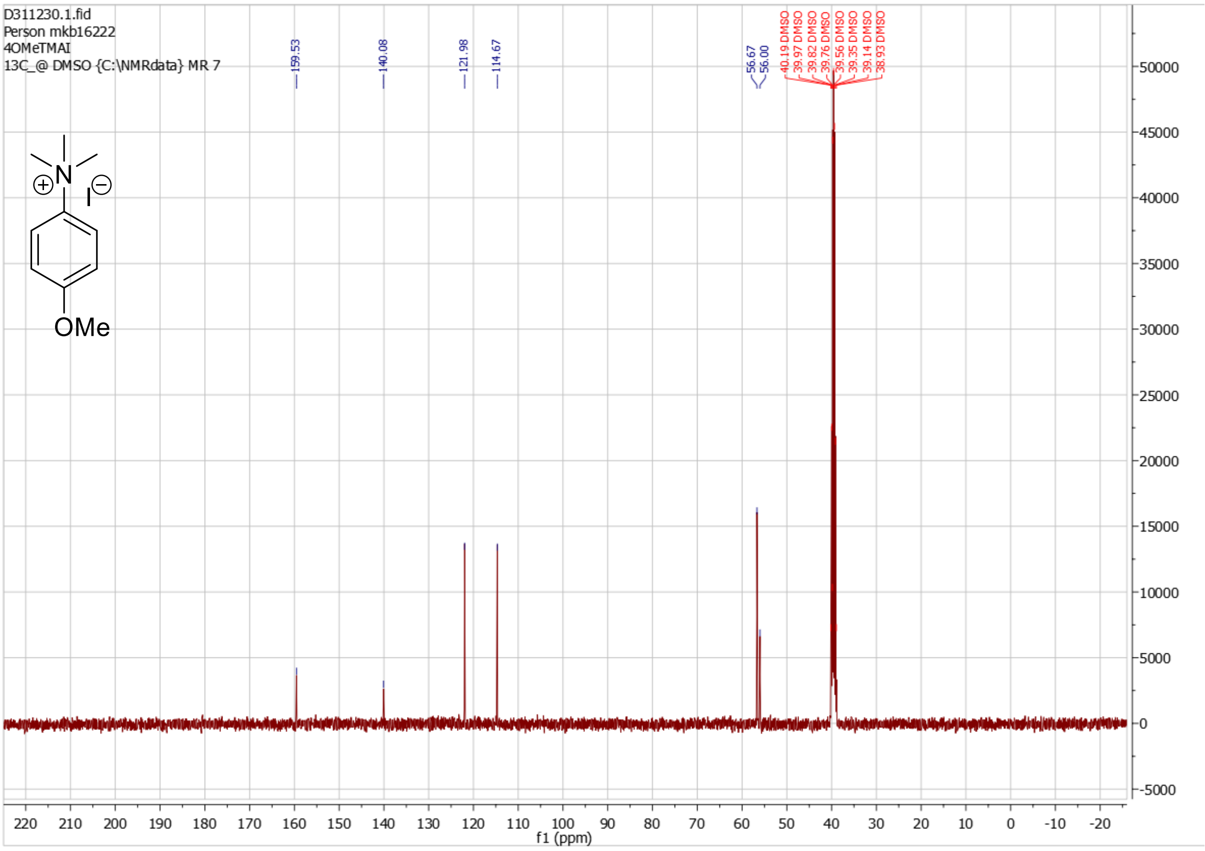
D311230.1.fid
Person mkb16222
4OMeTMAI
13C_@ DMSO {C:\NMRdata} MR 7
OMe
159.53
140.08
121.98
114.67
56.67
56.00
40.19 DMSO
39.97 DMSO
39.82 DMSO
39.76 DMSO
39.56 DMSO
39.35 DMSO
39.14 DMSO
38.93 DMSO
f1 (ppm)

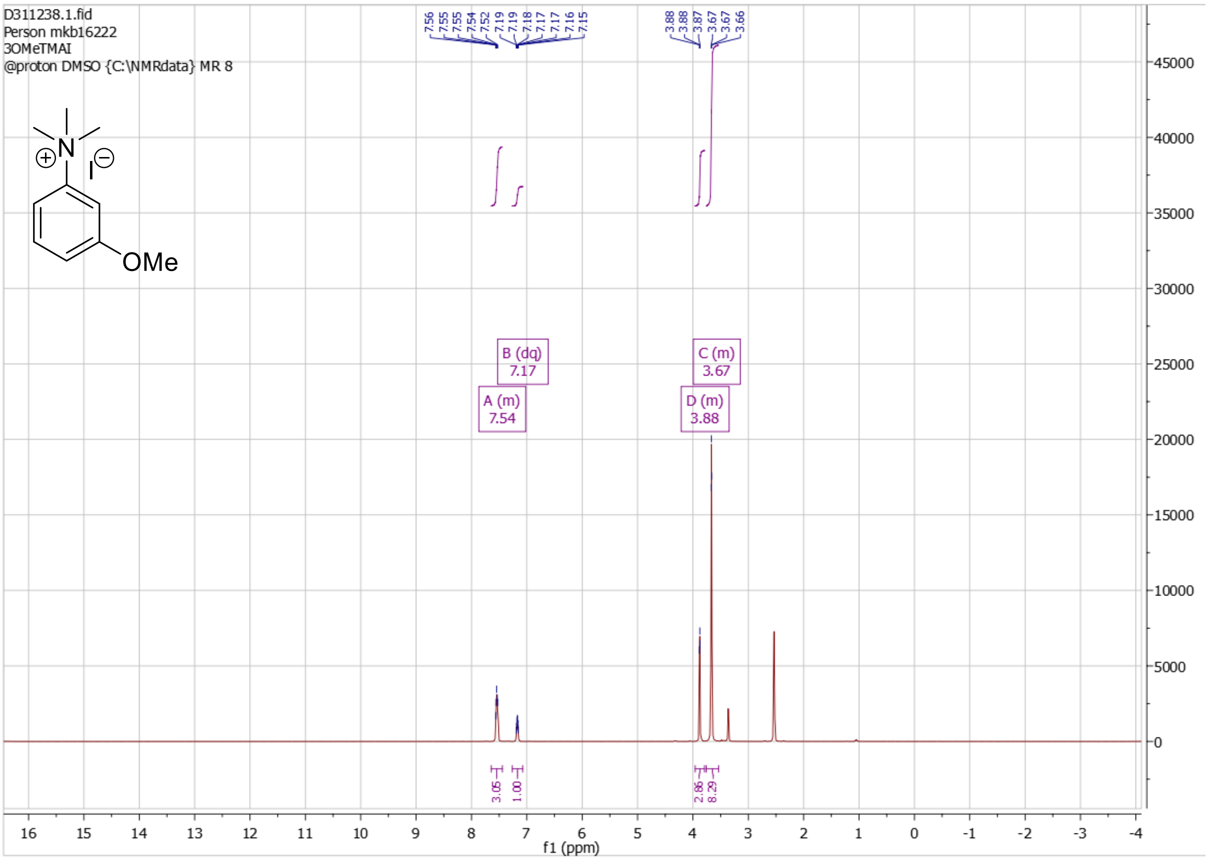
D311238.1.fid
Person mkb16222
3OMeTMAI
@proton DMSO {C:\NMRdata} MR 8
OMe
A (m)
7.54
B (dq)
7.17
C (m)
3.67
D (m)
3.88
3.05
1.00
2.86
8.29
f1 (ppm)

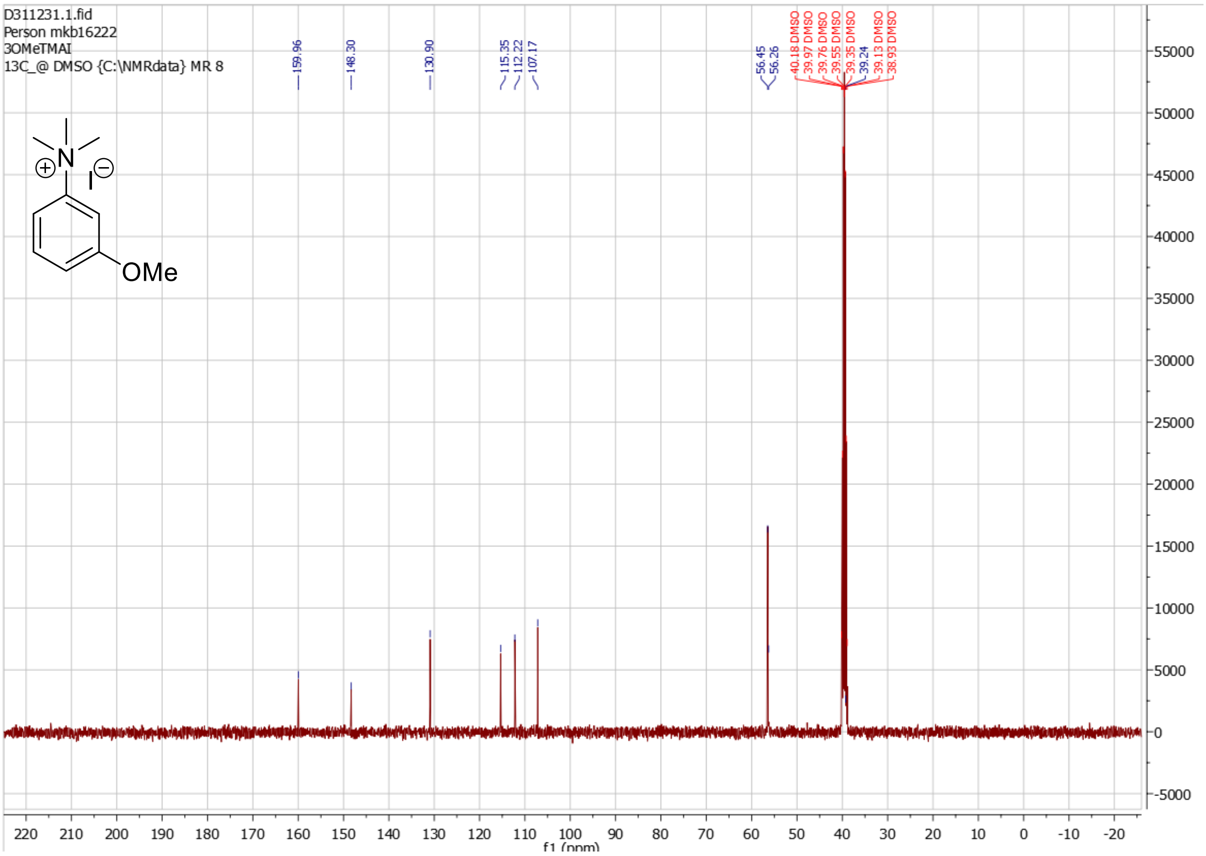
D311231.1.fid
Person mkb16222
3OMeTMAI
13C_@ DMSO {C:\NMRdata} MR 8
OMe
159.96
148.30
130.90
115.35
112.22
107.17
56.45
56.26
39.24
f1 (ppm)

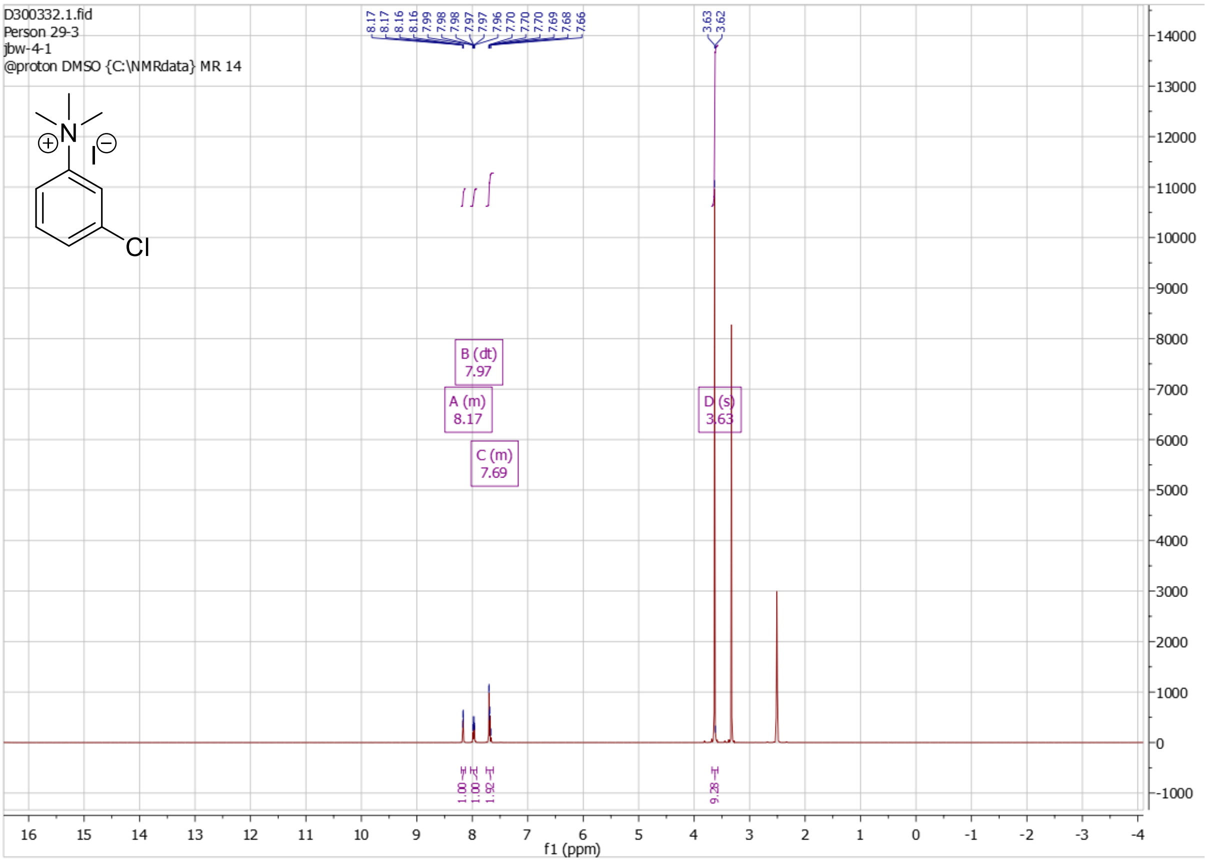
D300332.1.fid
Person 29-3
jbw-4-1
@proton DMSO {C:\NMRdata} MR 14
Cl
B (dt) 7.97
A (m) 8.17
C (m) 7.69
D (s) 3.63
f1 (ppm)

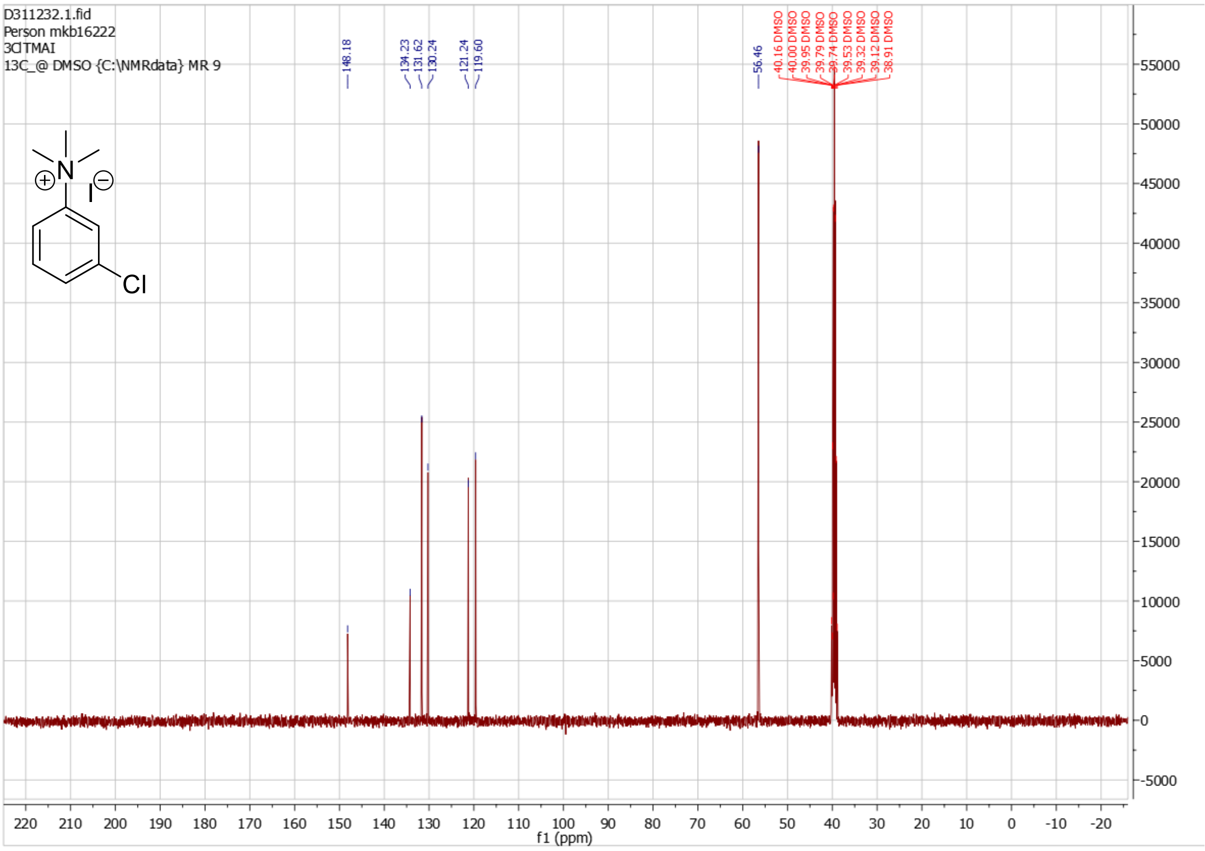
D311232.1.fid
Person mkb16222
3ClTMAI
13C_@ DMSO {C:\NMRdata} MR 9
148.18
134.23
131.62
130.24
121.24
119.60
56.46
Cl
f1 (ppm)

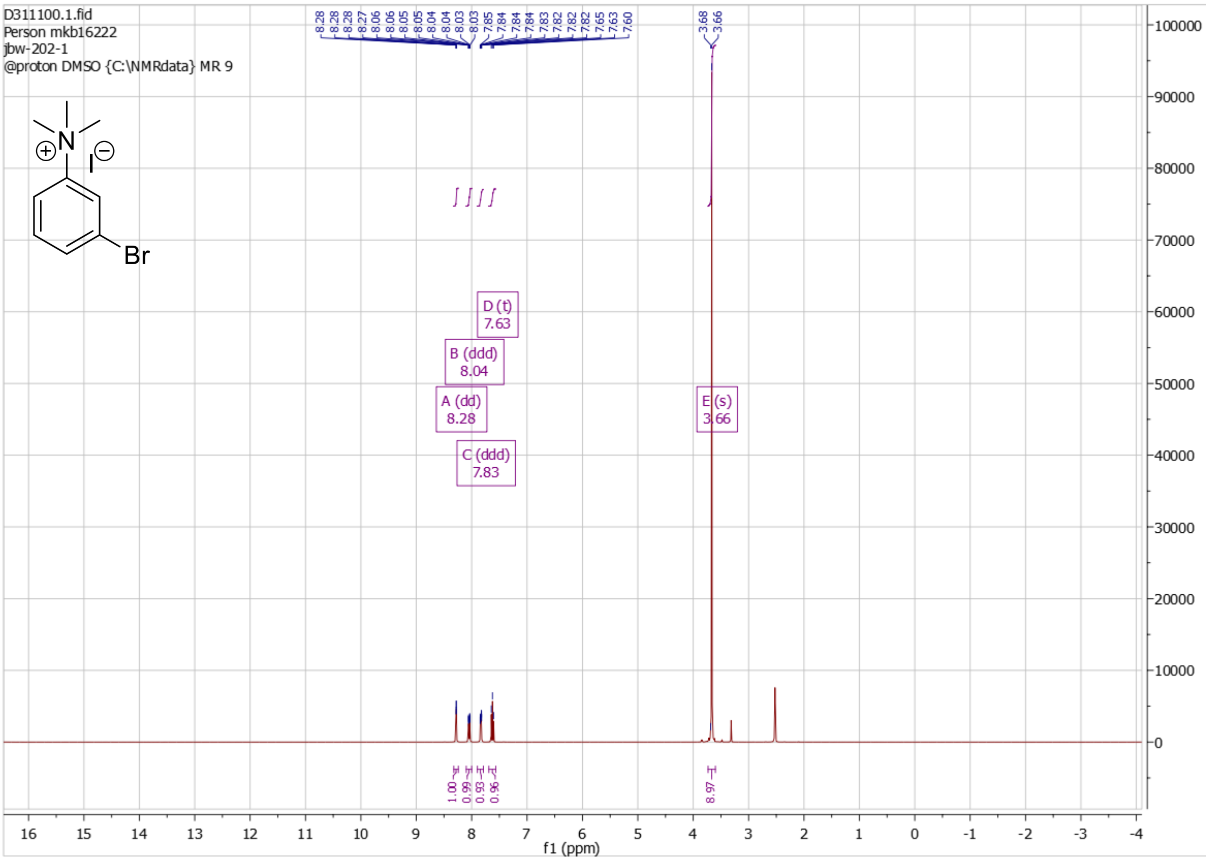
D311100.1.fid
Person mkb16222
jbw-202-1
@proton DMSO {C:\NMRdata} MR 9
8.28 8.28 8.28 8.27 8.06 8.06 8.05 8.05 8.04 8.04 8.03 8.03 7.85 7.84 7.84 7.84 7.83 7.82 7.82 7.82 7.65 7.63 7.60
3.68 3.66
N
I
Br
D (t) 7.63
B (ddd) 8.04
A (dd) 8.28
C (ddd) 7.83
E (s) 3.66
1.00 0.99 0.93 0.96
8.97
f1 (ppm)

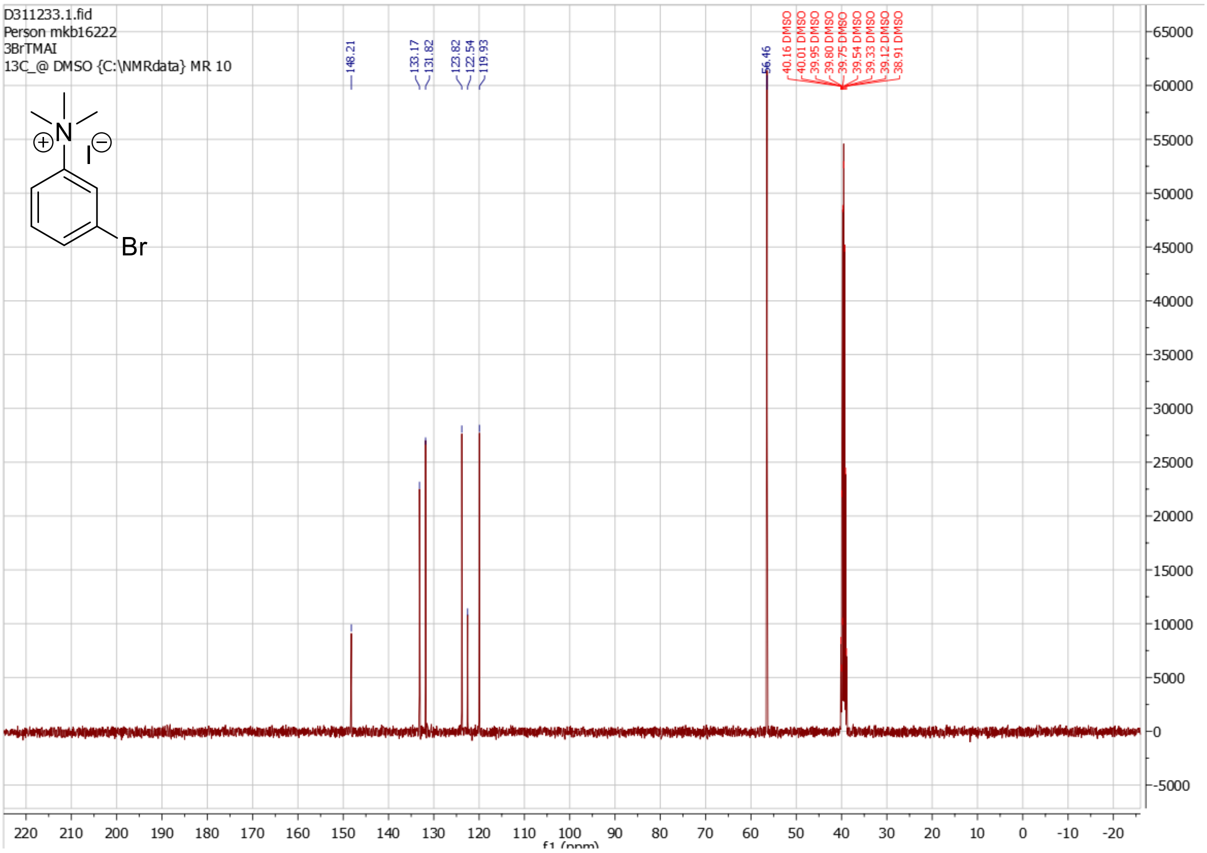
D311233.1.fid
Person mkb16222
3BrTMAI
13C_@ DMSO {C:\NMRdata} MR 10
148.21
133.17 131.82
123.82 122.54 119.93
56.46
40.16 DMSO 40.01 DMSO 39.95 DMSO 39.80 DMSO 39.75 DMSO 39.54 DMSO 39.33 DMSO 39.12 DMSO 38.91 DMSO
N
I
Br
f1 (ppm)

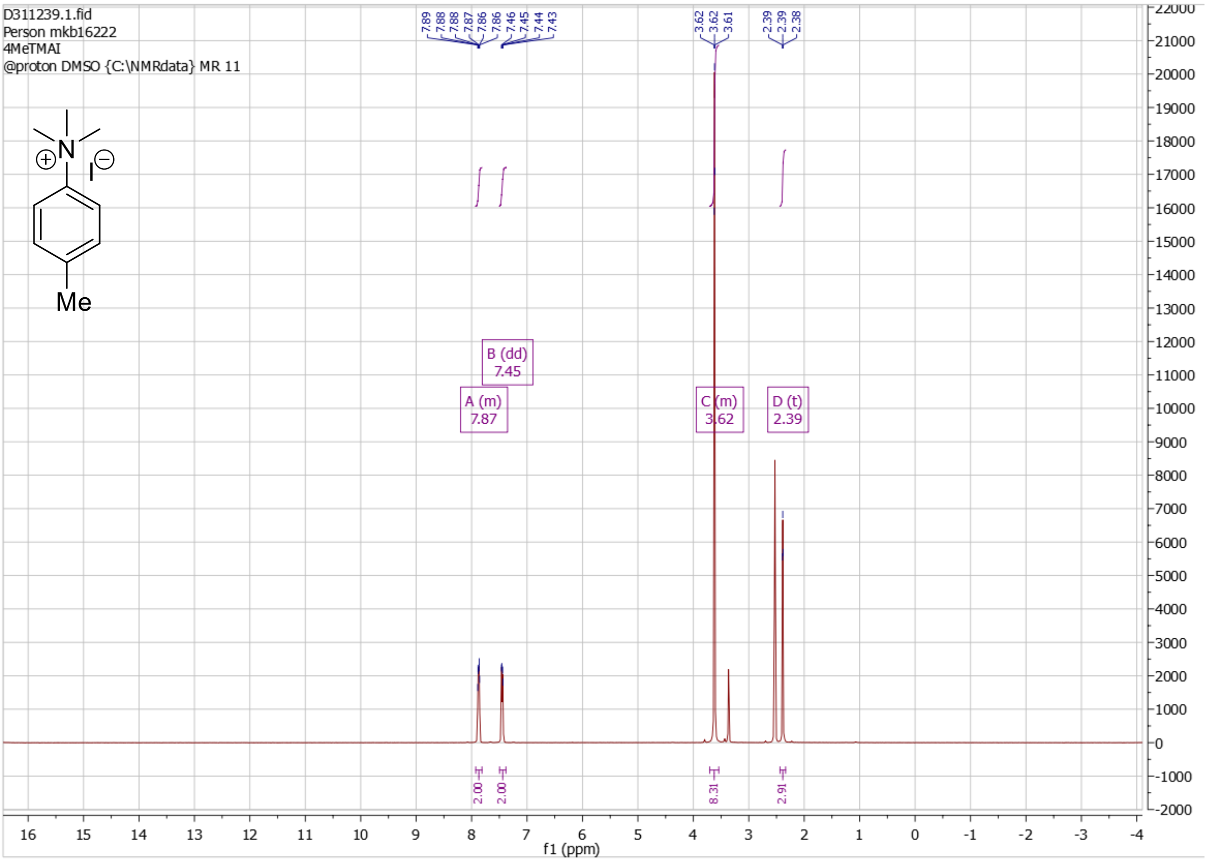
D311239.1.fid
Person mkb16222
4MeTMAI
@proton DMSO {C:\NMRdata} MR 11
N
I
Me
B (dd)
7.45
A (m)
7.87
C (m)
3.62
D (t)
2.39
2.00
2.00
8.31
2.91
f1 (ppm)

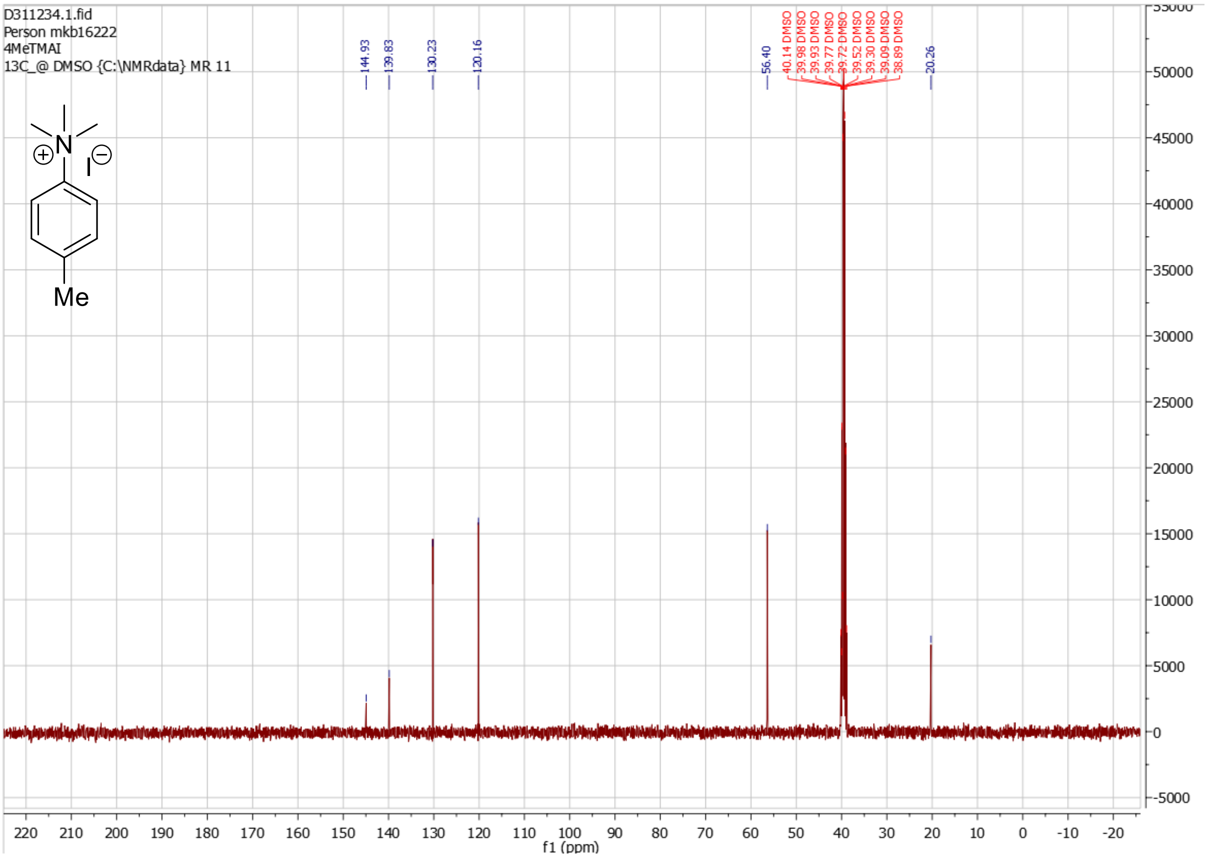
D311234.1.fid
Person mkb16222
4MeTMAI
13C_@ DMSO {C:\NMRdata} MR 11
144.93
139.83
130.23
120.16
56.40
40.14 DMSO
39.98 DMSO
39.93 DMSO
39.77 DMSO
39.72 DMSO
39.52 DMSO
39.30 DMSO
39.09 DMSO
38.89 DMSO
20.26
N
I
Me
f1 (ppm)

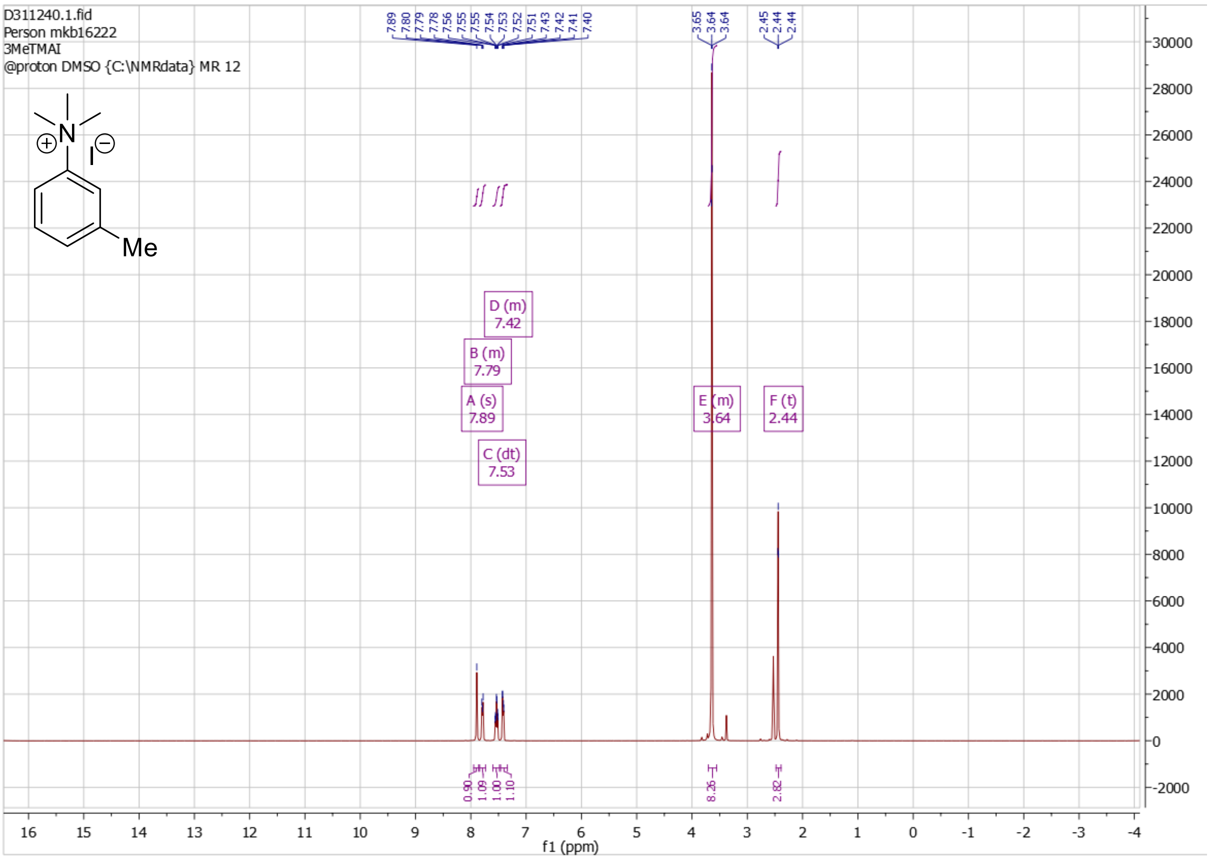
D311240.1.fid
Person mkb16222
3MeTMAI
@proton DMSO {C:\NMRdata} MR 12
Me
A (s) 7.89
B (m) 7.79
C (dt) 7.53
D (m) 7.42
E (m) 3.64
F (t) 2.44
f1 (ppm)

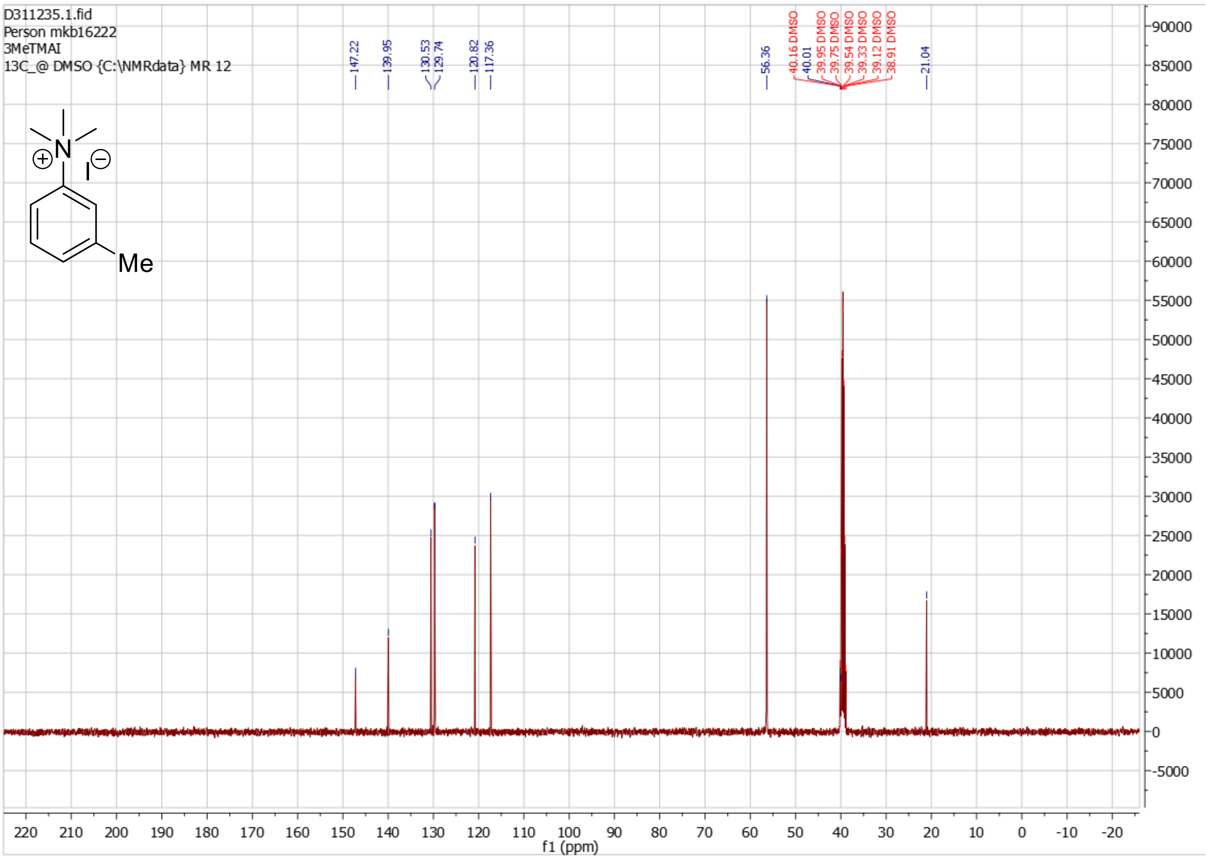
D311235.1.fid
Person mkb16222
3MeTMAI
13C_@ DMSO {C:\NMRdata} MR 12
Me
147.22
139.95
130.53
129.74
120.82
117.36
56.36
21.04
f1 (ppm)

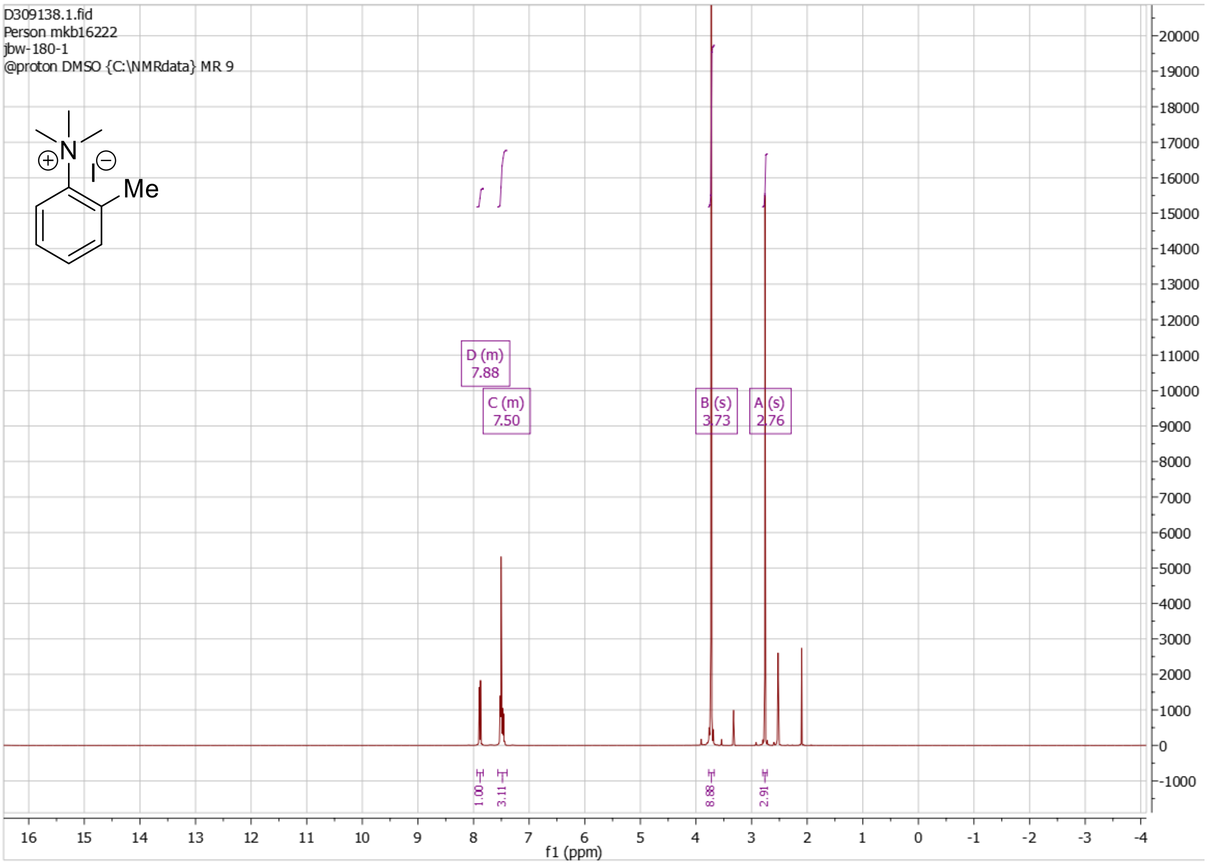
D309138.1.fid
Person mkb16222
jbw-180-1
@proton DMSO {C:\NMRdata} MR 9
Me
D (m)
7.88
C (m)
7.50
B (s)
3.73
A (s)
2.76
1.00
3.11
8.88
2.91
f1 (ppm)

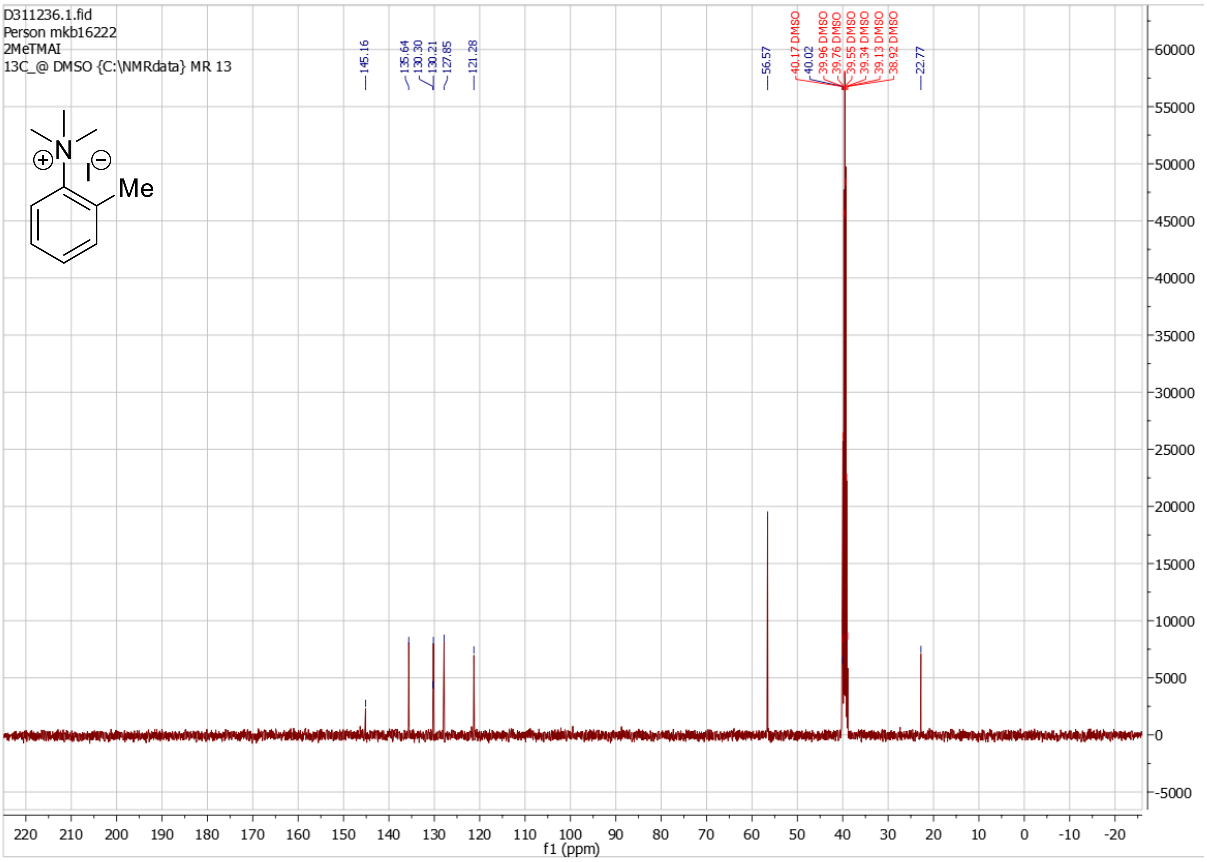
D311236.1.fid
Person mkb16222
2MeTMAI
13C_@ DMSO {C:\NMRdata} MR 13
Me
145.16
135.64
130.30
130.21
127.85
121.28
56.57
40.17 DMSO
40.02
39.96 DMSO
39.76 DMSO
39.55 DMSO
39.34 DMSO
39.13 DMSO
38.92 DMSO
22.77
f1 (ppm)

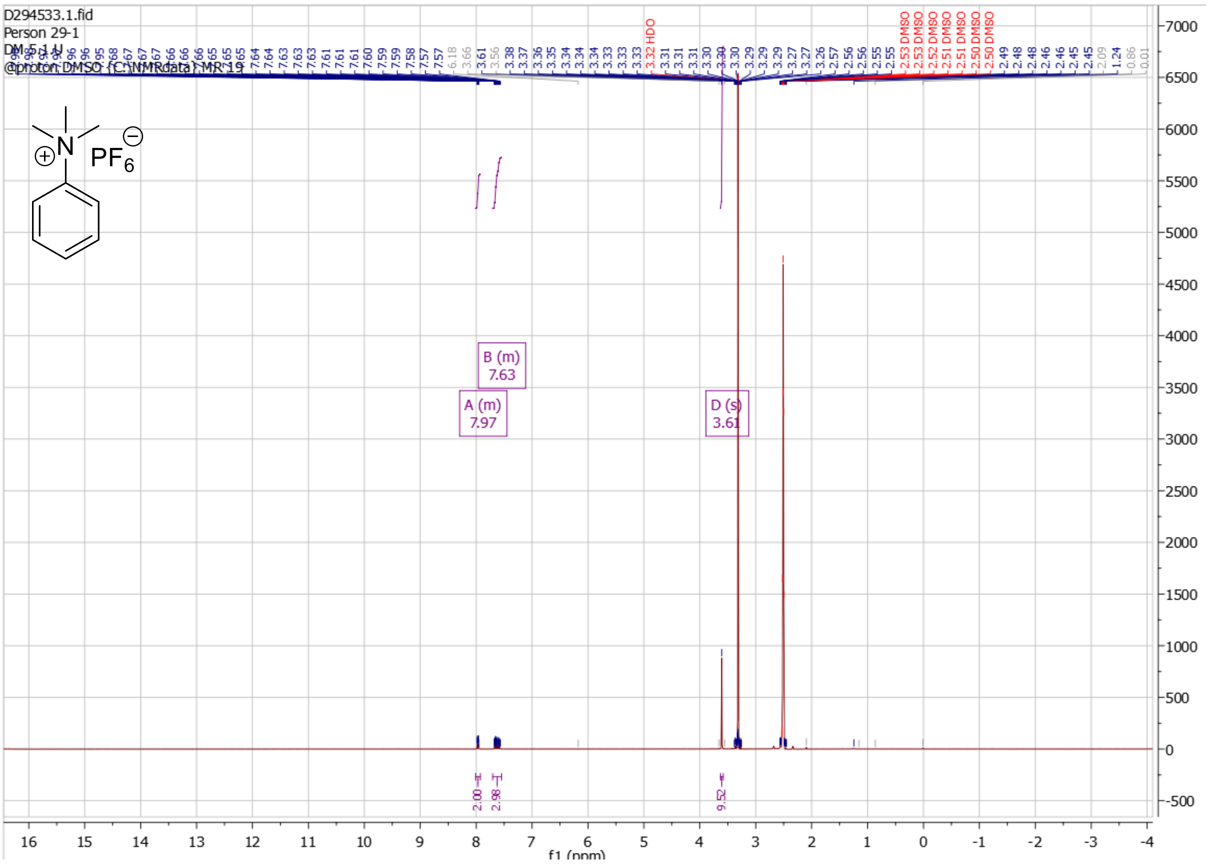
D294533.1.fid
Person 29-1
@proton DMSO {C:\NMRdata} MR 19
3.32 HDO
2.53 DMSO
2.50 DMSO
PF6
N
B (m)
7.63
A (m)
7.97
D (s)
3.61
2.00
2.98
9.52
f1 (ppm)

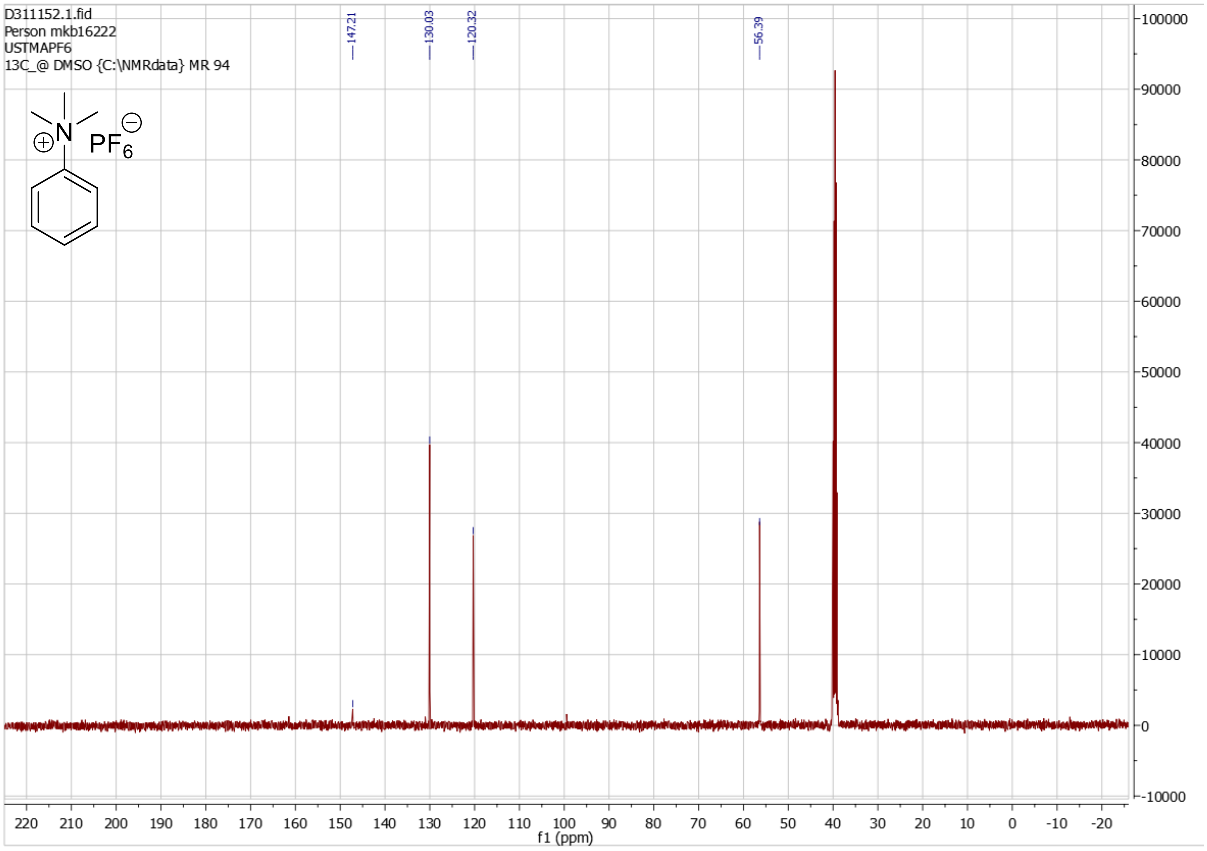
D311152.1.fid
Person mkb16222
USTMAPF6
13C_@ DMSO {C:\NMRdata} MR 94
147.21
130.03
120.32
56.39
PF6
N
f1 (ppm)

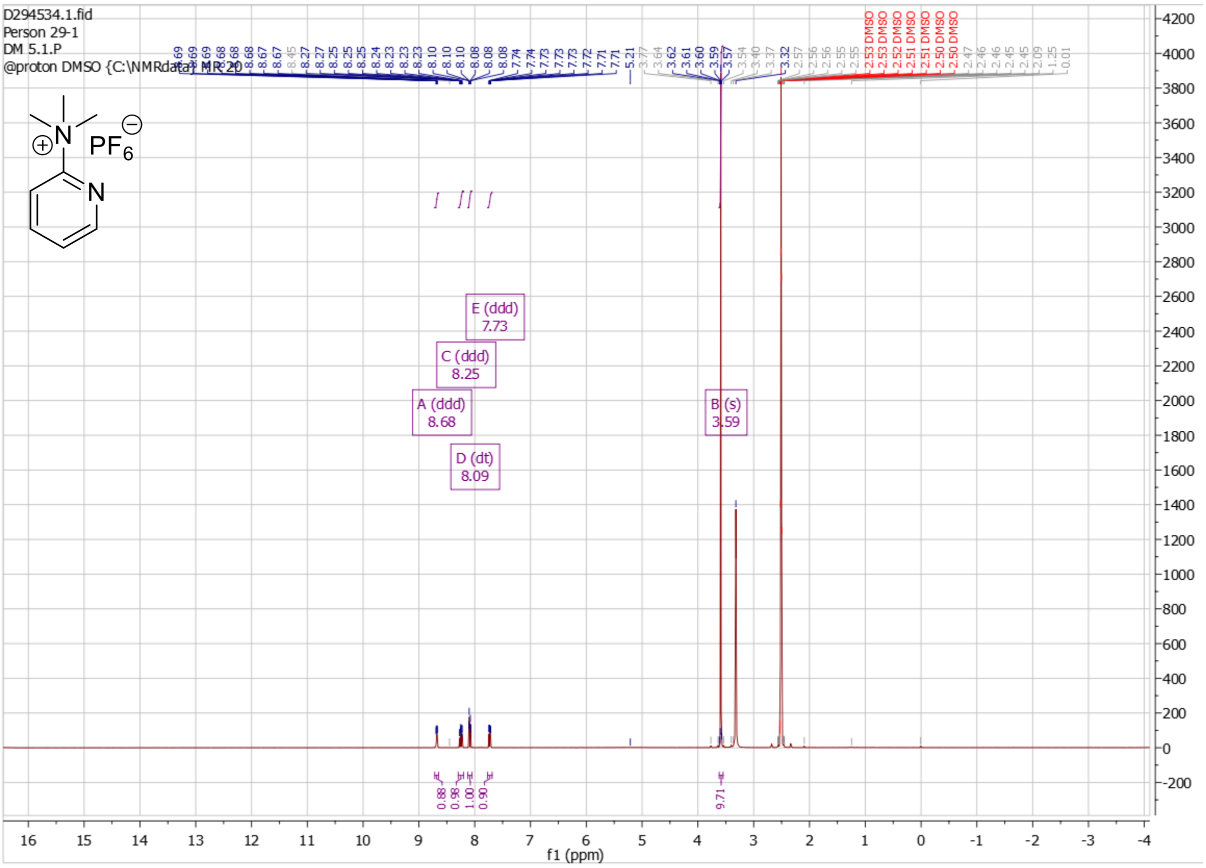

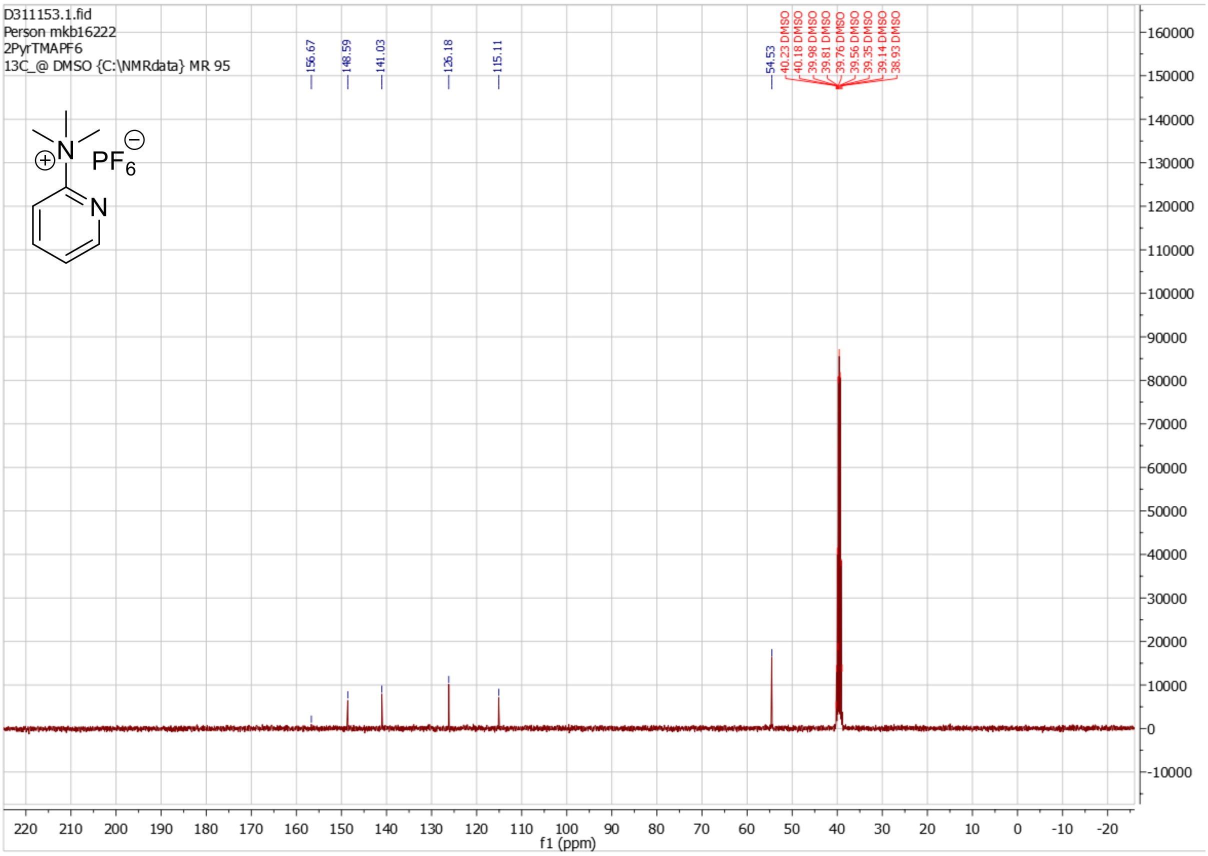

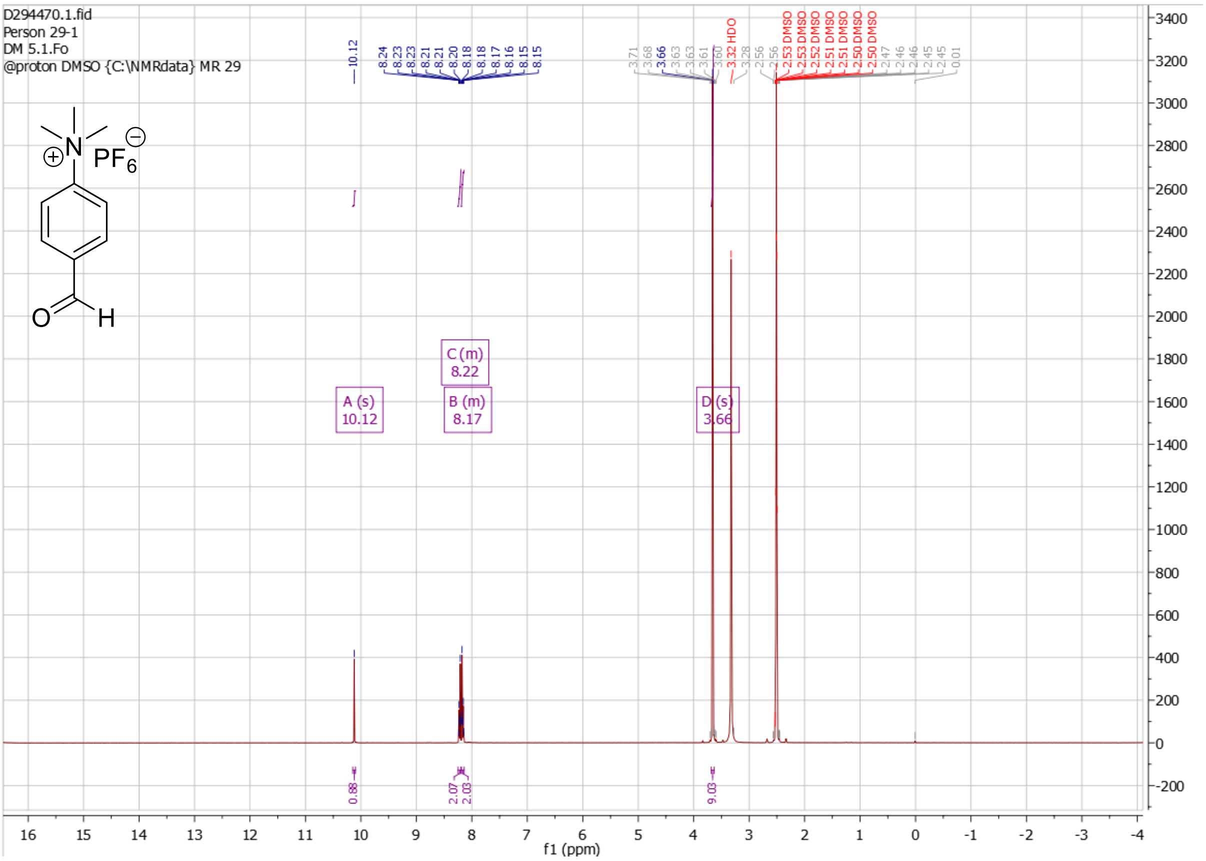
D294470.1.fid
Person 29-1
DM 5.1.Fo
@proton DMSO {C:\NMRdata} MR 29
A (s) 10.12
B (m) 8.17
C (m) 8.22
D (s) 3.66
3.32 HDO
f1 (ppm)

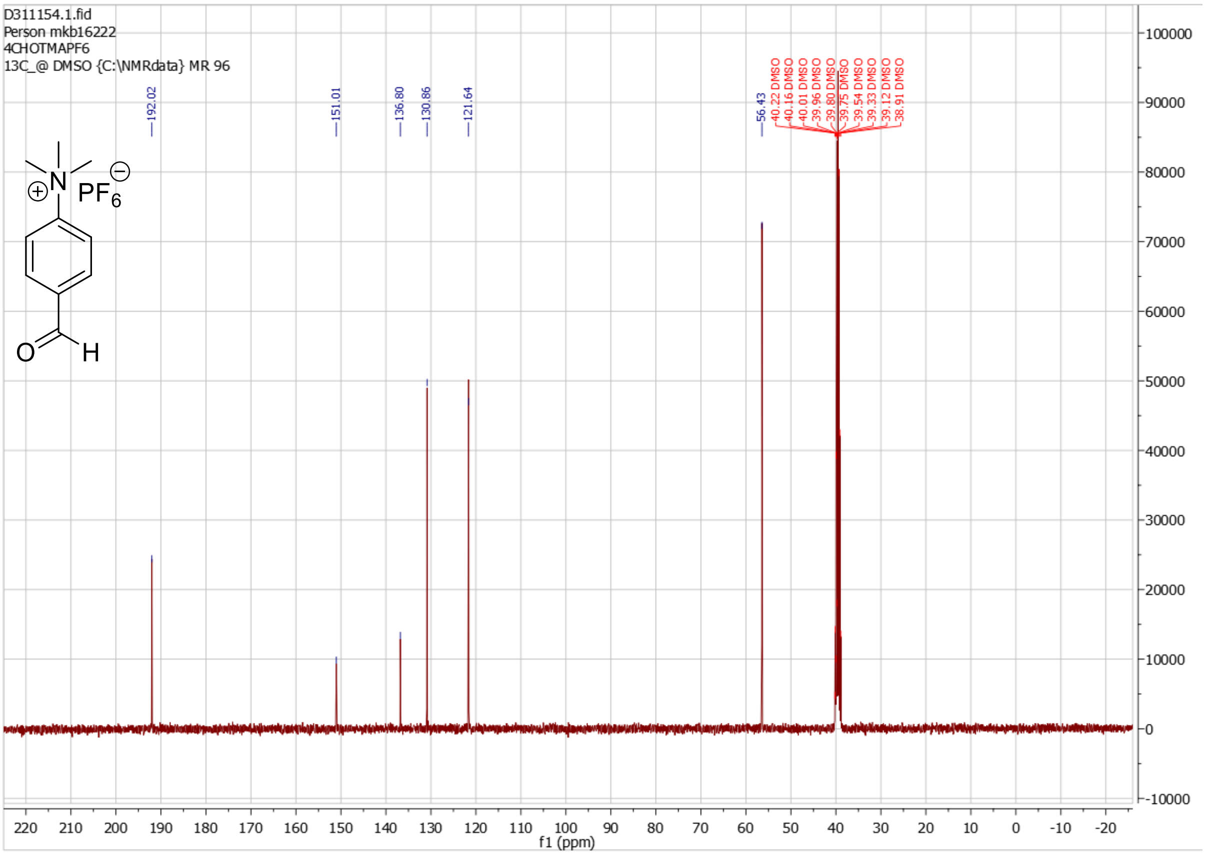
D311154.1.fid
Person mkb16222
4CHOTMAPF6
13C_@ DMSO {C:\NMRdata} MR 96
192.02
151.01
136.80
130.86
121.64
56.43
f1 (ppm)

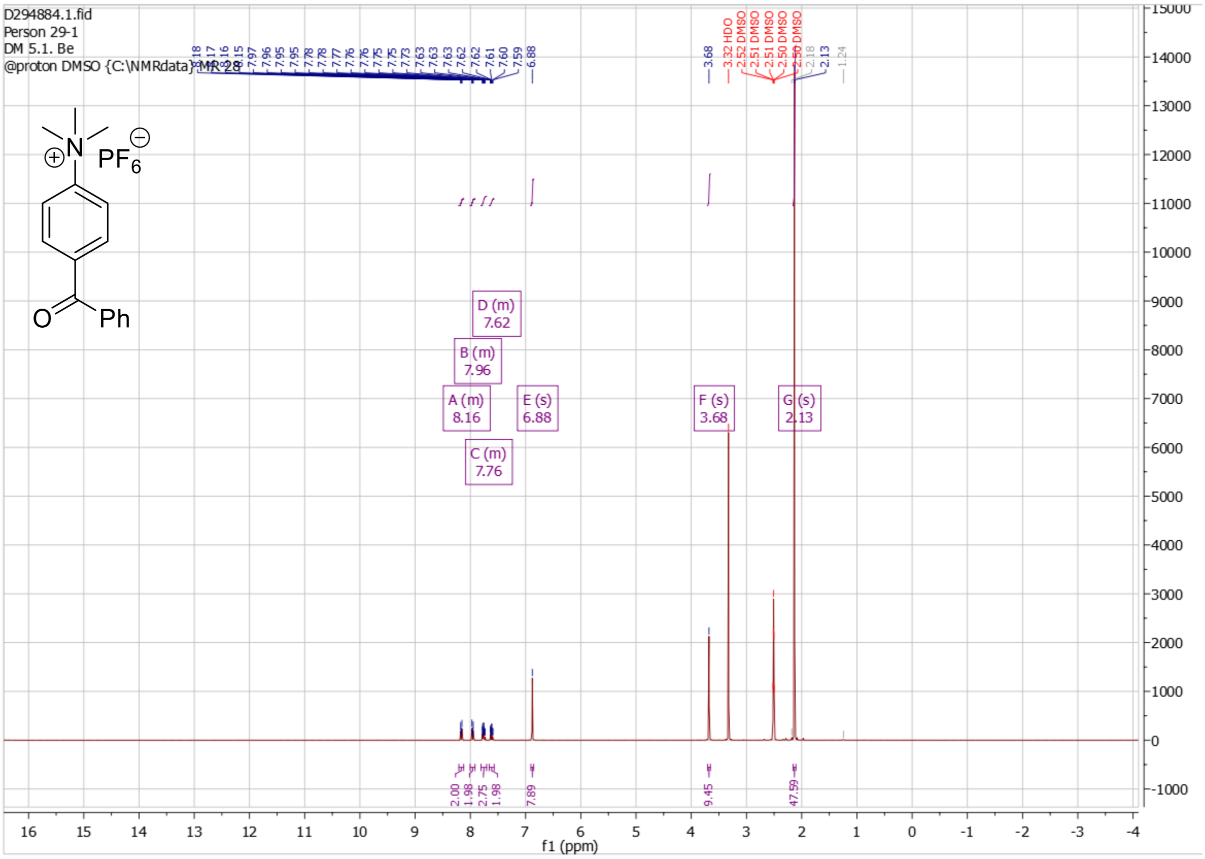
D294884.1.fid
Person 29-1
DM 5.1. Be
@proton DMSO {C:\NMRdata} MR 28
A (m) 8.16
B (m) 7.96
C (m) 7.76
D (m) 7.62
E (s) 6.88
F (s) 3.68
G (s) 2.13
f1 (ppm)

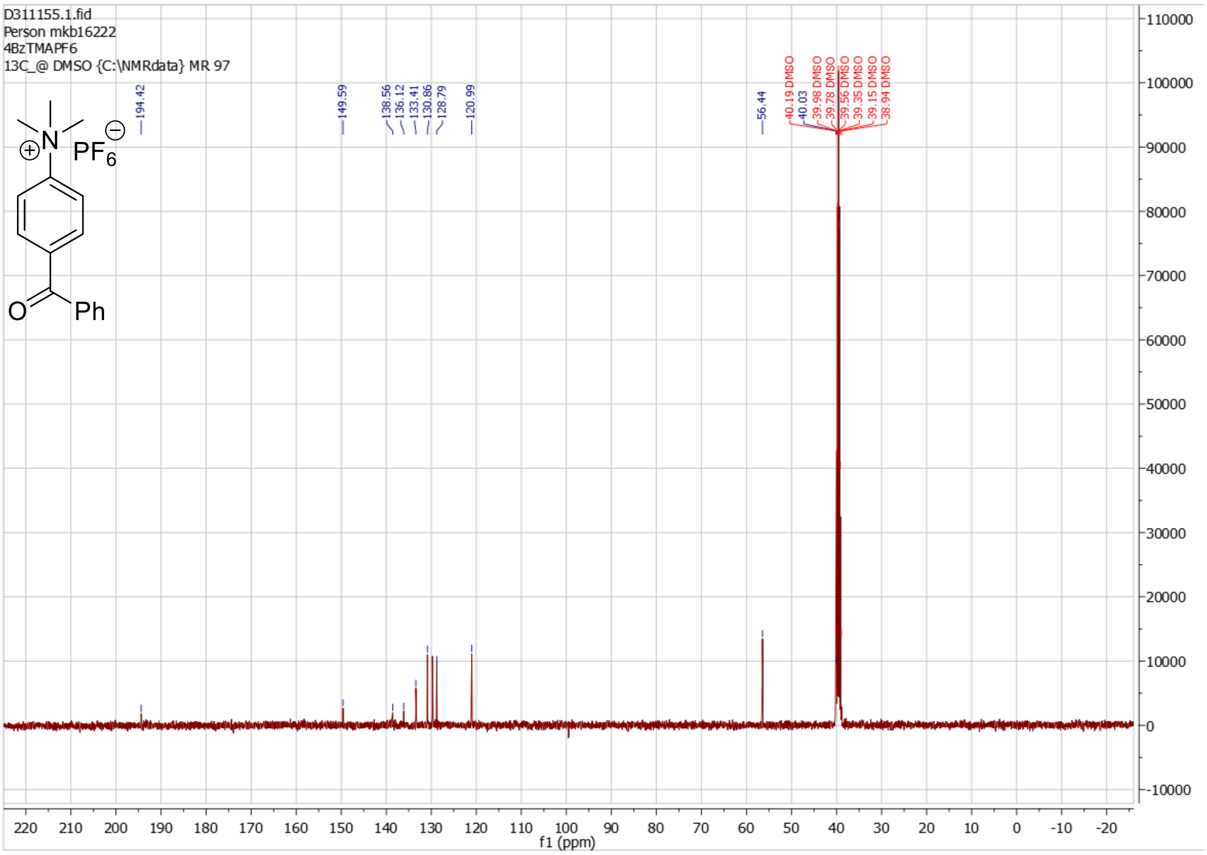
D311155.1.fid
Person mkb16222
4BzTMAPF6
13C_@ DMSO {C:\NMRdata} MR 97
194.42
149.59
138.56
136.12
133.41
130.86
128.79
120.99
56.44
f1 (ppm)

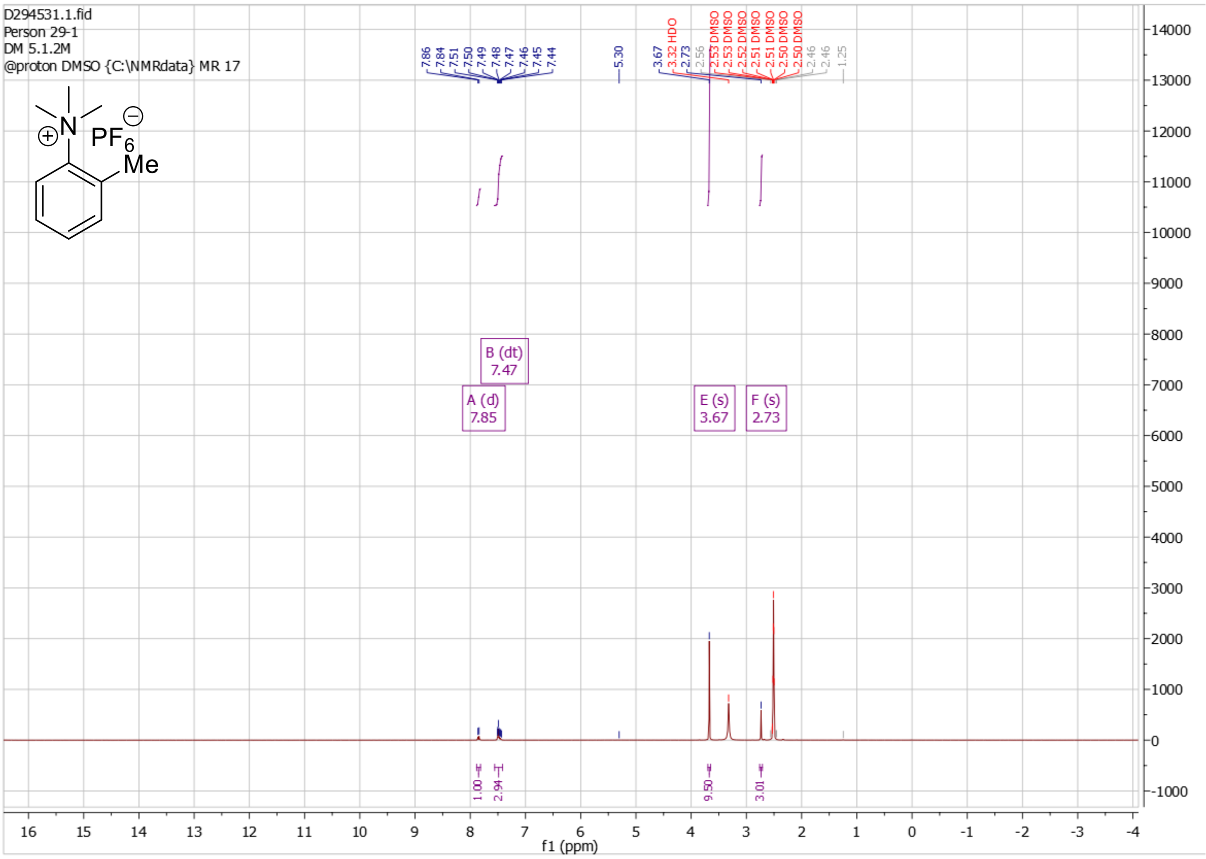
D294531.1.fid
Person 29-1
DM 5.1.2M
@proton DMSO {C:\NMRdata} MR 17
N
PF6
Me
7.86
7.84
7.51
7.50
7.49
7.48
7.47
7.46
7.45
7.44
5.30
3.67
3.32 HDO
2.73
2.56
2.53 DMSO
2.53 DMSO
2.52 DMSO
2.51 DMSO
2.51 DMSO
2.50 DMSO
2.50 DMSO
2.46
2.46
1.25
B (dt)
7.47
A (d)
7.85
E (s)
3.67
F (s)
2.73
1.00
2.94
9.50
3.01
f1 (ppm)

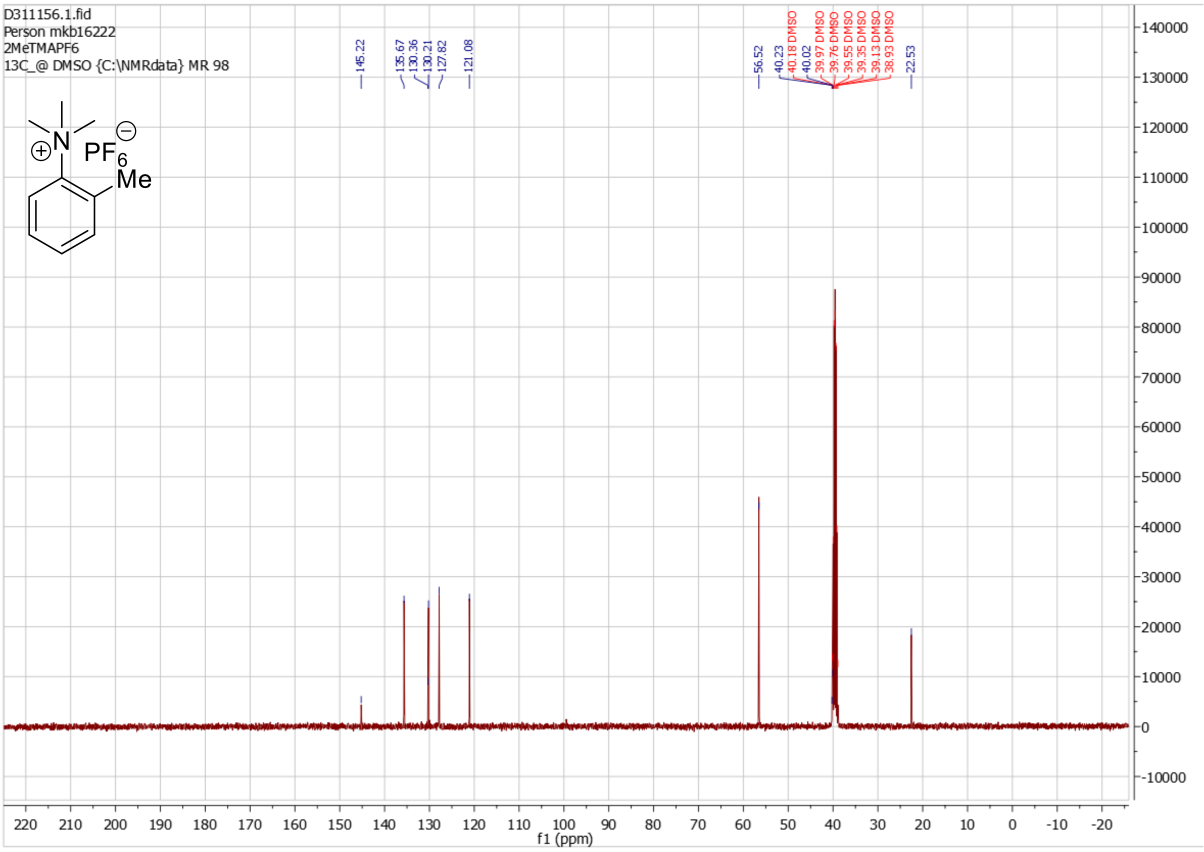
D311156.1.fid
Person mkb16222
2MeTMAPF6
13C_@ DMSO {C:\NMRdata} MR 98
N
PF6
Me
145.22
135.67
130.36
130.21
127.82
121.08
56.52
40.23
40.18 DMSO
40.02
39.97 DMSO
39.76 DMSO
39.55 DMSO
39.35 DMSO
39.13 DMSO
38.93 DMSO
22.53
f1 (ppm)

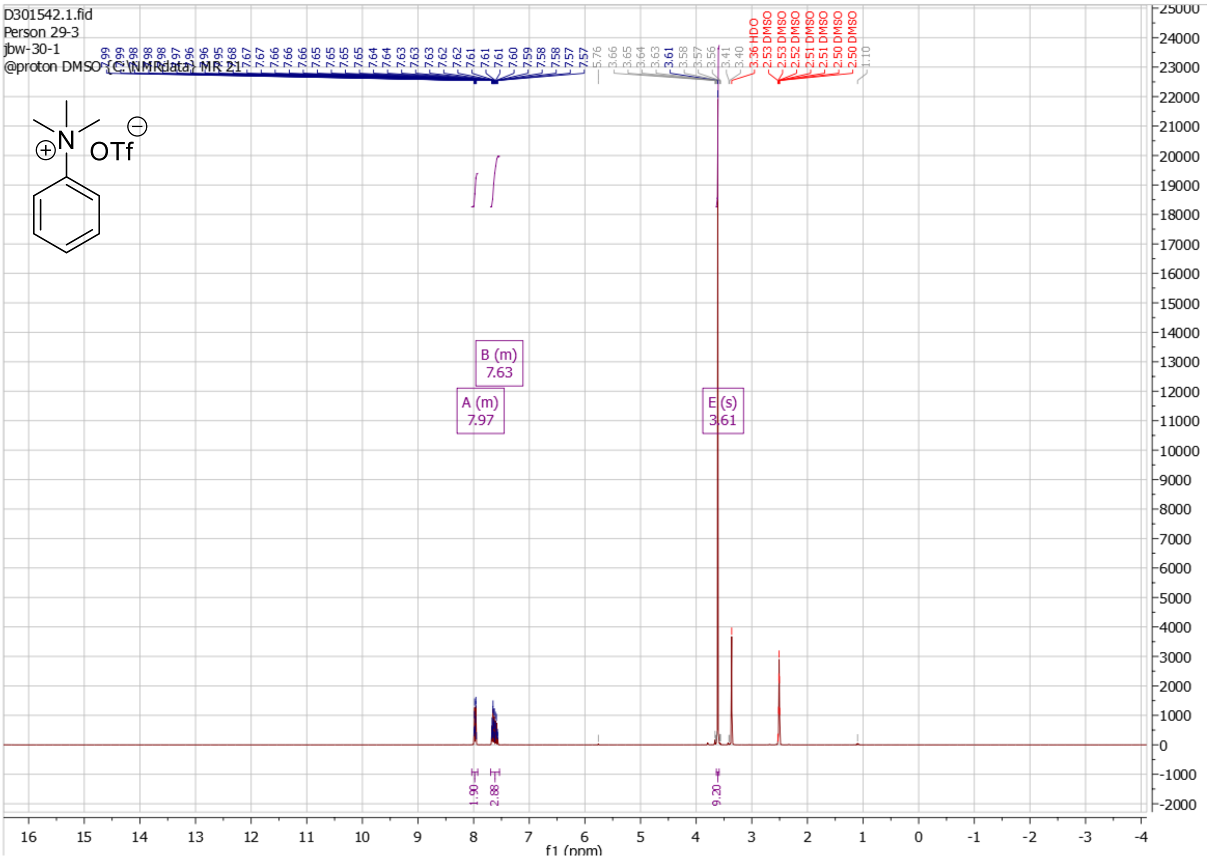
D301542.1.fid
Person 29-3
jbw-30-1
@proton DMSO {C:\NMRdata} MR 21
OTf
B (m)
7.63
A (m)
7.97
E (s)
3.61
f1 (ppm)

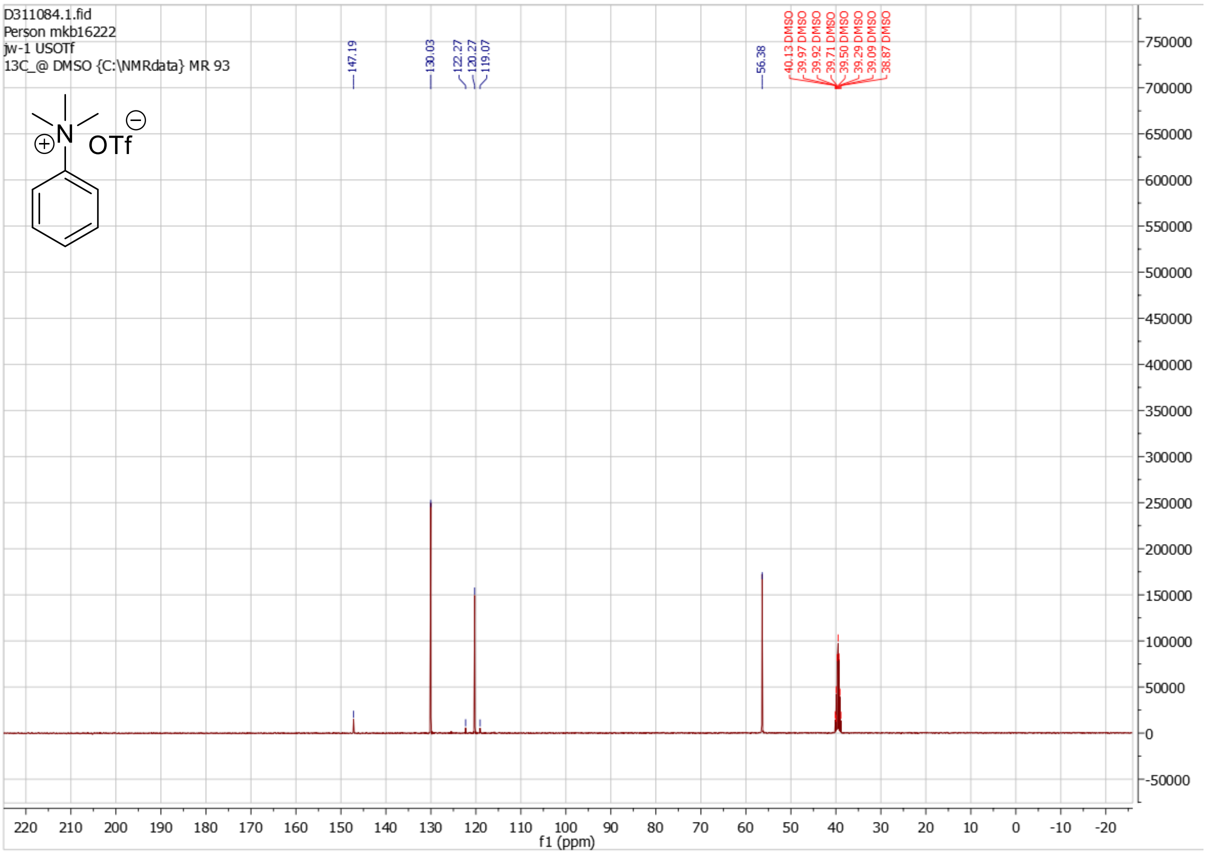
D311084.1.fid
Person mkb16222
jw-1 USOTf
13C_@ DMSO {C:\NMRdata} MR 93
OTf
147.19
130.03
122.27
120.27
119.07
56.38
f1 (ppm)

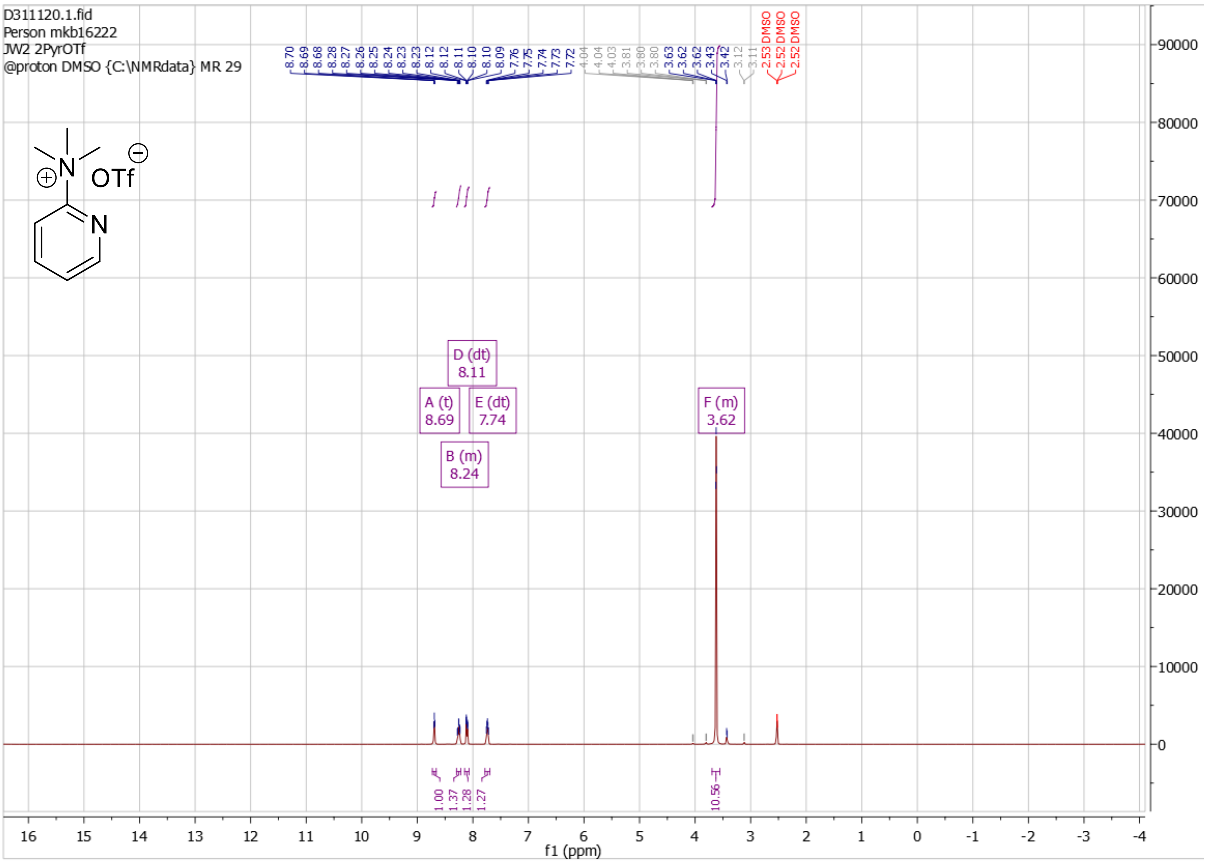
D311120.1.fid
Person mkb16222
JW2 2PyrOTf
@proton DMSO {C:\NMRdata} MR 29
OTf
N
D (dt)
8.11
A (t)
8.69
E (dt)
7.74
F (m)
3.62
B (m)
8.24
1.00
1.37
1.28
1.27
10.56
f1 (ppm)

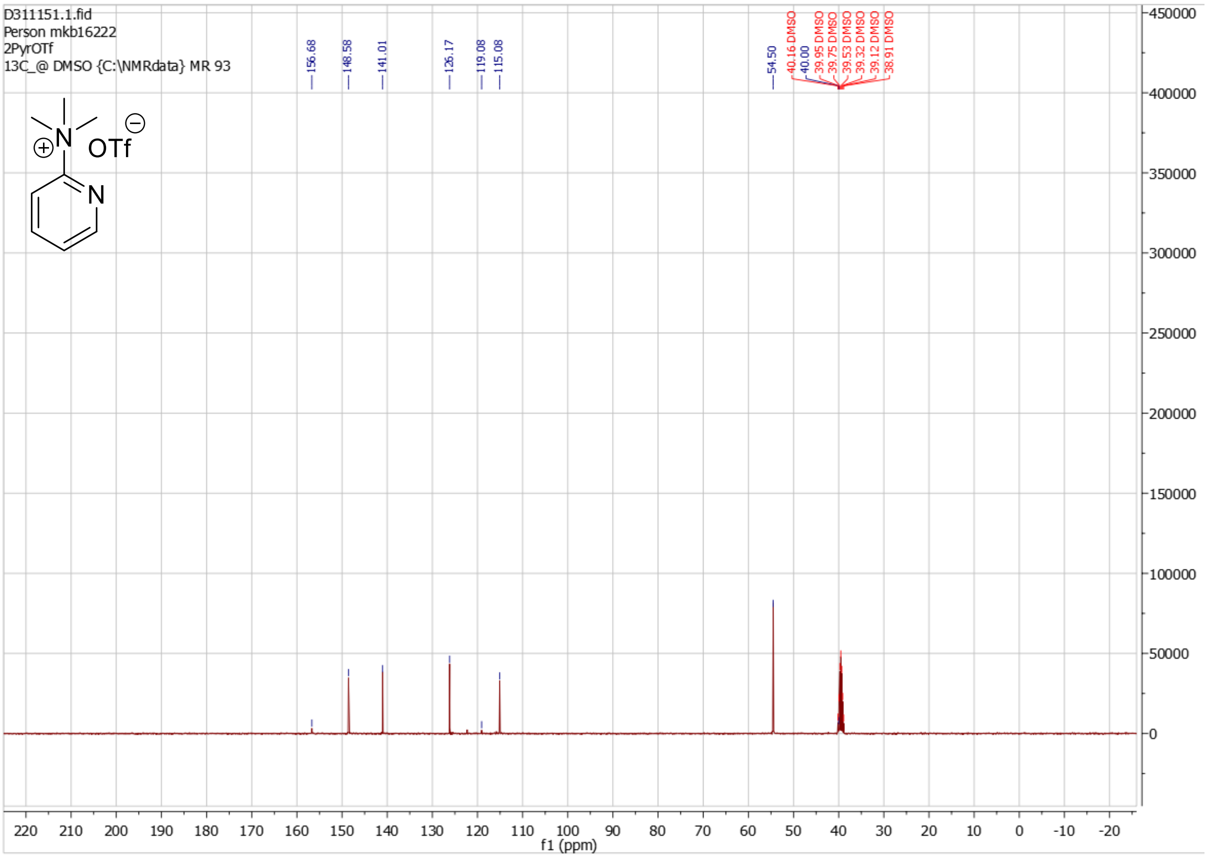
D311151.1.fid
Person mkb16222
2PyrOTf
13C_@ DMSO {C:\NMRdata} MR 93
156.68
148.58
141.01
126.17
119.08
115.08
54.50
OTf
N
f1 (ppm)

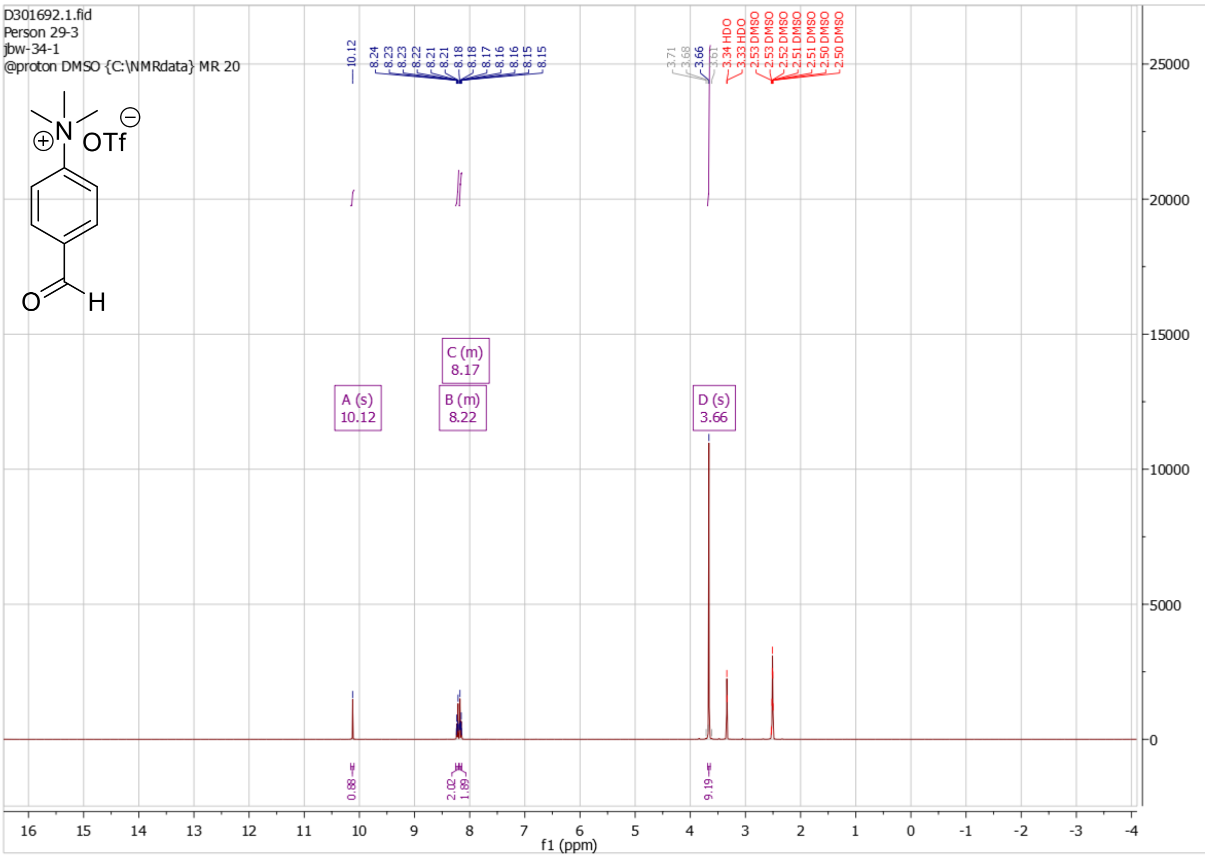
D301692.1.fid
Person 29-3
jbw-34-1
@proton DMSO {C:\NMRdata} MR 20
OTf
A (s) 10.12
B (m) 8.22
C (m) 8.17
D (s) 3.66
f1 (ppm)

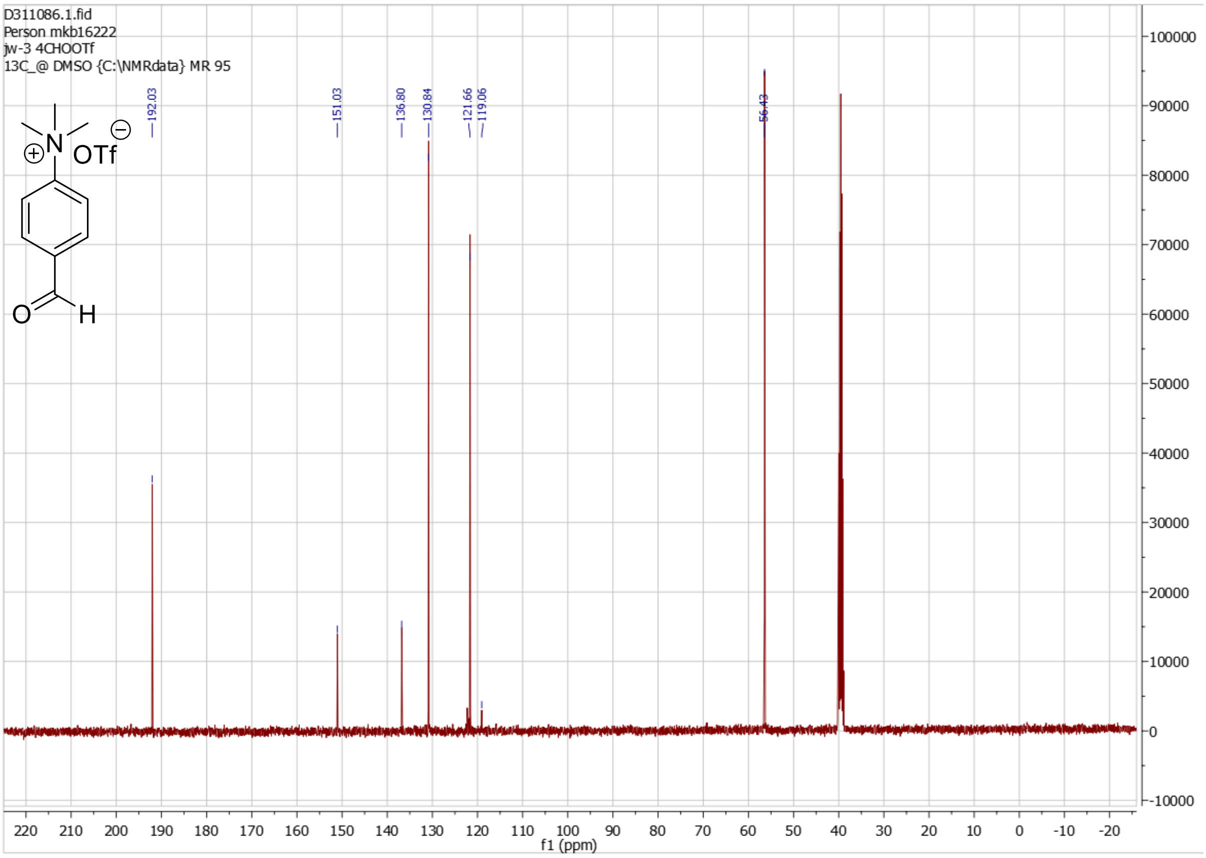
D311086.1.fid
Person mkb16222
jw-3 4CHOOTf
13C_@ DMSO {C:\NMRdata} MR 95
OTf
192.03
151.03
136.80
130.84
121.66
119.06
56.43
f1 (ppm)

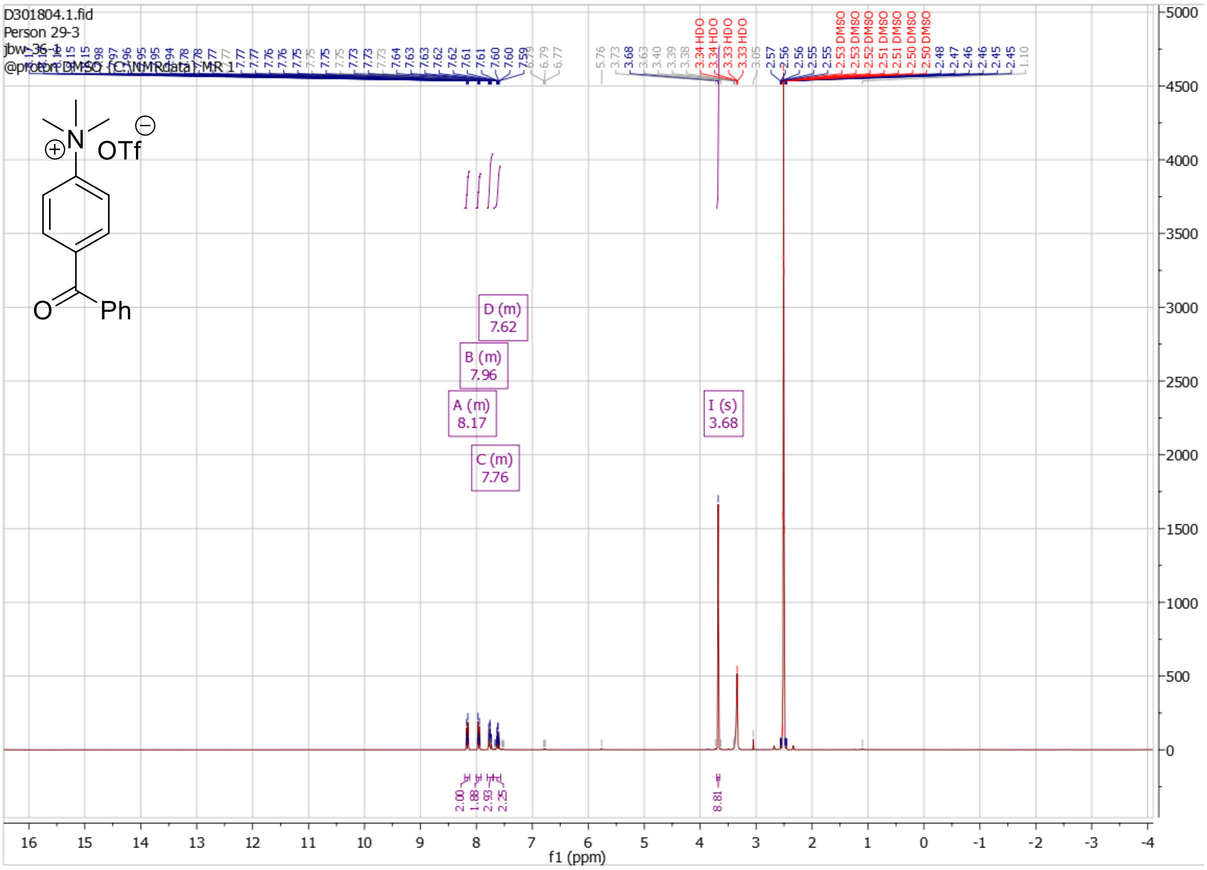

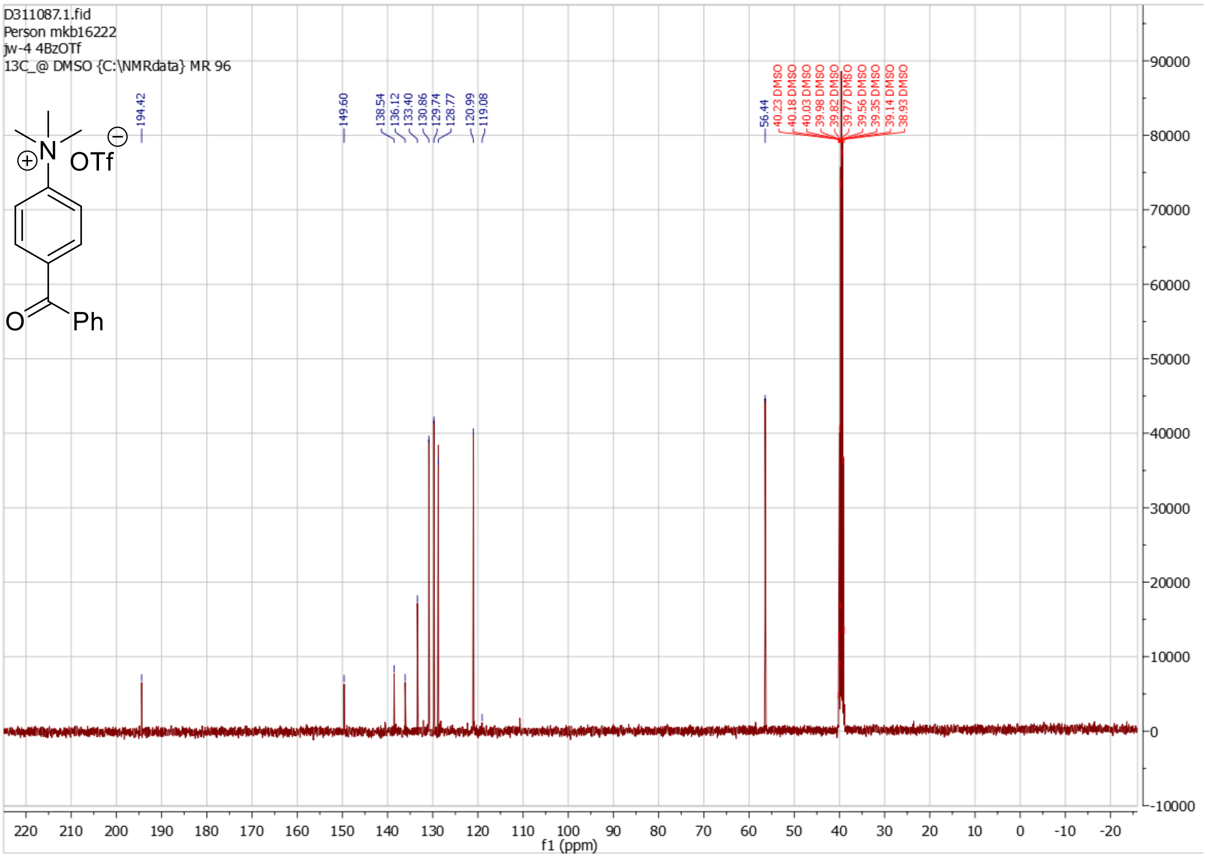

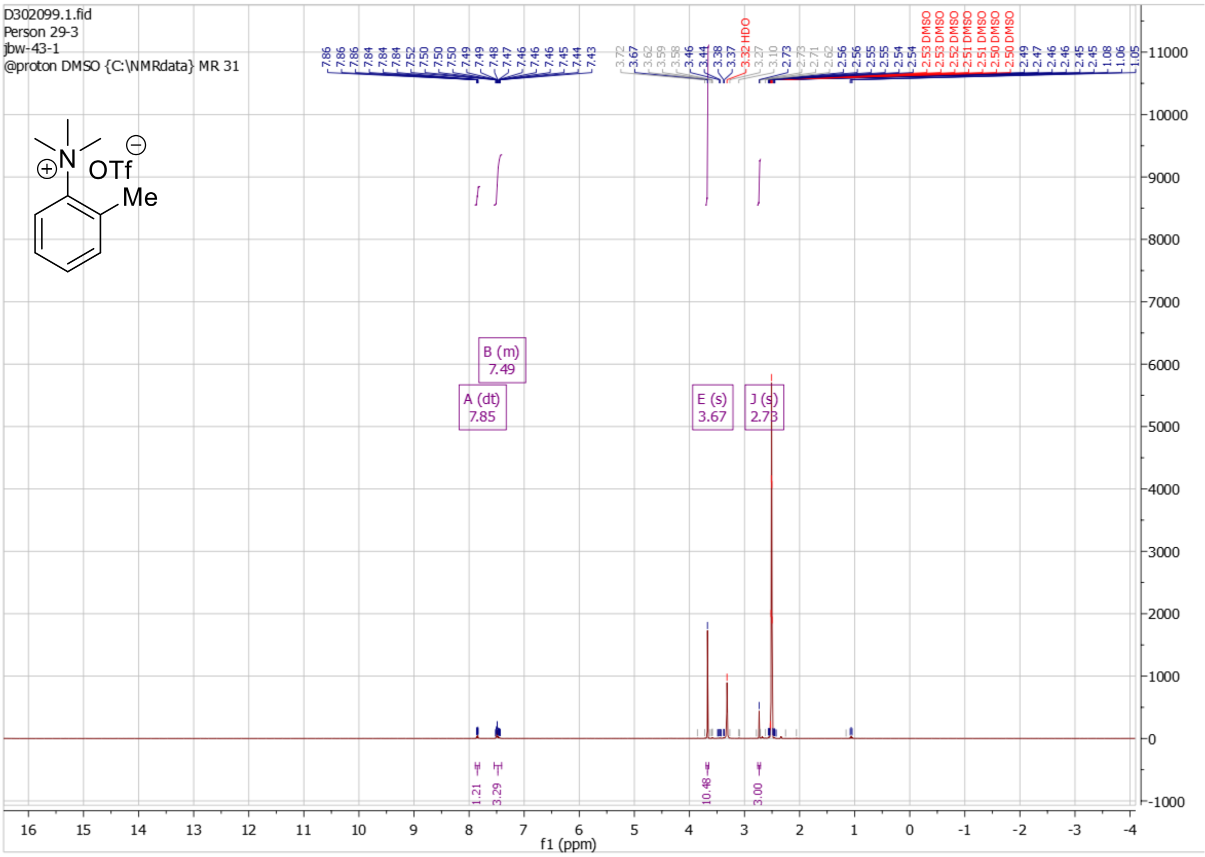
D302099.1.fid
Person 29-3
jbw-43-1
@proton DMSO {C:\NMRdata} MR 31
OTf
Me
B (m)
7.49
A (dt)
7.85
E (s)
3.67
J (s)
2.73
f1 (ppm)

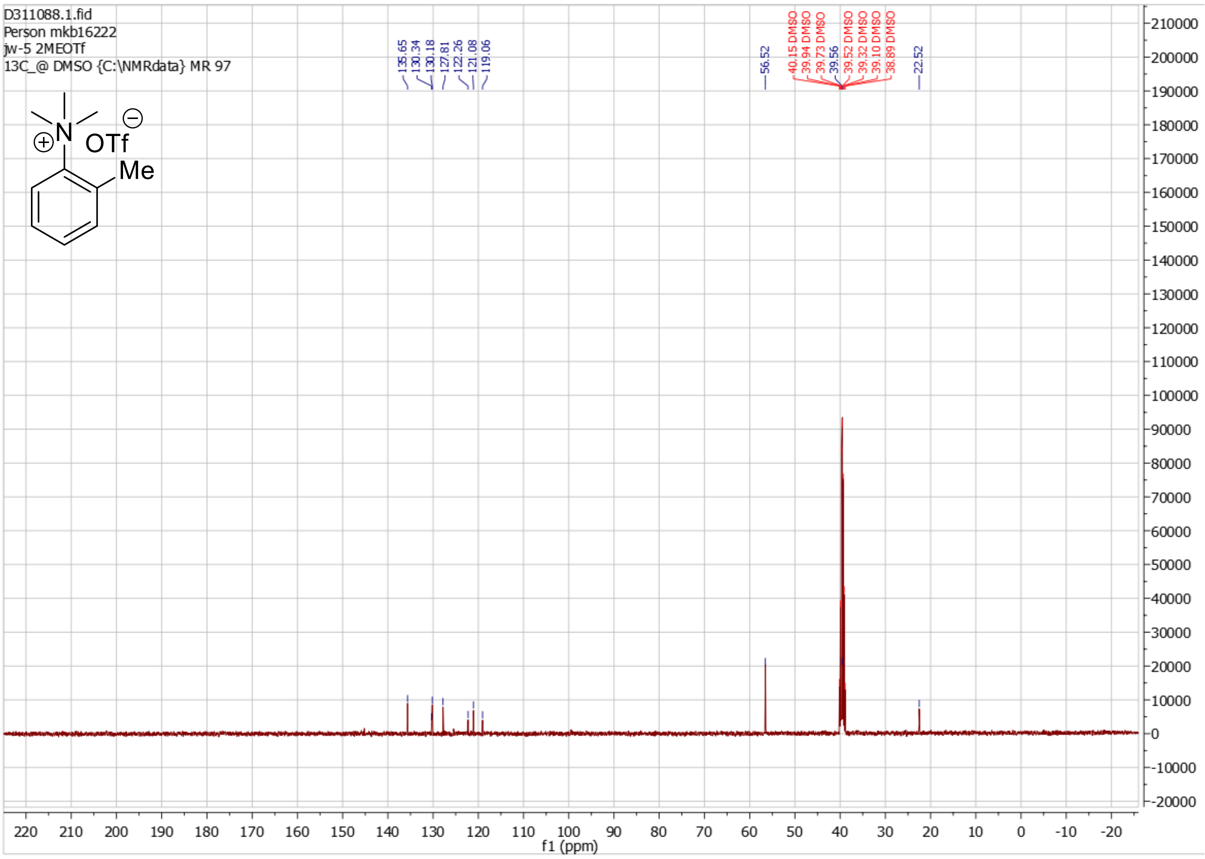
D311088.1.fid
Person mkb16222
jw-5 2MEOTf
13C_@ DMSO {C:\NMRdata} MR 97
OTf
Me
135.65
130.34
130.18
127.81
122.26
121.08
119.06
56.52
22.52
f1 (ppm)

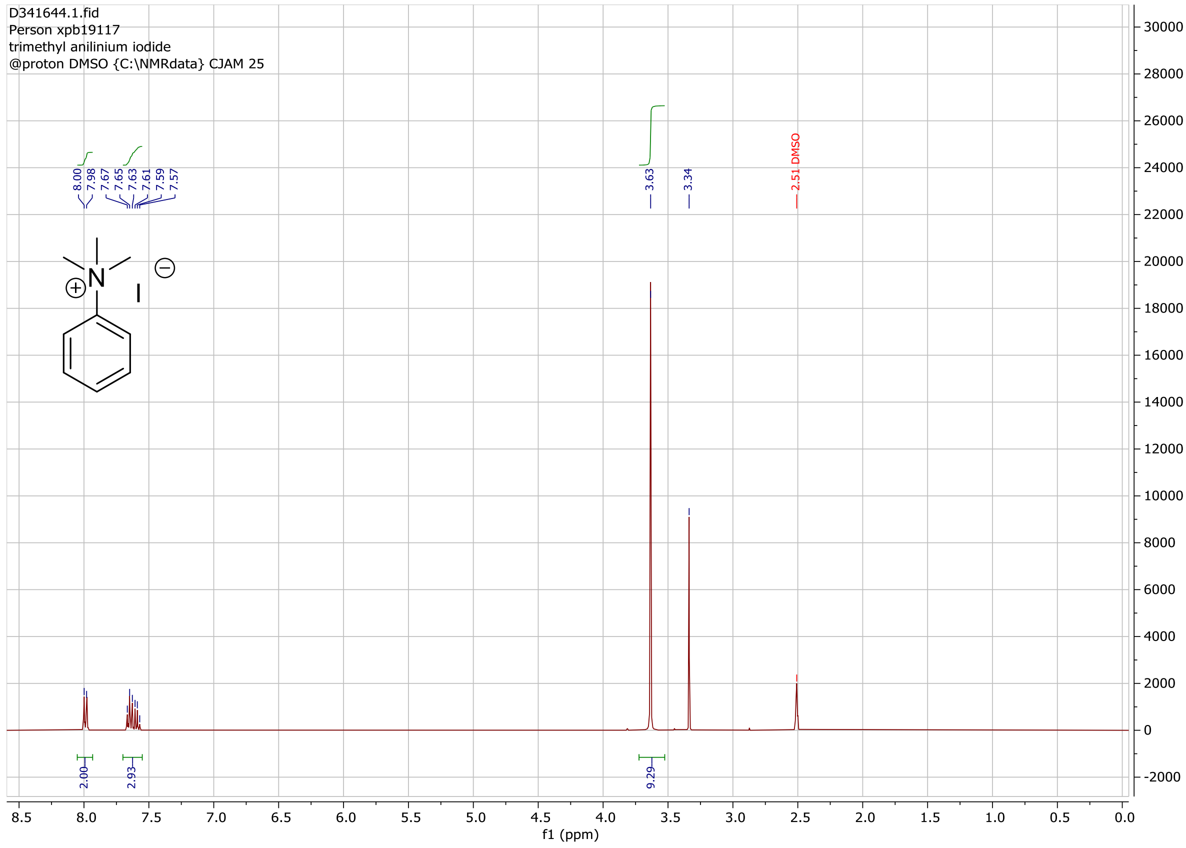
D341644.1.fid
Person xpb19117
trimethyl anilinium iodide
@proton DMSO {C:\NMRdata} CJAM 25
8.00
7.98
7.67
7.65
7.63
7.61
7.59
7.57
3.63
3.34
2.51 DMSO
2.00
2.93
9.29
f1 (ppm)

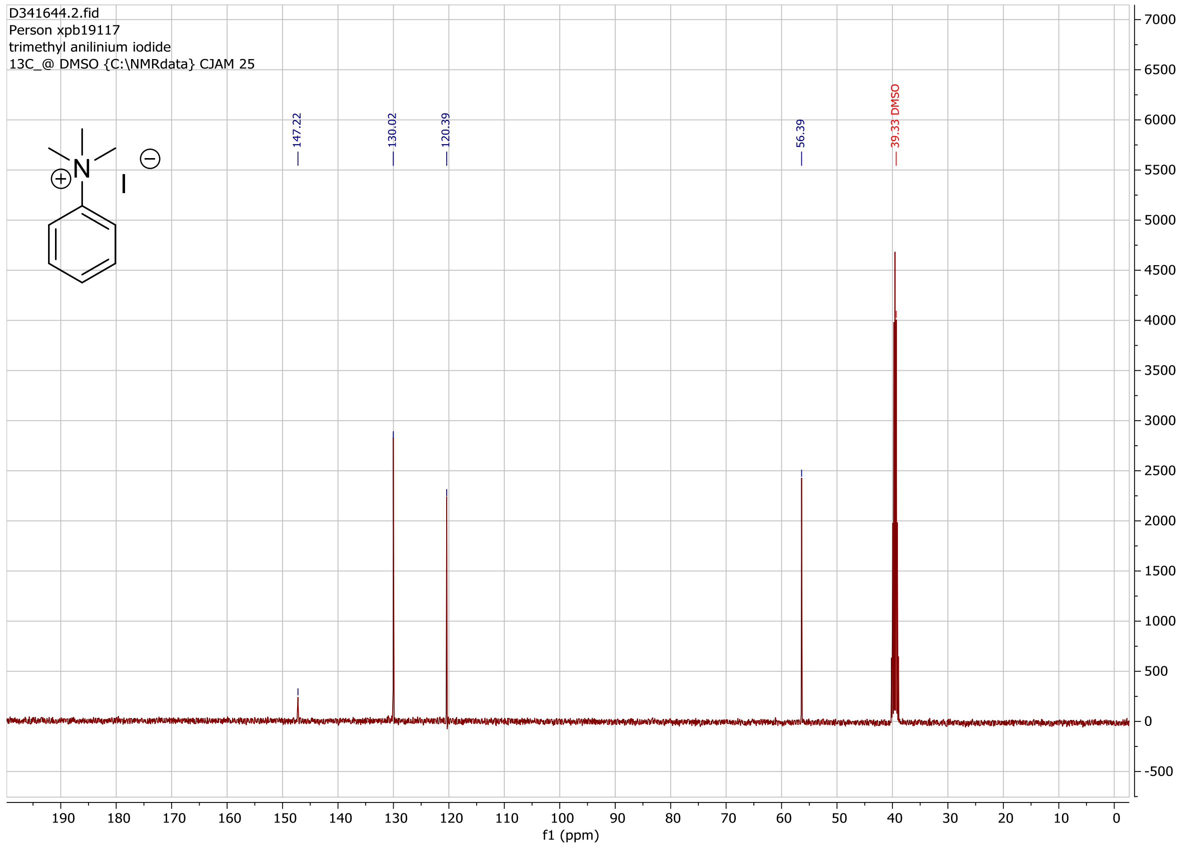
D341644.2.fid
Person xpb19117
trimethyl anilinium iodide
13C_@ DMSO {C:\NMRdata} CJAM 25
147.22
130.02
120.39
56.39
39.33 DMSO
f1 (ppm)

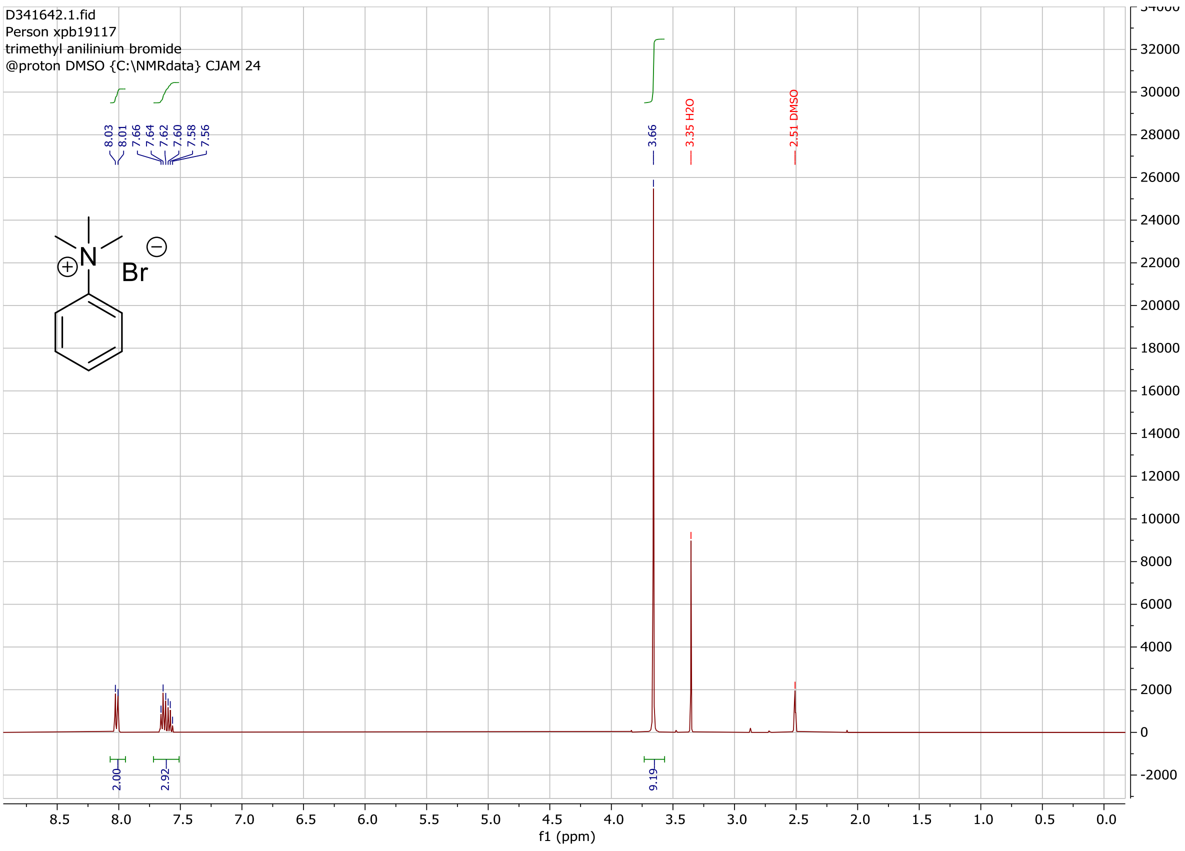
D341642.1.fid
Person xpb19117
trimethyl anilinium bromide
@proton DMSO {C:\NMRdata} CJAM 24
8.03
8.01
7.66
7.64
7.62
7.60
7.58
7.56
3.66
3.35 H2O
2.51 DMSO
N
Br
2.00
2.92
9.19
f1 (ppm)

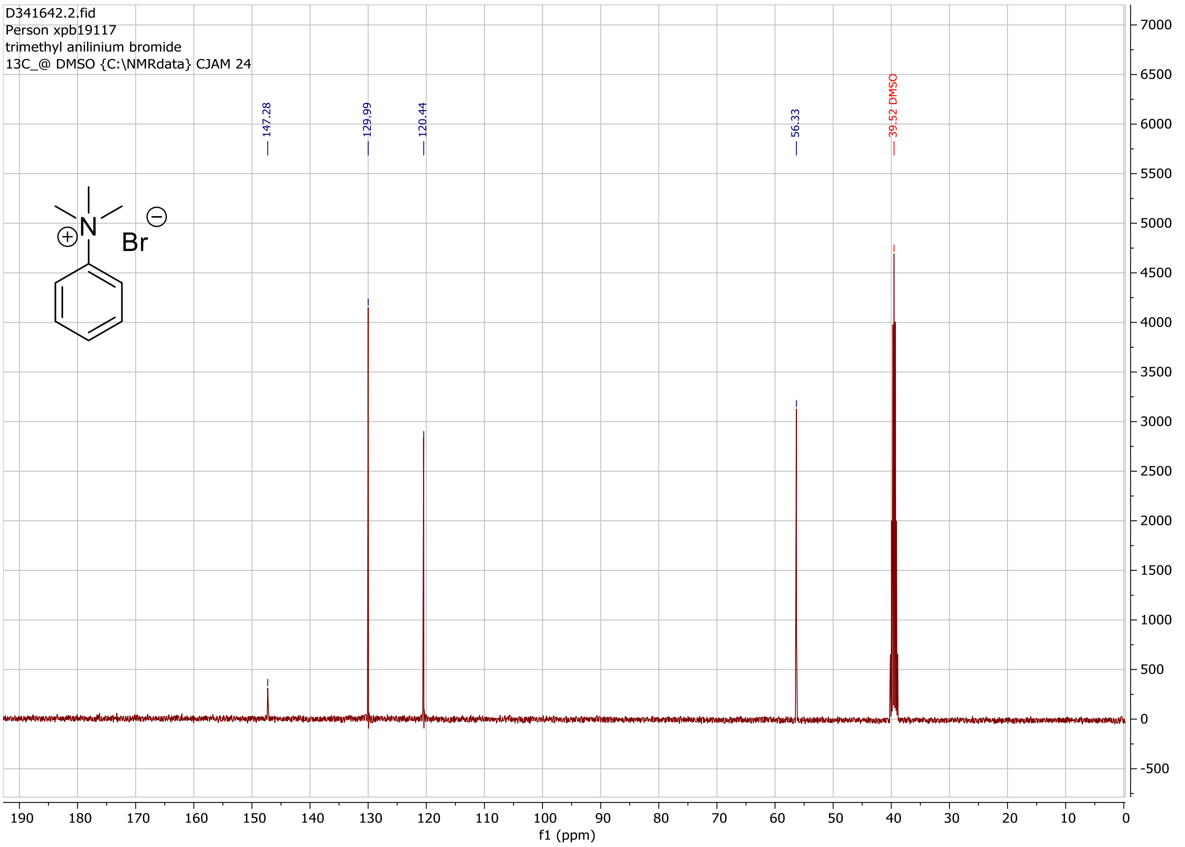
D341642.2.fid
Person xpb19117
trimethyl anilinium bromide
13C_@ DMSO {C:\NMRdata} CJAM 24
147.28
129.99
120.44
56.33
39.52 DMSO
N
Br
f1 (ppm)

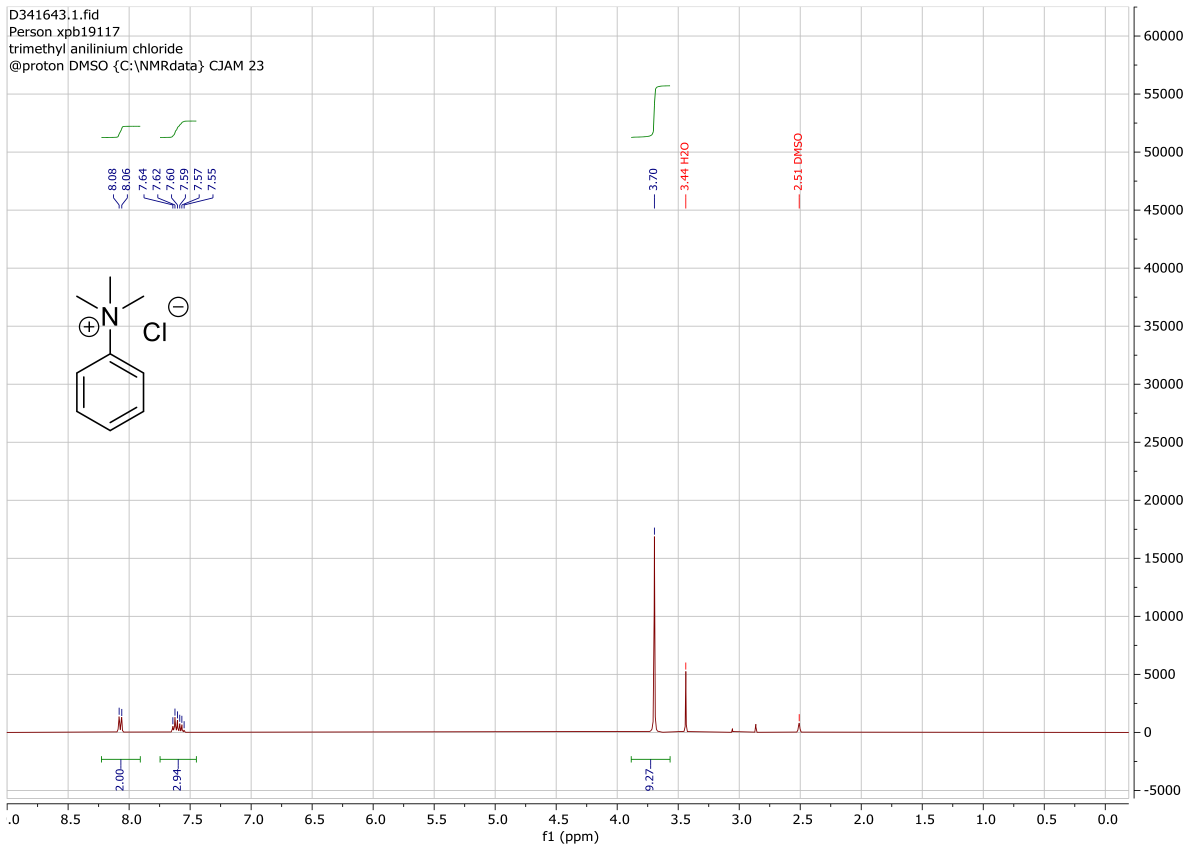
D341643.1.fid
Person xpb19117
trimethyl anilinium chloride
@proton DMSO {C:\NMRdata} CJAM 23
8.08
8.06
7.64
7.62
7.60
7.59
7.57
7.55
3.70
3.44 H2O
2.51 DMSO
2.00
2.94
9.27
f1 (ppm)
Cl

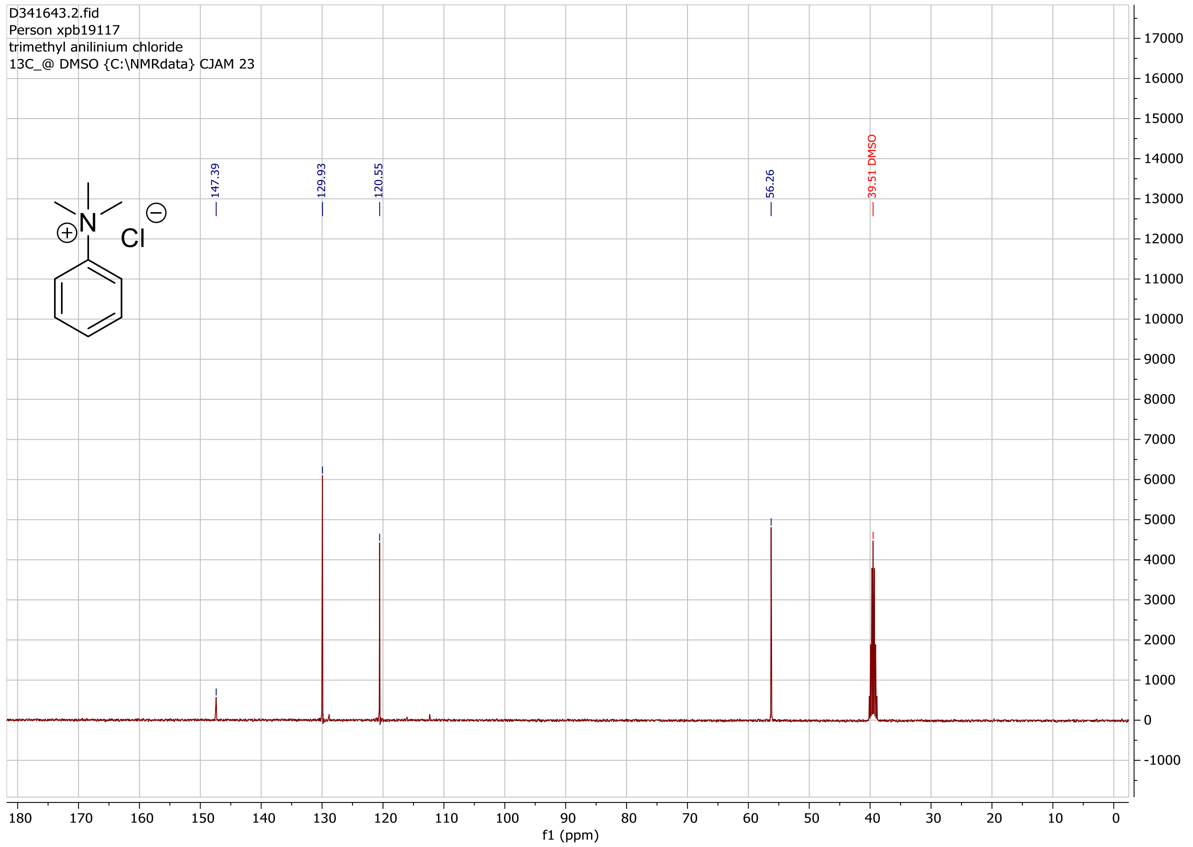
D341643.2.fid
Person xpb19117
trimethyl anilinium chloride
13C_@ DMSO {C:\NMRdata} CJAM 23
147.39
129.93
120.55
56.26
39.51 DMSO
f1 (ppm)
Cl
